# Genome and epigenome analysis of monozygotic twins discordant for congenital heart disease

**DOI:** 10.1186/s12864-018-4814-7

**Published:** 2018-06-04

**Authors:** Guoliang Lyu, Chao Zhang, Te Ling, Rui Liu, Le Zong, Yiting Guan, Xiaoke Huang, Lei Sun, Lijun Zhang, Cheng Li, Yu Nie, Wei Tao

**Affiliations:** 1 Key Laboratory of Cell Proliferation and Differentiation, School of Life Sciences, Peking University, Beijing, 100871 China; 2 Department of Cardiovascular Surgery, Center for Cardiovascular Regenerative Medicine, Fuwai Hospital, Peking Union Medical College, Chinese Academy of Medical Sciences, Beijing, 100871 China; 3 Center for Bioinformatics, School of Life Sciences, Peking University, Beijing, 100871 China

**Keywords:** CHD, MZ twins, *ZIC3*, *NR2F2*, DNA methylation, RRBS, WGS

## Abstract

**Background:**

Congenital heart disease (CHD) is the leading non-infectious cause of death in infants. Monozygotic (MZ) twins share nearly all of their genetic variants before and after birth. Nevertheless, MZ twins are sometimes discordant for common complex diseases. The goal of this study is to identify genomic and epigenomic differences between a pair of twins discordant for a form of congenital heart disease, double outlet right ventricle (DORV).

**Results:**

A monoamniotic monozygotic (MZ) twin pair discordant for DORV were subjected to genome-wide sequencing and methylation analysis. We identified few genomic differences but 1566 differentially methylated regions (DMRs) between the MZ twins. Twenty percent (312/1566) of the DMRs are located within 2 kb upstream of transcription start sites (TSS), containing 121 binding sites of transcription factors. Particularly, *ZIC3* and *NR2F2* are found to have hypermethylated promoters in both the diseased twin and additional patients suffering from DORV.

**Conclusions:**

The results showed a high correlation between hypermethylated promoters at *ZIC3* and *NR2F2* and down-regulated gene expression levels of these two genes in patients with DORV compared to normal controls, providing new insight into the potential mechanism of this rare form of CHD.

**Electronic supplementary material:**

The online version of this article (10.1186/s12864-018-4814-7) contains supplementary material, which is available to authorized users.

## Background

Congenital heart disease (CHD) is the leading non-infectious cause of death in infants. In Asia, CHD occurs in 9.3 per 1000 live births [1]. Double outlet right ventricle (DORV), defined when both great arteries originate from the morphological right ventricle in a heart, is a rare form of congenital heart disease, accounting for 1–3% of all CHD cases [2–4]. Multiple factors have been identified in contributing to the disease, of which both genetic and epigenetic changes and the interplay between them and the related environment play a key role in the pathogenesis [5, 6].

During the normal development of the heart, the outflow tract initially connects exclusively with the primitive right ventricle and must remodel to divide into a separate pulmonary artery and aorta; subsequently, there is continued remodeling to establish direct continuity from the left ventricle to the aorta [4]. In double outlet right ventricle, drainage of the left ventricle is commonly achieved through a ventricular septal defect (VSD) at different locations and with varying relations to the pulmonary and aortic outflow tract. An insufficient mitral valve and an atrial septal defect (ASD) can be found in DORV cases [3]. Since temporal and spatial expression of transcription factors (TFs) is a major determinant of cell lineage specification and patterning of the heart [7–10], mutations or expression dysregulation in them can impair cardiac development and lead to congenital heart malformations and dysfunction [7]. Changes of epigenetic modifications also affect the expression patterns of these TFs [11]. Environmental alterations or stresses including drugs may perturb the TFs-related transcriptional and epigenetic programs in the process of cardiac development and give rise to CHD [12–14].

Cytosine methylation, an epigenetic modification, is essential in mammalian development and particularly in cell-lineage specification [15, 16]. Although each individual’s genome is fixed throughout life and across cell types, epigenetic modifications are plastic and influence the temporal and spatial pattern of gene expression [14]. Cell type-specific DNA methylation patterns emerge during development and play a role in gene expression by directing chromatin activation or interfering with the binding of TFs [17]. Emerging evidence suggests that DNA methylation is responsive to both physical and social environments during pregnancy and early life [14, 18].

Monozygotic (MZ) twins share nearly all of their genetic variants before and after birth. Nevertheless, MZ twins are often discordant for common complex diseases, such as type 1 diabetes (T1D; 61%), type 2 diabetes (41%) [19], autism (58 to 60%) [20, 21], schizophrenia (58%), and different types of cancer (up to 16%) [22, 23]. These observations support the model that for many complex traits, genotype alone may not fully determine phenotypic variation, and the interplay between genes and environment needs to be considered and epigenetics has been proposed to be one of the main mediators of this interaction [24–26]. Therefore, disease-discordant MZ twin pairs provide an ideal model for examining epigenetic functions in diseases due to their shared genetic and environmental factors during the pregnancy [24]. Analysis of discordant MZ twins has been successfully used to study epigenetic mechanisms in aging, cancer, autoimmune disease, and psychiatric, neurological and other traits [20, 21, 23]. However, comprehensive analysis of genome-wide DNA methylation in a MZ twin pair discordant for double outlet right ventricle (DORV) is lacking.

In this study, we dissected the contributions of DNA methylation pattern to the pathogenesis of DORV through DNA methylation profiling at single nucleotide level by utilizing the whole-blood-DNA derived from a MZ twin pair discordant for DORV. The two years and two months old Chinese girl, given a diagnosis of DORV at birth, has a healthy MZ twin sister. Their parents have no past or family history of heart diseases and are not consanguineous. Thus, we hypothesize that genome-wide analysis in this phenotype-discordant twins will provide insights into genetic and epigenetic factors affecting normal heart development.

## Results

### Genome sequence differences in MZ twins discordant for DORV

**Fig. 1:**
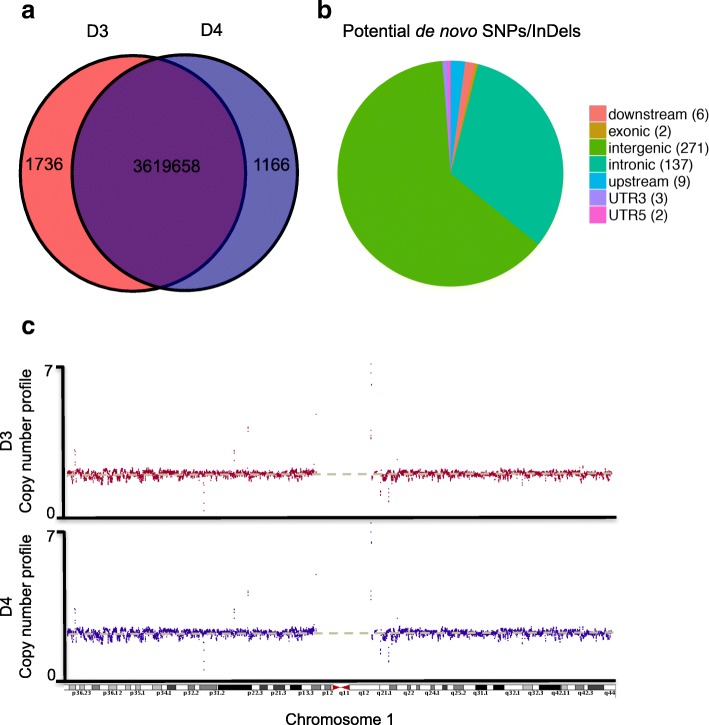
Overview of whole genome sequencing. **a** The numbers of SNVs and InDels detected in D3 and D4, 99.9% loci shared between the MZ twins. **b** There are 1736 SNVs or InDels specific in the disease sample D3, of which only 430 loci showing high confidence confirmed by Varscan are regarded as potential de novo SNVs/InDels. All loci are classified into 7 categories (upstream, downstream, intergenic, exonic, intronic, UTR5, UTR3) according to the relative position of nearby genes. The pie plot shows the number of loci and proportion of each category. **c** Normalized copy number profiles of D3 and D4. Each point shows a 5 kb window (chromosome 1) of sequencing reads normalized by GC-content and mappability using Control-FREEC

We first tried to identify de novo genomic sequence variation through whole genome sequencing. DNA extracted from whole blood samples of the MZ twins discordant for DORV (septal-defect heart (D3) and normal heart (D4) was sequenced by Illumina HiSeq X-Ten (150 bp paired-end reads) and aligned to the hg19 reference human genome. The average sequencing depth of D3 and D4 were 31.9 and 28.5 respectively, and 4-fold coverage represented more than 91% of the hg19 human reference genome (Additional file 1: Table S1). Single-nucleotide variations (SNVs) and short insertions/deletions (InDels) were identified using SAMtools [27] and filtered by Varscan [28]. More than 99.9% SNVs shared between the MZ twins (Fig. 1a). Three hundred and sixteen SNVs and 114 InDels were identified specifically in D3. After filtering by the dbSNP (Version 138) database, forty-one SNVs and 71 InDels in D3 remained (Additional file 2: Table S2). There was one deletion located in non-coding RNA (ANKRD30BP2, pseudogene) and one synonymous SNV in the exon of DSPP gene, but none of the SNVs or InDels altered proteins (Fig. 1b & Additional file 2: Table S2). No pathogenic variation specific to D3 was detected when filtered by the Online Mendelian Inheritance in Man (OMIM) and Human Genetic Mutation Database (HGMD). Sequence variants in promoter of *GATA4* and *TBX1*, which were closely related to VSD [29, 30], were found in both of our samples, indicating that the sequences variants in the promoters of these two transcription factor may not contribute to the pathogenesis of DORV in D3 (Additional file 3: Table S3).

Copy number variants (CNVs) are deletions or amplifications of DNA segments that arise from inappropriate chromatid recombination or segregation during cell division. As CNVs alter the gene expression dosage of contiguous genes, they may result in syndromic CHDs [31]. Using Control-FREEC [32], we detected 15 focal CNVs; however, these CNVs did not pass CNVnator confirmation criteria [33], suggesting the absence of bona fide CNVs (Fig. 1c; Additional file 4: Figure S1 & Additional file 5: Table S4). Structural variation (SV), typically a large insertion/deletion, inversion or translocation affecting a sequence length from 1 kb to 3 Mb, was not detected to be different between the two samples using the CREST software (Additional file 6: Figure S2). Together, the genomic analyses suggest that the DORV in D3 is not likely caused by genome alterations.

### DNA methylation differences between the MZ twin pair

**Fig. 2:**
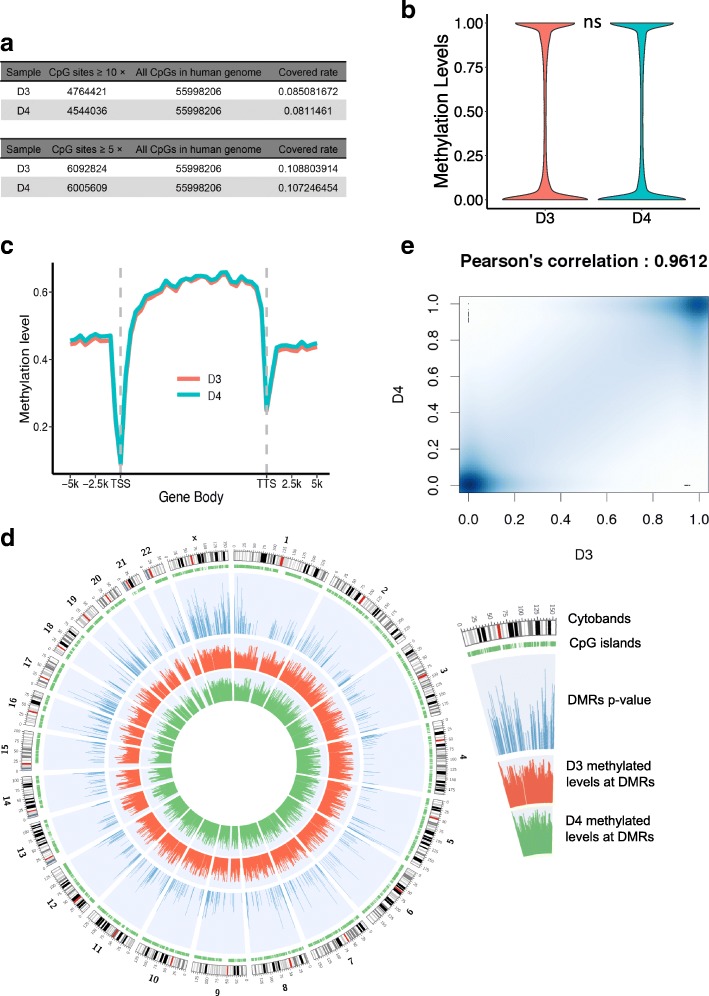
Comparison of CpG methylomes between two samples. **a** Overall reads coverage of reduced representation bisulfite sequencing (RRBS) at CpGs between the twins. The RRBS strategy shows a high covered rate of CpGs in two samples. 10.88% and 10.72% of all human CpGs are sequenced by more than 5 reads (5×) in D3 and D4, respectively. **b** Overall distribution of CpGs’ methylated levels in D3 and D4. This violin plot shows that most CpGs are 100% methylated or 0% methylated, and the global methylation level is unchanged between the pair (Wilcoxon test non-significant). **c** Average CpGs’ methylation profile in gene body, upstream (− 2 kb of TSS) and downstream (+ 2 kb of TTS). **d** Circos plot shows the distribution of the DMRs along the genome. The tracks from outsider to inner: CpG islands downloaded from UCSC (green); the –log10 *p*-value of detected DMRs (blue); the average methylation levels per 1 kb in D3 (red); the average methylation levels per 1 kb in D4 (green). **e** Scatter diagram of all CpGs’ methylation levels calculated by RRBS data between two samples, D3 and D4, showing a similar overall CpGs methylation levels with Pearson correlation coefficient equal to 0.9613

DNA methylation is involved in multiple processes, including regulation of gene expression, silencing of retrotransposons, genomic imprinting, X-chromosome inactivation and occurrence of various diseases [34, 35]. We next sought to compare genome-scale DNA methylation profiles between the MZ twins at nucleotide resolution. DNA samples were extracted from whole blood acquired before heart surgery, and gel-free reduced representation bisulfite sequencing (RRBS) libraries were constructed as previously reported [36] and then sequenced on Illumina HiSeq2000 platform (100 bp paired-end reads). After aligning the reads to the hg19 reference genome, we obtained 56 million and 48 million high-quality, 100-nucleotide, uniquely mapped reads from the D3 and D4 samples, respectively. The two samples had very similar sequencing-depth patterns of cytosine sites and covered more than 34 million C sites with sequencing depth at least 5 fold (Additional file 7: Table S5). More than 6 million high-quality CpG dinucleotides were supported by at least by 5 reads (depth ≥ 5×), which covered 10% of all CpG sites (Fig. 2a). In addition, we checked the methylation status of samples from blood cell and heart tissue in ENCODE project (Accession ID: ENCFF388NTJ, ENCFF107RMQ). We found a high correlation between B cell and heart with Pearson’s correlation coefficient equal to 0.9238, and visualizing the genome-wide methylation between them in IGV showed similar patterns (Additional file 8: Figure S3), suggesting that the DNA methylation pattern in blood correlate with that in heart.

The methylation levels of CpG dinucleotides in both samples showed a bimodal distribution with most CpG sites being unmethylated or extensively methylated (Fig. 2b). As expected, genome-wide patterns of DNA methylation (CpG and non-CpG) were highly correlated between the MZ twin pair (Figs. 2c-e & Additional file 9: Figure S4), suggesting that DORV is not associated with a global reprogramming of methylation.

**Fig. 3:**
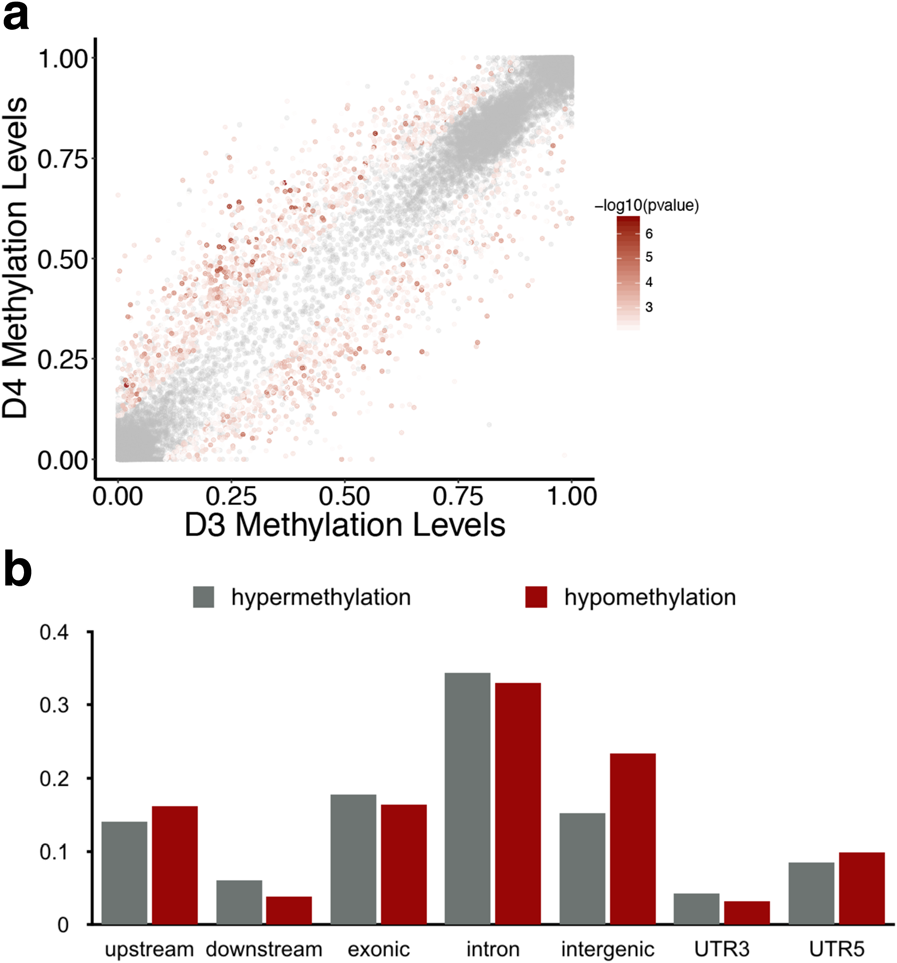
Distribution of D3 specific DMRs. **a** The scatter plot shows the found DMRs, the red color indicates the *p*-value, and non-significant regions are showed in gray. **b** The distribution of significantly DMRs. All DMRs are classified into 2 main categories (hypermethylation and hypomehtylation), and each category is further classified into 7 classes (upstream, downstream, intergenic, exonic, intronic, UTR5, UTR3) according to the relative position of nearby genes. The bar plot shows the proportion of each class. There are 294 genes that have a differentially methylated region in upstream (2 kb) of transcription start site

Next, we sought to identify differentially methylated regions (DMRs) between the twin pair using a window sliding strategy to reduce the sampling variation of individual CpG sites (see Methods). We identified 1566 significant DMRs between the MZ twin pair (Fig. 3a). Hypermethylation and hypomethylation DMRs in D3 showed similar genomic distributions, with 80% (1254/1566) of the DMRs distributed in gene bodies or intergenic regions and 20% (312/1566) of the DMRs located within 2 kb upstream of TSS (Fig. 3b and Additional file 10: Table S6). We concluded that DMRs between the MZ twins may be involved in the pathogenesis of DORV.

### Gene ontology (GO) and transcription factor binding sites enriched in DMRs

**Fig. 4:**
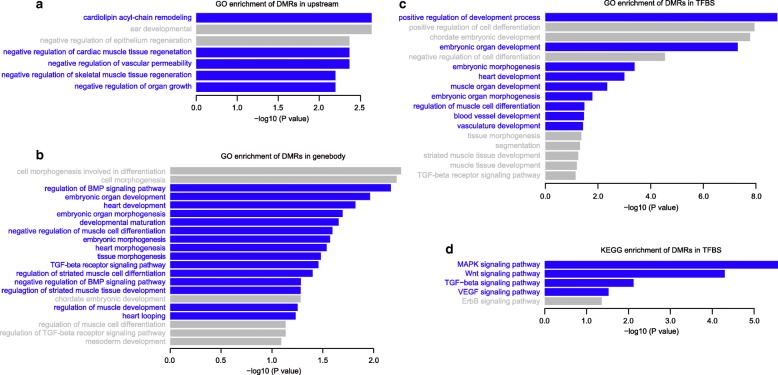
Gene Ontology (GO) and KEGG analysis of D3 specific DMRs. Gene Ontology (GO) and pathway enrichment analysis of differentially methylated regions using Enrichr and DAVID under default parameters. The bar charts show the most relevant and significantly enriched terms. Terms that are highly related to CHD are marked in blue. The x-axis represents the –log10 of the enrichment p-value. The y-axis represents the enriched terms in GO or KEGG databases. **a** GO enrichment analysis of genes associated with differentially methylated regions in 2 kb upstream of genes. **b** GO enrichment analysis of genes associated with differentially methylated regions in gene body. **c** GO enrichment analysis of TFs whose binding sites are differentially methylated. **d** KEGG pathway enrichment analysis of TFs whose binding sites are differentially methylated

We analyzed the genes that contained DMRs within 2 kb upstream of their TSS. In total, three hundred and twelve DMRs were located in the upstream of 621 TSSs (belonging to 294 genes annotated in Refseq) (Fig. 3b & Additional file 11: Table S7). These DMR-associated genes were then subjected to KEGG (Kyoto Encyclopedia of Genes and Genomes) pathway and Gene Ontology (GO) analysis using the Enrichr and DAVID Web servers [37–39]. GO analysis revealed that genes associated with cardiolipin acyl-chain remodeling, cardiac muscle tissue regeneration, vascular function and organ growth are enriched in DMR-associated genes (Fig. 4a & Additional file 11: Table S7), suggesting that DMRs may be associated with the abnormal heart development of D3.

We next analyzed the 851 genes containing DMRs in their gene bodies. These genes were also enriched in several biological processes that are involved in various stages of cardiovascular development (Fig. 4b and Additional file 12: Table S8). Furthermore, multiple signaling-pathways involved in heart development were also enriched, including regulation of BMP signaling and TGF beta receptor signaling (Fig. 4b and Additional file 12: Table S8). These results suggest that gene regulatory networks during the heart development of D3 may be affected by the DMRs.

Moreover, DMRs can influence gene expression through directly altering the binding of transcription factors to their targets. We analyzed the TF binding motifs in DMRs by using ANNOVAR (using tfbsConsSites database downloaded from UCSC). We identified 138 TFs (Additional file 10: Table S6) that belong to GATA family and NKX family, which are essential for cardiac development (Additional file 13: Table S9). GO analysis showed that many of these TFs were involved in heart development, muscle organ development, regulation of muscle cell differentiation, and blood vessel development (Fig. 4c and Additional file 13: Table S9). These TFs were also enriched in KEGG signaling pathways including MAPK signaling, Wnt signaling, TGF beta signaling, and VEGF signaling pathway (Fig. 4d and Additional file 13: Table S9), which were all involved in heart development [40–45]. Taken together, these results imply that DMRs in TFs binding sites may contribute to the DORV pathogenesis.

### Aberrant promoter methylation of *ZIC3* and *NR2F2* in the diseased twin

Since promoter hypermethylation is typically associated with the repression of gene transcription [44], we then focused on promoter DMRs for further analysis. We found that DMRs existed in the upstream of multiple genes whose family members have been reported to be involved in pathogenesis of CHDs, and their normal functions are critical in morphogenesis and establishment of the cardiovascular system during cardiac development [46–48]. These genes include *CITED1* (member of CREB-binding protein/p300-interacting transactivator with Asp/Glu-rich C-terminal domain (CITED) family of proteins, hypomethylated), *GATA2* (member of the GATA family of zinc-finger TFs, hypermethylated), *SOX3* (member of the SOX (SRY-related HMG-box) family of transcription factors, hypomethylated) (Additional file 14: Figure S5, Additional file 15: Figure S6 and Additional file 16: Figure S7).

We also found that genes encoding important epigenetic factors contained DMRs in their upstream of TSS, implying that these factors may play roles in causing CHDs through regulating expression of genes implicated in heart development. The genes include *NSD1* (Nuclear Receptor Binding SET Domain Protein 1, which preferentially methylates ‘Lys-36’ of histone H3 and ‘Lys-20’ of histone H4, hypomethylated), *MTA2* (Metastasis Associated 1 Family, Member 2, a component of NuRD, hypomethylated), *MECP2* (Methyl CpG Binding Protein 2, hypermethylated) and *SUV39H1* (Suppressor of Variegation 3–9 Homolog 1, a histone methyltransferase that trimethylates lysine 9 of histone H3, hypomethylated) (Additional file 17: Figure S8, Additional file 18: Figure S9, Additional file 19: Figure S10 and Additional file 20: Figure S11). We also utilized the public expression profiling data of embryo and adult heart from ENCODE (Accession ID: ENCFF704AHC, ENCFF199GQY, ENCFF987YOV) to analyze the possible contribution of these genes to DORV. We found that these genes were expressed in both embryo and heart (Additional file 21: Table S10), suggesting that these genes may play important roles in embryonic and cardiac development and dysregulated expression of them may contribute to CHD such as DORV.

**Fig. 5:**
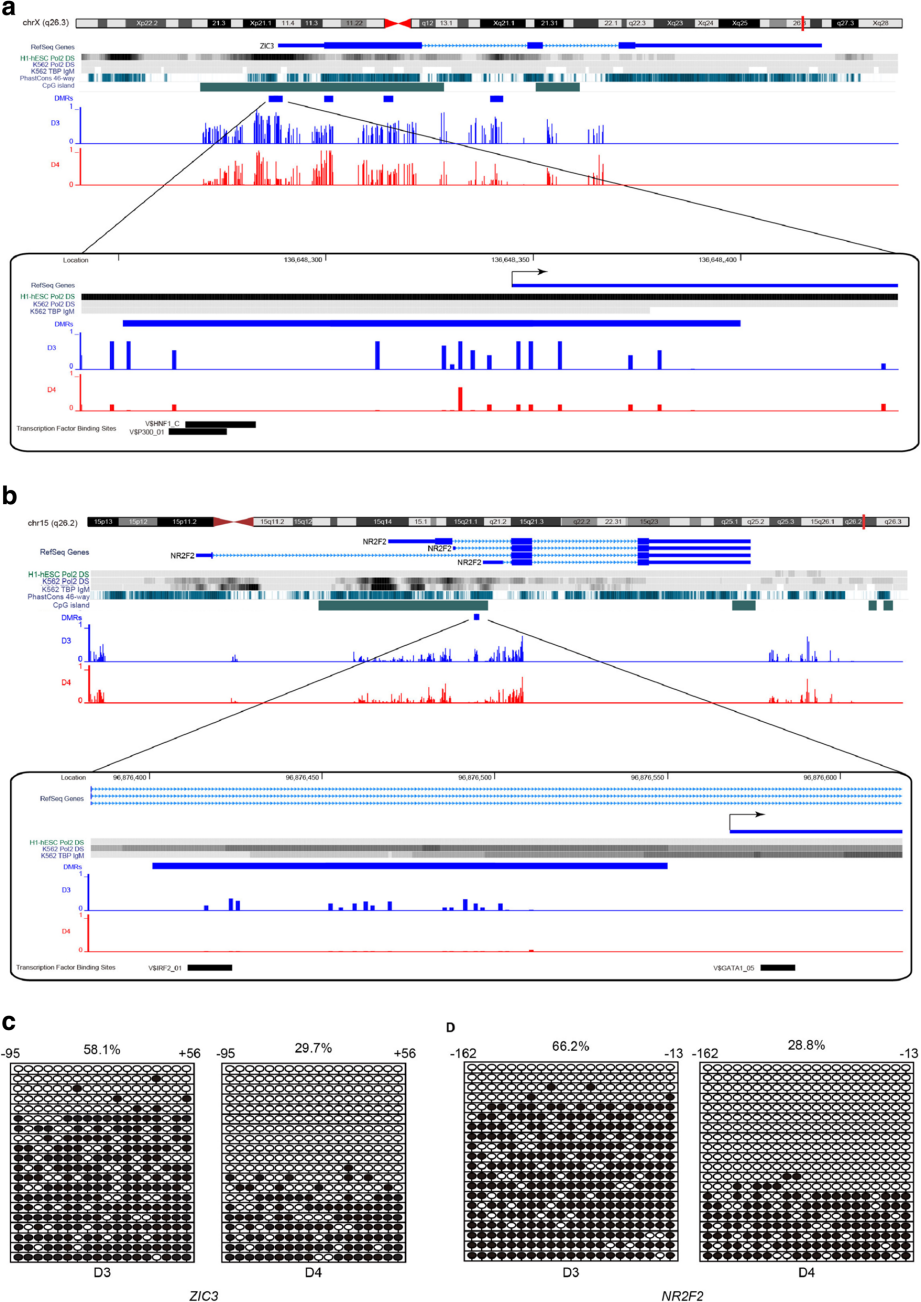
Aberrant methylation in the upstream regions of *ZIC3* and *NR2F2,* visualized in UCSC genome browser. DMRs are indicated by light blue bar; methylated levels in the twins are showed in blue (D3) and red (D4) bars. Transcription factor binding sites are also showed in zooming-in panels, which are indicated by black bars with names marked in front. An arrow gives TSS and transcriptional orientation. Two genes, *ZIC3* (**a**) and *NR2F2* (**b**), are showing differentially methylated in the upstream of TSS, and these two genes are known be associated with CHD. Transcription factor binding sites analyses are performed by ChIP-seq data of RNA Pol II and TBP derived from ENCODE. Bisulfite sequencing data (**c** and **d**) show methylation status of the same region of *ZIC3* and *NR2F2* as in (**a**) and (**b**). Methylated and unmethylated CpG sites are shown as black and white circles, respectively

Moreover, by scrutinizing all the DMRs located in gene promoter regions, we noticed that two genes, *ZIC3* and *NR2F2*, encode TFs annotated with CHD in OMIM (300,265 and 107,773) [49–51]. In the DORV diseased twin D3, the upstream of *ZIC3* (harboring P300 and HNF1 binding sites) was hypermethylated, corresponding to a region with high Pol II binding density in hESC cells (ENCODE data) (Fig. 5a and c). Similarly, hypermethylation of *NR2F2* was detected in the upstream of the TSS (harboring an IRF2 binding site) of the shortest *NR2F2* transcript variant, corresponding to a region with high intensity of TBP and Pol II binding in K562 cells (ENCODE data) (Fig. 5b and d). These results suggested a possible association of hypermethylated promoters of *ZIC3* and *NR2F2* and their functions during the heart development of DORV patients.

### Hypermethylation and dysregulation of *ZIC3* and *NR2F2* in additional DORV patients

**Fig. 6:**
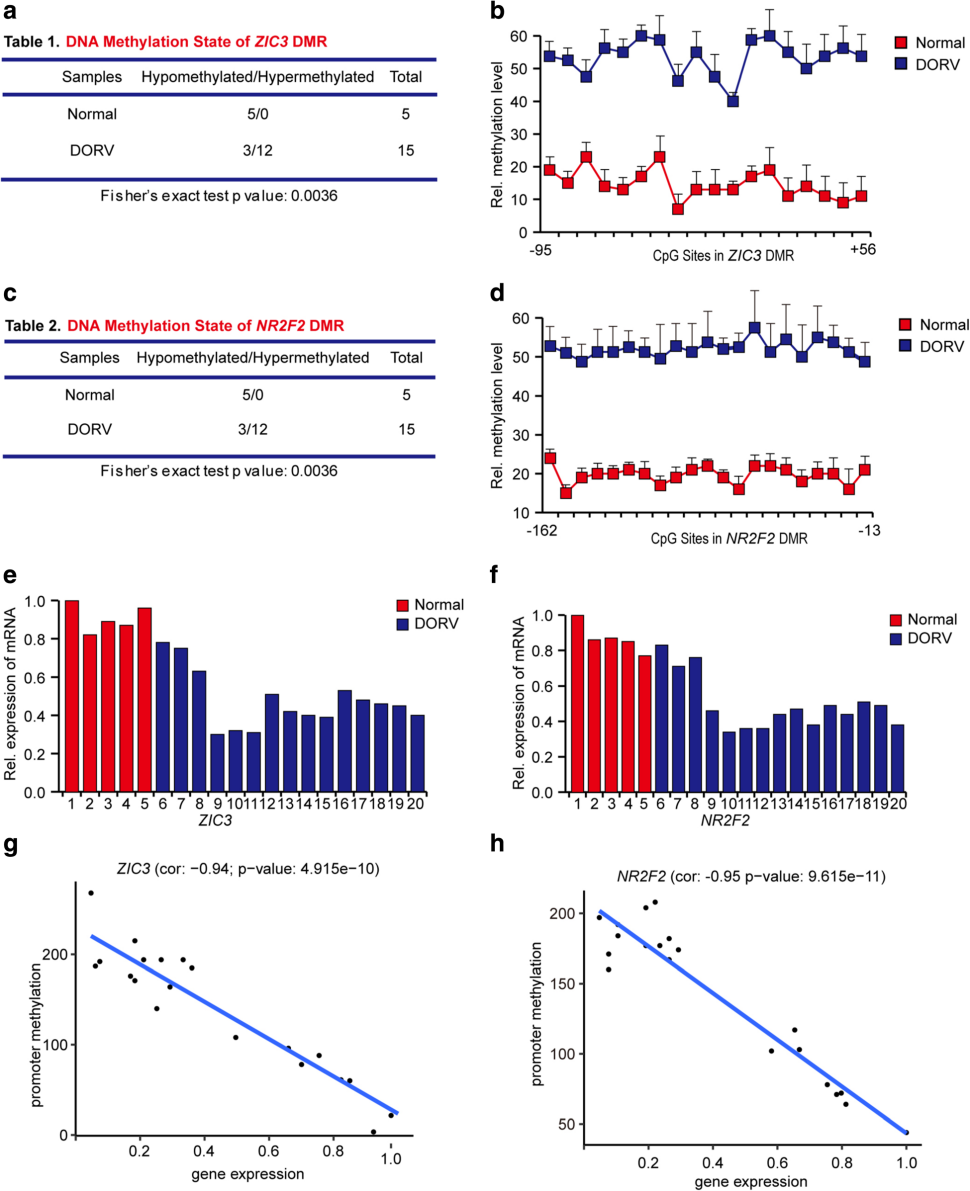
DNA methylation and gene expression detection of *ZIC3* and *NR2F2* from clinical cases. (**a** and **c**) Statistical summaries about DNA methylation status of DMRs in *ZIC3* and *NR2F2* in 20 clinical samples, consisting of five normal providers and fifteen DORV patients. (**b** and **d**) Diagrams exhibiting average methylated levels of individual CpG sites in DMRs of *ZIC3* and *NR2F2* from the indicated groups, respectively. (**e** and **f**) Histograms showing relative gene expression levels of *ZIC3* and *NR2F2* in different groups of specimens. (**g** and **h**) Scatterplots showing the gene expression levels of *ZIC3* (**g**) and *NR2F2* (**h**) are negatively correlated with their promoter methylation status. Pearson’s correlation coefficient and *p*-values were listed above the plot

In order to further confirm the dysregulation of *ZIC3* and *NR2F2* in DORV pathogenesis, we collected twenty DNA samples of whole blood from normal individuals and clinical DORV patients. In order to guarantee the similarity in age and in gender ratio between the normal group and the patient cases, the samples included five controls with normal heart development (aged 0.8–3.8 years; 3 males, 2 females) and fifteen cases with DORV diagnosis (aged 1–3.5 years; 9 males, 6 females). Using bisulfite sequencing, we confirmed hypermethylated promoters of both *ZIC3* (Fig. 6a and b) and *NR2F2* (Fig. 6c and d) in twelve of fifteen DORV patients compared to normal subjects (Additional file 22: Figure S12, Additional file 23 Figure S13). The association of DORV and hypermethylated *ZIC3* and *NR2F2* promoters showed a significant correlation by Fisher’s exact test (*p* values are both 0.0036) (Fig. 6a and c). Correspondingly, samples harboring hypermethylated promoters of the two genes have comparatively lower gene expression levels (Fig. 6e and f), and the methylation and gene expression levels of these two genes were negatively correlated (Fig. 6g and h). These results confirm that lower gene expression levels of *ZIC3* and *NR2F2* are associated with promoter hypermethylation of these two genes in normal individuals and DORV patients.

## Discussion

This study represents the first analysis of genome and epigenome profiling of MZ twins discordant for DORV, and provides the evidence for the presence of epigenetic differences between the twin pair. Genetic variations between twins can affect proteins coding, gene transcription and epigenetic modifications [52]. We therefore first performed genomic sequence variation detection and revealed some differences between the twin pair, but stringent filtering analyses of CNVs, SNVs and short InDels failed to identify genomic differences that may contribute to pathogenic DORV. Even though sequence variants within the promoter regions of the *GATA4* and *TBX1* were reported to contribute to congenital heart disease by altering their gene expression [10, 29, 30], our results showed the sequence variants existed in both the normal and diseased twins, indicating that the pathogenesis of DORV may not be due to these differences. It is possible that the shared sequence variants showed different penetrance, which may cause the DORV in one of the MZ twins. Notably, sequences alignment in our study covered 91% of the hg19 human reference genome, leaving the rest of the genome not assessed. Therefore, we cannot rule out the possibility that genome sequence differences are involved in DORV of this twin pair.

Epigenetic variation at specific genomic regions has high heritability, and MZ twins typically share similar epigenetic profiles [25]. However, a set of factors including dietary components, physical changes, psychological states and environmental changes could affect epigenomes of the two years and two months old twins after birth [53]. In this study, many DMRs-related genes (within upstream or gene body) are enriched in pathways that contribute to cardiac development. Most importantly, we found that *ZIC3* and *NR2F2*, which are annotated with CHD in OMIM database, were hypermethylated at the TSS upstream region of the diseased twin compared to the normal heart sample. ZIC3 is a member of the ZIC family of C2H2-type zinc finger proteins, which functions as a TF in early stages of left-right body axis formation and heart development [53]. ZIC3 acts in organizer formation by inhibiting the canonical Wnt signaling pathway, and its expression is regulated by determinants of the early neural fate specification and dorsal-ventral (D-V) axis formation, including BMP, FGF, and Nodal signaling pathways [54]. Mutations in *ZIC3* result in heterotaxy or isolated CHD (phenotypes including DORV, ASD and VSD [49, 50]. In addition, the *ZIC3* gene is located in X chromosome, so it may contribute to the pathogenesis of CHD in male and female differently. NR2F2 was identified to be a member of the steroid thyroid hormone superfamily of nuclear receptors, involving in the regulation of many different genes in development [55, 56]. In human cases and mouse models, NR2F2 has been crucially implicated in angiogenesis and heart development, and abnormal expression or depletion of NR2F2 leads to AVSD (atrioventricular septal defect) and VSD (ventricular septal defects) [51]. Consistently, we also found DMRs in BMP and Wnt signaling pathway-related genes, indicating that the regulation of these signaling pathways by ZIC3 or NR2F2 may be critical for normal heart development. Using additional normal and clinical DORV samples, we confirmed promoter hypermethylation of *ZIC3* and *NR2F2* in DORV patients and the anti-correlation between their methylation and gene expression. Taken together, aberrant methylation at promoter regions of *ZIC3* and *NR2F2* and their dysregulated gene transcription levels, may contribute to DORV in human heart development. However, the decisive conclusion needs further investigations, since epigenetic changes in blood may not be able to fully reflect the causative basis of the disease due to lack of more DORV cases in twins and difficulty in obtaining heart samples.

DMRs were also found in the upstream of *CITED1*, *GATA2*, *SOX3* and some important epigenetic genes, including *MTA2*, *NSD1*, *MECP2* and *SUV39H1*, indicating that they might also contribute to DORV. The MZ twins shared the similar but not exactly the same environment, especially the postnatal environment. Thus, we suggest that the non-shared environmental and stochastic factors, including physical changes, chemical pollutants, dietary components, temperature changes and other external stresses during pregnancy [6, 57, 58], may contribute to the pathogenesis of DORV through the mediation of epigenetic changes.

## Conclusions

In conclusion, disease-discordant MZ twin pairs are outstanding subjects to study epigenetic mechanisms driving a number of pathologies. Here, using DNA methylation profiling technology to analyze genome-wide DNA methylation, we described differentially methylated regions in a DORV-discordant MZ twin pair. A limitation to our study is that we only obtained one MZ twin pair discordant for DORV, and the present results call for more DORV discordant twins and extensive tests for the generalizability of our findings. Nevertheless, our results provide new insights into the mechanism of DORV, a rare disease that has been less studied by genomic and epigenomic approaches.

## Methods

### Patients and materials

Genomic DNA was extracted from the donated whole blood samples of DORV patients and normal people by using the DNeasy Blood & Tissue Kit (Qiagen, Cat no. 69504). This study was conducted in accordance with the principles of the Declaration of Helsinki and has been reviewed and approved by the Medical Ethics Committee of Fuwai Hospital. Written informed consent was obtained from the twins’ parents and mentioned samples’ providers.

### Reduced representation bisulfite sequencing (RRBS)

MspI-digested RRBS library was prepared as published [36], one hundred bp paired-end reads were generated from Illumina Hiseq2000 platform (BIOPIC, Peking University, Beijing). Raw reads were trimmed adapters and low quality bases using trim_galore in RRBS mode. Human genome (hg19) was indexed with bismark_genome_preparation (a script from bismark mapping package), and then, all clean reads aligned to indexed human genome using bismark (−-bowtie2). To extract the methylation information for individual cytosines, bismark_methylation_extractor (−p --cytosine_report --CX --no_overlap) in paired-end mode was applied, and the output CX_report file was sorted by chromosomes using linux shell commands (awk). The sorted CX_report files were then used for downstream analysis.

### DNA methylation detection and quantitative RT-PCR

To monitor CpG methylation of screened DMRs in promoters, genomic DNA was treated with sodium bisulfite using EpiTect Bisulfite Kit (Qiagen, USA). The converted DNA was then amplified by PCR with specific primers (*ZIC3*: Forward: 5′-GAGTGATTGATTTTATTAGTTTAAGGATAT-3’Reverse: 5’-AACCAAAAAACTCCCTAAATACC-3′; *NR2F2*: Forward: 5′-GAAGTAGGAAAGGGTGGG-3’ Reverse: 5’-CGAACCCAAACTATTATCTAAC-3′), PCR products were purified, ligated into pEasy-T5 vector (Transgene, China) and then transduced into competent *Escherichia.coli*. When bacteria colonies appeared on the plate, at least 20 independent clones were selected and sequenced. The sequenced results were analyzed by BiQ analyzer (Max Plank Institute, Germany). Total RNA was extracted with RNAliquid Kit (Aidlab, China) and mRNA expression levels were detected with one-step RT-PCR kit (Takara, China) on lightcycler (Roche, USA). RT-qPCR primers were listed as follows: *ZIC3*: Forward: 5′- GGCGCTCAGTTTCCTAACTAC-3’Reverse: 5′- CTGCCGCATATAACGGAAGAA-3′; *NR2F2*: Forward: 5′- AACCAGCCGACGAGATTCG-3’ Reverse: 5′- CCCGGATGAGGGTTTCGATG-3′.

### DMRs detection

Differentially methylated regions (DMRs) were detected based on a windows swiping method. We used a 100 bp window sliding on the genome at a 50 bp step to find differentially methylated windows (DMWs) between two samples, and the neighboring windows were joined together as a DMR. Only >10X CpG sites were used to calculate DMWs. To test the different average methylations in a window between two samples, Wilcoxon test was applied, and *p*-value < 0.01 was then considered as the DMW. A significant DMW was discarded if less than five CpG sites in the window and average methylation levels between two samples were less than 10%. Finally, adjacent DMWs were joined together as DMRs using BEDtools (intersectBed). DMRs were annotated by BEDtools (intersectBed) and ANNOVAR [59].

### Gene ontology (GO) and pathway enrichment

Annotated DMRs were separated into 3 subsets: gene upstreams (2 kb in front of TSS), gene bodies and transcript factor binding sites. Genes which had DMRs in upstreams and gene bodies were submitted to Database for Enrichr and Annotation, Visualization and Integrated Discovery (DAVID) respectively, GO enrichments used GOTERM_BP_FAT database under default parameters, and pathway enrichments used Kyoto Encyclopedia of Genes and Genomes (KEGG) database. DMRs which annotated as transcript factors binding sites by ANNOVAR (using tfbsConsSites database downloaded from UCSC [60]) were considered to influence the TFs function, so we analyzed those TFs using DAVID. GO (biological process) and pathway enrichments were obtained to understand those DMRs’ biological meanings.

## Additional files

Additional file 1: **Table S1.** Overview of WGS data. (XLSX 39 kb)

Additional file 2: **Table S2.** D3 specific SNVs and short InDels (XLSX 82 kb)

Additional file 3: **Table S3.** Sequence variations reported related to VSD. (XLSX 30 kb)

Additional file 4: **Figure S1.** Comparison of copy number profiles of 22 pairs of autochromosomes and X chromosome between twin pair. Normalized copy number profiles of D3 and D4. Each point shows a 5 kb windows (all chromosomes) of sequencing reads normalized by GC-content and map-ability using Control-FREEC. (PDF 1756 kb)

Additional file 5: **Table S4.** Analysis of CNVs two samples. (XLSX 46 kb)

Additional file 6: **Figure S2.** D3 specific structure variations (SVs) analysis. Alignments of reads in both D3 (top panel) and D4 (bottom panel) at each break points detected by CREST. The header lines in blue given the detail information of each structure variation (columns meanings: left_chr, left_pos, left_strand, number of left soft-clipped reads, right_chr, right_pos, right_strand, number right soft-clipped reads, SV type, coverage at left_pos, coverage at right_pos, assembled length at left_pos, assembled length at right_pos, average percent identity at left_pos, percent of non-unique mapping reads at left_pos, average percent identity at right_pos, percent of non-unique mapping reads at right_pos, start position of consensus mapping to genome, starting chromosome of consensus mapping, position of the genomic mapping of consensus starting position, end position of consensus mapping to genome, ending chromosome of consensus mapping, position of genomic mapping of consensus ending position, and consensus sequences). (PDF 626 kb)

Additional file 7: **Table S5.** Overview of RRBS data. (XLS 26 kb)

Additional file 8: **Figure S3.** Analysis of WGBS data of B cell and heart tissue from the ENCODE project. (A) Scatter plot and Pearson’s correlation analysis of DNA methylation of B cell and heart. Pearson’s correlation coefficient was listed above the plot. (B) Visualization of the DNA methylation status of B cell and heart in chr1:1-3 M by IGV. (JPG 500 kb)

Additional file 9: **Figure S4.** Comparison of systemic changes of methylome between two samples. (A) Cumulative depth distribution of RRBS data. The x-axis represents the depth of cytosine, and the y-axis represents the fraction of cytosine ≤ depth. (B) Methylation levels distribution in two samples of three different kinds of cytosine (CG, CHG, CHH). The x-axis is the methylation level; y-axis shows the log2 counts of the cytosine under a methylated level. (C) CHH methylation profile in the gene body, upstream and downstream. (D) CHG methylation profile in the gene body, upstream and downstream. (PDF 891 kb)

Additional file 10: **Table S6.** DMRs between two samples. (XLSX 580 kb)

Additional file 11: **Table S7.** GO and KEGG analysis of DMRs located in upstream region. (XLSX 43 kb)

Additional file 12: **Table S8.** GO and KEGG analysis of DMRs located in gene body. (XLSX 99 kb)

Additional file 13: **Table S9.** GO and KEGG analysis of DMRs located in TF binding sites. (XLSX 80 kb)

Additional file 14: **Figure S5.** Aberrant methylation in the upstream regions of *CITED1.* Visualizing the methylation levels of DMRs near *CITED1* with UCSC genome browser. Methylated levels in the twins are showed in blue (D3) and red (D4). Transcription factor binding sites are also showed in zooming-in panels, which indicated by black bars with names marked in front. Arrows give TSSs and transcriptional orientation. Transcription factor binding sites, Pol II ChIP-seq and TBP ChIP-seq data from ENCODE. (PDF 910 kb)

Additional file 15: **Figure S6.** Aberrant methylation in the upstream regions of *GATA2.* Visualizing the methylation levels of DMRs near *GATA2* with UCSC genome browser. Methylated levels in the twins are showed in blue (D3) and red (D4). An arrow gives TSS and transcriptional orientation. Transcription factor binding sites, Pol II ChIP-seq and TBP ChIP-seq data from ENCODE. (PDF 767 kb)

Additional file 16: **Figure S7.** Aberrant methylation in the upstream regions of *SOX3.* Visualizing the methylation levels of DMRs near *SOX3* with UCSC genome browser. Methylated levels in the twins are showed in blue (D3) and red (D4). Transcription factor binding sites are also showed in zooming-in panels, which indicated by black bars with names marked in front. An arrow gives TSS and transcriptional orientation. Transcription factor binding sites, Pol II ChIP-seq and TBP ChIP-seq data from ENCODE. (PDF 590 kb)

Additional file 17: **Figure S8.** Aberrant methylation in the upstream regions of *NSD1.* Visualizing the methylation levels of DMRs near *NSD1* with UCSC genome browser. Methylated levels in the twins are showed in blue (D3) and red (D4). Transcription factor binding sites are also showed in zooming-in panels, which indicated by black bars with names marked in front. An arrow gives TSS and transcriptional orientation. Transcription factor binding sites, Pol II ChIP-seq and TBP ChIP-seq data from ENCODE. (PDF 781 kb)

Additional file 18: **Figure S9.** Aberrant methylation in the upstream regions of *MTA2.* Visualizing the methylation levels of DMRs near *MTA2* with UCSC genome browser. Methylated levels in the twins are showed in blue (D3) and red (D4). Transcription factor binding sites are also showed in zooming-in panels, which indicated by black bars with names marked in front. An arrow gives TSS and transcriptional orientation. Transcription factor binding sites, Pol II ChIP-seq and TBP ChIP-seq data from ENCODE. (PDF 633 kb)

Additional file 19: **Figure S10.** Aberrant methylation in the upstream regions of *MECP2.* Visualizing the methylation levels of DMRs near *MECP2* with UCSC genome browser. Methylated levels in the twins are showed in blue (D3) and red (D4). Transcription factor binding sites are also showed in zooming-in panels, which indicated by black bars with names marked in front. Arrows give TSSs and transcriptional orientation. Transcription factor binding sites, Pol II ChIP-seq and TBP ChIP-seq data from ENCODE. (PDF 969 kb)

Additional file 20: **Figure S11.** Aberrant methylation in the upstream regions of *SUV39H1.* Visualizing the methylation levels of DMRs near *SUV39H1* with UCSC genome browser. Methylated levels in the twins are showed in blue (D3) and red (D4). Transcription factor binding sites are also showed in zooming-in panels, which indicated by black bars with names marked in front. An arrow gives TSS and transcriptional orientation. Transcription factor binding sites, Pol II ChIP-seq and TBP ChIP-seq data from ENCODE. (PDF 748 kb)

Additional file 21: **Table S10.** Expression profiling data of embryo and adult heart. (XLSX 9 kb)

Additional file 22: **Figure S12.** DNA methylation detection of *ZIC3* from clinical samples. Bisulfite sequencing tested DNA methylation status of DMRs in *ZIC3* in 20 clinical samples, five normal providers (1–5) and fifteen DORV patients (6–20). Methylated and unmethylated CpG sites are indicated as respective black and white circles. (PDF 5226 kb)

Additional file 23: **Figure S13.** DNA methylation detection of *NR2F2* from clinical samples. Bisulfite sequencing detected DNA methylation status of DMRs in *NR2F2* in 20 clinical samples, five normal providers (1–5) and fifteen DORV patients (6–20). Methylated and unmethylated CpG sites are indicated as black and white circles, respectively. (PDF 5463 kb)

## Abbreviations

ASD: Atrial septal defect
CHD: Congenital heart disease
*CITED1*: Cbp/p300 interacting transactivator with Glu/Asp rich carboxy-terminal domain 1
CNVs: Copy number variations
DAVID: Database for Annotation, Visualization and Integrated Discovery
DMRs: Differentially methylated regions
DMWs: Differentially Methylated Windows
DORV: Double outlet right ventricle
*GATA2*: GATA-binding protein 2
*GATA4*: GATA-binding factor 4
HGMD: Human Genetic Mutation Database
HNF1: Hepatocyte nuclear factor 1
InDels: Insertions/deletions
IRF2: Interferon regulatory factor 2
KEGG: Kyoto Encyclopedia of Genes and Genomes
MAPK: Mitogen activated protein kinase
*MECP2*: Methyl CpG binding protein 2
*MTA2*: Metastasis associated 1 family member 2
MZ: Monozygotic
*NR2F2*: Nuclear receptor subfamily 2 group F member 2
*NSD1*: Nuclear receptor binding SET domain protein 1
OMIM: Online Mendelian Inheritance in Man
P300: Histone acetyltransferase p300
Pol II: RNA polymerase II
RRBS: Reduced representation bisulfite sequencing
SNVs: Single nucleotide variations
*SOX3*: SRY-box 3
*SUV39H1*: Suppressor of variegation 3–9 homolog 1
T1D: Type 1 diabetes
TBP: TATA-box binding protein
*TBX1*: T-box transcription factor 1
TFs: Transcription factors
TGF: Transforming growth factor
TSS: Transcription start sites
VEGF: Vascular endothelial growth factor
VSD: Ventricular septal defect
*ZIC3*: Zic family member 3
