# Supplementary figures and images for: Genome and epigenome analysis of monozygotic twins discordant for congenital heart disease
Source: BMC Genomics. 2018 Jun 4;19:428. doi: 10.1186/s12864-018-4814-7 (PMC5987557; doi:10.1186/s12864-018-4814-7)

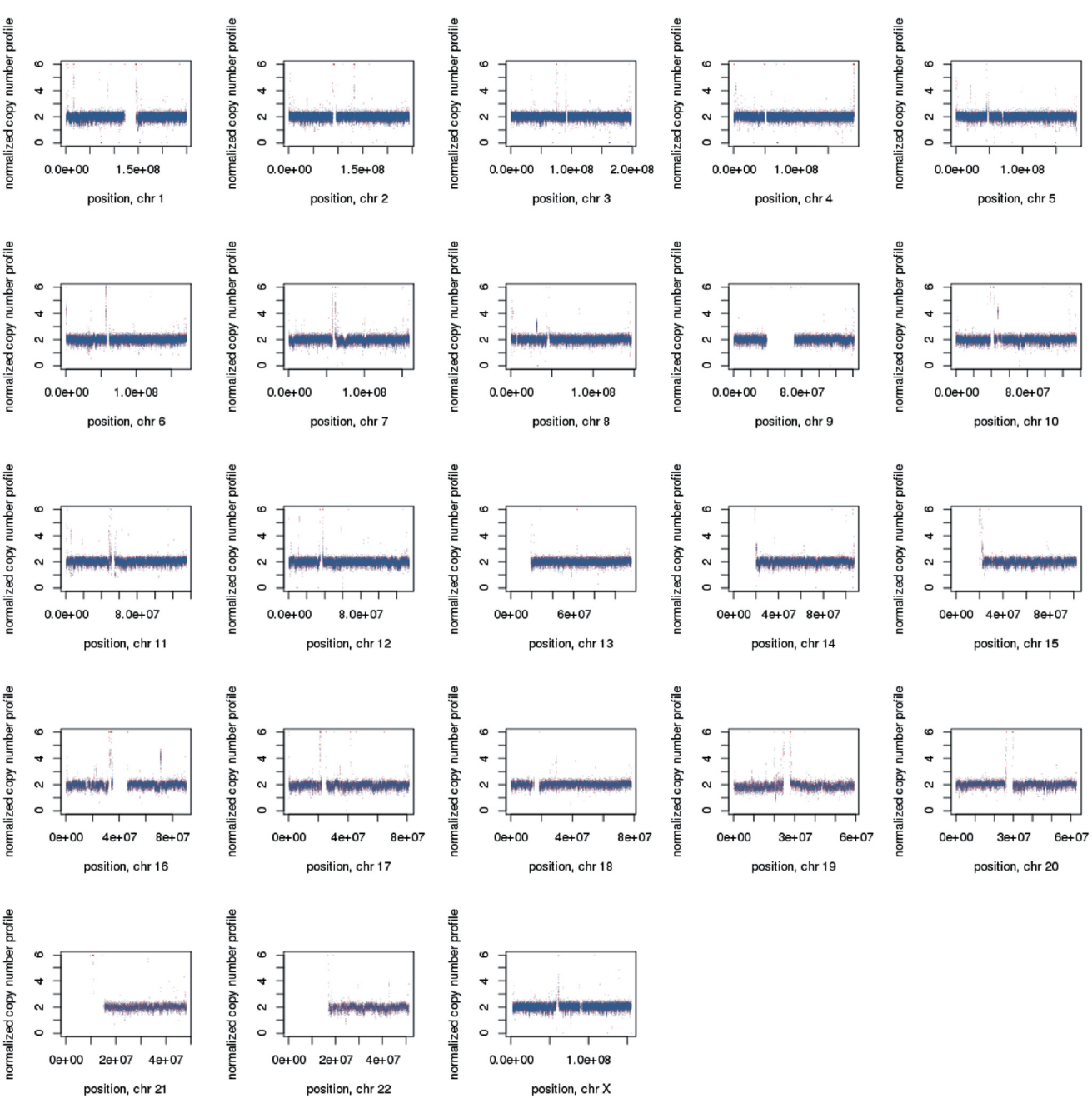

Supplement: Supplementary file 4 — Figure S1. Comparison of copy number profiles of 22 pairs of autochromosomes and X chromosome between twin pair. Normalized copy number profiles of D3 and D4. Each point shows a 5 kb windows (all chromosomes) of sequencing reads normalized by GC-content and map-ability using Control-FREEC. (PDF 1756 kb) [file 12864_2018_4814_MOESM4_ESM.pdf]

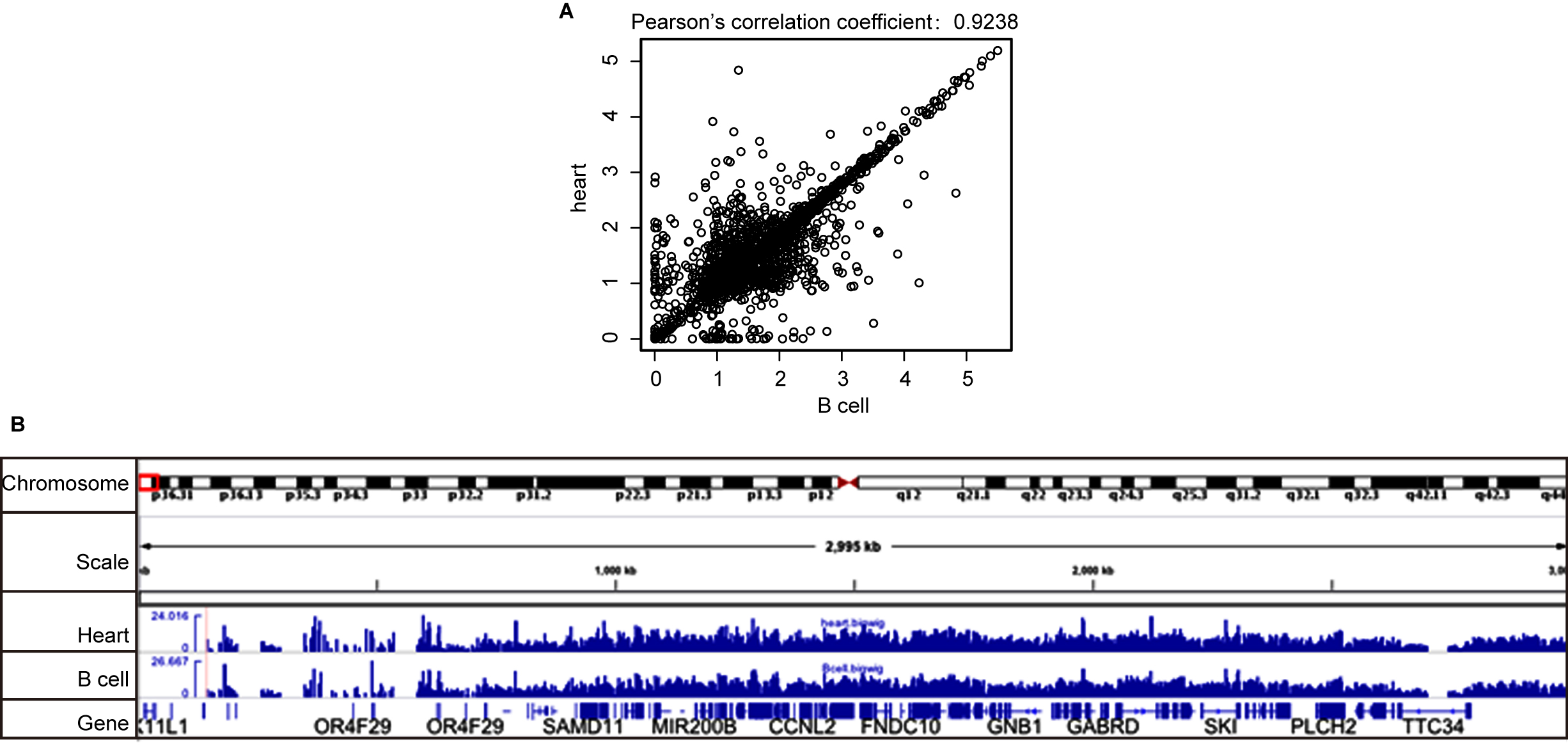

Supplement: Supplementary file 8 — Figure S3. Analysis of WGBS data of B cell and heart tissue from the ENCODE project. (A) Scatter plot and Pearson’s correlation analysis of DNA methylation of B cell and heart. Pearson’s correlation coefficient was listed above the plot. (B) Visualization of the DNA methylation status of B cell and heart in chr1:1-3 M by IGV. (JPG 500 kb) [file 12864_2018_4814_MOESM8_ESM.jpg]

**A**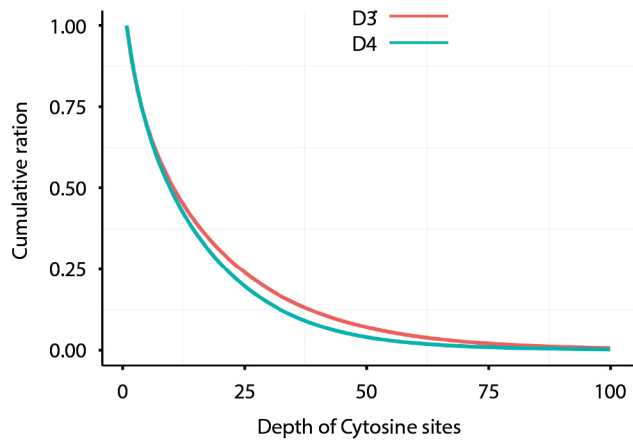**B**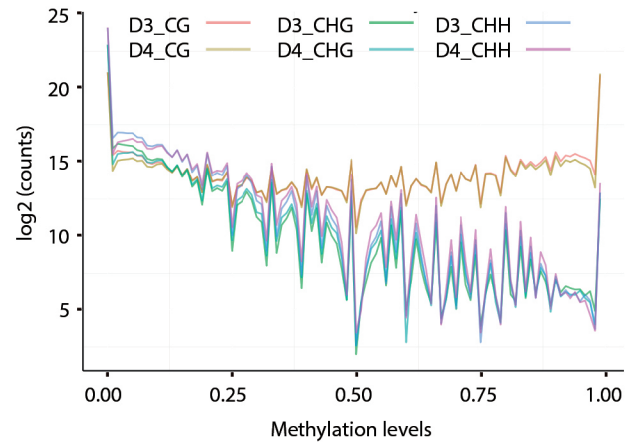**C**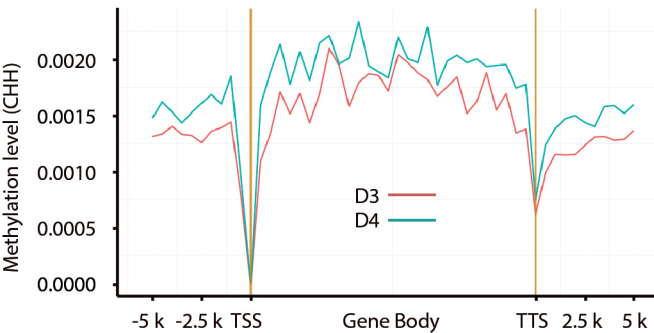**D**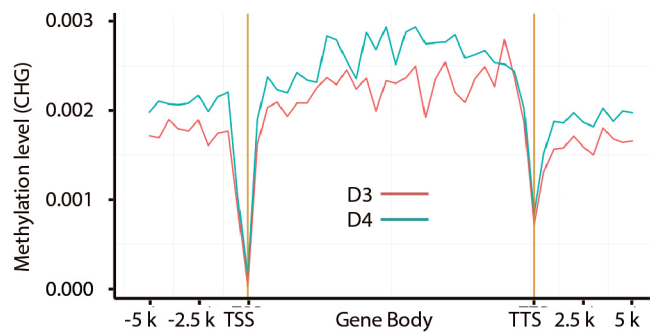

Supplement: Supplementary file 9 — Figure S4. Comparison of systemic changes of methylome between two samples. (A) Cumulative depth distribution of RRBS data. The x-axis represents the depth of cytosine, and the y-axis represents the fraction of cytosine ≤ depth. (B) Methylation levels distribution in two samples of three different kinds of cytosine (CG, CHG, CHH). The x-axis is the methylation level; y-axis shows the log2 counts of the cytosine under a methylated level. (C) CHH methylation profile in the gene body, upstream and downstream. (D) CHG methylation profile in the gene body, upstream and downstream. (PDF 891 kb) [file 12864_2018_4814_MOESM9_ESM.pdf]

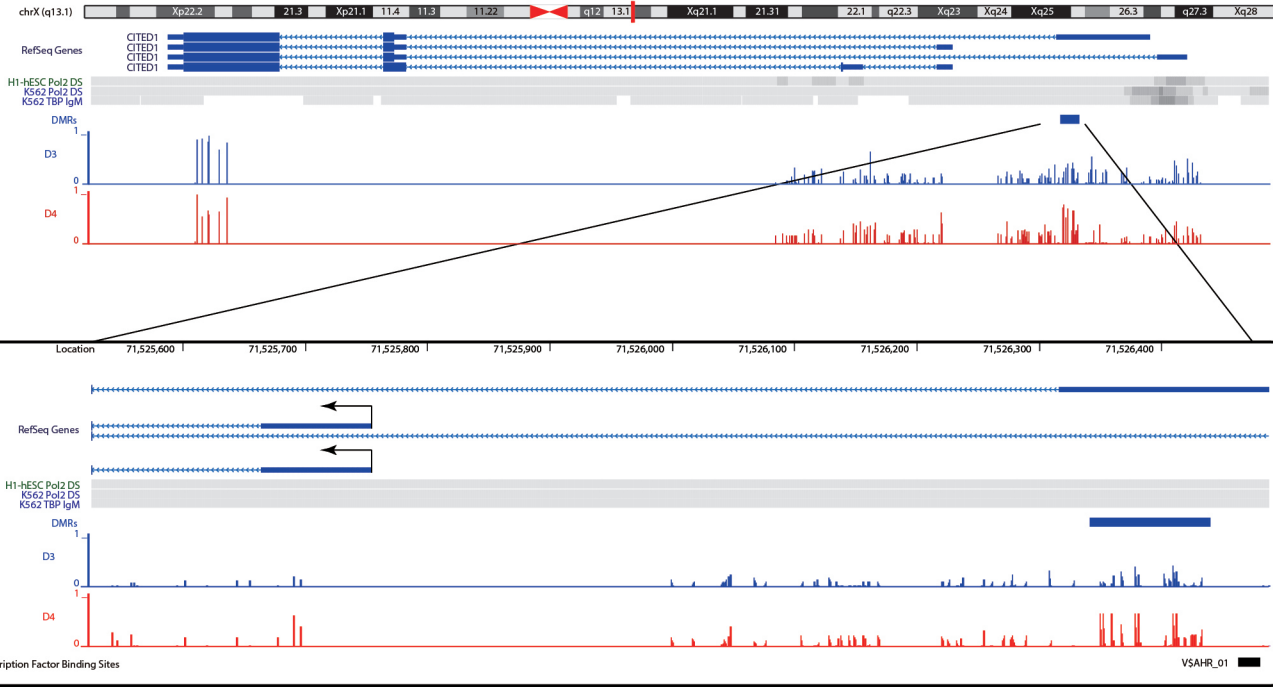

Supplement: Supplementary file 14 — Figure S5. Aberrant methylation in the upstream regions of CITED1. Visualizing the methylation levels of DMRs near CITED1 with UCSC genome browser. Methylated levels in the twins are showed in blue (D3) and red (D4). Transcription factor binding sites are also showed in zooming-in panels, which indicated by black bars with names marked in front. Arrows give TSSs and transcriptional orientation. Transcription factor binding sites, Pol II ChIP-seq and TBP ChIP-seq data from ENCODE. (PDF 910 kb) [file 12864_2018_4814_MOESM14_ESM.pdf]

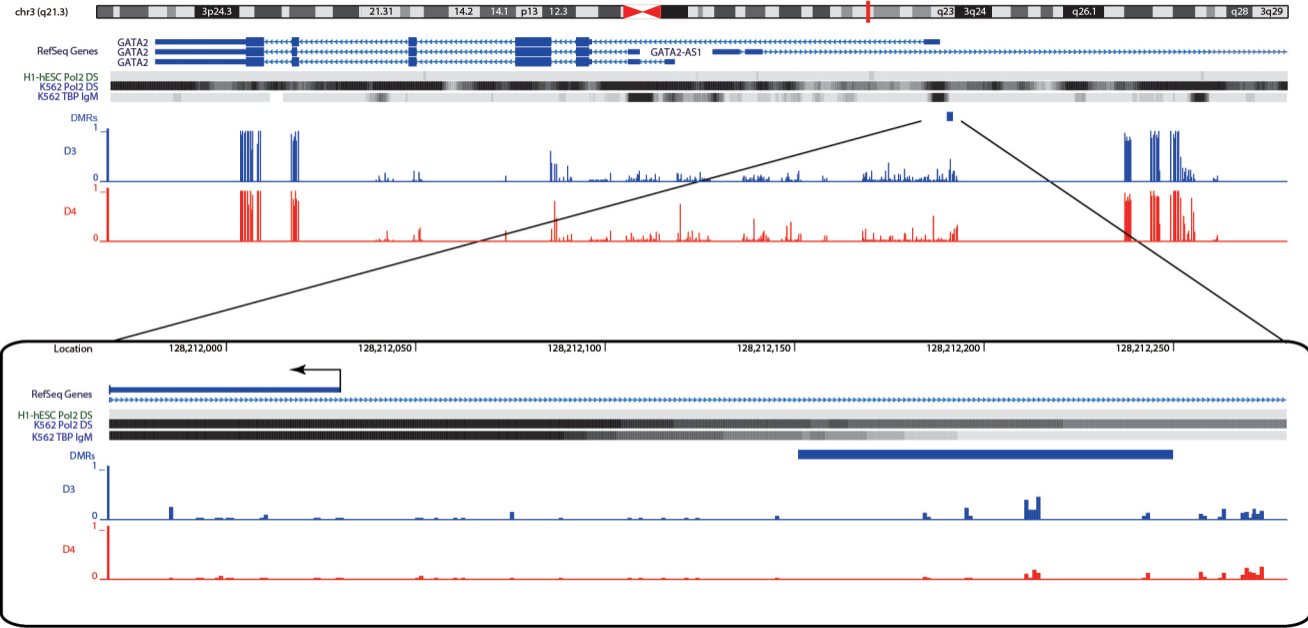

Supplement: Supplementary file 15 — Figure S6. Aberrant methylation in the upstream regions of GATA2. Visualizing the methylation levels of DMRs near GATA2 with UCSC genome browser. Methylated levels in the twins are showed in blue (D3) and red (D4). An arrow gives TSS and transcriptional orientation. Transcription factor binding sites, Pol II ChIP-seq and TBP ChIP-seq data from ENCODE. (PDF 767 kb) [file 12864_2018_4814_MOESM15_ESM.pdf]

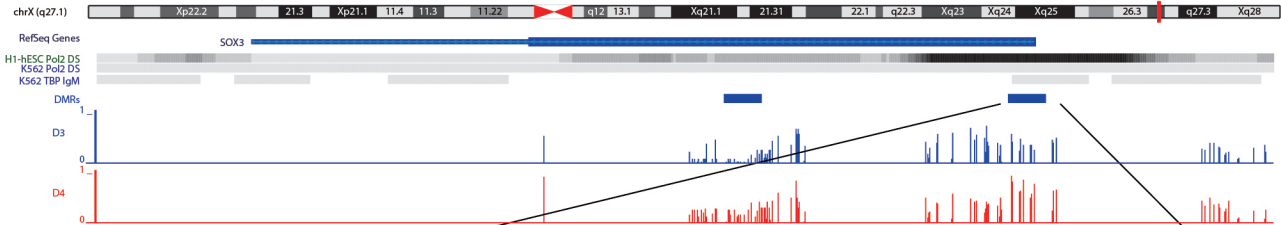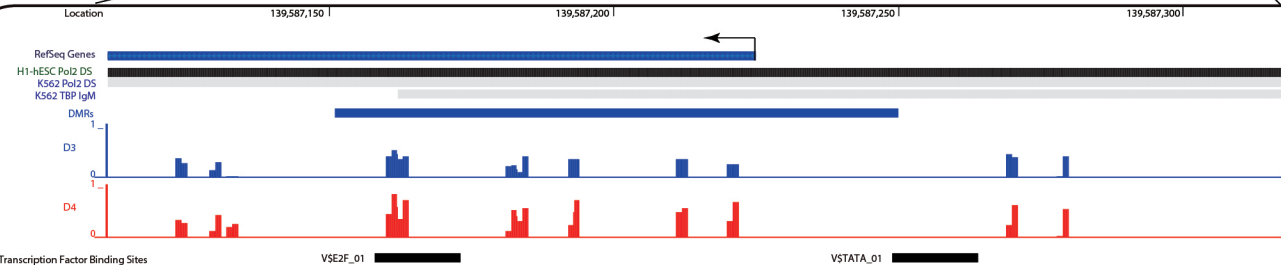

Supplement: Supplementary file 16 — Figure S7. Aberrant methylation in the upstream regions of SOX3. Visualizing the methylation levels of DMRs near SOX3 with UCSC genome browser. Methylated levels in the twins are showed in blue (D3) and red (D4). Transcription factor binding sites are also showed in zooming-in panels, which indicated by black bars with names marked in front. An arrow gives TSS and transcriptional orientation. Transcription factor binding sites, Pol II ChIP-seq and TBP ChIP-seq data from ENCODE. (PDF 590 kb) [file 12864_2018_4814_MOESM16_ESM.pdf]

chr11 (q12.3) 11p15.4 15.2 p15.1 p14.3 14.1 11p13 11p12 p11.2 12.1 q13.4 11q14.1 q14.3 11q21 q22.1 11q22.3 11q23.3 24.2 11q25

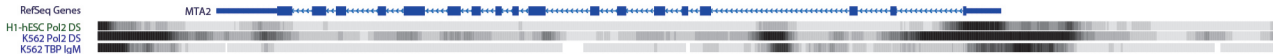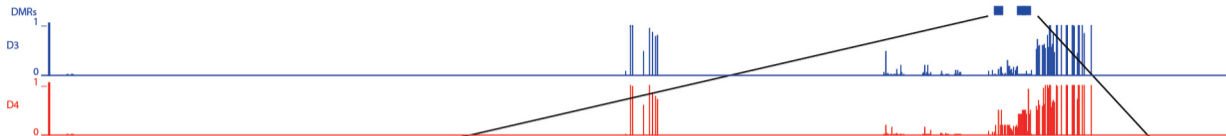

Location 623,693,00 623,694,00 623,695,00 623,696,00 623,697,00 623,698,00 623,699,00 623,700,00 623,701,00

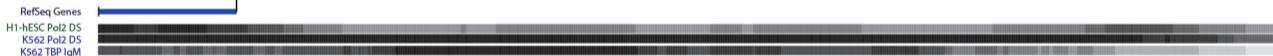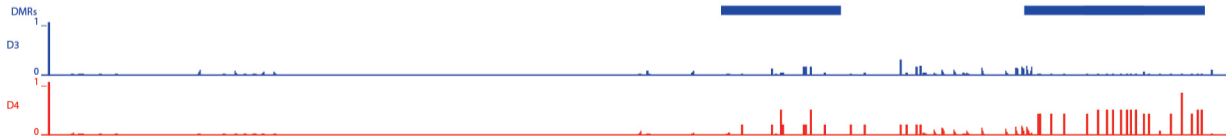

Transcription Factor Binding Sites

V\$OCT\_C

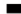

V\$USF\_Q6

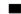

V\$PAX2\_01  
V\$PAX6\_01

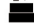

Supplement: Supplementary file 18 — Figure S9. Aberrant methylation in the upstream regions of MTA2. Visualizing the methylation levels of DMRs near MTA2 with UCSC genome browser. Methylated levels in the twins are showed in blue (D3) and red (D4). Transcription factor binding sites are also showed in zooming-in panels, which indicated by black bars with names marked in front. An arrow gives TSS and transcriptional orientation. Transcription factor binding sites, Pol II ChIP-seq and TBP ChIP-seq data from ENCODE. (PDF 633 kb) [file 12864_2018_4814_MOESM18_ESM.pdf]

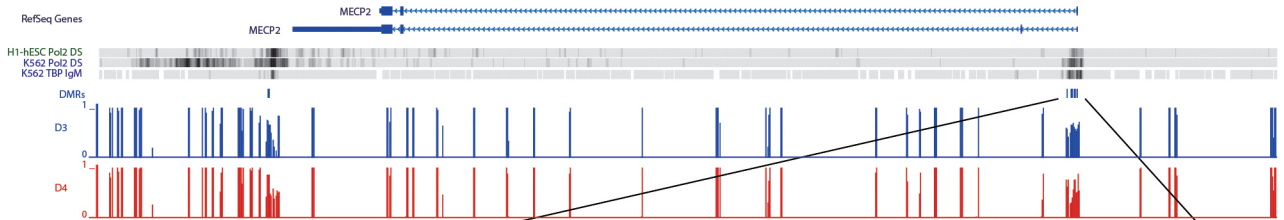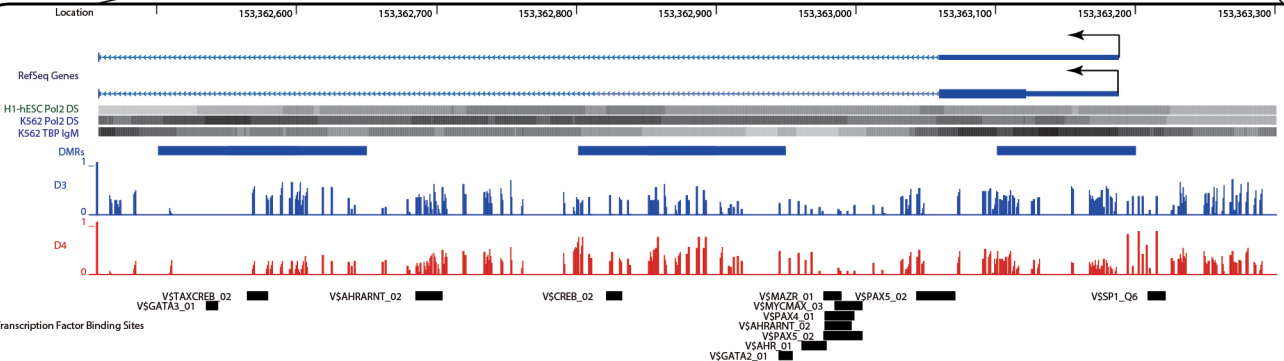

Supplement: Supplementary file 19 — Figure S10. Aberrant methylation in the upstream regions of MECP2. Visualizing the methylation levels of DMRs near MECP2 with UCSC genome browser. Methylated levels in the twins are showed in blue (D3) and red (D4). Transcription factor binding sites are also showed in zooming-in panels, which indicated by black bars with names marked in front. Arrows give TSSs and transcriptional orientation. Transcription factor binding sites, Pol II ChIP-seq and TBP ChIP-seq data from ENCODE. (PDF 969 kb) [file 12864_2018_4814_MOESM19_ESM.pdf]

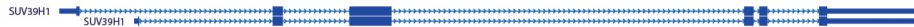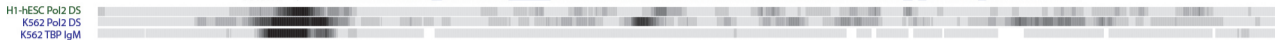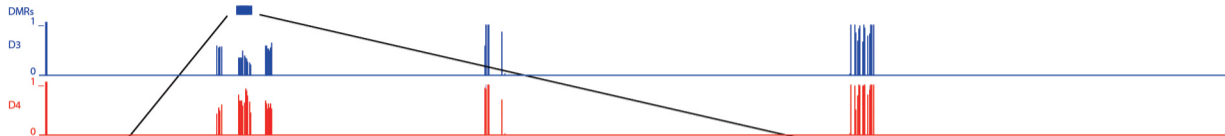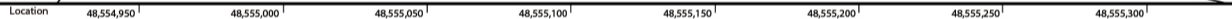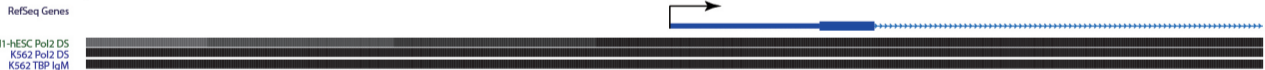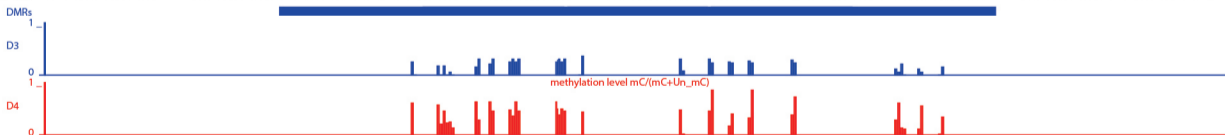

Transcription Factor Binding Sites

VSEGR3\_01

V\$AHRRARNT\_02

VSEGR2\_01

VSP53\_01

VSE2F\_01

Supplement: Supplementary file 20 — Figure S11. Aberrant methylation in the upstream regions of SUV39H1. Visualizing the methylation levels of DMRs near SUV39H1 with UCSC genome browser. Methylated levels in the twins are showed in blue (D3) and red (D4). Transcription factor binding sites are also showed in zooming-in panels, which indicated by black bars with names marked in front. An arrow gives TSS and transcriptional orientation. Transcription factor binding sites, Pol II ChIP-seq and TBP ChIP-seq data from ENCODE. (PDF 748 kb) [file 12864_2018_4814_MOESM20_ESM.pdf]

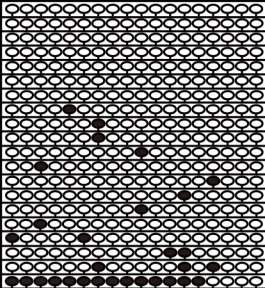

1

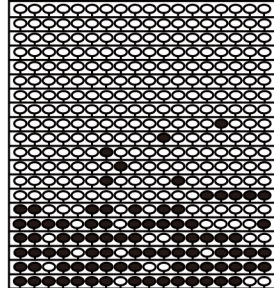

2

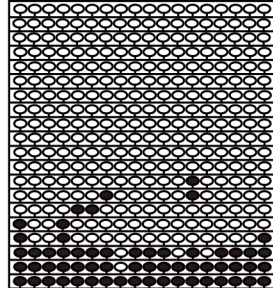

3

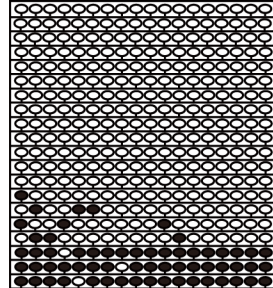

4

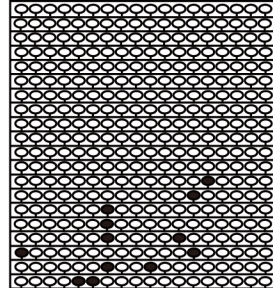

5

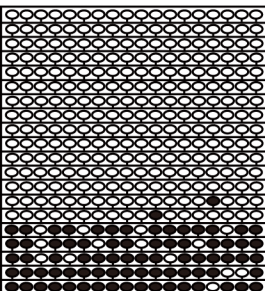

6

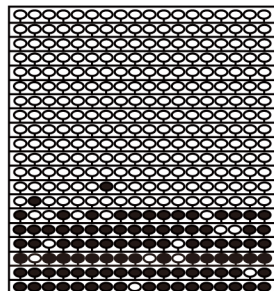

7

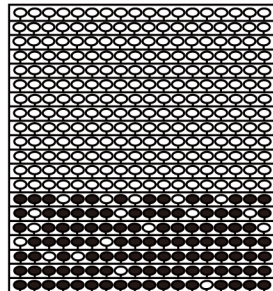

8

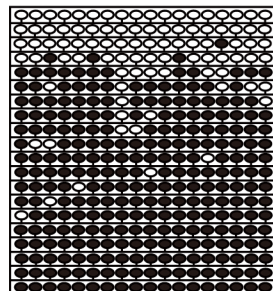

9

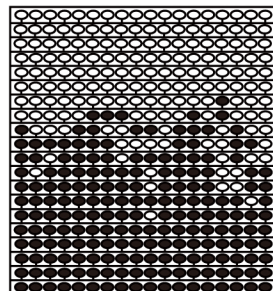

10

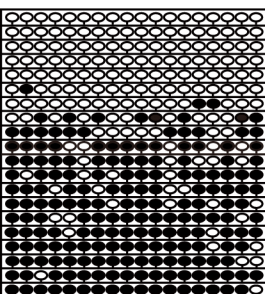

11

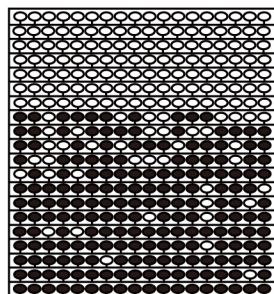

12

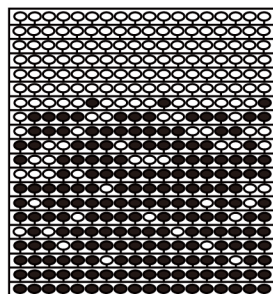

13

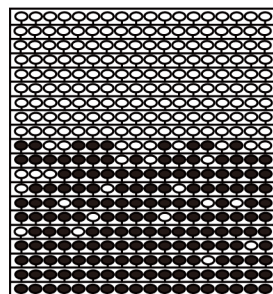

14

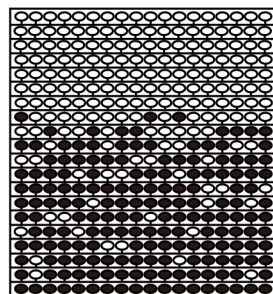

15

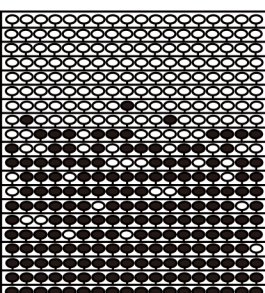

16

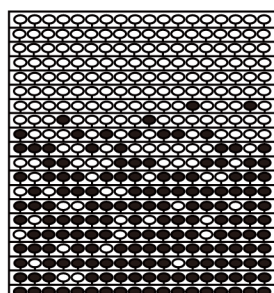

17

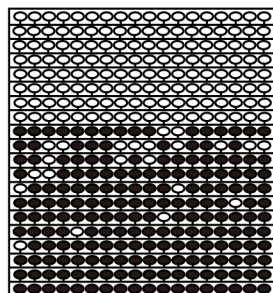

18

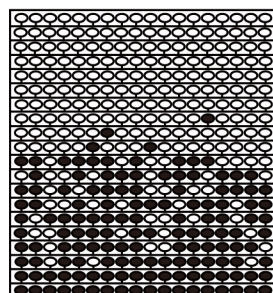

19

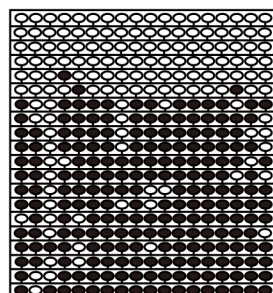

20

Supplement: Supplementary file 22 — Figure S12. DNA methylation detection of ZIC3 from clinical samples. Bisulfite sequencing tested DNA methylation status of DMRs in ZIC3 in 20 clinical samples, five normal providers (1–5) and fifteen DORV patients (6–20). Methylated and unmethylated CpG sites are indicated as respective black and white circles. (PDF 5226 kb) [file 12864_2018_4814_MOESM22_ESM.pdf]

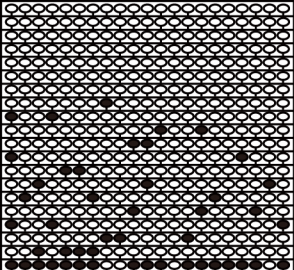

1

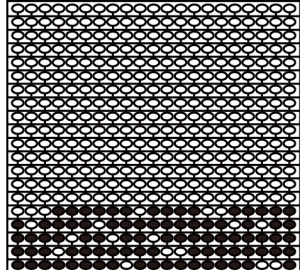

2

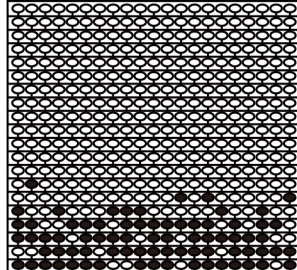

3

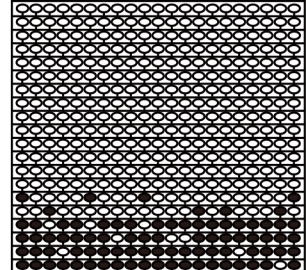

4

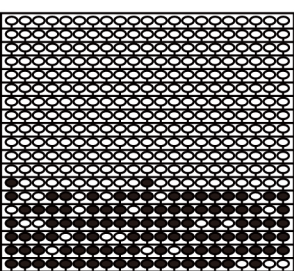

5

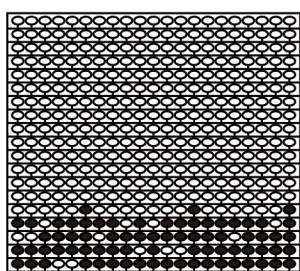

6

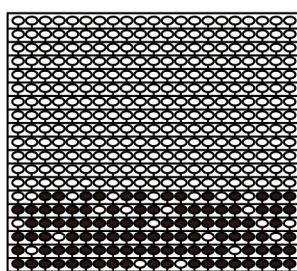

7

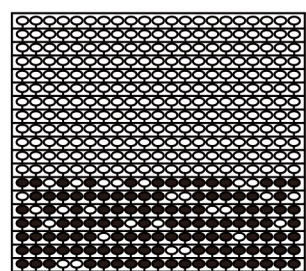

8

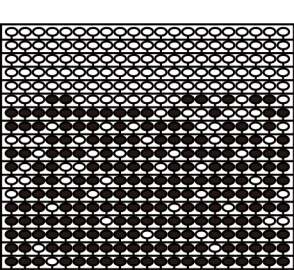

9

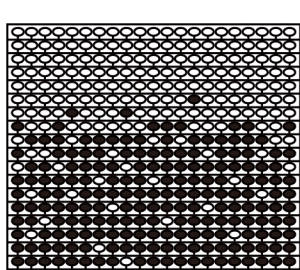

10

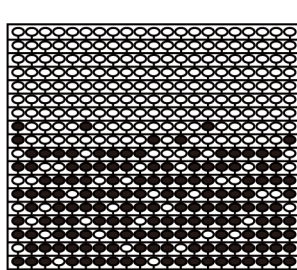

11

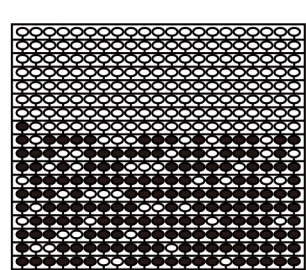

12

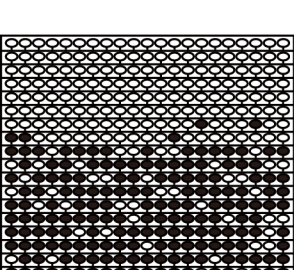

13

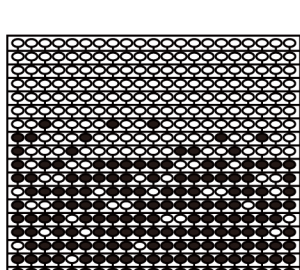

14

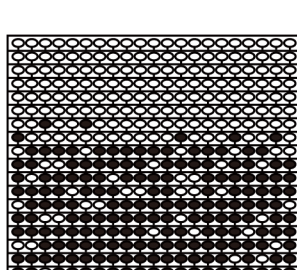

15

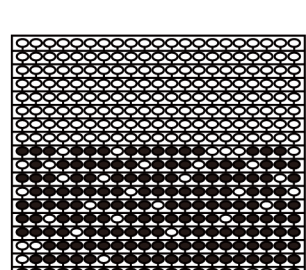

16

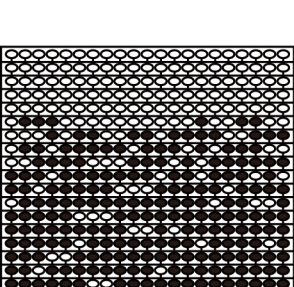

17

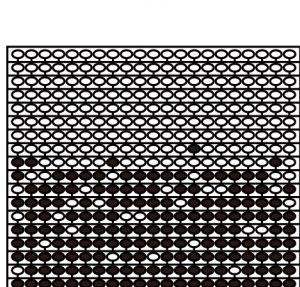

18

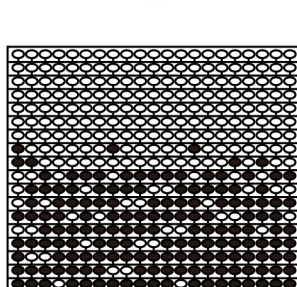

19

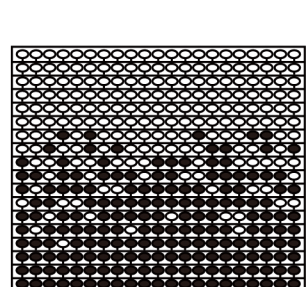

20

Supplement: Supplementary file 23 — Figure S13. DNA methylation detection of NR2F2 from clinical samples. Bisulfite sequencing detected DNA methylation status of DMRs in NR2F2 in 20 clinical samples, five normal providers (1–5) and fifteen DORV patients (6–20). Methylated and unmethylated CpG sites are indicated as black and white circles, respectively. (PDF 5463 kb) [file 12864_2018_4814_MOESM23_ESM.pdf]
