# Supplementary material for: Genome and epigenome analysis of monozygotic twins discordant for congenital heart disease
Source: BMC Genomics. 2018 Jun 4;19:428. doi: 10.1186/s12864-018-4814-7 (PMC5987557; doi:10.1186/s12864-018-4814-7)
Supplement: Supplementary file 6 — Figure S2. D3 specific structure variations (SVs) analysis. Alignments of reads in both D3 (top panel) and D4 (bottom panel) at each break points detected by CREST. The header lines in blue given the detail information of each structure variation (columns meanings: left_chr, left_pos, left_strand, number of left soft-clipped reads, right_chr, right_pos, right_strand, number right soft-clipped reads, SV type, coverage at left_pos, coverage at right_pos, assembled length at left_pos, assembled length at right_pos, average percent identity at left_pos, percent of non-unique mapping reads at left_pos, average percent identity at right_pos, percent of non-unique mapping reads at right_pos, start position of consensus mapping to genome, starting chromosome of consensus mapping, position of the genomic mapping of consensus starting position, end position of consensus mapping to genome, ending chromosome of consensus mapping, position of genomic mapping of consensus ending position, and consensus sequences). (PDF 626 kb) [file 12864_2018_4814_MOESM6_ESM.pdf]

chr7 61873537 - 3 chr7 61876123 + 3 ITX 63 63 17 56 0.953333333333333 0 0.992098765432099 0 1 chr7 61876216 150 chr7 61873601

AAACTAGAGGGAAGCTCTCTGAGAAACCACTTTGTGTGACATGCATATATCTCTCTCTCAGGCCAATGGTAAAAGGAAATATCCTAGAATAAAACCTGGAAGTAAGCTCTCTGAGAACCTGCTTCATGTTGTGTGCATTCATCTCACAAA

```
                                   61873400            61873420            61873440            61873460            61873480            61873500            61873520            61873540            61873560            61873580            61873600            61873620            61873640            61873660            61873680
chr7:61873391-61873686             tgataagctactttctgatgtgtgcattcatctcacagagttaaaactttctattgattcagcagtttggaagcactgttttcgaccattctgcaagtggacgattgggagcttacttagaccaatggtgaaatagtgaatattacagaataaaacctggaagtaagctctctgagaacctgcttcatgttgtgtgcattcatctcacaaagttaaatatttcttttcattcagcagataggaaacactgtttttgtagtacctgtgaagggatatttgggatcacagtgaggcct

ST-E00144:68:H2LCKCCXX:4:2114:12703:70013+ TaAGcTATaTTCTGATGTGTGCATTCATCTCACAGAGTTAAAACTTTCTATTGATTCAGCAGTTTGGAAGCACTtTTTTCGACCATTCTGCAAGTGGACGATTGGgAGCTTACTtAGACCAAtGGTGAAATAGTGAATATtACAGAATAA
ST-E00144:68:H2LCKCCXX:4:1101:20072:52256+ AGCTACTTTCTGATGTGTGCATTCATCTCACAGAGTTAAAACTTTCTATTGATACAGCAGTTTGGAAGCACTGTTTTCGACCATTCTGCAAGTGGACGATTGGGAGCTTACTTAGACCAATGGTGAAATAGTGAATATTACAGAATAAAA
ST-E00144:68:H2LCKCCXX:4:1124:16367:61451+ AGCTACTTTCTGATGTGTGCATTCATCTCACAGAGTTAAAACTTTCTATTGATACAGCAGTTTGGAAGCACTGTTTTCGACCATTCTGCAAGTGGACGATTGGGAGCTTACTTAGACCAATGGTGAAATAGTGAATATTACAGAATAAAA
ST-E00144:68:H2LCKCCXX:6:1102:31856:5510 + GCTACTTTCTGATGTGTGCATTCATCTCACAGAGTTAAAACTTTCTATTGATTCAGCAGTTTGGAAGCACTGTTTTCGACCATTCTGCAAGTGGACGATTGGGAGCTTACTTAGACCAATGGTGAAATAGTGAATATTACAGAATAAAAC
ST-E00144:68:H2LCKCCXX:6:1102:31947:5528 + GCTACTTTCTGATGTGTGCATTCATCTCACAGAGTTAAAACTTTCTATTGATTCAGCAGTTTGGAAGCACTGTTTTCGACCATTCTGCAAGTGGACGATTGGGAGCTTACTTAGACCAATGGTGAAATAGTGAATATTACaGAATAAAAC
ST-E00144:68:H2LCKCCXX:3:2217:26131:39018+ CTACTTTCTGATGTGTGCATTCATCTCACAGAGTTAAAACTTTCTATTGATACAGCAGTTTGGAAGCACTGTTTTCGACCATTCTGCAAGTGGACGATTGGGAGCTTACTTAGACCAATGGTGAAATAGTGAATATTACAGAATAAAACC
ST-E00144:68:H2LCKCCXX:7:1103:30211:53188+ TCTGATGTGTGCATTCATCTCACAGAGTTAAAACTTTCTATTGATTCAGCAGTTTGGAAGCACTGTTTTCGACCATTCTGCAAGTGGACGATTGGGAGCTTACTTAGACCAATGGTGAAATAGTGAATATTACAGAATAAAAcCTGGAAG
ST-E00144:68:H2LCKCCXX:7:2111:3324:8200  + GATGTGTGCATTCATCTCACAGAGTTAAAACTTTCTATTGATTCAGCAGTTTGGAAGCACTGTTTTCGACaATtcTGcAAGTGGACgATTGggAGCTTACtTAGACCAATGGtGAAATAGTGAATAtTACAGAAtAAAAactggAAGTAA
ST-E00144:68:H2LCKCCXX:1:2111:2431:53944 + CACAGAGTTAAAACTTTCTATTGATACAGCAGTTTGGAAGCACTGTTTTCGACCATTCTGCAAGTGAACGATTGGGAGCTTACTTAGACCAATGGTGAAATAGTGAATATTACAGAATAAAACCTGGAAGTAAGCTTtCaGAGAACCTGC
ST-E00144:68:H2LCKCCXX:6:1202:2938:26976 + CACAGAGTTAAAACTTTCTATTGATTCAGCAGTTTGGAAGCACTGTTTTCGACCATTCTGCAAGTGGACGaTTGGGAGCTTACTTAGACCAATGGTGAAATAGTGAATATTACAGAATAAAAccTgGaAGTAAGCTCTcTGAGAAC
ST-E00144:68:H2LCKCCXX:6:1220:25004:18151+ CACAGAGTTAAAACTTTCTATTGATTCAGCAGTTTGGAAGCACTGTTTTCGACCATTCTGCAAGTGGACGATTGGGAGCTTACTTAGACCAATGGTGAAATAGTGAATATTACAGAATAAAACCTGGAAGTAAGCTCTCTGAGAACCTGC
ST-E00144:68:H2LCKCCXX:4:1107:3202:20436 + AAACTTTCTATTGATTCAGCAGTTTGGAAGCACTGTTTTCGACCATTCTGCAAGTGGACGATTGGGAGCTTACTTAGACCAATGGTGAAATAGTGAATATTACAGAATAAAACCTGGAAGTAAGCTCTCTGAGAACCTGCTTCaTGTTG
ST-E00144:68:H2LCKCCXX:1:1107:10987:58216+ TTTCTATTGATTCAGCAGTTTGGAAGCACTGTTTTCGACCATTCTGCAAGTGGACGATTGGGAGCTTACTTAGACCAATGGTGAAATAGTGAATATTACAGAATAAAACCTGGAAGTAAGCTCTCTGAGAACCTGCTTCATGTTGTGTGC
ST-E00144:68:H2LCKCCXX:1:1213:16884:63649+ CTATTGATTCAGCAGTTTGGAAGCACTGTTTTCGACCATTCTGCAAGTGGACGATTGGGAGCTTACTTAGACCAATGGTGAAATAGTGAATATTACAGAATAAAACCTGGAAGTAAGCTCTCTGAGAACCTGCTTCATGTTGTGTGCATT
ST-E00144:68:H2LCKCCXX:1:1202:5313:52081 + GATTCAGCAGTTTGGAAGCACTGTTTTCGACCATTCTGCAAGTGGACGATTGGGAGCTTACTTAGACCAATGGTGAAATAGTGAATATTACAGAATAAAACCTGGAAGTAAGCTCTCTGAGAACCTGCTTCATGTTGTGTGCATTcatcT
ST-E00144:68:H2LCKCCXX:7:1220:5151:22475 + GATACAGCAGTTTGGAAGCACTGTTTTCGACCATTCTGCAAGTGGACGATTGGGAGCTTACTTAGACCAATGGTGAAATAGTGAATATTACAGAATAAAACCTGGAAGTAAGCTCTCTGAGAACCTGCTTCATGTTGTGTGCATTCATCT
ST-E00144:68:H2LCKCCXX:1:1223:14611:49830+ CAGCAGTTTGGAAGCACTGTTTTCGACCATTCTGCAAGTGGACGATTGGGAGCTTACTTAGACCAATGGTGAAATAGTGAATATTACAGAATAAAACCTGGAAGTAAGCTCTCTGAGAACCTGCTTCATGTTGTGTGCATTCATCTCACA
ST-E00144:68:H2LCKCCXX:4:2111:4918:62154 + CAGTTTGGAAGCACTGTTTTCGACCATTCTGCAAGTGGACGATTGGGAGCTTACTTAGACCAATGGTGAAATAGTGAATATTACAGAATAAAACCTGGAAGTAAGCTCtCTGAGAACCTGCTTCATGgTGTGtGCATTCATcTcaCAAAG
ST-E00144:68:H2LCKCCXX:8:1115:28851:57829+ AGCACTGTTTTCGACCATTCTGCAAGTGGACGATTGGGAGCTTACTTAGACCAATGGTGAAATAGTGAATATTACAGAATAAAACCTGGAAGTAAGCTCTCTGAGAACCTGCTTCATGTTGTGTGCATTCATCTCACAAAGTTAAATATT
ST-E00144:68:H2LCKCCXX:7:2111:25289:66637+ ACTGTTTTCGACCATTCTGCAAGTGGACGATTGGGAGCTTACTTAGACCAATGGTGAAATAGTGAATATTACAGAATAAAACCTGGAAGTAAGCTCTCTGAGAACCTGCTTCATGTTGTGTGCATTCATCTCACAAAGTTAAATATTTCT
ST-E00144:68:H2LCKCCXX:1:2207:2908:44925 + CtGTTTtCGACCATTCTGCAAGTGgGacGAtTGgGAGCTTACTTAGACCAATGGTGAAATAGTGAATATTACAGaATAAAACCTGGaAGTAaGCTCTcTgAgaacCTGCttCATGTTgTgtgcaTTCATcTCaCAAAGTTAAAtaTTTCTT
ST-E00144:68:H2LCKCCXX:6:1108:10084:18362+ TTCGACCATTCTGCAAGTGGaCGATTGGGAGCttACTTAGACCAATGGTGAAATAGTGAATATTACAGAATAAAACCTGGAAGTAAGCTCTCTGAGAACCTGCTTCATGTTGTGTGCATTCATCTCACAAAGTTAAATATTTCTTTTCAT
ST-E00144:68:H2LCKCCXX:6:2111:7364:18678 + CATTCTGCAAGTGGACGATTGGGAGCTTACTTAGACCAATGGTGAAATAGTGAATATTACAGAATAAAACCTGGAAGTAAGCTCTCTGAGAACCTGCTTCATGTTGTGTGCATTCATCTCACAaAGTTAAATATTTCTTTTCATTCaGCA
ST-E00144:68:H2LCKCCXX:6:1124:14276:31389+ AAGTGGACGATTGGGAGCTTACTTAGACCAATGGTGAAATAGTGAATATTACAGAATAAAACCTGGAAGTAAGCTCTCTGAGAACCTGCTTCATGTTGTGTGCATTCATCTCACAAAGTTAAATATTTCTTTTCATTCAGCAGATAGGAA
ST-E00144:68:H2LCKCCXX:6:2101:4552:3752  + GGGAGCTTACTTAGACCAATGGTGAAATAGTGAATATTACAGAATAAAACCTGGAAGTAAGCTCTCTGAGAACCTGCTTCATGTTGtGTGCATTCATCTCACAAAGtTAAATAttTCTTTTCATTccgcagatAGTgAacaCT
ST-E00144:68:H2LCKCCXX:3:1106:5669:36487 + TAGACCAATGGTGAAATAGTGAATATTACAGAATAAAACCTGGAAGTAAGCTCTCTGAGAACCTGCTTCATGTTGTGTGCATTCATCTCACAAAGTTAAATATTTCTTTTCATTCAGCAGATAGGAAACACTGTTTTTGTAGTACCTGTG
ST-E00144:68:H2LCKCCXX:3:1106:5618:36610 + TAGACCAATGGTGAAATAGTGAATATTACAGAATAAAACCTGGAAGTAAGCTCTCTGAGAACCTGCTTCATGTTGTGTGCATTCATCTCACAAAGTTAAATATTTCTTTTCATTCAGCAGATAGGAAACACTGTTTTTGTAGTACCTGTG
ST-E00144:68:H2LCKCCXX:7:2221:17230:5317 + ACCAATGGTGAAATAGTGAATATTACAGAATAAAACCTGGAAGTAAGCTCTCTGAGAACCTGCTTCATGTTGTGTGCATTCATCTCACAAAGTTAAATATTTCTTTTCATTCAGCAGATAGGaAacACTGTTTTTGTAGTAtctGTGAAG
ST-E00144:68:H2LCKCCXX:6:1220:13098:16164+ AATGGTGAAATAGTGAATATtACAGAATAAAACCTGgaAGTAAGCTCTCTGAgAACCTGCTTCATGTTGTGTGCATtCATCtCacaAagTTAAATATTTCTttTCATTCAGCAcaTaGGaAACaCTGtcTTTgTAGTACCTgtGAaG
ST-E00144:68:H2LCKCCXX:7:1108:14794:19381+ AATAGTGAATATTACAGAATAAAACCTGGAAGTAAGCTCTCTGAGAACCTGCTTCATGTTGTGTGCATTCATCTCACAAAGTTAAATATTTCTTTTCATTCAGCAGATAGGAAACACTGTTTTTGTAGTACCTGTGAAGGGATATTTGGG
ST-E00144:68:H2LCKCCXX:7:1108:14641:20383+ AATAGTGAATATTACAGAATAAAACCTGGAAGTAAGCTCTCTGAGAACCTGCTTCATGTTGTGTGCATTCATCTCACAAAGTTAAATATTTCTTTTCATTCAGCAGATAGGAAACACTGTTTTTGTAGTACCTGTGAAGGGATATTTGGG
ST-E00144:68:H2LCKCCXX:1:1224:18448:29015- ATAAGCTACTtTCTGATGTGTGCATTCATCTCACAGAGTTAAAACTTTCTAtTGATaCAGCAGTTTGGAAGCACTGTTTTCGACCATTCTGCAAGTGGACGATTGGGAGCTTACTTAGACCAATGGTGAAATAGTGAATATTACAGAATA
ST-E00144:68:H2LCKCCXX:7:2204:4522:37805 - TACTTTcTGATGTGTGCATTCATCTCACAGAGTTAAAACTTTCTATTGATTCAGCaGTTTGGAAGCACTGTTTTCGACCATTCTGCAAGTGGACGATTGGGAGCTTACTTAGACCAATGGTGAAATAGTGAATATTACAGAATAAAACCT
ST-E00144:68:H2LCKCCXX:7:1215:21421:22458- TTTCTGATGTGTGCATTCATCTCACAGAGTTAAAACTTTCTATTGATTCAGCAGTTTGGAAGCACTGTTTTCGACCATTCTGCAAGTGGACGATTGGGAGCTTACTTAGACCAATGGTGAAATAGTGAATATTACAGAATAAAACCTGGA
ST-E00144:68:H2LCKCCXX:7:2221:26486:54261- TTTCtGATgTGTGCATTCATCTCACAGAGTTAAAACTTTCTATTGATACAGCAGTTTGGAAGCACTGTTTTCGACCATTCTGCAAGTGGACGATTGGGAGCTTACTTAGACCAATGGTGAAATAGTGAATATTACAGAATAAAACCTGGA
ST-E00144:68:H2LCKCCXX:4:2116:9201:16059 - CTGATGTGTGCATTCAtCTCACAGAGTTAAAACTTTCTATTGATTCAGCAGTTTGGAAGCACTGTTTTCGACCATTCTGCAAGTGGACGATTGGGAGCTTACTTAGACCAATGGTGAAATAGTGAATATTACAGAATAAAACCTGGAAGT
ST-E00144:68:H2LCKCCXX:4:2115:32282:29806- TGTGCatTCATCTCACAGAGTTAAAACTTTCTATTGATTCAGCAGTTTGGAAGCACTGTTTTCGACCATTCTGCAAGTGGACGATTGGGAGCTTACTTAGACCAATGGTGAAATAGTGAATATTACAGAATAAAACCTGGAAGTAAGCTC
ST-E00144:68:H2LCKCCXX:1:1221:28070:24515- TTCATCTCACAGAGTTAAAACTTTCTATTGATACAGCAGTTTGGAAGCACTGTTTTCGACCATTCTGCAAGTGGACGATTGGGAGCTTACTTAGACCAATGGTGAAATAGTGAATATTACAGAATAAAACCTGGAAGTAAGCTCTCTGAG
ST-E00144:68:H2LCKCCXX:3:1213:20995:10152- CTCACAGAgTTAAAACTTTCtATTGATAcAgcagtTTGGAAGcACTGTTTTCGACCATTCTGCAAGTGgacgATTGGGAGCTTACTTAGtCCAATGGTGAAATAGTGAATATgACAGAATAAAACCTGGAAGTAAGCTCTCTGAGAacct
ST-E00144:68:H2LCKCCXX:8:1214:14915:58972- CAGAGTTAAAACTTTCTATTGATACAGCAGTTTGGAAGCACTGTTTTCGACCATTCTGCAAGTGGACGATTGGGAGCTTACTTAGACCAATGGTGAAATAGTGAATATTACAGAATAAAACCTGGAAGTAAGCTCTCTGAGAACCTGCTT
ST-E00144:68:H2LCKCCXX:3:2101:19797:10609- AGTTAAAACTttCTATTGATTCAGCAGTTTGGAAGCACTGTTTTCGACCATTCTGCAAGTGGACGATTGGGAGCTTACTTAGACCAATGGTGAAATAGTGAATATTACAGAATAAAACCTGGAAGTAAGCTCTCTGAGAACCTGCTTCAT
ST-E00144:68:H2LCKCCXX:1:2113:5070:40478 - GTTAAAACTTTCTATTGATTCAGCAGTTTGGAAGCACTGTTTTCGACCATTCTGCAAGTGGACGATTGGGAGCTTACTTAGACCAATGGTGAAATAGTGAATATTACAGAATAAAACCTGGAAGTAAGCTCTCTGAGAACCTGCTTCATG
ST-E00144:68:H2LCKCCXX:8:1105:29856:19557- TAAAACTTTCTATTGATTCAGCAGTTTGGAAGCACTGTTTTCGACCATTCTGCAAGTGGACGATTGGGAGCTTACTTAGACCAATGGTGAAATAGTGAATATTACAGAATAAAACCTGGAAGTAAGCTCTCTGAGAACCTGCTTCATGTT
ST-E00144:68:H2LCKCCXX:6:2220:30029:43238- ACTTTCTATTgATTcaGCAGtTTGGAAGCACtGTTTTCGACCATTCTGCAAGTGGACGATTGGGAGCTTACTTAGACCAATGGTGAAATAGTGAATATTACAGAATAAAACCTGGAAGTAAGCTCTCTGAGAACCTGCTTCATGTTGTGT
ST-E00144:68:H2LCKCCXX:4:1102:12987:25464- TATTGATTCAGCAGTTTGGAAGCACTGTTTTCGACCATTCTGCAAGTGGACGATTGGGAGCTTACTTAGACCAATGGTGAAATAGTGAATATTACAGAATAAAACCTGGAAGTAAGCTCTCTGAGAACCTGCTTCATGTTGTGTGCATTC
ST-E00144:68:H2LCKCCXX:1:2211:10754:43220- TGATTCAGCAGTTTGGAAGCACTGTTTTCGACCATTCTGCAAGTGGACGATTGGGAGCTTACTTAGACCAATGGTGAAATAGTGAATATTACAGAATAAAACCTGGAAGTAAGCTCTCTGAGAACCTGCTTCATGTTGTGTGCATTCATC
ST-E00144:68:H2LCKCCXX:4:1121:22233:3155 - TGATTCAGCAGTTTGGaAGCACTGTTTTCGACCATTCTGCAAGTGgACgTTGcGAGCTTACTTAgACCAATGGTGAAATAGTGAATATTACAGAATAAAACCTGgAAGTAAGCTCTCTGAGAACCTGCTTCATGTTGTGTGCATTCATC
ST-E00144:68:H2LCKCCXX:4:1121:22335:3401 - TGATTCAGCAGTTTGGAAGCACTGTTTTCGACCATTCTGCAAGTGGACGATTGGGAGCTTACTTAGACCAATGGTGAAATAGTGAATATTACAGAATAAAACCTGGAAGTAAGCTCTCTGAGAACCTGCTTCATGTTGTGTGCATTCATC
ST-E00144:68:H2LCKCCXX:7:2215:23411:36030- GATTCAGCAGTttGGAAgCACtgTTTtCGAccAgtCTGCAAGTGGACGATTgGGAGCTTACTTAGacCAAtGGTGAAATAGTGAATATTACAGAATAAAACCTGGAAGTAAGCTCTCTGAGAACCTGCTTCATGTTGTGTGCATTCATCT
ST-E00144:68:H2LCKCCXX:8:2111:7942:45910 - TTCAGCAGTTTGGAAGCACTGTTTTCGACCATTCTGCAAGTGGACGATTGGGAGCTTACTTAGACCAATGGTGAAATAGTGAATATTACAGAATAAAACCTGGAAGTAAGCTCTCTGAGAACCTGCTTCATGTTGTGTGCATTCATCTCA
ST-E00144:68:H2LCKCCXX:8:2111:7749:46174 - TTCAGCAGTTTGGAAGCACTGTTTTCGACCATTCTGCAAGTGGACGATTGGGAGCTTACTTAGACCAATGGTGAAATAGTGAATATTACAGAATAAAACCTGGAAGTAAGCTCTCTGAGAACCTGCTTCATGTTGTGTGCATTCATCTCA
ST-E00144:68:H2LCKCCXX:7:1220:3710:19346 - gaccAttctaCAAgtggAcgaTtGGgAgatTActTAGACCaAtgGTGAAAtAGTGAATATTaCAGAATAAAACCTGGAAGTAAGCTCTCTGAGAACCTGCTTCATGTTGTGTGCATTCATCTCACAAAGTTAAATATTTCTTTTCATTCA
ST-E00144:68:H2LCKCCXX:8:1206:3172:47035 - accgtcatgCAaatgGACgaTTgGgAGcttACtTaaaCCAAtggtGAAATAgtgAATATTaCAGAATAAAACCTgGAAGTAAGCTCTCTGAGAACCTGCTTCATGTTGTGTGCATTCATCTCACAAAGTTAAATATTTCTTTTCATTCAG
ST-E00144:68:H2LCKCCXX:6:1119:19270:53329- TGGACGATTGGGAGCTTACTTAGACCAATGGTGAAATAGTGAATATTACAGAATAAAACCTGGAAGTAAGCTCTCTGAGAACCTGCTTCATGTTGTGTGCATTCATCTCACAAAGTTAAATATTTCTTTTCATTCAGCAGATAGGAAACA
ST-E00144:68:H2LCKCCXX:3:1209:1791:56845 - TtTTTTcGtcCaTggtGcaaggGgGCGattgggagCTTACTTAGAGCAATGGTGAAATAGtGAATATTacAGAATAAAACCTGGAAGTAAGCTCTCTGAGAAgCTGCTTCCTGTTGTGTGCATTCATCTCACAAAGTTAAATATTTCTTT
ST-E00144:68:H2LCKCCXX:8:1117:23888:44345- ATTGGGAGCTTACTTAGACCAATGGTGAAATAGTGAATATTACAGAATAAAACCTGGAAGTAAGCTCTCTGAGAACCTGCTTCATGTTGTGTGCATTCATCTCACAAAGTTAAATATTTCTTTTCATTCAGCAGATAGGAAACACTGTTT
ST-E00144:68:H2LCKCCXX:8:1117:23928:44345- ATTGGGAGCTTACTTAGACCAATGGTGAAATAGTGAATATTACAGAATAAAACCTGGAAGTAAGCTCTCTGAGAACCTGCTTCATGTTGTGTGCATTCATCTCAcAAAGTTAAATATTTCTTTTCATTCAGCAGATAGGAAACACTGTTT
ST-E00144:68:H2LCKCCXX:8:1117:23989:44416- ATTGGGAGCTTACTTAGACCAATGGTGAAATAGTGAATATTACAGAATAAAACCTGGAAGTAAGCTCTCTGAGAACCTGCTTCATGTTGTGTGCATTCATCTCACAAAGTTAAATATTTCTTTTCATTCAGCAGATAGGAAACACTGTTT
ST-E00144:68:H2LCKCCXX:8:2206:17534:32004- TTGGGAGCTTACTTAGACCAAtGGTGAAATAGTGAATATTACAGAATAAAACCTGGAAGTAAGCTCTCTGAGAACCTGCTTCATGTTGTGTGCATTCATCTCACAAAGTTAAATATTTCTTTTCATTCAGCAGATAGGAAACACTGTTTT
ST-E00144:68:H2LCKCCXX:8:1223:18945:44363- TACTTAGACCAATGGTGAAATAGTGAATATTACAGAATAAAACCTGGAAGTAAGCTCTCTGAGAACCTGCTTCATGTTGTGTGCATTCATCTCACAAAGTTAAATATTTCTTTTCATTCAGCAGATAGGAAACACTGTTTTTGTAGAACC
ST-E00144:68:H2LCKCCXX:7:1222:28313:61926- CTTAGACCAATGGTGAAATAGTGAATATTACAGAATAAAACCTGGAAGTAAGCTCTCTGAGAACCTGCTTCATGTTGTGTGCATTCATCTCACAAAGTTAAATATTTCTTTTCATTCAGCAGATAGGAAACACTGTTTTTGTAGTACCTG
ST-E00144:68:H2LCKCCXX:7:1220:5151:22475 - TTAGACCAATGGTGAAATAGTGAATATTACAGAATAAAACCTGGAAGTAAGCTCTCTGAGAACCTGCTTCATGTTGTGTGCATTCATCTCACAAAGTTAAATATTTCTTTTCATTCAGCAGATAGGAAACACTGTTTTTGTAGTACCTGT

ST-E00144:68:H2LCKCCXX:7:1108:25147:41111- TAGACCAATGGTGAAATAGTGAATATTACAGAATAAAACCTGGAAGTAAGCTCtCTGaGAACCTGCTTCATGTTGTGTGCATTCATCTCACAAAGTTAAATATTTCTTTTCATTCAGCAGATAGGAAACACTGTTTTTGTAGTACCTGTG
ST-E00144:68:H2LCKCCXX:7:1108:24862:41779- TAGACCAATGGTGAAATAGTGAATATTACAGAATAAAACCTGGAAGTAAGCTCTCTGAgAACCTGCTTCATGTTGTGTGCATTCATCTCACAAAGTTAAATATTTCTTTTCATTCAGCAGATAGGAAACACTGTTTTTGTAGTACCTGTG
ST-E00144:68:H2LCKCCXX:4:1116:31724:53346- GACCAATGGTGAAATAGTGAATATTACAGAATAAAACCTGGAAGTAAGCTCTCTGAGAACCTGCTTCATGTTGTGTGCATTCATCTCACAAAGTTAaATATTTCTTTTCATTCAGCAGATAGGAAACACTGTTTTTGTAGTACCTGTGAA
ST-E00144:68:H2LCKCCXX:7:2206:23218:64703- TGAAATAGTGAATATTACaGAATAaaaCCTgGaAGTAaGCTCTCTGAGAACCTGCTTCATGTTGTGTGCATTCATCTCACAAAGTTAAATATTTCTTTTCATTCAGCAGATAGGAAACACTGTTTTTGTAGAACCTGTGAAGGGATATTT
ST-E00144:68:H2LCKCCXX:7:1103:30211:53188- aAataGtgaatcttACAGaTaAcACCtggAagtaAgctcTCtGagatCCTgttTcATGTTGTGTGCATTCATCTCACAAAGTTAAATATTTCTTTTCATTCAGCAGATAGGAAACACTGTTTTTGTAGTACCTGTGAAGGGATATTTGG
ST-E00144:68:H2LCKCCXX:1:1209:24071:40337- AATAGTGAATATTACAGAATAAAACCTGGAAGTAAGCTCTCTGAGAACCTGCTTCATGTTGTGTGCATTCATCTCACAAAGTTAAATATTTCTTTTCATTCAGCAGATAGGAAACACTGTTTTTGTAGTACCTGTGAAGGGATATTTGG
ST-E00144:68:H2LCKCCXX:6:1102:31856:5510 - gaataTTACAtAATaaaACCTGGaaGTaaGCTCTGagaaCCTGCTTCATGTTGTGTGCATTCATCTCACAAAGTTAAATATTTCTTTTCATTCAGCAGATAGGAAACACTGTTTTTGTAGAACCTGTGAAGGGATATTTGGGATCACA
ST-E00144:68:H2LCKCCXX:6:1102:31947:5528 - GaATaTtcCAgaATAaAaCcTGGAAGTAAGCTCTCTGaGAACCTGCTTCATGTTGTGTGCATTCATCTCACAAAGTTAAATATTTCTTTTCATTCAGCAGATAGGAAACACTGTTTTTGTAGAACCTGTGAAGGGATATTTGGGATCACA
ST-E00144:68:H2LCKCCXX:4:2118:24862:18221- CAGAATAAaaCCTGGAAGTAAGCTCTCTGAGAACCTGCTTCATGTTGTGTGCATTCATCTCACAAAGTTAAATATTTCTTTTCATTCAGCAGATAGGAAACACTGTTTTTGTAGTACCTGTGAAGGGATATTTGGGATCACAGTGAGGCC
ST-E00144:68:H2LCKCCXX:1:2112:26304:7286 - AGAATAAAACCTGGAAGTAAGCTCTCTGAGAACCTGCTTCATGTTGTGTGCATTtATCTCACAAAGTTAAATATTTCTTTTCATTCAGCAGATAGGAAACACTGTTTTTGTAGTACCTGTGAAGGGATATTTGGGATCACAGTGAGGCCT
ST-E00144:68:H2LCKCCXX:8:2105:1527:19504 - GTAAAAgGAAATAtCCTAGAATAAAACCTGGAAGTCAGCTCTCTGAGAACCTGCTTCATGTTGTGTGCATTCATCTCACAAAGTTAAATATTTCTTTTCATTCAGCAGATAGGAAACACTGTTTTTGTAGTACCTGTGAAGGGATATTTG
ST-E00144:68:H2LCKCCXX:8:2216:9262:49602 - AGAATAAAACCTGGAAGTAAGCTCTCTGAGAACCTGCTTCATGTTGTGTGCATTCATCTCACAAAGTTAAATATTTCTTTTCATTCAGCAGATAGGAAACACTGTTTTTGTAGAACCTGTGAAGGGATATTTGGGATCACAGTGAGGCCT
ST-E00144:68:H2LCKCCXX:8:2223:25745:16832- AAAAagaAATATCCtAGAATAAAACCTGGAAGTAAGCTCTCTGAGAACCTGCTTCATGTTGTGTGCATTCATCTCACAAAGTTAAATATTTCTTTTCATTCAGCAGATAGGAAACACTGTTTTTGTAGTACCTGTGAAGGGATATTTG
ST-E00144:68:H2LCKCCXX:8:2223:27055:17799- GTAAAAGGAAATATCCTAGAATAAAACCTGGAAGTAAGCTCTCTGAGAACCTGCTTCATGTTGTGTGCATTCATCTCACAAAGTTAAATATTTCTTTTCATTCAGCAGATAGGAAACACTGTTTTTGTAGTACCTGTGAAGGGATATTTG
ST-E00144:68:H2LCKCCXX:8:2223:26852:17869- GTAAAAGGAAATATCCTAGAATAAAACCTGGAAGTAAGCTCTCTGAGAACCTGCTTCATGTTGTGTGCATTCATCTCACAAAGTTAAATATTTCTTTTCATTCAGCAGATAGGAAACACTGTTTTTGTAGTACCTGTGAAGGGATATTTG
------------------------------------------------------------------------------------------------------------------------------------------------------------------------------------------------------------------------------
ST-E00192:51:H2KG3CCXX:8:1209:7151:48371 + TGATAAGCTACTTTCTGATGTGTGCATTCATCTCACAGAGTTAAAACTTTcTATTGATACAGCAGTTTGGAAGCACTGTTTTCGaCCATTCTGCAAGTGGACGATTGGGaGCTTACTTAGACCAATGGTGAAATAGTGAATATTACAGaA
ST-E00192:51:H2KG3CCXX:3:1217:17077:35713+ GATAAGCTATTTTCTGATGTGTGCATTCATCTCACAGAGTTAAAACTTTCTATTGATTCAGCAGTTTGGAAGCACTGTTTTCGACCATTCTGCAAGTGGACGATTGGGAGCTTACTTAGACCAATGGTGAAATAGTGAATATTACAGAAT
ST-E00192:51:H2KG3CCXX:3:1217:17027:35731+ GaTAAGCTATTTTCTGATGTGTGCATTCATCTCACAGAGTTAAAACTTTCTATTGATTCAGCAGTTTGGAAGCACTGTTTTCGACCATTCTGCAAGTGGACGATTGGGAGCTTACTTAGACCAATGGTGAAATAGTGAATATTACAGAAT
ST-E00192:51:H2KG3CCXX:2:2213:5699:7040  + GCTACTTTCTGATGTGTGCATTCATCTCACAGAGTTAAAACTTTCTATTGATTCAGCAGTTTGGAAGCACTGTTTTCGACCATTCTGCAAGTGGACGATTGGGAGCTTACTTAGACCAATGGTGAAATAGTGAATATTACAGAATAAAAC
ST-E00192:51:H2KG3CCXX:3:2115:7414:71648 + TACTTTCTGATGTGTGCATTCATCTCACAGAGTTAAAACTTTCTATTGATACAGCAGTTTGGAAGCACTGTTTTCGACCATTCTGCAAGTGGACGATTGGGAGCTTACTTAGACCAATGGTGAAATAGTGAATATTACAGAATAAAACCT
ST-E00192:51:H2KG3CCXX:5:1222:17727:27556+ ACTTTCTGATGTGTGCATTCATCTCACAGAGTTAAAACTTTCTATTGATTCAGCAGTTTGGAAGCACTGTTTTCGACCATTCTGCAAGTGGACGATTGGGAGCTTACTTAGACCAATGGTGAAATAGTGAATATTACAGaATAAAACCTG
ST-E00192:51:H2KG3CCXX:1:2205:3476:72316 + GTGCATTCATCTCACAGAGTTAAAACTTTCTATTGATACAGCAGTTTGGAAGCACTGTTTTCGACcATTCTGCAAGTGGACGATTGGGAGCTTACTTAGACCAATGGTGAAATAGTGAATATTACAGAATAAAAcCTGGAAGTAAGCCAA
ST-E00192:51:H2KG3CCXX:8:1222:21706:30861+ ATTCATCTCACAGAGTTAAAACTTTCTATTGATTCAGCAGTTTGGAAGCACTGTTTTCGACCATTCTGCAAGTGGACGATTGGGAGCTTACTTAGACCAATGGTGAAATAGTGAATATTACAGAATAAAACCTGGAAGTAAGCTCTCTGA
ST-E00192:51:H2KG3CCXX:6:2119:13819:51377+ ATCTCACAGAGTTAAAACTTTCTATTGATTCAGCAGTTTGGAAGCACTGTTTTCGACCATTCTGCAAGTGGACGATTGGGAGCTTACTTAGACCAATGGTGAAATAGTGAATATTACAGAATAAAACcTGGAAGTAAGCTCTCTGAGAAC
ST-E00192:51:H2KG3CCXX:6:2119:13921:51377+ ATCTCACAGAGTTAAAACTTTCTATTGATTCAGCAGTTTGGAAGCACTGTTTTCGACCATTCTGCAAGTGGACGATTGGGAGCTTACTTAGACCAATGGTGAAATAGTGAATATTACAGAATAAAACCTGGAAGTAAGCTCTCTGAGAAC
ST-E00192:51:H2KG3CCXX:1:1107:16154:47510+ AGAGTTAAAACTTTCTATTGATTCAGCAGTTTGGAAGCACTGTTTTCGACCATTCTGCAAGTGGACGATTGGGAGCTTACTTAGACCAATGGTGAAATAGTGAATATTACAGAATAAAACCTGGAAGTAAGCTCTCTGAGAACCTGCTTC
ST-E00192:51:H2KG3CCXX:1:1107:16174:47510+ AGAGTTAAAACTTTCTATTGATTCAGCAGTTTGGAAGCACTGTTTTCGACCATTCTGCAAGTGGACGATTGGGAGCTTACTTAGACCAATGGTGAAATAGTGAATATTACAGAATAAAACCTGGAAGTAAGCTCTCTGAGAACCTGCTTC
ST-E00192:51:H2KG3CCXX:1:1118:32861:39458+ AGAGTTAAAACTTTCTATTGATTCAGCAGTTTGGAAGCACTGTTTTCGACCATTCTGCAAGTGGACGATTGGGAGCTTACTTAGACCAATGGTGAAATAGTGAATATTACAGAATAAAACCTGGAAGTAAGCTCTCTGAGAACCTGCTTC
ST-E00192:51:H2KG3CCXX:1:2106:13332:50674+ GTTAAAACTTTCTATTGATTCAGCAGTTTGGAAGCACTGTTTTCGACCATTCTGCAAGTGGACGATTGGGAGCTTACTTAGACCAATGGTGAAATAGTGAATATTACAGAATAAAACCTGGAAGTAAGCTCTCTGAGAACCTGCTTCATG
ST-E00192:51:H2KG3CCXX:1:2106:13799:51307+ GTTAAAACTTTCTATTGATTCAGCAGTTTGGAAGCACTGTTTTCGACCATTCTGCAAGTGGACGATTGGGAGCTTACTTAGACCAATGGTGAAATAGTGAATATTACAGAATAAAACCTGGAAGTAAGCTCTCTGAGAACCTGCTTCATG
ST-E00192:51:H2KG3CCXX:8:2212:13870:11101+ ATTGATTCAGCAGTTTGGAAGCACTGTTTTCGACCATTCTGCAAGTGGACGATTGGGAGCTTACTTAGACCAATGGTGAAATAGTGAATATTACAGAATAAAaACCTGGAAGTAAGCTCTCTGAGAACCTGCTTCATGTtGTGTGCATTCA
ST-E00192:51:H2KG3CCXX:1:1112:5811:54770 + TTTGGAAGCACTGTTTTCGACCATTCTGCAAGTGGACGATTGGGAGCTTACTTAGACCAATGGTGAAATAGTGAATATTACAGAATAAAACCTGGAAGTAAGCTCTCTGAGAACCTGCTTCATGTTGTGTGCATTCATCTCACAAAGTTA
ST-E00192:51:H2KG3CCXX:3:1224:31358:22827+ TGGAAGCACTGTTTTCGACCATTCTGCAAGTGGACGATTGGGAGCTTACTTAGACCAATGGTGAAATAGTGAATATTACAGAATAAAACCTGGAAGTAAGCTCTCTGAGAACCTGCTTCATGTTGTGTGCATTCATCTCACAAAGTTAAA
ST-E00192:51:H2KG3CCXX:3:1224:31348:22845+ TGGAAGCACTGTTTTCGACCATTCTGCAAGTGGACGATTGGGAGCTTACTTAGACCAATGGTGAAATAGTGAATATTACAGAATAAAACCTGGAAGTAAGCTCTCTGAGAACCTGCTTCATGTTGTGTGCATTCATCTCACAAAGTTAAA
ST-E00192:51:H2KG3CCXX:2:1113:9739:29367 + ACTGTTTTCGACCATTCTGCAAGTGGACGATTGGGAGCTTACTTAGACCAATGGTGAAATAGTGAATATTACAGAATAAAACCTGGAAGTAAGCTCTCTGAGAACCTGCTTCATGTTGTGTGCATTCATCTCACAAAGTTAAATATTTCT
ST-E00192:51:H2KG3CCXX:6:2223:6755:25745 + ACTGTTTTCGACCATTCTGCAAGTGGACGATTGGGAGCTTACTTAGACCAATGGTGAAATAGTGAAtATTACAGAATAAAACCTGGAAGTAAGCTCTCTGAGAACCTGCTTCAtGTTGTGTGCaTTCATCTCACAAAGTTAaATATtTct
ST-E00192:51:H2KG3CCXX:8:1223:8805:43396 + ACTGTTTTCGACCATTCTGCAAGTGGACGATTGGGAGCTTACTTAGACCAATGGTGAAATAGTGAATATTACAGAATAAAACCTGGAAGTAAGCTCTCTGAGAACCTGCTTCATGTTGTGTGCATTCATCTCACAAAGTTAAATATTTCT
ST-E00192:51:H2KG3CCXX:6:2111:26324:49057+ GACCATTCTGCAAGTGGACGATTGGGAGCTTACTTAGACCAATGGTGAAATAGTGAATATTACAGAATAAAACCTGGAAGTAAGCTCTCTGAGAACCTGCTTCATGTTGTGTGCATTCATCTCACAAAGTTAAATATTTCTTTTCATTCA
ST-E00192:51:H2KG3CCXX:5:1222:6227:32320 + TCTGCAAGTGGACGATTGGGAGCTTACTTAGACCAATGGTGAAATAGTGAATATTACAGAATAAAACCTGGAAGTAAGCTCTCTGAGAACCTGCTTCATGTTGTGTGCATTCATCTCACAAAGTTAAATATTTCTTTTCATTCAGCAGAT
ST-E00192:51:H2KG3CCXX:2:1211:15839:24532+ aaGTGGACGATTGGGAGCTTACTTAGACCAATGGTGAAATAGTGAATAtTACAGAATAAAACCTGGAAGTAAGCTCTCTGAGAACCTGCTTCATGTTGTGTGCATTCATCTCACAAAGTTAAATATTTCTTTTCATTCAGCAGATAGGAA
ST-E00192:51:H2KG3CCXX:2:1211:15839:24603+ AAGTGGACGATTGGGAGCTTACTTAGACCAATGGTGAAATAGTGAATATTACAGAATAAAACCTGGAAGTAAGCTCTCTGAGAACCTGCTTCATGTTGTGTGCATTCATCTCACAAAGTTAAATATTTCTTTTCATTCAGCAGATAGGAA
ST-E00192:51:H2KG3CCXX:5:1121:25898:14142+ GGACGATTGGGAGCTTACTTAGACCAATGGTGAAATAGTGAATATTACAGAATAAAACCTGGAAGTAAGCTCTCTGAGAACCTGCTTCATGTTGTGTGCATTCATCTCACAAAGTTAAATATTTCTTTTCATTCAGCAgATAGGAAACAC
ST-E00192:51:H2KG3CCXX:8:1121:27948:64387+ ACGATTGGGAGCTTACTTAGACCAATGGTGAAATAGTGAATATTACAGAATAAAACCTGGAAGTAAGCTCTCTGAGAACCTGCTTCATGTTGTGTGCATTCATCTCACAAAGTTAAATATTTCTTTTCATTCAGCAGATAGGAAACACTG
ST-E00192:51:H2KG3CCXX:8:1121:27968:64422+ ACGATTGGGAGCTTACTTAGACCAATGGTGAAATAGTGAATATTACAGAATAAAACCTGGAAGTAAGCTCTCTGAGAACCTGCTTCATGTTGTGTGCATTCATCTCACAAAGTTAAATATTTCTTTTCATTCAGCAGATAGGAAACACTG
ST-E00192:51:H2KG3CCXX:6:2124:21137:36663+ CGATTGGGAGCTTACTTAGACCAATGGTGAAATAGTGAATATTACAGAATAAAACCTGGAAGTAAGCTCTCTGAGAACCTGCTTCATGTTGTGTGCATTCATCTCACAAAGTTAAATATTTCTTTTCATTCAGCAGATAGGAAACACTGT
ST-E00192:51:H2KG3CCXX:3:1223:10947:5194 + ACTTAGACCAATGGTGAAATAGTGAATATTACAGAATAAAACCTGGAAGTAAGCTCTCTGAGAACCTGCTTCATGTTGTGTGCATTCATCTCACAAAGTTAAATATTTCTTTTCATTCAGCAGATAGGAAACACTGTTTTTGTAGTACCT
[illegible]
```

```
[illegible]
ST-E00192:51:H2KG3CCXX:3:2215:4583:20014 + TAGTGAATATTACAGAATAAAACCTGGAAGTAAGCTCTCTGAGAACCTGCTTCATGTTGTGTGCATTCATCTCACAAAGTTAAATATTTCTTTTCATTCAGCAGATAGGAAACACTGTTTTTGTAGAACCTGTGAAGGGATATTTGGGAT
ST-E00192:51:H2KG3CCXX:3:1124:26263:22194+ ATTACAGAATAAAACCTGGAAGTAAGCTCTCTGAGAACCTGCTTCATGTTGTGTGCATTCATCTCACAAAGTTAAATATTTCTTTTCATTCAGCAGATAGGAAACACTGTTTTTGTAGTACCTGTGAAGGGATATTTGGGATCACAGTGA
ST-E00192:51:H2KG3CCXX:3:1124:26304:22194+ aTTACAGAATAAAACCTGGAAGTAAGCTCTCTGAGAACCTGCTTCATGtTGTGTGcATTCATcTCACAAaGTTAAATATTTCTtTgCATTCAGCAGAtAGGaAACACTGTTTTTGTAGTACCTGTGAAGGGATATTTGGGctcacaGTGa
ST-E00192:51:H2KG3CCXX:6:1105:30871:9431 + ACAGAATAAAACCTGGAAGTAAGCTCTCTGAGAACCTGCTTCATGTTGTGTGCATTCATCTCACAAAGTTAAATATTTCTTTTCATTCAGCAGATAGGAAACACTGTTTTTGTAGTACCTGTGAAGGGATATTTGGGATCACAGTGAGGC
ST-E00192:51:H2KG3CCXX:6:1105:30881:9448 + ACAGAATAAAACCTGGAAGTAAGCTCTCTGAGAACCTGCTTCATGTTGTGTGCATTCATCTCACAAAGTTAAATATTTCTTTTCATTCAGCAGATAGGAAACACTGTTTTTGTAGTACCTGTGAAGGGATATTTGGGATCACAGTGAGGC
ST-E00192:51:H2KG3CCXX:6:1113:23035:69556+ ACAGAATAAAACCTGGAAGTAAGCTCTCTGAGAACCTGCTTCATGTTGTGTGCATTCATCTCACAAAGTTAAATATTTCTTTTCATTCAGCAGATAGGAAACACTGTTTTTGTAGTACCTGTGAAGGGATATTTGGGATCACAGTGAGGC
ST-E00192:51:H2KG3CCXX:6:1113:22964:69573+ ACAGAATAAAACCTGGAAGTAAGCTCTCTGAGAACCTGCTTCATGTTGTGTGCATTCATCTCACAAAGTTAAATATTTCTTTTCATTCAGCAGATAGGAAACACTGTTTTTGTAGTACCTGTGAAGGGATATTTGGGATCACAGTGAGGC
ST-E00192:51:H2KG3CCXX:6:1113:23076:69661+ ACAGAaTAAAACCTGGAAGTAAGCTCTCTgAGAAcCTGcTTCATGTTGTGTGCATTCATcTCACAAAGTTAAaTATTtCTTTTCATTCAGCAGATAGGAAACACTGTtTTTGTAGTACCTgTGAAgggataTTTGGGAtCACAGTGAGGC
ST-E00192:51:H2KG3CCXX:3:1202:11556:55720- TGATAAGCTACTTTCTGATGTGTGCATTCATCTCACAGAGTTAAAACTTTCTATTGATTCAGCAGTTTGGAAGCACTGTTTTCGACCATTCTGCAAGTGGACGATTGGGAGCTTACTTAGACCAATGGTGAAATAGTGAATATTACAGAA
ST-E00192:51:H2KG3CCXX:8:1112:31693:23794- tGTGcATTCATCTCACAGAGTTAAAACTTTCTATTGATTCAGCAGTTTGgAaGCACTGTTTTCGACCaTTCTGCAAGTGGACGATTGaGAGCTTACTTAGACCAAtGGTGAAATAGTGAATATTACAGAATAAAACCTGGAAGTAAGCTC
ST-E00192:51:H2KG3CCXX:1:2106:29643:57372- TGCATTCATCTCACAGAGTTAAAACTTTCTATTGATTCAGCAGTTTGGAAGCACTGTTTTCGACCATTCTGCAAGTGGACGATTGGGAGCTTACTTAGACCAATGGTGAAATAGTGAATATTACAGAATAAAACCTGGAAGTAAGCTCTC
ST-E00192:51:H2KG3CCXX:1:2215:19544:36258- TCTCACAGAGTTAAAACTTTCTATTGATACAGCAGTTTGGAAGCACTGTTTTCGACCATTCTGCAAGTGGACGATTGGGAGCTTACTTAGACCAATGGTGAAATAGTGAATATTACAGAATAAAACCTGGAAGTAAGCTCTCTGAGAACC
ST-E00192:51:H2KG3CCXX:5:1202:18945:39722- CAGAGTTAAAACTTTCTATTGATTCAGCAGTTTGGAAGCACTGTTTTCGACCATTCTGCAAGTGGACGATTGGGAGCTTACTTAGACCAATGGTGAAATAGTGAATATTACAGAATAAAACCTGGAAGTAAGCTCTCTGAGAACCTGCTT
ST-E00192:51:H2KG3CCXX:1:1217:9850:48442 - TTAAAACTTTCTATTGATTCAGCAGTTTGGAAGCACTGTTTTCGACCATTCTGCAAGTGGACGATTGGGAGCTTACTTAGACCAATGGTGAAATAGTGAATATTACAGAATAAAACCTGGAAGTAAGCTCTCTGAGAACCTGCTTCATGT
ST-E00192:51:H2KG3CCXX:5:1219:5912:6249  - TTaAAACTTTCTATTGATACAGCAGTTTGGAAGCACTGTTTTCGACCATTcTGCAAGTGGACGATTGgGAGCTTACTTAGACCAATGGTGAAATAGTGAATATTACAGAATAAAACCTGGAAGTAAGCTCTCTGAGAACCTGCTTCATGT
ST-E00192:51:H2KG3CCXX:5:1216:20082:67885- TCTATTGATTCAGCAGTTTGGAAGCACTGTTTTCGACCATTCTGCAAGTGGACGATTGGGAGCTTACTTAGACCAATGGTGAAATAGTGAATATTACAGAATAAAACCTGGAAGTAAGCTCTCTGAGAACCTGCTTCATGTTGTGTGCAT
ST-E00192:51:H2KG3CCXX:1:1214:25045:49303- TATTGATTCAGCAGTTTGGAAGCACTGTTTTCGACCATTCTGCAAGTGGACGATTGGGAGCTTACTTAGACCAATGGTGAAATAGTGAATATTACAGAATAAAACCTGGAAGTAAGCTCTCTGAGAACCTGCTTCATGTTGTGTGCATTC
ST-E00192:51:H2KG3CCXX:2:1102:29653:2610 - TTCAGCAGTTTGGAAGCACTGTTTTCGACCATTCTGCAAGTGGACGATTGGGAGCTTACTTAGACCAATGGTGAAATAGTGAATATTACAGAATAAAACCTGGAAGTAAGCTCTCTGAGAACCTGCTTCATGTTGTGTGCATTCATCTCA
ST-E00192:51:H2KG3CCXX:2:1102:29613:2645 - TTCAGCAGTTTGGAAGCACTGTTTTCGACCATTCTGCAAGTGGACGATTGGGAGCTTACTTAGACCAATGGTGAAATAGTGAATATTACAGAATAAAACCTGGAAGTAAGCTCTCTGAGAACCTGCTTCATGTTGTGTGCATTCATCTCA
ST-E00192:51:H2KG3CCXX:5:1203:29663:40741- TTCAgcagtatgGAAGCACtGTTTTcGACCATTCTGcAAGTTGAcGATTGGGAGCTTACTTAGACCAATGGTGAAATaGtGAATATTACAgAATAAAACCTGGAAGTAAGCTCTCTGAGAACCTGCTTCATGTTGTGTGCATTCATCTCA
ST-E00192:51:H2KG3CCXX:1:2218:11820:16691- TCAGCAGTTTGGAAGCACTGTTTTCGACCATTCTGCAAGTGGACGATTGGGAGCTTACTTAGACCAATGGTGAAATAGTGAATATTACAGAATAAAACCTGGAAGTAAGCTCTCTGAGAACCTGCTTCATGTTGTGTGCATTCATCTCAC
ST-E00192:51:H2KG3CCXX:1:2218:12297:16709- TCAGCAGTTTGGAAGCACTGTTTTCGACCATTCTGCAAGTGGACGATTGGGAGCTTACTTAGACCAATGGTGAAATAGTGAATATTACAGAATAAAACCTGGAAGTAAGCTCTCTGAGAACCTGCTTCATGTTGTGTGCATTCATCTCAC
ST-E00192:51:H2KG3CCXX:3:1107:12226:33217- TCAgCAgTTTgGAAGCACTGTTTTcGAcCATtCTGCAAGTGGACGATTGGGAGCTTACTTAGACCAATGGTGAAATAGTGAATATTACAGAATAAAACCTGGAAGTAAGCTCTCTGAGAACCTGCTTCATGTTGTGTGCATTCATCTCAC

ST-E00192:51:H2KG3CCXX:2:1106:10348:12525- AGCAGTTTGGAAGCACTGTTTTCGACCATTCTGCAAGTGGACGATTGGGAGCTTACTTAGACCAATGGTGAAATAGTGAATATTACAGAATAAAACCTGGAAGTAAGCTCTCTGAGAACCTGCTTCATGTTGTGTGCATTCATCTCACAA
ST-E00192:51:H2KG3CCXX:1:1219:19168:68237- CAGTTTtGAAGCACTgTTTTCGACCATTCTGCAAGtgGACgATTgGgAGCTTACTtAGACCAATGGTGAAATAGTGAATATTACAgAATAAAACCtGgAAGTAAGCTCTCTGAGAACCTGCTTCATGTTGTGTGCATTCATCTCACAAAG
ST-E00192:51:H2KG3CCXX:3:1108:21391:60519- CAGTTTGGAAGCaCTGTTTTCGACCATTCTGCAAGTGGAcGATTgGGAGCTTACTTAGACCaaTGGTGAAATAGTGAATATTACAGAatgAAACCTGGAAGTAaGCTCTCTGAGAACCTGCttCaTGTTGTGTGCaTTCATCTCACAAaG
ST-E00192:51:H2KG3CCXX:3:1108:21340:60572- CAGTTTGGAAGCACTGTTTTCGACCATTCTGCAAGTGGACGATTGGGAGCTTACTTAGACCAATGGTGAAATAGTGAATATTACAGAATAAAACCTGGAAGTAAGCTCTCTGAGAACCTGCTTCATGTTGTGTGCATTCATCTCACAAAG
ST-E00192:51:H2KG3CCXX:6:1108:22964:64932- CAGTTTGGAAGCACTGTTTTCGACCATTCTGCAAGTGGACGATTGGGAGCTTACTTAGACCAATGGTGAAATAGTGAATATTACAGAATAAAACCTGGAAGTAAGCTCTCTGAGAACCTGCTTCATGTTGTGTGCATTCATCTCACAAAG
ST-E00192:51:H2KG3CCXX:3:1206:6349:63016 - AGTTTGGAAGCACTGTTTTCGACCATTCTGCAAGTGGACGATTGGGAGCTTACTTAGACCAATGGTGAAATAGTGAATATTACAGAATAAAACCTGGAAGTAAGCTCTCTGAGAACCTGCTTCATGTTGTGTGCATTCATCTCACAAAGT
ST-E00192:51:H2KG3CCXX:2:1103:3852:46631 - GAAGcAcTgttTTCGaCCATtCTGCAAGTGGaCgATTGGGAGCtTACTTAGACcAATGGtGAAATAGTGAATATTACAGaaTAAaaCCttGAAGTAAGCTCTCTGAGAACCTGCTTCATGTTGTGTGCATTCATCtCACAAAGTTAAATA
ST-E00192:51:H2KG3CCXX:1:1115:7506:21456 - gCACTGTTtacgACCATTCTgcAAgtgGACGATTggGAGCTTACTTAGACCAATGGTGAAATAGTgaaTATTACaGAATAaaACCTGGAAGTAAGCTCTCTGAGAACCTGCTTCATGTTGTGTGCATTCATCTCACAAAGTTAAATATTT
ST-E00192:51:H2KG3CCXX:6:1202:15829:44943- TTCGACCATTCTGCAAGTGGACGATTGGGAGCTTACTTAGACCAATGGTGAAATAGTGAATATTACAGAATAAAACCTGGAAGTAAGCTCTCTGAGAACCTGCTTCATGTTGTGTGCATTCATCTCACAAAGTTAAATATTTCTTTTCAT
ST-E00192:51:H2KG3CCXX:6:1202:15819:45031- TTCGACCATTCTGCAAGTGGACGATTGGGAGCTTACTTAGACCAATGGTGAAATAGTGAATATTACAGAATAAAACCTGGAAGTAAGCTCTCTGAGAACCTGCTTCATGTTGTGTGCATTCATCTCACAAAGTTAAATATTTCTTTTCAT
ST-E00192:51:H2KG3CCXX:3:1121:3771:42130 - ATTCTGCAAGTGGACGATTGGGAGCTTACTTAGACCAATGGTGAAATAGTGAATATTACAGAATAAAACCTGGAAGTAAGCTCTCTGAGAACCTGCTTCATGTTGTGTGCATTCATCTCACAAAGTTAAATATTTCTTTTCATTCAGCAG
ST-E00192:51:H2KG3CCXX:8:1104:29359:15812- CGATTGGGAGCTTACTTAGACCAATGGTgAAATAGTGAATATTACAGAATAAAACCTGGAAGTAAGCTCTCTGAGAACCTGCTTCATGTTGTGTGCATTCATCTCACAAAGTTAAATATTTCTTTTCATTCAGCAGATAGGAAACACTGT
ST-E00192:51:H2KG3CCXX:1:1215:2979:39985 - TTGGGAGCTTACTTAGaCCAATGtTGAAATAGTGAATATTACAGAATAAAACCTGgAAGTAAGCTCTCTGAGAACCTGCTTCATGTTGTGTGCATTCATCTCACAAAGTTAAATATTTCTTTTCATTCAGCAGATAGGAAACACTGTTTT
ST-E00192:51:H2KG3CCXX:1:1215:2999:39985 - gTggGAgcttActtaGACcAatggTGAAATAGtGAATATtACAGAATAAAACCTggAAGTAAGCTCTCTGAGAACCTGCTTCATGTTGTGTGCATTCATCTCACAAAGTTAAATATTTCTTTTCATTCAGCAGATAGGAAACACTGTTTT
ST-E00192:51:H2KG3CCXX:2:2124:1801:39845 - CtTtCTTAgaCCAAttGTGAAATAGTGAATATTACAGgATAAAACCTGGAAGTgAGCtCTcTGAgaACCTGCTTCATGTTGTGTGCAtTCATCTCACAAAGTTAAATAtTTCTTTTCATTCAGCAGATAGGAaACaCTGTTTTTGTAGAA
ST-E00192:51:H2KG3CCXX:8:1206:3202:44416 - TTacTTAGAcCAATGGTGAAATAGTGAATATTACAGAATAAAACCTgGAAGTAAGCTCTCTGAgAACCTGCTTCATGTTGTGTGCATTCATCTCACAAAGTTAAATATTTCTTTTCATTCAGCAGATAGGAAACACTGTTTTTGTAGTAC
ST-E00192:51:H2KG3CCXX:5:2108:11749:29578- TTAGACCAATGGTGAAATAGTGAATATTACAGAATAAAACCTGGAAGTAAGCTCTCTGAGAACCTGCTTCATGTTGTGTGCATTCATCTCACAAAGTTAAATATTTCTTTTCATTCAGCAGATAGGAAACACTGTTTTTGTAGTACCTGT
ST-E00192:51:H2KG3CCXX:2:1213:16225:8569 - AGACCAATGGTGAAATAGTGAATATTACAGAATAAAACCTGGAAGTAAGCTCTCTGAGAACCTGCTTCATGTTGTGTGCATTCATCTCACAAAGTTAAATATTTCTTTTCATTCAGCAGATAGGAAACACTGTTTTTGTAGAACCTGTGA
ST-E00192:51:H2KG3CCXX:2:1216:30008:39933- GACCAATGGTGAAATAGTGAATATTACAgAATAAAACCTGGAAGTAAGCTCTCTGAGAACCTGCTTCATGTTGTGTGCATTCATCTCACAAAGTTAAATATTTCTTTTCATTCAGCAGATAGGAAACACTGTTTTTGTAGTACCTGTGAA
ST-E00192:51:H2KG3CCXX:2:1201:22112:49742- GgtgAAATAGgGAATATTACAGAATAAAACCTGGAAGTAAGCTCTCTGAGAACCTGCTTCATGTTGTGTGCATTCATCTCACAAAGTTAAATATTTCTTTTCATTCAGCAGATAGGAAACACTGTTTTTGTAGTACCTGTGAAGGGATAT
ST-E00192:51:H2KG3CCXX:3:2114:7039:50006 - GAAATAGTGAATATTACAGAATAAAACCTGGAAGTAAGCTCTCTGAGAACCTGCTTCATGTTGTGTGCATTCATCTCACAAAGTTAAATATTTCTTTTCATTCAGCAGATAGGAAACACTGTTTTTGTAGTACCTGTGAAGGGATATTTG
ST-E00192:51:H2KG3CCXX:1:2113:6136:47211 - ATatTACAGAATAAAAcCTGGAAGTAAGCTCTCTGAgAACCTGCTTCATGtTGTGTGCATTCATCTCACAAAGTTAAATATTTCtTtgCATTCAGCAGATAGGAAACACTGTTTTTGTAGTaCCTGTGAAGGGATATTTGGGATCACAG
ST-E00192:51:H2KG3CCXX:3:2211:11799:64475- TATtAcaGAATAAAACCTGGAAGTAAGCTCTCTGAGAACCTGCTTCATGTTGTGTGCATTCATCTCACAAAGTTAAATATTTCTTTTCATTCAGCAGATAGGAAACACTGTTTTTGTAGTACCTGTGAAGGGATATTTGGGATCACAGTG
ST-E00192:51:H2KG3CCXX:3:2211:11749:64774- TATTACAGAATAAAACCTgGAAGTAAGCTCTCTGAGAACCTGCTTCATGTTGTGTGCATTCATCTCACAAAGTTAAATATTTCTTTTCATTCAGCAGATAGGAAACACTGTTTTTGTAGTACCTGTGAAGGGATATTTGGGATCACAGTG
ST-E00192:51:H2KG3CCXX:1:2110:8907:58339 - AGAATAAAACCTGGAAGTAAGCTCTCTGAGAACCTGCTTCATGTTGTGTGCATTCATCTCACAAAGTTAAATATTTCTTTTCATTCAGCAGATAGGAAACACTGTTTTTGTAGTACCTGTGAAGGGATATTTGGGATCACAGTGAGGCCT
ST-E00192:51:H2KG3CCXX:1:2110:8907:58410 - AGAATAAAACCTGGAAGTAAGCTCTCTGAGAACCTGCTTCATGTTGTGTGCATTCATCTCACAAAGTTAAATATTTCTTTTCATTCAGCAGATAGGAAACACTGTTTTTGTAGTACCTGTGAAGGGATATTTGGGATCACAGTGAGGCCT
```

```
                      61875980  61876000  61876020  61876040  61876060  61876080  61876100  61876120  61876140  61876160  61876180  61876200  61876220  61876240  61876260
chr7:61875975-61876271 tcaccataagcctcaatgcactcccaaatatccctttgcagattctacattaacagtttttcaaaactgctgaatgaaaagaaaggtttaactctgtgagagtaatgctcacaacacaatgcgttttctcagaaagcttccttctggtttttattctaggatatttcctttgccattggcctgagagagagatatatgcatgtcacacaaagtggtttctcagagagcttccctctagtttttatcctgggatattcactttttcaccattggcctcaatgagctcccaggaatat

ST-E00144:68:H2LCKCCXX:6:1217:14306:20049+ AAGCCTCAATGCACTCCCAAATATCCCTTtGCaGaTTcTACATTAACAGTTtTTCAAAACTGCTGAATGAAAAGAAAGgTTTAACTCTGTGAGAGTAATGCTCACAACACAATGcGttTTCTcAgAAAGCTTCCTtCTGGtTTTaTTCT
ST-E00144:68:H2LCKCCXX:8:1205:8501:12068 + CTCAATGCACTCCCAAATAgCCCTTTGCAGATTCTACATTAACAGTTTTtCAAAaCTGCTGAATGAAAAGAAAGGTTTAACTCTGTGAGAGTAATGCgCACAACACaATtcgtTTTCTCAGAAAGCTTCCTtcTGGTTTTTATTCTAtG
ST-E00144:68:H2LCKCCXX:1:1217:17666:12332+ CAATGCACTCCCAAATATCCCTTTGCAGATTCTACATTAACAGTTTTTCAAAACTGCTGAATGAAAAGAAAGGTTTAACTCTGTGAGAGTAATGCTCACAACACAATGCGTTTTCTCAGAAAGCTTCCTTCTGGTTTTTATTCTAGGATA
ST-E00144:68:H2LCKCCXX:1:1105:19117:56476+ ACTCCCAAATATCCCTTTGCAGATTCTACATTAACAGTTTTTCAAAACTGCTGAATGAAAAGAAAGGTTTAACTCTGTGAGAGTAATGCTCACAACACAATGCGTTTTCTCAGAAAGCTTCCTTCTGGTTTTTATTCTAGGATATTTCCT
ST-E00144:68:H2LCKCCXX:1:2103:2492:61082 + AAATATCCCTTTGCAGATTCTACATTAACAGTTTTTCAAAACTGCTGAATGAAAAGAAAGGTTTAACTCTGTGAGAGTAAgGCTCACAACACAAtGCGTtTTCTCAGAAAGCtTcCTTCTGGTtttTaTtcTaGGaTaTtTccTTTTgC
ST-E00144:68:H2LCKCCXX:4:1223:11363:8200 + TTTGCAGATTCTACATTAACAGTTTTTCAAAACTGCTGAATGAAAAGAAAGGTTTAACTCTGTGAGAGTAATGCTCACAACACAATGCGTTTTCTCAGAAAGCTTCCTTCTGGTTTTTATTCTAGGATATTTCCTTTTGCCATTGGCCTG
ST-E00144:68:H2LCKCCXX:1:1102:9871:24497 + TTGCAGATTCTACATTAACAGTTTTTCAAAACTGCTGAATGAAAAGAAAGGTTTAACTCTGTGAGAGTAATGCTCACAACACAATGCGTTTTCTCAGAAAGCTTCCTTCTGGTTTTTATTCTAGGATATTTCCTTTTGCCATTGGCCTG
ST-E00144:68:H2LCKCCXX:1:2114:12083:38069+ TTGcAGATtCTACATTAACAGTTTTTCAAAACTGCTGAATGAAAAGAAAGGTTTAACTCTGTGAGAGTAATGCTCACAACACAATGCGTTTTCTCAGAAAGCTTCCTTCTGGTTTTTATTCTAGGATATTTCCTTTTGCCATTGGCCTgA
ST-E00144:68:H2LCKCCXX:8:2121:19716:11734+ ATTCTACATTAACAGTTTTTCAAAACTGCTGCATGaccAGaAAGGTTTAcCTCTGTGAgAGTAATgcTCACAACACAATGCGTTTTCTCaGAAAGCTTCCTTCTGGTTTTTATTCTAgGATATTTCCTTTTAccATTGgCcTGaGaGAGA
ST-E00144:68:H2LCKCCXX:1:1114:8764:44978 + TCTACATTAACAGTTTTTCAAAACTGCTGAATGAAAAGAAAGGTTTAACTCTGTGAGAGTAATGCTCACAACACAATGCGTTTTCTCAGAAAGCTTCCTTCTGGTTTTTATTCTAGGATATTTCCTTTTGCCATTGGCCTGAGAGAGAGA
ST-E00144:68:H2LCKCCXX:1:1201:27075:38509+ TACATTAACAGTTTTTCAAAACTGCTGAATGAAAAGAAAGGTTTAACTCTGTGAGAGTAATGCTCACAACACAATGCGTTTTCTCAGAAAGCTTCCTTCTGGTTTTTATTCTAGGATATTTCCTTTTGCCATTGGCCTGAGAGAGAGATA
ST-E00144:68:H2LCKCCXX:8:1114:8318:52889 + ACATTAACAGTTTTTCAAAACTGCTGAATGAAAAGAAAGGTTTAACTCTGTGAGAGTAATGCTCACAACACAATGCGTTTTCTCAGAAAGCTTCCTTCTGGTTTTTATTCTAGGATATTTCCTTTTGCcaTTGGCCTGAGAgAGAGATAT
ST-E00144:68:H2LCKCCXX:4:1221:25512:17834+ CAGTTTTTCAAAACTGCTGAATGAAAAGAAAGGTTTAACTCTGTGAGAGTAATGCTCACAACACAATGCGTTTTCTCAGAAAGCTTCCTTCTGGTTTTTATTCTAGGATATTTCcTTTTGCCATTGGCCTGAGAGAGAGATATATGCATG
ST-E00144:68:H2LCKCCXX:1:1219:5283:5757  + AGTTtTtCAAAACtGcTGaATGaAAAGaAAGGTtTAACTCTGTGAGaGtaATgCTCACAACACAAtGCGTTTTCtCAGaAAGCttCctTaTgGttTTTAtTCTAGGATATttcctTatgcaatcgaccTAagaGAGagatAtaTGCAtGa
ST-E00144:68:H2LCKCCXX:3:2203:29247:24761+ TTTTCAAAACTGCTGAATGAAAAGAAAGGTTTAACTCTGTGAGAGTAATGCTCACAACACAATGCGTTTTCTCAGAAAGCTTCCTTCTGGTTTTTATTCTAGGATATTTCCTTTTGCCATTGGCCTGAGAGAGAGATATATGCATGTCAC
ST-E00144:68:H2LCKCCXX:8:1224:25684:11101+ CAAAACTGcTGAATGAAAAGAAAGGTTTAACTCTGTGAGAGTAATGCTCACAACACAATGCGTTTTCTCAGAAAGCtTCCTTCTGGTTTTTATTCTAGGATATtTCCTTTTGCCATTGGCCTGAGAGAGAGATATATGCATGTCacaCAA
ST-E00144:68:H2LCKCCXX:4:2105:5283:65354 + AAAACTGCTGtATGAAAAGAAAGGTTTAACTCTGTGAGAGTAATGCTCACAACACAATGCGTTTTCTCAGAAAGCTTCCTTCTGGTTTTTATTCTAGGATATTTCCTTTTACCATTGGCCTGAGAGaGaGatATATGcATgTCACACAAa
ST-E00144:68:H2LCKCCXX:4:2105:5303:65424 + AAAACTGCTGGATGAAAAGAAAGGTTTAACTCTGTGAGAGTAATGCTCACAACACAATGCGTTTTCTCAGAAAGCTTCCTTCTGGTTTTTATTCTAGGATATTTCCTTTTACCATTGGCCTGAGAGAGAGaTATATGCAtGTCAcACAAa
ST-E00144:68:H2LCKCCXX:1:1216:5283:52731 + AaaCTGCTGAATGAAAAGAAAGGTTTAACTCTGTGAGAGTAATGCTCACAACACAATGCGTTTTCTCAGAAAGCTTCCTTCTGGtTTTTATTCTAGGATATTTCCTTTTACcATTGGCCTGAGAGAGAGATATATGCATGTcAcacAAaG
ST-E00144:68:H2LCKCCXX:7:2207:24842:40689+ AAACTGCTGAATGAAAAGAAAGGTTTAACTCTGTGAGAGTAATGCTCACAACACAATGCGTTTTCTCAGAAAGCTTCCTTCTGGTTTTTATTCTAGGATATTTCCTTTTGCCATTGGCCTGAGAGAGAGATATATGCATGTCACACAAAG
ST-E00144:68:H2LCKCCXX:6:2218:29440:30685+ GCTGAATGAAAAGAAAGGTTTAACTCTGTGAGAGTAATGCTCACAACACAATGCGTTTTCTCAGAAAGCTTCCTTCTGGTTTTTATTCTAGGATATTTCCTTTTACCATTGGCCTGAGAGAGAGATATATGCATGTCACACAAAGTGGT
ST-E00144:68:H2LCKCCXX:8:2119:4613:61627 + ATGAAAAGAAAGGTTTAACTCTGTGAGAGTAATGCTCACAACACAATGCGTTTTCTCAGAAAGCTTCCTTCTGGTTTTTATTCTAGGATATTTCCTTTTACCATTGGCCTGAGAGAGAGATATATGCATGTCACACAAAGTGGttTCTCA
ST-E00144:68:H2LCKCCXX:3:2110:3588:67129 + AAGGTTTAACTCTGTGAGAGTAATGCTCACAACACAATGCGTTTTCTCAGAAAGCTTCCTTCTGGTTTTTATTCTAGGATATTTCCTTTTGCCATtGGCCTGAGAgAGaGATAgATGCATGTCAcACAAAGTGGTTTctcaGAgAGcTTc
ST-E00144:68:H2LCKCCXX:1:2108:10470:42165+ TTTAACTCTGTGAGAGtAATGCTCACAACACAATGCGTTTTCTCAGAAAGCTTCCTTCTGGttTTTATTCTAGgaTAtTTCCTTTTACCATTGGCcTGAGaGAgAGATATATGCaTGTCACACAAAGTGGTTTCTcAGAGAGCTTCcctC
ST-E00144:68:H2LCKCCXX:1:2220:8125:60642 + ACTCTGTGAGAGTAATGCTCACAACACAATGCGTTTTCTCAGAAAGCTTCCTTCTGGTTTTTATTCTAGGATATTTCCTTTTGCCATTGGCCTGAGAGAGAGATATATGCATGTCACACAAAGTGGTTTCTCAGAGAGCTTCCCTCTAGT
ST-E00144:68:H2LCKCCXX:3:2207:9982:28558 + CTCTGTGAGAGTAATGCTCACAACACAATGCGTTTTCTCAGAAAGCTTCCTTCTGGTTTTTATTCTAGGATATTTCCTTTTGCcATTGGCCTGAGAGAGAGaTATATGCATGTCACACAAAGTGGTTTCTCAGAGAGCttCCCTCTAGTt
ST-E00144:68:H2LCKCCXX:4:2101:21848:43906+ AGagTAATGCTCACAACACAATGCGTTTTCTCaGAAAGcTTcCtTCTGGTTTTTATTCTAGGaTATTTCCTTTTgccatTGGcCTGAGAGcgaGATATATGCATGTCACACAAAGTGGtTTCTCAgaGAGCgTCcCTatagTttttATCc
ST-E00144:68:H2LCKCCXX:4:2101:21878:43923+ AGAGTAATGCTCACAACACAATGCGTTTTCTCAGAAAGCTTCCTTCTGGTTTTTATTCTAGGATATTTCCTTTTGCCATTGGCCTGAGAgaGaGaGATATATGCATGTCACACaAAGTGgtttcTCAGAGaGCTTCCCTCTAGTTTTtatCc
ST-E00144:68:H2LCKCCXX:8:2221:19858:38456+ GTAATGCTCACAACACAATGCGTTTTCTCAGAAAGCTTCCTTCTGGTTTTTATTCTAGGATATTTCCTTTTACCATTGGCCTGAGAGAGAGATATATGCATGTCACACAAAGTGGTTTCTCAGAGAGCTTCCCTCTAGTTTTTATCcTGG
ST-E00144:68:H2LCKCCXX:6:2201:25796:38052+ AAGCTTCCTTCTGGTTTTTATTCTAGGATATTTCCTTTTGCCATTGGCCTGAGAGAGAGATATATGCATGTCACACAAAGTGGTTTCTCAGAGAGCTTCCCTCTAGTTTTTATCCTGGGATATTCACTTTTTCACCATTGGCCTCAATgA
ST-E00144:68:H2LCKCCXX:6:2107:17564:27732+ TCCTTCTGGTTTTTATTCTAGGATATTTCCTTTTGCCATTGGCCTGAGAGAGAGATATATGCATGTCACACAAAGTGGTTTCTCAGAGAGCTTCCCTCTAGTTTTTATCCTGGGATATTCACTTTTTCACCATTGGCCtcaATGAGCTCC
ST-E00144:68:H2LCKCCXX:3:2216:29927:52731- ACcatAatCCTCAATgCaCTCCCAAATATCCCTTTGCAGATTCTACATTAACAGTTTTTCAAAACTGCTGAATGAAAAGAAAGGTTTAACTCTGTGAGAGTAATGCTCACAACACAATGCGTTTTCTCAGAAAGCTTCCTTCTGGTTTTT
ST-E00144:68:H2LCKCCXX:4:2221:4420:13580 - ACCATaaGCCTCAATGCACTCCCAAATaTCCCTTTGCAGATTCTACATTAACAGTTTTTCAAAACTGCTGAATGAAAAGAAAGGTTTAACTCTGTGAGAGTAATGCTCACAACACAATGCGTTTTCTCAGAAAGCTTCCTTCTGGTTTTT
ST-E00144:68:H2LCKCCXX:3:1124:8744:31723 - TAAGCCTCAATGCACTCCCAAATATCCCTTTGCAGATTCTACATTAACAGTTTTTCAAAACTGCTGAATGAAAAGAAAGGTTTAACTCTGTGAGAGTAATGCTCACAACACAATGCGTTTTCTCAGAAAGCTTCCTTCTGGTTTTTATTC
ST-E00144:68:H2LCKCCXX:3:2210:26334:47563- TAAGCCTCAATgCACTCccaAATATCcCTTTGCAGATTCTACATtAACAGTTTtTCaAaACTGCTGAATGAAAAGAAAGGTTTAActCTGTGAGAGTAATGCTCACAACAcaaTGCGTTTTCTCAGAAAGCTTCCTTCTGGTTTTTATTC
ST-E00144:68:H2LCKCCXX:6:1218:23137:56616- AAGCCTCAATGCACTCCCAAATATCCCTTTGCAGATTCTACATTAACAGTTTTTCAAAACTGCTGAATGAAAAGAAAGGTTTAACTCTGTGAGAGTAATGCTCACAACACAATGCGTTTTCTCAGAAAGCTTCCTTCTGGTTTTTATTCT
ST-E00144:68:H2LCKCCXX:8:2123:27735:33182- CCTcAacgcaCTCCCAAATAtCCCTTTGCAGATTCTACctTaAcAgTtTtTCAATACTGCTGAATGAAAAGAAAGGTTTAACTCTGTGAGAGTAATGCTCACAACACAATGCGTTTTCTCAGAAAGCTTCCTTCTGGTTTTTATTCTAGG
ST-E00144:68:H2LCKCCXX:1:2118:6359:53012 - CACTCCCAAATATCCCTTTGCAGATTCTACATTAACAGTTTTTCAAAACTGCTGAATGAAAAGAAAGGTTTAACTCTGTGAGAGTAATGCTCACAACACAATGCGTTTTCTCAGAAAGCTTCCTTCTGGTTTTTATTCTAGGATATTTCC

ST-E00144:68:H2LCKCCXX:1:2118:6044:53065 - CaCTCCCAcATATCCCTTTGCAGATTCTACATTAACaGTTTTTCAAAACTGCTGAATGAAAAGAAAGGTTTAaCTCTgTGAGAGTAATGCTCACAACACAATGCGTTTTCTCcGAAAGCTTCCTTCTGGTTTTTATTCTAGGATATTTCC
ST-E00144:68:H2LCKCCXX:4:2122:16387:31740- TCCCAAATATCCCTtTGCAGATTCTACATTAACAGTTTTTCAAAACTGCTGAATGAAAAGAAAGGTTTAACTCTGTGAGAGTAATGCTCACAACACAATGCGTTTTCTCAGAAAGCTTCCTTCTGGTTTTTATTCTAGGATATTTCCTTT
ST-E00144:68:H2LCKCCXX:4:2122:17179:45031- TCCCAAATATCCCTTTGCAGATTcTACATTAACAGTTTTTCAAAACTGCTGAATGAAAAGAAAGGTTTAaCTCTGTGAGAGTAATGCTCACAACACAATGCGTTTTCTCAGAAAGCTTCCTTCTGGTTTTTATTCTAGGATATTTCCTTT
ST-E00144:68:H2LCKCCXX:8:1105:21949:42640- AAATATCCCTTTGCAGATTCTACATTAACAGTTTTTCAAAACTGCTGAATGAAAAGAAAGGTTTAACTCTGTGAGAGTAATGCTCACAACACAATGCGTTTTCTCAGAAAGCTTCCTTCTGGTTTTTATTCTAGGATATTTCCTTTTGCC
ST-E00144:68:H2LCKCCXX:4:2208:10104:15514- CcCtTTGCAGATTCTACATTAACAGtTTTTCAAAACTGCTGAATGAAAAGAAAGGTTTAACTCTGTGAGAGTAATGCTCACAACACAATGCGTTTTCTCAGAAAGCTTCCTTCTGGTTTTTATTCTAGGATATTTCCTTTTACCATTGGC
ST-E00144:68:H2LCKCCXX:1:2108:20559:61398- cAttttCAGaTtcTaCATTACCAGTTTTtTCAaaaCTgCTGaATgAAAAGAAaGTTTAACTCTGTGAgAGTAATgCTCACAACACAATGCGTTTTCTCAGAaAGCTTCCTTCTGGTTTTTATTCTAGGATATTTCCTTTTGCCATTGGCC
ST-E00144:68:H2LCKCCXX:8:2119:26923:14512- CCTTTGCAGATTCTACATTAACAGTTTTTCAAAACTGCTGAATGAAAAGAAAGGTTTAACTCTGTGAGAGTAATGCTCACAACACAATGCGTTTTCTCAGAAAGCTTCCTTCTGGTTTTTATTCTAGGATATTTCCTTTTACCATTGGCC
ST-E00144:68:H2LCKCCXX:1:2102:8358:11681 - TGCAGATTCTACATTAACAGTTTTTCAAAACTGCTGAATGAAAAGAAAGGTTTAACTCTGTGAGAGTAATGCTCACAACACAATGCGTTTTCTCAGAAAGCTTCCTTCTGGTTTTTATTCTAGGATATTTCCTTTTACCATTGGCCTGAG
ST-E00144:68:H2LCKCCXX:8:2210:29186:37665- aTgctCtcCcaacactccctttttaAGATTcTACATTAACAGTtTtTCAAAACTGCTGAATGAAAAGAAAGGTTTAAaTCTGTGAGAGTAAcGCTCACAACACAATGCGTTTTCTCAGAAAGCTTCCTTCtGGTTTTTATTCTAGGATATT
ST-E00144:68:H2LCKCCXX:4:2117:4958:53188 - CTACATTAACAGTtTTTCAAaACTGCTGAATGAAAAGAAAGGTTTAACTCTGTGAGAGTAATGCTCACAACACAATGCGTTTTCTCAGAAAGCTtCCTTCTGGTTTTTATTCTAGGATATTTCCTTTTACCATTGGCCTGAGAGAGAGAT
ST-E00144:68:H2LCKCCXX:1:1109:7902:20559 - ACAGTTTTTCAAAACTGCTGAATGAAAAGAAAGGTTTAACTCTGTGAGAGTAATGCTCACAACACAATGCGTTTTCTCAGAAAGCTTCCTTCTGGTTTTTATTCTAGGATATTTCCTTTTGCCATTGGCCTGAGAGAGAGATATATGCAT
ST-E00144:68:H2LCKCCXX:1:1217:11373:14723- agTTTTTcaaAAcTGCTGAATGAAaAGAAAGGTTTAaCTCTGTGAgAGTAATGCTCACAACacaATGCGTTTTCTCAGAAAGCTTCCTTCTggTTTTTATTCTAGGATATTTCCTtTTGCCATTGGCCTGAGAGAGAGATATATGCATGT
ST-E00144:68:H2LCKCCXX:6:1116:32201:3049 - AAAACTGCTGAATGAAAAGAAAGGTTTAACTCTGTGAGAGTAATGCTCACAACACAATGCGTTTTCTCAGAAAGCTTCCTTCTGGTTTTTATTCTAGGATATTTCCTTTTGCCATTGGCCTGAGAGAGAGATATATGCATGTCACACAAA
ST-E00144:68:H2LCKCCXX:7:1116:5689:48371 - TGCTGAATGAAAAGAAAGGTTTAACTCTGTGAGAGTAATGCTCACAACACAATGCGTTTTCTCAGAAAGCTTCCTTCTGGTTTTTATTCTAGGATATTTCCTTTTGCCATTGGCCTGAGAGAGAGATATATGCATGTCACACAAAGtGGT
ST-E00144:68:H2LCKCCXX:7:1116:5161:48406 - TGCTGAATGAAAAGAAAGGTTTAACTCTGTGAGAGTAATGCTCACAACACAATGCGTTTTCTCAGAAAGCTTCCTTCTGGTTTTTATTCTAGGATATTTCCTTTTGCCATTGGCCTGAGAGAGAGATATATGCATGTCACACAAAGTGGT
ST-E00144:68:H2LCKCCXX:7:1116:6268:48565 - TGCTGAATGAAAAGAAAGGTTTAACTCTGTGAGAGTAATGCTCACAACACAATGCGTTTTCTCAGAAAGCTTCCTTCTGGTTTTTATTCTAGGATATTTCCTTTTGCCATTGGCCTGAGAGAGAGATATATGCATGTCACACAAAGTGGT
ST-E00144:68:H2LCKCCXX:6:2101:19615:52520- GAAtGAAAAGAAAGGTTTAACTCTGTGAGAGTAATGCTCACAACACAATGCGTTTTCTCAGAAAGCTTCCTTCTGGTTTTtATTCTAGGATATTTCCTTTTACCAttGGCCTGAGAGAGAGATATAtGCATGTCACACAAAGTGGTTTct
ST-E00144:68:H2LCKCCXX:8:2118:11454:43660- AAAGAAAGGTTTAACTCTGTGAGAGTAATGCTCACAACACAATGCGTTTTCTCAGAAAGCTTCCTTCTGGTTTTTATTCTAGGATATTTCCTTTTGCCATTGGCCTGAGAGAGAGATATATGCATGTCACACAAAGTGGTTTCTCAGAGA
ST-E00144:68:H2LCKCCXX:4:2211:11769:12771- GGTttaaCTCTGtgaGAGTAATGCTCACAACACaATGCGTTTTCTCAGAAAGCTTCCTTCTGGTTTTTATTCTAGGATATTTCCTTTTGCCATTGGCcTGaGAGAGAGAtATATGCATGTCACACAAAGTGGTTTCTCAGAGAGCTtCCC
ST-E00144:68:H2LCKCCXX:4:2211:11789:12771- GGTTTCACTCTGTGAGAGTAATGCTCACAACACAATGCGTTTTCTCAGAAAGCTTCCTTCTGGTTTTTATTCTAGGATATTTCCTTTTGCCATTGGCCTGAGAGAGAGATATATGCATGTCACACAAAGTGGTTTCTCAGAGAGCTTCCC
ST-E00144:68:H2LCKCCXX:1:1213:8572:51008 - TGTGAGAGTAATGCTCACAACACAATGCGTTTTCTCAGAAAGCTTCCTTCTGGTTTTTATTCTAGGATATTTCCTTTTGCCATTGGCCTGAGAGAGAGATATATGCATGTCACACAAAGTGGTTTCTCAGAGAGCTTCCCTCTAGTTTTT
ST-E00144:68:H2LCKCCXX:1:1220:16864:71876- AGAGTAATGCTCACAACACAATGCGTTTTCTCAGAAAGCTTCCTTCTGGTTTTTATTCTAGGATATTTCCTTTTGCCATTGGCCTGAGAGAGAGATATATGCATGTCACACAAAGTGGTTTCTCAGAGAGCTTCCCTCTAGTTTTTATCC
ST-E00144:68:H2LCKCCXX:8:2204:21330:52292- CACAATGCGTTTTCTCAgAAAGCTTCCTTCTGGTTTTTATTCTAGGATATTTCCTTTTACCATTGGCCTGAGAGAGAGATATATGCATGTCACACAAAGTGGTTTCTCAGAGAGCTTCCCTCTAGTTTTTATCCTGGGATATTCACTTTT
ST-E00144:68:H2LCKCCXX:1:2208:12134:51202- CAATGCGTTTTCTCAGAAAGCTTCCTTCTGGTTTTTATTCTAGGATATTTCCTTTTGCCATTGGCCTGAGAGAGAGATATATGCATGTCACACAAAGTGGTTTCTCAGAGAGCTTCCCTCTAGTTTTTATCCTGGGATATTCACTTTTTC
```

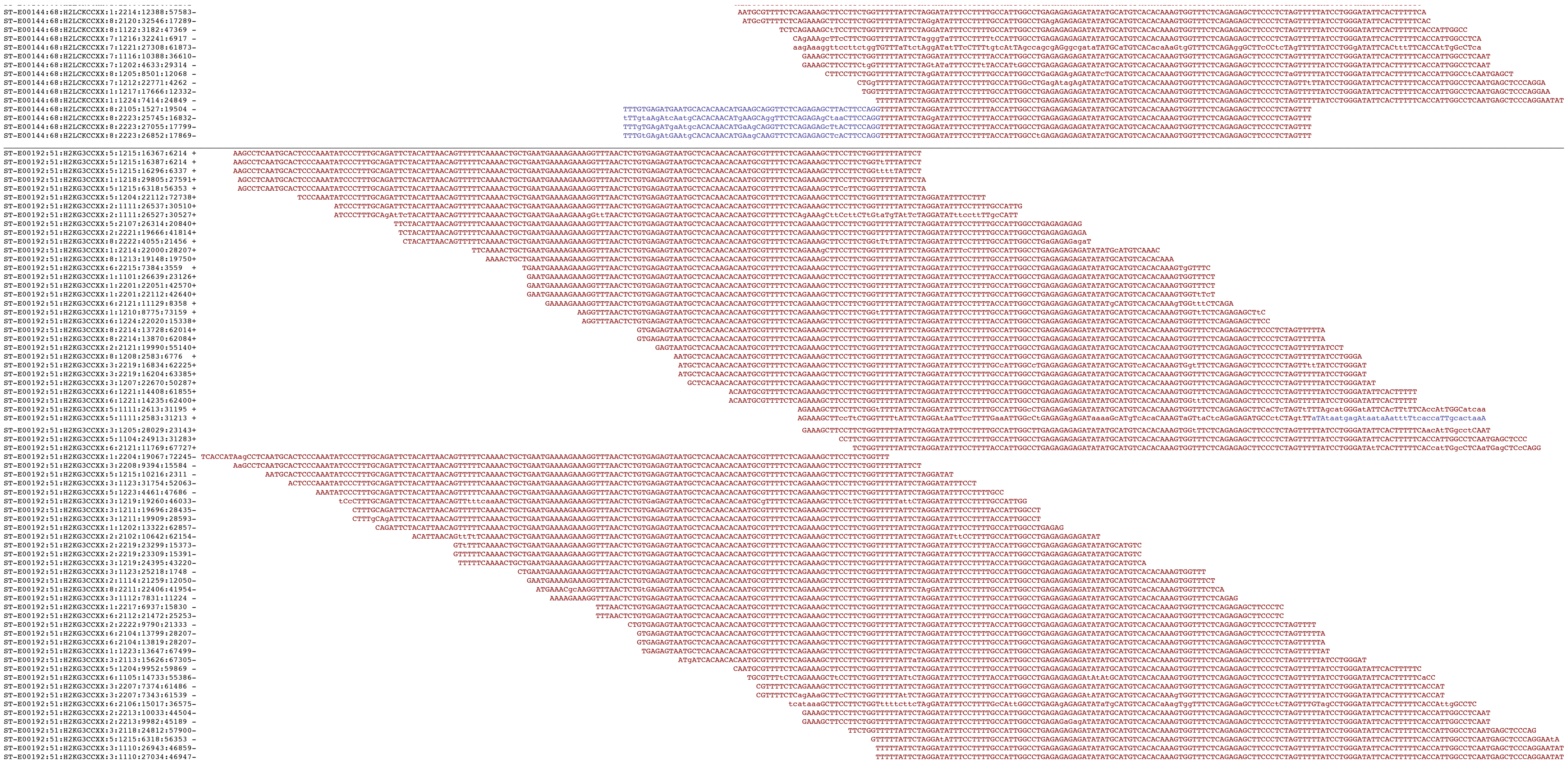

chrX 109500709 + 0 chr7 157407429 + 6 CTX 14 62 0 44 0.969333333333333 0 1 0 1 chrX 109500666 150 chr7 157407534

TTAAGAGTCCTCTATTTCAAAGGCTTTTTCTTCTATGACAAGATGGGGCCTAGTGCAGTGGGTCTGCTGTCCTTAAACAAAGGGGACCCTTAGTTACAGACAGCACCTGCAAGCCCCAGGGAGGGCCTCGGACACGGCCAGCCCTGCCAC

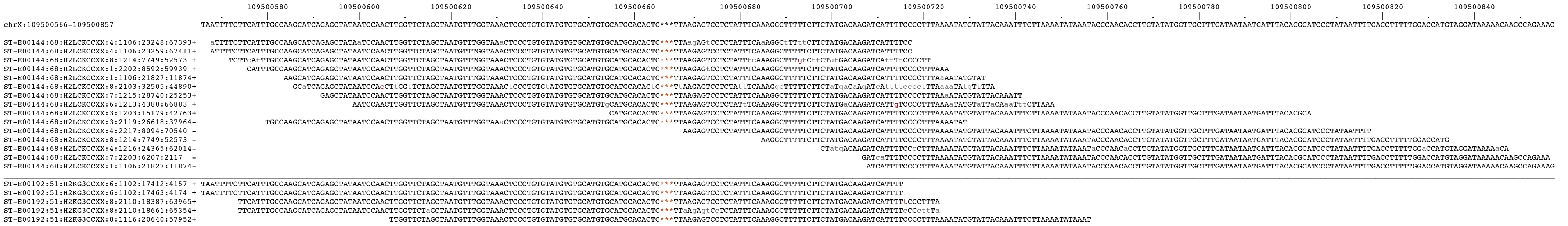

```
ST-E00192:51:H2KG3CCXX:6:2203:23979:17465+ CTAATGTTTGGTAAACTCCCTGTGTATGTGTGCATGTGCATGCACACTC***TTAAGAGTCCTCTATTTCAAAGGCTTTTTCTTCTATGACAAGATCATTTTCCCCTTTAAAATATGTATTACAAATTTCTTAAAATATAAATAcCCAACACC
ST-E00192:51:H2KG3CCXX:6:2203:24071:17518+ CTAATGTTTGGTAAACTCCCTGTGTATGTGTGCATGTGCATGCACACTC***TTAAGAGTCCTCTATTTCAAAGGCTTTTTCTTCTATGACAaGATCATTTTCCCCTTTAAAATATGTAtTACAAATTTCTTAAAATATAAATACCCaACACC
ST-E00192:51:H2KG3CCXX:2:2122:10744:25411+ TGTGTGCATGTGCATGCACACTC***TTAAGaGTCCTCTATTTCAAAGgCTTTTTCTTCTATGACAAGATCATTTTCCCCTTTAAAATATGTATTACAAATTTCTTAAAATATaAATACcCAACACCTTGTATATGGTTGCTTTGATAATAAT
ST-E00192:51:H2KG3CCXX:2:2122:10794:25499+ TGTGTGCATGTGCATGCACACTC***TTAAGAGTCCTCTATTTCAAAGGCTTTTTCTTCTATGACAAGATCATTTTCCCCTTTAAAATATGTATTACAAATTTCTTAAAATATAAATACCCAACACCTTGTATATGGTTGCTTTGATAATAAT
ST-E00192:51:H2KG3CCXX:1:1206:15169:66338+ GCACACTC***TTAAGAGTCCTCTATTTCAAAGGCTTTTTCTTCTATGACAAGATCATTTTCCCCTTTAAAATATGTATTACAAATTTCTTAAAATATAAATACCCAACACCTTGTATATGGTTGCTTTGATAATAATGATTTACACGCATCC
ST-E00192:51:H2KG3CCXX:1:1107:12885:64668+ AGGCTTTTTCTTCTATGACAAGATCATTTTCCCCTTTAAAATATGTATTACAAATTTCTTAAAATATAAATACCCAACACCTTGTATATGGTTGCTTTGATAATAATGATTTACACGCATCCCTATAATTTTGACCTTTTTGGAccATGT
ST-E00192:51:H2KG3CCXX:3:1118:11749:33551+ ATGACAAGATCATTTTCCCCTTTAAAATATGTATTACAAATTTCTTAAAATATAAATACCCAACACCTTGTATATGGTTGCTTTGATAATAATGATTTACACGCATCCCTATAATTTTGACCTTTTTGGACcATGTAGGaTAAAAACAAG
ST-E00192:51:H2KG3CCXX:3:1209:5841:70505 + ATGACAAGATCATTTTCCCCTTTAAAATATGTATTACAAATTTCTTAAAATATAAATACCCAACACCTTGTATATGGTTGCTTTGATAATAATGATTTACACGCATCCCTATAATTTTGACCTTTTTGGACCATGTAGGATAAAAACAAG
ST-E00192:51:H2KG3CCXX:1:1205:16387:8780 - ATTTTCTTCATTTGCCAAGCATCAGAGCTATAAtcCAACTTGGTTCTAGCTAATGTTtGGTAAACTCCCTGTgTaTGTGTGCATgTGCATgCACACTC***TTaAtAGTcCTCTATTTCAAAGGCTTTTTCTTCTATGACAAGATCATTTTcC
ST-E00192:51:H2KG3CCXX:3:1113:13088:35415- TTTTCTTCATTTGCCAAGCATCAGAGCTATAATCCAACTTGGTTCTAGCTAATGTTTGGTAAACTCCCTGTGTATGTGTGCATGTGCATGCACACTC***TTAAGAGTCCTCTATTTCAAAGGCTTTTTCTTCTATGACAAGATCATTTTCCC
ST-E00192:51:H2KG3CCXX:6:1213:7191:10609 - TTTTCTTCATTTGCCAAGCATCAGAGCTATAATCCAACTTGGTTCTAGCTAATGTTTGGTAAACTCCCTGTGTATGTGTGCATGTGCATGCACACTC***TTAAGAGTCCTCTATTTCAAAGGCTTTTTCTTCTATGACAAGATCATTTTCCC
ST-E00192:51:H2KG3CCXX:6:1213:7252:10785 - TTTTCTTCATtTGCCAAGCATCAGAGCTATAATCCAACTTGGTTCTAGCTAATGTTTGGTAAACTCCCTGTGTATGTGTGCATGTGCATGCACACTC***TTAAGAGTCCTCTATTTCAAAGGCTTTTTCTTCTATGACAAGATCATTTTCCC
ST-E00192:51:H2KG3CCXX:6:1213:7364:10802 - TTTTCTTCATTTGCCAAGCATCAGAGCTATAATCCAACTTGGTTCTAGCTAATGTTTGGTAAACTCCCTGTGTATGTGTGCATGTGCATGCACACTC***TTAAGAGTCCTCTATTTCAAAGGCTTTTTCTTCTATGACAAGATCATTTTCCC
ST-E00192:51:H2KG3CCXX:1:1221:28993:17887- TTCTtCATTTGCCAAGCATCAGAGCTATAATCCAACTTGGTTCTAGCTAATGTTTGgTAaaCTCCCTGtCTcTGTctGaatgTgaat***TaCtcatattcAccGTCCtaTATTTCaAaGGCTTTTTCTTCTATGACAAGATCATTTTCCCCT
ST-E00192:51:H2KG3CCXX:1:1118:16265:35713- ATTTGCCAaGCATCAGAGCTATAATCCAACTTGGTTCTAGCtAATGTTTGGTAAACTCCCTGTGTATGTGTGCATGTGCATGCACACTC***TTAAGAGTCCTCTATTTCAAAGGCTTTTTCTTCTATGACAAGATCATTTTCCCCTTTAaAA
ST-E00192:51:H2KG3CCXX:1:1118:16286:35749- ATTTGCCAAGCATCAGAGCTATAATCCAACTTGGTTCTAGCTAATGTTTGGTAAACTCCCTGTGTATGTGTGCATGTGCATGCACACTC***TTAAGAGTCCTCTATTTCAAAGGCTTTTTCTTCTATGACAAGATCATTTTCCCCTTTAAAA
ST-E00192:51:H2KG3CCXX:1:1118:16306:35749- ATTTGCCAAGCATCAGAGCTATAATCCAACTTGGTTCTAGCTAATGTTTGGTAAACTCCCTGTGTATGTGTGCATGTGCATGCACACTC***TTAAGAGTCCTCTATTTCAAAGGCTTTTTCTTCTATGACAAGATCATTTTCCCCTTTAAAA
ST-E00192:51:H2KG3CCXX:1:1118:16326:35749- ATTTGCCAAGCATCAGAGCTATAATCCAACTTGGTTCTAGCTAATGTTTGGTAAACtCCCTGTGTATGTGTGCATGTGCATGCACACTC***TTAAGAGTCCTCTATTTCAAAGGCTTTTTCTTCTATGACAAGATCATTTTCCCCTTTAAAA
ST-E00192:51:H2KG3CCXX:8:2214:20061:4209 - TTGCCAAGCATCAGAGCTATAATCCAACTTGGTTCTAGCTAATGTTTGGTAAaCTCCCTGTGTATGTGTGCATGTGCATGCACACTC***TTAAGAGTCCTCTATTTCAAAGGCTTTTTCTTCTCTGACAAGATCATTTTCCCCTTTAAAATA
ST-E00192:51:H2KG3CCXX:5:1106:17311:64844- AGAGCTATAATCCAACTTGGTTCTAGCTAATGTTTGGTAAACTCCCTGTGTATGTGTGCATGTGCATGCACACTC***TTAAGAGTCCTCTATTTCAAAGGCTTTTTCTTCTATGACAAGATCATTTTCCCCTTTAAAATATGTATTACAAAT
ST-E00192:51:H2KG3CCXX:5:1204:20366:68378- ACTTGGTTCTAGCTAATGTTTGGTAAACTCCCTGTGTATGTGTGCATGTGCATGCACACTC***TTAAGAGTCCTCTATTTCAAAGGCTTTTTCTTCTATGACAAGATCATTTTCCCCTTTAAAATATGTATTACAAATTTCTTAAAATATAA
ST-E00192:51:H2KG3CCXX:1:2104:19138:1977 - GCATGCACACTC***TTAAGAGTCCTCTATTTCAAaGGCTTTTTCTTCTATGACAAGATCATTTTCCCCTTTAAAATATGTATTACAAATTTCTTAAAATATAAATACCCAACACCTTGTATATGGTTGCTTTGATAATAATGATTTACACGC
ST-E00192:51:H2KG3CCXX:1:1104:8298:5528 - ACTC***TTAAGAGTCCTCTATTTCAAAGGCTTTTTCTTCTATGACAAGATCATTTTCCCCTTTAAAATATGTATTACAAATTTCTTAAAATATAAATACCCAACACCTTGTATATGGTTGCTTTGATAATAATGATTTACACGCATCCCTAT
ST-E00192:51:H2KG3CCXX:1:1204:30333:28435- ACTC***TTAAGAGTCCTCTATTTCAAAGGCTTTTTCTTCTATGACAAGATCATTTTCCCCTTTAAAATATGTATTACAAATTTCTTAAAATATAAATACCCAACACCTTGTATATGGTTGCTTTGATAATAATGATTTACACGCATCCCTAT
ST-E00192:51:H2KG3CCXX:1:1212:17189:68712- AAaGGCTTTTTCTTCTATGACAAGATCATTTTCCCCTTTAAAATATGTATTACAAATTTCTTAAAATATAAATACCCAACACCTTGTATATGGTTGCTTTGATAATAATGATTTACACGCATCCCTATAATTTTGACCTTTTTGGACCAT
ST-E00192:51:H2KG3CCXX:6:1114:26811:38720- AGGCTTTTTCTTCTATGACAAGATCATTTTCCCCTTTAAAATATGTATTACAAATTTCTTAAAATATAAATACCCAACACCTTGTATATGGTTGCTTTGATAATAATGATTTACACGCATCCCTATAATTTTGACCTTTTTGGACCATGT
```

```
157407280 . 157407300 . 157407320 . 157407340 . 157407360 . 157407380 . 157407400 . 157407420 . 157407440 . 157407460 . 157407480 . 157407500 . 157407520 . 157407540 . 157407560 .
chr7:157407280-157407578 TGGGAGTGTGTGATAGCAATGGCgctgtgggctgagctgtgccctgatgcccctgtgtgacgaccccaaccccccaggactgcggagtgtggccatgtttggagacagggcctctagagaggccagtaagtgaaggtgaggtcctgagggggggcctagtgcagtgggtctgctgtccttaaacaaaggggacccttagttacagacagcacctgcaagccccagggagggcctcggacacggccagccctgccacatcctggaccgcagcctccggcctccaggactatggggaaatgc

ST-E00144:68:H2LCKCCXX:3:2115:21553:19874+ AGtGTGTGATAGCAATGGCGCTGTGGGCTgAGCTGTGCCCTgATGCCCCTGTGTGACGACCCCAACCCCCaGGACTGCGGAGcGtGGCCatGtTTGGAGACAGGGCctcTAGAGaGGCCaGtcaGgGaAGGtgAGGTCCtGggGGgGGGC
ST-E00144:68:H2LCKCCXX:1:1204:12165:3506 + ATAGCAATGGCGCTGTGGGCTGAGCTGTGCCCTGATGCCCCTGTGTGACGACCCCAACCCCCAGGACTGCGGAGTGTGGCCATGTTTGGAGACAGGGCCTCTAGAGAGGCCAGTAAGTGAAGGTGAGGTCCTGAGGGGGGGCCTAGTGCA
ST-E00144:68:H2LCKCCXX:7:2123:13423:45875+ AATGGCGCTGTGGGCTGAGCTGTGCCCTGATGCCCCTGTGTGACGACCCCAACCCCCAGGACTGCGGAGTGTGGCCATGTTTGGAGACAGGGCCTcTAGAGAGGCCAGTAAGTGAAGGTGAGGtCCTGAGGGGGGGCCtAGtGCAGTGGG
ST-E00144:68:H2LCKCCXX:7:1210:29217:71085+ GCTGTGGGCTGAGCTGTGCCCTGATGCCCCTGTGTGACGACCCCAACCCCCAGGACTGCGGaGtGTGGCCATGTTTGGAGACAGGGCCtCtAGAGaGGCCAGTAAGtGAAGGgGagGccCtGAGGGGGGGCCtaGTGcagtGGgtctgct
ST-E00144:68:H2LCKCCXX:8:1102:9028:20049 + CTGTGGGCTGAGCTGTGCCCTGATGCCCCTGTGTGACGACCCCAACCCCCAGGACTGCGGAGTGTGGCCATGTTTGGAGACAGGGCCtCTAGAGAGGCCAGTAAGTGAAGGTGAGGTCCtGAGGGGGgGgcTAGTGCAGtgGGtCTGCtg
ST-E00144:68:H2LCKCCXX:8:1102:9049:20084 + CTGTGGGCTGAGCTGTGCCCTGATGCCCCTGTGTGACGACCCCAACCCCCAGGACTGCGGAGTGTGGCCATGTTTGGAGACAGGGCCtCTAGAGAGGCCAGTAAGTGAAGGTGAGGTCCtGAGGGGGGGcCtAGtgCAGTGGGTcTGCTG
ST-E00144:68:H2LCKCCXX:1:1203:10703:5757 + GGGCTGAGCTGTGCCCTGATGCCCCTGTGTGACGACCCCAACCCCCAGGACTGCGGAGTGTGGCCATGTTTGGAGACAGGGCCTCTAGAGAGGCCAGTAAGTGAAGGTGAGGtCCTGAGGGGGGGCctAGTGCAGTGGGTCTGCTGTCCT
ST-E00144:68:H2LCKCCXX:4:2110:8572:30545 + GAGCTGTGCCCTGATGCCCCTGTGTGACGACCCCAACCCCCAGGACTGCGGAGTGTGGCCATGTTTGGAGACAGGGCCTCTAGAGAGGCCAGTAAGTGAAGGTGAGGTCCTGAGGGGGGGCCTAGTGCAGTGGGTCTGCTGTCCTTAAAC
ST-E00144:68:H2LCKCCXX:6:1121:30323:35766+ GATGCCCCTGTGTGACGACCCCAACCCCCAGGACTGCGGAGTGTGGCCATGTTTGGAGACAGGGCCTCTAGAGAGGCCAGtaAGcGAAGGTGAGGTCCTGAGGGGGGGCCtaGTGCaGTGGGTCtGCTGTCCTTaAACAAAGGGGACCCt
ST-E00144:68:H2LCKCCXX:6:1203:30485:22300+ TGTGACGACCCCAACCCCCAGGACTGCGGAGTGTGGCCATGTTTGGAGACAGGGCCTCTAGAGAGGCCAGTAAGTGAAGGTGAGGTCCTGAGGGGGGGCCTAGTGCAGTGGGTCTGCTGTCCTTAAACAAAGGGGACCCTTAGTTACaGA
ST-E00144:68:H2LCKCCXX:3:2203:22558:58076+ CGACCCCAACCCCCAGGACTGCGGAGTGTGGCCATGTTTGGAGACAGGGCCTCTAGAGAGGCCAGTAAGTGAAGGTGAGGTCCTGAGGGGGGGCCTAGTGCAGTGGGTCTGCTGTCCTTAAACAAAGGGGACCCTTAGTTACAGACAGCA
ST-E00144:68:H2LCKCCXX:7:2115:23076:62875+ CGACCCCAACCCCCAGGACTGCGGAGTGTGGCCATGTTTGGAGACAGGGCCTCTAGAGAGGCCAGTAAGtGAAGGtGAGGTCCTGaGGgGGGGCCTAttgaAGTGGGTCtGCTGTCCTGaaacaAaGGGGaCCCTTgGTTaCAGgCAGca
ST-E00144:68:H2LCKCCXX:1:2111:15981:68342+ CCCCAACcCCCaggACTGCGGAGTGTgGCCATGTTTGGAGaCAGgGCctCtAgAgaGGCCaGTAAGcGAAGgTGAggTcCTGAGGGGGGGgCCTAGTGCaGTGGGTCTGCagtCCtTAaACAAAGGGGacCCTtaGTTaCAGAcaGCAcct
ST-E00144:68:H2LCKCCXX:7:1207:18813:54085+ CCAGGACTGCggAGtGtGgCcatgtttggaGaCAGGGcgtctAGcGAgGCCaGgacGtGAAGGtGaGGtcCtGagGGtgggcctaGTGCAgTGGgtttGcTcTCCTtaaacaaAGGgGaCgcgtgGtTgcatgcagcacCtgggggCcc
ST-E00144:68:H2LCKCCXX:6:1113:28323:25060+ GGACTGCGGAGTGTGGCCATGTTTGGAGACAGGGCCTCTAGAGAGGCCAGTAAGTGAAGGTGAGGTCCTGAGGGGGGGcCtaGtGCAgggGGtccgctGgcCttAAAccAAGGGGaccCttaggtaCaGacaggacctgcaagcCCCcGG
ST-E00144:68:H2LCKCCXX:8:1210:23827:55597+ GGACTGCGGAGTGTGGCCATGTTTGGAGACAGGGCCTCtAGaGAGGCCAGTAAGTGAAGGTGAGGTCCTGAGGGGGGGCCTAGTGCAGTGGGTCTGCTGTCCTTAAaCAAAGGGGACCCTTAGTTACAGACAGCACCTGCaAGCCCCAGG
ST-E00144:68:H2LCKCCXX:8:1210:23827:56230+ GGACTGCGGAGTGTGGCCATGTTTGGAGACAGGGCCTCTAGAGAGGCCAGTAAGTGAAGGTGAGGTCCTGAGGGGGGGCCTAGTGCAGTGGGTCTGCTGTCCTTAAACAAAGGGGACCCTTAGTTACAGACAGCACCtGCAAGCCCCAGG
ST-E00144:68:H2LCKCCXX:6:1113:1690:28611 + GGaGTGtggCCATGTTtGGAGACAGGGCCTCTAGAGAGGCCAGTAAGtGAaGGTGAGGtCCTGagGGgGGGCCtAGTGCaGTGGGTCTGCTGccCTtAAAcAAAGgGGACCcttaGttaCaGACAGCaCccGcAAGcCccaGGGAGGGCC
ST-E00144:68:H2LCKCCXX:6:2218:13433:59148+ GGAGTGTGGCCATGTTTGGAGACAGGGCCTCTAGAGAGGCCAGTAAGTGAAGGTGAGGTCCTGAGGGGGGGCCTAGTGCAGTGGGTCTGCTGTCCTTAAACAAAGGGGAcCCTTaGTTACAGACaGcACCTGCAAGCCCCAGGGAGgGCC
ST-E00144:68:H2LCKCCXX:6:2115:32678:53135+ GGCCTCTAGAGAGGCCAGTAAGTGAAGGTGAGGTCCTGAGGGGGGGCCTAGTGCAGTGGGTCTGCTGTCCTTAAACAAAGGGGACCCttAGTTACAGACAGCACCTGCAAGCCCCaGGGAGGGCCtCGGACACGGCCAGCCCTGCcACAT
ST-E00144:68:H2LCKCCXX:1:2122:9769:67288 + CCTCTAGAGAGGCCAGTAAGTGAAGGTGAGGTCCTGAGGGGGGGCCTAGTGCAGTGGGTCTGCTGTCCTTAAACAGAGGGGACCCTTAGTTACAGACAGCACCTGCAAGCCCCAGGGAGGGCCTCGGACACGGCCAGCCCTGCCACATCC
ST-E00144:68:H2LCKCCXX:1:2221:11647:72896+ TCTAGAGAGGCCAGTAAGTGAAGGTGAGGTCCTGAGGGGGGGCCTAGTGCAGTGGGTCTGCTGTCCTTAAACAAAGGGGACCCTTAGTTACAGACAGCACCTGCaaGCCCCAGGGAGGGCCTCGGACaCGGccaGCcCtgCcACAtCcTG
ST-E00144:68:H2LCKCCXX:4:1120:26334:32092+ AGAGAGGCCAGTAAGTGAAGGTGAGGTCCTGAGGGGGGGCCTAGTGCAGTGGGTCTGCTGTCCTTAAACAAAGGGGACCCTTAGTTACAGACAGCACCTGCAAGCCCCAGGGAGGGCCTCGGACACGGCCAGCCCTGCCACATCCTGGAC
ST-E00144:68:H2LCKCCXX:1:2216:24842:62488+ AGAGGCCAGTAAGTGAAGGTGAGGTCCTGAGGGGGGGCCTAGTGCAGTGGGTCTGCTGTCCTTAAACAAAGGGGACCCTTAGTTACAGACAGCACCTGCAAGCCCCAGGGAGGGCCTCGGACACGGCCAGCCCTGCCACATCCgGGaCCG
ST-E00144:68:H2LCKCCXX:6:1124:14286:66918+ AGAGGCCAGTAAGTGAAGGTGAGGTCCTGAGGGGGGGCCTAGTGCAGTGGGTCTGCTGTCCTTAAACAAAGGGGACCCTTAGTTACAGACAGCACCTGCAAGCCCCagGGaGGGCCTCGGACACGGCCAGCCCTGCCACcTCCTGGACCG
ST-E00144:68:H2LCKCCXX:8:1215:30902:3823 + CTGAGGGGGGGCCTAGTGCcGTGGGTcTGCTGTCCTTAAACAAAGGGGACCCTTAGTtACAGACAGCACCTGCAAGCCCCAgGGaGGGCCtCGGACACGGCcagCCctgCcACatCCggGccCgccgCctccggcCgccagggctaaggg
ST-E00144:68:H2LCKCCXX:8:1215:30922:4034 + CTGAGGGGGGGCCTAGTGCAGTGGGTCTGCTGtCCTTAAACAAaGGGGACCCTTagtTaCAGACAGCACCTGCAAgCCCCAgGGagGGCCTCGGACACGGcCagCCCtGCcacccccCtggacCgCagCcccgggCcCCaaGGcctacgcg
ST-E00144:68:H2LCKCCXX:1:1104:13230:13298+ GGGGGGGCCTAGTgCAGTGGGTCTGCTGTCCTTAAACAAAGGGGaCCCTTAGTTACAGACAGCACCTGCAAGCCCCaGGgAGGGCCTCGGACACGGCCAGCCCcgCCaCaTCCtGgACCgCAGcCTCcgGCCtCCaGGACtaTgGGGcaA
ST-E00144:68:H2LCKCCXX:6:1219:29968:62084- tGGGggtgtgtGagAGcaaTgGCGCTGTGGGCtGAGCtGTGCCCTGATGCCCCTGTGTGACGACCCCAACCCCcAGGACTGCGGAGTGTGGCCATGTTTGGAGACAGGGCCTCTAGAGAGGCCAGTAAGTGAAGGTGAGGTCCTGAGGGG
ST-E00144:68:H2LCKCCXX:7:1219:20437:21667- GTGTGTGAGAGCAATGGCGCTGTGGGCTGAGCTGTGCCCTGATGCCCCTGTGTGACGACCCCAACCCCCAGGACTGCGGAGTGTGGCCATGTTTGGAGACAGGGCCTCTAGAGAGGCCAGTAAGTGAAGGTGAGGTCCTGAGGGGGGGCC
ST-E00144:68:H2LCKCCXX:1:2120:22700:72105- gtgTGaTAGCAaTGGcGCgGTGGGCTGaGCTGTGCCCTGATGCCCCTGTGTGACGACCCCAACCCCCAGGACTGCGGAGTGTGGCCATGTTTGGAGACAGGGCCTCTAGAGAGGCCAGTAAGTGAAGGTGAGGTCCTGAGGGGGGGCCTA
ST-E00144:68:H2LCKCCXX:1:2118:29369:13615- TGATAGcaatGGCGCTGTGGGCTGAGCTGTGCCcTGAtGCCCCTGTGTGACGACCCCAACCCCCAGGACTGCGGAGTGTGGCCATGTTTGGAGACAGGGCCTCTAGAGAGGCCAGTAAGTGAAGGTGAGGTCCTGAGGGGGGGCCTAGTG
ST-E00144:68:H2LCKCCXX:8:1214:3984:12226 - gAgAGCaagGGCGCtGTGGGCtGAGCTGTGCCCTGATGCCCCTGtGTGACGACCCCAACCCCCAGGACTGCGGAGTGTGGCCATGTTTGGAGACAGGGCCTCTAGAGAGGCCAGTAAGTGAAGGTGAGGTCCTGAGGGGGGGCCTAGTGC
ST-E00144:68:H2LCKCCXX:8:2207:18143:65477- aGcAAtGGCgctGgGGGCTGAGCTGTGCCCtGAtGCCCCTGTGTGACGACCCCaaCCCCCAGGACTGCGGAGTGTGGCCATGTTTGGAGACAGGGCCTCTAGAGAGGCCAGTAAGTGAAGGTGAGGTCCTGAGGGGGGGCCTAGTGCAGT
ST-E00144:68:H2LCKCCXX:1:1217:19848:51659- GCAATGGCGCtGTGGGCTGAGCTGTGCCCTGATGCCCCTGTGTGACGACCCCAACCCCCAGGACgGCGGAGtGgGGCCAtGttTGGAGACAGGGCCTCTAGAGAGGCCAGTAAGtGAAGGTGAGGTCCTGAGGGGGGGCCTAGTGCAGTG
ST-E00144:68:H2LCKCCXX:1:1105:10368:32320- AATGGCGCTGTGGGCTGAGCTGTGCCCTGATGCCCCTGTGTGACGACCCCAACCCCCAGGACTGCGGAGTGTGGCCATGTTTGGAGACAGGGCCTCTAGAGAGGCCAGTAAGTGAAGGTGAGGtCCTGAGGGGGGGCCTAGTGCAGTGGG
ST-E00144:68:H2LCKCCXX:7:1218:8795:44785 - tGGCgCTGTGGGCTGAGCtGTGCCCTGATGCCCCTGTGTGACGaCCCCAACCCCCAGGACTGCGGAGTGTGGCCATGTTTGGAGACAGGGCCTCTAGAGAGGCCAGTAAGTGAAGGTGAGGTCCTGAGGGGGGGCCTAGTGCAGTGGGTC
ST-E00144:68:H2LCKCCXX:4:2215:4126:43449 - GGCGcgggGGGcTGaGCTGTGCCCTgatGCCCCTGTGTGACGACCCCAACCCCCAGGACTGCGGAGTGTGGCCATGTTTGGAGACAGGGCCTCTAGAGAGGCCAGTAAGTGAAGGTGAGGTCCTGAGGGGGGGCCTAGTGCAGTGGGTCT

ST-E00144:68:H2LCKCCXX:4:2119:7445:69872 - CTgTGGGCTGAGCTGTGCCCTGATGCCCCTGTGTGACGACCCCAACCCCCAGGACTGCGGAGTGTGGCCATGTTTGGAGACAGGGCCTCTAGAGAGGCCAGTAAGTGAAGGTGAGGTCCTGAGGGGGGGCCTAGTGCAGTGGGTCTGCTG
ST-E00144:68:H2LCKCCXX:1:1110:5902:41040 - AGCTGTGCCCTgATGCccCTGTGTGACGACCCCAaCCCCCAGGACTGCGGAGTGTGGCCATGTTTGGAGACAGGGCCTCTAGAGAGGCCAGTAAGTGAAGGTGAGGTCCTGAGGGGGGGCCTAGTGCAGTGGGTCTGCTGTCCTTAAACA
ST-E00144:68:H2LCKCCXX:1:1110:5912:41058 - AGCTGTGCCCTGATGCCCCTGTGTGACGACCCCAACCCCCAGGACTGCGGAGTGTGGCCATGTTTGGAGACAGGGCCTCTAGAGAGGCCAGTAAGTGAAGGTGAGGTCCTGAGGGGGGGCCTAGTGCAGTGGGTCTGCTGTCCTTAAACA
ST-E00144:68:H2LCKCCXX:6:2222:31257:6899 - TGTGCCCTGATgCCCCTGTGTGACGaCCCCAACCCCCaGGACTGCGGAGTGTGGCCATGTTTGGAGACAGGGCCTCTAGAGAGGCCAGTAAGTGAAGGTGAGGTCCTGAGGGGGGGCCTAGTGCAGTGGGTCTGCTGTCCTTAAACAAAG
ST-E00144:68:H2LCKCCXX:8:1107:5293:23812 - gCCCTGatgCCCCTGTGtGACGcccCCAACCCCCAGGACTGCGGAGTGTGGCCATGTTTGGAGACAGGGCCTCTAGAGAGGCCAGTAAGTGAAGGTGAGGTCCTGAGGGGGGGCCTAGTGCAGTGGGTCTGCTGTCCTTAAACAAAGGGG
ST-E00144:68:H2LCKCCXX:8:2220:8094:69802 - CCCTGTgTgACGaCcccaactCcaAgGACtGCGGaGTgtGGCCATGTTTGGAGACAGGGCCTCTAGAGAgGCCAGTAaGTGAAGGTGAGGTCCTGAGGGGGGGCCTAGTGCAGTGGGTCTGCTGTCCTTAAACAAAGGGGACcCTTAGTT
ST-E00144:68:H2LCKCCXX:1:1111:31835:58779- GtgtgACGaCCCcaaCCCcCAGgaCTGCGGAGTGTGGCCATGTTTGGAGACAGGGCCTCTAGAGAGGCCAGTAAGTGAAGGTGAGGTCCTGAGGGGGGGCCTAGTGCAGTGGGTCTGCTGTCCTTAAACAAAGGGGACCCTTAGTTACAG
ST-E00144:68:H2LCKCCXX:6:2204:5486:60080 - GTgtGacgaCcCCAaCcCCCAGGaCTGCgGAGTGTGGCCaTGTTTGGAGACAGGGCCTCTAGAGAGGCCAGTAAGTGAAgGTGAGGTCCTGAGGGGGGGCCTAGTGCAGTGGGTCTGCTGTCCTTAAACAAAGGGGACCCTTAGTTACAG
ST-E00144:68:H2LCKCCXX:8:2124:11830:21772- GaCCCCAACCCCCAGGACTGCGGAGTGTGGCCATGTTTGGAGACAGGGCCTCTAGAGAGGCCAGTAAGTGAAGGTGAGGTCCTGAGGGGGGGCCTAGTGCAGTGGGTCTGCTGTCCTTAAACAAAGGGGACCCTTAGTTACAGACAGCAC
ST-E00144:68:H2LCKCCXX:8:1211:18123:53663- AACccCcAGGactGCGGAGTGTGGCCAtGTTTGGAGACAGGGCCTCTAGAGAGGCCAGTAAGTGaagGtGAGGTCCTGAGGGGGGGCCTAGTgCAGTGGGTCTGCTGtCCTtAAaCaAAGGGGACCCTTAGTTACAGACAGCACCTGCAA
ST-E00144:68:H2LCKCCXX:8:1202:18417:9132 - CCCCAGgACtGCGgaGtGtGGCcaTGTTTGGAGACAGGGCCgCTAGAGAGGCCAGTAAGTGAaGGTGAGGTCCTGAGGGGGGGCCTAGTGCAGTGGGTCTGCTGTCCTTAAACAAAGGGGACCCTTAGTTACAGACAGCACCTGCAAGCC
ST-E00144:68:H2LCKCCXX:8:2210:12205:46156- AGGACTGCGGAGTGTGGCCATGTTTGGAGACAGGGCCTCTAGAGAGGCCAGTAAGTGAAGGTGAGGtCCTGAGGGGGGGCCTAGTGCAGTGGGTCTGCTGTCCTTAAACAAAGGGGACCCTTAGTTACAGACAGCACCTGCAAGCCCCAG
ST-E00144:68:H2LCKCCXX:3:2217:25928:67358- GGAGTGTGGCCATGTTtGGAGACAGGGCCTCTAGAGAGGCCAGTAAGTGAAGGTGAGGTCCTGAGGGGGGGCCTAGTGCAGTGGGTCTGCTGTCCTTAAACAAAGGGGACCCTTAGTTACAGACAGCACCTGCAAGCCCCAGGGAGGGCC
ST-E00144:68:H2LCKCCXX:8:1116:26943:69854- GTTTGGAGACAGGGCCTCTAGAGAGGCCAGTAAGTGAAGGTGAGGtCCTGAGGGGGGGCCTAGTGCAGTGGGTCTGCTGTCCTTAAACAAAGGGGACCCTTAGTTACAGACAGCACCTGCAAGCCCCAGGGAGGGCCTCGGACACGGCC
ST-E00144:68:H2LCKCCXX:6:2213:22944:31494- cagGGcCTCTAGAGAGGCCAGTAAGTGaAGGtGAGGTCCTGAGGGGGGGCCTAGTGCAGTGGGTCTGCTGTCCTTAAACAAAGGGGACCCTTAGTTACAGACAGCACCTGCAAGCCCCAGGGAGGGCCTCGGACACGGCCAGCCCTGCCA
ST-E00144:68:H2LCKCCXX:7:2124:12307:5159 - CaGGGCCTCTAGAGAGGCCAGTAAGTGAAGGTGAGGTCCTGAGGGGGGGCCTAGTGCAGTGGGTCTGCTGTCCTTAAACAAAGGGGACCCTTAGTTACAGACAGCACCTGCAAGCCCCAGGGAGGGCCTCGGACACGGCCAGCCCTGCCA
ST-E00144:68:H2LCKCCXX:1:2204:21635:41497- AgaGCCTCTAGAGAGGcCAGTAAGTGAAGGTGAGGtCcTGAGGGGGGGCCTAGTGCAGTGGGTCTGCTGTCCTTaAACAAAGGgGACCCTTAGtTACAGACAGCACCTGCAAGCCCCAGGGAGGGCCGCGGACACGGCCAGCCCTGCCAC
ST-E00144:68:H2LCKCCXX:8:1218:31531:40917- GAGAGGCCAGtAAGTGAAGGTGaGgtCCTGaGGGGGGGCCTAGTGCAGTGGGTCTGCtGTCCTTAAACAAAGGGGACCCTTAGTTACAGACAGCACCTGCAAGCCCCAGGGAGGGCCTCGGACACGGCCAGCCCTGCCACATCCTGGACC
ST-E00144:68:H2LCKCCXX:7:2124:29075:66444- gGccaGTAAGTGAAGGTGAGGtcCtGAGGGGGGGCCTAGTGCAGTGGGTCtGCTGTCCTTAAACAAAGGGGACCCTTAGTTACAGACAGCACCTGCAAGCCCCAGGGAGGGCCTCGGACACGGCCAGCCCTGCCACATCCTGGACCGCAG
ST-E00144:68:H2LCKCCXX:3:2203:22558:58076- CAGTAAGtGAAGGtGaGGTCCTGAGGGGGGGCCTAGTGCAGTGGGTCTGCTGTCCTTAAACAAAGGGGACCCTTAGTTACAGACAGCACCTGCAAGCCCCAGGGAGGGCCTCGGACACGGCCAGCCCTGCCACATCCTGGACCGCAGCCT
ST-E00144:68:H2LCKCCXX:1:2214:14499:66795- AGtAAGTGAAGGTGAgGTCCTGAGGGGGGGCCTAGTGCAGTGGGTCTGCTGTCCTTAAACAAAGGGGACCCTTAGTTACAGaCAGCACCTGCAAGCCCCAGGGAGGGCCTCGGACACGGCCAGCCCTGCCACATCCTGGACCGCAGCCTC
ST-E00144:68:H2LCKCCXX:6:2205:16864:12314- TGAAGGTGAgGTcCTGAGgGgGGGCCTAGTGCaGTGggTCTGCTGTCCtTAaACaAaGGggcCCCTTAGTTaCAGACAGCACCTgCAaGcCCCAGGGAGGgCCtCGGACacggCCaGCCCTGCCACATCCTGGACCGCAGCCtCCGGcCT
ST-E00144:68:H2LCKCCXX:6:1211:2715:19381 - TgAggGAagggGAGGtccTgagggGgGGCCTagTGCAGTGGgTCTGCtGTCCTTAAACAaAGGgGAcCCTtAGTTACgGACAGCaCCTGcaAGgCCCaGggaGGGCCTCGGACaCGGcCAgccCTGccACATCCTGGAcCGCAGCCTCCG
ST-E00144:68:H2LCKCCXX:4:1221:5760:59781 - CCtGAGGgGGGgCcTAGTGCAGTgcGTCTGCTGTCCTTAAACAAaGGGGACCCTTAGTTACAGACAGCACCTGCAAGCCCCAGGGAGGGCCTCGGACACGGCCAGCcCTGCCACATCCTGGACCGCAGCCTCCGGCCTCCAGGACTATGG
ST-E00144:68:H2LCKCCXX:3:2211:32861:59253- gggtttctgGctagctgggagacgggggcgccgagatgGcCaGCtagggGtggtgacgtcgcgagggggGGCCtAGTGCaGTGGGTCTGCtGTCCtTAAACAAAGGGGACCCTTAGTTACAGACAGCACCTGCAAGCCCCAGGGAGGGCC
ST-E00144:68:H2LCKCCXX:7:2213:3294:28084 - GGGgGgCctcgtgcaGTGGGTCTGCTGTCCTTAcACaAAGGGgaCcCTTAGTTACAGACAGCACCTGCAaGCCcCAGGGAGGGCCTCGGACACGGCCAGCcCTGCCACATCCTGGACCGCAGCCTCCGGCCTCCAGGACTATGGGGAAAT
ST-E00144:68:H2LCKCCXX:1:1119:13677:23952- TTAAGAGTCCTCTATTTCAAAGGCTTTTTCTTCTATGACAAGATGGGGCCTAGTGCAGTGGGTCTGCTGTCCTTAAACAAAGGGGACCCTTAGTTACAGACAGCACCTGCAAGCCCCAGGGAGGGCCTCGGACACGGCCAGCCCTGCCAC
ST-E00144:68:H2LCKCCXX:1:1119:13961:23952- TTAAGAGTCCTCTATTTCAAAGGCTTTTTCTTCTATGACAAGATGGGGCCTAGTGCAGTGGGTCTGCTGTCCTTAAACAAAGGGGACCCTTAGTTACAGACAGCACCTGCAAGCCCCAGGGAGGGCCTCGGACACGGCCAGCCCTGCCAC
ST-E00144:68:H2LCKCCXX:1:1119:13687:23970- TTAAGAGTCCTCTATTTCAAAGGCTTTTTCTTCTATGACAAGATGGGGCCTAGTGCAGTGGGTCTGCTGTCCTTAAACAAAGGGGACCCTTAGTTACAGACAGCACCTGCAAGCCCCAGGGAGGGCCTCGGACACGGCCAGCCCTGCCAC
ST-E00144:68:H2LCKCCXX:1:1119:13647:24005- TAAGAgtCCTCTATTTCAAAGGCTtTTTcTTCtATGACAAGATGGGGCCTAGTGCAGTGGGTCTGCTGTCCTTAAACAAAGgGGAccCTTAGTTACAGACAGcACCTGCAAGCCCCAGGGAGGGCCTCGGACACGGCCAGCCCTGCCAC
ST-E00144:68:H2LCKCCXX:1:1221:22690:34536- TTAAGAGTCCTCTATTTCAAAGGCTTTTTCTTCTATGACAAGATGGGGCCTAGTGCAGTGGGTCTGCTGTCCTTAAACAAAGGGGACCCTTAGTTACAGACAGCACCTGCAAGCCCCAGGGAGGGCCTCGGACACGGCCAGCCCTGCCAC
ST-E00144:68:H2LCKCCXX:1:1221:22508:35133- TTAaGAGTCCTCTATTTCAAAGGCTTTTTCTTCTATGACAAGATGGGGCCTAGTGCAGTGGGTCTGCTGTCCTTAAACAAAGGGGACCCTTAGTTACAGACAGCACCTGCAAGCCCCAGGGAGGGCCTCGGACACGGCCAGCCCTGCCAC
ST-E00144:68:H2LCKCCXX:4:2217:12621:2504 - GGGGCCTAGTGCaGTGGGTCTGCTGTCCTTAAACAAAGGGGaCCCTTaGTTACAGaCAGCACCTGCAAGCCCCAGGGAGGGCCTCGGACACGGCCAGCCCTGCCACATCCTGGACCGCAGCCTCCGGCCTCCAGGACTATGGGGAAATGC
```

```
ST-E00192:51:H2KG3CCXX:5:2109:3588:16182 + cGCTGTGGGCTGAGCTGTGCCCTGATGCCCCTGTGTGACGACCCCAAcCCCCAGGACTGCGGAGTGTGGCCATGTTTGGAGACAGGGccTCTAGAGAGGccAgTAAGTGAAGGTGAGGTcctGAGGGGGGGcctAGTGaagtgGG
ST-E00192:51:H2KG3CCXX:2:2108:20061:59587+ TGAGCTGTGCCCTGATGCCCCTGTGTGACGACCCCAACCCCCAGGACTGCGGAGtGtGGCCAtGTTTGGAGACAGGGCCTCTAGAGaGGCCAGtaAGTGAAGGTGAGGTCctGAGGGGGGGCCTAGTGcaGTGGGtCTGCTGtccttaaA
ST-E00192:51:H2KG3CCXX:8:2101:17402:41726+ CGACCCCAACCCCCAGGACTGCGGAGTGTGGCCATGTTTGGAGACAGGGCCTCTAGAGAGGCCAGTAAGTGAAGGTGAGGTCCTGAGGGGGGGCCTAGTGCAGTGGGTCTGCTGTCCTTAAACAAAGGGGACCCtTAGTTACAGACAGCA
ST-E00192:51:H2KG3CCXX:2:2121:23320:41427+ GACCCCAACCCCCAGGACTGCGGAGTGTGGCCATGTTTGGAGACAGGGCCTCTAGAGcGGCCAGTAAGTGAAGGTGAGGTCCTGaGGGGGGGCCtAGTGCAGTGGGTCTGCTGTCCTTAAACAAAGGGGACCCTTAGTTACAGACAGCAc
ST-E00192:51:H2KG3CCXX:3:2220:29958:35063+ GGAGTGTGGCCATGTTTGGAGACAGGGCCTCTAGAGAGGCCAGTAAGTGAAGGTGAGGTCCTGAGGGGGGGCCTAGTGCAGTGGGTCTGCTGTCCTTAAACAAAGGGGACCCTtAGTTACAGACAGCACCTGCAAGCCCCAGGGAGGGCC
ST-E00192:51:H2KG3CCXX:6:2224:6511:52467 + ATGTTTGGAGACAGGGCCTCTAGAGAGGCCAGTAAGTGAAGGTGAGGTCCTGAGGGGGGGCCTAGTGCAGTGGGTCTGCTGTCCTTAAACAAAGGGGacccttAGTTACAGACAGCACCTGCAAGCCCCAGGGAGGGcctcGGACACGGC
ST-E00192:51:H2KG3CCXX:6:2224:5983:52714 + ATGTTTGGAGACAGGGCCTCTAGAGcGGCCAGTAAGTGAAGGTGAGGTCCTGAGGGGGGGCCTAGTGCAGTGGGTCtGCTGTCCTTAAACAAAGGGGACCCTTAGTTACAGACAGCACCTGCAAGCCcCAGGGAGGGCgTCGGACACGGC
ST-E00192:51:H2KG3CCXX:2:2109:23482:33832+ AGACAGGGCCTCTAGAGAGGCCAGTAAGTGAAGGTGAGGTCCTGAGGGGGGGCCTAGTGCAGTGGGTCTGCTGTCCTTAAACAAAGGGGACCCTTAGTTACAGACAGCACCTGCAAGCCCCAGGGAGGGCCTCGGACACGGCCAGCCCTG
ST-E00192:51:H2KG3CCXX:6:1110:4836:10644 + GACAGGGCCTCTAGAGAGGCCAGTAAGTGAAGGTGAGGTCCTGAGGGGGGGCCTAGTGCAGTGGGTCTGCTGTCCTTAAACAAAGGGGACCCTTAGTTACAGACAGCACCTGCAAGCCCCAGGGAGGGCCTCGGACACGGCCAGCcCTGC
ST-E00192:51:H2KG3CCXX:8:1121:15971:41673+ AGGGCCTCTAGAGAGGCCAGTAAGTGAAGGTGAGGTCCTGAGGGGGGGCCTAGTGCAGTGGGTCTGCTGTCCTTAAACAAAGGGGACCCTTAGTTACAGACAGCaCCTGCAAGCCCCAGGGAGGGcCtCGGaCaCGGCCAGcCCTGCCAc
ST-E00192:51:H2KG3CCXX:8:1121:15971:41779+ AGGGCCTCTAGAGAGGCCAGTAAGTGAAGGTGAGGTCCTGAGGGGGGGCCTAGTGCAGTGGGTCTGCTGTCCTTAAACAAAGGGGACCCTTAGTTACAGACAGCACCTGCAAGCCCCAGGGAGGGCCTCGGACACGGCCAGCCCTGCCAC
ST-E00192:51:H2KG3CCXX:1:2108:9444:61873 + GGGCCTCTAGAGAGGCCAGTAAGTGAAGGTGAGGTCCTGAGGGGGGGCCTAGTGCAGTGGGTCTGCTGTCCTTAAACAAAGGGGACCCTTAGTTACAGACAGCACCTGCAAGCCCCAGGGAGGGCCTCGGACACGGCCAGCCCTGCCACA
ST-E00192:51:H2KG3CCXX:1:2108:9394:61926 + GGGCCTCTAGAGAGGCCAGTAAGTGAAGGTGAGGTCCTGAGGGGGGGCCTAGTGCAGTGGGTCTGCTGTCCTTAAACAAAGGGGACCCTTAGTTACAGACAGCACCTGCAAGCCCCAGGGAGGGCCTCGGACACGGCCAGCCCTGCCACA
[illegible]
```

```
ST-E00192:51:H2KG3CCXX:3:1118:32394:69415+ TAAGTGAAGGTGAGGTCCTGAGGGGGGGCCTAGTGCAGTGGGTCTGCTGTCCTTAAACAAAGGGGACCCTTAGTTACAGACAGCACCTGCAAGCCCCAGGGAGGGCCTCGGACACGGCCAGCCCTGCCACATCCTGGACCGCAGCCTCCG
ST-E00192:51:H2KG3CCXX:1:2110:9617:42341 + AAgtGAAGGtgAGGTCCTGAGGGGGGGcCTaGTgCAgTGGGTCTGCTGTCCTTAAaCaaaGtGGACCCTTgGTTACaGACAGCACCTGCAAGCCCCAGggAGGGCctCGGacACGgcCAGcCCTGCcaCAtccaggAccGCAGCcTccgg
ST-E00192:51:H2KG3CCXX:5:1212:15321:72579+ AAGTGAAGGTGAGGTCCTGAGGGGGGGCCTAGTGCAGTGGGTCTGCTGTCCTTAAACAAAGGGGACCCTTAGTTACAGACAGCACCTGCAAGCCCCAGgGAGGGCCTCGGacACGGCcAGcCCTGCcacaTCCTGgcccgCagcCTCggg
ST-E00192:51:H2KG3CCXX:2:1110:10886:31248+ GAAGGTGAGGTCCTGAGGGGGGGCCTAGTGCAGTGGGTCTGCTGTCCTTAAACAAAGGGGACCCTTAGTTACAGACAGCACCTGCAAGCCCCAGGGAGGGCCTCGGACACGGCCAGCCCTGCCACATCCTGGACCGCAGCCTCCGGCCTC
ST-E00192:51:H2KG3CCXX:2:1110:10774:31512+ GAAGGTGAGGTCCTGAGGGGGGGCCTAGTGCAGTGGGTCTGCTGTCCTTAAACAAAGGGGACCCTTAGTTACAGACAGCACCTGCAAGCCCCAGGGAGGGCCTCGGACACGGCCAGCCCTGCCACATCCTGGACCGCAGCCTCCGGCCTC
ST-E00192:51:H2KG3CCXX:3:1118:22305:54683+ CTGAGGGGGGGCCTAGTGCAGTGGGTCTGCTGTCCTTAAACAAAGGGGACCCTTAGTTACAGACAGCACCTGCAAGCCCCAGGGAGGGCCTCGGACACGGCCAGCCCTGCCACATCCTGGACCGCAGCCTCCGGCCTCCAGGACTATGGG
ST-E00192:51:H2KG3CCXX:3:1118:22376:54700+ CTGAGGGGGGGCCTAGTGCAGTGGGTCTGCTGTCCTTAAACAAAGGGGACCCTTAGTTACAGACAGCACCTGCAAGCCCCAGGGAGGGCCTCGGACACGGCCAGCCCTGCCACATCCTGGACCGCAGCCTCCGGCCTCCAGGACTATGGG
ST-E00192:51:H2KG3CCXX:2:1223:31957:27310- TGTGATAGCAatGGCGCTGTGGGCTGAGCTGTGCCCTGATGCCCCTGTGtGACGACCCCAACCCCCAGGACTGCGGAGTGTGGCCATGTTTGGAGACAGGGCCTCTAGAGAGGCCAGTAAGTGAAGGTGAGGTCCTGAGGGGGGGCCTAG
ST-E00192:51:H2KG3CCXX:3:2118:10825:24181- GCAATGGCGCTGTgGGCTGAGCTGTGCCCTGATGCCCCTGtGTGACGaCCCCAACCCCCAGGACTGCGGAGTGTGGCCATGTTTGGAGACAGGGCCTCTAGAGAGGCCAGTAAGTGAAGGTGAGGTCCTGAGGGGGGGCCTAGTGCAGTG
ST-E00192:51:H2KG3CCXX:5:1213:21655:23812- GCTGTgCCCTGATGCCCCTGTGTGACGACCCCAACCCCCAGGACTGCGGAGTGTGGCCATGTTTGGAGACAGGGCCTCTAGAGAGGCCAGTAAGTGAAGGTGAGGTCCTGAGGGGGGGCCTAGTGCAGTGGGTCTGCTGTCCTTAAACAA
ST-E00192:51:H2KG3CCXX:8:1101:1335:48248 - cGcgccGtgcGCttAGccGTGCCctGatGCCCCTGTGTGaCgACCCCAACCCCCAGGACTGCGGAGTGTGGCCATGTTTGGAGACAGGGCCTCTAGAGAGGCCAGTAAGTGAAGGTGAGGTCCTGAGGGGGGGCCTAGTGCAGTGGGTCT
ST-E00192:51:H2KG3CCXX:3:2219:23553:33076- cCCTGATGCCCCTGTGtGACGaCCCCAACCCCCAGGACTGCGGAGTGTGGCCATGTTTGGAGAcAGGGCCTCTAGAGAGGCCAGTAAGTGAAGGTGAGGTCCTGAGGGGGGGCCTAGTGCAGTGGGTCTGCTGTCCTTAAACAAAGGGGA
ST-E00192:51:H2KG3CCXX:6:1221:9404:69257 - CCTGATGCCCCTGTGTGACGACCCCAACCCCCAGGACTGCGGAGTGTGGCCATGTTTGGAGACAGGGCCTCTAGAGAGGCCAGTAAGTGAAGGTGAGGTCCTGAGGGGGGGCCTAGTGCAGTGGGTCTGCTGTCCTTAAACAAAGGGGAC
ST-E00192:51:H2KG3CCXX:6:1107:26293:70417- ATGCCCCTGTGTGACGACCCCAACCCCCAGGACTGCGGAGTGTGGCCATGTTTGGAGACAGGGCCTCTAGAGAGGCCAGTAAGTGAAGGTGAGGTCCTGAGGGGGGGCCTAGTGCAGTGGGTCTGCTGTCCTTAAACAAAGGGGACCCTT
ST-E00192:51:H2KG3CCXX:6:1203:10470:38438- CTGTGTGACGACCCCAACCCCCAGgACTGCgGAGTGTGGCCATGTTTGGAGACAGGGCCTCTAGAGAGGCCAGTAAGTGAAGGTGAGGTCCTGAGGGGGGGCCTAGTGCAGTGGGTCTGCTGTCCTTAAACAAAGGGGACCCTTAGTTAC
ST-E00192:51:H2KG3CCXX:2:2105:30699:66620- GaCGaCcCCagCCCCCAGgACTGCGGAGTGTGGCCATGTTTGGAGACAGGGCcTCTAGAGAGGCCAGTAAGTGAAGGTGAGGTCCTGAGGGGGGGCCTAGTGCAGTGGGTCTGCTGTCCTTAAACAAAGGGGACCCTTAGTTACAGACAG
ST-E00192:51:H2KG3CCXX:2:2103:28760:42587- CCaACCCCCAGGaCTGCGGAGtGTGGCCATGTtTGGAGACAGGGCCTCTAGAGAGGCCAGTAAGTGAAGGTGAGGTCCTGAGGGGGGGCCTAGTGCAGTGGGTCTGCTGTCCTTAAACAAAGGGGACCCTTAGTTACAGACAGCACCTGC
ST-E00192:51:H2KG3CCXX:2:2103:28801:42587- CCAaCCCCcAGGACTGCGGAGtGtGGCCATGTTTGGAGACAGGGCCTCTAGAGAGGCCAGTAAGTGAAGGTGAGGTCCTGAGGGGGGGCCTAGTGCAGTGGGTCTGCTGTCCTTAAACAAAGGGGACCCTTAGTTACAGACAGCACCTGC
ST-E00192:51:H2KG3CCXX:6:2105:14936:18995- ACCCCCAGGACTGCGGAGTGTGGCCATGTTTGGAGACAGGGCCTCTAGAGAGGCCAGTAAGTGAAGGTGAGGTCCTGAGGGGGGGCCTAGTGCAGTGGGTCTGCTGTCCTTAAACAAAGGGGACCCTTAGTTACAGACAGCACCTGCAAG
ST-E00192:51:H2KG3CCXX:2:1118:14499:35397- CCCAGGACTGCGGAGTGTGGCCATGTTTGGAGACAGGGCCTCTAGAGAGGCCAGTAAGTGAAGGTGAGGTCCTGAGGGGGGGCCTAGTGCAGTGGGTCTGCTGTCCTTAAACAAAGGGGACCCTTAGTTACAGACAGCACCTGCAAGCCCc
ST-E00192:51:H2KG3CCXX:5:2122:19879:40108- CCCagggCTgCgGAGTGcgGCCATGtttggaGACcggGCcTcTAGAGaggccAGtaagtgaAGgTGAGGTCCTGAgggGGGGCCtAGTgCAGTGGGTCTGctGtccctAAaCAAAGGgGACCcctAGTtACagACAGcaCCTGcaagCCC
ST-E00192:51:H2KG3CCXX:1:2217:15595:26185- GTGGCCATGTTTGGAGACAGGGCCTCTAGAGAGGCCAGTAAGTGAAGGTGAGGTCCTGAGGGGGGGCCTAGTGCAGTGGGTCTGCTGTCCTTAAACAAAGGGGACCCTTAGTTACAGACAGCACCTGCAAGCCCCAGGGAGGGCCTCGGA
ST-E00192:51:H2KG3CCXX:1:2217:15362:26413- GTGGCCATGTTTGGAGACAGGGCCTCTAGAGAGGCCAGTAAGTGAAGGTGAGGTCCTGAGGGGGGGCCTAGTGCAGTGGGTCTGCTGTCCTTAAACAAAGGGGACCCTTAGTTACAGACAGCACCTGCAAGCCCCAGGGAGGGCCTCGGA
ST-E00192:51:H2KG3CCXX:6:2222:9830:63279 - GTGGCCATGTTTGGAGACAGGGCCTCTAGAGAGGCCAGTAAGTGAAGGTGAGGTCCTGAGGGGGGGCCTAGTGCAGTGGGTCTGCTGTCCTTAAACAAAGGGGACCCTTAGTTACAGACAGCACCTGCAAGCCCCAGGGAGGGCCTCGGA

ST-E00192:51:H2KG3CCXX:6:2222:10307:63473- GTGGCCATGTTTGGAGACAGGGCCTCTAGAGAGGCCAGTAAGTGAAGGTGAGGTCCTGAGGGGGGGCCTAGTGCAGTGGGTCTGCTGTCCTTAAACAAAGGGGACCCTTAGTTACAGACAGCACCTGCAAGCCCCAGGGAGGGCCTCGGA
ST-E00192:51:H2KG3CCXX:3:2114:10744:56071- GcCATggTtgGAGACAGGgCcTCTAGAgAGgCCaGTAAGTtAAgGtGAGGtCCTgaGgGgggGccTAGTGCAGTgaGTCTGCtgTCCTTAaaCAAAGgggaCCCTTAGTTACaaACAGCAcCTgcAAgccCCAGGGAgGGccTCGgACAc
ST-E00192:51:H2KG3CCXX:3:2114:10784:56107- GCCaTGTTTGGAGACAGGGCCTCtAGAGAGGCCAGTAAGTGAAGGTGAGGtCCTGAGGGGGGGCCTAGTGCAGTGGGTCTGCTGTCCTTAAACAAAGGGGACCCTTAGTTACAGACAGCACCTGCAAGCCCCAGGGAGGGCCTCGGACAC
ST-E00192:51:H2KG3CCXX:3:2222:23928:23565- GCCATGTTTGGAGACAGGGCCTCTAGAGAGGCCAGTAAGTGAAGGTGAGGTCCTGAGGGGGGGCCTAGTGCAGTGGGTCTGCTGTCCTTAAACAAAGGGGACCCTTAGTTACAGACAGCACCTGCAAGCCCCAGGGAGGGCCTCGGACAC
ST-E00192:51:H2KG3CCXX:1:1103:28953:69890- TGTTTGGAGACaGGGCCTCTAGAGAGGCCAGTAAGTGAAGGTGAGGTCCTGAGGGGGGGCCTAGTGCAGTGGGTCTGCTGTCCTTAAACAAAGGGGACCCTTAGTTACAGACAGCACCTGCAAGCCCCAGGGAGGGCCTCGGACACGGCC
ST-E00192:51:H2KG3CCXX:8:1216:16438:44345- GTTTGGAGACAGGGCCTCTAGAGAGGCCAGTAAGTGAAGGTGAGGtCCTGAGGGGGGGCCTAGTGCAGTGGGTCTGCTGTCCTTAAACAAAGGGGACCCTTAGTTACAGACAGCACCTGCAAGCCCCAGGGAGGGCCTCGGACACGGCCA
ST-E00192:51:H2KG3CCXX:5:2121:20051:3207 - agaggGGCCAGTaCGTGaAgGtGaGGtcctGaGGGGGGGCCTAGTGCAGTGGGTCtGCtGtCcttaaccAAcGgggACCCTTaGtTACAgACAGCACCtGCAAGCCCCAGGGAGGGCCtCGGACACGGCCAGCCCTGCCACATCCTggAC
ST-E00192:51:H2KG3CCXX:2:1204:11322:3348 - GAGGCCAGTAAGTGAAGGTGAGGTCCTGAGGGGGGGCCTAGTGCAGTGGGTCTGCTGTCCTTAAACAAAGGGGACCCTTAGTTACAGACAGCACCTGCAAGCCCCAGGGAGGGCCTCGGACACGGCCAGCCCTGCCACATCCTGGACCGC
ST-E00192:51:H2KG3CCXX:6:1101:28658:54753- AggccagTAAgTGAAGGTGAgGtCCcgAGGGGgGGCCTAGTGCAGTggGTCTGCTGTCCTTAAACAAAGGGGACCCTTAGTTACAGACAGCACCTGCAAGCCCCAGGGAGGGCCTCGGACACGGCCAGCCCTGCCACATCCTGGACCGCA
ST-E00192:51:H2KG3CCXX:6:1114:27501:57425- GAAGGTGAGGTCCTGAGGGGGGGCCTAGTGCAGTGGGTCTGCTGTCCTTAAACAAAGGGGACCCTTAGTTACAGACAGCACCTGCAAGCCCCAGGGAGGGCCTCGGACACGGCCAGCCCTGCCACATCCTGGACCGCAGCCTCCGGCCTC
ST-E00192:51:H2KG3CCXX:3:1208:26415:9800 - GgTGAGGTCCTGAGGGGGGGCCTAGTGCAGTGGGTCTGCTGTCCTTAAACAAAGGGGACCCTTAGTTACAGACAGCACCTGCAAGCCCCAGGGAGGGCCTCGGACACGGCCAGCCCTGCCACATCCTGGACCGCAGCCTCCGGCCTCCAG
ST-E00192:51:H2KG3CCXX:3:2211:19046:56282- TGAGGTCCTGAGGGGGGGCCTAGTGCAGTGGGTCTGCTGTCCTTAAACAAAGGGGACCCTTAGTTACAGACAGCACCTGCAAGCCCCAGGGAGGGCCTCGGACACGGCCAGCCCTGCCACATCCTGGACCGCAGCCTCCGGCCTCCAGGA
```

---

chr20 20759401 + 3 chr14 97681609 + 2 CTX 31 22 51 29 0.952533333333333 0 0.991666666666667 0 1 chr20 20759303 150 chr14 97681664

ATAATAAAAAAAGATATAAAAACCAGTGTTAGCAAGGAGGAGAAACAGTAGAAATGCTTATATACAAATGGTGGAAGTAAATGTTACTACAGTCATTTTTATTCCTATATCTTTATCTTTTCCCATGCCCATATCCATATCCACATCTAT

```
                      20759280  20759300  20759320  20759340  20759360  20759380  20759400  20759420  20759440  20759460  20759480  20759500  20759520  20759540
chr20:20759261-20759550 TATCAATTTTTTTCTCAAATAAATTTTTGACccagaatggctataataaaaaaagatataaaaaccagtgttagcaaggaggagaaacagtagaaatgcttatatacaaatggtggaagtaaatgttactacagtcattttagaaaattatttgttagaaaactaaacatctgagccaagcatttttggcagaattctgaacatgtactcatctatggaaatgtactaaaatgttcacagcagcacagttcatattaacctctgcctggggctcagcacacaataccctg

ST-E00144:68:H2LCKCCXX:8:2213:19513:13035+ TATCAATTTTTTTCTCAAATAAATTTTTgACCCAGAATGGCTATAATAAAAAAAGATATAAAAACCAGTGTTAGCAaGGAGGaGAAACaGTAGAaATGCTTATATACAAATGGTGGAAGTAAATGTTACTACAGTCATTTTAGAAAATTA
ST-E00144:68:H2LCKCCXX:8:2213:19503:13088+ TATCAATTTTTTTCTCAAATAAATTTTTGACCCAGAATGGCTATAATAAAAAAAGATATAAAAACCAGTGTTAGCAAGGAGGAGAAACAGTAGAAATGCTTATATACAAATGGTGGAAGTAAATGTTACTACAGTCATTTTAGAAAATTA
ST-E00144:68:H2LCKCCXX:8:2123:18843:56036+ GGCTATAATAAAAAAAGATATAAAAACCAGTGTTAGCAAGGAGGAGAAACAGTAGAAATGCTTATATACAAATGGTGGAAGTAAATGTTACTACAGTCATTTTAGAAAATTATTTGTTAGAAAACTAAACATCTGAGCCAAGCATTTTTG
ST-E00144:68:H2LCKCCXX:6:2120:28628:13703+ ATAATAAAAAAAGATATAAAAACCAGTGTTAGCAAGGAGGAGAAACAGTAGAAATGCTTATATACAAATGGTGGAAGTAAATGTTACTACAGTCATTTTAGAAAATTATTTGTTAGAAAACTAAACATCTGAGCCAAGCATTTTTggcAg
ST-E00144:68:H2LCKCCXX:8:1121:23868:63895+ ATAATAAAAAAAGATATAAAAACCAGTGTTAGCAAGGAGGAGAAACAGTAGAAATGCTTATATACAAATGGTGGAAGTAAATGTTACTACAGTCATTTTTATTCCTATATCTTTATCTTTTCCCATGCCCATATCCATATCCACATCTAT
ST-E00144:68:H2LCKCCXX:8:1121:23908:63930+ ATAATAAAAAAAGATATAAAAACCAGTGTTAGCAAGGAGGAGAAACAGTAGAAATGCTTATATACAAATGGTGGAAGTAAATGTTACTACAGTCATTTTTATTCCTATATCTTTATCTTTTCCCATGCCCATATCCATATCCACATCTAT
ST-E00144:68:H2LCKCCXX:8:1121:24375:64070+ ATAATAAAAAAAGATATAAAAACCAGTGTTAGCAAGGAGGAGAAACAGTAGAAATGCTTATATACAAATGGTGGAAGTAAATGTTACTACAGTCATTTTTATTCCTATATCTTTATCTTTTCCCATGCCCATATCCATATcCACATCTAT
ST-E00144:68:H2LCKCCXX:1:1206:30008:22563+ ACCAGTGTTAGCAAGGAGGAGAAACAGTAGAAATGCTTATATACAAATGGTGGAAGTAAATGTTACTACAGTCATTTTAGAAAATTATTTGTTAGAAAACTAAACATCTGAGCCAAgCATTTTTGGCAGAATTCTGAACATGTACTCATc
ST-E00144:68:H2LCKCCXX:1:1206:29968:24110+ ACCAGTGTTAGCAAGGAGGAGAAACAGTAGAAATGCTTATATACAAATGGTGGAAGTAAATGTTACTACAGTCATTTTAGAAAATTATTTGTTAGAAAACtAAACATCTGAGCCAAGCATTTTTGcccgacttcTCAaCAtccacccacc
ST-E00144:68:H2LCKCCXX:4:2116:22974:61222+ AGTGTTAGCAAGGAGGAGAAACAGTAGAAATGCTTATATACAAATGGTGGAAGTAAATGTTACTACAGTCATTTTAGAAAATTATTTGTTAGAAAACTAAACATCTGaGCCAAGCATtTTTGGCAGAATTCTGAACATGTACTCATCTAT
ST-E00144:68:H2LCKCCXX:4:2116:22792:61363+ AGTGTTAGCAAGGAGGAGAAACAGTAGAAATGCTTATATACAAATGGTGGAAGTAAATGTTACTACAGTCATTTTAGAAAATTATTTGTTAGAAAACTAAACATCTGAGCCAAGCATTTTTGGCAGAATTCTGAACATGTACTCATCTAT
ST-E00144:68:H2LCKCCXX:3:2223:27248:54243+ GTTAGCAAGGAGGAGAAACAGTAGAAATGCTTATATACAAATGGTGGAAGTAAATGTTACTACAGTCATTTTAGAAAATTATTTGTTAGAAAACTAAACATCTGAGCCAAGCATTTTTGGCAGAATTCTGAACATGTACTCATCTATGGA
ST-E00144:68:H2LCKCCXX:3:1117:20498:33621+ TAGCAAGGAGGAGAAACAGTAGAAATGCTTATATACAAATGGTGGAAGTAAATGTTACTACAGTCATTTTAGAAAATTATTTGTTAGAAAACTAAACATCTGAGCCAAGCATTTTTGGCAGAATTCTGAACATGTACTCATCTATGGAAA
ST-E00144:68:H2LCKCCXX:4:1113:4156:60941 + ACaGTAGAAATGCTTATATACAAATGGTGGAAGTAAaTGTTACTACAGTCATTTTAGAAAATTATTTGTTAGAAAACTAAACATCTGAGCCAAGCATTTTTGGCAGAATTCTGAACATGTACTCATCTATGgAaATGTACTAAAATGTTC
ST-E00144:68:H2LCKCCXX:4:1113:4014:61047 + ACAGTAGAAATGCTTATATACAAATGGTGGAAGTAAATGTTACTACAGTCATTTTAGAAAATTATTTGTTAGAAAACTAAACATCTGAGCCAAGCATTTTTGGCAGAATTCTGAACATGTACTCATCTATGGAAATGTACTAAAATGTTC
ST-E00144:68:H2LCKCCXX:8:1223:20173:68044+ ACAGTAGAAATGCTTATATACAAATGGTGGAAGTAAATGTTACTACAGTCATTTTAGAAAATTATTTGTTAGAAAACTAAACATCTGAGCCAAGCATTTTTGGCAGAATTCTGAACATGTACTCATCTATGGAAATGTACTAAAATGTTC
ST-E00144:68:H2LCKCCXX:3:2121:18427:54015+ GTAGAAATGCTTATATACAAATGGTGGAAGTAAATGTTACTACAGTCATTTTAGAAAATTATTTGTTAGAAAACTAAACATCTGAGCCAAGCATTTTTGGCAgAATTCTGAACATGTACTCATCTATGGAAATGTACTAAAATGTTcACA
ST-E00144:68:H2LCKCCXX:3:2211:25827:49180+ AGAAATGCTTATATACAAATGGTGGAAGTAAATGTTACTACAGTCATTTTAGAAAATTATTTGTTAGAAAACTAAACATCTGAGCCAAGCATTTTTGGCAGAATTCTGAACATGTACTCATCTATGGAAATGTACTAAAATGTTCACAGC
ST-E00144:68:H2LCKCCXX:4:2124:5679:33551 + AGAAATGCTTATATACAAATGGTGGAAGTAAATGTTACTACAGTCATTTTAGAAAATTATTTGTTAGAAAACTAAACATCTGAGCCAAGCATTTTTGGCtGAATTCTGAACATGTACTCATCTATGGAAATGTACTAAAATGtTCACAGC
ST-E00144:68:H2LCKCCXX:6:1104:27207:26466+ ATACAAATGGTGGAAGTAAATGTTACTACAGTCATTTTAGAAAATTATTTGTTAGAAAACTAAACATCTGAGCCAAGCATTTTTGGCAGAATTCTGAACATGTACTCATCTATGGAAATGTACTAAAATGTTCACAGCAGCACAGTTCAT
ST-E00144:68:H2LCKCCXX:1:1106:12956:9097 + ACAAATGGTGGAAGTAAATGTTACTACAGTCATTTTAGAAAATTATTTGTTAGAAAACTAAACATCTGAGCCAAGCATTTTTGGCAGAATTCTGAACATGTACTCATCTATGGAAATGTACTAAAATGTTCACAGCAGCACAGTTCATAT
ST-E00144:68:H2LCKCCXX:1:1106:13504:10011+ ACAAATGGTGGAAGTAaaTGTTaCTACAGTCATtTtAGAAAATTATTTGTTaGaaAACTAAACATCTGAGCCAAgCATTttTgGCaGaATTCTgaACATGtACTCATCTctGgaAacgtaCtaaaatGttCACAGCAGCACAGctCatAt
ST-E00144:68:H2LCKCCXX:7:2209:10165:11400+ ATGGTGGAAGTAAATGTTACTACAGTCATTTTAGAAAATTATTTGTTAGAAAACTAAACATCTGAGCCAAGCATTTTTGGCAGAATTCTGAACATGTACTCATCTATGGAAATGTACTAAAATGTTCACAGCAGCACAGTTCATATTAAc
ST-E00144:68:H2LCKCCXX:8:2112:10388:21737+ TGGTGGAAGTAAATGTTACTACAGTCATTTTAGAAAATTATTTGTTAGAAAACTAAACATCTGAGCCAAGCATTTTTGGCAGAATTCTGAAcATGTACTCATCTATGGaAATGTACTaAAATGTTCACAGCAGCACAGTTCATATTAACC
ST-E00144:68:H2LCKCCXX:8:1224:8490:70382 + TAAATGTTACTACAGTCATTTTAGAAAATTATTTGTTAGAAAACTAAACATCTGAGCCAAGCATTTTTTtctgaaTTCTGAACATGTAcTcATCTATGGAAATGTACTAAAATGTTCACAGCAGCACAGTTCATATTAACCTCTgcCTgg
ST-E00144:68:H2LCKCCXX:4:1122:5882:47088 + CAGTCATTTTAGAAAATTATTTGTTAGAAAACTAAACATCTGAGCCAAGCATTTTTGGCAGAATTCTGAACATGTACTCATCTATGGAAATGTACTAAAATgtTCACAGCAGCACAGTTCATATTAACCTCTGCCTgGggCTCAGcaCaC
ST-E00144:68:H2LCKCCXX:8:2116:5110:44732 + AGTCATTTTAGAAAATTATTTGTTAGAAAACTAAACATCTGAGCCAAGCATTTTTGGCAGAATTCTGAACATGTACTCATCTATGGAAATGTACTAAAATGTTCACAGCAGCACAGTTCATATTAACCTCTGCCTGGGGCTCAGCACACA
ST-E00144:68:H2LCKCCXX:3:2220:20173:54507+ TAGAAAATTATTTGTTAGAAAACTAAACATCTGAGCCAAGCATTTTTGGCAGAATTCTGAACATGTACTCATCTATGGAAATGTACTAAAATGTTCACAGCAGCACAGTTCATATTAACCTCTGCCTGGGGCTCAGCACACAATAcCCTG
ST-E00144:68:H2LCKCCXX:7:1208:23198:13931+ TAGAAAATTATTTGTTAGAAAACTAAACATCTGAGCCAAGCATTTTTGGCAGAATTCTGAACATGTACTCATCTATGGAAATGTACTAAAATGTTCACAGCAGCACAGTTCATATTAACCTCTGCCTGGGGCTCAGCACACAATACCCTG
ST-E00144:68:H2LCKCCXX:8:1119:3172:26994 + TAGAAAATTATTTGTTAGAAAACTAAACATCTGAGCCAAGCATTTTTTttagaaTTtTGAACATGTACTCATCTATGGAAATGTACTAAAATGTTCACAGCAGCACAGgTCatATTAACCTCTGCCTGGGgCTCaGcacAcaATAacCtg
ST-E00144:68:H2LCKCCXX:8:1119:3730:27960 + TAGAAAAtTATTTGTTAGAAAACTAAACATCTGAGCCAAGCATTTTTGGCAGAATTCTGAACATGTACTCATCTATGGAAATGTACTAAAATGTTtACAGCagCAcAgtTCATATTAACCtCTGCCTGGGgCtCAGCAcACAATACCcTG
ST-E00144:68:H2LCKCCXX:8:1209:26293:67850- AAATAAATaTtTGAcCCAGAATGGaTATAATAAAAAAAGATATAAAAACCAGTGTTAgCAAGGAGGAGAAACAGTAGAAATGCTTATATACAAATGGTGGAAGTAAATGTTACTACAGTCATTTTAGAAAATTATTTGTTAGA
ST-E00144:68:H2LCKCCXX:8:1111:19544:72404- AAAAAAAGAtATAAAAACCAGTGtTAGCAAGGAGGAGAAACAGTAGAAATGCTTATATACAAATGGTGGAAGTAAATGTTACTACAGTCATTTTAGAAAATTATTTGTTAGAAAACTAAACATCTGAGCCAAGCATTTTTGGCAGAATTC
ST-E00144:68:H2LCKCCXX:8:2217:27045:24321- TAGCAAGGAgGAGAAACAGTAGAAATGCTTATATACAAATGGTGGAAGTAAATGTTACTACAGTCATTTTAGAAAATTATTTGTTAGAAAACTAAACATCTGAGCCAAGCATTTTTGGCAGAATTCTGAACATGTACTCATCTATGGAAA
ST-E00144:68:H2LCKCCXX:6:2113:4867:31793 - AGGAGAAACAGTAGAAATGCTTATATACAAATGGTGGAAGTAAATGTTACTACAGTCATTTTAGAAAATTATTTGTTAGAAAACTAAACATCTGAGCCAAGCATTTTTGGCAGAATTCTGAACATGTACTCATCTATGGAAATGTACtAA
ST-E00144:68:H2LCKCCXX:6:2113:4826:31863 - AGGAGAAACAGTAGAAATGCTTATATACAAATGGTGGAAGTAAATGTTACTACAGTCATTTTAGAAAATTATTTGTTAGAAAACTAAACATCTGAGCCAAGCATTTTTGGCAGAATTCTGAACATGTACTCATCTATGGAAATGTACTAA
ST-E00144:68:H2LCKCCXX:6:2113:4816:31881 - AgGAGAAACAGTAGAAATGCTTATATACAAATGGTGGAAGTAAATGTTACTACAGTCATTTTAGAAAATTATTTGTTAGAAAACTAAACATCTGAGCCAAGCATTTTTGGCAGAATTCTGAACATGTACTCATCTATGGAAATGTACTAA
ST-E00144:68:H2LCKCCXX:3:2203:19767:18397- GAGAAACAGTAGAAATGCTTATATACAAATGGTGGAAGTAAATGTTACTACAGTCATTTTAGAAAATTATTTGTTAGAAAACTAAACATCTGAGCCAAGCATTTTTGGCAGAATTCTGAACATGTACTCATCTATGGAAATGTACTAAAA
ST-E00144:68:H2LCKCCXX:1:2212:22477:60572- GaaacAGTAGAAATGCtTATATACAAAtGGTggAAGTAAATGTTACTACAGTCATTTTAGAAAATTATTTGTTAGAAAACTAAACaTCTGAGCCAAGCATTTTTGGCAGAATTCTGAACATGTACTCATCTATGGAAATGTACTAAAAt
ST-E00144:68:H2LCKCCXX:1:2104:23502:24761- AAATGCTTATATACAAATGGTGGAAGTAAATGTTACTACAGTCATTTTAGAAAATTATTTGTTAGAAAACTAAACATCTGAGCCAAGCATTTTTGGCAGAATTCTGAACATGTACTCATCTATGGAAATGTACTAAAATGTTCACAGCAG
ST-E00144:68:H2LCKCCXX:6:1122:2816:34043 - GgAAGTAAaTGTTACTACAGTCATTTTAGAAAATTATTTGTTAGAAAACTAAACATCTGAGCCAAGCATTTTTGGCAGAATTCTGAaCATGTACTCATCTATGGAAATGTACTAAAATGTTCACAGCAGCACAGTTCATATTAACCTCTG
ST-E00144:68:H2LCKCCXX:6:1122:3314:35467 - GGAAGTaAATGTTACTACAGTCATTTTAGAAAATTATTTGTTAGAAAACTAAACATCTGAGCCAAGCATTTTTGGCAGAATTCTGAACATGTACTCATCTATGGAAATGTACTAAAATGTTCACAGCAGCACAGTTCATATTAACCTCTG
ST-E00144:68:H2LCKCCXX:7:2113:23533:30474- ACAGTCATTTTAGAAAaTTATTTGTTAGAAAACTAAACATCTGaGCCAAGCATTTTTGGCAGAATTCTGAACATGTACTCAtCTAcgGAAATGTACTAAAATGTTCACAGCAGCACAGTTCATATTAACCTCTGCCTGGGGCTCAGCACA
---
ST-E00192:51:H2KG3CCXX:3:1113:17016:72491+ AATTTTTTTCTCAAATAAATTTTTGACCCAGAATGGCTATAATAAAAAAAGATATAAAAACCAGTGTTAGCAAGGAGGAGAAACAGTAGAAATGCTTATATACAAATGGTGGAAGTAAATGTTACTACAGTCATTTTAGAAAATTATTTG
ST-E00192:51:H2KG3CCXX:1:1202:28151:50287+ TTTTTTTCTCAAATAAATTTTTGACCCAGAATGGCTATAATAAAAAAAGATATAAAAACCAGtGTTAGcAAGGAGGAGAAACAGTAGAAATGCTTATATACAAATGGTGGAAGTAAATGTTAcTAcAGTcATTTTAGAAaATTATTTGTT
ST-E00192:51:H2KG3CCXX:8:1102:28679:18889+ TTTTTTTCTCAAATAAATTTTTGACCCAGAATGGCTATAATAAAAAAAGATATAAAAACCAGTGTTAGCAAGGAGGAGAAACAGTAGAAATGCTTATATACAAATGGTGGAAGTAAATGTTACTACAGTCATTTTAGAAAATTATTTGTT

ST-E00192:51:H2KG3CCXX:3:2101:8145:52591 + TTTCTCAAATAAATTTTTGACCCAGAATGGCTATAATAAAAAAAGATATAAAAACCAGTGTTAGCAAGGAGGAGAAACAGTAGAAATGCTTATATACAAATGGTGGAAGTAAATGTTACTACAGTCATTTTAGAAAATTATTTGTTAGAA
ST-E00192:51:H2KG3CCXX:5:2115:18569:32180+ CCcAGAATGGCTATAATAAAaAAAGATATAAAAACCAGTGTTAGCAAGGAGGAGAAACAGTAGAAATGCTTATATACAAATGGTGGAAGTAAATGTTACTAcAGTCATTTTAGAAAATTATTTGTTAGAAAACTAAACATCTgAGCCAaG
ST-E00192:51:H2KG3CCXX:3:1119:23401:60590+ TAAAAAAAGATATAAAAACCAGTGTTAGCAAGGAGGAGAAACAGTAGAAATGCTTATATACAAATGGTGGAAGTAAATGTTACTACAGTCATTTTAGAAAATTATTTGTTAGAAAACTAAACATCTGAGCCAAGCATTTTTGGCAGAATT
ST-E00192:51:H2KG3CCXX:3:1119:23340:60660+ TAAAAAAAGATATAAAAACCAGTGTTAGCAAGGAGGAGAAACAGTAGAAATGCTTATATACAAATGGTGGAAGTAAATGTTACTACAGTCATTTTAGAAAATTATTTGTTAGAAAACTAAACATCTGAGCCAAGCATTTTTGGCAGAATT
ST-E00192:51:H2KG3CCXX:2:2216:25329:7321 + AAAAAAAGATATAAAAACCAGTGTTAGCAAGGAGGAGAAACAGTAGAAATGCTTATATACAAATGGTGGAAGTAAATGTTACTACAGTCATTTTAGAAAATTATTTGTTAGAAAACTAAACATcTGAGCCAAGCATTTTTGGCAGAATTC
ST-E00192:51:H2KG3CCXX:2:1109:22995:51518+ TATAAAAACCAGTGTTAGCAAGGAGGAGAAACAGTAGAAATGCTTATATACAAATGGTGGAAGTAAATGTTACTACAGTCATTTTAGAAAATTATTTGTTAGAAAACTAAACATCTGAGCCAAGCATTTTTGGCAGAATTCTGAACATGT
ST-E00192:51:H2KG3CCXX:2:1109:22964:51571+ TATAAaAAcCAGTGTTAGCAAGGAGGaGAAACAGTAGAAATGCTTATATACAAAtGGTGGAAGTAAATGTTACTACAGTCATTTTAGAAAATtATTTGTTAGAAAACTAAACATCTGAGCCAAGCATTTTTGGCAGAATTCTGAACATGT
ST-E00192:51:H2KG3CCXX:8:2211:25492:71278+ AAAACCAGTGTTAGCAAGGAGGAGAAACAGTAGAAATGCTTATATACAAATGGTGGAAGTAAATGTTACTACAGTCATTTaAGAAAATTATTTGTTAGAAaACTAAACATCTGAGCaaAGCATTTTTGGcaGtatTcTGaAcATGTACTC
ST-E00192:51:H2KG3CCXX:2:1224:4796:53048 + AcAAGTGTTAGaAAGGAGGAGAAACAGTAGAAATGCTTATATACAAaTGGTGGAAGtAAATGTTActacagacAttTTAGAAAaatATTTgttaGaAAaCTaAAcaTaTGagcaAAgcattattgGCAGAaTgctgAacATGTActcATc
ST-E00192:51:H2KG3CCXX:1:2108:25004:69731+ TAGAAATGCTTATATACAAATGGTGGAAGTAAATGTTACTACAGTCATTTTAGAAAATTATTTGTTAGAAAACTAAACATCTGAGCCAAGCATTTTTGGCAGAATTCTGAACATGTACTCATCTATGGAAATGTACTAAAATGTTCACAG
ST-E00192:51:H2KG3CCXX:3:2213:13636:8446 + TAGAAATGCTTATATACAAATGGTGGAAGTAAATGTTACTACAGTCATTTTAGAAAATTATTTGTTAGAAAACTAAACATCTGAGCCAAGCATTTTTGGCAGAATTCTGAACATGTACTCATCTATGGAAATGTACTAAAATGTTCACAG
ST-E00192:51:H2KG3CCXX:2:1121:20457:53733+ AGAAATGCTTATATACAAATGGTGGAAGTAAATGTTACTACAGTCATTTTAGAAAATTATTTGTTAGAAAACTAAACATCTGAGCCAAGCATTTTTGGCAGAATTCTGAACATGTACTCATCTATGGAAATGTACTAAAATGTTCACAGC
ST-E00192:51:H2KG3CCXX:3:1217:6653:50569 + AGAAATGCTTATATACAAATGGTGGAAGTAAATGTTACTACAGTCATTtTAGAAAATTATTTGTTAGAAAACTAAACATCTGAGCCAAGCATTTTTggCagaatTcTgAAcATGTACTCATCTATGGAAATGtACTAaAATGtTCACAGc
ST-E00192:51:H2KG3CCXX:8:1206:23523:22862+ GGAAGTAAATGTTACTACAGTCATTTTAGAAAATTATTTGTTAGAAaACTAAACATCTGAGCCAAGCATTTTTGGCAGAgtTcTGAACATGTACTCATCTatGGaaAtGTACtAaaATGTTCACAGCAGCACAGTTCATATTAACCTcTG
ST-E00192:51:H2KG3CCXX:6:1115:27846:19241+ GTCATTTTAGAAAATTATTTGTTAGAAAACTAAACATCTGAGCCAAGCATTTTTGGCAGAATTCTGAACATGTACTCATCTATGGAAATGTACTAAAATGTTCACAGCAGCACAGTTCATATTAACCTCTGCCTGGGGCTCAGCACACAA
ST-E00192:51:H2KG3CCXX:5:1207:8835:36522 - TGACCCAGAATGGCTATAATAAAAAAAGATATAAAAACCAGTGTTAGCAAGGAGGAGAAACAGTAGAAATGCTTATATACAAATGGTGGAAGTAAATGTTACTACAGTCATTTTAGAAAATTATTTGTTAGAAAACTAAACATCTGAGCC
ST-E00192:51:H2KG3CCXX:2:1208:10825:31670- GCTATAATAAAAAAAGATATAAAAACCAGTGTTAGCAAGGAGGAGAAACAGTAGAAATGCTTATATACAAATGGTGGAAGTAAATGTTACTACAGTCATTTTAGAAAATTATTTGTTAGAAAACTAAACATCTGAGCCAAGCATTTTTGG
ST-E00192:51:H2KG3CCXX:5:1124:14621:35749- AACCAGTGTTAGCAAGGAGGAGAAACAGTAGAAATGCTTATATACAAATGGTGGAAGTAAATGTTACTACAGTCATTTTAGAAAATTATTTGTTAGAAAACTAAACATCTGAGCCAAGCATTTTTGGCAGAATTCTGAACATGTACTCAT
ST-E00192:51:H2KG3CCXX:3:2101:8145:52591 - CCAGTGTTAgCAAGGAGGAGAAACAGTAGAAATGCTTATATACAAATGGTGGAAGTAAATGTTACTACAGTCATTTTAGAAAATTATTTGTTAGAAAACTAAACATCTGAGCCAAGCATTTTTGGCAGAATTCTGAACATGTACTCATCT
ST-E00192:51:H2KG3CCXX:2:2201:18184:37173- GCAAGGAGGAGAAACAGTAGAAATGCTTATATACAAATGGTGGAAGTAAATGTTACTACAGTCATTTTAGAAAATTATTTGTTAGAAAACTAAACATCTGAGCCAAGCATTTTTGGCAGAATTCTGAACATGTACTCATCTATGGAAATG
ST-E00192:51:H2KG3CCXX:8:1121:25624:62646- GGAGGAGAAACAGTAGAAATGCTTATATACAAATGGTGGAAGTAAATGTTACTACAGTCATTTTAGAAAATTATTTGTTAGAAAACTAAACATCTGAGCCAAGCATTTTTGGCAGAATTCTGAACATGTACTCATCTATGGAAATGTACT
ST-E00192:51:H2KG3CCXX:8:2214:28192:67024- GTAGAAAtgttTATATACAAATGGTGGAAGTAAATGTTACTACAGTCATTTTAGAAcATTATTTGCTAGAAAACTAAACATCTGAcCCAAGCATTtTTGGCAGAATTCTGAACATcTACTCATCTATGGAAATGTACTAAAATGTTCACA
ST-E00192:51:H2KG3CCXX:5:1106:17585:49953- TAGAAATGCTTATATACAAATGGTGGAAGTAAATGTTACTACAGTCATTTTAGAAAATTATTTGTTAGAAAACTAAACATCTGAGCCAAGCATTTTTGGCAGAATTCTGAACATGTACTCATCTATGGAAATGTACTAAAATGTTCACAG
ST-E00192:51:H2KG3CCXX:5:1106:17666:49989- [illegible]
```

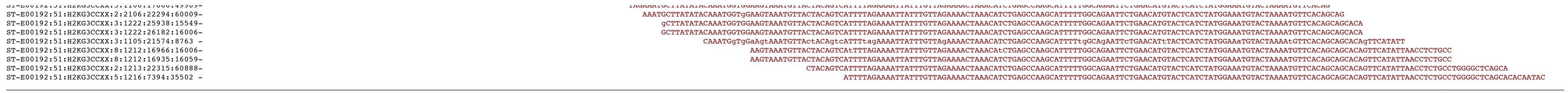

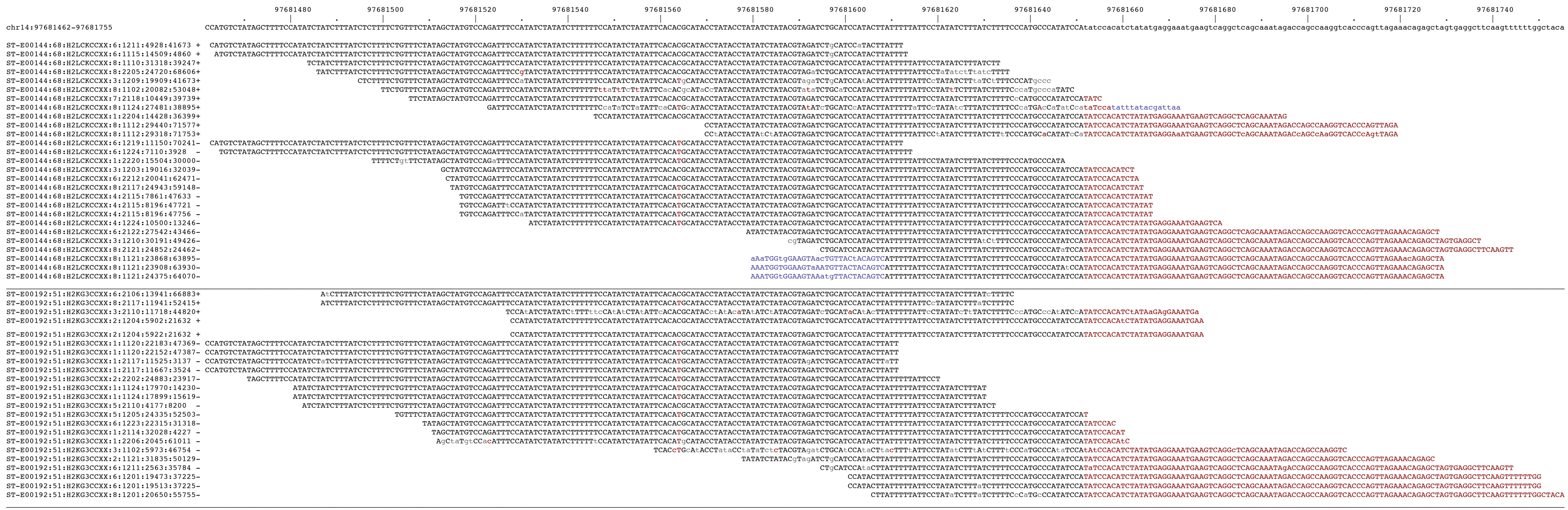

chrX 84546104 - 3 chr22 27071493 + 3 CTX 31 43 75 75 0.916853094705444 0 0.966666666666667 0 1 chrX 84546178 150 chr22 27071567

TGCCAGGATAACTGCCTAGCCATATGTAGAAGATTGAAATTGGACCCCTCGTTACACTATATGCAAAAATAAACTCTAGGAAGACCCTTTGGTCCATCTTTTGATAAACAGAATCCATACATGTCTACCCCAGTTGGAAGTATCTCTGCA

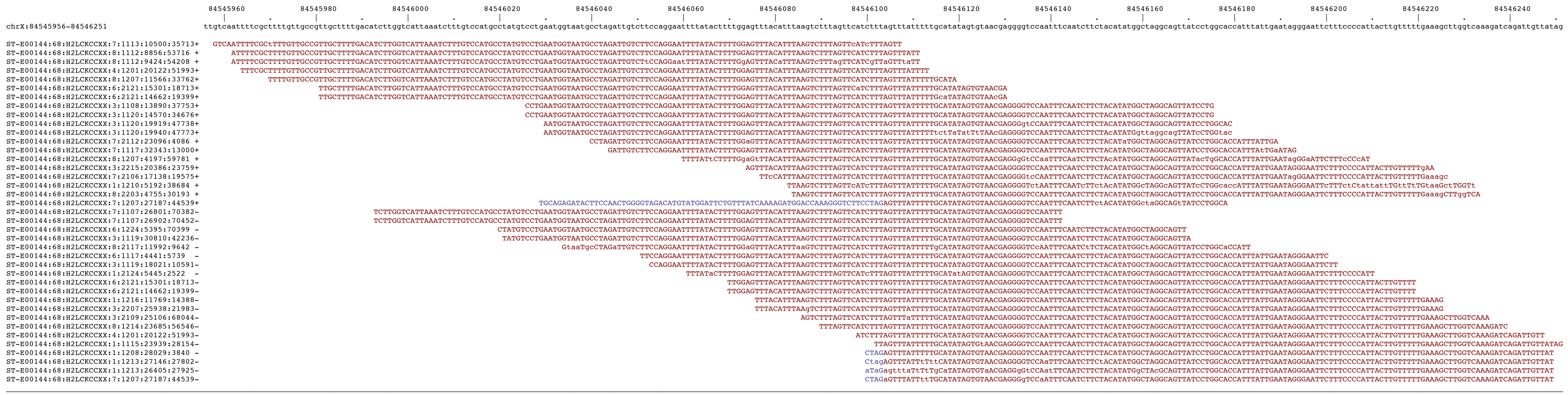

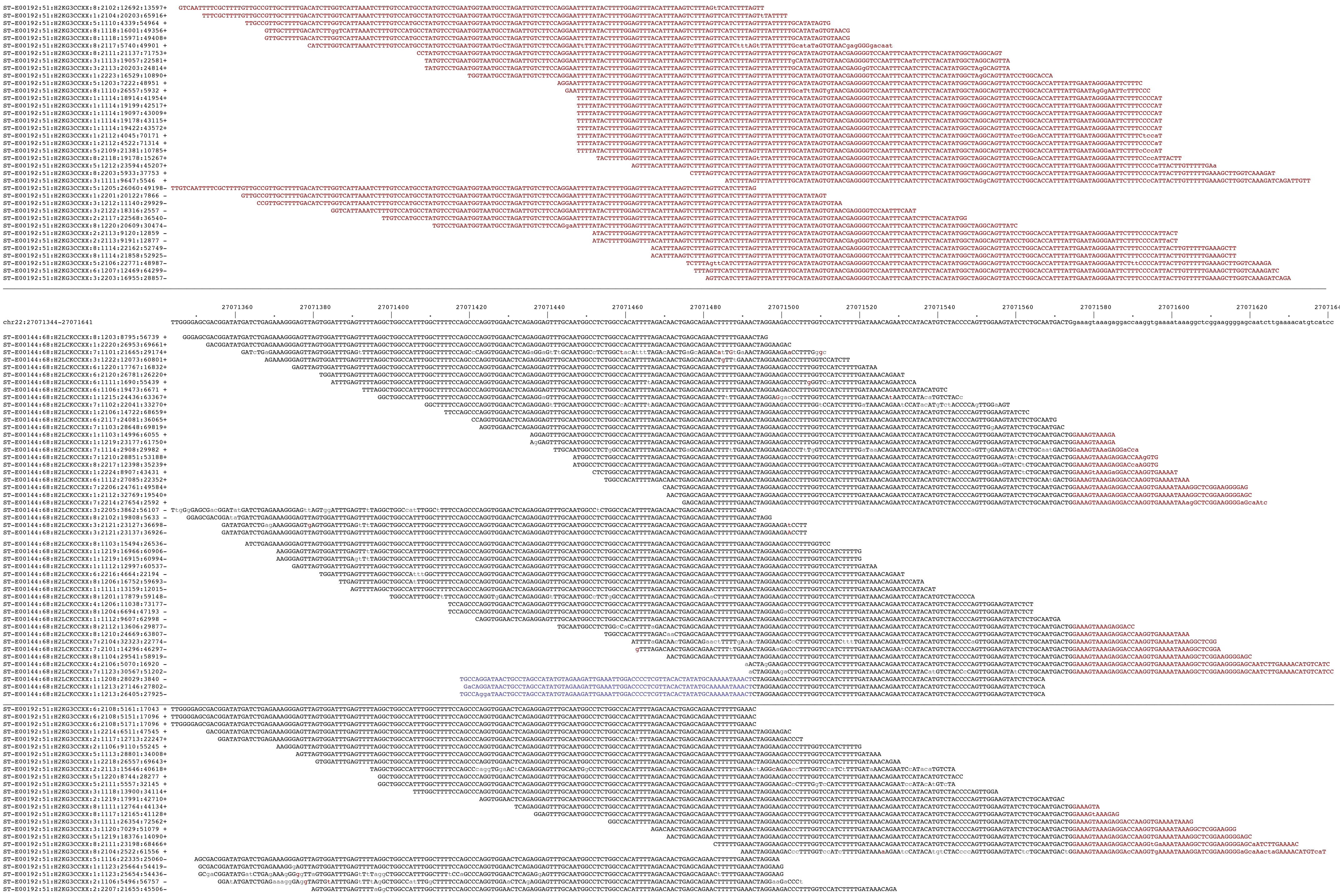
27071360
27071380
27071400
27071420
27071440
27071460
27071480
27071500
27071520
27071540
27071560
27071580
27071600
27071620
27071640
chr22:27071344-27071641

```
ST-E00192:51:H2KG3CCXX:2:2207:21675:45506-        AGTGGATTTGAGTTTTAGGCTGGCCATTTGGCTTTTCCAGCCCAGGTGGAACTCAGAGGAGTTTGCAATGGCCTCTGGCCACATTTTAGACAACTGAGCAGAACTTTTTGAAACTAGGAAGACCCTTTGGTCCATCTTTTGATAAACAGA
ST-E00192:51:H2KG3CCXX:5:1103:25492:6583 -          GATTTGAGTtTTAGGCTGGCCATTTGGCTTTTCCAGCCCAGGTGGAACTCAGAGGAGTTTGCAATGGCCTCTGGCCACATTTTAGACAACTGAGCAGAACTTTTTGAAACTAGGAAGACCCTTTGGTCCATCTTTTGATAAACAGAATCC
ST-E00192:51:H2KG3CCXX:3:2208:17839:34114-              TTTAGGCTGGCCATTTGGCTTTTCCAGCCCAGGTGGAACTCAGAGGAGTTTGCAATGGCCTCTGGCCACATTTTAGACAACTGAGCAGAACTTTTTGAAACTAGGAAGACCCTTTGGTCCATCTTTTGATAAACAGAATCCATCATACATGTC
ST-E00192:51:H2KG3CCXX:6:2108:16641:52573-                  GcCtGGcCATtTggctTtTCCAgcCCAgGTGGAACTCAGAGGAGTTTGCAATGGCCTCTgGCCACAtTTTAGAcAACTgagCAgAAcTTtTTGaAACTAGGaAGACCCTTTGGTCCATCTTTTGATAAACAGAATCCATCATACATGTCTACC
ST-E00192:51:H2KG3CCXX:6:1119:13565:2698 -                   GGCCATTTGGCTTTTCCAGCCCAGGTGGAACTCAGAGGAGTTTGCAATGGCCTCTGGCCACATTTTAGACAACTGAGCAGAACTTTTTGAAACTAGGAAGACCCTTTGGTCCATCTTTTGATAAACAGAATCCATCATACATGTCTACCCCAG
ST-E00192:51:H2KG3CCXX:2:2210:30252:2030 -                        TCCAGCCCAGGTGgAACTCAGAgGAGTTTGCAATGGCCTCTGGCCACATTTTAGACAACTGAGCAGAACTTTTTGAAACTAGGAAGACCCTTTGGTCCATCTTTTGATAAACAGAATCCATCATACATGTCTACCCCAGTTGGAAGTATCTCT
ST-E00192:51:H2KG3CCXX:1:1113:24263:36100-                                CTCAgAGgagTTTGCAATGgCCTCTGGCCACATTTTAGACAACTGAGCAGAACTTTTTGAAACTAGGAAGACCCTTTGGTCCATCTTTTGATAAACAGAATCCATCATACATGTCTACCCCAGTTGGAAGTATCTCTGCAATGACTGGAAAGT
ST-E00192:51:H2KG3CCXX:2:2106:6775:57109 -                                        GGcctCtGGCCACATTttAGACAACTGAGCAGAACTTTTTGAAACTAGGAAGACCCTTTGGTCCATCTTTTGATAAACAGAATCCATCATACATGTCTACCCCAGTTGGAAGTATCTCTGCAATGACTGGAAAGTAAAGAGGACCAAGGTGAA
ST-E00192:51:H2KG3CCXX:8:1113:8693:67042 -                                          CTCTGGCCACATTTTAGACAACTGAGCAGAACTTTTTGAAACTAGGAAGACCCTTTGGTCCATCTTTTGATAAACAGAATCCATCATACATGTCTACCCCAGTTGGAAGTATCTCTGCAATGACTGGAAAGTAAAGAGGACCAAGGTGAAAAT
ST-E00192:51:H2KG3CCXX:8:1113:8714:67112 -                                          cTCTGGCCACATTTTAGACAACTGAGCAGAACTTTTTGAAACTAGGAAGACCCTTTGGTCCATCTTTTGATAAACAGAATCCATCATACATGTCTACCCCAGTTGGAAGTATCTCTGCAATGACTGGAAAGTAAAGAGGACCAAGGTGAAAAT
ST-E00192:51:H2KG3CCXX:8:2209:25603:38878-                                             tgGCCACATTTTacACAACTGagCAGAACTTTTTGAAACTAGGAAGAcCCtTtgGTCCaTcTTtTGATAAAcAgAATCCATcAtACATGtCTACCCCAGTTGGaAgTATCTCTGCAATGACTGGAAAGTAAAGAGGACCAAGGTGAAAATAAA
ST-E00192:51:H2KG3CCXX:1:2215:4481:3418  -                                                TTTTAGAcgACTGAGCAGAACTTtTTGAAACTAGGAAGACCCTTTGGTCCATCTTTTGATAAaCAGAATCCATCATACATGTCTACCCCAGTTGGAAGTATCTCTGCAATGACTGGAAAGTAAAGAGGACCAAGGTGAAAATAAAGGCTCGGA
ST-E00192:51:H2KG3CCXX:1:2215:5435:3875  -                                                TTTTAGACAACTGAGCAGAACTTTTTGAAACTAGGAAGACCCTTTGGTCCATCTTTTGATAAACAGAATCCATCATACATGTCTACCCCAGTTGGAAGTATCTCTGCAATGACTGGAAAGTAAAGAGGACCAAGGTGAAAATAAAGGCTCGGA
ST-E00192:51:H2KG3CCXX:6:2112:19087:28963-                                                     CAACTGAGCAGAACTTTTTGAAACTAGGAAGACCCTTTGGTCCATCTTTTGATAAACAGAATCCATCATACATGTCTACCCCAGTTGGAAGTATCTCTGCAATGACTGGAAAGTAAAGAGGACCAAGGTGAAAATAAAGGCTCGGAGGGGAG
ST-E00192:51:H2KG3CCXX:5:2103:4755:61943 -                                                      AgCTgaGCAtaACTTTTttaaaCTAgGAAGACCCTTtGTcCATCTTTTgATAAACAgAATCATACATGTCTACCCCAGTTGGAAGTATCTCTGCAATGACTGGAAAGTAAAGAGGACCAAGGTGAAAATAAAGGCTCGGAGGGGA
ST-E00192:51:H2KG3CCXX:3:2221:30546:54155-                                                        TGAGCAGAACTTTTTGAAACTAGGAAGACCCTTTGGTCCATCTTTTGATAACCAGAATCCATCATACATGTCTACCCCAGTTGGAAGTATCTCTGCAATGACTGGAAAGTAAAGAGGACCAAGGTGAAAATAAAGGCTCGGAGGGGAGCAAT
ST-E00192:51:H2KG3CCXX:6:2206:13667:47000-                                                           CAgAACTTTTTGAAACTAGGAAGaCCCTTTGGTCCATCTTTTGATAAACAGAATCCATCATACATGTCTACCCCAGTTGGAAGTATCTCTGCAATGACTGGAAAGTAAAGAGGACCAAGGTGAAAATAAAGGCTCGGAGGGGAGCAATCTTG
ST-E00192:51:H2KG3CCXX:6:2107:7283:26343 -                                                            TTTTtgaaacgAGGaagACCCgTGGTCCATCTTTtGATAaaCAGAATCCATCATACATGTCTACCCCaGTTGGAAGTATCTCTGCAATGACTGGAAAGTAAAGAGGACCAAGGTGAAAATAAAGGCTCGGAGGGGAGCAATCTTGAAAC
ST-E00192:51:H2KG3CCXX:1:1214:15068:62752-                                                              TTGAAACTAGGAAGACCCTTTGGTCCATCTTTTGATAAAcagaATCCATCATACATGTCTACCCCAGTTGGAAGtATCTCTGCAATGACTGGAAAGTAAAGAGGACCAAGGgGAAAATAAAGGCTCGGAGGGGAGCAATCTTGAAAACATGT
ST-E00192:51:H2KG3CCXX:1:2110:26547:27925-                                                               TGAAACTAGGAAGACCCTTTGGTCCATCTTTTGATAAACAGAATCCATCATACATGTCTACCCCAGTTGGAAGTATCTCTGCAATGACTGGAAAGTAAAGAGGACCAAGGTGAAAATAAAGGCTCGGAGGGGAGCAATCTTGAAAACATGTC
ST-E00192:51:H2KG3CCXX:2:1208:30090:35045-                                                                 AACTAGGAAGACCCTTTGGTCCATCTTTTGATAAACAGAATCCATCATACATGTCTACCCCAGTTGGAAGTATCTCTGCAATGACTGGAAAGTAAAGAGGACCAAGGTGAAAATAAAGGCTCGGAGGGGAGCAATCTTGAAAACATGTCATC
ST-E00192:51:H2KG3CCXX:2:1208:30353:35502-                                                                 AACTAGGAAGACCCTTTGGTCCATCTTTTGATAAACAGAATCCATCATACATGTCTACCCCAGTTGGAAGTATCTCTGCAATGACTGGAAAGTAAAGAGGACCAAGGTGAAAATAAAGGCTCGGAGGGGAGCAATCTTGAAAACATGTCatC
```

chr3 53739293 - 0 chr8 33049742 + 6 CTX 58 18 0 43 0.885009009009009 0 0.992619047619048 0 1 chr3 53739335 148 chr8 33049846

GTTTGTGCCATTTCCCCAGCTTGAAAAAGCCCCCACCCCCAACCTCTGCCGCCCAGGCTGGAGTGCAGTGGTGCGATCTTGGCTCACTTCAACCTCTGCCTCCTGAGTTCAAGCAATTCTCCTGCCTCAGCCTCCCTAGAAGCTGGGAGG

```
                                    53739160          53739180          53739200          53739220          53739240          53739260          53739280          53739300          53739320          53739340          53739360          53739380          53739400          53739420          53739440
chr3:53739145-53739442              GCTTAGTGTGAGAAGCTGATCCCATTGTTAAGGCCCAGCACTCTGCTGTGTGAGCCCAGGAGGGCGCGATGATTCTGGCTGGTGGGAGTGGGCAGGCTGGGGCGATGTGAGGAAGGCTCCGTGGGAGGAGCAGCCCTGGGCGGCAGAGGTTGGGGGTGGGGGCTTTTTCAAGCTGGGGAAATGGCACAAACAAGGTGTGCGAAGTGACAAATATTCAGGGCCCCTTAAGTGCATTGCTCAGTAATATAATCTAGGAAAGAAACAAAACCTGTTTAAAAGCCTTGTAGATCTTCAGAATT

ST-E00144:68:H2LKCCXX:8:2209:7343:2575  +  TTAGTGTGAGAAGCTGATCCCATTGTTAAGGCCCAGCACTCTGCTGTGTGAGCCCAGGAGGGCGCGATGATTCTGGCTGGTGGGAGTGGGCAGGCTGGGGCGAtGTGAGGAAGGCTCCGTGGGAGGAGCAGCCCTGGGCGGCaGAGGtgG
ST-E00144:68:H2LKCCXX:3:1208:8734:4069  +           TGATCCCATTGTTAAGGCCCAGCACTCTGCTGTGTGAGCCCAGGAGGGCGCGATGATTCTGGCTGGTGGaAGTGGGCAGGCTGGGGCGATGTGAGGAAGGCTCCGTGGGaGGAGCcGCCCTGGGCGGCAgaGGttGGgGGtGGGGGC
ST-E00144:68:H2LKCCXX:3:1208:8978:4209  +           TGATCCCATTGTTAAGGCCCAGCACTCTGCTGTGTGAGCCCAGGAGGGCGCGATGATTCTGGCTGGTGGGAGTGGGCAGGCTGGGGCGATGTGAGGAAGGCTCCGTGGGAGGAGCAGCCCTGGGCGGCAGAGGTTGGGGGTGGGGGCtTT
ST-E00144:68:H2LKCCXX:1:1213:5466:5335  +              CCcATTGTTAAGGCCCAGCACTCTGCTGTGTGAGCCCAGGAGGGCGCGATGATTCTGGCTGGTGGGAGTGGGCAGGCTGGGGCGATGTGAGGAAGGCTCCGTGGGAGGAGCAGCgcGGGcGGCaGAGGctGGGGGcGGGGGcgcgggca
ST-E00144:68:H2LKCCXX:4:2101:3649:4157  +                AtTGTTAAGGCCCAGCACTCTGCTGTGTGAGCCCAGGAGGGCgCGATGATTCtGgCtgGtgGAGtGgGCAGGCtGGggcgATGTGAGGAAGGctCcGtGGGAgGaGCcgccgTggGCGgCAGaGGtcGGGGGGggGggctttTcgA
ST-E00144:68:H2LKCCXX:1:1102:28293:54225+                   TAAGGCCCAGCACTCTGCTGTGTGAGCCCAGGAGGGCGCGATGATTCTGGCTGGTGGGAGTGGGCAGGCTGGGGCGATGTGAGGAAGGCTCCGTGGGAGGAGCAGCCCTGGGCGGCAGAGGTTGGGGGTGGGGGCTtttcAAGcTGGGG
ST-E00144:68:H2LKCCXX:8:1209:19980:61275+                      AGCACTCTGCTGTGTGAGCCCAGGAGGGCGCGATGATTCTGGCTGGTGGGAGTGGGCAGGCTGGGGCGATGTGAGGAAGGCTCCGTGGGAGGAGCAGCCCTGGGCGGCAGAGGTTGGGGGTGGGGGCTTTTTCAAGCTGGGGAAATGGCA
ST-E00144:68:H2LKCCXX:6:1218:7963:23196 +                          GTGAGCCCAGGAGGGCGCGATGATTCTGGCTGGTGGGAGtGGGCAGGCTGGGGCGATGTGAGGAAGGCTCCGTGGGAGGAGCAGCCCTGGGCGGCAGAGGTTGGGGGtGGGGGCTTTTTCAAGCTGGGGAAATGGCACAAACAAGGTGTG
ST-E00144:68:H2LKCCXX:8:1124:2685:21649 +                          GAGCCCAGGAGGGCGCGATGATTCTGGCTGGTGGGAGTGGGCAGGCTGGGGCAtGtGaGGAAGGCTCCGTGGGAGGAGCAGCCCtGGGCGGCAGAGGgtGGGGGcGGGGGCTTTTTCAAGCtgGGgaAatGGCACAAccAaGGtGCG
ST-E00144:68:H2LKCCXX:7:1210:12104:7515 +                           GCCCAGGAGGGCGCGATGATTCTGGCTGGTGGGAGTGGGCAGGCTGGGGCGATGTGAGGAAGGCTCCGTGGGAGGAGCAGCCCTGGGCGGCAGAGGgTGGGGGTGGGGGCTgttCAAGCtGGGGAAATGGCACAAACAAGGtGTGCGAA
ST-E00144:68:H2LKCCXX:8:2215:28750:11453+                            CCCAGGAGGGCGCGATGATTCTGGCTGGTGGGAGTGGGCAGGCTGGGGCGATGTGAGGAAGGCTCCGTGGGAGGAGCAGCCCTGGGCGGCAGAGGTtGGGGGTGGGGGCtttTTCAAGCTGGGGAAATGGCACAAACAAGGTGTGCGAAG
ST-E00144:68:H2LKCCXX:1:1206:21675:44064+                            CCAGGAGGGCGCGATGATTCTGGCTGGTGGGAGTGGGCAGGCTGGGGCGATGTGAGGAAGGCTCCGTGGGAGGAGCAGCCCTGGGCGGCAGAGGTTGGGGGTGGGGGCTTTTTCAAGCTGGGGAAATGGCACAAACAAGGTGTGCGAAGT
ST-E00144:68:H2LKCCXX:3:2211:21432:36540+                             CcGGAGGGCGCGATGATTcTGGCTGGtggGAGcggGCAGGCTgggGCGATGtGAGGAAGGCTCCGTGgaGGAGCaGccCTGGGCGGCAGcGGTTGgGGgtgGGGGCtttTTcAagCtGGGgaActggCAcAAACAaGGtGtGCGaAGTG
ST-E00144:68:H2LKCCXX:4:2204:12337:46560+                               GGAGGGCGCGATGATTCTGGCTGGTGGGAGTGGGCAGGCTGGGGCGATGTGAGGAAGGCTCCGTGGGAGGAGCAGCCCTGGGCGGCAGAGGTTGGGGGTGGGGGCTTTTTCAAGCTGGGGAAATGGCACAAACAAGGTGTGCGAAGTGAC
ST-E00144:68:H2LKCCXX:1:1115:17747:44046+                               GAGGGCGCGATGATTCTGGCTGGTGGGAGTGGGCAGGCTGGGGCGATGTGAGGAAGGCTCCGTGGGAGGAGCAGCCCTGGGCGGCAGAGGTTGGGGGTGGGGGCTTTTTCAAGCTGGGGAAATGGCACAAACAAGGTGTGCGAAGtgACA
ST-E00144:68:H2LKCCXX:8:2202:5943:48037 +                                AGGGCGCGATGATTCTGGCTGGTGGGAGTGGGCAGGCTGGGGCGATGTGAGGAAGGCTCCGTGGGAGGAGCAGCCCTGGGCGGCAGAGGTTGGGGGgGGGGGCTTTTTCAAGCTGGGGAAATGGCACAAACAAGGTGTGCGAAGTGaCAA
ST-E00144:68:H2LKCCXX:3:1123:3852:18221 +                                      GCGATGATTCTGGCTGGTGGGAGtGGGCAGGCTGGGGCGATGTGAGGAAGGCTCCGTGGGAGGaGCAGCCCtGGGCGGCAGAGGTGGGGGtgGGGGCttTTTCAAGCTgGGGGAAATGGCACAaACAAGGTGTGCGAAGTGACAAgATT
ST-E00144:68:H2LKCCXX:3:1124:18346:30703+                                      GCGATGATTCTGGCTGGTGGGAGTGGGCAGGCTGGGGCGATGTGAGGAAGGCTCCGTGGGAGGAGCAGCCCTGGGCGGCAGAGGTTGGGGGTGGGGGCTTTTTCAAGCTGGGGAAATGGCACAAACAAGGTGTGCGAAGTGACAAATATT
ST-E00144:68:H2LKCCXX:7:2113:22538:29560+                                       GgTGATTCTGGCtGGtGGGaGTGGGCAGGCTGGGGCGATGTGAGGaAGGctCCgtGgGGAGGAGCAGcCctGgGCGGCAGAGGTTGGGGGgGgGGCcttTTcAaGCtGGGGAAAtGGCAgAaacaAGgGtgCGaagtGaCaaatataca
ST-E00144:68:H2LKCCXX:1:2223:17534:20155+                                             CTGGCTGGTGGGAGTGGGCAGGCTGGGGCGATGTGAGGAAGGCTCCGTGGGAGGAGCAGCCCTGGGCGGCAgaGGTTGGGGGTGGGGGCTTTTTCAAGCTGGGGAAATGGCACAAACAAGGTGtgcGAAGtGACAAATATTCAGGGCCCT
ST-E00144:68:H2LKCCXX:8:2115:17128:17377+                                                           GGCGATGTGAGGAAGGCTCCGTGGGAGGAGCAGCCCTGGGCGGCAGAGGTTGGGGGTGGGGGCTTTTTCAAGCTGGGGAAATGGCACAAACAAGGTGTGCGAAGTGACAAATATTCAGGGCCCCTTAAGTGCATTGCTCAGTAATATAATC
ST-E00144:68:H2LKCCXX:1:1104:3202:39704 +                                                                  AGGCTCCGTcGgAGGAGCAGCCCTgGCGGaAgAGGCTGGGGGtGGGGGCTTTtTCAaGCTGGGcAAAtGgcaCAAACAAGGTGcgCGAAGTGAcAaATATTCAtggCCCtTAAGTGCATtgctCAGTAATAtAAtCtAGGAAgAAaCA
ST-E00144:68:H2LKCCXX:4:1123:27745:46139+                                                                  AGGCTCCGTGGGAGGAGCAGCCCTGGGCGGCAGAGGtTGGGGGTGGGGGCTTTTTCAAGCTGGGGAAATGGCACAAACAAGGTGTGCGAAGTGACAAATATTCAGGGCCCCTTAAGTGCATTGCTCAGTAATATAATCTAGGAAAGAAACA
ST-E00144:68:H2LKCCXX:8:1111:2999:39458 +                                                                  AGGCTCCGTGGGAGGAGCAGCCCTGGGCGGCAGAGGTTGGGGGTGGGGGCTTTTTCAAGCTGGGGAAATGGCACAAACAAGGTGTGCGAAGTGACAAAGATTCAGGGCCCCTTAAGTGCATtgCtcaGtAAtATAATCTAGGAAAGAAACA
ST-E00144:68:H2LKCCXX:8:1111:3192:39546 +                                                                  AGGCTCCGTGGGAGGAGCAGCCCTGGGCGGCAGAGGTTGGGGGTGGGGGCTTTTTCAAGCTGGGGAAATGGCACAAACAAGGTGTGCGAAGTGACAAAtATTCAGGGCCCCTTAAGTGCATTGCTCAGTAATATAATCTAGGAAAGAAAga
ST-E00144:68:H2LKCCXX:1:2115:10277:22071+                                                                   GGCTCCGTGGGAGGAGCAGCCCTGGGCGGCAGAGGTTGGGGGTGGGGGCTTTTTCAAGCAGGGcAATTGGCACAAACAAGGTGTGCGAAGTGACAAATATTCAGGGCtCTTAAGTGCATTGCTCAGTAATATAATCTAGGaAAGAAACAA
ST-E00144:68:H2LKCCXX:4:2120:5141:69116 +                                                                      CCGTGGGAGGAGCAGCCCTGGGCGGCAGAGGTTGGGGGTGGGGGCTTTTTCAAGCTGGGGAAATGGCACAAACAAGGTGTGCGAAGTGACAAATATTCAGGGCCCCTTAAGTGCATTGCTCAGTAATATAATCTAGGAAAGAAACAAAACC
ST-E00144:68:H2LKCCXX:1:1106:3243:65969 +                                                                        GTGGGAGGAGCAGCCCTGGGCGGCAGAGGTTGGGGGTGGGGGCTTTTTCAAGCTGGGGAAATGGCACAaaCAAGGgGTGCGAAGTGACAAATATTCAGGGCCCCTTAAGTgcATTGCTCAGTAATATAATCgAGGaAAGacACAAAACCTG
ST-E00144:68:H2LKCCXX:1:1106:3192:65987 +                                                                        GTGGGAGGAGCAGCCCTGGGCGGCAGAGGTTGGGGGTGGGGGCTTTTTCAAGCTGGGGAAATGGCACAAACAAGGTGTGCGAAGTGACAAATAgtcAGGGCCCTgAAGTGCATTGCTcAGTAATATAATCTAGGAAAGAAACAAAACCTG
ST-E00144:68:H2LKCCXX:1:2116:12358:52432+                                                                           GGAGCAGCCCTGGGCGGCAGAGGTTGGGGGTGGGGGCTTTTTCAAGCTGGGGAAATGGCACAAACAAGGTGTGCGAAGTGACAAATATTCAGGGCCCCTTAAGTGCATTGCTCAGTAATATAATCTAGGAAAGAAACAAAACCTGTTTAA
ST-E00144:68:H2LKCCXX:8:1209:17189:64738+                                                                            GGAGCAGCCCTGGGCGGCAGAGGTTGGGGGTGGGGGCTTTTTCAAGCTGGGGAAATGGCACAAACAAGGTGTGCGAAGTGACAAATATTCAGGGCCCCTTAAGTGCATTGCTCAGTAATATAATCTAGGAAAGAAACAAAACCTGTTTAAA
ST-E00144:68:H2LKCCXX:3:1107:25461:43589+                                                                                     AGAGGTTGGGGGTGGGGGCTTTTTCAAGCTGGGGAAATGGCACAAACAAGGTGTGCGAAGTGACAAATATTCAGGGCCCCtTAAGTGCATTGCTCAGTAATATAATCTAGGAAAGAAACAAAACCTGTTTAAAAGCCTTGTAGATCTTCAG
ST-E00144:68:H2LKCCXX:3:1107:25481:43625+                                                                                     AGAGGTTGGGGGTGGGGGCTTTTTCAAGCTGGGGAAATGGCACAAACAAGGTGTGCGAAGTGACAAATATTCAGGGCCCCTTAAGTGCATTGCTCAGTAATATAATCTAGGAAAGAAACAAAACCTGTTTAAAAGCCTTGTAGATCTTCAG
ST-E00144:68:H2LKCCXX:6:2103:25289:31828+                                                                                      AGGTTGGGGGTGGGGGCTTTTTCAAGCTGGGGAAATGGCACAAACAaGGTGTgCGAAGTGACAAATATTCAGGGCCCCTTAAGTGCATTGCTCAgTAATATAATCTAGGAAAGAAACAAAACCTGTTTAAAAGcctTGTAgatCTTCAGaA
ST-E00144:68:H2LKCCXX:6:2103:25004:32039+                                                                                      AGGTTGGGGGTGGGGGCTTTTTCAAagcTGtggtAATGGCACAAACAAGGTGTGCGAAGTGACAAATATTCAGGGCCCCTTAAGTGCATTGCTCAGTAATATAATCTAGGaAAGAAACAAAACCTGgTTAAAAGcCTTGTAGATCTTCAGAA
ST-E00144:68:H2LKCCXX:7:2211:28070:20647-      tAGAAGCTGATCCCATTGTTAAGGCCCAGCACTCTGCTGTGTGAGCCCAGGAGGGCGCGATGATTCTGGCTGGTGGGAGTGGGCAGGCTGGGGCGATGTGAGGAAGGCTCCGTGGGAGGAGCAGCCCTGGGCGGCAGAGGTTGGGGGTGG
ST-E00144:68:H2LKCCXX:6:2210:26750:59464-              CATTGttacGGCCCAGCacTCTGCTGTGTGaGCCcagGAGGGCGCGATGAtTCgGGCTGGTGGGGgTGGGCAGGCTGGGGCGAtGTGAGGAAGGCTCCGTGGGAGgAGCAGCCCtGgCGGCAGAGGTTGGGGGTGGGGGCTTTtTCaAG
ST-E00144:68:H2LKCCXX:3:2102:3994:32602 -                TTaaGgcccAGCcctCTGgTGTGtGAGCCCAGGAGGGCGCGATGATTCTGgCTGGTGGGAGTGGGCAGGcTGGGGCGATGTGAGGAAGGCTCCGTGGGAGGAGCAGCCCtGGGCGGCAGAGGTTGGGGGTGGGGGCTTTTTCAAGCTGGG
ST-E00144:68:H2LKCCXX:3:2122:18021:61469-                 TAAGGCCCAGCACTCTGCtGTGTGAGcCCAgGAGGGCGCGATGATTCTGGCTGGTGGGAGTGGGCAGGCTGGGGCGATGTgGGAAGGCTCCGTGGGAGGAGCAGCCCTGGGCGGCAGAGGTTGGGGGTGGGGGCTTTTTCAAGCTGGGG
ST-E00144:68:H2LKCCXX:6:1213:25471:63965-                 TAAGGCCCAGCACTCTGCTGTGTGAGCCCAGGAGGGCGCGATGATTCTGGCTGGTGGGAGTGGGCAGGCTGGGGCGATGTGAGGAAGGCTCCGTGGGAGGAGCAGCCCTGGGCGGCAGAGGTTGGGGGTGGGGGCTTTTTCAAGCTGGGG
ST-E00144:68:H2LKCCXX:3:1103:11322:5598 -                  AagGCCCAGCACTCTGCtGTGTGAgCCCAGGAGGGCGCGATGATtCTGGCTGGTGGGAgTGGGCAGGCTGGGGCGATGTGAGGAAGGCtCCGTGGGAGGAGCAGCCCTGGGCGGCAGAGGTTGGGGGTGGGGGCTTTTTCAAGCTGGGGA
ST-E00144:68:H2LKCCXX:8:1217:3202:36223 -                  aaGGCCcaGCactCTGCtgtGtGaGcCCAGGAGGGCGCGATGATTCTGGCTGGTGGGAGTGGGCAGGcAGGCTGGGGCGATGTGAGGAAGGCTCcGTGGGAGGAGCAGCCCTGGGCGGCAGAGGTTGGGGGTGGGGGCTTTTTCAAGCTGGGGA
ST-E00144:68:H2LKCCXX:1:1116:9323:10398 -                    CCaGCACtcTGCtGtGtGgGCCCAGGaGGGCGCGATGATTCTGGCTGGTGGGAgTGGGCAGGcTGGGGCGATGtGAGGAAGGCTCCGTGGGAGGAGCAGCCCTGGGCGGCAGAGGTTGGGGGTGGGGGCTTTTTCAAGCTGGGGAAATGG
ST-E00144:68:H2LKCCXX:1:1109:23797:16199-                        tctGCTgtgtgaGccccGgaGGGCgcgaTGAtTCTGGCgGGTGGGAGTGGGCAGGCTgGGGCGATGTGAGGAAGGCTCCGTGGGAGGAGCAGCCCTGGGCGGCAGAGGTTGGGGGTGGGGGCTTTTTCAAGCTGGGGAAATGGCACAAAC
ST-E00144:68:H2LKCCXX:1:2105:27156:40618-                         CgGtGgGagcCCAGGaGGGCGCGATGATTCgGGCTGGTGGGAGTGGGCAGGCtGGGGCGATGTGAGGAAGgCtCCGTGGGAGGAGCAGCCCTGGGCGGCAGAGGTTGGGGGTGGGGGCTTTTTCAAGCTGGGGAAATGGCACAAACAAGG
ST-E00144:68:H2LKCCXX:6:1222:23218:63895-                          TGTGagcCcaGGaGGGCGCGATGAttCTGGCTGGTGGGAGTgGGCAGGCTGGGGCGATGTGAGGAAGGCTCCGTGGGAGGAGCAGCCCTGGGCGGCAGAGGTTGGGGGTGGGGGCTTTTTCAAGCTGGGGAAATGGCACAAACAAGGTGT
ST-E00144:68:H2LKCCXX:1:2219:32536:14951-                          GTgagccCaGgagGGCgCGCGATGATTCTGGCTGGTGGGAGTGGGCAGGCTGGGGCGATGTGgGAAGGCTCCGTGGGAGGAGCAGCCCTGGGCGGCAGAGGTTGGGGGTGGGGGCTTTTTCAAGCTGGGGAAATGGCACAAACAAGGTG
ST-E00144:68:H2LKCCXX:8:2108:16225:32795-                          GtGaGCccAGGgGGGCGCgatgatTCtGGtGGTGGGAGTGGGcaGGCtGGGGCGATGTGAGGAtGGCTCCGTGGGAGGAGCAGCCCTGGGCGGCAGAGGTTGGGGgTGGGgGaTTtTTTCAAGCTGGGGAAAtGGCACaAACAaGGTGTG
ST-E00144:68:H2LKCCXX:6:1108:25157:24954-                                       cTGGtGGGaGgGGGcAGGCtGGGGCGATGTGAGGAAGGCTCCGTGGGAGGAGCAGCCCTGGGCGGCAGAGGTTGGGGGTGGGGGCTTTTTCAAGCTGGGGAAATGGCACAAACAAGGTGTGCGAAGTGACAAATATTCAGGGCCCCTTAAG
ST-E00144:68:H2LKCCXX:4:1218:30638:15320-                                       TGGTGGGaGgGGcAGGctGGGGcgaTGtGAGGAAGGCTcCGTGGGAGGAGCAGCcCtGGgCGGCAGAGGTTGGGGGTGGGGGCTTTTTCAAGCTGGGGAAATGGCACAAACAAGGTGTGCGAAGTGACAAATATTCAGGGCCCCTTaAGT
ST-E00144:68:H2LKCCXX:8:2107:29988:35960-                                            GtGGGcgGGcTGGGGCgATGtGAGGAAGGCTCCGTGGGAGGAGCAGCCCTGGGCGGCAGAGGTTGGGGGTGGGGGCTTTTTCAAGCTGGGGAAATGGCACAAACAAGGTGTGCGAAGTGACAAATATTCAGGGCCCCTTAAGTGCATTGCT
ST-E00144:68:H2LKCCXX:6:2102:22538:48195-                                             gGGgtGGGGCGATGTGAGGaAGgctCAGTGGGAGgaGCAgcCCTGGGCGGCAGAGGTTGGGGGTGGGGGCTTTTTCAAGCTGGGGAAATGGCACAAACAAGGTGTGCGAAGTGACAAATATTCAGGGCCCCTTAAGTGCATTGCTCAGTAA
ST-E00144:68:H2LKCCXX:8:2213:9881:64387 -                                               TGgGgcgATGtGaGgaggGcgcggTgGaGGAGcAGcCTGgGcGGCAGAGGTTGGGGGTGgGGGCTTTTTCAAGCTGGGGAAATGGCACAAACAAGGTGTGCGAAGTGACAAATATTCAGGGCCCCTTAAGTGCATTGCTCAGTAATATA
ST-E00144:68:H2LKCCXX:1:2103:7973:18326 -                                                    TGTGAGGAAGgCTccGtGGGAGGAGCAGCCCTGgtCGGCAGAGGTTGGGGGTGGGGGCTTTTTCAAGCTGGGGAAATGGCACAAACAAGGTGTGCGAAGTGACAAATATTCAGGGCCCCTTAAGTGCATTGCTCAGTAATATAATCTAGGA
ST-E00144:68:H2LKCCXX:3:2124:12845:63578-                                                    TGTGAGGaAGGctCCGTGGGAGGAGCAGCCCTGGGCGGCAGAGGTtGGGGGTGGGGGCTTTTTCAAGCTGGGGAAATGGCACAAACAAGGTGTGCGAAGTGACAAATATTCAGGGCCCCTTAAGTGCATTGCTCAGTAATATAATCTAGGA
ST-E00144:68:H2LKCCXX:7:1210:12104:7515 -                                                      GAAGGCTCCGTGGGAGGAGCAGcCCTGgGCGGCAGAGGTtGGGGGTGGGGGCTTTTTCAAGCTGGGGAAATGGCACAAACAAGGTGTGCGAAGTGACAaATATTCAGGGCCCCTTAAGTGCATTGCTCAGTAATATAATCTAGGAAAGAAA
ST-E00144:68:H2LKCCXX:6:1219:4968:36329 -                                                       AAGGcTCcGgGGGAGGAgCAGCCctGGGCGGCaGAGGTTGGGGGTGGGGGCTTTTTCAAGCTGGGGAAATGGCACAAACAAGGTGTGCGAAGTGACAAATATTCAGGGCCCCTTAAGTGCATTGCTCAGTAATATAATCTAGGAAAGAAAC
ST-E00144:68:H2LKCCXX:6:2205:32586:11312-                                  gaGggtgggacgatgtgtGgtagGgtgcgTgGGAGGAGCgGCCctgggcGggAGagGTTGgGGgtGgGGgCtTTTTCAAGCTGGGGaaATGGCACAAACAAGGTGTGCGAAGTGACaaATATTCaGGGCCCCTTAAGTGCATTGCTCAGTA
ST-E00144:68:H2LKCCXX:7:1116:17189:68044-                                                           CGTGGGAGGAGCAGCCCTGGGCGGCAGAGGTTGGGGGTGGGGGCTTTTTCAAGCTGGGGAAATGGCACAAACAAGGTGTGCGAAGTGACAAATATTCAGGGCCCCTTAAGTGCATTGCTCAGTAATATAATCTAGGAAAGAAACAAAACCT
ST-E00144:68:H2LKCCXX:7:1116:18062:67165-                                                            GTGGGAgGAGCAGCCCTGGGCGGCAGAGGTTGGGGGTGGGGGCTTTTTCAAGCTGGGGAAATGGCACAAACAAGGTGTGCGAAGTGACAAATATTCAGGGCCCCTTAAGTGCATTGCTCAGTAATATAATCTAGGAAAGAAACAAAACCT
ST-E00144:68:H2LKCCXX:4:1204:27938:28857-                                                                      TGGgGGCAGAGGTtGGGGGTGGGGGCTTTTTCAAGCTGGGGAAATGGCACAAACAAGGTGTGCGAAGTGACAAATATTCAGGGCCCCTTAAGTGCATTGCTCAGTAATATAATCTAGGAAAGAAACAAAACCTGTTTAAAAGCCTTGTAG
ST-E00144:68:H2LKCCXX:7:2221:24081:12964-                                                                      GgGCGGCAGAGGTTGGGGtGGgcGCttttTCaCGCtgGGGaaatgGCACcaACAAggtGcGCGAaGTGACaAATATTCAGGGCCCCTTAAGTGCATTGCTCAGgAATATAaTCtaGGAAAGAAaCAAAACCTGTTTAAAAGCCTTGtAG
ST-E00144:68:H2LKCCXX:8:1223:27217:43888-                                                  gcgGtGaggaaGcCgaCGGGGggggaGgggggcAGgGggggagaGgttGgGGGtGGGGGcgttgcAAGctgGgaaATggcACAAACAAGGtGTGCGaAGtgacaAatagTCAGGGCCctTgaGgggatggctcgTAATATaatCTAG
ST-E00144:68:H2LKCCXX:1:2204:32556:67692-                                                                        GGGGttgGgGTtggggAttTttcAAGCTgGGgaAtgGcacAaaCAcggtGTGCGAAGTGACAAAtattCaGGGCCCCTTAaGtGCATTGCTCAGtAATATAAtCtaGGAaGAAACAAAACCTGTTTAAAaGcCTTGTA
ST-E00144:68:H2LKCCXX:6:1107:19107:12191-                                                                           gTTgGgGGTGGGGGCTTTTTCAAGCTGGGGAAATGGCACAAACAAGGTGTGCGAAGTGACAAATATTCAGGGCCCCTTAAGTGCATTGCTCAGTAATATAATCTAGGAAAGAAACAAAACCTGTTTAAAAGCCTTGTAGATCTTCAGAATT

ST-E00192:51:H2KG3CCXX:5:1218:17514:57917+     GTGTGAGAAGCTGATCCCATTGTTAAGGCCCAGCACTCTGCTGTGTGAGCCCAGGAGGGCGCGATGATTCTGGCTGGTGGGAGTGGGCAGGCTGGGGCGATGTGAGGAAGGCTCCGTGGGAGGAGCAGCCCTGGGCGGCaGaGGttGGG
ST-E00192:51:H2KG3CCXX:5:2119:17899:53628+         aAGCTGATCCCATTGTTAAGGCCCAGCACTCTGCTGTGTGAGCCCAGGAGGGCGCGATGATTCTGGCTGGtGGGaGTGGGCAGGCTGGGGCGATGTGAGGAAGGCTCCGTGGGAGGAGCAGCCcTGGGCGGCAGAGGttGGGGGtGGGGG
ST-E00192:51:H2KG3CCXX:5:1222:10449:50323+            GATCCCATTGTTAAGGCCCAGCACTCTGCTGTGTGAGCCCAGGAGGGCGCGATGATTCTGGCTGGTGGGAGTGgGCAGGCTGGGGCGAtGTGAGGAAGGCTCCGTGGGAGGAGCAGCCCTGgGCGGCaGAGGTTGGGGGTGGGGGgTtT
ST-E00192:51:H2KG3CCXX:2:2203:17372:2961 +             CCATTGTTAAGGCCCAGCACTCTGCTGTGTGAGCCCAGGAGGGCGCGATGATtCTGGCTGGTGGGAGTGGGCAGGCTGGGGCGAtGTGAGGAAGGCTCCGTGGGAGGAGCAGCCCTGGGCGGCAGAGGTTGGGGGTGGGGGCttTTTCaA
ST-E00192:51:H2KG3CCXX:6:2214:23726:43255+                AAGGCCCAGCACTCTGCTGTGTGAGCCCAGGAGGGCGCGATGATTCTGGCTGGTGGGAGTGGGCAGGCTGGGGCGaTGTGAGGAAGGCTCCGTGGGAGGAGCAGCCCTGGGCGGCAGAGGTTGGGGGTGGGGGCTggTTCAAGctGGGGA
ST-E00192:51:H2KG3CCXX:5:1223:9201:31424 +                      GCTGTGTGAGCCCAGGAGGGCGCGATGATTCTGGCTGGTGGGAGTGGGCAGGCTGGGGCGATGTGAGGAAGGCTCCGtGGGAGGAGCAGCCCTGGGCGGCAGAGGTTGGGGGTGGGGGCtTTTTcAGCTGGGGAAATGGCACAAACAAG
ST-E00192:51:H2KG3CCXX:3:1116:23543:32988+                              GGCGCGATGATTCTGGCaGGTGGGAGTGGGCAGGCTGGGGCGATGTGAGGAAGGCTCcGtGGGAGGAGCAGCCcTGGGcGGCagaGGtTGGGGGtGGGGGcTTtTTCAAGCtGGGGAAATGGCACAAACaaGgTGTGgGaagtGAcAaat
ST-E00192:51:H2KG3CCXX:6:2117:9587:53786 +                               GCGCGAtGATTCTGGctGGtGGGAGtGGGcAGGcTGGGGCGAagtGAGGAAGGCTCCGTGGGAGGAGcAGCCCTGGGCggCGAGGTTGGGGgTGGGGGCTTTTTCAAGcTGGGGAAATGGCACAAACAAGGTGtGCGAAGTGACAaATA
ST-E00192:51:H2KG3CCXX:6:2117:9627:53891 +                               GCGCGATGATTCTGGCTGGTGGGAGTGGGCAGGCTGGGGCGATGTGAGGAAGGCTCCGTGGGAGGAGCAGCCCTGGGCGGCAGAGGTTGGGGGTGGGGGCtTTTTCAAGCTGGGGAAAtgGCACAAACAAGGTGTGCGAAGTGACAAATA
ST-E00192:51:H2KG3CCXX:1:2209:28029:47475+                                 GATGATTCTGGCTGGTGGGAGTGGGCAGGCTGGGGCGATGTGAGGAAGGCTCCGTGGGAGGAGCAGCCCTGGGCGGCAGAGGTTGGGGGTGGGGGCTTTTTCAAGCTGGGGAAATGGCACAAACAAGGTGTGCGAAGTGACAAATATTCA
ST-E00192:51:H2KG3CCXX:8:1123:2238:69960 +                                  GATTCTGGCTGGTGGGAGTGGGCAGGCTGGGGCGATGTGAGGAAGGCTCCGTGGGAGGAGCAGcCCTGGGCGGCAgAGGttGGGGGTGGGGGCttTTTCAAGCtGGGgAAgtGGcacaAAcaaGGgTgCGaagtGaCAgctattcAGgG
ST-E00192:51:H2KG3CCXX:5:1123:11606:43466+                                        CTGGTGGGAGTGGGCAGGCTGGGGCGATGTGAGGAAGGCTCCGTGGGAGGAGCAGCCCTGGGCGGCAGAGGTTGGGGGTGGGGGCTTTTTCAAGCTGGGGAAATGGCACAAACAAGGTGTGCGAAGTGACAAATATTCAGGGCCCCTTAAG
ST-E00192:51:H2KG3CCXX:3:2110:6105:57355 +                                          GGGAGTGGGCAGGCTGGGGCGATGTGAGGAAGGCTCCGTGGGAGGAGCAGCCCTGGGCGGCAGAGGTTGGGGGTGGGGGCTTTTTCAAGCTGGGGAAATGGCACAAACAAGGTGTGCGAAGTGACAAATATTCAGGGCCCCTTAAGTGCAT
ST-E00192:51:H2KG3CCXX:6:1103:4451:35432 +                                                GGGGCGATGTGAGGAAGGCTCCGTGGGAGGAGCAGCCCTGGGCGGCAGAGGTTGGGGGTGGGGGCTTTTTCAAGCTGGGGAAATGGCACAAACAAGGTGTGCGAAGTGACAAATATTCAGGGCCCCTTAAGTGCATTGCTCAGTAATATAA
ST-E00192:51:H2KG3CCXX:2:2219:26872:13298+                                                            GGCTCCGTGGGAGGAGCAGCCCTGGGCGGCAGAGGTTGGGGGTGGGGGCTTTTTCAAGCTGGGGAAATGGCACAAACAAGGTGTGCGAAGTGACAAATATTCAGGGCCCCTTAAGTGCATTGCTCAGTAATATAATCTAGGAAAGAAACAA
ST-E00192:51:H2KG3CCXX:1:1212:6927:22458 +                                                              TCCGTGGGAGGAGCAGCCCTGGGCGGCAGAGGTTGGGGGTGGGGGCTTTTTCAAGCTGGGGAAATGGCACAAACAAGGTGTGCGAAGTGACAAATATTCAGGGCCCCTTAAGTGCATTGCTCAGTAATATAATCTAGGAAAGAAACAAAAC
ST-E00192:51:H2KG3CCXX:6:1109:30130:35960+                                                                GTGGGAGGAGCAGCCCTGGGCGGCAGAGGTTGGGGGTGGGGGCTTTTTCAAGCTGGGGAAATGGCACAAACAAGGTGTGCGAAGTGACAAATATTCAGGGCCCCTTAAGTGCATTGCTCAGTAATATAATCTAGGAAAGAAACAAAACCTG
ST-E00192:51:H2KG3CCXX:8:2101:18894:15197+                                                                GTGGGAGGAGCAGCCCTGGGCGGCAGAGGTTGGGGGTGGGGGCTTTTTCAAGCTGGGGAAATGGCACAAACAAGGTGTGCGAAGTGACAAATATTCAGGGccCTTAAGTGCATTGCTCAGTaATATAATCTAGGAAAGAAACAAaACCTG
ST-E00192:51:H2KG3CCXX:3:1206:23025:42183+                                                                     AGCCCTGGGCGGCAGAGGTTGGGGGTGGGGGCTTTTTCAAGCTGGGGAAATGGCACAAACAAGGTGTGCGAAGTGACAAATATTCAGGGCCCCTTAAGTGCATTGCTCAGTAATATAATCTAGGAAAGAAACAAAACCTGTTTAAAAGCCT
ST-E00192:51:H2KG3CCXX:1:1207:32323:58884+                                                                             CCCTGGGCGGCAGAGGTTGGGGGTGGGGGCTTTTTCAAGCTGGGGAAATGGCACAAACAAGGTGTGCGAAGTGACAAATATTCAGGGCCCCTTAAGTGCATTGCTCAGTAATATAATCTAGGAAAGAAACAAAACCtGTTTAAAAGCCTTG
[illegible]
```

```
ST-E00192:51:H2KG3CCXX:3:2111:3162:16076 +  AGAGGTTGGGGGTGGGGGCTTTTTCAAGCTGGGGAAATGGCACAAACAAGGTGTGCGAAGTGACAAATATTCAGGGCCCCTTAAGTGCATTGCTCAGTAATATAATCTAGGAAAGAAACAAAACCTGTTTAAAAGCCTTGTAGATCTTCAG
ST-E00192:51:H2KG3CCXX:6:1105:28141:67604- GCTTAGTGTGAGAAGCTGATCCCATTGTTAAGGCCCAGCACTCTGCTGTGTGAGCCCAGGAGGGCGCGATGATTCTGGCTGGTGGGAGTGGGCAGCTGGGGCGATGTGAGGAAGGCTCCGTGGGAGGAGCAGCCCTGGGCGGCAGAGGT
ST-E00192:51:H2KG3CCXX:6:1119:26547:45681- GCTTAGTGTGAGAAGCTGATCCCATTGTTAAGGCCCAGCACTCTGCTGTGTGAGCCCAGGAGGGCGCGATGATTCTGGCTGGTGGGAGTGGGCAGCTGGGGCGATGTGAGGAAGGCTCCGTGGGAGGAGCAGCCCTGGGCGGCAGAGGT
ST-E00192:51:H2KG3CCXX:1:1113:17839:13088- TGTGaGAAGCTGATCCCATTGTTAAGGCCCAGCACTCTGCTGTGTGAGCCCAGGAGGGCGCGATGATTCTGGCTGGTGGGAGTGGGCAGCTGGGGCGATGTGAGGAAGGCTCCGTGGGAGGAGCAGCCCTGGGCGGCAGAGGTTGGGGG
ST-E00192:51:H2KG3CCXX:8:2109:20366:41304- gtgagaaGCtgatcCcctttttacggCcCagccctccgcTGtGtgAGCCCAGgAGGGCGCGATGATTCTGGCTGGTGGGAGTGGGCaGGCTGGGGCGATGTGAGGAAGGCTCCGTGGGAGGAGCAGCCCTGGGCGGCAGAGGTTGGGGGT
ST-E00192:51:H2KG3CCXX:1:1111:12175:48318- GCtGatCCCATTGTTAaGGCCCAGCACTcTGCTGTGtGAGCCcAGGaGGGCGCGATGATTCTGGCTGGTGGGAGTGGGCAGCTGGGGCGATGTGAGGAAGGCTCCGTGGGAGGAGCAGCCCTGGGCGGCAGAGGTTGGGGGTGGGGGC
ST-E00192:51:H2KG3CCXX:1:1115:25045:40970- GATCCCATTGTTAaGGCCCAGCACTCTGCTGTGTGAGCCCAGGAGGGCGCGATGATTCTGGCTGGTGGGAGTGGGCAGCTGGGGCGATGTGAGGAAGGCTCCGTGGGAGGAGCAGCCCTGGGCGGCAGAGGTTGGGGGTGGGGGCTTTT
ST-E00192:51:H2KG3CCXX:1:1115:25065:40970- GAtCCCATTGTTAaGGCCCAGCACtCTGCTGTGTGAGCCCAGGAGGGCGCGATGATTCTGGCTGGTGGGAGTGGGCAGCTGGGGCGATGTGAGGAAGGCTCCGTGGGAGGAGCAGCCCTGGGCGGCAGAGGTTGGGGGTGGGGGCTTTT
ST-E00192:51:H2KG3CCXX:1:1115:25096:41023- GaGCCCATTGttaaGGCccAGCgCtCTGCTGtGTGaGCCCAGGaGGGCGCGATGAttCTGGCTGGTGGGAGTGGGCAGCTGGGGCGATGTGAGGAAGGCTCCGTGGGAGGAGCAGCCCTGGGCGGCAGAGGTTGGGGGTGGGGGCTTTT
ST-E00192:51:H2KG3CCXX:8:1107:26852:29156- GTGTGaGcCCAGGAGGGCGCGATGATTCTGGCTGGTGGGAGTGGGCAGCTGGGGCGATGTGAGGAAGGCTCCGTGGGAGGAGCAGCCCTGGGCGGCAGAGGTTGGGGGTGGGGGCTTTTTCAAGCTGGGGAAATGGCACAAACAAGGTG
ST-E00192:51:H2KG3CCXX:2:2123:7861:49180 - TGTGAGCCCAGGAGGGCGCGATGATTCTGGCTGGTGGGAGTGGGCAGCTGGGGCGATGTGAGGAAGGCTCCGTGGGAGGAGCAGCCCTGGGCGGCAGAGGTTGGGGGTGGGGGCTTTTTCAAGCTGGGGAAATGGCACAAACAAGGTGT
ST-E00192:51:H2KG3CCXX:2:1113:10419:47176- gGAgCcgAGGgGgCGCGATGAtTCTGgGTGGtGGGAGtGggggGCTGGGGCGATGTGAGGAAGGctccGTGGGAGGAGCAGCCCTGGGCGGCAGAGGTTGGGGGTGGGGGCTTTTTCAaGCTGGGGAAATGGCACAAACAAGgTGTGC
ST-E00192:51:H2KG3CCXX:2:1113:10439:47211- tGAGCCCAGgaGGGCGCGaTGATTCTGGcTGGtGGGAgTGGGCAGCtGGGGCGATGTGAGGAAGGCTCCGTGGGAGGAGCAGCCCTGGGCGGCAGAGGtTGGGGGTGGGGGCTTTTTCAAGCTGGGGAAATGGCACAAACAAGGTGTGC
ST-E00192:51:H2KG3CCXX:3:2111:30140:23601- TGAGCCCAGGaGGGCGCGAtGATTCTGGCTGGTGGGAGTGGGCAGCTGGGGCGATGTGAGGAAGGCTCCGTGGGAGGAGCAGCCCTGGGCGGCAGAGGTTGGGGGTGGGGGCTTTTTCAAGCTGGGGAAATGGCACAAACAAGGTGTGC
ST-E00192:51:H2KG3CCXX:3:2111:30364:23671- TGAGCCCAGGAGGGCGCGATGATtCTGGCTGGTGGGAGTGGGCAGCTGGGGCGATGTGAGGAAGGCTCCGTGGGAGGAGCAGCCCTGGGCGGCAGAGGTTGGGGGTGGGGGCTTTTTCAAGCTGGGGAAATGGCACAAACAAGGTGTGC
ST-E00192:51:H2KG3CCXX:5:1123:10388:30000- AGGAGGGCGCGATGaTTCTGGCTGGtGGGAGTGGGCAGCTGGGGCGATGTGAGGAAGGCTCCGTGGGAGGAGCAGCCCTGGGCGGCAGAGGTTGGGGGTGGGGGCTTTTTCAAGCTGGGGAAATGGCACAAACAAGGTGTGCGAAGTGA
ST-E00192:51:H2KG3CCXX:5:1209:21310:27257- GGCGCGATGATTCTGGCTGGTGGGAGTGGGCAGCTGGGGCGATGTGAGGAAGGCTCCGTGGGAGGAGCAGCCCTGGGCGGCAGAGGTTGGGGGTGGGGGCTTTTTCAAGCTGGGGAAATGGCACAAACAAGGTGTGCGAAGTGACAAAT
ST-E00192:51:H2KG3CCXX:6:1217:20914:36417- TtCTGGCTGGTGGGAGTGGGCAGGCTGGGGCGATGTGAGGAAGGCTCCGTGGGAGGAGCAGCCCTGGGCGGCAGAGGTTGGGGGTGGGGGCTTTTTCAAGCTGGGGAAATGGCACAAACAAGGTGTGCGAAGTGACAAATATTCAGGGCC
ST-E00192:51:H2KG3CCXX:3:2218:2471:4297  - CtGGTGGGaGTGGgCAGGCTGGGGCgaTGTGAGGAAGgCTCcGTGGGAgAAGCAGCCCTGGGCGGCAGAGGTTGGGGGgTGGGGGcTTTTCAAGCTGGGGAAATGGCACAAACAAGGTGTGCGAAGTGACAAATATTCAGGGCCCCTTAAG
ST-E00192:51:H2KG3CCXX:3:1201:22193:15004- GGgGGGgTGGGCaGctGGGgCgaTGTGAGGaaGGCTCcGtGGGaGggCAgCCctGGGCGGCAgaGGTTGGGGGTGGGGGCTTTTTCAAGCTGGGGAAATGGCACAAACAAGGTGTGCGAAGTGACAAATAT
ST-E00192:51:H2KG3CCXX:1:2106:8988:31230 - GGgGTGGGcaGGcTGGGGCgaTGTGaGGaaGGctCcGTGGGAGGAGcAGcCctGGGCGGCAGAGGTTGGGGGtGGGGGCTTTTTCAAGCTGGGGAAATGGCACAAACAAGGTGTGCGAAGTGACAAATATTCAGGGCCCCTTAAGTGCATT
ST-E00192:51:H2KG3CCXX:3:2218:2482:4280  - AGaGGgcAGGCTGGGgCGATGgGAGGAAGGCTCcgTgGgAGGAGCAGCCctGGgCGGCAGAGGTTGGGGGTGGGGGCTTTTTCAAGCTGGGGAAATGGCACAAACAAGGTGTGCGAAGTGACAAATATTCAGGGCCCCTTAAG
ST-E00192:51:H2KG3CCXX:6:1217:6054:39933  - aGTgGgcAGgCTGGGGCgaTGtGAGGAAGGCTCCGTGGGAGGAGCAGCCCTGGGCGGCAGAGGTTGGGGGTGGGGGCTTTTTCAAGcTGGGGAAATGGCACAAACAAGGTGTGCGAAGTGACAAATATTCAGGGCCCCTTAAGTGCATTGC
ST-E00192:51:H2KG3CCXX:1:2201:20650:11593- GGGCgttgTGaGGagGGCtccGtGGgGGAGCAGCCCTGGGcGGCaGaGGTtGGGGGTGGGGGCTTTTTCAAGCTGGGGAAATGGCACAAACAAGGTGTGCGAAGTGACAAATATTCAGGGCCCCTTA
ST-E00192:51:H2KG3CCXX:6:2215:24690:62752- GGCGATgtGAGGAAGGCTCCGTGGGAGGAGCAGCCCTGGGCGGCAGaGGTTGGGGGTGGGGGCTTTTTCAAGCTGGGGAAATGGCACAAACAAGgtGTGCGAAGTGACAAATATTCAGGGCCCCTTAAGTGCATTGCTCAGTAATATAAt
ST-E00192:51:H2KG3CCXX:1:2211:16468:7972  - AAGGCTCCGTGGGAGGAGCAGCCCTGGGCGGCAGAGGTTGGGGGTGGGGGCTTTTTCAAGCTGGGGAAATGGCACAAACAAGGTGTGCGAAGTGACAAATATTCAGGGCCCCTTAAGTGCATTGCTCAGTAATATAATCTAGGAAAGAAAC
ST-E00192:51:H2KG3CCXX:8:2223:13626:52977- CgCCGTGGGAGgaGCAGCCCTGGGCGGCAGAGGTtGGGGGTGGGGGCTTTTTCAAGCTGGGGAAATGGCACAAACAAGGTGTGCGAAGTGACAAATATTCAGGGCCCCTTAAGTGCATTGCTCAGTAATATAATCTAGGAAAGAAACAAAA
ST-E00192:51:H2KG3CCXX:3:1223:7952:73212  - AGCCCTGGGCGGCAGAGGTtGGGGGTGGGGGCTTtTTCAAGCtGGGGAAATGGCACAAACAAGGTGTGCGAAGTGACAAATATTCAGGGCCCCTTAAGTGCATTGCTCAGTAATATAATCTAGGAAAGAAACAAAACCTGTTTAAAAGCCT
ST-E00192:51:H2KG3CCXX:5:1102:29572:2645  - tagggCccggccctctgcggtGtgggCCcgGGgGGgcgcGagGtTttgGGttGgtGGGgggGGGCGGgctGGGggGatGTGaGGagGgttCgggGGGgGgggcgGCCctGGgcGgcaGaGgTtGGGGgtGGGGGCTTTTTCAAGCTGGGG
ST-E00192:51:H2KG3CCXX:3:2211:12977:15812- TGGGCGGCAGAGGTTGGGGGTGGGGGCTTTTTCAAGCTGGGGAAATGGCACAAACAAGGTGTGCGAAGTGACAAATATTCAGGGCCCCTTAAGTGCATTGCTCAGTAATATAATCTAGGAAAGAAACAAAACCTGTTTAAAAGCCTTGTAG
ST-E00192:51:H2KG3CCXX:6:2223:28750:70804- CGGCAGaGGTTGGGGGTGGGGGCTTTTTCAAGCTGGGGAAATGgCACAAACAAGGTGTGCGAAGTGACAAATATTCAGGGCCCCTTAAGTGCATTGCTCAGTAATATAATCTAGGAAAGAAACAAAAtCTGTTTAAAAGCCTTGTAGATCT
```

```
                 33049600   33049620   33049640   33049660   33049680   33049700   33049720   33049740   33049760   33049780   33049800   33049820   33049840   33049860   33049880
chr8:33049595-33049884  TTGCTTATTCTGATGTCTATCAGTTGTATTCTAATTGCTAAGATGCCTGTCTTTCCTACTATACTAGAAGTTTTTTTTCTTGAATTAGAGGCTTTTATATTATTTACTTTTTTTTTTTCGAGATGGAttttttgagatggagtctcactctgtcgcccaggctggagtgcagtggtgcgatcttggctcacttcaacctctgcctcctgagttcaagcaattctcctgcctcagcctccctagaagctgggattacaggcatgcgccaccatgcctggctaatttttgta

ST-E00144:68:H2LCKCCXX:7:1123:3821:41304 + TGCCTGTCTTTCCTACTATACTAGAAGTTTTTTTTCTTgaaTTaGAgGCTTTTaTATTATTTACTTTTTTTTTTTGtgTggtTTTTTGagtTtgaGTcTcacTcTgTcGCCCagGcTTgaGTgcagtGGTgcgatcTTGgTTtacTT
ST-E00144:68:H2LCKCCXX:4:1112:22426:47404+ AgGCTTTTATAtTATTTACTTTTTTTTTTTCGAtATGTaTtTTTTGAaATGgagTCtCcCTcTGTCgccagtcTTgagtGCAGtggaGcgATctTggcTcacTtCaacctCaGCagccTgAgtTcTagcagttCctaTgacTcAgcC
ST-E00144:68:H2LCKCCXX:4:1112:22711:47650+ AGGCTTTTATATTATTTACTTTTTTTTTTTCGAGATGGATTTTTTGAGATGGAGTCTCACTCTGTCGCCCAGGCTGGAGTGCAGTGGTGCGATCTTGGCTCACTTCAACCTCTGCCTCCTGagtTCAAGCAATTCTCCTGCCTCAGCCTC
ST-E00144:68:H2LCKCCXX:4:1112:22497:47773+ AGGCTTTTATATTATTTACTTTTTTTTTTTCGAGATGGATTTTTTGAGATGGAGTCTCACTCTGTCGCCCAGGCTGGAGTGCAGTGGTGCGATCTTGGCTCACTTCAACCTCTGCCTCCTGAGTTCAAGCAaTTcTCCTGCCTCAGCCTC
ST-E00144:68:H2LCKCCXX:3:1112:17930:28646+ GGCTTTTATATTATTTACTTTTTTTTTTTCGAGATGGATTTTTTGAgATGGAGTCTCACTCTGTCGCCCAGGCTGGAGTGCAGTGGTGCGATCTTGGCTCACTTCAACCTCTgCCTCCTgAGTTCAAGCAATTCTCCTGCCTCAGCCTC
ST-E00144:68:H2LCKCCXX:3:1215:13951:11453+ GGCTTTTATATTATTTACTTTTTTTTTTTCGAGATGGATTTTTTGAGATGGAGTCTCACTCTGTCGCCCAGGCTGGAGTgCAGTGGTGCGATCTTGGCTCACTTCAACCTCTGCCTCCTGAGTTCAAGCAATTCTCCTGCCTCAGCCTCC
ST-E00144:68:H2LCKCCXX:3:2124:20752:5088 + TATTTACTTTTTTTTTTTCGAGATGGATTTTTTGAGATgGAGTCTCACTCTGTCGCCCAGGCTGGAGTGCAGTGGTGCGATCTTgGCTCACTTCAACCTCTGCCTCCTGAGTTCAaGCAATTCTCCTgCCTCagcCtCCCTAGAAGCTGG
ST-E00144:68:H2LCKCCXX:4:1218:26040:63438+ CGAGATGGATTTTTTGAGATGGAGTCTCACTCTGTCGCCCAGGCTGGAGTGCAGTGGTGCGATCTTGGCTCACTTCAACCTCTGCCTCCTGAGTTCAAGCAATTCTCCTGCCTCAGCCTCCCTAGAAGCTGGgaTTACAGGCATGCGCCA
ST-E00144:68:H2LCKCCXX:7:2217:20792:35749+ TATTTACTTTTTTTTTTTTTtTttaTTTTTGagaTtGagTcTcacTcTgTcgccCAGgcTggagTGCAGTGGTGCGAtTTGgcTcacTTcaacTcTgcTcctttttCtctatCacTTcTccTttcTcaccttcctagaTTcTg
ST-E00144:68:H2LCKCCXX:8:2217:2674:16041 + TTTTTGAgATGGAGTCTCACTCTGTCGCCCAGGCTGGAGTGCAGTGGTGCGATCTTGGCTCACTTCAACCTCTGCCTCCTGAGTtCAAGCAATTCTCCTtCCTCAGCCTCCCTAGAAGCgGgtAttaCAGGcATGCgCC

ST-E00144:68:H2LCKCCXX:3:1113:6542:3963  + TgAGATGGAGTCTCACTCTGTCGCCCAGGCTGGAGTGCAGTGGTGCGATCTTGGCTCACTTCAACctCTGCCTCCTGagTTCAAGCAATTCTCCTGCCTCAGCCTCCcTAGAAGCTGgGATTcCAGGcAtgCGCCACCATGCCTGGCtA
ST-E00144:68:H2LCKCCXX:8:1106:3202:27328 + GAGATGGAGTCTCACTCTGTCGCCCAGGCTGGAGTGCAGTGGTGCGATCTTGGCTCACTTCAACCTCTGCCTCCTGAGTTCAAGCAATTCTCCTGCCTCAGCCTCCCTAGAAGCTGGGATTACAGGCATGCGCCACCATGCCTgGCTAAT
ST-E00144:68:H2LCKCCXX:1:2224:5851:7726  + TGgAGTCTCACTCTGTCGCCCAGGCTGGAGTGCAGTGGTGCGATCTTGGCTCACTTCAACCTCTGCCTCCTGAGTTCAAGCAATTCTCCTGCCTCAGCCTCCCTAGAAGCTGGGATTACAGGCATGCGCCACCATGCCTcgCTaATTTtt
ST-E00144:68:H2LCKCCXX:8:2203:11637:59482+ TGGAGTCTCACTCTGTCGCCCAGGCTGGAGTGCAGTGGTGCGATCTTGGCTCACTTCAACCTCTGCCTCCTGAGTTCAAGCAATTCTCCTGCCTCAGCCTCCCTAGAAGCTGGGATTACAGGCATGCGCCACCATGCCTGGCTaATTTTT
ST-E00144:68:H2LCKCCXX:3:1122:17331:10626+ GTTTGTGCCATTTCCCCAGCTTGAAAAAGCCCCCACCCCCAACCTCTGCGCCCAGGCTGGAGTGCAGTGGTGCGATCTTGGCTCACTTCAACCTCTGCCTCCTGAGTTCAAGCAATTCTCCTGCCTCAGCCTCCCTAGAAGCTGGGAGG
ST-E00144:68:H2LCKCCXX:3:1122:17483:11242+ GTTTGTGCCATTTCCCCAGCTTGAAAAAGCCCCCACCCCCAACCTCTGCGCCCAGGCTGGAGTGCAGTGGTGCGATCTTGGCTCACTTCAACCTCTGCCTCCTGAGTTCAAGCAATTCTCCTGCCTCAGCCTCCCTaGcAGCTGGGAGG
ST-E00144:68:H2LCKCCXX:3:1122:19087:11347+ GTTTGTGCCATTTCCCCAGCTTGAAAAAGCCCCCACCCCCAACCTCTGCGCCCAGGCTGGAGTGCAGTGGTGCGATCTTGGCTCACTTCAACCTCTGCCTCCTGAGTTCAAGCAATTCTCCTGCCTCAGCCTCCCTAGAAGCTGGGAG
ST-E00144:68:H2LCKCCXX:1:1202:24852:44222- ccccattCtcgTGTctttCCgttcTAtTctatTtgtTttgtTttcTgTCTTTCcTaCtaTacTaGatGTTTTTTTTCTTGAATTAGAGGCTTTTATATTATTTACTTTTTTTTTTTCGAGATGGATTTTTTGAGATGGAGTCTCACTCTG
ST-E00144:68:H2LCKCCXX:8:2117:32465:18660- TCTtccctCtataCtagactTTTTTTTTcTTgaaTtagtggCTTTTaTaTTcTTttcTTTTTTTTTTTCGAGATGGATTTTTTGAGATGGAGTCTCACTCTGTCGCCCAGGCTGGAGTGCAGTGGTGCGATCTTGgCTCACTTCAACCT
ST-E00144:68:H2LCKCCXX:1:1202:24852:44187- cctcattctaccttccacgcactctattatttttgcTgtgatcttcTTCTctccTaatttAatggtatTTTtTtTcTTGAATtAGcggCtTTTtTaTTAtTTacgtTttTTTtTCGaGATGGAttTttTGAgATgGAGTCtCACTCTG
ST-E00144:68:H2LCKCCXX:7:2224:11505:64844- tcttgaaTtagaggCTTTTtTATTaTTTacTTTTTTTTTTTCGAGATGGATTTTTTGAGATGGAGTCTCACTCTGTCGCCCAGGCTGGAGTGCAGTGGTGCGATCTTGGCTCACTTCAACCTCTGCCTCCTGAGTTCAAGCAATTCTCCT
ST-E00144:68:H2LCKCCXX:3:2115:10531:9150  - tcctttTTTTTTtCGagaTGGATTTTTTGAGATGGAGTCTCACTCTGTCGCCCAGGCTGGAGTGCAGTGGTGCGATCTTGGCTCACTTCAACCTCTGCCTCCTGAGTTCAAGCAATTCTCCTGCCTCAGCCTCCCTAGAAGCTGGGATT
ST-E00144:68:H2LCKCCXX:7:1118:3943:27662  - ttttTTTTTGaGATGgATTTTTTGaGaTGgtgtctcaCTCTGTcgccaggcTGgAGTGCAGTGGTGCGATCTTGGCTCACTTCAaCCTCTGCCTCCTGAGTTCAAGCAATTCTCCTGCCTCAGCCTCCCTAGAAGCTGGGATTACAGG
ST-E00144:68:H2LCKCCXX:1:2202:25075:51395- TTTctTgagAtggaGTctCacTCTGTCgCCCAgGCTggtGTGcAGTGGTGCGATCTTGGCTCACTTCAACCTCTGCCTCCTGAGTTCAAGCAATTCTCCTGCCTCAGCCTCCCTagaaGCTGGGATTACAGGCATgCGCCACCATGCCTG
ST-E00144:68:H2LCKCCXX:3:1122:17331:10626- TTGTGCCATTTCCCCAGCTTGAAAAAGCCCCCaCCCCAACCTCTGCGCCCAGGCTGGAGTGCAGTGGTGCGATCTTGGCTCACTTCAACCTCTGCCTCCTGAGTTCAAGCAATTCTCCTGCCTCAGCCTCCCTAGAAGCTGGGAGGCT
ST-E00144:68:H2LCKCCXX:3:1122:17483:11242- TTGTGCCATTTCCCCAGCTTGAAAAcGCCCCCACCCCAACCTCTGCGCCCAGGCTGGAGTGCAGTGGTGCGATCTTGGCTCACTTCAACCTCTGCCTCCTGAGTTCAAGCAATTCTCCTGCCTCAGCCTCCCTAGAAGCTGGGAGGCT
ST-E00144:68:H2LCKCCXX:3:1122:19087:11347- TTGTGCCATTTCCCCAGCTTGAAAAAGCCCCCACCCCAACCTCTGCGCCCAGGCTGGAGTGCAGTGGTGCGATCTTGGCTCACTTCAACCTCTGCCTCCTGAGTTCAAGCAATTCTCCTGCCTCAGCCTCCCTAGAAGCTGGGAGGCT
```

```
ST-E00192:51:H2KG3CCXX:3:2103:13241:5405 + TTGCTTATTCTGATGTCTATCAGTTGTATTCTAATTGCTAAGATGCCTGTCTTTCCTACTATACTAGAAGTTTTTTTTCTTGAATTAGAGGCTTTTATATTATTTACTTTTTTTTTTTtgAGaTggaTTTTTTaGaTgGagTtTCACTC
ST-E00192:51:H2KG3CCXX:3:1222:22223:8974 + ACTTTTTTTTTTTctagaTgGATTTTTTgataTtGAgTCTCacTCTctGTCGCCagGcTGGAgTGgaGTGgTgggATcTTGGCTCACTtcaaCTcTcTgccTtcTgagtTcaagttaTTgTcctgcCTCAGCCtccCTagacgctgggaTTt
ST-E00192:51:H2KG3CCXX:8:1209:23391:35749+ TTTTCGAGATGGATTTTTTGAGATGGAGTCTCACTCTGTCGCCCAGGCTGGAGTGCAGTGGTGCGATCTTGGCTCACTTCAACCTCTGCCTCCTGAGTTCAAGCAATTCTCCTGCCTCAGCCTCCCTAGAAGCTGGGATTACAGGCATGC
ST-E00192:51:H2KG3CCXX:6:2107:17433:37770+ TtCGAGATGGATTTTTTGAGATGGAGTCTCACTCTGTCGCCCAGGCTGGAGTGCAGTGGTGCGATCTTGGCTCACTTCAACcTCTGccTCcTgAGTTCAagCAATTCTcCTGCCTCAGCCTCCCTAGAAtcTGGGaTtacaggCatGCGC
ST-E00192:51:H2KG3CCXX:6:2221:8663:24515 + TCGAGATGGATTTTTTGAGATGGAGTCTCACTCTGTCGCCCAGGCTGGAGTGCAGTGGTGCGATCTTGGCTCACTTCAACCTCTGCCTCCTGAGTTCAAGCAATTCTCCTGCCTCAGCCTCCCTAGAAGCTGGGaTTACAGGCATacGC
ST-E00192:51:H2KG3CCXX:6:2102:14489:17342+ ATTTTTGAGATgGAGTCTCACTCTGTCGCCCAGGCTGGAGTGCAGTGGTGCGATCTTGGCTCACTTCAACCTCTGCCTCCTGAGTTCAAGCAATTCTCCTGcctCAGCgTCCCTAGAcgCTGGGATtAcAggCATGCGCCAcCATgcctT
ST-E00192:51:H2KG3CCXX:8:2217:17209:43044+ AAAATAAATTAACAAACGTATCCTAATTTTTTTTTTTTTTTTTTattTgGagacTcaCTcTgTcGcccaggcTGGTgTgcagTtGTgcGATcTTgGCTcacTtcAaCcTCtCcTCctgGGTTCAAgCAATTCTcT
ST-E00192:51:H2KG3CCXX:2:1123:25898:53557+ GGAGTCTCACTCTGTCGCCCAGGCTGGAGTGCAGTGGTGCGATCTTGGCTCACTTCAACCTCTGCCTCCTGAGTTCAAGCAATTCTCCTGCCTCAGCCTCCCTAGAAGCTGGGATTACAGGCATGCGCCACCATGCCTGGCTAATTTTTG
ST-E00192:51:H2KG3CCXX:6:1105:23604:13615+ GGAGTCgCTCACTCTGgCGCCCAGGCTGGAGTGCAGtGGTGCGATCTTGGCTCACTTCAACcTcttccTCCTgAGTTCAAGCAATgCTcCtGCCTCAGCCTCCCTAGAAgCgGGGATTacGGCATGcgCCACCatgctTGGcTAcTTTtG
ST-E00192:51:H2KG3CCXX:3:1105:23543:42939+ AGTCTCACTCTGTCGCCCAGGCTGGAGTGCAGTGGTGCGATCTTGGCTCACTTCAACCTCTGCCTCCTGAGTTCAAGCAATTCTCCTGCCTCAGCCTCCCTAGAAGCTGGGATTACAGGCATGCGCCACCATGCCTgGCTaATTtTGTA
ST-E00192:51:H2KG3CCXX:1:1112:9861:5563  - TactctcattTCTaTCAcTTGtAttcTAcTtgcTatttTgcTtgTcTTTttcTcTCtataCTAGAAGTTTTTTTTCTTGAATTAGAGGCTTTTATATTATTTaCTTTTTTTTTTTcgaGATGGATTTTTTGAGATGGAGTCTCACTCTG
ST-E00192:51:H2KG3CCXX:3:2218:10327:2082 - attttctacgAtgcCTGtcTTtcctacTaTaCTaTatGTTTTTTTTCTTGAATTAgaGGCTTTTATATTATTTACTTTTTTTTTTTCGAGATGGATTTTTTGAGATGGAGTCTCACTCTGTCGCCCAGGCTGGAGTGCAGTGGTGCGATC
ST-E00192:51:H2KG3CCXX:3:1107:12337:10732- CCtgTCtTcCcttCtaTAcTacaAgTTTTTTTTTCTTgtTTagaGGcTTTTATATTATTTACTTTTTTTTTTTCGAGATGGATTTTTTGAGATGGAGTCTCACTCTGTCGCCCAggcTGGAGTGCAGTGGTGCGATCTTGGCTCACTTC
ST-E00192:51:H2KG3CCXX:3:2218:10125:1906 - actttctacgtttCCttTCTTtcCTtCTattcTagatgTTTTTTTTCtgaATTAGAGGCTTTTATATTATTTACTTTTTTTTTTTCGAGATGGATTTTTTGAGATGGAGTCTCACTCTGTCGCCCAGGCTGGAGTGCAGTGGTGCGATC
ST-E00192:51:H2KG3CCXX:8:1108:26476:39827- TCTTtccTcctatcctagtcGTTTTTTTCTTgatTTatgGgcTTTTATATTATTTACTTTTTTTTTTTCGAGATGGATTTTTTGAgATGGAGTCTCACTCTGTCGCCCAGGtTgGAGTGCAGTGGTGCGATCTTGGCTCACTTCAAC
ST-E00192:51:H2KG3CCXX:2:1109:32769:31213- AagatgCctGtcTtttcTtcttatcTatatGTTTTTTTTcTtgaTtTAgaGgCTTTtaTaTTaTTTaCTTTTTTTTTTTCGAGATGGATTTTTTGAGATGGAGTCTCACTCTGTCGCCCAGGCTGGAGTGCAGTGGTGCGATCTTGGCTC
ST-E00192:51:H2KG3CCXX:3:2212:11921:55333- aatttcTcatcttcCTcTcTTcCTacTaTtTTtaTGTTTTTTTTCTTgTATTAgAgGCTTTTATATTATTTaCTTTTTTTTTTTCgAGaTGGATTTTTTGAGATGGAGTCTCACTCTGTCGCCCAGGCTGGAGTGCAGTGGTGCGATC
ST-E00192:51:H2KG3CCXX:8:1111:23685:70435- cTtCTttactttatGTTTTTTTTCTTgAaTTagaggCTTTTATATTATTTaCTTTTTTTTTTTCGAGATGGATTTTTTGAGATGGAGTCTCACTCTGTCGCCCAGGCTGGAGTGCAGTGGTGCGATCTTGGCTCACTTCAACCTCTGCCT
ST-E00192:51:H2KG3CCXX:2:1114:28963:33235- gacTTagatGCTTTTATaTTaTTtaCTTTTTTTTTTTCGAGATGGATTTTTTGAGATGGAGTCTCACTCTGTCGCCCAGGCTGGAGTGCAGTGGTGCGATCTTGGCTCACTTCAACCTCTGCCTCCTGAGTTCAAGCAATTCTCCTGCCT
ST-E00192:51:H2KG3CCXX:3:2220:10845:52907- TTtTTTCgagaTggaTTTTTGagATGGAGTCTCACTCTgTCgCCCAGGCTGGAGTGCAGTGGTGCGATCTTGGCTCACTTCAACCTCTGCCTCCTGAGTTCAAGCAATTCTCCTgcTgcCTCAGCCTCCCTAGAAGCTGGGATTACAGGCAT
ST-E00192:51:H2KG3CCXX:5:1218:3852:41392 - tTtTtCgagaTgtATTTTTgagATGgagTCTCACTCTGtCgcccAGGcTGGAGTGCAGTGGTGCGATCTTGGCTCACTTCAACCTCTGCCTCCTGAGTTCAAGCAATTCTCCTGCCTCAGCCTCCCTAGAAGCTGGGATTACAGGCATG
ST-E00192:51:H2KG3CCXX:6:1219:22264:5423  - TTTTCgAgaTggATTTTTgagATggagTCTCACTCTGTCGCCCAGGCTGGAGTGCAGTGGTGCGATCTTGGCTCACTTCAACCTCTGCCTCCTGAGTTCAAGCAATTCTCCTGCCTCAGCCTCCCTAGAAgCTGGGATTACAGGCATGC
ST-E00192:51:H2KG3CCXX:8:2213:29947:53610- TCGagAtgGATTTTTTGAgATGGAGTCTCaCTCTGTcGCCCAGcTGGAGTGCAGTGGTGCGATCTTGGCTCACTTCAACCTCTGCCTCCTGAGTTCAAGCAATTCTCCTGCCTCAGCCTCCCTAGAAGCTGGGATTACAGGCATGCGCC
ST-E00192:51:H2KG3CCXX:8:2213:30090:53680- TCgagaTggATTTTTTGagatggAGTCTCACTCTGTCgCCcAGGCTGGAGTGCAGTGGTGCGATCTTGGCTCACTTCAACCTCTGCCTCCTGAGTTCAAGCAATTCTCCTGCCTCAGCCTCCCTAGAAGCTGGGATTACAGGCATGCGCC
ST-E00192:51:H2KG3CCXX:8:1101:18569:62910- ATGGATttTcTGaGATggAGTCTCACTCTGTCGCCCAgGCTGgAGTGCAGTGGTGCGATCTTGGCTCACTTCAACCTCTGCCTCCTGAGTTCAAGCAATTCTCCTGCCTCAGCCTCCCTAGAAGCTGGGATTACAGGCATGCGCCACCAT
ST-E00192:51:H2KG3CCXX:1:1114:19676:48688- ttgAgctgGAGTCTCACTCTGTCGCCCagGCTGgagTGtAgTggTGCgaTcTTGGCTCACTTCAACCTCTGCCTCCTGAGTTCAAGCAATTCTCCTGCCTCAGCCTCCCTAGAaGCTGGGATTACAGGCATGCgcCACCATGCCTGGCTA
```

chr7 76812482 + 0 chr8 76679695 + 6 CTX 44 29 0 49 0.893170254403131 0 0.98962962962963 0 1 chr7 76812434 150 chr8 76679795

AAATTCCTCATCACAACAGACGCCTCTCCCACACCTCTTTCTTCCCTCTTTAGCCCTTTGTCAGATTGATAAATGGCAAAAATTTTCTCCCTTTCTGTAAGTTGCCTGTTCACTCTGATGATAGTTTCTTTTGCTGTGCAGAAGCTCCTT

```
                 76812360   76812380   76812400   76812420   76812440   76812460   76812480   76812500   76812520   76812540   76812560   76812580   76812600   76812620
chr7:76812341-76812625  AGAAAGCCTTGGAAAGCAATATGGCTGTGCTGAAATCTCCTTCATGAAATGGAGGCATGACTTTCACAGCATATCAAGCGGCAAAACTTCCCCAAATTCCTCATCACAACAGACGCCTCTCCCACACCTCTTTCTTCCCTCTTTTAAAATCTCCTCAGAAACAGGGCTTCCCACCCTCAGGCTATATATTTCTTCTCTGTATCTTTTTCCATCTTCTCCTTGATCTCTTTGGCCATTTCATTATTTTGTAAAGCAGTCTGCAGAGTTTTTTCCACTTTTCCTTCC

ST-E00144:68:H2LCKCCXX:6:1202:20589:6038 + CTTGGAAAGCAATATGGCTGTGCTGAAATCTCCTTCATGAAATGGAGGCATGACTTTCACAGCATATCAAGCGGCAAAACTTCCCCAAATTCCTCATCACAACAGACGCCTCTCCCACACCTCTTTCTTCCCTCTTTTAAAATCTCCtCA
ST-E00144:68:H2LCKCCXX:8:1101:11566:25816+ TTGGAAAGCAATATGGCTGTGCTGAAATCTCCTTCATGAAATGGAGGCATGACTTTCACAGCATATCAAGCGGCAAAACTTCCCCAAATtCCTCATCACAACAGACGCCTCTCCCACACCTCTTTCTTCCCTCTTTTAAAATCTCCTCAg
ST-E00144:68:H2LCKCCXX:7:2217:16164:67956+ GGAAAGCAATATGGCTGTGCTGAAATCTCCTTCATGAAATGGAGGCATGACTTTCACAGCATATCAAGCGGCAAAACTTCCCCAAATTCCTCATCACAACAGACGCCTCTCCCACACCTCTTTCTTCCCTCTTTTAaAATCTCCTCAGAA
ST-E00144:68:H2LCKCCXX:1:2224:31703:5528 + TGTGCTGAAaTCTCCTTCATGAAATGGAGGCAtGACTTTCACAGCATAtCAAGCGGCAAAACTTCCCCAAATTCCTCATCACAACAGACGCCTcgCCCACACCTCTTTCTTCCCtCTCTTTTAAaATCTCctCAGAAaCAGGgcgtCC
ST-E00144:68:H2LCKCCXX:3:1114:15159:16955+ GAAATCTCCTTCATGAAATGGAGGCATGACTTTCACAGCATATCAAGCGGCAAAACTTCCCCAAATTCCTCATCACAACAGACGCCTCTCCCACACCTCTTTCTTCCCTCTTTTAAAATCTCCTCAGAAACAGGGCTTCCCACCCtCaGg
ST-E00144:68:H2LCKCCXX:3:2222:5679:29191 + gcAaTcTCcTTcaTgataTgGAttCATGACTTTCACAGCATATCAAGCgGCaAAACTTCCCCAAATTCCTcATCACAACAGACGCCTctCCCACACCTcTTtCTTccctCttTTAAaaTCTCCTCagaaacaggGctgaccaacctcAGg
ST-E00144:68:H2LCKCCXX:7:2107:25979:53487+ GAAATCTCCTTCATGAAATGGAGGCATGACTTtCACAGCATATCAAGCGGCAAAACTTCCCCaAaTTCCTCATCACAACAgaCGCCTCTCCCACACCTCTTTCTTCCCTCTTTTAAAATCTCCTCAGAAACAGGGCTTCCCACCCTCAGg
ST-E00144:68:H2LCKCCXX:6:1217:25461:65037+ ATCTCCTTCATGAAATGGAGGCATGACTTTCACAGCATATCAAGCGGCAAAACTTCCCCAAATTCCTCATCACAACAGACGCCTCTCCCACACCTCTTTCTTCCCTCTTTTAAAATCTCCTCAGAAACAGGGCTTCCCACCCTCAGGCT
ST-E00144:68:H2LCKCCXX:8:1111:6978:39563 + CTCCTTCATGAAATGGAGGCATGACTTTCACAGCATATCAAGCGGCAAAACTTCCCCAAATTCCTCATCACAACAGACGCCTCTCCCACACCTCTTTCTTCCCTCTTTTAAAATCTCCTCAGAAACAGGGCTTCCCACCCTCAGGCTAta
ST-E00144:68:H2LCKCCXX:8:1213:22802:71788+ TTCATGAAATGGAGGCATGACTTTCACAGCATATCAAGCGGCAAAACTTCCCCAAATTCCTCATCACAACAGACGCCTCTCCCACACCTCTTTCTTCCCTCTTTTAAAATCTCCTCAGAAaCAGGGCTTCCCACCCTCAGGCTcATaTATT
ST-E00144:68:H2LCKCCXX:8:1210:9911:2698  + ATGAAATGGAGGCATGACTTTCACAGCATATCAAGCGGCAAAACTTCCCCAAATTCCTCATCACAACAGACGCCTCTCCCACACCTCTTTCTTCCCTCTTTTAAAATCTCCTCAGAAACAGGGCTTCCCACCCTCAGGCTaTATATTTCT
ST-E00144:68:H2LCKCCXX:1:2118:32617:32813+ GCATGACTTTCACAGCATATCAAGCGGCAAAACTTCCCCAAAttCCTCATCACAACAGACGCCTCTCCCACACCTCTTTCTTCCCTCTTTTAAAATCTCCTCAgAAACAGGGCTTCCCACCCTCAgGCTATATAtTTCTTCTCTGTATCT
ST-E00144:68:H2LCKCCXX:4:1222:6257:66057 + ATGACTTTCACAGCATATCAAGCGGCAAAACTTCCCCAAATTCCTCATCACAACAGACGCCTCTCCCACACCTCTTTCTTCCCTCTTTTAAAATCTCCTCAGAAACAGGGCTTCCCACCCTCAGGCTATATATTTCTTCTCTGTATCTTT
ST-E00144:68:H2LCKCCXX:1:1107:13241:16762+ AAAACTTCCCCAAATTCCTCATCACAACAGACGCCTCTCCCACACCTCTTTCTTCCCTCTtTtAAAATCTCCTCAGAAACAGGGCTTCCCaCCCTCAGGCTATATATTTCTTCTCTGTATCTTTTTCcATCTTCTCCgTGaTcTCTgTGG
ST-E00144:68:H2LCKCCXX:6:1109:20934:62787+ CTCATCACAACAGACGCCTCTCCCACACCTCTTTCTTCCCTCTTTTAAAATCTCCTCAGAAACAGGGCTTCCCACCCTCAGGCTATATATTTCTTCTCTGTATCTTTTTCCATCTTCTCCTTGATCTCTTTGGCCATTTcaTTATTTTtT
```

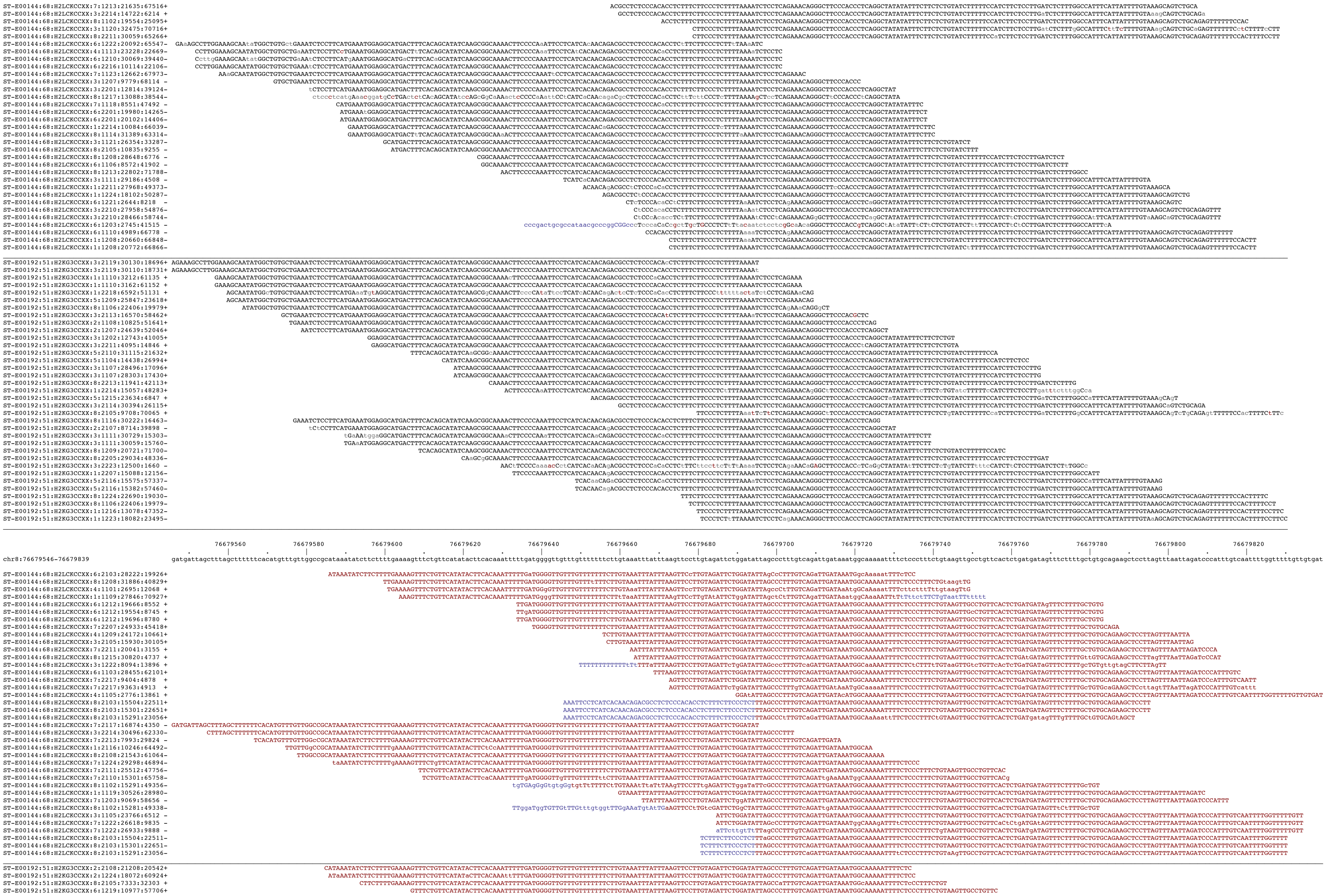

76679560 76679580 76679600 76679620 76679640 76679660 76679680 76679700 76679720 76679740 76679760 76679780 76679800 76679820
chr8:76679546-76679839

```
ST-E00192:51:H2KG3CCXX:2:2119:20579:54366+  ACTTCACAAATTTTGATGGGGTTGTTTGTTTTTTTCTTGTAAATTTATTTAAGTTCCTTGTAGATTCTGGATATTAGCcCTTTGTCaGATTGATAAATGGCaAAAATTTTCTCCCTTTcTGTAAGTtgCCTGTTcaCTctGATGATAGT
ST-E00192:51:H2KG3CCXX:2:2201:4989:44345 +  GTTTTTTTCTTGTAAATTTATTTAAGTTCCTTGTAGATTCTGGATATTAGCCCTTTGTCAGATTGATAAATGGCAAAAATTTTCTCCCTTTCTGTAAGTTGCCTGTTCACTCTGATGATAGTTTCTTTTGCTGTGCAGAAGCTCCTTAGT
ST-E00192:51:H2KG3CCXX:5:1120:7871:41462 +  GTAAATTTATTTAAGTTCCTTGTAGATTCTGGATATTAGCCCTTTGTCAGATTGATAAATGGCAAAAATTTTCTCCcTTTCTGTAAGTTGCcTGTTCACTctGAtGATAGTTTCTTTTGCTGTGCAGAAGCTCCTTAGTTtAATTAGATC
ST-E00192:51:H2KG3CCXX:3:1215:26476:30650+  AAATTTATTTAAGTTCCTTGTAGATTCTGGATATTAGCCCTTTGTCAGATTGATAAATGGCAAAAATTTTCTCCCTTTCTGTAAGTTGCCTGTTCACTCTGATGATAGTTTCTTTTGCTGTGCAGAAGCTCCTTAGTTTAATTAGATCCC
ST-E00192:51:H2KG3CCXX:3:1215:26466:30668+  AAATTTATTTAAGTTCCTTGTAGATTCTGGATATTAGCCCTTTGTCAGATTGATAAATGGCAAAAATTTTCTCCCTTTCTGTAAGTTGCCTGTTCACTCTGATGATAGTTTCTTTTGCTGTGCAGAAGCTCCTTAGTTTAATTAGATCCC
ST-E00192:51:H2KG3CCXX:2:1101:10947:42183+  AAGTTCCTTGTAGATTCTGGATATTAGCCCTTTGTCAGATTGATAAATGGCAAAAATTTTCTCCCTTTCTGTAAGTTGCCTGTTCACTCTGATGATAGTTTCTTTTGCTGTGCAGAAGCTCCTTAGTTTAATTAGATCCCATTTGTCAAT
ST-E00192:51:H2KG3CCXX:6:2112:28932:52133+  TAGATTCTGGATATTAGCCCTTTGTCAGATTGATAAATGGCAAAAATTTTCTCCCTTTCTGTAAGTTGCCTGTTCACTCTGATGATAGTTTCTTTTGCTGTGCAGAAGCTCCTTAGTTTAATTAGATCCCATTTGTCAaTTTGGTTTTT
ST-E00192:51:H2KG3CCXX:5:1109:13504:19961+  AGATTCTGGATATTAGCCCTTTGTCAGATTGATAAATGGCAAAAATTTTCTCCCTTTCTGTAAGTTGCCTGTTCACTCTGATGATAGTTTCTTTTGCTGTGCAGAAGCTCCTTAGTTTAATTAGATCCCATTTGTCAATTTGGTTTTTG
ST-E00192:51:H2KG3CCXX:8:2219:11921:67569+  CTGGATATTAGCCCTTTGTCAGATTGATAAATGGCAAAAATTTTCTCCCTTTCTGTAAGTTGCCTGTTCACTCTGATGATAGTTTCTTTTGCTGTGCAGAAGCTCCTTAGTTTAATTAGATCCCATTTGTCAATTTGGTTTTTGTTGTG
ST-E00192:51:H2KG3CCXX:5:1201:4512:58427 -  TAGCTTttttcaCATGtTTGTtGgCcgcataaatATCTtCttTTGAAAAGttTCTGttCAtATaCtTCaCaAATtgTTGATGGGgtTGTTTGTTTTTTtcTTGtaAAttTATTTAAGTTCCTTGTAGATTctGgATATTAGCCCtTTGTC
ST-E00192:51:H2KG3CCXX:5:1215:17778:49408-  TTTCACATGTTTGTTGGCCGCATAAATATCTTCTTTTGAAAAGTTTCTGTTCATATACTTCACAAATTTTGATGGGGTTGTTTGTTTTTTTCTTGTAAATTTATTTAAGTTCCTTGTAGATTCTGGATATTAGCCCTTTGTCAGATTGA
ST-E00192:51:H2KG3CCXX:8:1220:4572:68026 -  CCGCATAAATATCTTCTTTTGAAAAGTTTCTGTTCATATACTTCACAAATTTTGATGGGGTTGTTTGTTTTTTTCTTGTAAATTTATTTAAGTTCCTTGTAGATTCTGGATATTAGCCCTTTGTCAGATTGATAAATGGCAAAAATTTT
ST-E00192:51:H2KG3CCXX:8:2220:3679:71718 -  CCGCATAAATATCTTCTTTTGAAAAGTTTCTGTTCATATACTTCACAAATTTTGATGGGGTTGTTTGTTTTTTTCTTGTAAATTTATTTAAGTTCCTTGTAGATTCTGGATATTAGCCCTTTGTCAGATTGATAAATGGCAAAAATTTT
ST-E00192:51:H2KG3CCXX:6:2215:26162:28347-  TTTGaAAAtTTTCTGTTCATATACTTCaCaAATTTTTGATGGGGTTGTTTGTTTTTTTCTTGTAAATTTATTTAAGTTCCTTGTAGATTCTGGATATTAGCCCTTTGTCAGATTGATAAATGGCAAAAATTTTCTCCCTTTCTGTAAGTT
ST-E00192:51:H2KG3CCXX:5:1121:8460:2645  -  CTGTTCATATACTTCACAAATTTTGATGGGGTTGTTTGTTTTTTTCTTGTAAATTTATTTAAGTTCCTTGTAGATTCTGGATATTAGCCCTTTGTCAGATTGATAAATGGCAAAAATTTTCTCCCTTTCTGTAAGTTGCCTGTTCACTC
ST-E00192:51:H2KG3CCXX:1:1117:29998:16393-  aAaTTTTgATgggGTTgTTTGTTTTTTTCTTGTAaATTTATTTAAGTTCCTTGTAGATTCTGGaTATTAGCCCTTTGTCAGATTgAtAAAtggCAAAAATTTTCTCCCTTTCTGTAAGTTGCCTGTTCACTCTGATGATAGTTTCTTTT
ST-E00192:51:H2KG3CCXX:1:1117:29998:16428-  AaATTTTTgATGGGGTTGTTTGTTTTTTTCTTGTAAATTTATTTAAGTTCCTTGTAGATTCTGGATATTAGCCCTTTGTCAGATTGATAAATGGCAAAAATTTTCTCCCTTTCTGTAAGTTGCCTGTTCACTCTGATGATAGTTTCTTTT
ST-E00192:51:H2KG3CCXX:3:2118:16824:28101-  TTTGTTTTTTTCTTGTAAATTTATTTAAGTTCCTTGTAGATTCTGGATATTAGCCCTTTGTCAGATTGATAAATGGCAAAAATTTTCTCCCTTTCTGTAAGTTGCCTGTTCACTCTGATGATAGTTTCTTTTGCTGTGCAGAAGCTCCTT
ST-E00192:51:H2KG3CCXX:3:2204:22487:33446-  TTTGTTTTTTTCTTGTAAATTTATTTAAGTTCCTTGTAGATTCTGGATATTAGCCCTTTGTCAGATTGATAAATGGCAAAAATTTTCTCCCTTTCTGTAAGTTGCCTGTTCACTCTGATGATAGTTTCTTTTGCTGTGCAGAAGCTCCTT
ST-E00192:51:H2KG3CCXX:2:2117:13109:17518-  TGTTTTTTTCTTGTAAATTTATTTAAGTTCCTTGTAGATTCTGGATATTAGCCCTTTGTCAGATTGATAAATGGCAAAAATTTTCTCCCTTTCTGTAAGTTGCCTGTTCACTCTGATGATAGTTTCTTTTGCTGTGCAGAAGCTCCTTAG
ST-E00192:51:H2KG3CCXX:1:1213:24111:16920-  TTGTAAATTTATTTAAGTTCCTTGTAGATTCTGGATATTAGCCCTTTGTCAGATTGATAAATGGCAAAAATTTTCTCCCTTTCTGTAAGTTGCCTGTTCACTCTGATGATAGTTTCTTTTGCTGTGCAGAAGCTCCTTAGTTTAATTAGA
ST-E00192:51:H2KG3CCXX:3:1207:21208:16182-  TTTATTTAAGTTCCTTGTAGATTCTGGATATTAGCCCTTTGTCAGATTGATAAATGGCAAAAATTTTCTCCCTTTCTGTAAGTTgCCTGTTCaCTCTGATGATAGTTTCTTTTGCTGTGCAGAAGCTCCTTAGTTTAATTAGATCCCATT
ST-E00192:51:H2KG3CCXX:3:1123:25319:35116-  TAAGTTCcTTGTAGaTtCTGGaTATTAGCCcTTTGTCAGATTGATAAATGGCAAAAATTTTCTCCCTTTCTGTAAGTTGCCTGTTCACTCTGATGATAGTTTCTTTTGCTGTGCAGAAGCTCCTTAGTTTAATTAGATCCCATTTGTCAA
ST-E00192:51:H2KG3CCXX:1:1107:12703:9923 -  ATTCTGGATATTAGCCCTTTGTCAGATTGATAAATGGCAAAAATTTTCTCCCTTTCTGTAAGTTGCCTGTTCACTCTGATGATAGTTTCTTTTGCTGTGCAGAAGCTCCTTAGTTTAATTAGATCCCATTTGTCAATTTGGTTTTTGTT
```

---

chr7 62622064 - 0 chr8 85976652 + 5 CTX 24 43 0 59 0.863333333333334 0 1 0 1 chr7 62622122 150 chr8 85976742

GGTTACTACCTGTTAAATAAAAAAAAACTCTATAGCATTTTATCAAGACTCAGATTCACATAGGCAAACGGTCTGAGGTGCCTGACATCCAGGCATTCTTTTACACATCAGTCCCTCCCTAGTCTCTGTTCCCAATGCAACTTGTCCCAA

```
                 62621940  62621960  62621980  62622000  62622020  62622040  62622060  62622080  62622100  62622120  62622140  62622160  62622180  62622200
chr7:62621921-62622213  actgcactgcagcctgggcaacaagagtgacactccatctcaaaaaaaaaaaaattagaaattttaaaagttttctaattttctaattcttaatgtgttgagtaatttttttattgtattctggacatcttacacactatgctctgtgaatctgagtcttgataaaatgctatagagttttttttatttaacaggtagtaaccttgctagagtcagccacaaattcctaccgaccttctgtttgcattggttcattctagtgccaattctgtttacaaggcccttataagaggt

ST-E00144:68:H2LCKCCXX:8:1219:21503:54876+  GCCTGGGCAACAAGAGTGACACTCCATCTCAAAAAAAAAAAAATTAGAAATTTTAAAaGTTTTCTAATTTTCTAATTCTTaatTtGTTgagtaaTTTTTTAtTGTTTTtTggAcATCTTAcACAtTtTgtTtTTaaTctGaGtctTgAT
ST-E00144:68:H2LCKCCXX:8:1217:8693:30369 +  AGTGACACTCCATCTCAAAAAAAAAAAAATTAGAAATTTTAAAAGTTTTCTAATTTTCTAATTCTTAATGTGTTGAGTAATTTTTTTATTGTATTCTGGACATCTTAcACACTATGCTCTGTGAaTcTGAGTCTTGATAAtATGCTATAGAG
ST-E00144:68:H2LCKCCXX:1:1105:31308:52274+  CATCTCAAAAAAAAAAAAATTAGAAATTTTAAAAGTTTTcTAATTTTcTAATTcTTAATGTGTTGaGTacTTTTTTtTTGTATTCTGGaCATCTTACACACTATGCTCTGTGAATcTGaGTattgaTAttaTGCTTTAGaGtTTTTTTT
ST-E00144:68:H2LCKCCXX:8:2222:20234:25394+  ATTAGAAATTTTAAAAGTTTTCTAATTTTCTAATTCTTAATGTGTTGAGTAATTTTTTTATTGTATTCTGGACATCTTACACACTATGCTCTGTGAATCTGAGTCTTGATAAAATGCTATAGAGTTTTTTTTATTTAACAGGTAGTAACC
ST-E00144:68:H2LCKCCXX:1:2217:6745:58357 +  TTAGAAATTTTAAAAGTTTTCTAAtTTTCTAATTCTTAATGCGtTGAGTAATTTTTTTATTGTATTCTGGACATCTTACACACTATGcTCTGTGAATCtGAGTCTTGATAAAATGCTATAGAGTtTTTTttTctTTAacaggtAGTaAgCT
ST-E00144:68:H2LCKCCXX:1:2217:6207:58761 +  TTAGAAATTTTAAAAGTTTTCTAATTTTCTAATTCTTAATGTGTTGAGTAATTTTTTTATTGTATTCTGGACATCTTACACACTATGCTCTGTGAATCTGAGTCTTGATAAAATGCTATAGAGtTTTTTTTATTTAACAGGTAGTAACCT
ST-E00144:68:H2LCKCCXX:6:2103:12033:8798 +  TAGAAATTTTAAAAGTTTTCTAATTTTCTAATTCTTAATGTGTTGAGTAATTTTTTTATTGTATTCTGGACATCTTACACACTATGCTCTGTGAATCTGAGTCTTGATAAAATGCTATAGAGTTTTTTTTATTTAACAGGTAGTAACCTT
ST-E00144:68:H2LCKCCXX:3:2112:25928:56704+  ATTTTCTAATTCTTAATGTGTTGAGTAATTTTTTTATTGTATTCTGGACATCTTACACACTATGCTCTGTGAATCTGAGTCTTGATAAAATGCTATAGAGTTTTTTTTATTTAACAGGTAGTAACCTTGCTAGAGTCAGCCACAAATTCC
ST-E00144:68:H2LCKCCXX:7:2111:6948:8042  +  ATTTTCTAATTCTTAATGTGTTGAGTAATTTTTTTATTGTATTCTGGACATCTTACACACTATGCTCTGTGAATCTGAGTCTTGATAAAATGCTATAGAGTTTTTTTTATTTAAcAGttaGTAAccTTgCTAgagTCAgCcaCAAAttcc
ST-E00144:68:H2LCKCCXX:3:2110:11627:50709+  TCTAATTCTTAATGTGTTGAGTAATTTTTTTATTGTATTCTGGACATCTTACACACTATGCTCTGTGAATCTGAGTCTTGATAAAATGCTATAGAGTTTTTTTTATTTAACAGGTAGTaAccTTGCTAGAgTCAGCCACaaATTtCTacc
ST-E00144:68:H2LCKCCXX:7:2207:3456:32232 +  TAATTTTTTTATTGTATTCTGGACATCTTACACACTATGCTCTGTGAATCTGAGTCTTGATAAAATGCTATAGAGTTTTTTTTATTTAACAGGTAGTAACCTTGCTAGAGTCAGCCACAAATTCCTACCGACCTTCTGTTTGCATTGGTT
ST-E00144:68:H2LCKCCXX:8:1224:4593:12121 -  ACTGCAcTGCAgcCTGGGCaACAaGAGTGACACTCcatCtcAAAAAAAAAAAAATTAGAAATTTTAAAAGTTTTCTAATTTTCTAATTCTTAATGTGTTGAGTAATTTTTTTATTGTATTCTGGACATCTTACACACTATGCTCTGTGAATC
ST-E00144:68:H2LCKCCXX:1:2223:24365:68132-  TCtCAAAAAAAAAAAAATTAGAAATTTTAAAAGTTTTCTAATTTTCTAATTCTTAATGTGTTGAGTAATTTTTTTATTGTATTCTGGACATCTTACACACTATGCTCTGTGAATCTGAGTCTTGATAAAATGCTATAGAGTTTTTTTTATT
ST-E00144:68:H2LCKCCXX:1:2223:24182:67112-  CAAAAAAAAAAAAATTAGAAATTTTAAAAGTTTTCTAATTTTCTAATTCTTAATGTGTTGAGTAATTTTTTTATTGTATTCTGGACATCTTACACACTATGCTCTGTGAATCTGAGTCTTGATAAAATGCTATAGAGTTTTTTTTATT
ST-E00144:68:H2LCKCCXX:8:2116:2380:34307 -  tacacgaAaggcaggcaaaaaaacatgaAaatcAAaaaaAAAAAAAAAAAATTAGAAATTTTAAAAGTTTTCTAATTTcTAATTCTTAATGTGTTGAGTAATTTTTTTATTGTATTCTGGACATCTTACACACTATGCTCTGTGAATCTG
ST-E00144:68:H2LCKCCXX:3:1221:7709:42974 -  AAAAAATTAGAAATTTTAAAAGTTTTCTAATTTTCTAATTCTTAATGTGTTGAGTAATTTTTTTATTGTATTCTGGACATCTTACACACTATGCTCTGTGAATCTGAGTCTTGATAAAATGCTATAGAGTTTTTTTTATTTAACAGGTAG
ST-E00144:68:H2LCKCCXX:6:2122:19260:50674-  TAAtttTCTAAttcttAAtatatTGagTAaTTTTTTATTGTATTCTgGACaTCTTAcaaACTATGCTCTGTGAATCTGAGTCTTGATAAAATGCTATAGAGTTTTTTTTATTTAACAGGTAGTAACCTTGCTAGAGTCAGCCACAAATT
ST-E00144:68:H2LCKCCXX:6:2114:19503:54155-  TAAtttTTTATtgtAttcTGGACATCtTACAcaCTATGCTCTGTGAATCTGAGTCTTGATAAAATGCTATAGAGTTTTTTTTCTTTAACAGGTAGTaaCCTTGCTAGAGtaAGcCACAAATTCCTACCGACCgtcgGgTGGCATTGGTT
ST-E00144:68:H2LCKCCXX:7:1202:30912:10626-  ttatagtattatgaaaaaattacacactattatcttTGAATCTGAGTCTTGATAAAATGCTATAGAGTTTTTTTTATTTAACAGGtAGTAACCTTGCTAGAGTCAGCCACAAATTCCTACCGACCTTCTGTTTGCATTGGTTCATTCTA
ST-E00144:68:H2LCKCCXX:1:2217:6745:58357 -  CTATGcTtTGTGaAtCTGagTCTTGATAAaaTGCTATAGaGTTTTTTTTATTTAACAGGTAGTAACCTTGCTAgAGTCAGCCACAAATTCCTACCGACCTTCTGTTTGCATTGGTTCATTCTAGTGCCAATTCTGTTTACAAGGCCCTT
ST-E00144:68:H2LCKCCXX:1:2217:6207:58761 -  cTatgcTCtgTGAATCTGAGTCTTtATAAAATGCTAgAgAGTTgTTtTTgAtTtAACAGGTAGTAACCTTGCTAgagTCAgCCACAAATTCCTACCGACCTTCTGTTTGCATTGGTTCATTCTAGTGCCAATTCTGTTTACAAGGCCCTT
ST-E00144:68:H2LCKCCXX:3:2112:25928:56704-  ATgcTCTGtgaATCTgAGTCTTGATAAAATGCTATAgAGTTTTTTTTATTTAACAGGTAGTAACCTTGCTAGAGTCAGCCACAAATTCCTACCGACCTTCTGTTTGCATTGGTTCATTCTAGTGCCAATTCTGTTTACAAGGCCCTTAT
ST-E00144:68:H2LCKCCXX:8:1212:13048:50604-  ATGCTcTgtgAATCtGAGTCTTGATAAAATGCTATAGAGTTTTTTTTATTTAACAGGTAGTAACCTTGCTAGAGTCAGCCACAAATTCCTACCGACCTTCTGTTTGCATTGGTTCATTCTAGTGCCAATTCTGTTTACAAGGCCCTTAT
ST-E00144:68:H2LCKCCXX:6:2216:8379:31371 -  TgctctgTGAAtctGAgtaatgATAAAATgCTATAgaGTTTTTTTTATTTAaCAGGTAgTAACCTTGCTAGAGTCAGCCACaAATTCCTACCGACCTTCTGTTTGCATTGGTTCATTCTAGTGCCAATTCTGTTTACAAGGCCCTTATA
ST-E00144:68:H2LCKCCXX:8:1206:30891:13650-  AaAaaaAAcAcAatttgCtcttTGAATCTGagTCTTGATAAAATGCTATAGAGTTTTTTTTATTTAACAGGTAGTAACCTTGCTAGAGTCAGCCACAAATTCCTACCGACCTTCTGTTTGCATTGGTTCATTCTA
ST-E00144:68:H2LCKCCXX:8:2116:3994:27855 -  AATtTTtTActgaatacTaaAaAtAtTaCACAtTtTgCttTgTgaaTCTgAgTCTTGATAAAATGcTATAGAGTTTTTTTTATTTAACAGGTAGTAACCTTGCTAGAGTCAGCCACAAATTCCTACCGACCTTCTGTTTGCATTGGTT
ST-E00144:68:H2LCKCCXX:1:2120:32576:59711-  gtgAATcTGAgTCTTGATAAAATgCTATAGAGTTTTTTTTATTTAACAGGTAGTAACCTTGCTAGAGTCAGCCACAAATTCCTACCGACCTTCTGTTTGCATTGGTTCATTCTAGTGCCAATTCTGTTTACAAGGCCCTTATAAGAGGT
```

```
ST-E00192:51:H2KG3CCXX:5:1205:24527:50938+  CAAAAAAAAAAAAATTAGAAATTTTAAAAGTTTTCTAATTtTCTAATTCTTAATGTGTTGAgtaATTTTTTATTgtaTTCTgGtcATcTTACACACTATGCTCTGTGAAtCTGAGTcTTGATAaaaTGcTATAGAGTTTTTTTTaTtTaa
ST-E00192:51:H2KG3CCXX:3:1118:30699:3717 +  AAATTAGAAATTTTAAAAGTTTTCTAATTTTCTAATTCTTAATGTGTTGAGTAATTTTTTTATTGTATTCTGGACATCTTACACACTATGCTCTGTGAATCTGAGTCTTGATAAAATGCTATAGAGTTTTTTTTATTTAACAGGTAGTAA
ST-E00192:51:H2KG3CCXX:3:1221:17828:30756+  AAGTTTTCTAATTTTCTAATTCTTAATGTGTTGAGTAATTTTTTTATTGTATTCTGGACATCTTACACACTATGCTCTGTGAATCTGAGTCTTGATAAAATGCTATAGAGTTTTTTTTATTTAACAGGTAGTAACCTTGCTAGaGTCAGC
ST-E00192:51:H2KG3CCXX:3:1221:17818:30773+  AAGTTTTCTAATTTTCTAATTCTTAATGTGTTGAGTAATTTTTTTATTGTATTCTGGACATCTTACACACTATGCTCTGTGAATCTGAGTCTTGATAAAATGCTATAGAGTTTTTTTTATTTAACAGGTAGTAACCTTGCTAGAGTCAGC
ST-E00192:51:H2KG3CCXX:2:2114:14794:7532 +  TTTCTAATTTTCTAATTCTTAATGTGTTGAGTAATTTTTTTATTGTATTCTGGACATCTTACACACTATGCTCTGTGAATCTGAGTCTTGATAAAATGCTATAGAGTTTTTTTTATTTAACaggTAgTAACttTGCTAGaGTCAGCCACA
ST-E00192:51:H2KG3CCXX:5:2104:10662:65354+  TCTAATTCTTAATGTGTTGAGTAATTTTTTTATTGTATTCTGGACATCTTACACACTATGCTCTGTGAATCTGAGTCTTGATAAAATGCTATAGAGTTTTTTTTATTTAACAGGTAGTAACCTTGCTAGAGTCAGCCaCAaATTCCtACC
ST-E00192:51:H2KG3CCXX:2:2204:4106:69186 +  CTAATTCTTAATGTGTTGAGTAATTTTTTTATTGTATTCTGGACATCTTACACACTATGCTCTGTGAATCTGAGTCTTGATAAAATGCTATAGAGTTTTTTTTATTTAACAGGTAGTAACCTTGCTAGAGTCAGCCACAAATTCCTACCG
ST-E00192:51:H2KG3CCXX:6:2103:15382:71067+  TCTTAATGTGTTGAGTAATTTTTTTATTGTATTCTGGACATCTTACACACTATGCtCTGTGAATCTGAGTCTTGATAAAATGCTATAGAGTTTTTTTTATTTAACAGGTAGTAACCtTGCTAGAGTcagCCACAAATTCCTACCGAcCTT
ST-E00192:51:H2KG3CCXX:3:2219:2502:29279 +  AATGtgTTGAGTAATTTTTTTATTGTATTCTGGACATcTTACACACTATGCTCtGTGAATCtGAGTCTTgATAAAATGcTaTAGAGTTTTTTTTaTTTAAcagtTAGTAttATTGCTAGagtcagtcatAtattactctcgcacTTcagt
ST-E00192:51:H2KG3CCXX:6:1221:19412:46613+  GTGTTGAGTAATTTTTTTATTGTATTCTGGACATCTTACACACTATGCTCTGTGAATCTGAGTCTTGATAAAATGCTATAGAGTTTTTTTTATTTAACAGGTAGTAACCTTGCTAGAGTCAGCCACAAATTCCTACCGACCTTCTGTTTG
ST-E00192:51:H2KG3CCXX:6:1221:19473:46648+  GTGTTGAGTAATTTTTTTATTGTATTCTGGACATCTTACACACTATGCTCTGTGAATCTGAGTCTTGATAAAATGCTATAGAGTTTTTTTTATTTAACAGgTAGTAACCTTGCTAGAGTCAGCCACAAATTCCTACCGACCTTCTGTTTG
ST-E00192:51:H2KG3CCXX:1:2205:6775:60027 +  ATTCTGGACATCTTACACACTATGCTCTGTGAATCTGAGTCTTGATAAAATGCTATAGAGTTTTTTTTATTTAACAGGTAGTAACCTTGCTAGAGTCAGCCACAAATTCCTACCGACCTTCTGTTTGCATTGGTTCATTCTAGTGCCAA
ST-E00192:51:H2KG3CCXX:3:2101:14276:68167-  AACAAGAgatACACTACAtctcAAAAAAAAAAAAATTAGAAATTTTAAAAGTTTTCTAATTTTCTAATTCTTAATGTGTTGAGTAATTTTTTTATTGTATTCTGGACATCTTACACACTATGCTCTGTGAATCTGAGTCTTGATAAAATGCT
ST-E00192:51:H2KG3CCXX:3:2216:2248:37102 -  AAGAGTGACAcTCCAtCTCAAAAAAAAAAAAATTAGAAATTTTAAAAGTTTTCTAATTTTCTAATTCTTAATGTGTTGAGTAATTTTTTTATTGTATTCTGGACATCTTACACACTATGCTCTGTGAATCTGAGTCTTGATAAAATGCTATA
ST-E00192:51:H2KG3CCXX:3:2216:2664:37471 -  AAGAGTGACACTCCATCTCAAAAAAAAAAAAATTAGAAATTTTAAAAGTTTTCTAATTTTCTAATTCTTAATGTGTTGAGTAATTTTTTTATTGTATTCTGGACATCTTACACACTATGCTCTGTGAATCTGAGTCTTGATAAAATGCTATA
ST-E00192:51:H2KG3CCXX:3:2216:2827:37471 -  AAGAGTGAcacTccATcTCAAAAAAAAAAAAATTAgAAATTTTAAAagTTTTCTAATTTTCTAATTCTTAATGTGTTGAGTAATTTTTTTATTGTATTCTGGACATCTTACACACTATGCTCTGTGaATCTGAGTCTTGATAAAATGCTATA
ST-E00192:51:H2KG3CCXX:3:2209:18630:16709-  AcActccatctcAAAAAAAAAAAAATTAGAAATTTTAAAAGTTTTCTAATTTTCTAATTCTTAATGTGTTGAGTAATTTTTTTATTGTATTCTGGACATCTTACACACTATGCTCTGTGAATCTGAGTCTTGATAAAATGCTATAGAGTTTT
ST-E00192:51:H2KG3CCXX:2:2221:8277:28418 -  cactcCatcTCAAAAAAAAAAAAATTAGAAATTTTAAAAGTTTTCTAATTTTCTAATTCTTaATGTGTTGAGTAATTTTTTTATTGTATTCTGGACATCTTACACACTATGCTCTGTGAATCTGAGTCTTGATAAAATGCTATAGAGTTTTT
ST-E00192:51:H2KG3CCXX:2:2221:8440:28558 -  CActacATcTCAAAAAAAAAAAAATTAGAAATTTTAAAAGTTTTCTAATTTTCTAATTCTTAATGTGTTGAGTAATTTTTTTATTGTATTCTGGACATCTTACACACTATGCTCTGTGAATCTGAGTCTTGATAAAATGCTATAGAGTTTTT
ST-E00192:51:H2KG3CCXX:8:2123:11424:22651-  caatacAAAAaaaaaaaatTataaaTTTtaaaAgTTTTCTAATTTtctAattCtTAatgTGTTGagtAATTTTTTTATTGTAtTcTgtAcaTCTTACACaCTATGCTCTGTGAATCTGAGTCTTgATAAAATGCTATAGAGTTTTTTTTA
ST-E00192:51:H2KG3CCXX:1:1201:8907:44345 -  cAcAacAAaAAATTAGAAATTTTAAAAGTTTTCTAATTTTCTAATTCTTAATGTGTTGAGTAATTTTTTTATTGTATTCTGGACATCTTACACACTATGCTCTGTGAATCTGAGTCTTGATAAAATGCTATAGAGTTTTTTTTATTTAAC
ST-E00192:51:H2KG3CCXX:3:2209:13423:29736-  AAATtagaAATTTtAAAAatatatAATTTtcTAATTcTtAATGTgTTGagTAATTTTTTTATTGTATTCTGGACATCTTactcacTtTGCTCTGTGAATCTGAGTCTTGATAAAATGCTATAGAGTTTTTTTTATTTAACAGGTAGTAA
ST-E00192:51:H2KG3CCXX:1:2109:19970:39071-  CTAATTCTTAATGTGTtGAGTAATTTTTTTATTGTATTCTGGACATCTTaCacACTATGCTCTGTGAATCTGAGTCTTGATAAAATGCTATAGAGTTTTTTTTATTTAACAGGTAGTAACCTTGCTAGAGTCAGCCACAAATTCCTACCG
ST-E00192:51:H2KG3CCXX:8:2223:19290:20383-  AaTtaTcaAAataaaAatgTgTTGAGTAATTTTTTTATTgTaTTCTgGAcaTcTTACACACTATGCTcTGtGAATCTGAGTCTTGATAAAATGCTATAGAGTTTTTTTTATTTAACAGGTAGTAAcCTTGCTaGaGTCAGcCa
ST-E00192:51:H2KG3CCXX:1:1121:29866:37050-  TGAGTAATTTTTTTATTGTATTCTGGACATCTTACACACTATGCTCTGTGAATCTGAGTCTTGATAAAATGCTATAGAGTTTTTTTTATTTAACAGGTAGTAACCTTGCTAGAGTCAGCCACAAATTCCTACCGACCTTCTGTTTGCATT
ST-E00192:51:H2KG3CCXX:2:1215:25380:34729-  AttaTggAcatcTtAtACaCTaTgCTcTgTgAAtcTGAGTCTTGATAAAATGCTATAGAGTTTTTTTTATTTAACaGGTAGTAACCTTGCTAGAGTCAGCCACAAATTCCTACCGACCTTCTGTT
ST-E00192:51:H2KG3CCXX:3:2104:26121:6688 -  ACACacTatgctctgTgAAtctgAatCTTGATAAAATGCTATAGAGTTTTTTTTATTTAACAGGTAGTAACCTTGCTAGAGTCAGCCACAAATTCCTACCGACCTTCTGTTTGCATTGGTTCATTCTAGTGCCAATTCTGTTTACAAGG
ST-E00192:51:H2KG3CCXX:1:1104:4339:12279 -  ATGCTCtGTgAAtcTGAGTcTTGATAAAATGCTATAGAGTTTTTTTTATTTAACAGGTAGTAACCTTGCTAGAGTCAGCCACAAATTCCTACCGACCTTCTGTTTGCATTGGTTCATTCTAGTGCCAATTCTGTTTACAAGGCCCTTAT
```

```
                 85976520  85976540  85976560  85976580  85976600  85976620  85976640  85976660  85976680  85976700  85976720  85976740  85976760  85976780
chr8:85976507-85976798  cacacctgacctaaaacctaaatgccttattttcttctgcagtgccacttgaccccaatacaaactcgacagtggttccaaacagccagaaaatggcactttcaatttttccatcctacaagatctaaataattcttgtcataaaataggcaaacggtctgaggtgcctgacatccaggcattcttttacacatcagtccctccctagtctctgttcccaatgcaacttgtcccaaatcatccgtctttccctccagcctatcccctcagtcccaaacccaagcattgctga

ST-E00144:68:H2LCKCCXX:8:2207:2004:9712  +  ACACCTGACCTAAAACCTAAATGCCTTATTTTCTTCTGCAGTGCCACTTGACCCCAATACAAACTCGACAGTGGttCCAAACAGCCAGAAAATGGCACTTTCAATTTTTCCATCcTACAAgATCTaAATAATTcTTgTactaAAAtaggc
ST-E00144:68:H2LCKCCXX:8:2212:13322:42077+  ACACCTGACCTAAAACCTAAATGCCTTATTTTCTTCTGCAGTGCCACTTGACCCCAATACAAACTCGACAGTGGTTCCAAACAGCCAGAAAATGGCACTTTCAATTTTTCCATCCTACAAGATCTAAATAATTCTTGTCatAAAATAGGC
ST-E00144:68:H2LCKCCXX:1:2120:25624:33006+  CTGACCTAAAACCTAAATGCCTTATTTTCTTCTGCAGTGCCACTTGACCCCAATACAAACTCGaCAGTGGTTCCAAACAGCCAGAAAAtGGCACTTTCAATTTTTCCaTCCTACAAGATCTAAATAATTCTTGTCATAAAATAGGCAAac
ST-E00144:68:H2LCKCCXX:1:2120:25654:33059+  CTGACCTAAAACCTAAATGCCTTATTTTCTTCTGCAGTGCCACTTGACCCCAATACAAACTCGACAGTGGTTCCAAACAGCCAGAAAAtGGCACTTTCAATTTTTCCATCCTACAAGaTCTAAATAATTCTTGTCATAAAATaGGCAAcc
ST-E00144:68:H2LCKCCXX:4:2222:25045:5352 +  GACCTAAAACCTAAATGCCTTATTTTCTTCTGCAGTGCCACTTGACCCCAATACAAACTCGACAGTGGTTCCAAACAGCCAGAAAATGGCACTTTCAATTTTTCCATCCTACAAGATCTAAATAATTCTTGTCATAAAATAGGCAAACGG
ST-E00144:68:H2LCKCCXX:6:2120:5121:19961 +  ACCTAAATGCCTTATTTTCTTCTGCAGTGCCACTTGACCCCAATACAAACTCGACAGTGGTTCCAAACAGCCAGAAAATGGCACTTTCAATTTTTCCATCCTACAAGATCTAAATAATTCTTGTCATAAAATAtGCAAACGGTcTGAggT
ST-E00144:68:H2LCKCCXX:7:1117:8480:10872 +  ACCTAAATGCCTTATTTTCTTCTGCAGTGCCACTTGACCCCAATACAAACTCGACAGTGGTTCCAAACAGCCAGAAAATGGCACTTTCAATTTTTCCATCCTACAAGATCTAAATAATTCTTGTCATAAAATAGGCAAACGGTCTGAGGT
ST-E00144:68:H2LCKCCXX:6:2102:10053:53856+  ATGCCTTATTTTCTTCTGCAGTGCCACTTGACCCCAATACAAACTCGACAGTGGTTCCAAACAGCCAGAAAATGGCACTTTCAATTTTTCCATCCTACAAGATCTAAATAATTCTTGTCATAAAATAGGCAAACGGTCTGAGGTGCCTGA
ST-E00144:68:H2LCKCCXX:8:2108:32150:53768+  TaTTTTcTTCTGCAGTGCCACTTGACCCCAAtACAAACTCGACAGTGGTtCCAAACAGCCAGAAAATGGCACTTTCAAtTTTTCCATCCTaCAAGaTCTAAATAAtTCTTGTCATAAAATAGGCAAACGGTCTgAGGTGccTGAcaTCCA
ST-E00144:68:H2LCKCCXX:8:2211:31622:68782+  GCAGTGCCACTTGACCCCAATACAAACTCGACAGTGGTTCCAAACAGCCAGAAAATGGCACTTTCAATTTTTCCATCCTACAAGATCtAAATAATtCTTGTCAtAAAATAGGCAAACGGTCtGAGGtGCCTGACatCCAGgCattcTTTT
ST-E00144:68:H2LCKCCXX:3:2122:10561:51114+  AGTGCCACTTGACCCCAATACAAACTCGACAGTGGTTCCAAACAGCCAGAAAATGGCACTTTCAATTTTTCCATCCTACAAGATCtAAATAATTCTTGTCATAAAATAGGCAAACGGTCTGAGGTGCCTGACATCCAgGCATTCTTTTAC
ST-E00144:68:H2LCKCCXX:4:1112:5933:3858  +  CCACTTGACCCCAATACAAACTCGACAGTGGTTCCAAACAGCCAGAAAATGGCACTTTCAATTTTTCCATCCTACAAGATCTAAATAATTCTTGTCATAAAATAGgCAAACGGTCTGAGGtGCCTGACATCCAGGCaTTCTTTTAcacaT
ST-E00144:68:H2LCKCCXX:8:2206:28080:17957+  TCGACAGTGGTTCCAAACAGCCAGAAAATGGCACTTTCAATTTTTCCATCCTACAAGATCTAAATAATTCTTGTCATAAAATAGGCAAACGGTCTGAGGTGCCTGACATCCAGGCATTCTTTTACACATCAGTCCCTCcCTAGTCTCTGT
ST-E00144:68:H2LCKCCXX:1:2222:19168:14969+  AAACAGCCAGAAAATGGCACTTTCAATTTTTCCATCCTACAAGATCTAAATAATTCTTGTCATAAAATAGGCAAACGGTCTGAGGTGCCTGACATCCAGGCATTCTTTTACACATCAGTCCCTCCCTAGTCTCTGTTCCCAATGCAACTT
ST-E00144:68:H2LCKCCXX:8:1111:24111:68395+  AAACAGCCAGAAAATGGCACTTTCAATTTTTCCATCCTACAAGATCTAAATAATTCTTGTCATAAAATAGGCAAACGGTCTGAGGTGCCTGACATCCAGGCATTCTTTTACACATCAGTCCCTCCCTAGTCTCTGTTCCCAATGCAACTT
ST-E00144:68:H2LCKCCXX:8:1224:26050:30896+  AAACAGCCAGAAAATGGCACTTTCAATTTTTCCATCCTACAAGATCTAAATAATTCTTGTCATAAAATAGGCAAACGGTCTGAgGTgCCTGACATCCAGGCATTCTTTTACacATCAgtCaCTCCCcActctCTGTTcCCAATgaaACtt
ST-E00144:68:H2LCKCCXX:1:1222:9627:9730  +  CCAGAAAATGGCACTTTCAATTTTTccaTCcTatAaGATCTAAATAATTCTTGTcATAAAATAGGCAaACGGTCTGAGGTGCctGACaTCcaGgCatTCTTTTAcACATCAGTCCCTCcCTaGTCTCTGTTcccaaTGCAActTGTcCCt
ST-E00144:68:H2LCKCCXX:8:1208:21482:52872+  TTTCAATTTTTCCATCCTACAAGATCTAAATAATTCTTGTCATAAAATAGGCAAACGGTCTGAGGTGCCTGACATCCAGgCATTCTTTTACACATCAGTCCCTCCCTAGTCTCTGTTCCCAATGCAACTTGTCcCAaatCATCCGtCTTt
ST-E00144:68:H2LCKCCXX:1:1222:23147:3084 +  TTCCATCCTAcaAGATCTaAATAATTCTTGTCATAAcATAGgCAAACGGtctGaGGTGCcTGACATCCAGGCaTtCTTTTACaCaTccgTCCctCCCTaGTCTCTGTTCccaATGCaactaGtcCCaAaTcATCcGTcTTTCCC
ST-E00144:68:H2LCKCCXX:6:2222:14804:70804+  TTCCATCCTACAAGATCTAAATAATTCTTGTCATAAAATAGGCAAACGGTCTGAGGTGCCTGACATCCAGGCATTCTTTTACACATCAGTCCCTCCCTAGTCTCTGTTCCCAATGCAACTTGTCCCAAATCATCCGTCTTTCCcTCCAGC
```

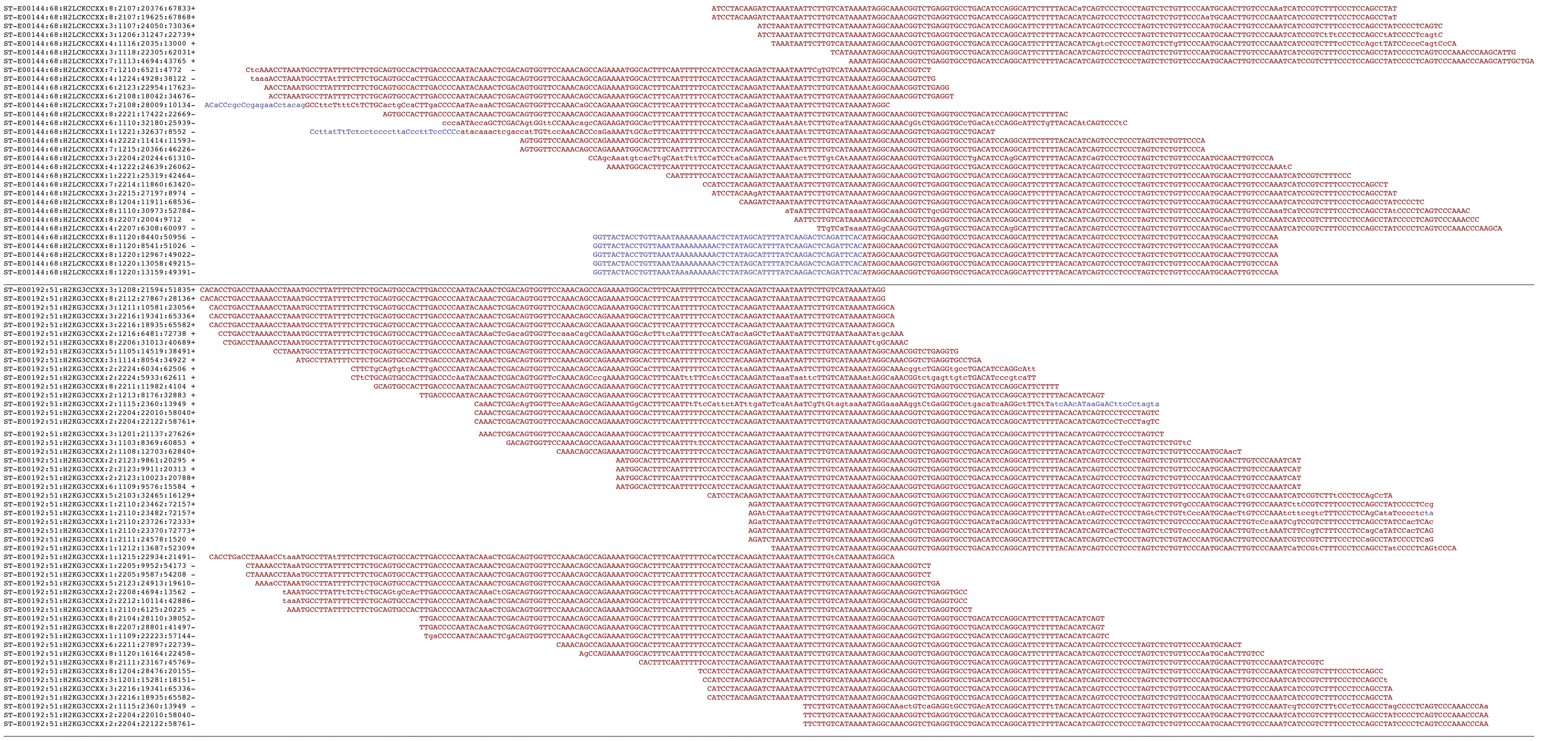

chr1 16940961 + 3 chr2 52196826 + 1 CTX 166 34 73 78 0.979450980392157 0 0.945641025641026 0 1 chr1 16940885 150 chr2 52196897

TTCCAAATTTTCCTTGAATCTGTCCTCTTCTATCTCCTCCGTCACCACCCTAGTCTAGGCTGCCTTCACCTGGGGAGCCAACTATGAATACACTGTGCATTCACTGCTGGGTGAAAATAGAGCTGTGTGCATTAAATATTTCTCCTTTGA

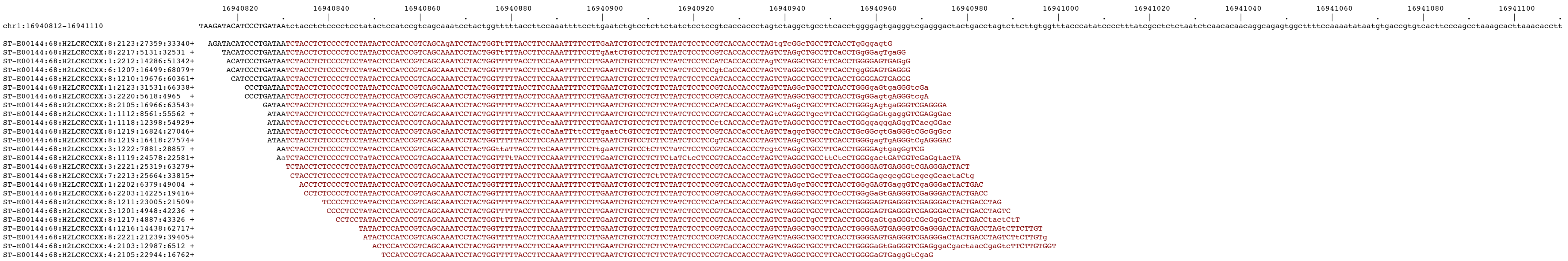

```
ST-E00144:68:H2LCKCCXX:1:2111:3568:36434 + CCGTCAGCAAATCCTACTAgTTTTTACCTTCCAAATTTTCCTTGAATCTGTCgTCTTCTATCTCCTCCATCACCACCCTAGTgTAGGCTGCcTTCACCTGGGGAgTGAGGGTCgAGGGACTaCTGACcTAGTCTTCTTGTGcTTTgcccA
ST-E00144:68:H2LCKCCXX:1:1203:21594:46912+ GTCAGCAAATCCTACTGGTTTTTACCTTCCAAATTTTCCTTGAATCTGTCCTCTTCTATCTCCTCCGTCACCACCCTAGTCTAGGCTGCCTTCACCTGGGGAGTGAGGGTCGAGGGACTACTGACCTAGTCTTCTTGTGGTTTACCCATA
ST-E00144:68:H2LCKCCXX:1:1203:21645:47246+ GTCAGCAAATCCTACTGGTTTTTACCTTCCAAATTTTCCTTGAATCTGTCCTCTTCTATCTCCTCCGTCACCACCCTAGTCTAGGCTGCCTTCACCTGGGGAGTGAGGGTCGAGGGACTACTGACCTAGTCTTCTTGTGGTTTACCCaT
ST-E00144:68:H2LCKCCXX:3:2109:23654:24146+ GTCAGCAAATCCTACTGGTTTTTACCTTCCAAATTTTCCTTGAATCTGTCCTCTTCTATCTCCTCCGTCACCACCCTagTCTAGGCTGCCTTCACCTGGGGAGTGagGGTCGAGGgacTACTGACCTAGTCTTCTTGTGgTTTACCcATA
ST-E00144:68:H2LCKCCXX:4:2224:18265:16639+ CAGCAAATCCTACTGGTTTTTACCTTCCAAATTTTCCTTGAATCTGTCCTCTTCTATCTCCTCCATCACCACCCTAGTCTAGGCTGCCTTCACCTGGGgagTGaGgGTCGAGGGACTACTGaCCTAggctTcTTGTGGtTTACCCaTaTC
ST-E00144:68:H2LCKCCXX:8:2108:22599:47211+ AGCAAATCCTACTGGTTTTTACCTTCCAAATTTTCCTTGAATCTGTCCTCTTCTATCTCCTCCGTCACCACCCTAGTCTAGGCTGCCTTCACCTGGGGAGTGAGGGTCGAGGGACTACTGACCTAGTCTTCTTGTGGTTTACCCATATCC
ST-E00144:68:H2LCKCCXX:1:2117:10744:60431+ CAAATCCTACTGGTTTTTACCTTCCAAATTTTCCATGAATCTGTCCTCTTCTATCTCCTCCATCaCCACCCTAGTCTAGGCTGCCTTCACCTGgggAgTGAGGGTCGAGGGACTACTGACCTAGTCTTCTTGTGGTctaCCcatatcCCC
ST-E00144:68:H2LCKCCXX:4:1116:22518:44715+ AAATCCTACTGGtTTTTACCTTCCAAATTTTCCTTGAATCTGTCcTCTtcTATcTCCTCCGTCACCACCCTAGTCTAGGCTgCCTTCACCTGGGgagTgAGGGtcgAGGgacTACtGACcTagTctTcTTGTGGTtTAcCCATATCCCCT
ST-E00144:68:H2LCKCCXX:6:2214:4826:22229 + AAATCCTACTGGTTTTTACCTTCCAAATTTTCCTTGAATCTGTCCTCTTCTATCTCCTCCGTCACCACCCTAGTCTAGGCTGCCTTCACCTGGGGAGTGAGGGGcGAGGgACTACTGACCTaGtCTTCTTGTggtTTACCCATATccCCT
ST-E00144:68:H2LCKCCXX:3:1108:14448:26132+ AATCCTACTGGTTTTTACCTTCCAAATTTTCCTTGAATCTGTCCTCTTCTATCTCCTCCGTCACCACCCTAGTCTAGGCTGCCTTCACCTGGGGAGTGAGGGTCGAGGGACTACTGACCTAGTCTTCTTGTGGTTTACCCATATCCCCTT
ST-E00144:68:H2LCKCCXX:1:2122:16478:69872+ CCTACTGGTTTTTACCTTCCAAATTTTCCTTGAATCTGTCCTCTTCTATCTCCTCCATCACCACCCTAGTCTAGGTTGCCTTCACCTGGGGAGTGAGGGTCGAGGGACTACTGACCTAGTCTTCTTGTGGTTTACCCATATCCCCTTTAT
ST-E00144:68:H2LCKCCXX:6:2210:6897:19381 + ACTGGTTTTTACCTTCCAAATTTTCCTTGAATCTGTCCTCTTCTATCTCCTCCGTCACCACCCTAGTCTAGGCTGCCTTCACCTGGGGAGTGAGGGTCGAGGgaCTACTGACCTAGtCTTCTTGTGgTTTACCCATATCCCCTTtaTCgc
ST-E00144:68:H2LCKCCXX:1:2210:6034:30545 + TGGTTTTTACCTTCCAAATTTTCCTTGAATCTGTCCTCTTCTATCTCCTCCATCACCACCCTAGTCTAGGCTGCCTTCACCTGGGGAGTGAGGGTCGAGGGACTACTGACCTAGTCTTCTTGTGgTTTACCCATATCCcCTTTATgGCCT
ST-E00144:68:H2LCKCCXX:1:2217:7019:39001 + TGGTTTTTACCTTCCAAATTTTCCTTGAATCTGTCCTCTTCTATCTCCTCCGTCACCACCCTAGTCTAGGCTGCCTTCACCTGGGGAGTGAGGGTCGAGGGACTACTGACCTAGTCTTCTTGTGGTTTACCCATATCCCCTTTATCGCCT
ST-E00144:68:H2LCKCCXX:1:2217:6988:39018 + TGGTTTTTACCTTCCAAATTTTCCTTGAATCTgTCCTCTTCTATCTCCTCCGTCACCACCCTAGTCTAGGCTGCCTTCACCTGGGGAGTGAGGGTCGAGGGACTACTGACCTAGTCTTCTTGTGGTTTACCCATATCCCCTTTATCGCCT
ST-E00144:68:H2LCKCCXX:1:1202:10815:66075+ TTTTACCTTCCAAATTTTCCTTGAATCTGTCCTCTTCTATCTCCTCCATCACCACCCTAGTCTAGGCTGCCTTCACCTGGGGAGTGAGGGTCGAGGGACTACTGACCTAGTCTTCTTGTGGTTTACCCATATCCCCTTTATCGCCTCTCT
ST-E00144:68:H2LCKCCXX:6:1112:1720:58234 + TTtTACCTTCCAAATTTTCCTTGAATCTGTCCTCTTCTATCTCCTCCGTCACCACCCTAGTCTAGGCTGCCTTCACCTGGGGAGTGAGGGTCGAGGgACTACTGACCTAGTCTTCTTGTGGgtTtACcCATATCCCCtTTATCGCCtcTCT
ST-E00144:68:H2LCKCCXX:6:2224:20163:54384+ TTTTACCTTCCAAATTTTCCTTgAATCTGTCCTCtTCTATCTCCTCCGTCACCACCCTAGTCTAGGCTGCCTTCACCTGGGGAgTGAGGGTCGAGGGACTACTGACCTAGTCTTCTTGTGGggTaCCCATATCCCCTTTATCGCCTCTCT
ST-E00144:68:H2LCKCCXX:8:1219:18316:59623+ TTTTACCTTCCAAATTTTCCTTGAATCTGTCCTCTTCTATCTCCTCCGTCACCACCCTAGTCTAGGCTGCCTTCACCTGGGGAGtGAGGGTCGAGGGACTACTGACCTAGTCTTCTTGTGGtTTACCCATATCCCCTTTATCGCcTCTcT
ST-E00144:68:H2LCKCCXX:6:2119:27451:31107+ TTTACCTTCCAAATTTTCCTTGAATCTGTCCTCTTCTATCTCCTCCGTCACCACCCTAGTCTAGGCTGCCTTCACCTGGGGcGtGAGGGtCGAGGgactaCTGACCTAGcCTtCtTGTGGttTgCCCATATCCcCTttATCGCCTcTCTc
ST-E00144:68:H2LCKCCXX:1:2116:30810:22440+ TTACCTTCCAAATTTTCCTTGAATCTGTCCTCTTCTATCTCCTCCGTCACCACCCTAGTCTAGGCTGCCTTCACCTGGGGAGTGAGGGTCGAGGGACTACTGACCTAGTCTTCTTGTGGTTTACCCATATCCCCTTTATCGCCTCTCTAA
ST-E00144:68:H2LCKCCXX:1:2101:10216:39054+ TACCTTCCAAATTTTCCTTGAATCTGTCCTCTTCTATCTCCTCCGTCACCACCCTAGTCTAGGCTGCCTTCACCTGGGGAGTGAGGGTCGAGGGACTACTGACCTAGTCTTCTTGTGGTTTACCCATATCCCCTTTATCGCCTcTCTAAT
ST-E00144:68:H2LCKCCXX:7:2213:10094:53364+ ACCTTCCAAATTTTCCTTGAATcTGTCcTCTTCTATCTCCTCCGTCACCACCCTAGTCTAGGCTGCCTTCACCTGGGtAGTGAGGGTCGAGGGAcTACTGACCTaGTCTTCTTGTGGTTTACCCaTAtcCCCtttaTCGcctCTcTAaTC
ST-E00144:68:H2LCKCCXX:7:1104:17869:11312+ CTTCCAAATTTTCCTTGAATCTGTCCTCTTCTATCTCCTCCGTCACCACCCTAGTCTAGGCTGCCTTCACCTGGGGAGTGAGGGTCGAGGGACTACTGACCTAGTCTTCTTGTGGTTTACCCATATCCCCTTTATCGCCTCTCTAATCTC
ST-E00144:68:H2LCKCCXX:7:1104:17839:11330+ CTTCCAAATTTTCCTTGAATCTGTCCTCTTCTATCTCCTCCGTCACCACCCTAGTCTAGGCTGCCTTCACCTGGGGAGTGAGGGTCGAGGGACTACTGACCTAGTCTTCTTGTGGTTTACCCATATCCCCTTTATCGCCTCTCTAaTCTC
ST-E00144:68:H2LCKCCXX:8:2110:32404:6600 + CTTCCAAATTTTCCTTGAATCTGTCCTCTTCTATCTCCTCCGTCACCACCCTAGTCTAGGCTGCCTTCACCTGGGGAGtGAGGGTCGAGGGACTACTGACCTAGTCTTCTTGTGGTTTaCCCaTATCCCCTTTaTCGCCTCTCtAAtCTC
ST-E00144:68:H2LCKCCXX:4:1219:27268:29771+ AATTTTCCTTGAATCTGTCCTCTTCTATCTCCTCCATCACCaCCCTAGTCTAGGCTGCCTTCaCCTGGGGAGtgAGGGTCGaGGGaCTACTGaCCTAGTCTTCTTGTGGttTACcCATatCCCCTTcATcGCctCTCTAATCTCAACACA
ST-E00144:68:H2LCKCCXX:1:1210:9668:45523 + ATTTTCCTTGAATCTGTCCTCTTCTATCTCCTCCGTCACCACCCTAGTCTAGGCTGCCTTCACCTGGGGAGTGAGGGTCGAGGGaCTACTGACCTAGTCTTCTTGTGGTTTACCCATATCCCCTTTATCGCCTCTCTaATCTCAACACAA
ST-E00144:68:H2LCKCCXX:4:2205:6613:72966 + ATTTTCCTTGAATCTGTCCTCTTCTATCTCCTCCGTCACCACCCTAGTCTAGGCTGCCTTCACCTGGGGAGTGAGGGTCGAGGGACTACTGACCTAGTCTTCTTGTGGTTTACCCATATCCCCtTTATCgCCTCTCTAAtCTCAACACAA
ST-E00144:68:H2LCKCCXX:7:2111:27583:72404+ ATTTTCCTTGAATCTGTCCTCTTCTATCTCCTCCGTCACCACCCTAGTCTAGGCTGCCTTCACCTGGGGAGTGAGGGTCGAGGGACTACTGACCTAGTCTTCTTGTGGTTTACCCATATCCCCTTTATCGCCTCTCTAATCTCAACACAA
ST-E00144:68:H2LCKCCXX:7:2111:27329:72456+ ATTTTCCTTGAATCTgTCCTCTTCTATCTCCTCCgtCACCACCCTAGTCTAgGCTGCCTTCACCTGGGGAGTGAGGGTCGAGGGACTACTGACCTAGTCTTCTTGTGGTTTACCCATATCCCCTTTATCGCCTCTCTAATCTCAACaCAA
ST-E00144:68:H2LCKCCXX:7:2213:21127:16357+ ATTTTCCTTGAATCTGTCCTCTTCTATCTCCTCCGTCACCACCCTAGTCTAGGCTGCCTTCACCTGGGGAGTGAGGGtCGAGGGaCTaCTGACCTaGTCTTCTTGTGGTTTACCCATATCCCCTtTATCGCCTCTCTAATCTCAACACAA
ST-E00144:68:H2LCKCCXX:7:1115:20021:13000+ TTTCCTTGAATCTGTCCTCTTCTATCTCCTCCGTCACCACCCTAGTCTAGGCTGCCTTCACCTGGGGAGTGAgGGTCGAGGGACTACTGACCTAGTCTTCTTGTGGTTTACCCATATCCCCTTTATCGCCTCTCTAaTCTCAACACAACA
ST-E00144:68:H2LCKCCXX:7:1224:22660:20383+ TTCcTTGAATCTGTCCTCTTCTATCTCCTCCGTCaCCACCCTAGTCTAGGCTgcCTTCACCTGGgGaGTGAGGGTCGAGGGACTACTGACcTaGTCTTCTTGTGGTTTAcccaTATCCCcTGtATCGcCtctctAaTctaaACAaaACAG
ST-E00144:68:H2LCKCCXX:1:1112:7140:67692 + CCTTGAATCTGTCCTCTTCTATCTCCTCCGTCACCACCCTAGTCTAGGCTGCCTTCACCTGGGGAGtGaGGGTCGAGGGACTACTGACCTAGTCTTCTTGTGGTTTACcCaTATCCCCTTTATCGCCTCTCTAaTCTCAACACAACAGgc
ST-E00144:68:H2LCKCCXX:1:2215:15595:62611+ TGAATCTGTCCTCTTCTATCTCCTCCATCACCACCCTAGTCTAGGCTGCCTTCACCTGGGGAGTGAGGGTCGAGGGACTACTGACCTAGTCTTCTTGTGGTTTACCCATATCCCCTTTATCGCCTCTCTAATCTCAACACAACAGGCAGA
ST-E00144:68:H2LCKCCXX:1:1218:7242:19100 + AATCTGTCCTCTTCTATCTCCTCCATCACCACCCTAGTCTAGGCTGCCTTCACCTGGGGAGTGAGGGTCGAGGGACTACTGACCTAGTCTTCTTGTGGTttACCCATATCCCCTTTATCGCCTCTCTcaTCTcaaCACAACAGGCAGAGT
ST-E00144:68:H2LCKCCXX:1:2207:8551:33498 + ATCTGTCCTCTTCTATCTCCTCCATCACCACCCTAGTCTAGGCTGCCTTCACCTGGGGAGTGAGGGTCGAGGGACTACTGACCTAGTCTTCTTGTGGTTTACCCATATCCCCTTTATCGCCTCTCTAATCTCAACACAACAGGCAGAGTG
ST-E00144:68:H2LCKCCXX:1:2214:19980:47246+ ATCTGTCCTCTTCTATCTCCTCCGTCACCACCCTAGTCTAGGCTGCCTTCACCTGGGGAGTGAGGGTCGAGGGaCTACTGACCTAGTCTTCTTGTGGTTTACCCATaTCCCCTTTATCGCCTCTCTACTCTCAacacaaCAGGCaGAgTG
ST-E00144:68:H2LCKCCXX:1:2214:19950:47299+ ATCTGTCCTCTTCTCTCTCcTCCGTCACCACCCTAGTCTaGGCTGCCTTCACCTGGtgAGTGAGGtTCGAGGGACtACTGACCTAGTCTTCTTGTGGTTTACCCATATCCCCTTTATCGCCTCTCTAATCTCAaCACaACaGGCaGaGTG
ST-E00144:68:H2LCKCCXX:7:1205:21015:3752 + ATCTGTCCTCTTCTATCTCCTCCGTCACCACCCTAGTCTAGGCTGCCTTCACCTGGGGAGTGAGGGTCGAGGGACTACTGACCTAGTCTTCTTGTGGTTTACCCATATCCCCTTTATCGCCTCTCTAATCTCAACACAACAGGCAGAGTG
ST-E00144:68:H2LCKCCXX:8:2213:18711:44767+ TGTCCTCTTCTATCTCCTCCGTCACCACCCTAGTCTAGGCTGCCTTCACCTGGGGaGTGAGGGTCGAGGGACTACTGACCTAGTCTTCTTGTGGTTTACCCATaTCCCCTTTATCGCCTCTCTAATCTCAACACAACAGGCAGcGtgGCt
ST-E00144:68:H2LCKCCXX:1:1113:29653:3524 + TCTTCTATCTCCTCCGTCACCACCCTAGTCTAGGCTGCCTTCACCTGGGGAGTGAGGGTCGAGGGACTACTGACCTAGTCTTCTTGTGGTTTACCCATATCCCCTTTATCGCCTCTCTAATCTCAACACAACAGGCAGAGTGGCTTTTCC
ST-E00144:68:H2LCKCCXX:1:2112:10764:20911+ CTTCTATCTCCTCCGTCACCACCCTAGTCTAGGCTGCCTTCACCTGGGGAGTGAGGGTCGAGGGACTACTGACCTAGTCTTCTTGTGGTTTACCCATATCCCCTTTATCGCCTCTCTAATCTaaACACAACaGGCAGAGTGGCTtTTCCA
ST-E00144:68:H2LCKCCXX:4:1214:5029:5387  + ATCTCCTCCGTCACCACCCTAGTCTAGGCTGCCTTCACCTGGGGAGTGAGGGTCGAGGGACTACTGACCTAGTCTTCTTGTGGTTTACCCATATCCCCTTTATCGCCTCTCTAATCTCAACACAACAGGCAGAGTGGCTTTTCCAAAATA
ST-E00144:68:H2LCKCCXX:6:1214:24426:26431+ CTCCTCCGTCACCACCCTAGTCTAGGCTGCCTTCACCTGGGGAGTGAGGGTCGAGGGACTACTGACCTAGTCTTCTTGTGGTTTACCCATATCCCCTTTATCGCCTCTCTAATCTCAACACAACAGGCAGAgTGGCTTTTCCAaAATATA
ST-E00144:68:H2LCKCCXX:8:2202:24223:44363+ CTCCTCCATCACCACCCTAGTCTAGGCTGCCTTCACCTGGGGAGTGAGGGTCGAGGGACTACTGACCTAGTCTTCTTGTGGTttACCCATATCCCCTTTATCGCCTCTCTcaTCTCAACACAACAGGCAGAGTGGCTGTTCCAAAATaTA
ST-E00144:68:H2LCKCCXX:3:2115:19747:3946 + CCTCCATCACCACCCTAGTCTAGGCTGCCTTCACCTGGGGAGTGAGGGTCGAGGGACTACTGaCCTAGtCTTCTTGTGGTTTACCCATATCCCCTTTATCGCCTCTCTAcTCTCAACACAACAGGCAGcgTggCTTTTCCAAAATAaaAT
ST-E00144:68:H2LCKCCXX:1:1224:5618:31828 + CTCCATCACCACCCTAGTCTAGGCTGCCTTCACCTGGGGAGTGAGGGTCGAGGGACTACTGACcTAGTCTTCTTGTGGTTTACCCATATCCCCTTTATCGCCTCTCTAAtcTcaacACAACAGGCAGAGTGGcTTTtCCaAcaTATAtTG
ST-E00144:68:H2LCKCCXX:1:1108:16712:3119 + GTCACCACCCTAGTCTAGGCTGCCTTCACCTGGGGaGTGAGGGTCGAGGGACTACTGACCTaGtCTTCTTGTGGTTTACCCATATCCCCTTTATCGCCTCTCTAATCTCAACACAACaGgCAGAGtGGCTTTTCCAaaataTAATgTGAC
ST-E00144:68:H2LCKCCXX:1:1108:16651:3295 + GTCACCACCCTAGTCTAGGCTGCCTTCACCTGGGGAGTGAGGGTCGAGGGACTACTGACCTAGTCtTCTTGTGGTTTACCCATATCCCCTTTATCGCCTCTCTAATCTCAACACAACAGGCAGAGTGGCTTTTCCAAAATATAATGTGAC
ST-E00144:68:H2LCKCCXX:6:2104:29115:59869+ ATCAcCACcCTAGTCTAGGCTGCCTTCaCCTGGGGAGTGAGGGTCGAGGgACTACTGACCTAGTCTTCtTGTGGTTTACCCATATCACAtTTATagaCtCTCTAATaTCAACACAACAGGCAGAGTGGCtTTTcCAAAATAtAATGTgac
ST-E00144:68:H2LCKCCXX:7:2118:26933:9782 + CACCACCCTAGTCTAGGCTGCCTTCACCTGGGGAGTGAGGGTCGAGGGACTACTGACCTAGTCTTCTTGTGGTTTACCCATATCCCCTTTATCGCCTCTCTAATCTCAACACAACAGGCaGAGTGGCTTTTCCAAAATAtAaTGTGACCG
ST-E00144:68:H2LCKCCXX:3:1209:8876:24919 + ACCACCCTAGTCTAGGCTGCCTTCACCTGGGGAGTGAGGGTCGAGGGACTACTGACCTAGTCTTCTTGTGGTTTACCCATATCCCCTTTATCGCCTCTCTAATCTCAACACAACAGGCAGAGTGGCTTTTCCAAAATATAATGTGACCGT
ST-E00144:68:H2LCKCCXX:4:2114:2258:54419 + GTCTAGGCTGCCTTCACCTGGGGAGTGAGGGTCGAGGGACTACTGACCTAGTCTTCTTGTGGTTTACCCATATCCCCTTTATCGCCTCTCTAATCTCAACACAACAGGCAGAGggGCtTTTCCAAAATATAATGTGAccGtctcacTtcC
ST-E00144:68:H2LCKCCXX:7:1210:27146:49883+ CTAGGCTGCCTTCACCTGGGGAGTgAGGGTCGAGGGACTACTGACCTAGTCTTCTTGTGGTTTACCCATATCCCCTtTaTCGCctcTCTAATCTCAACACaaCAGGCAGaGTGGCTTTTCCAAAATATAATGTGACCGTGtcACTTcCcA
ST-E00144:68:H2LCKCCXX:4:2114:14073:3963 + AGGCTGCCTTCACCTGGGGAGTGAGGGTCGAGGGACTACTGACCTAGTCTTCTTGTGGTTTACCCATATCCCCTtTATCGCCTCTCTAATCTCAACACAACAGGCAGAGTGGCTTTTCCAAAATATAATGTgaCcgtgTCaCTTCCCAcC
ST-E00144:68:H2LCKCCXX:8:2218:21624:13773+ GGCTGCCTTCACCTGGGGAGTGAGGGTCGAGGGACTACTGACCTAGTCTTCTTGTGGTTTACCCATATCCCCTTTATCGCCTCTCTAATCTCAACACAACAGGCAGAGTGGCTTTTCCAAAATATAATGTGACCGtgtCaCTTCCCAGCC
ST-E00144:68:H2LCKCCXX:8:2218:21584:13949+ GGCTGCCTTCACCTGGGGAGTGAGGGTCGAGGGACTACTGACCTAGTCTTCTTGTGGTtTACCCATATCCCCTTTATCGCCTCTCTAATCTCAACACAACAGGCAGAGTGGCTTTTCCAAAATATAATGTGACCGTGTCACTTCCCAGCC
ST-E00144:68:H2LCKCCXX:8:2218:21482:14054+ ggCTGCCTTCACCTGGGGAGTGAGGGTCGAGGGACTACTGACCTAGTCTTCTTgTGGTTTACCCATATCCCCTTTATCGCCTCTCTAATCTCAACACAACAGGCaGAGTGGCTTTTCCAAAATATAaTGTgACCGTgTCACTTCCCAGCC
ST-E00144:68:H2LCKCCXX:7:2211:31663:53804+ GCTGCCTTCACCTGGGGAGTGAGGGTCGAGGGACTACTGaCCTAGTCTTCTTGTGGTTTACCCATATCCCCTTTATCGCCTcTCtAATCTCAACACAAcAGGCAGAGTGGCTTTtCcAAAAtATAATGTGAccGTGtCActtcccagcct
ST-E00144:68:H2LCKCCXX:8:2222:26121:30808+ GCTGCCTTCACCTGGGGAGTGAGGGTCGAGGGACTACTGACCTAGTCTTCTTGTGGTTTACCCATATCCCCTTTATCGCCTCTCTAATCTCAACaCAACaGGCagaGTGGCTTTTCCAAAATATAAtGTGaCCGTgtCcCTtCCCcgcCT
ST-E00144:68:H2LCKCCXX:7:1124:23837:37050+ TTCACCTGGGGAGTGAGGGTCGAGGGACTACTGACCTAGTCTTCTTGTGGTTTACCCATATCCCCTTTATCGCCTCTCTAATCTCAACACAACAGGCAGAGTGGCTTTTCCAAAATATAATGTGACCGTGTCACTTCCCAGCCTAAAGCA
ST-E00144:68:H2LCKCCXX:6:1201:27725:64598+ ACCTGGGGAGTGAGGGTCGAGGGACTACTGACCTAGTCTTCTTGTGGTTTACCCATATCCCCTTTATCGCCTCTCTAATCTCAACACAACAGGCAGAGTGGCTTTTCCAAAATATAATgTGACCGTGTCACTTcCCAGCCTAAagcaCTT
ST-E00144:68:H2LCKCCXX:8:1119:13910:65037+ ACCTgGGGaGTGAGGGTCGagGGACTACTGACCTAGTCTTCTTGTGGcTtACCCAtaTcCCCTTTATCGccTcTCTAATCTCAaCACaACAGGCAGagtGGCTTTTCCAAAaTatAATGTGACCgTGTCcCtTCCCAgcCTAA
ST-E00144:68:H2LCKCCXX:4:2120:30759:58955+ TGGGGAGTGAGGGTCGAGGGACTACTGACCTAGTCTTCTTGTGGTTTACCCATATCCCCTTTATCGCcTCTCTcATCTCAACACAACAGGCAGAGTGGCTTTTCCAAaATATcAtgTGACcGTGTCACTTcCCAGCctAAAGCACtTAAA
ST-E00144:68:H2LCKCCXX:3:2117:4461:67868 + GGGGAGTGAGGGTCGAGGGACTACTGACCTAGTCTTCTTGTGGTTTACCCATATCCCCTTtATCGCCtCcctAAtCTCAACACAACAGGCAGAGtgGCTtTTCCAAActataatGTgaCCGTGccaCTtCCCAGCCtAaaGcaCttcaAC
ST-E00144:68:H2LCKCCXX:3:2217:8764:9114  + GGGGAGTGAGGGTCGAGGGACTACTGACCTAGTCTTCTTGTGGTTTACCCATATCCCCTTTATCGCCTCTCTAATCTCAACACAACAGGCAGAGTGGCTTTTCCAAAATATAATGTGACCGTGTCACTTCCCAGCCTAAAGCACttAAAC
ST-E00144:68:H2LCKCCXX:4:2212:20203:20594+ GGGAGTGAGGGTCGAGGGACTACTGACCTAGTCTTCTTGTGGTTTACCCATATCCCCTTTATCGCCtCtCTAATCTCAACACAACAGGCAGAGTGGCTTTTCCAAAATATAATgTGAcCGtgTCactgCCCAGCCtAAAGCACTtaAACA
ST-E00144:68:H2LCKCCXX:1:1119:22853:21139- TAAGATACaTCCCTGATAATCTACCTCTCCCCTCCTATACTCCATCCGTCAGCAAATCCTACTGGTTTTTACCTTCCAAATTTTCCTTGAATCTGTCCTCTTCTATCTCCTCCGTCACCACCCTAGTCTAGGCTGCCTTCACCTGGGGAG
ST-E00144:68:H2LCKCCXX:1:2112:11860:44855- TAAGATACATCCCTGATAATCTACCTCTCCCCTCCTATACTCCATCCGTCAGCAAATCCTACTGGTTTTTACCTTCCAAATTTTCCTTGAATCTGTCCTCTTCTATCTCCTCCATCACCACCCTAGTCTAGGCTGCCTTCACCTGGGGAG

ST-E00144:68:H2LCKCCXX:4:1216:11129:34026- aAGAtACAtcCCTGATAATCtgCctctCCCctCCtatactCCAtCCGTCAGCAAATCCTACTGGTTTTTACCTTCcAAATtTtCCTTGAATCTGTCCTCTTCTatCtCCtCCGTCAccaCCCTAGTCTAGGCTGCCTTCACCTGGGGAGt
ST-E00144:68:H2LCKCCXX:3:2108:28770:7057 - ATACATCCCTGATAATCTACCTCTCCCCTCCTATaCTCCATCCGTCAGCAAATCCTACTGGTTTTTACCTTCCAAATTTTCCTTGAATCTGTCCTCTTCTATCTCCTCCGTCACCACCCTAGTCTAGGCTGCCTTCACCTGGGGAGTGAG
ST-E00144:68:H2LCKCCXX:1:1123:25471:14037- TCCCTGATAATCTACCTCTCCCCTCCTATACTCCATCCGTCAGCAAATCCTACTGGTTTTTACCTTCCAAATTTTCCTTGAATCTGTCCTCTTCTATCTCCTCCGTCACCACCCTAGTCTAGGCTGCCTTCACCTGGGGAGTGAGGGTCG
ST-E00144:68:H2LCKCCXX:3:1117:20741:13263- TCCCTGATAATCTACCTCTCCCCTCCTATACTCCATCCGTCAGCAAATCCTACTGGTTTTTACCTTCCAAATTTTCCTTGAATCTGTCCTCTTCTATCTCCTCCGTCACCaCCCTAGTCTAGGCTGCCTTCACCTGGGGAGTGAGGGTCG
ST-E00144:68:H2LCKCCXX:8:1122:19574:37085- TGATAATCTACCTCTCCCCTCCTAtaCTCCATCCGTCAGCAAATCCTACTGGTTTTTACCTTCCAAATTTTCCTTGAATCTGTCCTCTTCTATCTCCTCCATCACCACCCTAGTCTAGGCTGCCTTCACCTGGGGAGTGAGGGTCGAGGG
ST-E00144:68:H2LCKCCXX:6:1219:8917:17500 - TAATCTACCTCTCCCCTCCTaTACTCCATCCGTCAGCAAATCCTACTGGTTTTTACCTTCCAAATTTTCCTTGAATCTGTCCTCTTCTATCTCCTCCGTCACCACCCTAGTCTAGGCTGCCTTCACCTGGGGAGTGAGGGTCGAGGGACT
ST-E00144:68:H2LCKCCXX:7:1104:30729:13298- TCTACCTCTCCCCTCCTATaCTCCATCCGTcaGCAAaTCCTaCTGGTTTTTACCTTCCaaaTTTTCCTTgAATCTGTCCTCTTCtATCTCCTCCGTccCCaCCCTaGTCTAGGCTGCCTTCACCTGGGGaGTgAggGTcgAgggaCTaCT
ST-E00144:68:H2LCKCCXX:1:2219:25096:37401- CtCCCtCcCCCCTCCTccACTCCATCCGTCAcCAAATCCTtCtgGcTTTTACCTtCCaactTTTCCttGAaTCTGtCCTCTTCTATCtCCTCCGtCACCaCCCTAgTcTAGGCTGcCTTCACCTGGGGAGTGAGGGTcGAGGGACTACT
ST-E00144:68:H2LCKCCXX:1:1202:7881:30017 - TAcCTCTCCCCTCCTAtACTCCATCCGTCAGCAcATCCTACTGGTTTtTACCTTCCcaATtTtCCTtGAATCTGTCCTCTtcTAtCtCCgCCGtCACCACCCTAGTCTAGGCTGCCTTCACCTGGGGAGTGaGGgTCGAGGGACTACTGA
ST-E00144:68:H2LCKCCXX:7:2124:3679:11277 - TACCTCTCCCCTCCTATCCTCCATCCGTCAGCAAATCCTACTGGTTTTTACCTTCCAAaTTTTCCTTGAATCTGTCCTCTTCTATCTCCTCCATCACCACCCTAGTCTAGGCTGCCTTCACCTGGGGAGTGAGGGTCGAGGGACTACTGA
ST-E00144:68:H2LCKCCXX:6:2206:8105:7936  - ACCTCTCCCCTCCTaTACTCCATCCGTCAGCAAATCCTACTGGTTTTTACCTTCCAAATTTTCCTTGAATCTGTCCTCTTCTATCTCCTCCATCACCACCCTAGTCTAGGCTGCCTTCACCTGGTGAGTGAGGGTCGAGGGACTACTGAC
ST-E00144:68:H2LCKCCXX:4:1214:31886:23741- TCTCCCCTCCTataCTCCATCCGTCAGCAAATCCTACTGGTTTTTACaTTCCAAATTTTCCTTGAATCTGTCCTCTTCTATCTCCTCCGTCACCACCCTAGTCTAGGCTGCCTTCACCTGGGGAGTGAGGGTCGAGGGACTAcTGACCTA
ST-E00144:68:H2LCKCCXX:6:2122:5567:18731 - CCCTCCTaTaCTCCATCCGTCAGCAAATCCTACTGGTTTtTACCTTCCAAATTTTCCTTGAATCTGTCCTCTTCTATCTCCTCCGTCACCACCCTAGTCTAGGCTGCCTTCACCTGGGGAGTGAGGGTCGAGGGACTACTGACCTAGTCT
ST-E00144:68:H2LCKCCXX:6:2216:18854:32672- CCCTCCTataCTCCATCcGTCAGCAAATCCTACTGGTTTTTACCTTCCAAATtTTCCTTGAATCTGTCCTCTTCTATCTCCTCCGTCACCACCCTAGTCTAGGCTGCCTTCACCTGGGGAGTGAGGGTCGAGGGACTACTGACCTAGTCT
ST-E00144:68:H2LCKCCXX:3:2120:27826:20823- CCTataCTCCATCCGTCAGCAAATCCTACTGGTTTTTACCTTCCAATTTTTCCTTGAATCTGTCCTCTTCTATCTCCTCCGTCACCACtCTAGTCTAGGCTGCCTTCACCTGGGGAGTGAGGGTCGAGGGACTACTGACCTAGTCTTCTT
ST-E00144:68:H2LCKCCXX:8:2109:26598:14758- CCTATACtCCATCCGtCAGCAAATCCtACTGGTTTTTACCTTCCAAATTTTCCTTGAATCTGTCCTCTtCTATCTCCtCCATCaCCACCCTAGTCTAGgCTGCCTTCACCTGGGGAGTGAGGGTCGAGGGACTACTGACCTAGTCTTCtT
ST-E00144:68:H2LCKCCXX:4:2121:16996:60009- ATACTCCATCCGTCAGCAAATCCTACTGGTTTTTACCTTCCAAATTTTCCTTGAATCTGTCCTCTTCTATCTCCTCCGTCACCACCCTAGTCTAGGCTGCCTTCACCTGGGGAGTGAGGGTCGAGGGACTACTGACCTAGTCTTCTTGTG
ST-E00144:68:H2LCKCCXX:3:1124:13038:59517- TACTCCATCCGTCAGCAAATCCTACTGGTTTTTACCTTCCAAATTTTCCTTGAATCTGTCCTCTTCTATCTCCTCCGTCACCACCCTAGTCTAGGCTGCCTTCACCTGGGGAGTGAGGGTCGAGGGACTACTGACCTAGTCTTCTTGTGG
ST-E00144:68:H2LCKCCXX:1:1118:8206:65002 - cCtCCATCCGTCAGCAAATCCTACTGGTTTTTACCTTCCAAATTTTCCTTGAATCTGTCCTCTTCTAtCTCCTCCGTCACCACCCTAGTCTAGGCTGCCTTCACCTGGGGAGTGAGGGTCGAGGGACTACTGACCTAGTCTTCTTGTGGT
ST-E00144:68:H2LCKCCXX:7:1208:6308:31793 - CATCCGTCAGCAaATCCTaCTGGTTTTTACCTTCCAAATTTtCCTTGaATCTGTCCTCTTCtATCTCCTCCGTCACCACCCTAGTCTAGGCTGCCTTCACCTGGGGAGTGAGGGTCGAGGGACTACTGACCTAGTCTTCTTGTGGTTTAC
ST-E00144:68:H2LCKCCXX:7:1208:6328:31863 - CaTCCGTCAGCAAATCCTACTGGTTTTTACCTTCCAAATTTTCCTTGAATCTGTCCTCTTCTAtCTCCTCCGTCACCACCCTAGTCTAGGCTGCCTTCACCTGGGGAGTGAGGGTCGAGGGACTACTGACCTAGTCTTCTTGTGGTTTAC
ST-E00144:68:H2LCKCCXX:8:2103:24223:73054- TCAGCAAATCCTACTGGTTTTTACCTTCCAAATTTTCCTTGAATCTGTCCTCTTCTATCTCCTCCGTCACCACCCTAGTCTAGGCTGCCTTCACCTGGGGAGTGAGGGTCGAGGGACTACTGACCTAGTCTTCTTGTGGTTTACCCATAT
ST-E00144:68:H2LCKCCXX:4:2206:14560:8921 - CAAaTCCTACTcGTTTTTACCTTCCAaATTTTCCTTGAATCTGTCCTCTTCTATCTCCTCCGTCACCACCCTAGTCTAGGCTGCCTTCACCTGGGGAGTGAGGGTCGAGGGACTACTGACCTAGTCTTCTTGTGGTTTACCCATATCCCC
ST-E00144:68:H2LCKCCXX:7:2119:32475:62734- AATCCTACTggTTTTTACCTTCCaaATTTTCCTTGAATCTGTCCTCTTCTATCTCCTCCGTCACCaCCCTAGTCTAGGCTGCCTTCACCTGGGGAGTGAGGGTCGAGGGACTACTGACCTAGTCTTCTTGTGGTTTACCCATATCCCCTT
ST-E00144:68:H2LCKCCXX:1:1109:16011:41779- ATCCTACTGGTTTTTACCTTCCAAATTTTCCTTGAATCTGTCCTCTTCTATCTCCTCCGTCACCACCCTAGTCTAGGCTGCCTTCACCTGGGGAGTGAGGGTCGAGGGACTACTGACCTAGTCTTCTTGTGGTTTACCCATATCCCCTTT
ST-E00144:68:H2LCKCCXX:7:2106:8460:51835 - TCCTACTGGTTTTTACCTTCCAaATTTTCCTTGAATCTGTCCTCTTCTATCTCCTCCGTCACCACCCTAGTaTAGGCTGCCTTCACCTGGGGAGTGAGGGTCGAGGGACTACTGACCTAGTCTTCTTGTGGTTTACCCATATCCCCTTTA
ST-E00144:68:H2LCKCCXX:8:2104:30018:31969- CCTACTGgTTTTTACCTTCCAACTTtTCCTTGAATCTGTCCTCTTCTATCTCCTCCGTCACCACCCTAGTCTAGGCTGCCTTCACCTGGGGAGTGAGGGTCGAGGGACTACTGACCTAGTCTTCTTGTGGTTTACCCATATCCCCTTTAT
ST-E00144:68:H2LCKCCXX:8:1110:2228:54577 - CTACtGGTTTTTACCTTCCAaATTTTCCTTGAATCTGTCCTCTTCTATCtCCTCCGTCACCACCCTAGTCTAGGCTGCCTTCACCTGGGGAGTGAGGGTCGAGGGACTACTGACCTAGTCTTCTTGTGGTTTACCCATATCCCCTTTATC
ST-E00144:68:H2LCKCCXX:1:1217:32576:70013- ttcctcatttccaCcctaacCCAactctaAtggTTtTtaCCcTCcaaATTTtcCaTgaAtctgcCCtCatctaTcgcCTcCgtCACCacccTagTctaGtCTgccTTcacCTgGGGaGTcAgGgtcgAGGGaCTACTGACcTagTCTTCT
ST-E00144:68:H2LCKCCXX:8:1223:14306:57531- TGGTTTtTACCTTCCAaatTTTCCTTGaATCTGTCCTCTTCTATCTCCtCCGTCACCACCCTAGTCTAGGCTGCCTTCACCTGGGGAGTGAGGGTCGAGGGACTACTGACCTAGTCTTCTTGTGGTTTACCCATATCCCCTTTATCGcCT
ST-E00144:68:H2LCKCCXX:8:1223:14367:58339- TGGTTTTTACCTTCCAAATTTTcCTTGAATCTGTCCTCTTCTATCTCCTCCGTCACCACCCTAGTCTAGGCTGCCTTCACCTGGGGAGTGAGGGTCGAGGGACTACTGACCTAGTCTTCTTGTGGTTTACCCATATCCCCTTTATCGCCT
ST-E00144:68:H2LCKCCXX:4:1206:27562:13685- TTTTACCtTcCaAaTTTTccTTgAATCTGTCCTcTTCTATCTCCTCCGTCACCACCCTAGTCTAGGCTGCCTTCACCTGGGGAGTGAGGGTCGAGGGACTACTGACCTAGTCTTCTTGTGGTTTACCCATATCCCCTTTATCGCCTcTCT
ST-E00144:68:H2LCKCCXX:4:2116:2218:46051 - TTtTaCCTTCCAatttTTCCTtGAATCTGTCCTCtTCtATCTCCTCCGTCACCaCCCTCGTCTAGGCTGCCTTCaCCTGGGGAGTGAGGGTCGAGGGACTACTGACCTAGTCTTCTTGTGGTTTACCCATATCCCCTTTATCGCCTCTCT
ST-E00144:68:H2LCKCCXX:3:2124:18295:38913- TACCTTCCAAATTTTCCTTGAATCTGTCCTCTTCTATCTCCTCCGTCACCACCCTAGTCTAGGCTGCCTTCACCTGGGGAGTGAGGGTCGAGGGACTACTGACCTAGTCTTCTTGTGGTTTACCCATATCCCCTTTATCGCCTCTCTAAT
ST-E00144:68:H2LCKCCXX:3:2124:18569:39071- TACCTTCCAAATTTTCCTTGAATCTGTCCTCTTCTATCTCCTCCGTCACCACCCTAGTCTAGGCTGCCTTCACCTGGGGAGTGAGGGTCGAGGGACTACTGACCTAGTCTTCTTGTGGTTTACCCATATCCCCTTTATCGCCTCTCTAAT
ST-E00144:68:H2LCKCCXX:3:2210:22152:51641- TACCTTCCAAATTTTCCTTGAATCTGTCCTCTTCTATCTCCTCCGTCACCACCCTAGTCTAGGCTGCCTTCACCTGGGGAGTGAGGGTCGAGGGACTACTGACCTAGTCTTCTTGTGGTTTACCCATATCCCCTTTATCGCCTCTCTAAT
ST-E00144:68:H2LCKCCXX:3:2210:21939:57425- TACCTTCCAAATTTTCCTTGAATCTGTCCTCTTCTATCTCCTCCGTCACCACCCTAGTCTAGGCTGCCTTCACCTGGGGAGTGAGGGTCGAGGGACTACTGACCTAGTCTTCTTGTGGTTTACCCATATCCCCTTTATCGCCTCTCTAAT
ST-E00144:68:H2LCKCCXX:6:1116:32536:37841- TACCTtCCAaaTTTTCCTTGAATCTGTCCTCTTCtatCTCCTCCGTCaCCACCCTAGTCTAGGCTGCCTTCACCTGGGGAGTGAGGGTCGAGGGACTACTGACCTAGTCTTCTTGTGGTTTACCCATATCCCCTTTATCGCCTCTCTAAT
ST-E00144:68:H2LCKCCXX:4:2114:26557:60783- CTTccaaaTTTTCCTTGAATCTGTCCTCTTCTATCTCCTCCGTCACCACCCTAGTCTAGGCTGCCTTCACCTGGGGAGTGAGGGTCGAGGGACTACTGACCTAGTCTTCTTGTGGTTTACCCATATCCCCTTTATCGCCTCTCTAATCTC
ST-E00144:68:H2LCKCCXX:4:1214:6389:53909 - TTCCAACTTtTCCTTGAATCTGTCCTCTTCTAtCTCCTCCGTCACCACCCTAGTCTAGGCTGCCTTCACCTGGGGAGCCAACTATGAATACACTGTGCATTCACTGCTGGGTGAAAATAGAGCTGTGTGCATTAAATATTTCTCCTTTGA
ST-E00144:68:H2LCKCCXX:4:2110:26973:64985- TTCCAcattttcCttgAatctgtCCTCTTCTaTCTCCTCCGTcACCACCCTAGTCTaggCTGCCTTCAccTGGGGagCCAAcTATgatTacaCTGTGCATTCACTGcTGgGTgAAAATAGAGCTGTGTGCATTAAATATTTCTCCTTTGA
ST-E00144:68:H2LCKCCXX:4:2110:26862:65143- tTCCaACTTTTCCTTGAATCTGTCCTCTTCTATCTCCTCCGTCACCACCCTAGTCTAgGCTGCCTTCACCTGGGGAG
ST-E00144:68:H2LCKCCXX:4:2110:27055:65160- TTCCAAATTTTCCTTGAATCTGTCCTCTtCTATCTCCTCCGTCACCaCCCTAGTCTAGGCTGCCTTCACCTGGGGAGCCAACTATGAATACACTGTGCATTCACTGCTGGGTGAAAATAGAGCTGTGTGCATTAAATATTTCTCCTTTGA
ST-E00144:68:H2LCKCCXX:1:1112:18782:6389 - CCAAATTTTCCTTGAATCTGTCCTCTTCTATCTCCTCCATCACCACCCTAGTCTAGGCTGCCTTCACCTGGGGAGTGAGGGTCGAGGGACTACTGACCTAGTCTTCTTGTGGTTTACCCATATCCCCTTTATCGCCTCTCTAATCTCAAC
ST-E00144:68:H2LCKCCXX:1:1112:19249:8112 - CCAAATTTTCCTTGAATCTGTCCTCTTCTATCTCCTCCATCACCACCCTAGTCTAGGCTGCCTTCACCTGGGGAGTGAGGGTCGAGGGACTACTGACCTAGTCTTCTTGTGGTTTACCCATATCCCCTTTATCGCCTCTCTAATCTCAAC
ST-E00144:68:H2LCKCCXX:6:1103:22771:31652- CCAAaTTTTCCTTGAATCTGTCCTCTTCTATCTCCTCCGTCACCACCCTAGTCTAGGCTGCCTTCACCTGGGGAGTGAGGGTCGAGGGACTACTGACCTAGTCTTCTTGTGGTTTACCCATATCCCCTTTATCGCCTCTCTAATCTCAAC
ST-E00144:68:H2LCKCCXX:3:2220:15961:47633- AAATTTTCCTTGAATCTGTCCTCTTCTATCTCCTCCGTCACCACCCTAGTCTAGGCTGCCtTCACCTGGGGAGTGAGGGTCGAGGGACTACTGACCTAGTCTTCTTGTGGTTTACCCATATCCCCTTTATCGCCTCTCTAATCTCAACAC
```

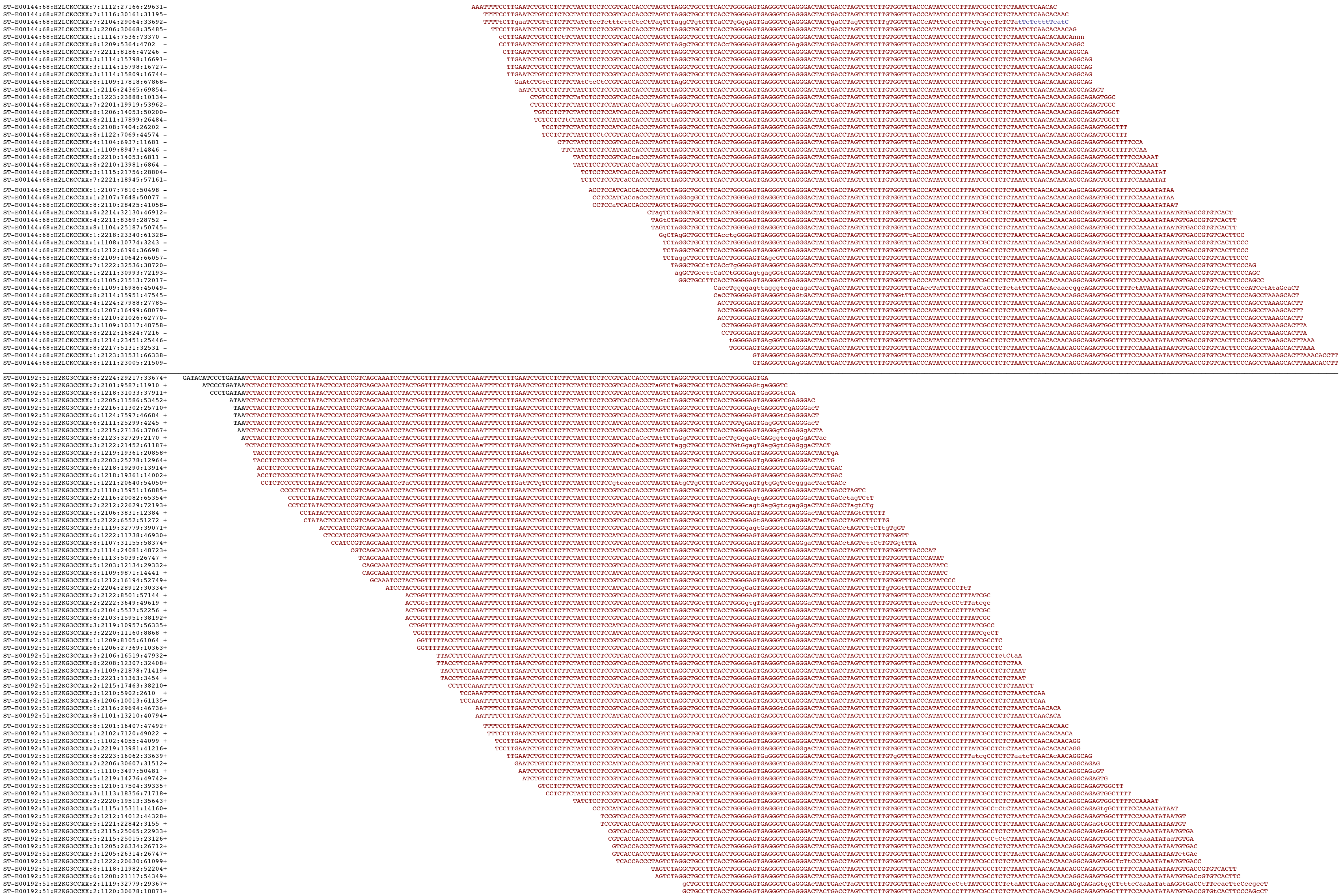

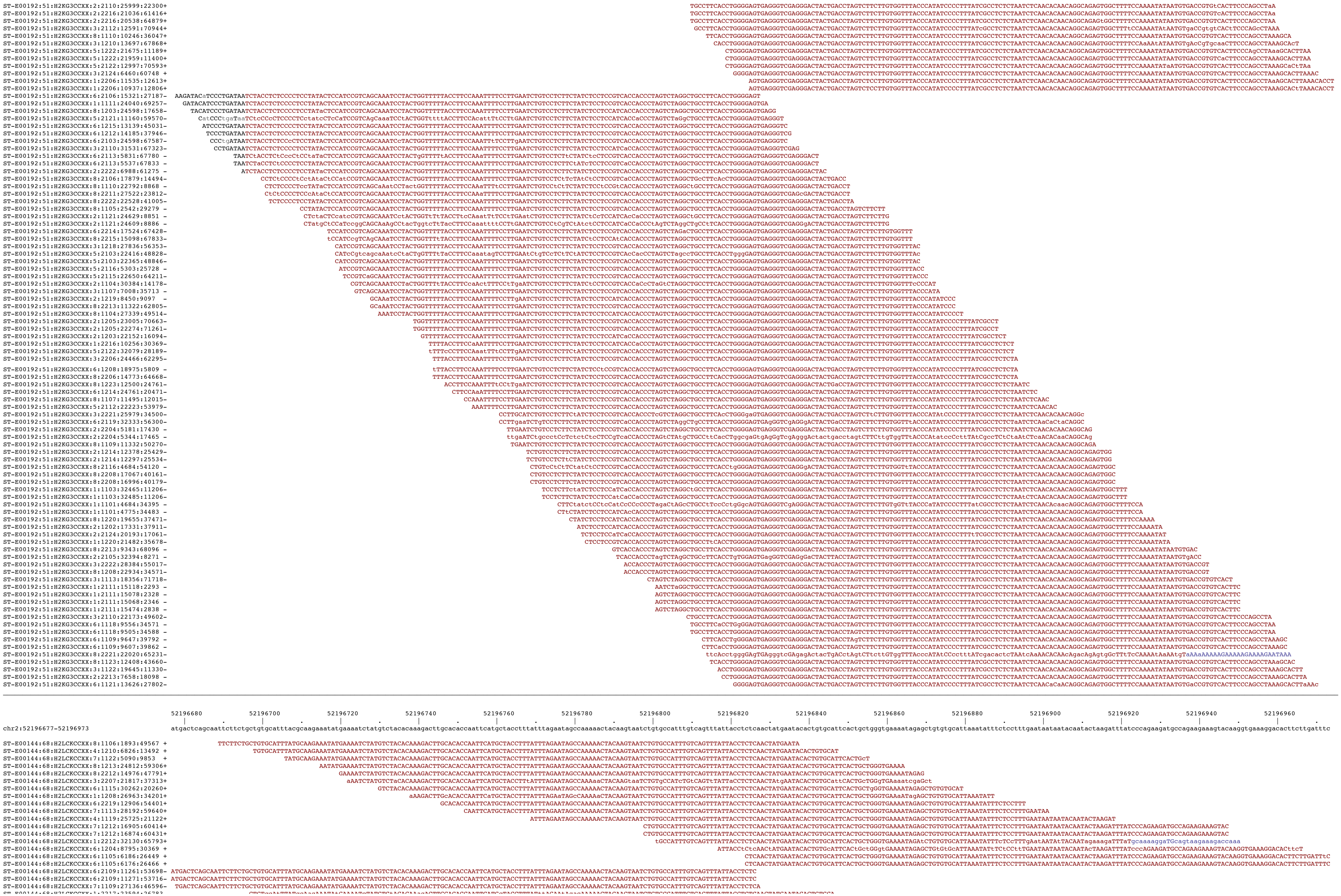

ST-E00192:51:H2KG3CCXX:2:2110:25999:22300+
ST-E00192:51:H2KG3CCXX:6:1121:13626:27802-
52196680
52196700
52196720
52196740
52196760
52196780
52196800
52196820
52196840
52196860
52196880
52196900
52196920
52196940
52196960
chr2:52196677-52196973
ST-E00144:68:H2LCKCCXX:8:1106:1893:49567 +
ST-E00144:68:H2LCKCCXX:7:1109:27736:46596-

```
[illegible]
ST-E00144:68:H2LCKCCXX:8:2222:22152:35608-  [illegible]
ST-E00144:68:H2LCKCCXX:7:2109:30079:72861-  [illegible]
ST-E00144:68:H2LCKCCXX:1:2223:19128:2733 -  [illegible]
ST-E00144:68:H2LCKCCXX:6:1121:16448:21438-  [illegible]
ST-E00144:68:H2LCKCCXX:7:2211:19391:71050-  [illegible]
ST-E00144:68:H2LCKCCXX:3:2121:29531:16287-  [illegible]
ST-E00144:68:H2LCKCCXX:4:1124:24527:36944-  [illegible]
ST-E00144:68:H2LCKCCXX:8:1209:23817:22985-  [illegible]
ST-E00144:68:H2LCKCCXX:8:2113:14834:26906-  [illegible]
ST-E00144:68:H2LCKCCXX:4:1212:18021:49549-  [illegible]
ST-E00144:68:H2LCKCCXX:7:1112:10155:23899-  [illegible]
ST-E00144:68:H2LCKCCXX:8:1106:1893:49567 -  [illegible]
ST-E00144:68:H2LCKCCXX:4:1108:31744:69802-  [illegible]
ST-E00144:68:H2LCKCCXX:1:1117:16418:19592-  [illegible]
ST-E00144:68:H2LCKCCXX:4:1210:6826:13492 -  [illegible]
ST-E00144:68:H2LCKCCXX:4:2110:26862:65143-  [illegible]
```

```
ST-E00192:51:H2KG3CCXX:8:1216:16864:28382+  [illegible]
ST-E00192:51:H2KG3CCXX:8:2109:20011:29086+  [illegible]
ST-E00192:51:H2KG3CCXX:3:1206:5933:56986 +  [illegible]
ST-E00192:51:H2KG3CCXX:8:2215:24314:56441+  [illegible]
ST-E00192:51:H2KG3CCXX:2:1201:16945:2715 +  [illegible]
ST-E00192:51:H2KG3CCXX:6:1219:21787:56318+  [illegible]
ST-E00192:51:H2KG3CCXX:6:1219:22020:56581+  [illegible]
ST-E00192:51:H2KG3CCXX:8:2107:20214:68501+  [illegible]
ST-E00192:51:H2KG3CCXX:8:1212:26090:3752 +  [illegible]
ST-E00192:51:H2KG3CCXX:1:2105:25431:22792+  [illegible]
ST-E00192:51:H2KG3CCXX:1:2105:25431:22827+  [illegible]
ST-E00192:51:H2KG3CCXX:8:1109:29795:33762+  [illegible]
ST-E00192:51:H2KG3CCXX:5:2116:3324:56124 +  [illegible]
ST-E00192:51:H2KG3CCXX:1:1211:10246:44662+  [illegible]
ST-E00192:51:H2KG3CCXX:1:2111:3659:15496 +  [illegible]
ST-E00192:51:H2KG3CCXX:3:2216:18458:7796 +  [illegible]
ST-E00192:51:H2KG3CCXX:2:2217:3507:16252 +  [illegible]
ST-E00192:51:H2KG3CCXX:2:2104:22142:46314+  [illegible]
ST-E00192:51:H2KG3CCXX:3:1102:16489:43554+  [illegible]
ST-E00192:51:H2KG3CCXX:3:1102:16478:43607+  [illegible]
ST-E00192:51:H2KG3CCXX:2:2217:14235:13773+  [illegible]
ST-E00192:51:H2KG3CCXX:6:1210:2613:40232 +  [illegible]
ST-E00192:51:H2KG3CCXX:1:2216:12835:51360+  [illegible]
ST-E00192:51:H2KG3CCXX:1:2216:12449:53083+  [illegible]
ST-E00192:51:H2KG3CCXX:1:2216:12571:53153+  [illegible]
ST-E00192:51:H2KG3CCXX:2:1211:7333:15883 +  [illegible]
ST-E00192:51:H2KG3CCXX:3:1108:25563:29631+  [illegible]
ST-E00192:51:H2KG3CCXX:6:1210:19584:45822-  [illegible]
ST-E00192:51:H2KG3CCXX:1:2202:12033:5809 -  [illegible]
ST-E00192:51:H2KG3CCXX:6:2203:13748:42253-  [illegible]
ST-E00192:51:H2KG3CCXX:3:1114:8988:34008 -  [illegible]
ST-E00192:51:H2KG3CCXX:8:1124:29288:59957-  [illegible]
ST-E00192:51:H2KG3CCXX:2:2207:26080:41779-  [illegible]
ST-E00192:51:H2KG3CCXX:2:1204:26517:32197-  [illegible]
ST-E00192:51:H2KG3CCXX:1:1222:8572:58603 -  [illegible]
ST-E00192:51:H2KG3CCXX:1:1222:8074:58937 -  [illegible]
ST-E00192:51:H2KG3CCXX:8:1204:21411:67938-  [illegible]
ST-E00192:51:H2KG3CCXX:8:2214:11525:52010-  [illegible]
ST-E00192:51:H2KG3CCXX:6:1202:14590:32953-  [illegible]
ST-E00192:51:H2KG3CCXX:1:2221:10896:59992-  [illegible]
ST-E00192:51:H2KG3CCXX:1:2221:11921:60607-  [illegible]
ST-E00192:51:H2KG3CCXX:2:2104:22142:46314-  [illegible]
```

---

chr7 100803313 + 0 chr1 107830339 + 5 CTX 54 36 0 39 1 0 0.931869939314093 0 1 chr7 100803275 150 chr1 107830449

ACACACACACACACATGCCCTGGCACACACACCCATCGTGTAATGGTTAGGAGCAGAGATTTACAGTTCACTAAACACTTTATTGAATTCCAACTTTGCCATTAGTTGAGCTCTATGACCTGTGGCACTCTCAGTTGTAAAATGGGTAC

```
                         100803180  100803200  100803220  100803240  100803260  100803280  100803300  100803320  100803340  100803360  100803380  100803400  100803420  100803440  1008
chr7:100803168-100803462 GGGCTCATCTTTGCAGTGGGAGAGAGGGCAGAACTGAACTTGCCACCTCACTGGTCGAGCTTCACCTTCCcacacacg**cacacacacaca**ccctggcg**cgcacacacaca**cacacacacacaTGCCCTGGCACACACACCCATCGTGTCACCAGTGTTGCGCTCAGACCCTAATGACGCATCTCCCTGCAGCCTGGTTCACCTTCCCTCAGGGACCTGGCACTCACCTCCCTCTGCCTTTTCCCCACTCTGAAAAACAGGACCCAGTCAGCTGTACCCCCCAGGGCCCCAGA

ST-E00144:68:H2LCKCCXX:8:2102:21990:30721+  [illegible]
ST-E00144:68:H2LCKCCXX:1:1203:9861:51413 +  [illegible]
ST-E00144:68:H2LCKCCXX:1:1208:19940:30123+  [illegible]
ST-E00144:68:H2LCKCCXX:1:2103:13281:41269+  [illegible]
ST-E00144:68:H2LCKCCXX:3:2223:15595:52415+  [illegible]
ST-E00144:68:H2LCKCCXX:3:1206:21178:51852+  [illegible]
ST-E00144:68:H2LCKCCXX:3:2215:18042:1871 +  [illegible]
ST-E00144:68:H2LCKCCXX:7:2120:12408:24357+  [illegible]
ST-E00144:68:H2LCKCCXX:3:2111:4461:14213 +  [illegible]
ST-E00144:68:H2LCKCCXX:1:1118:18813:34606+  [illegible]
ST-E00144:68:H2LCKCCXX:8:1209:24741:64879+  [illegible]
ST-E00144:68:H2LCKCCXX:4:2207:10683:30791+  [illegible]
ST-E00144:68:H2LCKCCXX:6:2220:13048:17026+  [illegible]
ST-E00144:68:H2LCKCCXX:4:1219:18519:10152+  [illegible]
ST-E00144:68:H2LCKCCXX:7:2113:31176:8833 +  [illegible]
ST-E00144:68:H2LCKCCXX:6:1118:15392:35678+  [illegible]
ST-E00144:68:H2LCKCCXX:1:2104:22112:8851 +  [illegible]
ST-E00144:68:H2LCKCCXX:3:1215:31480:42833+  [illegible]
ST-E00144:68:H2LCKCCXX:4:1205:26436:5088 +  [illegible]
ST-E00144:68:H2LCKCCXX:6:2103:18072:33182+  [illegible]
ST-E00144:68:H2LCKCCXX:6:1113:8145:65248 +  [illegible]
ST-E00144:68:H2LCKCCXX:6:1113:8125:65389 +  [illegible]
ST-E00144:68:H2LCKCCXX:8:1222:13647:41304+  [illegible]
ST-E00144:68:H2LCKCCXX:4:1121:11891:3911 +  [illegible]
ST-E00144:68:H2LCKCCXX:4:1121:11911:3911 +  [illegible]
ST-E00144:68:H2LCKCCXX:1:1112:25065:18362+  [illegible]
ST-E00144:68:H2LCKCCXX:1:1112:24639:18502+  [illegible]
ST-E00144:68:H2LCKCCXX:7:2202:25025:37348+  [illegible]
ST-E00144:68:H2LCKCCXX:7:2202:25035:37366+  [illegible]
ST-E00144:68:H2LCKCCXX:8:1101:19544:25605+  [illegible]
ST-E00144:68:H2LCKCCXX:3:2222:15403:53382+  [illegible]
ST-E00144:68:H2LCKCCXX:7:2105:14641:13597+  [illegible]
ST-E00144:68:H2LCKCCXX:7:1114:22670:51448-  [illegible]
ST-E00144:68:H2LCKCCXX:8:1209:2827:57583 -  [illegible]
ST-E00144:68:H2LCKCCXX:7:1201:22000:31688-  [illegible]
ST-E00144:68:H2LCKCCXX:8:2108:9140:26783 -  [illegible]
ST-E00144:68:H2LCKCCXX:8:1210:7333:31248 -  [illegible]
ST-E00144:68:H2LCKCCXX:7:1103:21767:37753-  [illegible]
ST-E00144:68:H2LCKCCXX:8:1114:18255:4315 -  [illegible]
ST-E00144:68:H2LCKCCXX:7:2207:26263:49373-  [illegible]
ST-E00144:68:H2LCKCCXX:3:1110:7729:8692  -  [illegible]
ST-E00144:68:H2LCKCCXX:6:2203:31470:33780-  [illegible]
ST-E00144:68:H2LCKCCXX:3:1222:32789:70206-  [illegible]
ST-E00144:68:H2LCKCCXX:4:1106:15474:70347-  [illegible]
ST-E00144:68:H2LCKCCXX:1:1215:9475:4754  -  [illegible]
ST-E00144:68:H2LCKCCXX:8:2215:16935:41480-  [illegible]
[illegible]
```

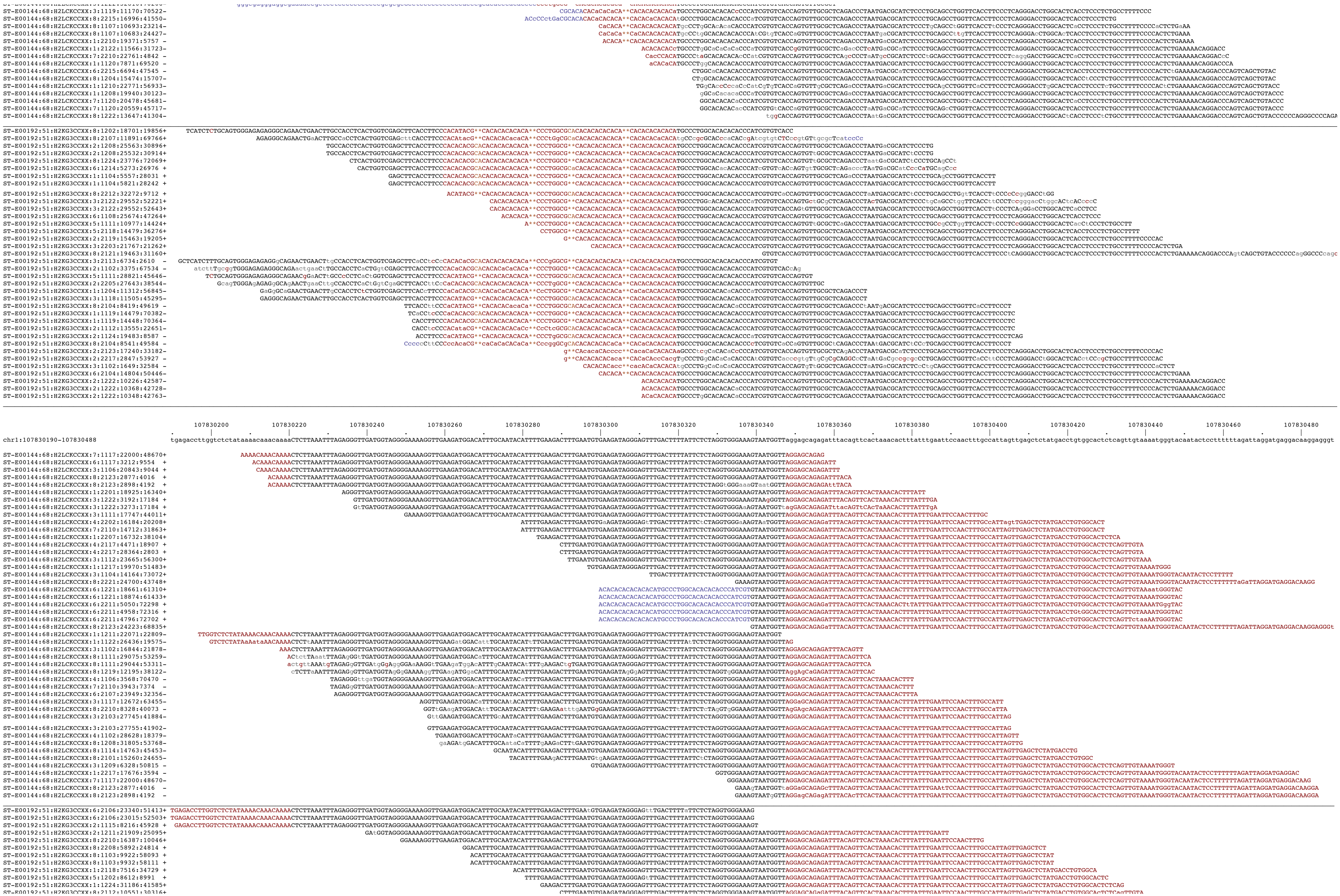

107830200
107830220
107830240
107830260
107830280
107830300
107830320
107830340
107830360
107830380
107830400
107830420
107830440
107830460
107830480
chr1:107830190-107830488

```
ST-E00192:51:H2KG3CCXX:8:2112:10591:30387+ CTTTGAATGTGAAGATAGGGAGTTTGACTTTTATTCTCTAGGTGGGAAAGTAATGGTTAGGAGCAGAGATTTACAGTTCACTAAACACTTTATTTGAATTCCAACTTTGCCATTAGTTGAGCTCTATGACCTGTGGCACTCTCAGTTGTA
ST-E00192:51:H2KG3CCXX:3:2109:18559:9308 + GTGAAGATAGGGAGTTTGACTTTTATTCTCTAGGTGGGAAAGTAATGGTTAGGAGCAGAGATTTACAGTTCACTAAACACTTTATTTGAATTCCAACTTTGCCATTAGTTGAGCTCTATGACCTGTGGCACTCTCAGTTGTAAAATGGGT
ST-E00192:51:H2KG3CCXX:2:2115:23746:55702+ AGGGAGTTTGACTTTTATTCTCTAGGTGGGAAAGTAATGGTTAGGAGCAGAGATTTACAGTTCACTAAACACTTTATTTGAATTCCAACTTTGCCATTAGTTGAGCTCTATGACCTGTGGCACTCTCAGTTGTAAAATGGGTACAATACT
ST-E00192:51:H2KG3CCXX:8:1117:18579:16305+ GGGAGTTTGACTTTTATTCTCTAGGTGGGAAAGTAATGGTTAGGAGCAGAGATTTACAGTTCACTAAACACTTTATTTGAATTCCAACTTTGCCATTAGTTGAGCTCTATGACCTGTGGCACTCTCAGTTGTAAAATGGGTACAATACTC
ST-E00192:51:H2KG3CCXX:1:2205:5293:72333 + TTGACTTTTATTCTCTAGGTGGGAAAGTAATGGTTAGGAGCAGAGATTTACAGTTCACTAAACACTTTATTTGAATTCCAACTTTGCCATTAGTTGAGCTCTATGACCTGTGGCACTCTCAGTTGTAAAATGGGTACAATACTCCTTTTT
ST-E00192:51:H2KG3CCXX:8:2109:6044:3418 + CTTTTATTCTCTAGGTGGGAAAGTAATGGTTAGGAGCAGAGATTTACAGTTCACTAAACACTTTATTTGAATTCCAACTTTGCCATTAGTTGAGCTCTATGACCTGTGGCACTCTCAGttGTAAAATGGGTACAATACTCCTTTTTAGA
ST-E00192:51:H2KG3CCXX:8:2109:6136:3471 + CTTTTATTCTCTAGGTGGGAAAGTAATGGTTAGGAGCAGAGATCTACAGtTCAcTAAACACTTTATTTGAATTCCAACTTTGCCATTAGTTGAGCTcTATGACctGTGGCACtCTCAGtTGTAAAATGGGtACAATACTAcTtaTTtAgA
ST-E00192:51:H2KG3CCXX:5:1120:27156:40900+ CTCTAGGTGGGAAAGTAATGGTTAGGAGCAGAGATTTACAGTTCACTAAACACTTTATTTGAATTCCAACTTTGCCATTAGTTGAGCTCTATGACCTGTGGCACTCTCAGTTGTAAAATGGGTACAATACTCCTTTTTAGATTAGGATG
ST-E00192:51:H2KG3CCXX:2:1208:22629:29015- GAGACCTTGGTCTCTATAAAACAAACAAAACTCTTAAATTTAGAGGGTTGATGGTAGGGGAAAAGGTTGAAGATGGACATTTGCAATACATTTTGAAGACTTTGAATGTGAAGATAGGGAGTTTGACTTTTATTCTCTAGGTGGGAAAGT
ST-E00192:51:H2KG3CCXX:3:2203:8582:26273 - CCTTGgTCTCTATAAAACAAACAAAACTCTTAAATTTAGAGGGTTGATGGTAgGGGAAAAGGTTGAAGATGGACATTTGCAATACATTTTGAAGACTTTGAATGTGAAGATAGGGAGTTTGACTTTTATTCTCTAGGTGGGAAAGTAATG
ST-E00192:51:H2KG3CCXX:6:2117:16976:15074- TCTATAAAACAAACAAAACTCTTAAAtTTAGAGGGTTGATGGTAGGGGAAAAGGTTGAAGATGGACATTTGCAATACATTTTGAAGACTTTGAATGTGAAGATAGGGAGTTTGACTTTTATTCTCTAGGTGGGAAAGTAATGGTTAGGAG
ST-E00192:51:H2KG3CCXX:3:1206:5141:43660 - TATAAAACAAACAAAACTCTTAAATTTAGAGGGTTGATgGTAGGGGAAAAGGTTGAAGATGGACATTTGCAATACATTTTGAAGACTTTGAATGTGAAGATAGGGAGTTTGACTTTTATTCTCTAGGTGGGAAAGTAATGGTTAGGAGCA
ST-E00192:51:H2KG3CCXX:3:2103:18062:19944- TATaAAaCAAAcaaaACTCtTaATTTAGagGgtTGATGGTAGGGGAaAAGGTTGAAGAtGGaCATTTGCAATACATTTTGAAGACTTTGAATGTGAAGATAGGGAGTTTGACTTTTATTCTCTAGGTGGGAAAGTAATGGTTAGGAGCA
ST-E00192:51:H2KG3CCXX:3:2103:18123:20014- TATAAAACAAAcAaaACTCTTAaATTTAGAggGTTGATGGTAGGGGAAAAGGTTGAAGAtGGACAtTTGCAATACATTTTGAAGAcTTTGAATGTGAAGATAGGGAGTTTGACTTTTATTCTCTAGGTGGGAAAGTAATGGTTAGGAGCA
ST-E00192:51:H2KG3CCXX:2:1222:22355:69116- AtAAAaCAAACAAAACTCTTAAATTTAGAGGGTTGATGGTAGGGGAAAAGGTTGAAGATGGACATTTGCAATACATTTTGAAGACttTGAATGTGAAGATAGGGAGTTTGACTTTTATTCTCTAGGTGGGAAAGTAATGGTTAGGAGCAG
ST-E00192:51:H2KG3CCXX:1:1223:9191:66215 - ACAAACAAAACTCTTAAATTTAGAGGGTTGATGGTAGGGGAAAAGGTTGAAGATGGACATTTGCAATACATTTTGAAGACTTTGAATGTGAAGATAGGGAGTTTGACTTTTATTCTCTAGGTGGGAAAGTAATGGTTAGGAGCAGAGATT
ST-E00192:51:H2KG3CCXX:6:1204:28161:16481- AAATTTAGAGGGTTGATGGTAGGGGAAAAGGTTGAAGATGGACATTTGCAATACATTTTGAAGACTTTGAATGTGAAGATAGGGAGTTTGACTTTTATTCTCTAGGTGGGAAAGTAATGGTTAGGAGCAGAGATTTACAGTTCACTAAAC
ST-E00192:51:H2KG3CCXX:5:2123:30272:3155 - ATTTAGAGGGTTGATGGTAGGGGAAAAGGTTGAAGATGGACATTTGCAATACATTTTGAAGACTTTGAATGTGAAGATAGGGAGTTTGACTTTTATTCTCTAGGTGGGAAAGTAATGGTTAGGAGCAGAGATTTACAGTTCACTAAACAC
ST-E00192:51:H2KG3CCXX:5:2123:30323:3243 - ATTTAGAGGGTTGATGGTAGGGGAAAAGGTTGAAGATGGACATTTGCAATACATTTTGAAGACTTTGAATGTGAAGATAGGGAGTTTGACTTTTATTCTCTAGGTGGGAAAGTAATGGTTAGGAGCAGAGATTTACAGTTCACTAAACAC
ST-E00192:51:H2KG3CCXX:8:1117:12215:15689- GATGGTAGGGGAAAAGGTTGAAGATGGACATTTGCAATACATTTTGAAGACTTTGAATGTGAAGATAGGGAGTTTGACTTTTATTCTCTAGGTGGGAAAGTAATGGTTAGGAGCAGAGATTTACAGTTCACTAAACACTTTATTTGAATT
ST-E00192:51:H2KG3CCXX:1:2107:29460:7585 - AAAAGGTTGAAGATGGACATTTgCAATACATTTTGAAGACTTTGAATGTGAAGATAGGGAGTTTGACTTTTATTCTCTAGGTGGGAAAGTAATGGTTAGGAGCAGAGATTTACAGTTCACTAAACACTTTATTTGAATTCCAACTTTGCC
ST-E00192:51:H2KG3CCXX:3:1111:18996:19663- ACATTTGCAATACATTTTGAAGACTTTGAATGTGAAGATAGGGAGTTTGACTTTTATTCTCTAGGTGGGAAAGTAATGGTTAGGAGCAGAGATTTACAGTTCACTAAACACTTTATTTGAATTCCAACTTTGCCATTAGTTGAGCTCTAT
ST-E00192:51:H2KG3CCXX:3:2114:19219:34641- ATTTGCAATACATTTTGAAGACTtTGAATGTGAAGATAGGGAGTTTGACTTTTATTCTCTAGGTGGGAAAGTAATGGTTAGGAGCAGAGATTTACAGTTCACTAAACACTTTATTTGAATTCCAACTTTGCCATTAGTTGAGCTCTATGA
ST-E00192:51:H2KG3CCXX:6:1211:13850:9484 - CTTTGAATGTGAAGATAGGGAGTTTGACTTTTATTCTCTAGGTGGGAAAGTAATGGTTAGGAGCAGAGATTTACAGTTCACTAAACACTTTATTTGAATTCCAACTTTGCCATTAGTTGAGCTCTATGACCTGTGGCACTCTCAGTTGTA
ST-E00192:51:H2KG3CCXX:3:1111:22995:12349- GaaGATAGGGAGTTTGaCTTTTATtCTCTAGGTGGGAAAGTAATGGTTAGGAGCAGAgATTTACAGTTCACTAAACaCTTTATTTGAATTCCAACTTTGCCATTAGTTGAGCTCTATGAcCTGTGGCACTCTCAGTTGTAAAATGGGTAC
ST-E00192:51:H2KG3CCXX:8:2210:16387:10046- TTTTATTCTCTAGGTGGGAAAGTAATGGTTAGGAGCAGAGATTTACAGTTCACTAAACACTTTATTTGAATTCCAACTTTGCCATTAGTTGAGCTCTATGACCTGTGGCACTCTCAGTTGTAAAATGGGTACAATACTCCTTTTTAGAT
ST-E00192:51:H2KG3CCXX:2:2106:23340:51413- ttTAcTCTCTAgGTgGGAAAGTAATGGTTAGGAGCAGAGatttaCAGttCaCTAAaCaCTTTAtTGAATTCCAACTTtGCCATTAGTTGAGCTCTATGACCTGTGGCACTCTCAGTTGgAAaATGGGTACAATACTCCTTTTTAGAtt
ST-E00192:51:H2KG3CCXX:6:2106:23015:22503- TTTATgCTCTAGGTGGGAAAGTAATGGTTAGGAGCAGAGATTTACAGTTCACTAAACACTTTATTTGAATTCCAACTTTGCCATTAGTTGAGCTCTATGACCTGTGGCACTCTCAGTTGTAAAATGGGTACAATACTCCTTTTTAGATT
ST-E00192:51:H2KG3CCXX:5:2107:8572:49496 - ATTcgcTaggtagtaaagTAATTGTTatgagCAGAGATTTACAGTTCACTAAACACTTTATTTGAATTCCAACtTTGCCATTAGTTGAGCTCTATgACCTGTGGCACTCTCAGTTGTAAAATGGGTACAATACTCCTTTTTAGATTAGG
ST-E00192:51:H2KG3CCXX:6:1113:3618:15320 - GTAATGGTTAGGAGCAGAGATTTACAGTTCACTAAACACTTTATTTGAATTCCAACTTTGCCATTAGTTGAGCTCTATGACCTGTGGCACTCTCAGTTGTAAAATGGGTACAATACTCCTTTTTAGATTAGGATGAGGACAAGGAGGGT
```

chr4 138773854 - 3 chr1 160345744 + 3 CTX 29 70 17 37 0.972761904761905 0 0.986 0 1 chr1 160345856 150 chr4 138773906

GCTTTCCATATGAAAAAGCTGTCGCTCAGGGAGGGCATGCGGTTTGCTCACTGTGGCACAAAGGGGAAAGGTGGAGACAGAGCAGGGACACGGGACCCCTGCCTGCCTGGTGTTCTTTGTCTACTGTAAGCAAACAAGAGCTAGCATCAT

```
                 138773720    138773740    138773760    138773780    138773800    138773820    138773840    138773860    138773880    138773900    138773920    138773940    138773960    138773980    138774000
chr4:138773705-138774002 catcatgagctcacagtgaggcctgtggggaagaacctgtgaatctctccaaattccctttgtatgtgtggcttctaatacttctatatgctcacattctcctatgctcaacatttagcaatttattaaaaattttaactgagttcttacctgcctgcctggtgttctttgtctactgtaagcaaacaagagctagcatcatgtctctccttggaggtgtctgcctttccttagatattggctttgttggttgccctacaaccacaggtcgctgatcaattcaagaaaagttgtgatt

ST-E00144:68:H2LCKCCXX:1:1107:28080:17078+ ATCATGAGCTCACAGTGAGGCCTGTGGGGAAGAACCTGTGAATCTCTCCAAATTCCCTTTGTATGTGTGGCTTCTAATACTTCTATATGCTCACATTCTCCTATGCTCAACATTTAGCAaTTTATTAAAAATTTTAaCTGAGTTCTTACC
ST-E00144:68:H2LCKCCXX:1:2220:30465:35379+ GAGCTCACAGTGAGGCCTGTGGGGAAGAACCTGTGAATCTCTCCAAATTCCCTTTGTATGTGTGGCTTCTAATACTTCTATATGCTCACATTCTCCTATGCTCAACATTTAGCAATTTATTAAAAATTTTAaCTGAGTTCTTACCTGCcT
ST-E00144:68:H2LCKCCXX:1:1118:29714:59148+ TCCAAATTCCCTTTGTATGTGTGGCTTcTAATActTCtATaTGCTCACATtCTCCcaTGCTCaaCATTTAGCAATttATTAAAaATTtTAACTGAgtTCtTACctGCcTGcCtggTgttcTTTgtCTACTgtAAGCAAACAAgaGctaGC
ST-E00144:68:H2LCKCCXX:3:1109:10896:46947+ TTCCCTTTGTATGTGTGGCTTCTAATACTTCTATATGCTCACATTCTCCTATGCTCAACATTTAGCAATTTATTAAAAATTTTAACTGAGTTCTTACCTGCCTGCCTGGTGTTCTTTGTCTACTGTAAGCAAACAAGAGCTAGCATCATG
ST-E00144:68:H2LCKCCXX:4:1220:2989:24110 + CCTATGCTCAACAtTTAGcAATTTATtAAAAAtttTAACTGAGtTCTTACCTGCctGCCTGGTGTTCTTTGTCTACTGtaaGCAAACAAGAGCtAGCATCATGTCTCTCcTTGGAgGTGTCTGCCTTTCtttatttaTgGtcttTctttg
ST-E00144:68:H2LCKCCXX:4:1118:14611:48424+ TCAACATTTAGCAATTTATTAAAAATTTTAACTGAGTTCTTACCTGCCTGcCTGgTGTTCTTtGTCtACTGTAAGCAAACAAGAGCTAGCATCATGTCTCTCcTTGGAGGTGTCTGCCTTTCCTTAGATATTGGCTTTGTTGGTtGCCCT

ST-E00144:68:H2LCKCCXX:6:1121:6318:26044 + TTTAGCAATTTATTAAAAATTTTAACTGAGTTCTTACCTGCCTGCCTGGTGTTCTTTGTCTACTGTAAGCAAACAAGAGCTAGCATCATGTCTCTCCTTGGAGGTGTCTGCCTTTCCTTAGATATTGGCTTTGTTGGTTGCCCTACAACC
ST-E00144:68:H2LCKCCXX:6:1106:13291:34993+ AGCAATTTATTAAAAATTTTAACTGAGTTCTTACCTGCCTGCCTGGTGTTCTTTGTCTACTGTAAGCAAACAAGAGCTAGCATCATGTCTCTCCTTGGAGGTGTCTGCCTTTCCTTAGATATTGGCTTTGTTGGTTGCCCTACAACCACA
ST-E00144:68:H2LCKCCXX:8:2120:32241:60326+ ATTAAAAATTTTAACTGAGTTCTTACCTGCCTGCCTGGTGTTCTTTGTCTACTGTAAGCAAACAAGAGCTAGCATCATGTCTCTCCTTGGAGGTGTCTGCCTTTCCTTAGAtATTGGCTgTTGTTGGTTgccctACAACCACAGGTcgCTG
ST-E00144:68:H2LCKCCXX:4:1116:26710:41884+ TACCTGCCTGCCTGGTGTTCTTTGTCTACTGTAAGCAAACAAGAGCTAGCATCATGTCTCTCCTTGGAGGTGTCTGCCTTTCCTTAGATATTGGCTTTGTTGGTTGCCCTACAACCACAGGTCGCTGATCAATTCAAGAAAAGTTGTGAT
ST-E00144:68:H2LCKCCXX:4:1207:22162:64457+ ACCTGCCTGCCTGGTGTTCTTTGTCTACTGTAAGCAAACAAGAGCTAGCATCATGTCTCTCCTTGGAGGTGTCTGCCTTTCCTTAGATATTGGCTTTGTTGGTTGCCCTACAACCACAGGTCGCTGATCAATTCAAGAAAAGTTGTGATT
ST-E00144:68:H2LCKCCXX:8:1208:6339:42886 - CATCATGAGCTCACAGTGAGGCCTGTGGGGAAGAACCTGTGAATCTCTCCAAATTCCCTTTGTATGTGTGGCTTCTAATACTTCTATATGCTCACATTCTCCTATGCTCAACATTTAGCAATTTATTAAAAATTTTAACTGAGTTCTTAC
ST-E00144:68:H2LCKCCXX:7:1221:20609:15320- AGCTCACAGTGAGGCCTGTGGGGAAGAACCTGTGAATCTCTCCAAATTCCCTTTGTATGTGTGGCTTCTAATACTTCTATATGCTCACATTCTCCTATGCTCAACATTTAGCAATTTATTAAAAATTTTAACTGAGTTCTTACCTGCCTG
ST-E00144:68:H2LCKCCXX:3:1123:10409:66250- CTCACAGTGAGGCCTGTGGGGAAGAACCTGTGAATCTCTCCAAATTCCCTTTGTATGTGTGGCTTCTAATACTTCTATATGCTCACATTCTCCTATGCTCAACATTTAGCAATTTATTAAAAATTTTAACTGAGTTCTTACCTGCCTGCC
ST-E00144:68:H2LCKCCXX:4:2113:3608:61750 - tCACAGTgAgGcctgTgggGAAGAACCTGTgAAtCtCTCCAAATTCCCTTTGTATGTGTGGCTTCTAATACTTCTATATGCTCACATTCTCCTATGCTCAACATTTAGCAATTTATTAAAAATTTTAACTGAGTTCTTACCTGCCTGCCT
ST-E00144:68:H2LCKCCXX:7:1205:13454:60027- GTGGGGAAGAacctGgGAAgCTCTCCAAATTCCCTGTGTAgGTGTGGCTTCTAATACTTCTATATgCTCgccTTCTCCTATGCTCcgCaTtTcGCAATTTATTaagAATTTTaaCTGAGTTCTTACCTGCCTGCCTGGTGTTCTTTtGTCT
ST-E00144:68:H2LCKCCXX:4:2113:3385:61469 - gcacattGagttCtttagggaatAacCTGtGAatctCtCAAAATtCCCTTtgtATGTGTGgCTTCTAATACTtCtATaTGCtCACATTccTaTtCTCAACATTTAGCAATTTATTAAAAATTTTAACTGAgTTCTTACCTGCCTgCCT
ST-E00144:68:H2LCKCCXX:8:1102:27481:37700- CCTGTGAATCTCTCCAAATTCCCTTTGTATGTGTGGCTTCTAATACTTCTATATGCTCACATTCTCCTATGCTCAACATTTAGCAATTTATTAAAAATTTTAACTGAGTTCTTACCTGCCTGCCTGGTGTTCTTTGTCTACTGTAAGCA
ST-E00144:68:H2LCKCCXX:3:2207:6217:68378 - GTGAATCTCTCCAAATTCCCTTTGTATGTGTGGCTTCTAATACTTCTATATGCTCACATTCTCCTATGCTCAACATTTAGCAATTTATTAAAAATTTTAACTGAGTTCTTACCTGCCTGCCTGGTGTTCTTTGTCTACTGTAAGCAAACA
ST-E00144:68:H2LCKCCXX:1:1112:22579:2030 - ATCTCTCCAAATTCCCTTTGTATGTGTGGCTTCTAATACTTCTATATGCTCACATTCTCCTATGCTCAACATTTAGCAATTTATTAAAAATTTTAACTGAGTTCTTACCTGCCTGCCTGGTGTTCTTTGTCTACTGTAAGCAAACAAGAG
ST-E00144:68:H2LCKCCXX:3:1210:4390:14758 - CTCTCCAAATTCCCTTTGTATGTGTGGCTTCTAATACTTCTATATGCTCACATTCTCCTATGCTCAACATTTAGCAATTTATTAAAAATTTTAACTGAGTTCTTACCTGCCTGCCTGGTGTTCTTTGTCTACTGTAAGCAAACAAGAGCT
ST-E00144:68:H2LCKCCXX:4:2213:17290:14740- CAAATTCCCTTTGTATGTGTGGCTTCTAATACTTCTATATGCTCACATTCTCCTATGCTCAACATTTAGCAATTTATTAAAAATTTTAACTGAGTTCTTACCTGCCTGCCTGGTGTTCTTTGTCTACTGTAAGCAAACAAGAGCTAGCAT
ST-E00144:68:H2LCKCCXX:6:2218:17158:33393- ATTCCCTTTGTATGTGTGGCTTCTAATACTTCTATATGCTCACATTCTCCTATGCTCAACATTTAGCAATTTATTAAAAATTTTAACTGAGTTCTTACCTGCCTGCCTGGTGTTCTTTGTCTACTGTAAGCAAACAAGAGCTAGCATCAT
ST-E00144:68:H2LCKCCXX:1:2206:27725:67059- CCCTTTGTATGTGTGGCTTCTAATACTTCTATATGCTCACATTCTCCTATGCTCAACATTTAGCAATTTATTAAAAATTTTAACTGAGTTCTTACCTGCCTGCCTGGTGTTCTTTGTCTACTGTAAGCAAACAAGAGCTAGCATCATGT
ST-E00144:68:H2LCKCCXX:6:1117:20092:27855- CTAATACTTCTATATGCTCACATTCTCCTATGCTCAACATTTAGCAATTTATTAAAAATTTTAACTGAGTTCTTACCTGCCTGCCTGGTGTTCTTTGTCTACTGTAAGCAAACAAGAGCTAGCATCATGTCTCTCCTTGGAGGTGTCTGC
ST-E00144:68:H2LCKCCXX:1:1204:17158:26466- TCCTATGCTCAACATtTAGCAATTTATTAAAAATTTTAACTGAGTTCTTACCTGCCTGCCTGGTGTTCTTTGTCTACTGTAAGCAAACAAGAGCTAGCATCATGTCTCTCCTTGGAGGTGTCTGCCTTTCCTTAGATATTGGCTTTGTTG
ST-E00144:68:H2LCKCCXX:1:2117:14377:30650- GCTCAACATTTAGCAATTTATTAAAAATTTTAACTGAGTTCTTACCGGCCTGCCTGGTGTTCTTTGTCTACTGTAAGCAAACAAGAGCTAGCATCATGTCTCTCCTTGGAGGTGTCTGCCTTTCCTTAGATATTGGCTTTGTTGGTTGCC
ST-E00144:68:H2LCKCCXX:1:2124:10886:7163 - ATTTATTAAAAATTTTAACTGAGTTCTTACCTGCCTGCCTGGTGTTCTTTGTCTACTGTAAGCAAACAAGAGCTAGCATCATGTCTCTCCTTGGAGGTGTCTGCCTTTCCTTAGATATTGGCTTTGTTGGTTGCCCTACAACCACAGGTC
ST-E00144:68:H2LCKCCXX:8:1210:19645:27363- aTTtATtaaaaatTTTAACTGcGTTCTTACCTGCCTgCcTGGTGTTCtgTGTCTACTGTAAGCAAACAAGAGCTAGCATCATGTCTCTCCTTGGAGGTGTCTGCCTTTCCTTAGATATTGGCTTTGTTGGTTGCCCTACAACCACAgGTC
ST-E00144:68:H2LCKCCXX:8:2103:14895:40970- ATTTATTAAAAATTTTAACTGAGTTCTTACCTGCCTGCCTGGTGTTCTTTGTCTACTGTAAGCAAACAAGAGCTAGCATCATGTCTCTCCTTGGAGGTGTCTGCCTTTCCTTAGATATTGGCTTTGTTGGTTGCCCTACAACCACAGGTC
ST-E00144:68:H2LCKCCXX:7:2107:8186:56739 - AGCAGGGACACGGGACCCCTGCCTGCCTGGTGTTCTTTGTCTACTGTAAGCAAACAAGAGCTAGCATCATGTCTCTCCTTGGAGGTGTCTGCCTTTCCTTAGATATTGGCTTTGTTGGTTGCCCTACAACCACAGGTCGCTGATCAATTC
ST-E00144:68:H2LCKCCXX:7:2107:8277:57636 - AGCAGGGACACGGGACCCCTGCCTGCCTGGTGTTCTTTGTCTACTGTAAGCAAACAAGAGCTAGCATCATGTCTCTCCTTGGAGGTGTCTGCCTTTCCTTAGATATTGGCTTTGTTGGTTGCCCTACAACCACAGGTCGCTGATCAATTC
ST-E00144:68:H2LCKCCXX:7:2107:8318:57847 - AGCAGGGACACGGGaCCCCTGCCTGCCTGGTGTTCTTTGTCTACTGTAAGCAAACAAGAGCTAGCATCATGTCTCTCCTTGGACGTGTCTGCCTTTCCTTAGATATTGGCTTTGTTGGTTGCCCTACAACCACAGGTCGCTGATCAATTC

ST-E00192:51:H2KG3CCXX:1:2201:30658:47176+ CATCATGAGCTCACAGTGAGGCCTGTGGGGAAGAACCTGTGAATCTCTCCAAATTCCCTTTGTATGTGTGGCTTCTAATACTTCTATATGCTCACATTCTCCTATGCTCAACATTTAGCAATTTATTAAAAATTTTAAtTGAgTTCTTAg
ST-E00192:51:H2KG3CCXX:3:2212:25390:61855+ CACAGTGAGGCCTGTGGGGAAGAACCTGTGAATCTCTCCAAATTCCCTTTGTATGTGTGGCTTCTAATACTTCTATATGCTCACATTCTCCTATGCTCAACATTTAGCAATTTATTAAAAATTTTAACTGAGTTCTTACCTGCCTGCCTG
ST-E00192:51:H2KG3CCXX:3:2212:25309:64211+ CACAGTGAGGCCTGTGGGGAAGAACCTGTGAATCTCTCCAAATTCCCTTTGTATGTGTGGCTTCTAATACTTCTATATGCTCACATTCTCCTATGCTCAACATTTAGCAATTTATTAAAAATTTTAACTGAGTTCTTACCTGCCTGcCTG
ST-E00192:51:H2KG3CCXX:3:2218:15007:70769+ TGAGGCCTGTGGGGAAGAACCTGTGAATCTCTCCAAATTCCCTTTGTATGTGTGGCTTCTAATACTTCTATATGCTCACATTCTCCTATGCTCAACATTTAGCAATTTATTAAAAATTTTAACTGAGTTCTTACCTGCCTGCCTGGTGTT
ST-E00192:51:H2KG3CCXX:3:2218:15169:70980+ TGAGGCCTGTGGGGAAGAACCTGTGAATCTCTCCAAATTCCCTTTGTATGTGTGGCTTCTAATACTTCTATATGCTCACATTCTCCTATGCTCAACATTTAGCAATTTATTAAAAATTTTAACTGAGttCTTACCTGCCTGCCTGGTGTt
ST-E00192:51:H2KG3CCXX:8:1206:14773:7356 + AGAACCTGTGAATCTCTCCAAATTCCCTTTGTATGTGTGGCTTCTAATACTTCTATATGCTCACATTCTCCTATGCTCAACATTTAGCAATTTATTAAAAATTTTAACTGAGTTCTTACCTGCCTGCCTGGTGTTCTTTGTCTACTGTAa
ST-E00192:51:H2KG3CCXX:2:2218:4826:48037 + TCCCTTTGTATGTGTGGCTTCTAATACTTCTATATGCTCACATTCTCCTATGCTCAACATTTAGCAATTTATTAAAAATTTTAACTGAGTTCTTACCTGCCTGCCTGGTGTTCTTTGTCTACTGTAAGCAAACAAGAGCTAGCATCATGT
ST-E00192:51:H2KG3CCXX:3:2212:7668:50745 + ATATGCTCACATTCTCCTATGCTCAACATTTAGCAATTTATTAAAAATTTTAACTGAGTTCTTACCTGCCTGCCTGGTGTTCTTTGTCTACTGTAAGCAAACAAGaGCTAGCATCATGTCTCTCCTtGGAGGTGTCTGCCTTtcctTAGA
ST-E00192:51:H2KG3CCXX:5:1203:29734:7462 + AATTTATTAAAAATTTTAACTGAGTTCTTACCTGCCTGCCTGGTGTTCTTTGTCTACTGTAAGCAAACAAGAGCTAGCATCATGTCTCTCCTTGGAGGTGTCTGCCTTTCCTTAGATATTGGCTTTGTTGGTTGCCCTACAACCACAGGT
ST-E00192:51:H2KG3CCXX:3:2201:8054:63227 + TTTTAACTGAGTTCTTACCTGCCTGCCTGGTGTTCTTTGTCTACTGTAAGCAAACAAGAGCTAGCATCATGTCTCTCCTTGGAGGTGTCTGCCTTTCCTTAGATATTGGCTTTGTTGGTTGCCCTACAACCACAGGTCGCTGATCAATTC
ST-E00192:51:H2KG3CCXX:3:2201:8379:63438 + TTTTAACTGAGTTCTTACCTGCCTGCCTGGTGTTCTTTGTCTACTGTAAGCAAACAAGAGCTAGCATCATGTCTCTCCTTGGAGGTGTCTGCCTTTCCTTAGATATTGGCTTTGTTGGTTGCCCTACAACCACAGGTCGCTGATCAATTC
ST-E00192:51:H2KG3CCXX:3:2201:8521:63649 + TTTTAACTGAGTTCTTACCTGCCTGCCTGGTGTTCTTTGTCTACTGTAAGCAAACAAGAGCTAGCATCATGTCTCTCCTTGGAGGTGTCTGCCTTTCCTTAGATATTGGCTTTGTTGGTTGCCCTACAACCACAGGTCGCTGATCAATTC
ST-E00192:51:H2KG3CCXX:3:1201:15534:20911+ TTTAACTGAGTTCTTACCTGCCTGCCTGGTGTTCTTTGTCTACTGTAAGCAAACAAGAGCTAGCATCATGTCTCTCCTTGGAGGTGTCTGCCTTTCCTTAGATATTGGCTTTGTTGGTTGCCCTACAACCACAGGTCGCTGATCAATTCA
ST-E00192:51:H2KG3CCXX:8:2223:24101:43238+ TAACTGAGTTCTTACCTGCCTGCCTGGTGTTCTTTGTCTACTGTAAGCAAACAAGAGCTAGCATCATGTCTCTCCTTGGAGGTGTCTGCCTTTCCTTAGATATTGGCTTTGTTGGTTGCCCTACAACCACAGGTCGCTGATCAATTCAAG
ST-E00192:51:H2KG3CCXX:1:2220:5547:6776 + GAGTTCTTACCTGCCTGCCTGGTGTTCTTTGTCTACTGTAAGCAAACaAGAGCTAGCATCATGTCTCTCCTTGGaGgTGTCTGCCTTTCCTTAGATATTGGCTTTGTTGGTTGCcCTACAACCACAGgTCGCTGATCAATTCAagAAAAG
ST-E00192:51:H2KG3CCXX:5:1102:27349:28435+ GAGTTCTTACCTGCCTGCCTGGTGTTCTTTGTCTACTGTAAGCAAACAAGAGCTAGCATCATGTCTCTCCTTGGAGGTGTCTGCCTTTCCTTAGATATTGGCTTTGTTGGTTGCCCTACAACCACAGGTCGCTGATCAATTCAAGAAAAG
ST-E00192:51:H2KG3CCXX:8:1123:22091:58955- TGAGCTCACAGTGAGGCCTGTGGGGAAGAACCTGTGAATCTCTCCAAATTCCCTTTGTATGTGTGGCTTCTAATACTTCTATATGCTCACATTCTCCTATGCTCAACATTTAGCAATTTATTAAAAATTTTAACTGAGTTCTTACCTGCC
ST-E00192:51:H2KG3CCXX:5:1214:30069:19258- AGCTCACAGTGAGGCCTGTGGGGAAGAACCTGTGAATCTCTCCAAATTCCCTTTGTATGTGTGGCTTCTAATACTTCTATATGCTCACATTCTCCTATGCTCAACATTTAGCAATTTATTAAAAATTTTAACTGAGTTCTTACCTGCCTG
ST-E00192:51:H2KG3CCXX:2:2205:32820:68782- AtgattggCTCaCAGTGagGcctgtgggGAacAACCttTGAaTcttTCCAAATTCCCTttGTaTGTGTGtCTTCTAATACTTCtATatGCgCgcaTTCTCCtAtgCTCAACAtTcTAGCAAtTTATTAAAAaATTTTagCTGAGTTCTTACC
ST-E00192:51:H2KG3CCXX:5:1105:22173:42921- TCACAGTGAGGCCTGTGGGGAAGAACCTGTGAATCTCTCCAAATTCCCTTTGTATGTGTGGCTTCTAATACTTCTATATGCTCACATTCTCCTATGCTCAACATTTAGCAATTTATTAAAAATTTTAACTGAGTTCTTACCTGCCTGCCT
ST-E00192:51:H2KG3CCXX:2:1123:25583:58251- AAGAaCCtgTGAATCTCTCCAAATTCCCtTTGTATGTGTGGCTTCTAATACTTCTATATGCTCACATTCTCCTATGCTCAACATTTAGCAATTTATTAAAAATTTTAACTGAGTTCTTACCTGCCTGCCTGGTGTTCTTTGTCTACTGTA
ST-E00192:51:H2KG3CCXX:2:2207:29937:29824- CTCTCCAAATTCCCTTTGTATGTGTGGCTTCTAATACTTCTATATGCTCACATTCTCCTATGCTCAACATTTAGCAATTTATTAAAAATTTTAACTGAGTTCTTACCTGCCTGCCTGGTGTTCTTTGTCTACTGTAAGCAAACAAGAGCT
ST-E00192:51:H2KG3CCXX:2:2207:29156:33393- CtctCCAAATTCCCTTTGTATGTGgCTTCTAATACTTCTATATGCTCACATTCTCCTATGCTCAACATTTAGCAATTTATTAAAAATTTTAACTGAGTTCTTACCTGCCTGCCTGGTGTTCTTTGTCTACTGTAAGCAAACAAGAGCT
ST-E00192:51:H2KG3CCXX:5:2118:14103:38333- CCTTTGTATGTGTGGCTTCTAATACTTCTATATGCTCACATTCTCCTATGCTCAACATTTAGCAATTTATTAAAAATTTTAACTGAGTTCTTACCTGCCTGCCTGGTGTTCTTTGTCTACTGTAAGCAAACAAGAGCTAGCATCATGTCT
ST-E00192:51:H2KG3CCXX:6:1218:21939:34571- CTTCTAATACTTCTATATGCTCACATTCTCCTATGCTCAACATTTAGCAATTTATTAAAAATTTTAACTGAGTTCTTACCTGCCTGCCTGGTGTTCTTTGTCTACTGTAAGCAAACAAGAGCTAGCATCATGTCTCTCCTTGGAGGTGTC
ST-E00192:51:H2KG3CCXX:6:1201:23817:71085- CTAATACTTCTATATGCTCACATTCTCCTATGCTCAACATTTAGCAATTTATTAAAAATTTTAACTGAGTTCTTACCTGCCTGCCTGGTGTTCTTTGTCTACTGTAAGCAAACAAGAGCTAGCATCATGTCTCTCCTTGGAGGTGTCTGC
ST-E00192:51:H2KG3CCXX:1:2206:28973:6741 - ACATTCTCCTATGCTCAACATTTAGCAATTTATTAAAAATTTTAACTGAGTTCTTACCTGCCTGCCTGGTGTTCTTTGTCTACTGTAAGCAAACAAGAGCTAGCATCATGTCTCTCCTTGGAGGTGTCTGCCTTTCCTTAGATATTGGCT
ST-E00192:51:H2KG3CCXX:8:2213:29034:43484- ACATTCTCCTATGCTCAACATTTAGCAATTTATTAAAAATTTTAACTGAGTTCTTACCTGCCTGCCTGGTGTTCTTTGTCTACTGTAAGCAAACAAGAGCTAGCATCATGTCTCTCCTTGGAGGTGTCTGCCTTTCCTTAGATATTGGCT
ST-E00192:51:H2KG3CCXX:8:2213:29044:43537- ACATTCTCCTATGCTCAACATTTAGCAATTTATTAAAAATTTTAACTGAGTTCTTACCTGCCTGCCTGGTGTTCTTTGTCTACTGTAAGCAAACAAGAGCTAGCATCATGTCTCTCCTTGGAGGTGTCTGCCTTTCCTTAGATATTGGCT
ST-E00192:51:H2KG3CCXX:2:1123:13667:62893- TCCTATGCTCAACATTTAGCAATTTATTAAAAATTTTAACTGAGTTCTTACCTGCCTGCCTGGTGTTCTTTGTCTACTGTAAGCAAACAAGAGCTAGCATCATGTCTCTCCTTGGAGGTGTCTGCCTTTCCTTAGATATTGGCTTTGTTG
ST-E00192:51:H2KG3CCXX:2:2218:4826:48037 - TATGCTCAACATTTAGCAATTTATTAAAAATTTTAACTGAGTTCTTACCTGCCTGCCTGGTGTTCTTTGTCTACTGTAAGCAAACAAGAGCTAGCATCATGTCTCTCCTTGGAGGTGTCTGCCTTTCCTTAGATATTGGCTTTGTTGGTT
ST-E00192:51:H2KG3CCXX:8:2212:29288:60660- CATTTAGCAATTTATTAAAAATTTTAACTGAGTTCTTACCTGCCTGCCTGGTGTTCTTTGTCTACTGTAAGCAAACAAGAGCTAGCATCATGTCTCTCCTTGGAGGTGTCTGCCTTTCCTTAGATATTGGCTTTGTTGGTTGCCCTACAA
ST-E00192:51:H2KG3CCXX:8:1117:10043:32707- TAGCAATTTATTAAAAATTTTAACTGAGTTCTTACCTGCCTGCCTGGTGTTCTTTGTCTACTGTAAGCAAACAAGAGCTAGCATCATGTCTCTCCTTGGAGGTGTCTGCCTTTCCTTAGATATTGGCTTTGTTGGTTGCCCTACAACCAC
ST-E00192:51:H2KG3CCXX:3:1101:2177:44539 - TTATTAAAAATTTTAACTGAGTTCTTACCTGCCTGCCTGGTGTTCTTTGTCTACTGTAAGCAAACAAGAGCTAGCATCATGTCTCTCCTTGGAGGTGTCTGCCTTTCCTTAGATATTGGCTTTGTTGGTTGCCCTACAACCACAGGTCGC

                 160345620    160345640    160345660    160345680    160345700    160345720    160345740    160345760    160345780    160345800    160345820    160345840    160345860    160345880
chr1:160345603-160345893 GAAAGAGGCTCACAGTGCTGGGATGTCAGCGCAGGGAGGAGAAGGGAGCAAGCAGAGATGACCCTGAAAGGCTGAGAGGGAGTGGAAGAAGGGAGGCAGTGGTGGcagtgcagcaacactgtgctagaggggagaaagcccacacccggcagccagaggtcccgtgtccctgctctgtctccacctttccccttttgtgccacagtgagcaaaccgcatgccctccctgagcaacagctttttcatatggaaagcaggaaagacaacaagacccaccggggtgtgatgagga

ST-E00144:68:H2LCKCCXX:7:1222:16773:53118+ AAGAGGCTCACAGTGCTGGGATGTCAGCGCAGGGAGGAGAAGGGAGCAAGCAGAGATGACCCTGAAAGGCTGAGAGGGAGTGGAAGAAGGGAGGCAGTGGTGGCAGTGCAGCAACACTGTGCTAGAGGGGAGAAAGCCCACACCCGGCAG
```

```
ST-E00144:68:H2LCKCCXX:4:2124:27055:2715 + AGGCTCACAGTGCTGGGATGTCAGCGCAGGGAGGAGAAGGGAGCAAGCAGAGATGAGCCTGAAAGGCTGAGAGGGAGtGGAAGAAGGGAGGCAGTGGTGGCAGTGCAGCAACACTGTGCTAGAGGGGAGAAAGCCCACACCCGGCAGCCA
ST-E00144:68:H2LCKCCXX:6:1202:26913:54718+ TCACAGTGCTGGGATGTCAGCGCAGGGAGGAGAAGGGAGCAAGCAGAGATGAGCCTGAAAGGCTGAGAGGGAGTGGAAGAAGGGAGGCAGTGGTGGCAGTGCAGCAACACTGTGCTAGAGGGGAGAAAGCCCACACCCGGCAGCCAGAGG
ST-E00144:68:H2LCKCCXX:8:1108:11820:57618+ AGTGCTGGGATGTCAGCGCAGGGAGGAGAAGGGAGCAAGCAGAGATGAGCCTGAAAGGCTGaGAGGGAGtGGAAGAAGGGAGGCAGTGGTGGCAGTGCAGCAACACTGTGCTAGAGGGGAGAAAGCCCACACCCGGCAGCCAGAGGTCCC
ST-E00144:68:H2LCKCCXX:8:2217:5151:26062 + TGCTGGGATGTCAGCGCAGGGAGGAGAAGGGAGCAAGCAGAGATGAGCCTGAAAGGCTGAGAGGGAGtGGAAGAAGGGAGGCAGTGGTGGCAGTGCAGCAACACTGTGCTAGAGGGGAGAAAGCCCACACCCGGCAGCCAGAGGTCCCGT
ST-E00144:68:H2LCKCCXX:8:1107:24883:26238+ GCTGGGATGTCAGCGCAGGGAGGAGAAGGGAGCAAGCAGAGATGAGCCTGAAAGGCTGAGAGGGAGTGGAAGAAGGGAGGCAGTGGTGGCAGTGCAGCAACACTGTGCTAGAGGGGAGAAAGCCCACACCCGGCAGCCAGAGGTCCCGTG
ST-E00144:68:H2LCKCCXX:8:1103:25512:66110+ GGATGTCAGCGCAGGGAGGAGAAGGGAGCAAGCAGAGATGAGCCTGAAAGGCTGAGAGGGAGTGGAAGAAGGGAGGCAGTGGTGGCAGTGCAGCAACACTGTGCTAGAGGGGAGAAAGCCCACACCCGGCAGCCAGAGGTCCCGTGTCCC
ST-E00144:68:H2LCKCCXX:3:2204:4765:25851 + GCAGGGAGGAGAAGGGAGCAAGCAGAGATGAGCCTGAAAGGCTGAGAGGGAGtGGAAGAAGGGAGGcAGTGGTGGCAGTGCAGCAACACTGTGCtAGAGGGGAGAAAGCCcACACCCGGCAGCCAGAGGTCccGGgtCCCtGcTctgtCt
ST-E00144:68:H2LCKCCXX:8:2122:30973:32777+ gCAGGGAGGAGAAGGGAGCAAGCAGAGATGAGCCTGAAAGGCTGcGAGGGAGtGGAAGAAGGGaGGCAGTGGTGGCAGtGCAGCAACACTGTGCTAGAGGGGAGAaAGCCcACACCCggCAGCcaGAGGTCCCGTGtCCCtgCtCTgtCT
ST-E00144:68:H2LCKCCXX:1:2211:20214:5493 + GAGAAGGGAGCAAGCAGAGATGAGCCTGAAAGGCTGAGAGGGAGTGGAAGAAGGGAGGCAGTGGTGGCAGTGCAGCAACACTGTGCTAGAGGGGAGAAAGCCCACACCCGGCAGCCAGAGGTCCCGTGTCCCTGCTCTGTCTCCACCTTT
ST-E00144:68:H2LCKCCXX:4:2201:4735:58181 + GAGAAGGGAGCAAGCAGAGATGAGCCTGAAAGGCTGAGAGGGAGTGGAAGAAGGGAGGCAGTGGTGGCAGTGCAGCAACACTGTGCTAGAGGGGAGAAAGCCCACACCCGGCAGCCAGAGGTCCCGTGTCCCTGcTcTGTCTCCACCTTT
ST-E00144:68:H2LCKCCXX:8:1221:13799:50252+ AAGGGAGCAAGCAGAGATGAGCCTGAAAGGCTGAGAGGGAGTGGAAGAAGGGAGGCAGTGGTGGCAGTGCAGCAACACTGTGCTAGAGGGGAGAAAGCCCACACCCGGCAGCCAGAGGTCCCGTGTCCCTGCTCTGTCTCCACCTTTCCC
ST-E00144:68:H2LCKCCXX:3:1204:32079:47176+ AGGGAGCAAGCAGAGATGAGCCTGAAAGGCTGAGAGGGAGTGGAAGAAGGGAGGCAGTGGTGGCAGTGCAGCAACACTGTGCTAGAGGGGAGAAAGCCCACACCCGGCAGCCAGAGGTCCCGTGTCCCTGCTCTGTCTCCACCTTTCCCC
ST-E00144:68:H2LCKCCXX:3:1204:31927:47229+ AGGGAGCAAGCAGAGATGAGCCTGAAAGGCTGAGAGGGAGTGGAAGAAGGGAGGCAGTGGTGGCAGTGCAGCAACACTGTGCTAGAGGGGAGAAAGCCCACACCCGGCAGCCAGAGGTCCCGTGTCCCTGCTCTGTCTCCACCTTTCCCC
ST-E00144:68:H2LCKCCXX:1:1110:21137:17465+ AAGCAGAGATGAGCCTGAAAGGCTGAGAGGGAGTGGAAGAAGGGAGGCAGTGGTGGCAGTGCAGCAACACTGTGCTAGAGGGGAGAAAGCCCACACCCGGCAGCCAGAGGTCCCGTGTCCCTGCTCTGTCTCCACCTTTCCCCTTTGTGC
ST-E00144:68:H2LCKCCXX:8:2201:27532:47352+ GAGATGAGCCTGAAAGGCTGAGAGGGAGTGGAAGAAGGGAGGCAGTGGTGGCAGTGCAGCAACACTGTGCTAGAGGGGAGAAAGCCCACACCCGGCAGCCAGAGGTCCCGTGTCCCTGCtCTGTCTCCACCTTtCCCCTTTGTGCCACAG
ST-E00144:68:H2LCKCCXX:1:1117:21726:32197+ aGCCTGAAAGGCTGAGAGGGAGTGGAAGAAGGGAGGCAGTGGTGGCAGTGCAGCAACACTGTGCTAGAGGGGaGAAAGCCCACACCCGGCAGCCAGAGGTCCCGTGTCCCTGCTCTGTCTCCACCTTTCCCCTTTGTGCCACAGTGAGCA
ST-E00144:68:H2LCKCCXX:1:1117:21929:32408+ AGCCTGAAAGGCTGAGAGGGAGTGGAAGAAGGGAGGCAGTGGTGGCAGTGCAGCAACACTGTGCTAGAGGGGAGAAAGCCCACACCCGGCAGCCAGAGGTCCCGTGTCCCTGCTCTGTCTCCACCTTTcCCCTTTGTGCCACAGTGaGCA
ST-E00144:68:H2LCKCCXX:6:2118:23573:1889 + AAAGGCTGAGAGGGAGTGGAAGAAGGGAGGCAGTGGTGGCAGTGCAGCAACACTGTGCTAGAGGGGAGAAAGCCCACACCCGGCAGCCAGAGGTCCCGTgTCCCTGCgCtGTCTCCACCttTCCCctttatacCACacggagCAAcccgc
ST-E00144:68:H2LCKCCXX:4:2114:11160:73353+ CTGAGAGGGAGTGGAAGAAGGGAGGCAGTGGTGGCAGTGCAGCAACACTGTGCTAGAGGGGAGAAAGCCCACACCCGGCAGCCAGAGGTCCCGTGTCCCTGCTCTGTCTCCACCTTTCCCCTTTGTGCCACAGTGAGCAAACCGCATGCC
ST-E00144:68:H2LCKCCXX:1:1122:7719:56001 + TgAGAGGGAGTGGAAGAAGGGAGGCAGTGGTGGCAGTGCAGCAACACTGTGCTAGAGGGGAGAAAGCCCACACCCGGCAGCCAGAGGTCCCGTGTCCCTGCTCTGTCTCCACCTTTCCCCTTTGTGCCACAGTGAGCAAACCGCAtGcCc
ST-E00144:68:H2LCKCCXX:8:2209:22203:53522+ TGAGAGGGAGTGGAAGAAGGGAGGCAGTGGTGGCAGTGCAGCAACTCTGTGCTAGAGGGAGaGAAAGCCCACACCCGGCAGCCAGAGGtCCCGTGTCtctGCTCTGTCTCCACCTTTCCCCTttGTGCCACAGTGAGCAAACCGCATGCCC
ST-E00144:68:H2LCKCCXX:7:1101:16123:26079+ AGGGAGTGGAAGAAGGGAGGCAGTGGTGGCAGTGCAGCAACACTGTGCTAGAGGGGAGAAAGCCCACACCCGGCAGCCAGAGGTCCCGTGTCCCTGCTCTGTCTCCACCTTTCCCCTTTGTGCCACAGTGAgCAaACcgCATGCccTccC
ST-E00144:68:H2LCKCCXX:1:2221:11332:57759+ GTGGAAGAAGGGAGGCAGTGGTGGCAGTGCAGCAACACTGTGCTAGAGGGGAGAAAGCCCACACCCGGCAGCCAGAGGTCCCGTGTCCCTGCTCTGTCTCCACCTTTCCCCTTTGTGCCACaGTGaGCaAACCGCATGCCCTCCCTGaGc
ST-E00144:68:H2LCKCCXX:4:2209:31703:44978+ GGAAGAAGGGAGGCAGTGGTGGCAGTGCAGCAACACTGTGCTAGAGGGGAGAAAGCCCACACCGGCaGCCAGAGGTCCCGTGTCCCTGaTctGTcTCaaacTTTcCcctTTgtGtaacagtGggcgaaacgcatGcctagactggacga
ST-E00144:68:H2LCKCCXX:6:2220:23086:2240 + GGAAGAAGGGAGGCAGTGGTGGCAGTGCAGCAACACTGTGCTAGAGGGGAGAAAGCCCACACCCGGCAGcCAGAGtTCCCGTGTCCCTGCTCTGTCTCCACCTTTCCCCTTTGTGCCACAGTGAGCAAACCGCATGCCCTCCCTGcGCGA
ST-E00144:68:H2LCKCCXX:1:1213:14641:9870 + GAAGAAGGGAGGCAGTGGTGGCAGTGCAGCAACACTGTGCTAGAGGGGAGAAAGCCCacACCCGGCAGCCAGAGGTCCCGTGTCCCTGCTCTGTCTCCACCTTTCCgCtTTGTGCCACAgagAgCAAaCCGCatgCCCTCCCtcagccac
ST-E00144:68:H2LCKCCXX:3:1209:4999:43765 + AGAAGGGAGGCAGTGGTGGCAGTGCAGCAACACTGTGCTAGAGGGGAGAAAGCCCACACCCGGCAGCCAGAGGTCCCGTGTCCCTGCTCTGTCTCCACCTTTCCCCTTTGTGCCACAGTGAGCAAACCGCATGCCCTCCCTGAGCgACAG
ST-E00144:68:H2LCKCCXX:8:2119:29887:54138+ GAAGGGAGGCAGTGGTGGCAGTGCAGCAACACTGTGCTAGAGGGGAGAAAGCCCACACCCGGCAGCCAGAGGTCCCGTGTCCCTGCTCTGTCTCCACCTTTCCCCTTTGTGCCACAGTGAGCAAACCGCATGCCCTCCCTGAGCGACAGC
ST-E00144:68:H2LCKCCXX:8:2119:29856:54366+ GAAGGGAGGCAGTGGTGGCAGTGCAGCAACACTGTGCTAGAGGGGAGAAAGCCCACACCCGGCAGCCAGAGGTCCCGTGTCCCTGCTCTGTCTCCACCTTTCCCCTTTGTGCCACAGTGAGCAAACCGCATGCCCTCCCTGAGCGACAGC
ST-E00144:68:H2LCKCCXX:1:2211:26101:62980+ GAGGCAGTGGTGGCAGTGCAGCAACACTGTGCTAGAGGGGAGAAAGCCCACACCCGGCAGCCAGAGGTCCCGTGTCCCTGCTCTGTCTCCACCTTTCCCCTTTGTGCCACAGTGAGCAAACCGCATGCCCTCCCTGAGCGACAGCTTTTT
ST-E00144:68:H2LCKCCXX:8:1201:10561:25482+ ggCaGtgGTggCagTGcAGCaACacTGgGccaGAGGGGcGgaagggcccaCcCggcaGCcagacGTCCCGtGTcCCggCTccgtcgCaCcttTccCccatgTGCcaCAGcAGcAAaccgCcggcgCacgccgcgCGAacacttgcaca
ST-E00144:68:H2LCKCCXX:8:2121:8734:39792 + AGTGGTGGCAGTGCAGCAACACTGTGCTAGAGGGGAGAAAGCCCACACCCGGCAGCCAGAGGTCCCGTGTCCCTGCTCTGTCTCCACCTTTCCCCTTTGTGCCACAGTGAGCAAACCGCATGCCCTCCCTGaGCGacaGCTTTTtCatAT
ST-E00144:68:H2LCKCCXX:8:2121:8785:39880 + AGTGGTGGCAGTGCAGCAACACTGTGCTAGAGGGGAGAAAGCCCACACCCGGCAGCCAGAGGTCCCGTGTCCCTGCTCTGTCTCCACCTTTCCCCTTTGTGCCACAGTGAGCAAACCGCATGCCCTCCCTGAGCGaCAGCTTTttCATaT
ST-E00144:68:H2LCKCCXX:1:1217:25147:67516+ GTGGCAGTGCAGCAACACTGTGCTAGAGGGGAGAAAGCCCACACCCGGCAGCCAGAGGTCCCGTGTCCCTGCTCTGTCTCCACCTTTCCCCTTTGTGCCACAGTGAGCAAACCGCATGCCCTCCCTGAGCGACAGCTTTTTCATATGGA
ST-E00144:68:H2LCKCCXX:1:2217:32789:13000+ cAGCAACaCTGTGCTAGAgGGGcGAAAGCCCACACCCGGCAGCCAGAGGTCCCGTGTCCcTGCTCTgTgtCcACCTTTcctCtTTTgTgctaagGgGgggatACGGaagccctgcctgggcggagagttTTTcggaTtggagggaggg aaG
ST-E00144:68:H2LCKCCXX:7:1204:18559:63385+ CAGCAACACTGTGCTAGAGGGGAGAAAGCCCACACCCGGCAGCCAGAGGTCCCGTGTCCCTGCTCTGTCTCCACCTTTCCCCTTTGTGCCACAGTGAGCAAACCGCATGCCCTCCCTGAGCGACAGCTTTTTCATATGGAAAGCAGGAAA
ST-E00144:68:H2LCKCCXX:1:1213:23309:69819+ ACTGTGCTAGAGGGGAGAAAGCCCACACCCGGCAGCCAGAGGTCCCGTGTCCCTGCTCTGTCTCCACCTTTCCCCTTTGTGCCACAGTGAGCAAACCGCATGCCCTCCCTGAGCGACAGCTTTTTCATATGGAAaGCAGGAAAGACAACA
ST-E00144:68:H2LCKCCXX:8:2207:28384:46965+ ACTGTGCTAGAGGGGAGAAAGCCCACACCCGGCAGCCAGAGGTCCCGTGTCCCTGCTCTGTCTCCACCTTTCCCCTTTGTGCCACAGTGAGCAAACCGCATGCCCTCCCTGAGCGACAGCTTTTTCAtATGGAAAGCAGGAAAGACAACA
ST-E00144:68:H2LCKCCXX:4:1207:12550:35397+ AGGGGaGacaGcCCACACCcGGCAGCCAGaGGTCCCGTGTCCCTGCTCTGTCTCCAgCTtCCCCTTtGTGCCACAGTGAGCAAACCGcATgcCCTCCCTGcgCGacaGctTTTTCATATGGAAAGCAGGAAAGACaacAcgacCcaccG
ST-E00144:68:H2LCKCCXX:1:1222:28943:47018+ GGAGAAAGCCCACACCCGGCAGCCAGAGGTCCCGTGTCCCTGCTCTGTCTCCACCTTTCCCCTTTGTGCCACAGTGAGCAAACCGCATGCCCTCCCTGAGCGACAGCTTTTTCATATGGAAAGCAGGAAAGaCAACAAGacCcaccGG
ST-E00144:68:H2LCKCCXX:1:1216:31399:37173- AAAgagGctcacaGtGGTGGGATGTcAGcGcaGGGAGGAGAAGGGaGCAAGCAGAGATGAGCCTGAAAGGCTGAGAGGGAGTGGAAGAAGGGAGGCAGTGGTGGCAGTGCAGCAACACTGTGCTAGAGGGGAGAAAGCCCACACCCGGCA
ST-E00144:68:H2LCKCCXX:6:2201:32038:42816- AGaGGCTCACAgTgctgGgaTGTcAGCGCAGGGAGGAGAAGGGAGCGCAAGCAgAGATGAGCCTGAAAGGCTGAGAGGGAGTGGAAGAAGGGAGGCAGTGGTGGCAGTGCAGCAACACTGTGCTAGAGGGGAGAAAGCCCACACCCGGCAGC
ST-E00144:68:H2LCKCCXX:4:2201:12002:53294- CTCACAGTGCTGGGATGTcCAGCGCAGGGAGGAGAAGGGAGCAAGCAGAGATGAGCCTGAAAGGCTGAGAGGGAGTGGAAGAAGGGAGGCAGTGGTGGCAGTGCAGCAACACTGTGCTAGAGGGGAGAAAGCCCACACCCGGCAGCCAGAG
ST-E00144:68:H2LCKCCXX:8:2214:31237:19276- AggGCGGGGAtGgCAGcGCAGGGAGGAGAAGGGAGCAAGCAGAGATGAGccTGAAAGGCTGAGAGGGAGTGGAAGAAGGGAGGCAGTGGTGGCAGTGCAGCAACACTGTGCTAGAGGGGAGAAAGCCCACACCCGGCAGCCAGAGGTCCC
ST-E00144:68:H2LCKCCXX:4:1203:20731:12683- GgATGTCAGCGCAGGGAGGAGAAGGGAGCAAGCAGAGATGAGCCTGAAAGGCTGAgaGGgagTGGAAGAAGGGAGGCAGTGGTGGCAGTGCAGCAACACTGTGCTAGAGGGGAGAAAGCCCACACCCGGCAGCCAGAGGTCCCGTGTCCc
ST-E00144:68:H2LCKCCXX:7:2113:30810:37208- AtgtCAGcGCAgggaGGAGAAGGGAGCAAGCAgaGatGAGcCTgAcagGgtGAGAGGGAGtGGaaGAAGGGAGGCAGTGGTGGCAGTGCAGCaACaCTGtGCTAGAGGGGAGAAAGCCCACACCCGGCAGCCAGAGGTCCCGTGTCCCTG
ST-E00144:68:H2LCKCCXX:8:1124:28131:42165- gaacagtgctaccactgcataaatgaaAgcgCAgGGaagaGaAaggaggcAgCagAGatgcgcctGAAAGGCTGAGAGGGAGTGGAAGAAGGGaggcaGTGGTGGCAGTGCAGCAACACTGTGCTAGAGGGGAGAAAGCCCACACCCGGC
ST-E00144:68:H2LCKCCXX:1:2101:6115:58884 - CGcAGGGAGGAGAaGGGAGCAAGCAGAGagGAGCCTGAAAGGCTGAGAGGGAGTGGAAGAAGGGAGGCAGTGGTGGCAGTGCAGCAACACTGTGCTAGAGGGGAGAAAGCCCACACCCGGCAGCCAGAGGTCCCGTGTCCCTGCTCTGTC
ST-E00144:68:H2LCKCCXX:1:2105:7719:6249  - AGGGAGGAGAAGGGAGCAAGCAGAGATGAGCCTGAAAGGCTGAGAGGGAGTGGAAGAAGGGAGGCAGTGGTGGCAGTGCAGCAACACTGTGCTAGAGGGGAGAAAGCCCACacCCGGCAGCCAGAGGTCCCGTGTCCCTGCTCTGTCTCC
ST-E00144:68:H2LCKCCXX:1:2105:7658:6460  - AGGGAGGAGAaGGGAGCAAGCAGAGATGAGCCtGaaaGGCTGAGAGGGAGTGGAAGAAGGGAGGCAGTGGTGGCAGTGCAGCAACACTGTGCTAGAGGGGAGAAAGCCCACACCCGGCAGCCAGAGGTCCCGTGTCCCTGCTCTGTCTCC
ST-E00144:68:H2LCKCCXX:3:1209:17473:7708 - GGGaGGaGAAGGGAGCAAGCAGAGATGAGCCTGAAAGGCTGAGAGGGAGtGGAAGAAGGGAGGCAGTGGTGGCAGTGCAGCAACACTGTGCTAGAGGGGAGAAAGCCCACACCCGGCAGCCAGAGGTCCCGTGTCCCTGCTCTGT
ST-E00144:68:H2LCKCCXX:3:1209:14590:32215- GAGGAGaAGGGAGCAAGCAGAGATGAGCCTGAAAGGCTGAGAGGGAGTGGAAGAAGGGAGGCAGTGGTGGCAGTGCAGCAACACTGTGCTAGAGGGGAGAAAGCCCACACCCGGCAGCCAGAGGTCCCGTGTCCCTGCTCTGTCTCCACC
ST-E00144:68:H2LCKCCXX:3:1209:14641:32232- GAGGAGAAGGGAGCAAGCAGAGATGAGCCTGAAAGGCTGAGAGGGAGTGGAAGAAGGGAGGCAGTGGTGGCAGTGCAGCAACACTGTGCTAGAGGGGAGAAAGCCCACACCCGGCAGCCAGAGGTCCCGTGTCCCTGCTCTGTCTCCACC
ST-E00144:68:H2LCKCCXX:8:2116:11038:14599- GAGGAGAAGGGAGCAAGCAGAGATGAGCCTGAAAGGCTGAGAGGGAGTGGAAGAAGGGAGGCAGTGGTGGCAGTGCAGCAACACTGTGCTAGAGGGGAGAAAGCCCACACCCGGCAGCCAGAGGTCCCGTGTCCCTGCTCTGtCTCCACC
ST-E00144:68:H2LCKCCXX:8:1119:32079:15918- tGGcAgcaaGCagAgatggGCCTGAaagGCTgAgaGgGagtggAAGAAGGGAcGcaGTGGTgGCagTGcAGCAaCacTGTgTGCTAGAGGGGGAGAAaGCccaCacCCGGCAGCCAGAGGTCCCgtGTCCCTGCTCTGTCTCCACCTTTCCCC
ST-E00144:68:H2LCKCCXX:4:1124:23857:41023- GCAAGCAGAGATGAGCCTGAAAGGCTGAGAGGGAGTGGAAGAAGGGAGGCAGTGGTGGCAGTGCAGCAACACTGTGCTAGAGGGGAGAAAGCCCACACCCGGCAGCCAGAGGTCCCGTGTCCCTGCTCTGTCTCCACCTTTCCCCTTTGT
ST-E00144:68:H2LCKCCXX:6:1215:7191:19575 - aGGCTGAGAGGGAGTGGaAGAAGGGAGGCagtgGTGGCAGTGCAGCAACACTGTGCtagAgGGGGAGAAAGCCCACACCCGGCAGCCAGAGGTCCCGTGTCCCTGCTCTGTCTCCACCTTTCCCCtTTGTGCCACAGTGAGCAAACCGCAT
ST-E00144:68:H2LCKCCXX:7:2114:7110:68624 - GCTGAGAGGGAGTGGAAGAAGGGAGGCAGTGGTGGCAGTGCAGCAACACTGTGCTAGAGGGGAGAAAGCCCACACCCGGCAGCCAGAGGTCCCGTGTCCCTGCTCTGTCTCCACCTTTCCCCTTTGTGCCACAGTGAGCAAACCGCATGC
ST-E00144:68:H2LCKCCXX:7:2214:2471:52995 - GAGagggAGTGGaAGAaGGGAGGcAGTGGTGGcaGTGCAGCAACACTGtGCTAGAGGGGAGAAaGCCCACACCCGGCAGCCAGAGGTCCCGTGTCCCTGCTCTGTCTCCACCTTTCCCCTTTGTGCCACAGTGAGCAAACCGCATGCCCT
ST-E00144:68:H2LCKCCXX:7:2217:32475:58691- GGGAGTgGaAGaaGgGAGGCAGTGGTGGCAGTGCAGCAACACTGTGCTAGAGGGGAGAAAGCCCACACCCGGCAGCCAGAGGTCCCGTGTCCCTGCTCTGTCTCCACCTTTCCCCTTTGTGCCACAGTGAGCAAACCGCATGCCCTCCC
ST-E00144:68:H2LCKCCXX:8:2201:24943:60730- GGGAGTGGAAGAAGGGAGGCAGTGGTGGCAGTGCAGCAACACTGTGCTAGAGGGGAGAAAGCCCACACCCGGCAGCCAGAGGTCCCGTGTCCCTGCTCTGTCTCCACCTTTCCCCTTTGTGCCACAGTGAGCAAACCGCATGCCCTCCCT
ST-E00144:68:H2LCKCCXX:8:2222:28993:67604- GGGAGTGGAAGAAGGGAGGCAGTGGTGGCAGTGCAGCAACACTGTGCTAGAGGGGAGAAAGCCCACACCCGGCAGCCAGAGGTCCCGTGTCCCTGCTCTGTCTCCACCTTTCCCCTTTGTGCCACAGTGAGCAAACCGCATGCCCTCCCT
ST-E00144:68:H2LCKCCXX:6:2118:7201:15232 - GTGGAAGAAGGGAGGCAGTGGTGGCAGTGCAGCAACACTGTGCTAGAGGGGAGAAAGCCCACACCCGGCAGCCAGAGGTCCCGTGTCCCTGCTCTGTCTCCACCTTTCCCCTTTGTGCCACAGTGAGCAAACCGCATGCCCTCCCTGAGC
ST-E00144:68:H2LCKCCXX:6:2211:26050:1326 - gggga agaaGgGAGGCAGTgGTGGCAGtGCAGcaACactGTGCTAGAGGGGAGAaAGcCCaCacCCGGCAGCCAGAGGTCCCGTGTCCCTGCTCTGTCTCCACCTTTCCCCTTTGTGCCACAGTGAGCAAACCGCATGCCCTCCCTGAGC
ST-E00144:68:H2LCKCCXX:8:1116:13200:38667- gtgGAAGAAGGgaGGCAgtgGtggccGTGCAGCAACaCTgTgCTagAGGGGagaaAGcCCaCACCCGgcagCtagAagtCCCgTgtCCCTgCTCtGtCtcCaCcTttCCCCTTTGTgCcAGTGAgCAaaCCGCATgcccTcccTGagC
ST-E00144:68:H2LCKCCXX:4:1112:11170:8253 - GAAGAAGGGAGGCAGTGGTGGCAGTGCAGCAACACTGTGCTAGAGGGGAGAAAGCCCACACCCGGCAGCCAGAGGTCCCGTGTCCCTGCTCTGTCTCCACCTTTCCCCTTTGTGCCACAGTGAGCAAACCGCATGCCCTCCCTGAGCGAC
ST-E00144:68:H2LCKCCXX:6:1208:12215:8024 - GAAGaAGGGAGGCAGTGGTGGCAGTGCAGCAACACTGTGCTAGAGGGGAGAAAGCCCACACCCGGCAGCCAGAGGTCCCGTGTCCCTGCTCTGTCTCCACCTTTCCCCTTTGTGCCACAGTGAGCAAACCGCATGCCCTCCCTGAGCGAC
ST-E00144:68:H2LCKCCXX:3:2212:20853:36628- GCAGTGGTGGCAGTGCAGCAACACTGTGCTAGAGGGGAGAAAGCCCACACCCGGCAGCCAGAGGTCCCGTGTCCCTGCTCTGTCTCCACCTTTCCCCTTTGTGCCACAGTGAGCAAACCGCATGCCCTCCCTGAGCGACAGCTTTTTCAT
ST-E00144:68:H2LCKCCXX:8:1115:6491:61539 - TgGTGGCAGTGCAGCAACACTGTGCTAGAGGGGAGAAAGCCCACACCCGGCAGCCAGAGGTCCCGTGTCCCTGCTCTGTCTCCACCTTTCCCCTTTGTGCCACAGTGAGCAAACCGCATGCCCTCCCTGAGCGACAGCTTTTTCATaTGG
ST-E00144:68:H2LCKCCXX:7:2121:31846:42447- GGCAGTGCAGCAACACTGTGCTAGAGGGGAGAAAGCCCACACCCGGCAGCCAGAGGTCCCGTGTCCCTGCTCTGTCTCCACCTTTCCCCTTTGTGCCACAGTGAGCAAACCGCATGCCCTCCCTGAGCGACAGCTTTTTCATATGGAAAG
ST-E00144:68:H2LCKCCXX:7:1217:14225:65618- CgGtGCTagcGGGGAgaaagcccccaCCCGgCAGCcaGcGgtCCCGTGtcCCTGCTCTGtCtccaCctTTCCCCTtGtGCCACAGTgAGccAACCGCATgCCCTCCCTGAGCGACAGCtTttTCATATGGAAAGCAGGAaAGACAACAA
ST-E00144:68:H2LCKCCXX:8:1209:7749:53346 - GAGGGGAGAAAGCCCACACCCGGCAGCCAGAGGTCCCGTGTCCCTGCTCTGTCTCCACCTTTCCCCTTTGTGCCACAGTGAGCAAACCGCATGCCCTCCCTGAGCGACAGCTTTTTCATATGGAAAGCAGGAAAGACAACAAGACCCACC
ST-E00144:68:H2LCKCCXX:7:2108:14134:12050- AGGGGaGAaaGCCCACACCCGGCAGCCAGAGGTCCCGTGTCCCTGCTCTGTCTCCACCTTTCCCCTTTGTGCCACAGTGAGCAAACCGCATGCCCTCCCTGAGCGACAGCTTTTTCATATGGAAAGCAGGAAAGACAACAAGACCCACCG
ST-E00144:68:H2LCKCCXX:7:2208:6288:72790 - AGCCcgCaCcCGGcaGCcAGAGGTCCCGTGTCCCTGCTCTGTCTCCACCTTTCCCCTTTGTGCCACAGTGAGCAAACCGCATGCCCTCCCTGAGCGACAGCTTTtTCATATGGAAAGCAGGAAAGACAACAAGACCCACCGGGGTGTGAT
ST-E00144:68:H2LCKCCXX:6:1221:25309:41462- GCCCaCaCCcgGCAGCCAGAGGtCCCGTGTCCCTGCTCTGTCTCCACCTTTCCCCTTTGTGCCACAGTGAGCAAaCCGCATGCCCTCCCTGAGCGACAGCTTTTTCATATGGAAAGCAGGAAAGACAACAAGACCCACCGGGGTGTGATG
ST-E00144:68:H2LCKCCXX:6:2123:3750:21069 - aCaCCCgGCAGCCAGAggTCCCGTGTCCCTGCTCTGTCTCCACctTTCCCCTTTGTGCCACAGTGAGCAAACCGCATGCCCTCCcTgAGCgACAGCTTTTTCATATGGAAAGCAGGAAAGACAACAAGACCCACCGGGGTGTGATGAGGA
ST-E00144:68:H2LCKCCXX:7:2107:8186:56739 - ATGATGCTAGCTCTTGTTTGCTTACAGTAGACAAAGAACACCAGCCAGGCAGGGGTCCCGTGTCCCTGCTCTGTCTCCACCTTtCCCCTTTGTGCCACAGTGAGCAAACCGCATGCCCTCCCTGAGCGACAGCTTTTTCATATGGAAAGC
ST-E00144:68:H2LCKCCXX:7:2107:8277:57636 - ATGATGCTaGCTCTTGtTTGCTTACAGTAGACAAAGAACACCAGCCAGGCAGGGGTCCCGTGTCCCTGCTCTGTCTCCACCTTTCCCCTTTGTGCCACAGTGAGCAAACCGCATGCCCTCCCTGAGCGACAGCTTTTTCATATGGAAAGC
ST-E00144:68:H2LCKCCXX:7:2107:8318:57847 - ATGATGCTAGCTCTTGTTTGCTTACAGTAGACAAAGAACACCAGCCAGGCAGGGGTCCCGTGTCCCTGCTCTGTCTCCACCTTtCCCCTTTGTGCCACAGTGAGCAAACCGCATGCCCTCCCTGAGCGACAGCTTTTTCATATGGAAAGC
```

```
ST-E00192:51:H2KG3CCXX:2:2105:29694:30281+ AAAGAGGCTCACAGTGCTGGGATGTCAGCGCAGGGAGGAGAAGGGAGCAAGCAGAGATGAGCCTGAAAGGCTGAGAGGGAGtGGAAGAAGGGAGGCAGTGGTGGCAGTGCAGCAACACTGTGCTAGAGGGGAGAAAGCCCACACCCggCA
ST-E00192:51:H2KG3CCXX:2:2105:29633:30562+ AAAGAGGCTCACAGTGCTGGGATGTCAGCGCAGGGAGGAGAAGGGAGCAAGCAGAGATGAGCCTGAAAGGCTGAGAGGGAGTGGAAGAAGGGAGGCAGTGGTGGCAGTGCAGCAACACTGTGCTAGAGGGGAGAAAGCCCACACCCGGCA
ST-E00192:51:H2KG3CCXX:8:1115:1791:8886  + GAGGCTCACAGTGCTGGGATGTCAGCGCAGGGAGGAGAAGGGAGCAAGCAGAGATGAGCCTGAAAGGCTGAGAGGGAGTGGAAGAAGGGAGGCAGTGGTGGCAGTGCAGCAACACTGTGCTAGAGGGGAGAAAGCCCACACCCGGCAGCC
ST-E00192:51:H2KG3CCXX:1:2202:26527:70188+ GGCTCACAGTGCTGGGATGTCAGCGCAGGGAGGAGAAGGGAGCAAGCAGAGATGAGccTGAAAGGCTGAGAGGGAGTGGAAGAAGGGAGGCAGTGGTGGCAGTGCAGCAACACTGTGCTAGAGGGGAGAAAGCagaCACCaGGcaGCcAG
ST-E00192:51:H2KG3CCXX:8:1108:2918:31371 + CAGTGCTGGgATGTCAGCGCAGGGaGGcGAAGGGAGCAAGCAGaGATGagCCTGAAAGGCTGaGaggGaGtGgAAGAAGGGAGGCAGTGGTGGCAGTGCAGCAACACTgTGCTAGAGGGgAGAAAGCCCcaCaCCCGgCAgcCAGaGgtCC
ST-E00192:51:H2KG3CCXX:1:1214:22589:7989 + GTGCTGGGATGTCAGCGCAGGGAGGAGAAGGGAGCAAGCAGAGATGAGCCTGAAAGGCTGAGAGGGAGTGGAAGAAGGGAGGCAGTGGTGGCAGTGCAGCAACACTGTGCTAGAGGGGAGAAAGCCCACACCCGGCAGCCAGAGGTCCCG
ST-E00192:51:H2KG3CCXX:2:2212:4288:38350 + GTGCTGGGATGTCAGCGCAGGGAGGAGAAGGGAGCAAGCAGAGATGAGCCTGAAAGGCTGAGAGGGAGTGGAAGAAGGGAGGCAGTGGTGGCAGTGCAGCAACACTGTGCTAGAGGGGAGAAAGCCCACACCCGGCAGCCAGAGGTCCCG
ST-E00192:51:H2KG3CCXX:1:2119:8003:69854 + TGGGATGTCAGCGCAGGGAGGAGAAGGGAGCAAGCAGAGATGAGCCTGAAAGGCTGAGAGGGAGTGGAAGAAGGGAGGCAGTGGTGGCAGTGCAGCAACACTGTGCTAGAGGGGAGAAAGCCCACACCCGGCAGCCAGAGGTCCCGTGTC
ST-E00192:51:H2KG3CCXX:2:1213:15230:61416+ ATGTCAGCGCAGGGAGGAGAAGGGAGCAAGCAGAGATGAGCCTGAAAGGCTGAGAGGGAGTGGAAGAAGGGAGGCAGTGGTGGCAGTGCAGCAACACTGTGCTAGAGGGGAGAAAGCCCACACCCGGCAGCCAGAGGTCCCGTGTCCCTG
ST-E00192:51:H2KG3CCXX:6:2123:13220:14617+ GAGGAGAAGGGAGCAAGCAGAGATGAGCCTGAAAGGCTGAGAGGGAGTGGAAGAAGGGAGGCAGTGGTGGCAGTGCAGCAACACTGTGCTAGAGGGGAGAAAGCCCACACCCGGCAGCCAGAGGTCCCGTGTCCCTGCTCTGTCTCCACC
ST-E00192:51:H2KG3CCXX:3:2213:12845:47158+ AGGAGAAGGGAGCAAGCAGAGATGAGCCTGAAAGGCTGAGAGGGAGTGGAAGAAGGGAGGCAGTGGTGGCAGTGCAGCAACACTGTGCTAGAGGGGAGAAAGCCCACACCCGGCAGCCAGAGGTCCCGTGTCCCTGCTCTGTCTCcACCT
ST-E00192:51:H2KG3CCXX:3:2213:13139:47492+ AGGAGAAGGGAGCAAGCAGAGATGAGCCTGAAAGGCTGAGAGGGAGTGGAAGAAGGGAGGCAGTGGTGGCAGTGCAGCAACACTGTGCTAGAGGGGAGAAAGCCCACACCCGGCAGCCAGAGGTCCCGTGTCCCTGCTCTGTCTCCACCT
ST-E00192:51:H2KG3CCXX:3:2213:13119:47527+ AGGAGAAGGGAGCAAGCAGAGATGAGCCTGAAAGGCTGAGAGGGAGTGGAAGAAGGGAGGCAGTGGTGGCAGTGCAGCAACACTGTGCTAGAGGGGAGAAAGCCCACACCCGGCAGCCAGAGGTCCCGTGTCCCTGCTCTGTCTCCACCT
ST-E00192:51:H2KG3CCXX:3:2213:12814:47738+ AGGAGAAGGGAGCAAGCAGAGATGAGCCTGAAAGGCTGAGAGGGAGTGGAAGAAGGGAGGCAGTGGTGGCAGTGCAGCAACACTGTGCTAGAGGGGAGAAAGCCCACACCCGGCAGCCAGAGGTCCCGTGTCCCTGCTCTGTCTCCACCT
ST-E00192:51:H2KG3CCXX:8:2220:12621:32848+ GGAGAAGGGAGCAAGCAGAGATGAGCCTGAAAGGCTGAGAGGGAGTGGAAGAAGGGAGGCAGTGGTGGCAGTGCAGCAACACTGTGCTAGAGGGGAGAAAGCCCACACCCGGCAGCCAGAGGTCCCGTGTCCCTGCTCTGTCTCCACCTT
ST-E00192:51:H2KG3CCXX:2:2219:29207:69274+ GAGAAGGGAGCAAGCAGAGATGAGCCTGAAAGGCTGAGAGGGAGTGGAAGAAGGGAGGCAGTGGTGGCAGTGCAGCAACACTGTGCTAGAGGGGAGAAAGCCCACACCCGGCAGCCAGAGGTCCCGTGTCCCTGCTCTGTCTCCACCTTT
ST-E00192:51:H2KG3CCXX:1:2218:25674:65020+ AAGCAGAGATGAGCCTGAAAGGCTGAGAGGGAGTGGAAGAAGGGAGGCAGTGGTGGCAGTGCAGCAACACTGTGCTAGAGGGGAGAAAGCCCACACCCGGCAGCCAGAGGTCCCGTGTCCCTGCTCTGTCTCCACCTTTCCCCTTTGTGC
ST-E00192:51:H2KG3CCXX:1:2213:14773:22264+ GATGAGCCTGAAAGGCTGAGAGGGAGTGGAAGAAGGGAGGCAGTGGTGGCAGTGCAGCAACACTGTGCTAGAGGGGAGAAAGCCCACACCCGGCAGCCAGAGGTCCCGTGtcCCTGCTCTGTCTCCACCtTtCCCCTttGtgCCaCAgtg
ST-E00192:51:H2KG3CCXX:1:2205:18285:1871 + TGAGCCTGAAAGGCTGAGAGGGAGTGGAAGAAGGGAGGCAGTGGTGGCAGTGCAGCAACACTGTGCTAGAGGGGAGAAAGCCCACACCCGGCAGCCAGAGGTCCCGTGTCCCTGCTCTGTCTCCACCTTTCCCCTTTGTGCCACAGTGAG
ST-E00192:51:H2KG3CCXX:2:2120:14682:69046+ AGAGGGAGTGGAAGAAGGGAGGCAGTGGTGGCAGTGCAGCAACACTGTGCTAGAGGGGAGAAAGCCCACACCCGGCAGCCAGAGGTCCCGTGTCCCTGCTCTGTCTCCACCTTTCCCCTTTGTGCCACAGTGAGCAAACCGCATGCCCTC
ST-E00192:51:H2KG3CCXX:5:1102:19625:68184+ GAGTGGAAGAAGGGAGGCAGTGGTGGCAGTGCAGCAACACTGTGCTAGAGGGGAGAAAGCCCACACCCGGCAGCCAGAGGTCCCGTGTCCCTGCTCTGTCTCCACCTTTCCCCTTTGTGCCACAGTGAGCAAACCGCATGCCCTCCCTGA
ST-E00192:51:H2KG3CCXX:8:2203:26882:6389 + GGAGGCAGTGGTGGCAGTGCAGCAACACTGTGCTAGAGGGGAGAAAGCCCACACCCGGCAGCCAGAGGTCCCGTGTCCCTGCTCTGTCTCCACCTTTCCCCTTTGTGCCACAGTGAGCAaaCCGCATGCCCTccCTGAGCGACAGCTTT
ST-E00192:51:H2KG3CCXX:3:2218:14398:13386+ AGGCAGTGGTGGCAGTGCAGCAACACTGTGCTAGAGGGGAGAAAGCCCACACCCGGCAGCCAGAGGTCCCGTGTCCCTGCTCTGTCTCCACCTTTCCCCTTTGTGCCACAGTGAGCAAACCGCATGCCCTCCCTGAGCGACAGCTTTTTC
ST-E00192:51:H2KG3CCXX:3:1224:16215:14424+ CAGTGCAGCAACACTGTGCTAGAGGGGAGAAAGCCCACACCCGGCAGCCAGAGGTCCCGTGTCCCTGCTCTGTCTCCACCTTTCCCCTTTGTGCCACAGTGAGCAAACCGCATGCCCTCCCTGAGCGACAGCTTTTTCATATgGAAAGCA
ST-E00192:51:H2KG3CCXX:3:1224:16245:14652+ CAGTGCAGCAACACTGTGCTAGAGGGGAGAAAGCCCACACCCGGCAGCCAGAGGTCCCGTGTCCCTGCTCTGTCTCCACCTTTCCCCTTTGTGCCACAGTGAGCAAACCGCATGCCCTCCCTGAGCGACAGCTTTTTCATATgGAAAGCA
ST-E00192:51:H2KG3CCXX:3:1224:16235:14670+ CAGTGCAGCAACACTGTGCTAGAGGGGAGAAAGCCCACACCCGGCAGCCAGAGGTCCCGTGTCCCTGCTCTGTCTCCACCTTTCCCCTTTGTGCCACAGTGAGCAAACCGCATGCCCTCCCTGAGCGACAGCTTTTtCATATGGAaAGCA
ST-E00192:51:H2KG3CCXX:3:1224:16306:14687+ CAGTGCAGCAACACTGTGCTAGAGGGGAGAAAGCCCACACCCGGCAGCCAGAGGTCCCGTGTCCCTGCTCTGTCTCCACCTTTCCCCTTTGTGCCACAGTGAGCAAACCGCATGCCCTCCCTGAGCGACAGCTTTTTCATATGGAAAGCA
ST-E00192:51:H2KG3CCXX:2:2119:26953:59289+ AGGGGGAGAAAGCCCACACCCGGCAGCCAGAGGTCCCGTGTCCCTGCTCTGTCTCCACCTTTCCCCTTTGTGCCACAGTGAGCAAACCGCATGCCCTCCCTGAGCGACAGCTTTTTCATATGGAAAGCAGGAAAGACAACAAGAccCAC
ST-E00192:51:H2KG3CCXX:1:2209:28740:6723 + GGGGGAGAAAGCCCACACCCGGCAGCCAGAGGTCCCGTGTCCCTGCTCTGTCTCCACCTTTCCCCTTTGTGCCACAGTGAGCAAACCGCATGCCCTCCCTGAGCGACAGCTTTTTCATATGGAAAGCAGGAAAGACAACAAGACCCACC
ST-E00192:51:H2KG3CCXX:2:1220:17899:67340+ GAGAAAGCCCACACCCGGCAGCCAGAGGTCCCGTGTCCCTGCTCTGTCTCCACCTTTCCCCTTTGTGCCACAGTGAGCAAACCGCATGCCCTCCCTGAGCGACAGCTTTTTCATATGGAAAGCAGGAAAGaCAACAAGACCCACCGGGGg
ST-E00192:51:H2KG3CCXX:8:2219:20214:12771+ GCCCACACCCGGCAGCCAGAGGTCCCGTGTCCCTGCTCTGTCTCCACCTTTCCCCTTTGTGCCACAGTGAGCAAACCGCATGCCCTCCCTaCCTGAGCGACAGCTTTTTCATATGGAAAGCAGGAAAGACAACAAGACCCACCGGGGTGTGATG
ST-E00192:51:H2KG3CCXX:8:2116:19696:45734- GAAAGAGGCTCACAGTGCTGGGATGTCAGCGCAGGGAGGAGAAGGGAGCAAGCAGAGATGAGCCTGAAAGGCTGAGAGGGAGTGGAAGAAGGGAGGCAGTGGTGGCAGTGCAGCAACACTGTGCTAGAGGGGAGAAAGCCCACACCCGGC
ST-E00192:51:H2KG3CCXX:1:1214:14865:50024- GGCTCACAGTGCTGGGATGTCAGCGCAGGGAGGAGAAGGGAGCAAGCAGAGATGAGCCTGAAAGGCTGAGAGGGAGTGGAAGAAGGGAGGCAGTGGTGGCAGTGCAGCAACACTGTGCTAGAGGGGAGAAAGCCCACACCCGGCAGCCAG
ST-E00192:51:H2KG3CCXX:1:1214:15230:50164- GGCTCACAGTGCTGGGATGTCAGCGCAGGGAGGAGAAGGGAGCAAGCAGAGATGAGCCTGAAAGGCTGAGAGGGAGTGGAAGAAGGGAGGCAGTGGTGGCAGTGCAGCAACACTGTGCTAGAGGGGAGAAAGCCCACACCCGGCAGCCAG
ST-E00192:51:H2KG3CCXX:8:1219:22244:28629- ACAGTGCTgGGATGTCAGCGCAGGGAGGAGAAGGGAGCAAGCAGAGATGAGCCTGAAAGGCTGAGAGGGAGTGGAAGAAGGGAGGCAGTGGTGGCAGTGCAGCAACACTGTGCTAGAGGGGAGAAAGCCCACACCCGGCAGCCAGAGGTC
ST-E00192:51:H2KG3CCXX:2:2218:4918:18696 - CagcgcAGGGAGGAGAAGGGAGCAAGCAGAgaTGAGcCTGAAAGGCTGAGAGGGAGTGGAAGAAGGGAGGCAGTGGTGGCAGTGCAGCAACACTGTGCTAGAGGGGAGAAAGCCCACACCCGGCAGCCAGAGGTCCCGTGTCCCTGCTCT
ST-E00192:51:H2KG3CCXX:1:1209:26923:48758- CGcAGGGAGGAGAAGGGAGCAAGCAGAGATGAGCCTGAAAGGCTGAGAGGGAGTGGAAGAAGGGAGGCAGTGGTGGCAGTGCAGCAACACTGTGCTAGAGGGGAGAAAGCCCACACCCGGCAGCCAGAGGTCCCGTGTCCCTGCTCTGTC
ST-E00192:51:H2KG3CCXX:5:1102:30962:23126- AggaGAagGgAGtaAGcaGAGATGacCCTGaAAGGCtgAgAGGGaGTGGaAGAAGGGAGGCAGTGGTGGCAGTGCAGCAacactGTGCTAGAGGGGaGAgAAgCCCACacCCGGCAGCCAGAGGTCCCGTGTCCCTGCTCTGTCTCCaCCT
[illegible]
```

```
ST-E00192:51:H2KG3CCXX:5:1102:31277:23495- AGGAGaAgGGAGCAAGCAGAGATGAGCCTGAAAGGCTGAGAGGGAGTGGAAGAAGGGaGGCAGTGGTGGCAGTGCAGCAACACTGTGCTAGAGGGGAGAAAGCCCACACCCGGCAGCCAGAGGTCCCGTGTCCCTGCTCTGTCTCCACCT
ST-E00192:51:H2KG3CCXX:8:1223:4877:43167 - GGAGCAAGCAGAGATGAGCCTGAAAGGCTGAGAGGGAGTGGAAGAAGGGAGGCAGTGGTGGCAGTGCAGCAACACTGTGCTAGAGGGGAGAAAGCCCACACCCGGCAGCCAGAGGTCCCGTGTCCCTGCTCTGTCTCCACCTTTCCCCTT
ST-E00192:51:H2KG3CCXX:1:1103:21888:69960- CAAGCAGAGATGAGCCTGAAAGGCTGAGAGGGAGTGGAAGAAGGGAGGCAGTGGTGGCAGTGCAGCAACACTGTGCTAGAGGGGAGAAAGCCCACACCCGGCAGCCAGAGGTCCCGTGTCCCTGCTCTGTCTCCACCTTTCCCCTTTGTG
ST-E00192:51:H2KG3CCXX:8:1210:24974:42992- GCAGAGATGAGCCTGAAAGGCTGAGAGGGAGTGGAAGAAGGGAGGCAGTGGTGGCAGTGCAGCAACACTGTGCTAGAGGGGAGAAAGCCCACACCCGGCAGCCAGAGGTCCCGTGTCCCTGCTCTGTCTCCACCTTTCCCCTTTGTGCCA
ST-E00192:51:H2KG3CCXX:2:1122:14601:22388- GaGaTGAGCCTGAAaGGCTGAGAGGGAGTGGAAGAAGGGAGGCAGTGGTGGCAGTGCAGCAACACTGTGCTAGAGGGGAGAAAGCCCACACCCGGCAGCCAGAGGTCCCGTGTCCCTGCTCTGTCTCCACCTTTCCCCTTTGTGCCACA
ST-E00192:51:H2KG3CCXX:1:1115:7760:49391 - CCTGAAAGGCTGAGAGGGAGTGGAAGAAGGGAGGCAGTGGTGGCAGTGCAGCAACACTGTGCTAGAGGGGAGAAAGCCCACACCCGGCAGCCAGAGGTCCCGTGTCCCTGCTCTGTCTCCACCTTTCCCCTTTGTGCCACAGTGAGCAAA
ST-E00192:51:H2KG3CCXX:5:1114:31835:16586- ccTGAAAGGctgAGAGGGAGtGGAAGAAGGGAGGCaGtGGTGGCAGTGCAGCAACACTGTGCTAGAGGGGAGAAAGCCCACACCCGGCAGCCAGAGGTCCCGTGTCCCTGCTCTGTCTCCACCTTTCCCCTTTGTGCCACAGTGAGCAAA
ST-E00192:51:H2KG3CCXX:1:1115:9993:40987 - GGAGTGGAAGAAGGGAGGCAGTGGTGGCAGTGCAGCAACACTGTGCTAGAGGGGAGAAAGCCCACACCCGGCAGCCAGAGGTCCCGTGTCCCTGCTCTGTCTCCACCTTTCCCCTTTGTGCCACAGTGAGCAAACCGCATGCCCTCCCTG
ST-E00192:51:H2KG3CCXX:5:2116:31054:68641- GAGTGGAAGAAGGGAGGCAGTGGTGGCAGTGCAGCAACACTGTGCTAGAGGGGAGAAAGCCCACACCCGGCAGCCAGAGGTCCCGTGTCCCTGCTCTGTCTCCACCTTTCCCCTTTGTGCCACAGTGAGCAAACCGCATGCCCTCCCTGA
ST-E00192:51:H2KG3CCXX:6:2207:31399:6723 - AGTGGAAGAAGGGAGGCAGTGGTGGCAGTGCAGCAACACTGTGCTAGAGGGGAGAAAGCCCACACCCGGCAGCCAGAGGTCCCGTGTCCCTGCTCTGTCTCCACCTTTCCCCTTTGTGCCACAGTGAGCAAACCGCATGCCCTCCCTGAG
ST-E00192:51:H2KG3CCXX:8:2114:30790:49690- CAGtGGgGGCAGTGCAGCAACACTGTGCTAGAGGGGAGAAAGCCCACACCCGGCAGCCAGAGGTCCCGTGTCCCTGCTCTGTCTCCACCTTTCCCCTTTGTGCCACAGTGAGCAAACCGCATGCCCTCCCTGAGCGACAGCTTTTTCATA
ST-E00192:51:H2KG3CCXX:2:2124:21036:48266- GGCAGTGCAGCAACACTGTGCTAGAGGGGAGAAAGCCCACACCCGGCAGCCAGAGGTCCCGTGTCCCTGCTCTGTCTCCACCTTTCCCCTTTGTGCCACAGTGAGCAAACCGCATGCCCTCCCTGAGCGACAGCTTTTTCATATGGAAAG
ST-E00192:51:H2KG3CCXX:5:1107:17148:48776- AGTGCAGCAACACTGTGCTAGAGGGGAGAAAGCCCACACCCGGCAGCCAGAGGTCCCGTGTCCCTGCTCTGTCTCCACCTTTCCCCTTTGTGCCACAGTGAGCAAACCGCATGCCCTCCCTGAGCGACAGCTTTTTCATATGGAAAGCAG
ST-E00192:51:H2KG3CCXX:5:1108:4116:24163 - AGCAACACTGTGCTAGAGgGGAGAaAGCCCACACCCGGCAGCCAGAGGTCCCGTGTCCCTGCTCTGTCTCCACCTTTCCCCTTTGTGCCACAGTGAGCAAACCGCATGCCCTCCCTGAGCGACAGCTTTTTCATATGGAAAGCAGGAAAG
ST-E00192:51:H2KG3CCXX:1:1216:23309:71331- ACACTGTGCTAGAGGGGAGAAAGCCCACACCCGGCAGCCAGAGGTCCCGTGTCCCTGCTCTGTCTCCACCTTTCCCCTTTGTGCCACAGTGAGCAAACCGCATGCCCTCCCTGAGCGACAGCTTTTTCATATGGAAAGCAGGAAAGACAA
ST-E00192:51:H2KG3CCXX:1:2106:16133:73036- ACACTGTGCTAGAGGGGAGAAAGCCCACACCCGGCAGCCAGAGGTCCCGTGTCCCTGCTCTGTCTCCACCTTTCCCCTTTGTGCCACAGTGAGCAAACCGCATGCCCTCCCTGAGCGACAGCTTTTTCATATGGAAAGCAGGAAAGACAA
ST-E00192:51:H2KG3CCXX:3:2123:7354:54876 - ACACTGTGCTAGAGGGGAGAAAGCCCACACCCGGCAGCCAGAGGTCCCGTGTCCCTGCTCTGTCTCCACCTTTCCCCTTTGTGCCACAGTGAGCAAACCGCATGCCCTCCCTGAGCGACAGCTTTTTCATATGGAAAGCAGGAAAGACAA
ST-E00192:51:H2KG3CCXX:3:2123:7425:55069 - ACACTGTGCTAGAGGGGAGAAAGCCCACACCCGGCAGCCAGAGGTCCCGTGTCCCTGCTCTGTCTCCACCTTTCCCCTTTGTGCCACAGTGAGCAAACCGCATGCCCTCCCTGAGCGACAGCTTTTTCATATGGAAAGCAGGAAAGACAA
ST-E00192:51:H2KG3CCXX:2:1209:24812:72034- TgTGCTAgAggggAgaacgcCcAcacCcggcAgCaAgAgggCCcGtGTCCCTgtgCtGTCTCCaCCttTCCCCtttGtGCcAcaGTgaGCAaaCCGCAtGcCCTCCCTGAGCaACtgCTtTttCaTAgGGAaAGCAGGAAAGACAaCAaG
ST-E00192:51:H2KG3CCXX:2:2105:29694:30281- GCcCACACCCgGCAGCcAGAGGTCCcGTGTCCCTGCTCTGTCTCCACCTTTCCCCTTTGTGCCACAGTGAGCAAACCGCATGCCCTCCCTGAGCGACAGCTTTTTCATATGGAAAGCAGGAAAGACAACAAGACCCACCGGGGTGTGATG
ST-E00192:51:H2KG3CCXX:2:2105:29633:30562- GCCCACACCCGGCAGCCAGAGGTCCCGTGTCCCTGCTCTGTCTCCACCTTTCCCCTTTGTGCCACAGTGAGCAAACCGCATGCCCTCCCTGAGCGACAGCTTTTTCATATGGAAAGCAGGAAAGACAACAAGACCCACCGGGGTGTGATG
```

---

chr1 164023466 + 6 chr12 88622429 - 0 CTX 33 52 67 0 0.82 0 0.989462365591398 0 1 chr1 164023376 150 chr12 88622371

AACATTGAAGGTAGTGCTGAAAGGAGAGGAGTGAGTTTTCTGAACATTTTGGATCCCAGAAAAGATGCTGTAACAGTTTCCATGACCAAATGAGGTGGTGCTGGATGGGAAGATGGCAGCTGGGAAGGTGGTGGCTGAGAAGATGACTGC

```
164023320 164023340 164023360 164023380 164023400 164023420 164023440 164023460 164023480 164023500 164023520 164023540 164023560 164023580 164023600

chr1:164023319-164023615 CAATTAGCTAAGGAAGTAGAAAAAGAGTGGAAATTTACAATGAAAGGAGGTCAGTATAACATTGAAGGTAGTGCTGAAAGGAGAGGAGTGAGTTTTCTGAACATTTTGGATCCCAGAAAAGATGCTGTAACAGTTTCCATGACCAAATATATAAATGGAATAAATTTATCAATCTGAGTATCAAGAATTCAAAAAGGAAGGCACTCAAGGCTCTCAGGGACCAAGGCCTGTCTATTGTGACTGCTAATCCAAGTGAGTTTGGGTTAAAAGGGGTTAACAGCCCTCTGCTGTAACACA

ST-E00144:68:H2LCKCCXX:7:1102:13332:34500+ CAATTAGCTAAGGAAGTAGAAAAAGAGTGGAAATTTACAATGAAAGGAGGTCAGTATAACATTGAAGGTAGTGCTGAAAGGAGAGGAGTGAGTTTTCTGAACATTTTGGATCCCAGAAAAGATGCTGTAACAGTTTCCATGACCAAATAT
ST-E00144:68:H2LCKCCXX:4:2120:14337:23899+ AGCTAAGGAAGTAGAAAAAGAGTGGAAATTTACAATGAAAGGAGGTCAGTATAACATTGAAGGTAGTGCTGAAAGGAGAGGAGTGAGTTTTCTGAACATTTTGGATCCCAGAAAAGATGCTGTAACAGTTTCCATGACCAAATATATAAA
ST-E00144:68:H2LCKCCXX:1:2205:32038:34588+ GCTAAGGAAGTAGAAAAAGAGTGGAAATTTACAATGAAAGGAGGTCAGTATAACATTGAAGGTAGTGCTGAAAGGAGAGGAGTGAGTTTTCTGAACATTTTGGATCCCAGAAAAGATGCTGTAACAGTTTCCATGACCAAATATAtAAAT
ST-E00144:68:H2LCKCCXX:7:1222:31673:22774+ AGTAGAAAAAGAGTGGAAATTTACAATGAAAGGAGGtCAGTATAACATTgAAGGTAGTGCTGAAAGGAGAGGAGtGAGTTTTCTGAACATTTTGGATCccaGAAAAGATGCTGTAACAgTTTCCATGAcgaAAtATAaAAAgGgAAaAAA
ST-E00144:68:H2LCKCCXX:8:2211:9262:21614 + AAAGAGTGGAAATTTACAATGAAAGGAGGTCAGTATAACATTGAAGGTAGTGCTGAAAGGAGAGGAGTGAGTTTTCTGAACATTTTGGATCCCAGAAAAGATGCTGTAACAGTTTCCATGACCAAATATATAAATGGAATAAATTTATCA
ST-E00144:68:H2LCKCCXX:1:2210:23198:28382+ AGAGTGGAAATTTACAATGAAAGGAGGTCAGTATAACATTGAAGGTAGTGCTGAAAGGAGAGGAGTGAGTTTTCTGAACATTTTGGATCCCAGAAAAGATGCTGTAACAGTTTCCATGACCAAATATATAAATGGAATAAATTTATCAAT
ST-E00144:68:H2LCKCCXX:7:1224:11891:3207 + AATTTACAATGAAAGGAGGTCAGTATAACATTGAAGGTAGTGCTGAAAGGAGAGGAGTGAGTTTTCTGAACATTTTGGATCCCAGAAAAGATGCTGTAACAGTTTCCATGACCAAATATATAAATGGAATAAATTTATCAATCTGAGTAT
ST-E00144:68:H2LCKCCXX:7:1102:28801:32531+ AAAGGAGGTCAGTATAACATTGAAGGTAGTGCTGAAAGGAGAGGAGTGAGTTTTCTGAACATTTTGGATCCCAGAAAAGATGCTGTAACAGTTTCCATGACCAAATATATAAATGGAATAAATTTATCAATCTGAGTATCAAGAATTCAA
ST-E00144:68:H2LCKCCXX:8:1112:27836:24673+ GTATAACATTGAAGGTAGTGCTGAAAGGAGAGGAGTGAGTTTTCTGAACATTTTGGATCCCAGAAAAGATGCTGTAACAGTTTCCATGACCAAATATATAAATGGAATAAATTTATCAATCTGAGTATCAAGAATTCAAAAAGGAAGGCA
ST-E00144:68:H2LCKCCXX:3:1109:24111:47299+ AACATTGAAGGTAGTGCTGAAAGGAGAGGAGTGAGTTTTCTGAACATTTTGGATCCCAGAAAAGATGCTGTAACAGTTTCCATGACCAAATGAGGTGGTGCTGGATGGGAAGATGGCAGcTGGGAAGGTGGtGGcTGAGAAGaTGACtGC
ST-E00144:68:H2LCKCCXX:3:1109:23827:47791+ AACATTGAAGGTAGTGCTGAAAGGAGAGGAGTGAGTTTTCTGAACATTTTGGATCCCAGAAAAGATGCTGTAACAGTTTCCATGACCAAATGAGGTGGTGCTGGATGGGAAGATGGCAGCTGGGAAGGTGGTGGCTGAGAAGATGACTGC
ST-E00144:68:H2LCKCCXX:3:2224:16357:42376+ AACATTGAAGGTAGTGCTGAAAGGAGAGGAGTGAGTTTTCTGAACATTTTGGATCCCAGAAAAGATGCTGTAACAGTTTCCATGACCAAATGAGGTGGTGCTGGATGGGAAGATGGCAGCTGGGAAGGTGGTGGCTGAGAAGATGACTGC
ST-E00144:68:H2LCKCCXX:6:1117:2553:64492 + GAAGGTAGTGCTGAAAGGAGAGGAGTGAGTTTTCTGAACATTTTGGATCCCAGAAAAGAtGCTGgAACAGTTTCCATGACCAAATATATAAATGGAATAAATTTATCAATCTGAGTATCAAGAATtCAAAAAGGAAGGCACTCAAGGCTC
ST-E00144:68:H2LCKCCXX:1:2219:21411:39563+ AGAGGAGTGAGTTTTCTGAACATTTTGGATCCCAGAAAAGATGCTGTAACAGTTTCCATGACCAAATATATAAATGGAATAAATTTATCAATCTGAGTATCAAgAATTCAAAAAGGAAGGCACTCAAGGCTCTCAGGGACCAAGGCcTGt
ST-E00144:68:H2LCKCCXX:7:2111:17615:26167+ TTTCTGAACATTTTGGATCCCAGAAAAGATGCTGTAACAGTTTCCATGACCAAATATATAAATGGAATAAATTTATCAATCTGAGTATCAAGAATTCAAAAAGGAAGGCACTCAAGGCTCTCAGGgACCAAGGCCTGTCTaTTGTGACTG
ST-E00144:68:H2LCKCCXX:1:1110:21716:26062+ TTTCCATGACCAAATATATAAATGGAATAAATTTATCAATCTGAGTATCAAGAATTCAAAAAGGAAGGCACTCAAGGCTCTCAGGGACCAAGGCCTGTCTATTGTGACTGCTAATCCAAGTGAGTTTGGGTTAAAAGGGGTTAACAGCCC
ST-E00144:68:H2LCKCCXX:4:1214:13109:67200+ TCCATGACCAAATATATAAATGGAATAAATTTATCAATCTGAGTATCAAGAATTCAAAAAGGAAGGCACTCAAGGCTCTCAGGGACCAAGGCCTGTCTATTGTGACTGCTAATCCAAGTGAGTTTGgGTTAAAAGGgGTTAACAGCCCTC
ST-E00144:68:H2LCKCCXX:4:1218:9891:45980 + TCCATGACCAAAgATATAAATGGAATAAATTTATCAATCTGAGTATCAAGAATTCAAAAAGGAAGGCACTCAAGGCTCTCAGGGACcAAGGCcTGTCTATTGtGACTGCTAATCCAAGTGAGtttGGgtgAAAAGGGGTTAACAGCCCTC
ST-E00144:68:H2LCKCCXX:1:2123:4390:18766 + CCATGACCAAATATATAAATGGAATAAATTTATCAATCTGAGTATCAAGAATTcAAAAAGGAAGGCACTCAAGGCaCTCAGGGacCAAGGCCTGtCtATtGTGACTGCTAATCCAAGTGAGtTtggGTTAAAAGGtGTGAACAGCcCTCT
ST-E00144:68:H2LCKCCXX:8:2221:13139:55755+ ACCAAATATATAAATGGAATAAATTTATCAATCTGAGTATCAAGAATTCAAAAAGGAAGGCACTCAAGGCTCTCAGGGACCAAGGCCTGTCTATTGTGACTGCTAATCCAAGTGAGTTTGGGTTAAAAGGGGTTAACAGCCCTCTGCTGT
ST-E00144:68:H2LCKCCXX:7:2114:26973:8200 + CCAAATATATAAATGGAATAAATTTATCAATCTGAGTATCAAGAATTCAAAAAGGAAGGCACTCAAGGCTCTCAGGGACCAAGGCCTGTCTATTGTGACTGCTAATCCAAGTGAGTTTGGGTTAAAAGGGGTTAACAGCCCTCTGCTGTA
ST-E00144:68:H2LCKCCXX:1:1112:19889:33164+ AAATATATAAATGGAATAAATTTATCAATCTGAGTATCAAGAATTCAAAAAGGAAGGCACTCAAGGCTCTCAGGGACCAAGGCCTGTCTATTGTGACTGCTAATCCAAGTGAGTTTGGGTTAAAAGGGGTTAACAGCCCTCTGCTGTAAC
ST-E00144:68:H2LCKCCXX:8:2218:18417:35081+ AATATATAAATGGAATAAATTTATCAATCTGAGTATCAaGAATTCAAAAAGGAAGGCACTCAAGGCTCTCAGGGACCAAGGCCTGTCTATTGTGACTGCTAATCCAAGTGAGTTTGGGTTAAAAGGGGTTAACAGCCCTCTGCTGTAACA
ST-E00144:68:H2LCKCCXX:3:1122:32800:36856- CAGTATAaCagtGAAGGtAgtGcgGAAAGGAGAGGAGtgAGTTTtCTGAACATTTtGGATCCCaGAAAAGATGcTGTAACAGTTTCgATGAcCAAAaATATAAAtGGAATAaATTTATCAAtCTGAGTATCAAGAATtCAAAAAGGAAG
ST-E00144:68:H2LCKCCXX:3:1109:24111:47299- AGGTAGTGCTGAAAGGAGAGGAGTGAGTTTTCTGAACATTTTgGATCCCAgAAAAGATGCTGTAACAGTTTCCATGACCAAATGAGGTGGTGCTGGATGGGAAGATGGCAGCTGGGAAGGTGGTGGCTGAGAAGATGACTGCTGGGAAGA
ST-E00144:68:H2LCKCCXX:3:1109:23827:47791- AGGTAGTGCTGAAAGGAGAGGAGTGAGTTTTCTGAACATTTTGGATCCCAGAAAAGATGCTGTAACAGTTTCCATGACCAAATGAGGTGGTGCTGGATGGGAAGATGGCAGCTGGGAAGGTGGTGGCTGAGAAGATGACTGCTGGGAAGA
ST-E00144:68:H2LCKCCXX:3:2224:16357:42376- AGGTAGTGCTGATAGGAGAGGAGTGAGTTTTCTGAACATTTTGGATCCCAGAAAAGATGCTGTAACAGTTTCCATGACCAAATGAGGTGGTGCTGGATGGGAAGATGGCAGCTGGGAAGGTGGTGGCTGAGAAGATGACTGCTGGGAAGA
ST-E00144:68:H2LCKCCXX:1:1216:5344:45840 - GTAGTGCTgAAagGAgAGGAGtGAGTTTtCTGAACATTTTGGATCCCAGAAAAGATgcTGTAACAGTTTCCATGACCAAATATATAaAtgGAATAAATTTATCAATCTGAGTATCAAGAAtTCAAAAAGGAAGGCACTCAAGGCTCTCAG
ST-E00144:68:H2LCKCCXX:6:2113:12408:22001- TGCTGAAAGGAGAGGAGTGAGTTTTCTGAACaTTTTGGATCCCAGAAAAGATGCTGTAACAGTTTcCATgAcCAAATATATAAATGGAATAAATTTATCAATCTGAGTATCAAGAATTCAAAAAGGAAGGCACTCAAGGCTCTCAGGGAC
ST-E00144:68:H2LCKCCXX:3:2123:22183:12841- AGTGAgtTgTCTGAACaTTTTGGATCCCAGAAATGATGCTgTAACAGTTTCcATGACCAAATATATAaATGGAATAAatgTATCAATCTGAGTATCAAGAATTCAAAAAGGAAGGCACTCAAGGCTCTCAGGGACCAAGGCCTGTCTATT
ST-E00144:68:H2LCKCCXX:7:1102:13332:34500- TTGGATCCCAGAAAAGATGCTGTAACAGTTTCCATGACCAAATATATAAATGGAATAcATTTATCAATCTGAGTATCAAGAATTCAAAAAGGAAGGCACTCAAGGCTCTCAGGGACCAAGGCCTGTCTATTGTGACTGCTAATCCAAGTG
ST-E00144:68:H2LCKCCXX:4:1212:16215:23214- cCAGAaAAGATGCTGTAACAGTTTccatcaCCaaatAtatAaaTGGAatAAaTTTATCaAtCTGAgtATCAAGaattCAAAAAGGAAGGcACTCAAGGCTCtCAGGGACCAAGGCCTGTCTATTGTGACTGCtaAtCCAAGTGAGTTTgg
ST-E00144:68:H2LCKCCXX:1:1107:14357:57443- GAAAAGATGCTGTAACAGTTTCCATGACCAAATATATAAATGGAATAAATTTATCAATCTGAGTATCAAGAATTCAAAAAGGAAGGCACTCAAGGCTCTCAGGGACCAAGGCCTGTCTATTGTGACTGCTAATCCAAGTGAGTTTGGGTT
ST-E00144:68:H2LCKCCXX:7:2111:17615:26167- ATGCTGTAACAGTTTCCATGACCAAATATATAAATGGAATAAATTTATCAATCTGAGTATCAAGAATTCAAAAAGGAAGGCACTCAAGGCTCTCAGGGACCAAGGCCTGTCTATTGTGACTGCTAATCCAAGTGAGTTTGGGTTAAAAGG
ST-E00144:68:H2LCKCCXX:4:2221:22193:50622- CTGTAACAGTTTCCATGACCAAATATATAAATGGAATAAATTTATCAATCTGAGTATCAAGAATTCAAAAAGGAAGGCACTCAAGGCTCTCAGGGACCAAGGCCTGTCTATTGTGACTGCTAATCCAAGTGAGTTTGGGTTAAAAGGGGT
ST-E00144:68:H2LCKCCXX:7:1221:17717:39142- ACAGTTTCCATGACCAAATATATaaATGGAATAAATTTATCAATCTGAGTATCAAGAATTCAAAAAGGAAGGCACTCAAGGCTCtCAGGGACCAAGGCCTGTCTATTGtGACTGCTAATCCAAGTGAGTTTGGGTTAAAAGGGGTTAACA
ST-E00144:68:H2LCKCCXX:7:1208:25116:31072- CAtGACCAAATATATAAAtggAATAAATTTATCAATCTGAGTATCAAGAATTCAAAAAGGAAGGCACTCAAGGCTCTCAGGGACCAAGGCCTGTCTATTGTGACTGCTAATCCAAGTGAGTTTGGGTTAAAAGGGGTTAACAGCCCTCTG
```

```
ST-E00192:51:H2KG3CCXX:2:2221:26019:54472+ AGAAAAAGAGTGGAAAtTTACAATGAAAGGAGGTCAGtATAACATTGAAGGTAGTGCTGAAAGGaGaGGAGggAGTTTTCTGAACATTTTGGATCCCAGAAAAGATGCTGTAACAGtTTCCATGACCAAATATATAAaTGGAATAAATTT
ST-E00192:51:H2KG3CCXX:2:2221:26588:54753+ AGAAAAAGAGTGGAAATTTACAATGAAAGGAGGTCAGTATAACATTGAAGGTAGTGCTGAAAGGAGAGGAGTGAGTTTTCTGAACATTTTGGATCCCAGAAAAGATGCTGTAACAGTTTCCATGACCAAATATATAAATGGAATAAATTT
ST-E00192:51:H2KG3CCXX:2:2119:31033:3243 + AAAGAGTGGAAATTTACAATGAAAGGAGGTCAGTATAACATTGAAGGTAGTGCTGAAAGGAGAGGAGTGAGTTTTCTGAACATTTTGGATCCCAGAAAAGATGCTGTAACAGTTTCCATGACCAAATATATAAATGGAATAAATTTATCA
ST-E00192:51:H2KG3CCXX:3:1212:31419:14810+ AACATTGAAGGTAGTGCTGAAAGGAGAGGAGTGAGTTTTCTGAACATTTTGGATCCCAGAAAAGATGCTGTAACAGTTTCCATGACCAAATATATAAATGGAATAAATTTATCAATCTGAGTATCAAGAATTCAAAAAGGAAGGCACTCA
ST-E00192:51:H2KG3CCXX:2:2119:12804:70083+ CATTGAAGGTAGTGCTGAAAGGAGAGGAGTGAGTTTTCTGAACATTTTGGATCCCAGAAAAGATGCTGTAACAGTTTCCATGACCAAATATATAAATGGAATAAATTTATCAATCTGAGTATCAAGAATTCAAAAAGGAAGGCACTCAG
ST-E00192:51:H2KG3CCXX:1:1206:24974:8077 + ATTGAAGGTAGTGCTGAAAGGAGAGGAGTGAGTTTTCTGAACATTTTGGATCCCAGAAAAGATGCTGTAACAGTTTCCATGACCAAATATATAAATGGAATAAATTTATCAATCTGAGTATCAAGAATTCAAAAAGGAAGGCACTCAAGG
ST-E00192:51:H2KG3CCXX:3:1113:26659:12859+ GAAAGGAGAGGAGTGAGTTTTCTGAACATTTTGGATCCCAGAAAAGATGCTGTAACAGTTTCCATGACCAAATATATAAATGGAATAAATTTATCAATCTGAGTATCAAGAATTCAAAAAGGAAGGCACTCAAGGCTCTCAGGGACCAAG
ST-E00192:51:H2KG3CCXX:8:2120:29816:49795+ AGTGAGTTTTCTGAACATTTTGGATCCCAGAAAAGATGCTGTAACAGTTTCCATGACCAAATATATAAATGGAATAAATTTATCAATCTGAGTATCAAGAATTCAAAAAGGAAGGCACTCAAGGCTCTCAGGGACCAAGGCCTGTCTATT
ST-E00192:51:H2KG3CCXX:3:1212:3760:71226 + GTGAGTTTTCTGAACATTTTGGATCCCAGAAAAGATGCTGTAACAGTTTCCATGACCAAATATATAAATGGAATAAATTTATCAATCTGAGTATCAAGAATTCAAAAAGGAAGGCACTCAAGGCTCTCAGGGACCAAGGCCTGTCTATTG
ST-E00192:51:H2KG3CCXX:3:1212:3781:71226 + GTGAGTTTTCTGAACATTTTGGATCCCAGAAAAGATGCTGTAACAGTTTCCATGACCAAATATATAAATGGAATAAATTTATCAATCTGAGTATCAAGAATTCAAAAAGGAAGGCACTCAAGGCTCTCAGGGACCAAGGCCTGTCTATTG
ST-E00192:51:H2KG3CCXX:5:1215:25674:52573+ GAGTTTTCTGAACATTTTGGATCCCAGAAAAGATGCTGTAACAGTTTCCATGACCAAATATATAAATGGAATAAATTTATCAATCTGAGTATCAAGAATTCAAAAAGGAAGGCACTCAAGGCTCTCAGGGACCAAGGCCTGTCTATTGTG
ST-E00192:51:H2KG3CCXX:5:1215:25715:52643+ GAGTTTTCTGAACATTTTGGATCCCAGAAAAGATGCTGTAACAGTTTCCATGACCAAATATATAAATGGAATAAATTTATCAATCTGAGTATCAAGAATTCAAAAAGGAAGGCACTCAAGGCTCTCAGGGACCAAGGCCTGTCTATTGTG
ST-E00192:51:H2KG3CCXX:5:1215:25715:52714+ GaGTTtTCTGAACATTTTGgATCCCAGAAAAGATGCTGTAACAGTTTCCATGACCAAATATATAAATGGAATAAATTTATCAATCTGAGTATCAAGAATTcAAAAAGGAAGGCACTCAAGGCTCTCAGGGACCAAGGCCTGgctATgGtG
ST-E00192:51:H2KG3CCXX:1:2209:17808:38526+ AGTTTTCTGAACATTTTGGATCCCAGAAAAGATGCTGTAACAGTTTCCATGACCAAATATATAAATGGAATAAATTTATCAATCTGAGTATCAAGAATTCAAAAAGGAAGGCACTCAAGGCTCTCAGGGACCAAGGCCTGTCTATTGTGA
ST-E00192:51:H2KG3CCXX:8:2107:26933:3735 + GAACATTTTGGATCCCAGAAAAGATGCTGTAACAGTTTCCATGACCAAATATATAAATGGAATAAATTTATCAATCTGAGTATCAAGAATTCAAAAAGGAAGGCACTCAAGGCTCTCAGGGACCAAGGCCTGTCTATTGTGACTGCTAAT
ST-E00192:51:H2KG3CCXX:6:2219:13890:42077+ TTTGGATCCCAGAAAAGATGCTGTAACAGTTTCCATGACCAAATATATAAATGGAATAAATTTATCAATCTGAGTATCAAGAATTCAAAAAGGAAGGCACTCAAGGCTCTCAGGGACCAAGGCCTGTCTATTGTGACTGCTAATCCAAG
ST-E00192:51:H2KG3CCXX:6:2122:3040:63051 + AAGATGCTGTAACAGTTTCCATGACCAAATATATAAATGGAATAAATTTATCAATCTGAGTATCAAGAATTgAaAAAGGAAGGCACTCAAGGCTCTCAGGGACCAAGGCCTGTCTATTGTGACTGCTAATCCAAGTGAGTTTGGGTTAAA
ST-E00192:51:H2KG3CCXX:3:2102:2339:50586 + GATGCTGTAACAGTTTCCATGACCAAATATATAAATGGAATAAATTTATCAATCTGAGTATCAAGAATTCAAAAAGGAAGGCACTCAAGGCTCTCAGGGACCAAGGCCTGTCTATTGTGACTGCTAATCCAAGTGAGtTTGGGTTAAAAG
ST-E00192:51:H2KG3CCXX:8:1203:27735:40460+ TAACAGTTTCCATGACCAAATATATAAATGGAATAAATTTATCAATCTGAGTATCAAGAATTCAAAAAGGAAGGCACTCAAGGCTCTCAGGGACCAAGGCCTGTCTATTGTGACTGCTAATCCAAGTGAGTTTGGGTTAAAAGGGGTTAA
ST-E00192:51:H2KG3CCXX:5:1120:4288:56880 + TTTCCATGACCAAATATATAAATGGAATAAATTTATCAATCTGAGTATCAAGAATTCAAAAAGGAAGGCACTCAAGGCTCTCAGGGACCAAGGCCTGTCTATTGTGACTGCTAATCCAAGTGAGTTTGGGTTAAaAGGGGTTAACAGCCC
ST-E00192:51:H2KG3CCXX:2:1207:19331:11206+ ACCAAATATATAAATGGAATAAATTTATCAATCTGAGTATCAAGAATTCAAAAAGGAAGGCACTCAAGGCTCTCAGGGACCAAGGCCTGTCTATTGTGACTGCTAATCCAAGTGAGTTTGGGTTAAAAGGGGTTAACAGCCCTCTGCTGT
ST-E00192:51:H2KG3CCXX:5:2118:10348:5915 + ACCAAATATATAAATGGAATAAATTTATCAATCTGAGTATCAAGAATTCAAAAAGGAAGGCACTCAAGGCTCTCAGGGACCAAGGCCTGTCTATTGTGACTGCTAATCCAAGTGAGTTTGGGTTAAAAGGGGTTAACAGCCCTCTGCTGT
ST-E00192:51:H2KG3CCXX:8:1118:29623:26783- CAATTAGCTAAGGAAGTAGAAAAAGAGTGGAAATTTACAATGAAAGGAGGTCAGTATAACATTGAAGGTAGTGCTGAAAGGAGAGGAGTGAGTTTTCTGAACgTTTGGATCCCAGAAAAGATGCTGTAACAGTTTCCATGACCAAATAT
ST-E00192:51:H2KG3CCXX:8:2121:20873:22423- TAAGGAAgtAGAAAAAGAGTGGAAAtTTACAATGAAAGGAGGTCAGTATAACATTGAAGGTAGTGCTGAAAGGAGAGGAGTGAGTTTTCTGAACATTTTGGATCCCAGAAAAGATGCTGTAACAGTTTCCATGACCAAATATATAAAT
ST-E00192:51:H2KG3CCXX:2:2103:25908:20770- AGAGTGGAAATTTACAATGAAAGGAGGTCAGTATAACATTGAAGGTAGTGCTGAAAGGAGAGGAGTGAGTTTTCTGAACATTTTGGATCCCAGAAAAGATGCTGTAACAGTTTCCATGACCAAATATATAAATGGAATAAATTTATCAAT
ST-E00192:51:H2KG3CCXX:1:1119:25268:48318- TACAATGAAAGGAGGTCAGTATAACATTGAAGGTAGTGCTGAAAGGAGAGGAGTGAGTTTTCTGAACATTTTGGATCCCAGAAAAGATGCTGTAACAGTTTCCATGACCAAATATATAAATGGAATAAATTTATCAATCTGAGTATCAAG
ST-E00192:51:H2KG3CCXX:3:2123:14733:19416- CAATGAAAGGAGGTCAGTATAACATTGAAGGTAGTGCTGAAAGGAGAGGAGTGAGTTTTCTGAACATTTTGGATCCCAGAAAAGATGCTGTAACAGTTTCCATGACCAAATATATAAATGGAATAAATTTATCAATCTGAGTATCAAGAA
ST-E00192:51:H2KG3CCXX:1:2204:21015:72527- TCAGTATAACATTGAAGGTAGTGCTGAAAGGAGAGGAGTGAGTTTTCTGAACATTTTGGATCCCAGAAAAGATGCTGTAACAGTTTCCATGACCAAATATATAAATGGAATAAATTTATCAATCTGAGTATCAAGAATTCAAAAAGGAAG
ST-E00192:51:H2KG3CCXX:3:1106:24781:72509- GAAGGTAGTGCTGAAAGGAGAGGAGTGAGTTTTCTGAACATTTTGGATCCCAGAAAAGATGCTGTAACAGTTTCCATGACCAAATATATAAATGGAATAAATTTATCAATCTGAGTATCAAGAATTCAAAAAGGAAGGCACTCAAGGCTC
ST-E00192:51:H2KG3CCXX:8:2223:2857:25921 - GAGtgtGTTTTCTGAAcATTTTgGATCCCAGAAAAGaTgaTGTAACAGTTTCCATGACCAAATATATaAaTGGAATAAATTTATCaATCTGAGTATCAAGAATTCAAAAAGGAAGGCACTCAAGGCTCTCAGGGACCAAGGCCTGTCTAT
ST-E00192:51:H2KG3CCXX:8:2223:2867:25939 - gAGTGAGTTTTCTGAACATTTTGgATCCCAGAAAAGATGCTGTAACAGTTTCCATGACCAAATATATaAATGGAATAAATTTATCAATCTGAGTATCAAGAATTCAAAAAGGAAGGCACTCAAGGCTCTCAGGGACCAAGGCCTGTCTAT
ST-E00192:51:H2KG3CCXX:8:1106:29907:53118- GAaCATTTTGGATCCCAGAAAAGATGCTGTAACAGTTTCCATGACCAAATATATAAATGGAATAAATTTATCAATCTGAGTATCAAGAATTCAAAAAGGAAGGCACTCAAGGCTCTCAGGGACCAAGGCCTGTCTATTGTGACTGCTAAT
ST-E00192:51:H2KG3CCXX:3:1210:14195:47457- TCCCAGAAAAGATGCTGTAACAGTTTCCATGACCAAATATATAAATGGAATAAATTTATCAATCTGAGTATCAAGAATTCAAAAAGGAAGGCACTCAAGGCTCTCAGGGACCAAGGCCTGTCTATTGTGACTGCTAATCCAAGTGAGTTT
ST-E00192:51:H2KG3CCXX:3:2206:26273:72597- CCAGAAAAGATGCTGTAACAGTTTCCATGAccAAATATATAAATGGAATAAATTTATCAATCTGAGTATCAAGAATTCAAAAAGGAAGGCACTCAAGGCTCTCAGGGACCAAGGCCTGTCTATTGTGACTGCTAATCCAAGTGAGTTTGG
ST-E00192:51:H2KG3CCXX:3:2206:26293:72597- ccAGAAAAGATGcTGTAACAGtttCcATGACcAAATATATAAATggAATAAAttTATCaatcTGAGtATCAAgaagTCAaAaaGgcAgGCACTCAAGGCtCtCaGGGACCAAGGcCtGTCTATTGTGACTGcTAATCCAagTGaGTTtGG
ST-E00192:51:H2KG3CCXX:1:1223:9607:36768 - CAGAAAAGATGCTGTAACAGTTTCCATGACCAAATATATAAATGGAATAAATTTATCAATCTGAGTATCAAGAATTCAAAAAGGAAGGCACTCAAGGCTCTCAGGGACCAAGGCCTGTCTATTGTGACTGCTAATCCAAGTGAGTTTGGG
ST-E00192:51:H2KG3CCXX:2:2119:12804:70083- CAGAAAAGATGCTGTAACAGTTTCCATGACCAAATATATAAATGGAATAAATTTATCAATCTGAGTATCAAGAATTCAAAAAGGAAGGCACTCAAGGCTCTCAGGGACCAAGGCCTGTCTATTGTGACTGCTAATCCAAGTGAGTTTGGG
ST-E00192:51:H2KG3CCXX:2:2208:19016:10450- GTTTCCATGACCAAATATATAAATGGAATAAATTTATCAATCTGAGTATCAAGAATTCAAAAAGGAAGGCACTCAAGGCTCTCAGGGACCAAGGCCTGTCTATTGTGACTGCTAATCCAAGTGAGTTTGGGTTAAAAGGGGTTAACAGCC
ST-E00192:51:H2KG3CCXX:3:1110:11200:40583- TATATAAATGGAATAAATTTATCAATctGAGTAtCAAGAATTCAAAAAGGAAGGCACTCAAGGCTCTCAGGGACCAAGGCCTGTCTATTGTGACTGCTAATCCAAGTGAGTTTGGGTTAAAAGGGGTTAACAGCCCTCTGCTGTAACACA
ST-E00192:51:H2KG3CCXX:3:1110:11271:40636- TATATAAATGGAATAAATTTATCAATCTGAGTATCAAGAATTCAAAAAGGAAGGCACTCAAGGCTCTCAGGGACCAAGGCCTGTCTATTGTGACTGCTAATCCAAGTGAGTTTGGGTTAAAAGGGGTTAACAGCCCTCTGCTGTAACACA
```

```
88622300 88622320 88622340 88622360 88622380 88622400 88622420 88622440 88622460 88622480 88622500 88622520 88622540 88622560

chr12:88622284-88622575 acccttcccagcccctacctctccctctcaaccatcttctttaatccccaagcttctcttttttcttctcagctgccatcttcccagcagtcatcttctcagccaccaccttcccagctgccatcttcccatccagcaccacctccaagagtatccacttcttttcctacactgtcctctcctcaggacaattctagcactgcctgtaccccattcttctccctcattgccctctcctgaggcccataaacccatcccaccaccttaaaccccttatcttattctccactgctt

ST-E00144:68:H2LCKCCXX:7:1107:30942:35678+ CCCAGCCCCTACCTCTCCCTCTCAACCATCTTCTTTAATCCCCAAGCTTCTCTTTTTTCTTCTCAGCTGCCATCTTCCCAGCAGTCATCTTCTCAGCCACCACCTTCCCAGCTGCCATCTTCCCaTCCAGCACCacCTCCAAGAGtATC
```

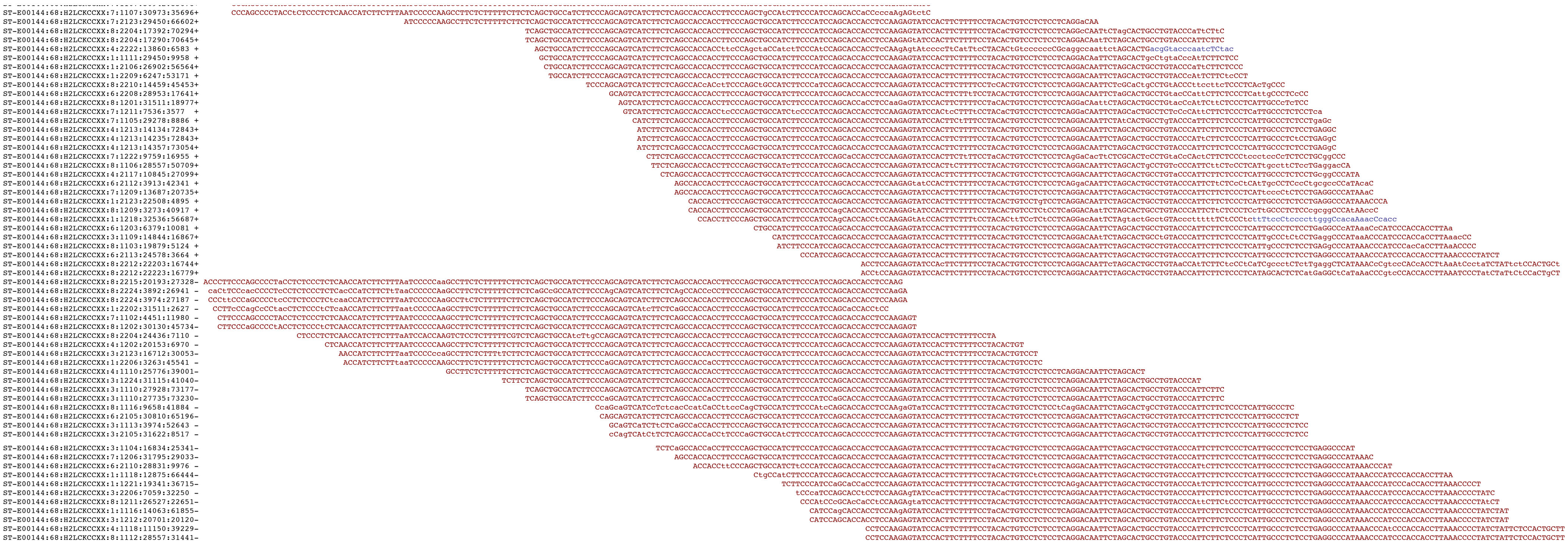

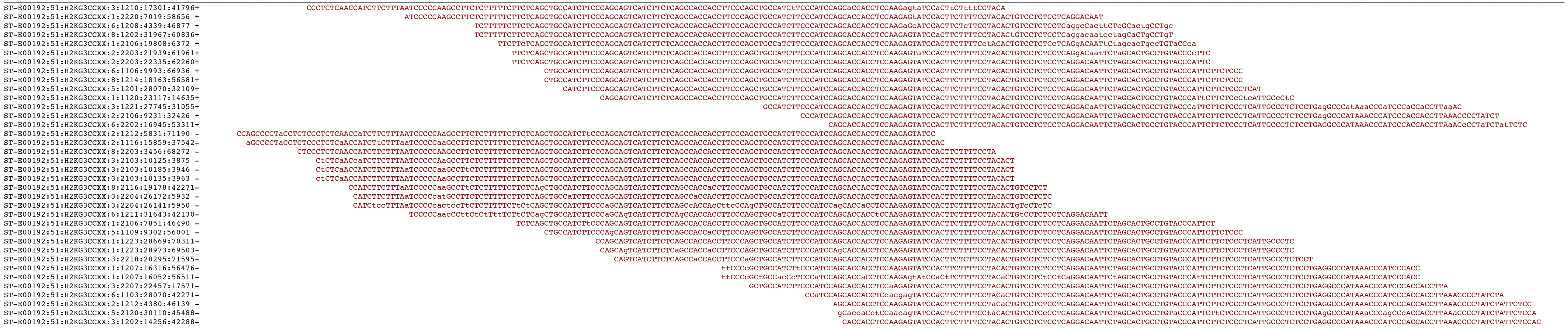

chr6 169957144 + 6 chr6 57373416 + 0 INS 50 72 50 0 0.95 0 1 0 1 chr6 169957018 150 chr6 57373438

TGAGATGATGGCACTGAGACTCTCATACCTTGCTGGGGAGTGTCAAGATGTACAACCCCATGCAAAACTGAGAGGCAGTTACGTTGCGGTTAAGCACACATCGCCCTCAGCAACTGCACTCGTGTCTTAGATCATTTTCTTAGGGTAGGT

169957000 169957020 169957040 169957060 169957080 169957100 169957120 169957140 169957160 169957180 169957200 169957220 169957240 169957260 169957280

chr6:169956998-169957290 ctaaagaaaccacatgtaactgagatgatggcactgagactctcataccttgctggggagtgtcaagatgtacaaccccatgcaaaactgagaggcagttacgttgcggttaagcacacatcgccctcagcaactgcactcgtgtctctcgcaagagaaacgagaacatgtgttcacacaaacagctgtacaagaatttttatggcagcattatttgtaatcaccaaaaactggaaacatcaaaagatccccaactgagggctgggtaaactgtgtttctcccacacaggga

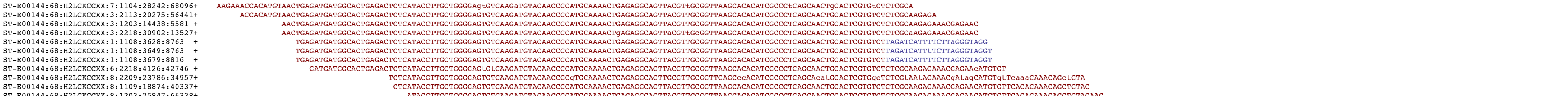

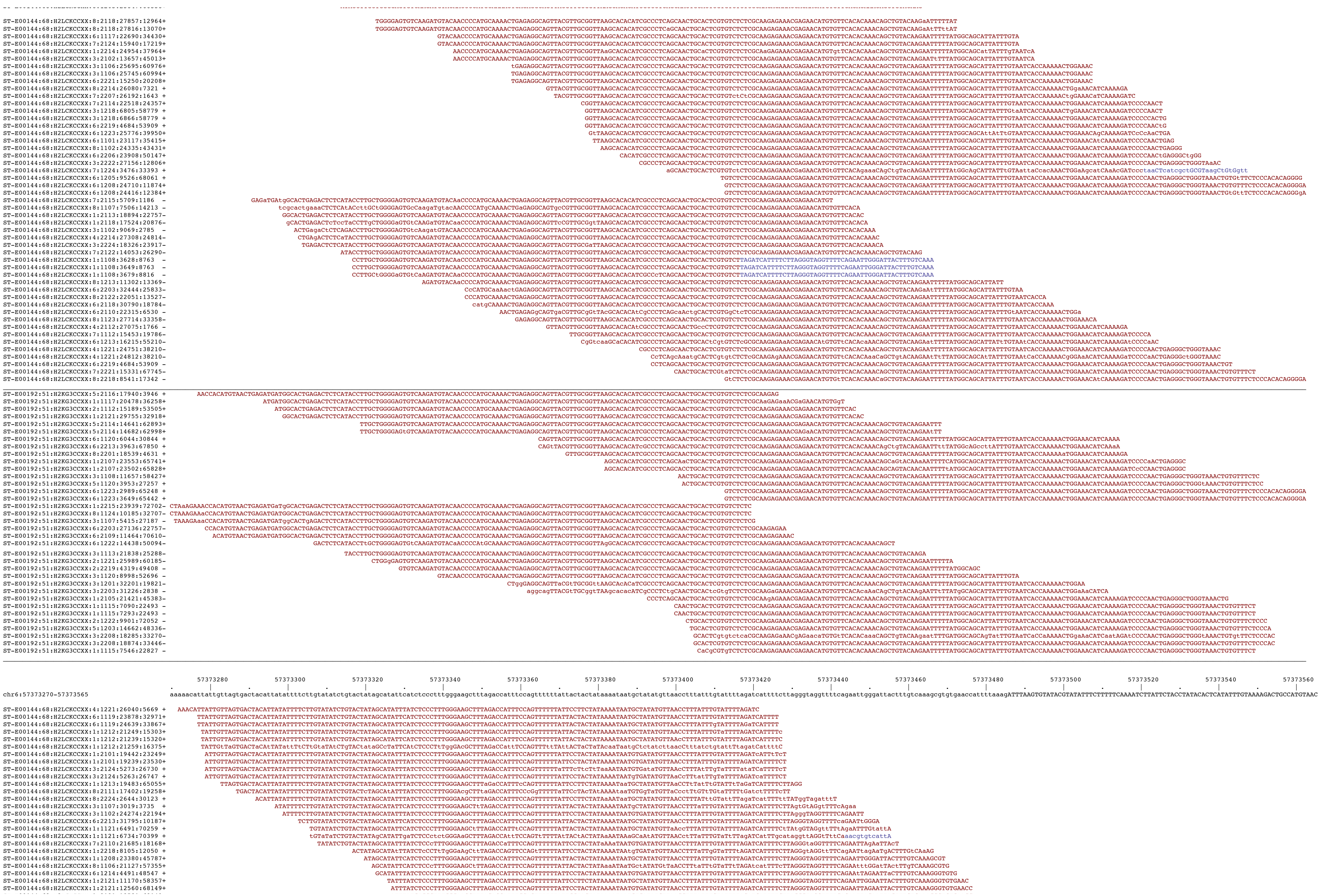
chr6:57373270-57373565
57373280
57373300
57373320
57373340
57373360
57373380
57373400
57373420
57373440
57373460
57373480
57373500
57373520
57373540
57373560

```
ST-E00144:68:H2LCKCCXX:1:2121:12581:68149+ ATTTATCTCCCTTTGGGAAGCTTTAGACCATTTCCAGTTTTTTATTCCTACTATAAAATAATGTGATATGTTAACCTTTATTTGTATTTTAGATCATTTTCTTAGGGTAGGTTTTCAGAATTAGAATTACTTTGTCAAAGGGTGTGAACC
ST-E00144:68:H2LCKCCXX:4:1110:7486:56230 + ATTTATCTCCCTTTGGGAAGCTTTAGACCATTTCCAGTTTTTTATTCCTTCTATAAAATAATGCTATATGTTAACCTTTATTTGTATTTTAGATCATTTTCTTAGGGTAGGTTTTCAGAATTGGAATTACTTTGTCAAAGGGTGTGAACC
ST-E00144:68:H2LCKCCXX:8:1211:18214:11488+ TTTATCTCCCTTTGGGAAGCTTTAGACCATTTCCAGTTTTTTATTCCTACTATAAAATAATGTGATATGTTAACCTTTATTTGTATTTTAGATCATTTTCTTAGGGTAGtTTTTCAGAaTTAGaATTAcTTTgTCAAAGGGTGTgAACCA
ST-E00144:68:H2LCKCCXX:8:1112:6430:4438  + GAAGCTTTAGACCATTTCCAGTTTTTTATTCCTACTATAAAATAATGTGATATGTTAACCTTTATTTGTATTTTAGATCATTTTCTTAGGGTAGGTTTTCAGAATTAGAATTACTTTGTCAAAGGGTGTGAACCATTTCAAAGATTTAAG
ST-E00144:68:H2LCKCCXX:8:2120:9922:26660 + GCTTTAGACCATTTCCAGTTTTTTATTACTACTATAAAATAATGCTATATGTTAACCTTTATTTGTATTTTAGATCATTTTCTTAGGGTAGGTTTTCAGAATTGGGATTACTTTGTCAAAGCGTGTGAACCATTTTaAAGATTTAAGTGT
ST-E00144:68:H2LCKCCXX:8:2113:28780:64528+ CTTTAGACCATTTCCAGTTTTTTATTACTACTATAAAATAATGCTATATGTTAACCTtTATTTGTATTTTAGATCATTTTCTTAGGGtAGGTTTTCAGAATTgGGATTACTTTGTCAAAGCGTGtGAACCATTTTAaAGaTTTAAGTGta
ST-E00144:68:H2LCKCCXX:6:2101:22579:72597+ TAGACCATTTCCAGTTTTTTATTACTACTATAAAATAATGCTATATGTTAAcCTTTATTTGTATTTTAGATCATTTTCTTAGGGTAGGTTTTCAGAATTGGGATTACTTTGTCAAAGCgTGTgAACcATTTTaAaGATTTAAgtgtaTac
ST-E00144:68:H2LCKCCXX:4:2108:12205:38491+ AGACCATTTCCAGTTTTTTATTCCTTCTATAAAATAATGCTATATGTTAACCTTTATTTGTATTTTAGATCATTTTCTTAGGGTAGGTTTTCAGAATTGGAATTACTTTGTCAAAGGGTGTGAACCATTTTAAAGATTTAAGTGTATATG
ST-E00144:68:H2LCKCCXX:6:1221:9505:1678  + CCATTTCCAGTTTTTTATTCCTACTATAAAATAATGTGATATGTTAACCTTTATTTGTATTTTAGATCATTTTCTTAGGGTAGGTTTTCAGAATTAGAATTACTTTGTCAAAGGGTGTGAACCATTTCAAAGATTTAAGTGTATATGTAT
ST-E00144:68:H2LCKCCXX:6:2113:17321:14125+ GTTTTTTATTCCTACTATAAAATAATGTGATATGTTAACCTTTATTTGTATTTTAGATCATTTTCTTAGgGTAGGTTTTCAGAATTAGAATTACTTTGTCAAAGGGTGTGAACCATTTCAAaGATTTAAGTGTATATGTATATTTCtTTT
ST-E00144:68:H2LCKCCXX:1:1205:19473:44398+ TTTTTATTACTACTATAAAATAATGCTATATGTTAACCTtTATTTGTATTTTAGATCATTTTCTTAGGGTAGGTTTTCAGAATTGGGATTACTTTgTcAAAGcGTgTGAACCATTTTAAAGATTTAAGTGTATACGTATATTTCTTTTTC
ST-E00144:68:H2LCKCCXX:8:2101:27288:32795+ TTATTACTACTATAAAATAATGCTATATGTTAACCTTTATTTGTATTTTAGATCATTTTCTTAGtGTAGGTTTTCAGAATTGGGATTACTTTGTCAAAGCGTGTGAACCATTTTAAAGAtTTAAGTGtATACGTATATTTCTTTTTCAAA
ST-E00144:68:H2LCKCCXX:6:1207:4694:71542 + TTCCTTCTATAAAATAATGCTATATGTTAACCTTTATTTGTATTTTAGATCATTTTCTTAGGGTAGGTTTTCAGAATTGGAATTACTTTGTCAAAGGGTGTGAACCATTTTAAAGATTTAAGTGTATATGTATATTTCTTTTTCAAAATC
ST-E00144:68:H2LCKCCXX:6:1207:4704:71560 + TTCCTTCTATAAAATAATGCTATATGTTAACCTTTATTTGTATTTTAGATCATTTTCTTAGGGTAGGTTTTCAGAATTGGAATTACTTTGTCAAAGGGTGTGAACCATTTTAAAGATTTAAGTGTATATGTATATTTCTTTTTCAAAATC
ST-E00144:68:H2LCKCCXX:8:2123:3700:8675  + TTACTACTATAAAATAATGCTATATGTTAACCTTTATTTGTATTTTAGATCATTTTCTTAGGGTAGGTTTTCAGAATTGGGATTACTTTGTCAAAGCGTGTGAACCATTTTAAAGATTTAAGTGTaTACGTATATTTCTTTTTCAAaatc
ST-E00144:68:H2LCKCCXX:1:1124:31237:62066+ CTACTATAAAATAATGTGATATGTTAACCTTTATTTGTATTTTAGATCATTTTCTTAGGGTAGGTTTTCAGAATTAGAATTACTTTGTCAAAGGGTGTGAACCATTTCAAAGATTTAAGTGTATATGTATATTTCTTTTTCAAAATCTTA
ST-E00144:68:H2LCKCCXX:8:2222:15555:65248+ TATAAAATAATGCTATATGTTAACCTTTATTTGTATTTTAGATCATTTTCTTAGGGTAGGTTTTCAGAATTGGAATTACTTTGTCAAAGGGTGTGAACCATTTTAAAGATTTAAGTGTATATGTATATTTCTTTTTCAAAATCTTATTCT
ST-E00144:68:H2LCKCCXX:1:2112:30323:26941+ TAAAATAATGTGATATGTTAACCTTTATTTGTATTTTAGATCATTTTCTTAGGGTAGGTTTTCAGAATTAGAATTACTTTGTCAAAGGGTGTGAAcCATTTCAAAGATTTAAGTGTATATGTATATTTCTTTTTCAAaatctTATTCTac
ST-E00144:68:H2LCKCCXX:1:2212:32465:38843+ CTATATGTTAACCTTTATTTGTATTTTAGATCATTTTCTTAGGGTAGGTTTTCAGAATTGGAATTACTTTGTCAAAGGGTGTGAACcATTTTAAAGATTTAAGTGTAtaTgtATATTTcTTTTTCAAatttTTATTttacatatacattt
ST-E00144:68:H2LCKCCXX:4:1105:23989:55034+ TATATGTTAACCTTTATTTGTATTTTAGATCATTTTCTTAGGGTAGGTTTTCAGAATTGGGATTACTTTGTCAAAGCGTGTGAACCATTTTAAAGATTTAAGTGTATACGTATATTTCTTTTTCAAAATCTTATTCTACCTATACACTCA
ST-E00144:68:H2LCKCCXX:7:1121:16448:28541+ GTGATATGTTAACCTTTATTTGTATTTTAGATCATTTTCTTAGGGTAGGTTTTCAGAATTAGAATTACTTTGTCAAAGGGTGTGAACCATTTCAAAGATTTAAGTGTATATGTATATTTCTTTTTCAAAATCTTATTCTACCTATACACT
ST-E00144:68:H2LCKCCXX:8:1222:13606:56528+ ATATGTTAACCTTTATTTGTATTTTAGATCATTTTCTTAGGGTAGGTTTTCAGAATTAGAATTACTTTGTCAAAGGGTGTGAACCATTTCAAAGATTTAAGTGTATATGTATATTTCTTTTTCAAAATCTTATTCTACCTATACACTCaT
ST-E00144:68:H2LCKCCXX:6:2119:5750:52872 + TGTTAACCTTTATTTGTATTTTAGATCATTTTCTTAGGGTAGGTTTTCAGAATTGGGATTACTTTGTCAAAGCGTGTGAACCATTTTAAAGATTTAAGTGTATACGTATATTTCTTTTTCAAAATCTTATTCTACCTATACACTCATATT
ST-E00144:68:H2LCKCCXX:6:2119:5719:52889 + TGTTAACCTTTATTTGTATTTTAGATCATTTTCTTAGGGTAGGTTTTCAGaATTGGGATTACTTTGTCAAAGCGTGTGAACCATTTTAAAGATTTAAGTGTATACGTATATTTCTTTTTCAAAATCTTATTCTACcTATACACTCATATT
ST-E00144:68:H2LCKCCXX:1:1119:10246:35168+ TTTGTATTTTAGATCATTTTCTTAGGGTAGGTTTTCAGAATTGGAATTACTTTGTCAAAGGGTGTGAACCATTTTAAAGATTTAAGTGTATATGTATATTTCTTTTTCAAAATCTTATTCTACCTATACACTTATATTTGTATAAGACTG

ST-E00144:68:H2LCKCCXX:1:1203:21635:14213+ GTATTTTAGATCATTTTCTTAGGGTAGGTTTTCAGAATTGGGATTACTTTGTCAAAGCGTGTGAACCATTTTAAAGATTTAAGTGTATACGTATATTTCTTTTTCAAAATCTTATTCTACCTATACACTCATATTTGTAAAAGACTGCCA
ST-E00144:68:H2LCKCCXX:1:1203:21665:14406+ GTATTTTAGATCATTTTCTTAGGGTAGGTTTTCAGAATTGGGATTACTTTGTCAAAGCGTGTGAACCATTTTAAAGATTTAAGTGTATACGTATATTTCTTTTTCAAAATCTTATTCTACCTATACACTCATATTTGTAAAAGACTGCCA
ST-E00144:68:H2LCKCCXX:8:1102:10480:6530 + ATTTTAGATCATTTTCTTAGGGTAGGTTTTCAGAATTAGAATTACTTTGTCAAAGGGTGTGAACCATTTCAAAGATTTAAGTGTATATGTATATTTCTTTTTCAAAATCTTATTCTACcTATAcAcTCATATTTGTATAATAcTgCcATG
ST-E00144:68:H2LCKCCXX:1:1120:11891:68008+ TTAGATCATTTTCTTAGGGTAGGTTTTCAGAATTGGGATTACTTTGTCAAAGCGTGTGAACCATTTTAAAGATTTAAGTGTATACGTATATTTCTTTTTCAAAATCTTATTCTACCTATACACTCATATTTGTAAAaGACTGCCATGTAA
ST-E00144:68:H2LCKCCXX:8:1124:21939:23601+ TAgATCATTTtcTTCGGGTAGGTTTTCAGAATTAGAATTACTTTGTCAaAGGtTGTgAACCATTTCAAAcaTgTAcGTGTATaTGTATATtTCTttTTCcAAaTCTTaTTCTaCCTATACactCAtATttTGTATAATAatGCtatgtaaC
ST-E00144:68:H2LCKCCXX:8:1124:21878:23917+ TAGATCATTTTCTTAGGGTAGGTTTTCAGAATTAGAATTACTTTGTCAAAGGGTGTGAACCATTTCAAaGATTTAAGTGTATATGTATATTTCTTTTTCAAAATCTTATTCTACCTATACACTCATATTTGTATAATACTgCCATGTAAC
ST-E00144:68:H2LCKCCXX:1:2116:14408:57495- ACATTATTGTTAGTGACTACATTATATTTTCTTGTATATCTGTACTATAGCATATTTATCTCCCTTTGGGAAGCTTTAGACCATTTCCAGTTTTTTATTCCTACTATAAAATAATGTGATATGTTAACCTTTATTTGTATTTTAGATCAT
ST-E00144:68:H2LCKCCXX:1:2116:14479:57618- ACATTATTGTTAGTGACTACATTATATTTTCTTGTATATCTGTACTATAGCATATTTATCTCCCTTTGGGAAGCTTTAGACCATTTCCAGTTTTTTATTCCTACTATAAAATAATGTGATATGTTAACCTTTATTTGTATTTTAGATCAT
ST-E00144:68:H2LCKCCXX:3:1216:26466:30562- TTATTGTTAGTGaCTACATTATATTTTCTTGTATATCTGTACTATAGCATATTTATCTCCCTTTGGGAAGCTTTAGACCATTTCCAGTTTTTTATTCCTTCTATAAAATAATGCTATATGTTAACCTTTATTTGTATTTTAGATCATTTT
ST-E00144:68:H2LCKCCXX:4:1103:29125:8833 - ATTATATTTTCTTGTATATCTGTACTATAGCATATTTATCTCCCTTTGGGAAGCTTTAGACCATTTCCAGTTTTTTATTCCTTCTATAAAATAATGCTATATGTTAACCTTTATTTGTATTTTAGATCATTTTCTTAGGGTAGGTTTTCA
ST-E00144:68:H2LCKCCXX:3:2217:16499:26589- TATATTTTCTTGTATATCTGTACTATAGCATATTCATCTCCCTTTGGGAAGCTTTAGACCATTTCCAGTTTTTTATTACTACTATAAAATAATGCTATATGTTAACCTTTATTTGTATTTTAGATCATTTTCTTAGGGTAGGTTTTCAGA
ST-E00144:68:H2LCKCCXX:7:1121:18813:50604- TATATTTTCTTGTATATCTGTACTATAGCATATTCATCTCCCTTTGGGAAGCTTTAGACCATTTCCAGTTTTTTATTACTACTATAAAATAATGCTATATGTTAACCTTTATTTGTATTTTAGATCATTTTCTTAGGGTAGGTTTTCAGA
ST-E00144:68:H2LCKCCXX:7:1121:19158:51131- TATATTTTCTTGTAtATCTGTACTATAGCATATTCATCTCCCTTTGGGAAGCTTTAGaCCATTTCCAGTTTTTTATTACTACTATAAAATAATGCTATATGTTAaCCTTTATTTGTATTTTAGATCATTTTCCTAGGGTAGGTTTtCAGA
ST-E00144:68:H2LCKCCXX:6:1209:21107:32426- ATTTTCTTGTATATCTGTACTATAGCATATTTATCTCCCTTTGGGAAGCTTTAGACCATTTCCAGTTTTTTATTCCTTCTATAAAATAATGCTATATGTTAACCTTTATTTGTATTTTAGATCATTTTCTTAGGGTAGGTTTTCAGAATT
ST-E00144:68:H2LCKCCXX:6:1119:23878:32971- GTACTATAGCATATTCATCTCCCTTTGGGaAGCTTTcGACCATTTCCAGTTTTTTATTACTACTATAAAATAATGCTATATGTTAACCTTTATTTGTATTTTAGATCATTTTCTTAGGGTAGGTTTTCAGAATTGGGATTACTTTGTCAA
ST-E00144:68:H2LCKCCXX:6:1119:24639:33867- GTACTATAGCATATTCATCTCCCTTTGGGAAGCTTTAGACCATTTCCAGTTTTTTATTACTACTATAAAATAATGCTATATGTTAACCTTTATTTGTATTTTAGATCATTTTCTTAGGGTAGGTTTTCAGAATTGGGATTACTTTGTCAA
ST-E00144:68:H2LCKCCXX:3:2124:9932:38913 - CTATAGCATATTCATCTCCCTTTGGGAAGCTTTAGACCATTTCCAGTTTTTTATTACTACTATAAAATAATGCTATATGTTAACCTTTATTTGTATTTTAGATCATTTTCTTAGGGTAGGTTTTCAGAATTGGGATTACTTTGTCAAAGC
ST-E00144:68:H2LCKCCXX:3:2124:9932:38983 - CTATAGCATATTCATCTCCCTTTGGGAAGCTTTAGACCATTTCCAGTTTTTTATTACTACTATAAAATAATGCTATATGTTAACCTTTATTTGTATTTTAGATCATTTTCTTAGGGTAGGTTTTCAGAATTGGGATTACTTTGTCAAAGC
ST-E00144:68:H2LCKCCXX:1:1212:21249:15303- GCATATTCATCTCCCTTTGGGAAGCTTTAGACCATTTCCAGTTTTTTATTACTACTATAAAATAATGCTATATGTTAACCTTTATTTGTATTTTAGATCATTTTCTTAGGGTAGGTTTTCAGAATTGGGATTACTTTGTCAAAGCGTGTG
ST-E00144:68:H2LCKCCXX:1:1212:21239:15320- GCATATTCATCTCCCTTTGGGAAGCTTTAGACCATTTCCAGTTTTTTATTACTACTATAAAATAATGCTATATGTTAACCTTTATTTGTATTTTAGATCATTTTCTTAGGGTAGGTTTTCAGAATTGGGATTACTTTGTCAAAGCGTGTG
ST-E00144:68:H2LCKCCXX:1:1212:21259:16375- GCATATtCAtCTCCCTTTGGGAAGCTTTaGACcATTTCCAGTtTTtTATTACTACTATAAAaTAaTGCTATATGTTAACCTTTATTTGTATTTTAGATCATTTTCTTAGGGTAGGTTTTCAGAATTGGGATTACTTTGTCAAAGCGTGTG
ST-E00144:68:H2LCKCCXX:4:2122:5963:22475 - CCTTTgGGAAGCTTTAGACCATTTCCAGTTTTTTATTCCTTCTATAAAATAATGCTATATGTTAACCTTTATTTGTATTTTAGATCATTTTCTTAGGGTAGGTTTTCAGAATTGGAATTACTTTGTCAAAGGGTGTGAACCATTTTAAAG
ST-E00144:68:H2LCKCCXX:6:1218:7090:48477 - gGAAGCTTTAGACCATTTCCAGTTTTTTATTACTACTATAAAATAATGCTATATGTTAACCTTTATTTGTATTTTAGATCATTTTCTTAGGGTAGGTTTTCAGAATTGGGATTACTTTGTCAAAGCGTGTGAACCATTTTAAAGATTTAA
ST-E00144:68:H2LCKCCXX:6:1218:7008:48512 - GGAAGCTTTAGACCATTTCcAGTTTTTTATTACTACTATAAAATAATGCTATATGTTAACCTTTATTTGTATTTTAGATCATTTTCTTAGGGTAGGTTTTCAGAATTGGGATTACTTTGTCAAAGCGTGTGAACCATTTTAAAGATTTAA
ST-E00144:68:H2LCKCCXX:1:1217:23157:16006- GCTTTAGACCATTTCCAGTTTTTTATTCCTTCTATAAAATAATGCTATATGTTAACCTTTATTTGTATTTTAGATCATTTTCTTAGGGTAGGTTTTCAGAATTGGAATTACTTTGTCAAAGGGTGTGAACCATTTTAAAGATTTAAGTGT
ST-E00144:68:H2LCKCCXX:8:2121:16275:43959- TTTAGaCCATTTCCAGTTTTTTATTACTACTATAAAATAATGCTATATGTTAACCTTTATTTGTATTTTAGATCATTTTCTTAGGGTAGGTTTTCAGAATTGGGATTACTTTGTCAAAGCGTGTGAACCATTTTAAAGATTTAAGTGTAT
ST-E00144:68:H2LCKCCXX:3:2103:19950:24550- TTTCCAGTTTTTTATTCCTTCTATAAAATAATGCTATATGTTAACCTTTATTTGTATTTTAGATCATTTTCTTAGGGTAGGTTTTCAGAATTGGAATTACTTTGTCAAAGGGTGTGAACCATTTTAAAGATTTAAGTGTATATGTATATT
ST-E00144:68:H2LCKCCXX:1:2104:2339:5686  - TTTTTTATTCCTACTATaAAATAATGTgaTATGTTAACCTTTATTTGTATTTTAGATCATTTTCTTAgGGgAGGTTTTCAGAATTAGAATTACTTTGTCAAAGGGTGTGAACCATTTCAAAGATTTAAGTGTATATGTATATTTCTTTTT
ST-E00144:68:H2LCKCCXX:1:2104:2502:5827  - TTTTTTATTCCTACTATAAAATAATGTtATATGTTAACCTTTATTTgTATTTTAGATCATTTTCTTAGGgtAGGTTTTCAGAATTAGAATTACTTTGTCAAAGGGTGTGAACCATTTCAAAGATTTAAGTGTATATGTATATTTCTTcTT
ST-E00144:68:H2LCKCCXX:6:1103:12835:65213- TTTTTTATTCCTACTATAAAATAATGTGATATGTTAACCTTTATTTGTATTTTAGATCATTTTCTTAGGGTAGGTTTTCAGAATTAGAATTACTTTGTCAAAGGGTGTGAACCATTTCAAAGATTTAAGTGTATATGTATATTTCTTTTT
ST-E00144:68:H2LCKCCXX:3:2209:15616:45980- TTTTATTCCTACTATAaAATAATGTGATATGTTAACCTTTATTTGTATTTTAGATCATTTTCTTAGGGTAGGTTTTCAGAATTAGAATTACTTTGTCAAAGGGTGTGAACCATTTCAAAGATTTAAGTGTATATGTATATTTCTTTTTCa
ST-E00144:68:H2LCKCCXX:1:2116:6674:3664  - TCTATAAAATAATGCTATATGTTAACCTTTATTTGTATTTTAGATCATTTTCTTAGGGTAGGTTTTCAGAATTGGAATTACTTTGTCAAAGGGTGTGAACCATTTTAAAGATTTAAGTGTATATGTATATTTCTTTTTCAAAATCTTATT
ST-E00144:68:H2LCKCCXX:1:2116:6623:3788  - CTATAAAATAATGCTATATGTTAACCTTTATTTGTATTTTAGATCATTTTCTTAGGGTAGGTTTTCAGAATTgGAATTACTTTGTCAAAGGGTGTGAACCATTtTAAAGATTTAAGTGTATATGTATATTTCTTTTTCAAAATCTTATT
ST-E00144:68:H2LCKCCXX:6:2206:8582:30211 - aaatCatGctaTATGTtAaCctttaTTTGTAtTtAGAtCattTtCTTAGGGTAGGTTtTCAGaAgTGGGATTACTTTGTCAAAGCGTGTGAACCATTtgAAAGAtTTAAGTGtATACGTATATTtCTTTTTCAAAAtCtTATTCTACCT
ST-E00144:68:H2LCKCCXX:8:2103:13890:16551- TAATGCTATATGTTAACCTTtaTTTGTATTTtaGaTccTTTTCtTAGGGTAGGTTTTCAGAATTGGAATTACTTTGTCAAAGGGTGTGAACCATTTTAAAGATTTAAGTGTATaTGTATATTTCTTTTTCAAAATCTTATTCTACCTATA
ST-E00144:68:H2LCKCCXX:6:2109:22558:49496- ATATGTTAACCTTTATTTGTATTTTAGATCATTTTCTTAGGGTAGGTTTTCAGAATTGGGATTACTTTGTCAAAGCGTGTGAACCATTTTAAAGATTTAAGTGTATACGTATATTTCTTTTTCAAAATCTTATTCTACCTATACACTCAT
ST-E00144:68:H2LCKCCXX:1:1113:12672:70241- ACCTTTATTTGTATTTTAGATCATTTTCTTAGGGTAGGTTTTCAGAATTAGAATTACTTTGTCAAAGGGTGTGAACCATTTCAAAGATTTAAGTGTATATGTATATTTCTTTTTCAAAATCTTATTCTACCTATACACTCATATTTGTAT
ST-E00144:68:H2LCKCCXX:4:1107:23969:67200- TTTATTTGtATTtTAGATCATTTTCTTAGgGTAGGTTTTCAGAATTGGAATTACTTTGTCAAAGGGTGTGAACCATTTTAAAGATTTAAGtGTATATGTATATTTCTTtTTCAAAaTCTTATTCTACCtATACACTTATATTTGTATAAG
ST-E00144:68:H2LCKCCXX:1:1213:19483:65055- TTGTATTTTatATCATTTTCTTAGGGTAGGTTTTCAGAATTGGAATTACTTTGTCAAAGGGTGTGAACCATTTTAAAGATTTAAGTGTATATGTATATTTCTTTTTCAAAATCTTATTCTACCTATACACTTATATTTGTATAAGAcTG
ST-E00144:68:H2LCKCCXX:1:2101:19442:23249- TGTATTTTagATCATTTTCTTAgGGTAGGTTTTCAGAATTAGAATTACTTTGTCAAAGGGTGTGAACCATTTCAAAGATTTAAGTGTATATGTATATTTCTTTTTCAAAATCTTATTCTACCTATACACTCATATTTGTATAATACTGCC
ST-E00144:68:H2LCKCCXX:1:2101:19239:23530- TGTATTTTAGATCATTTTCTTAGGGTAGGTTTTCAGAATTAGAATTACTTTGTCAAAGGGTGTGAACCATTTCAAAGATTTAAGTGTATATGTATATTTCTTTTTCAAAATCTTATTCTACCTATACACTCATATTTGTATAATACTGCC
ST-E00144:68:H2LCKCCXX:7:1212:9505:51395 - TTTTAGATCATTTTCTTAGGGTAGGTTTTCAGAATTGGGATTACTTTGTCAAAGCGTGTGAACCATTTTAAAGATTTAAGTGTATACGTATATTTCTTTTTCAAAATCTTATTCTACCTATACACTCATATTTGTAAAAGACTGCCATGT
ST-E00144:68:H2LCKCCXX:1:1201:21746:38843- TTTAGATCATTTTCTTAGGGTAgGTTTTCAGAATTGgGATTACTTTGTCAAAGCGTGTGAACCATTTTAAAGATTTAAGTGTATACGTATATTTCTTTTTCAAAATCTTATTCTACCTATACACTCATATTTGTAAAAGACTGCCATGTA
```

```
ST-E00192:51:H2KG3CCXX:1:1210:2705:60431 + AAAAACATTATTGTTAGTGACTACATTATATTTTCTTGTATATCTGTACTATAGCATATTTATCTCCCTTTGGGAAGCTTTAGACCATTTCCAGTTTTTTATTtcTTCTATAAAATAatGcTaTATGtTaACcTTTaTTTGTATTTTcga
ST-E00192:51:H2KG3CCXX:2:1102:24406:63033+ AACATTATTGTTAGTGACTACATTATATTTTCTTGTATATCTGTACTATAGCATATTTATCTCCCTTTGGGAAGCTTTAGACCATTTCCAGTTTTTTATTCCTACTATAAAATAATGTGATATGTTAACCTTTATTTGTATTTTAGATCA
ST-E00192:51:H2KG3CCXX:2:1102:22771:63473+ AACATTATTGTTAGTGACTACATTATATTTTCTTGTATATCTGTACTATAGCATATTTATCTCCCTTTGGGAAGCTTTAGACCATTTCCAGTTTTTTATTCCTACTATAAAATAATGTGATATGTTAACCTTTATTTGTATTTTAGATCA
ST-E00192:51:H2KG3CCXX:3:2213:8775:10749 + TGTTAGTGACTACATTATATTTTCTTGTATATCTGTACTATAGCATATTTATCTCCCTTTGGGAAGCTTTAGACCATTTCCAGTTTTTTATTCCTTCTATAAAATAATGCTATATGTTAACCTTTATTTGTATTTTAGATCATTTTCTTA
ST-E00192:51:H2KG3CCXX:2:2207:22061:50780+ TGACTACATTATATTTTCTTGTATATCTGTACTATAGCATATTCATCTCCCTTTGGGAAGCTTTAGACCATTTCCAGTTTTTTATTACTACTATAAAATAATGCTATATGTTAACCTTTATTTGTATTTTAGATCATTTTCTTAGGGTAG
ST-E00192:51:H2KG3CCXX:2:2124:13139:33850+ TTATATTTTCTTGTATATCTGTACTATAGCATATTCATCTCCCTTTGGGAAGCTTTAGACCATTTCCAGTTTTTTATTACTACTATAAAATAATGCTATATGTTAACCTTTATTTGTATTTTAGATCATTTTCTTAGGGTAGGTTTTCAG
ST-E00192:51:H2KG3CCXX:6:1118:28354:21456+ ATTTTCTTGTATATCTGTACTATAGCATATTTATCTCCCTTTGGGAAGCTTTAGACCATTTCCAGTTTTTTATTCCTTCTATAAAATAATGCTATATGTTAACCTTTATTTGTaTTTTAGATCATTTTCTTAGGgTAGGTTTTCAGAATT
ST-E00192:51:H2KG3CCXX:6:1118:28151:21561+ ATTTTCTTGTATATCTGTACTATAGCATATTTATCTCcCTTTGGGAAGCTTTAGACCATTTCCAGTTTTTTATTCCTTCTATaAAaTAATGCTATaTGTTAAcCTTTATTTGTATTTTAGATtATTTTCTTagGgTAGgTTTTaGAATT
ST-E00192:51:H2KG3CCXX:8:1206:14976:59183+ TGTATATCTGTACTATAGCATATTTATCTCCCTTTGGGAAGCTTTAGACCATTTCCAGTTTTTTATTCCTACTATAAAATAATGTGATATGTTAACCTTTATTTGTATTTTAGATCATTTTCTTAGGGTAGGTTTTCAGAATTAGAATTA
ST-E00192:51:H2KG3CCXX:3:2216:30516:29174+ TATATCTGTACTATAGCATATTTATCTCCCTTTGGGAAGCTTTAGACCcTTTCCAGTTTTTTATTCCTACTATAAAATAATGTGATATGTTAAccTTTATTTGTatTTTAgATCATTTTCTTAGGGTAGGTTTTtaGaaTTAGAATTAcT
ST-E00192:51:H2KG3CCXX:2:2208:3009:34905 + TATCTGTACTATAGCATATTTATCTCCCTTTGGGAAGCTtTAGaCCATTTCCAGTtTTTTATTCcTAttAtAAAATAATGTGATATGtTaACCTTTATTTGTATTTTAGtTcatTttCTTAGGGTAggaTtTtAgcaTtAGtaTtacttt
ST-E00192:51:H2KG3CCXX:6:1113:11759:37823+ TATAGCATATTTATCTCCCTTTGGGAAGCTTTAGACCATTTCCAGttTTtTATTCCTACTATAAAATAATGTGATATGTTAACCTTTATTTGTATTTTAGaTCATTTTCTTAGGGTAGGTTTTCAGAATTAGAaTTACTTTGTCAAAGGG
ST-E00192:51:H2KG3CCXX:6:2110:11911:44451+ TATAGCATATTTATCTCCCTTTGGGAAGCTTTAGACCATTTCCAGTTTTTTATTCCTTCTATAAAATAATGCTATATGTTAACCTTTATTTGTATTTTAGATCATTTTCTTAGGGTAGGTTTTCAGAATTGGAATTACTTTGTCAAAGGG
ST-E00192:51:H2KG3CCXX:1:1119:28110:39071+ ATAGCATATTTATCTCCCTTTGGGAAGCTTTAGACCATTTCCAGTTTTTTATTCCTACTATAAAATAATGTGATATGTTAACCTTTATTTGTATTTTAGATCATTTTCTTAGGGTAGGTTTTCAGAaTTAGAATTACTTTGTCAAAGGGT
ST-E00192:51:H2KG3CCXX:1:1213:24994:43449+ AGCATATTCATCTCCCTTTGGGAAGCTTTAGACCATTTCCAGTTTTTTATTACTACTATAAAATAATGCTATATGTTAACCTTTATTTGTATTTTAGATCATTTTCTTAGGGTAGGTTTTCAGAATTGGGATTACTTTGTCAAAGCGTGT
ST-E00192:51:H2KG3CCXX:3:1213:25075:43449+ AGCATATTCATCTCCCTTTGGGAAGCTTTAGACCATTTCCAGTTTTTTATTACTACTATAAAATAATGCTATATGTTAACCTTTATTTGTATTTTAGATCATTTTcTTAGGGTAGGTTTTCAGAATTGGGATTACTTTGTCAAaGCGTGT
ST-E00192:51:H2KG3CCXX:5:1217:2218:73195 + AGcATATTTATCTCCCTtTGGGAAGCTTTAGACCATTTCCAGaTTTTTATTCCTACTATAAAATAATGTGATATGTTAACCTTTATTTGTAtTTTAGATtATTTTcttAGGGtAGGTTTTcagAATTAGAATTactTTGttaAAGGGTGT
ST-E00192:51:H2KG3CCXX:8:2208:12378:11435+ AGCATATTTATCTCCCTTTGGGAAGCTTTAGACCATTTCCAGTTTTTTATTCCTTCTATAAAATAATGCTATATGTTAACCTTTATTTGTATTTTAGATCATTTTCTTAGGGTAGGTTTTCAGAATTGGAATTACTTTGTCAAAGGGTGT
ST-E00192:51:H2KG3CCXX:8:2119:16945:42658+ CTCCCTTTGGGAAGCTTTAGACCATTTCCAGTTTTTTATTCCTACTATAAAATAATGTGATATGTTAACCTTTATTTGTATTTTAGATCATTTTCTTAGGGTAGGTTTTCAGAATTAGAATTACTTTGTcAAAGgGTGTGAACCATTTca
ST-E00192:51:H2KG3CCXX:8:1114:6430:32531 + TTGGGAAGCTTTAGACCATTTCCAGTTTTTTATTCCTTCTATAAAATAATGCTATATGTTAACCTTTATTTGTATTTTAGATCATTTTCTTAGGGTAGGTTTTCAGAATTGGAATTACTTTGTCAAaGGGTGTGAACCATTTTAAAGATT
ST-E00192:51:H2KG3CCXX:1:2204:31115:63719+ TTTAGACCATTTCCAGTTTTTTATTACTACTATAAAATAATGCTATATGTTAACCTTTATTTGTATTTTAGATCATTTTcTTAGGGTAGGTTTTCAGAATTGGGATTACTTTGTCaAAGCGTGTGAACCATTTTAAAGATTTAAGTGTAT
ST-E00192:51:H2KG3CCXX:3:2102:12733:72913+ CCAGTTTTTTATTCCTTCTATAAAATAATGCTATATGTTAACCTTTATTTGTATTTTAGATCATTTTCTTAGGGTAGGTTTTCAGAATTGGAATTACTTTGTCAAAGGGTGTGAACCATTTTAAAGATTTAAGTGTATATGTATATTTCT
ST-E00192:51:H2KG3CCXX:2:1101:19676:64158+ TTTTTATTACTACTATAAAATAATGCTATATGTTAACCTTTATTTGTATTTTAGATCATTTTCTTAGGGTAGGTTTTCAGAATTGGGATTACTTTGTCAAAGCGTGTGAACCATTTTAAAGATTTAAGTGTATACGTATATTTCTTTTTC
ST-E00192:51:H2KG3CCXX:2:1106:17128:71946+ ATTCCTACTATAAAATAATGTGATATGTTAACCTTTATTTGTATTTTAGATCATTTTCTTAGGGTAGGTTTTCAGAATTAGAATTACTTTGTCAAAGGGTGTGAACCATTTCAAAGATTTAAGTGTATATGTATATTTCTTTTTcaAAAT
ST-E00192:51:H2KG3CCXX:3:2116:31683:59464+ ATTACTACTATAAAATAATGCTATATGTTAACCTTTATTTGTATTTTAGATCATTTTCTTAGGGTAGGTTTTCAGAATTGGGATTACTTTGTCAAAGCGTGTGAACCATTTTAAAGATTTAAGTGTATACGTATATTTCTTTTTCAAAAT
ST-E00192:51:H2KG3CCXX:8:1215:24832:52626+ ATTACTACTATAAAATAATGCTATATGTTAACCTTTATTTGTATTTTAGATCATTTTCTTAGGGTAGGTTTTCAGAATTGGGATTACTTTGTCAAAGcGTGTGAACCATTTTAAAGATTTAAGTGTATAcgTATATTTCTTTTTCAAAAT
ST-E00192:51:H2KG3CCXX:1:1208:7617:64580 + CCTTCTATAAAATAATGCTATATGTTAACCTTTATTTGTATTTTAGATCATTTTCTTAGGGTAGGTTTTCAGAATTGGAATTACTTTGTCAAAGGGTGTGAACCATTTTAAAGATTTAAGTGTATATGTATATTTCTTTTTCAAAATCTT

ST-E00192:51:H2KG3CCXX:1:1208:7689:64668 + CCTTCTATAAAATAATGCTATATGTTAACCTTTATTTGTATTTTAGATCATTTTCTTAGGGTAGGTTTTCAGAATTGGAATTACTTTGTCAAAGGGTGTGAACCATTTTAAAGATTTAAGTGTATATGTATATTTCTTTTTCAAaATCTT
ST-E00192:51:H2KG3CCXX:1:2208:6937:63754 + CCTTCTATAAAATAATGCTATATGTTAACCTTTATTTGTATTTTAGATCATTTTCTTAGGGTAGGTTTTCAGAATTGGAATTACTTTGTCAAAGGGTGTGAACCATTTTAAAGATTTAAGTGTATATGTATATTTCTTTTTCAAAATCTT
ST-E00192:51:H2KG3CCXX:6:1204:10388:20577+ AAATAATGCTATATGTTAACCTTTATTTGTATTTTAGATCATTTTCTTAGGGTAGGTTTTCAGAATTGGAATTACTTTGTCAAAGGGTGTGAACCATTTTAAAGATTTAAGTGTATATGTATATTTCTTTTTCAAAATCTTATTCTACCT
ST-E00192:51:H2KG3CCXX:6:1204:10327:20612+ AaATAATGCTATATGTTAACCTTTaTTTGTATtTTAGATCATTTTCTTAGGGTAGGTTTTCAGAATTGGAATTACTTTGTCAAAGGGTGTGAACCATTTTAAAGATTTAAGTGTATATGTATATTTCTTTTTCAAAATCTTATTCTacCT
ST-E00192:51:H2KG3CCXX:6:2203:11586:71208+ AAATAATGCTATATGTTAACCTTTATTTGTATTCTAGATCATTTTCTTAGGGTAGGTTTTCAGAATTGGAATTACTTTGTCAAAGGGTGTGAACCATTTTAAAGATTTAAGTGTATATGTATATTTCTTTTTCAAAATCTTATTCTACCT
ST-E00192:51:H2KG3CCXX:3:2102:10855:69556+ ATAATGCTATATGTTAACCTTTATTTGTATTTTAGATCATTTTCTTAGGGTAGGTTTTCAGAATTGGAATTACTTTGTCAAAGGGTGTGAACCATTtTAAAGATTTAAGTGTATATGTATATTTCTTTTTCAAAATCTTATTCTACCTAT
ST-E00192:51:H2KG3CCXX:8:1224:18224:35239+ GTGATATGTTAACCTTTATTTGTATTTTAGATCATTTTCTTAGGGTAGGTTTTCAGAATTAGAATTACTTTGTCAAAGGGTGTGAACCATTTCAAAGATTTAAGTGTATATGTATATTTCTTTTTCAAAATCTTATTCTACCTATACACT
ST-E00192:51:H2KG3CCXX:3:1219:15646:8165 + ATGTTAACCTTTATTTGTATTTTAGATCATTTTCTTAGGGTAGGTTTTCAGAATTGGGATTACTTTGTCAAAGCGTGTGAACCATTTTAAAGATTTAAGTGTATACGTATATTTCTTTTTCAAAATCTTATTCTACCTATACACTCATAT
ST-E00192:51:H2KG3CCXX:8:1213:16154:31617+ GTTAACCTTTATTTGTATTTTAGATCATTTTCTTAGGGTAGGTTTTCAGAATTAGAATTACTTTGTCAAAGggTGTGAACCATTTCAAgATTTAAGTGTATATGTATaTTTCTTTTTCAAAATCTTAtTCTAcTTATAcaCTcaTATTt
ST-E00192:51:H2KG3CCXX:8:1213:16143:31635+ GTtAACaTgTaTTTGTATTTTAgATtaTTTTtTTAGGGTAGGTTTTCAGAATTAGAATTACTTTGTCAAAgggTGTGAaccAtTTCAAAGatTTAAttgTaTATaTATATTTCtttttTagaaatctTATTctaCtTATAcactcATAttt
ST-E00192:51:H2KG3CCXX:1:2111:8115:33727 + TTAACCTTTATTTGTATTTTAGATCATTTTCTTAGGGTAGGTTTTCAGAATTGGGATTACTTTGTCAAAGCGTGTGAACCATTTTAAAGATTTAAGTGTATACGTATATTTCTTTTTCAaaaTCTTATTCTACcTATACACTCATATTTG
ST-E00192:51:H2KG3CCXX:3:2113:7607:44802 + AACCTTTATTTGTATTTTAGATCATTTTCTTAGGGTAGGTTTTCAGAATTGGAATTACTTTGTCAAAGGGTGTGAACCATTTTAAAGATTTAAGTGTATATGTATATTTCTTTTTCAAAATCTTATTCTACCTATACACTTATATTTGTA
ST-E00192:51:H2KG3CCXX:3:2113:7668:44873 + AACCTTTATTTGTATTTTAGATCATTTTCTTAGGGTAGGTTTTCAGAATTGGAATTACTTTGTCAAAGGGTGTGAACCATTTTAAAGATTTAAGTGTATATGTATATTTCTTTTTCAAAATCTTATTCTACCTATACACTTATATTTGTA
ST-E00192:51:H2KG3CCXX:1:1212:13301:62506+ CCTTTATTTGTATTTTAGATCATTTTCTTAGGGTAGGTTTTCAGAATTGGAATTACTTTGTCAAAGGGTGTGAACCATTTTAAAGATTTAAGTGTATATGTATATTTCTTTTTCAAAATCTTATTCTACCTATACACTTATATTTGTATA
ST-E00192:51:H2KG3CCXX:8:1201:5892:29279 + TTTATTTGTATTTTAGATCATTTTCTTAGGGTAGGTTTTCAGAATTGGAATTACTTTGTCAAAGGGTGTGAACCATTTTAAAGATTTAAGTGTATATGTATATTTCTTTTTCAAAATCTTATTCTACCTATACACTTATATTTGTATAAG
ST-E00192:51:H2KG3CCXX:8:1201:5679:29683 + TTTATTTGTATTTTAGATCATTTTCTTAGGGTAGGTTTTCAGAATTGGAATTACTTTGTCAAAGGGTGTGAACCATTTTAAAGATTTAAGTGTATATGTATATTTCTTTTTCAAAATCTTATTCTACCTATACACTTATATTTGTATAAG
ST-E00192:51:H2KG3CCXX:8:1201:5719:29683 + TTTATTTGTATTTTAGATCATTTTCTTAGGGTAGGTTTTCAGAATTGGAATTACTTTGTCAAAGGGTGTGAACCATTTTAAAGATTTAAGTGTATATGTATATTTCTTTTTCAAAATCTTATTCTACCTATACACTTATATTTGTATAAG
ST-E00192:51:H2KG3CCXX:1:1110:5060:66690 + GTATTTTAGATCATTTTCTTAGGGTAGGTTTTCAGAATTAGAATTACTTTGTCAAAGGGTGTGAACCATTTCAAAGATTTAAGTGTATATGTATATTTCTTTTTCAAAATCTTATTCTACCTATACACTCATATTTGTATAATACTGCCA
ST-E00192:51:H2KG3CCXX:2:2203:20193:13720- AAaACATTATTgtTAgTGACTACATTATATTTTCTTGtaTATCTGTACTATAGCAtATTCAtCTCcCtTTGGGAAGCTTTaGACCATTTCCAgtTTTTTATTACTACtaTAAAATAATgCTaTATGTTAACCTTTATTTGTAtTTTAGAT
ST-E00192:51:H2KG3CCXX:5:1213:20975:67006- ACATTATTGTTAGTGACTACATTATATTTTCTTGTATATCTGTACTATAGCATATTTATCTCCCTTTGGGAAGCTTTAGACCATTTCCAGTTTTTTATTCCTACTATAAAATAATGTGATATGTTAACCTTTATTTGTATTTTAGATCAT
ST-E00192:51:H2KG3CCXX:2:2118:19422:53030- TATTGTTAGTGACTACATTATATTTTCTTGTATATCTGTACTATAGCATATTTATCTCCCTTTGGGAAGCTTTAGACCATTTCCAGTTTTTTATTCCTTCTATAAAATAATGCTATATGTTAACCTTTATTTGTATTTTAGATCATTTTC
ST-E00192:51:H2KG3CCXX:3:1112:10459:49496- ACTACATTATATTTTCTTGTATATCTGTACTATAGCATATTTATCTCCCTTTGGGAAGCTTTAGACCATTTCCAGTTTTTTATTCCTTCTATAAAATAATGCTATATGTTAACCTTTATTTGTATTTTAGATCATTTTCTTAGGGTAGGT
[illegible]
```

```
ST-E00192:51:H2KG3CCXX:3:1112:10470:49514- ACTACATTATATTTTCTTGTATATCTGTACTATAGCATATTTATCTCCCTTTGGGAAGCTTTAGACCATTTCCAGTTTTTTATTCCTTCTATAAAATAATGCTATATGTTAACCTTTATTTGTATTTTAGATCATTTTCTTAGGGTAGGT
ST-E00192:51:H2KG3CCXX:3:1112:10429:49619- ACTACATTATATTTTCTTGTATATCTGTACTATAGCATATTTATCTCCCTTTGGGAAGCTTTAGACCATTTCCAGTTTTTTATTCCTTCTATAAAATAATGCTATATGTTAACCTTTATTTGTATTTTAGATCATTTTCTTAGGGTAGGT
ST-E00192:51:H2KG3CCXX:3:1105:16154:4579 - CATTATATTTTCTTGTATATCTGTACTATAGCATATTTATCTCCCTTTGGGAAGCTTTAGACCATTTCCAGTTTTTTATTCCTTCTATAAAATAATGCTATATGTTAACCTTTATTTGTATTTTAGATCATTTTCTTAGGGTAGGTTTTC
ST-E00192:51:H2KG3CCXX:2:1119:8287:31494 - TATTTTCTTGTATATCTGTACTATAGCATATTTATCTCCCTTTggGAAGCTTTAGACCATTTCCAGTTTTTTATTCCTTCTATAAAATAATGCTATATGTTAACCTTTATTTGTATTTTAGATCATTTTCTTAGGGTAGGTTTTCAGAAT
ST-E00192:51:H2KG3CCXX:2:1119:8277:31512 - TATTTTCTTGTATATCTGTACTATAGCATATTTATCTCCCTTTGGGAAGCTTTAGACCATTTCCAGTTTTTTATTCCTTCTATAAAATAATGCTATATGTTAACCTTTATTTGTATTTTAGATCATTTTCTTAGGGTAGGTTTTCAGAAT
ST-E00192:51:H2KG3CCXX:8:1123:11647:72228- ATCTCCCTTTGGGAAGCTTTAGACCATTTCCAGTTTTTTATTCCTTCTATAAAATAATGCTATATGTTAACCTTTATTTGTATTTTAGATCATTTTCTTAGGGTAGGTTTTCAGAATTGGAATTACTTTGTCAAAGGGTGTGAACCATTT
ST-E00192:51:H2KG3CCXX:2:1115:19158:72333- TTGGGAAGCTTTAGACCATTTCCAGTTTTTTATTCCTACTATAAAATAATGTGATATGTTAACCTTTATTTGTATTTTAGATCATTTTCTTAGGGTAGGTTTTCAGAATTAGAATTACTTTGTCAAAGGGTGTGAACCATTTCAAAGATT
ST-E00192:51:H2KG3CCXX:2:2207:22061:50780- TggGAAGCTTTAGACCATTTCCAGTTTTTTATTACTACTATAAAATAATGCTATATGTTAACCTTTATTTGTATTTTAGATCATTTTCTTAGGGTAGGTTTTCAGAATTGGGATTACTTTGTCAAAGCGTGTGAACCATTTTAAAGATTT
ST-E00192:51:H2KG3CCXX:6:1203:14398:43554- TGGGAAGCTTTagACCATTTCCAGTTTTTTATTCCTACTATAAAATAATGTGATATGTTAACCTTTATTTGTATTTTAGATCATTTTCTTAGGGTAGGTTTTCAGAATTAGAATTACTTTGTCAAAGGGTGTGAACCATTTCAAAGATTT
ST-E00192:51:H2KG3CCXX:3:1218:6491:13474 - GGAAGCTTTAGACCATTTCCAGTTTTTTATTACTACTATAAAATAATGCTATATGTTAACCTTTATTTGTATTTTAGATCATTTTCTTAGGGTAGGTTTTCAGAATTGGGATTACTTTGTCAAAGCGTGTGAACCATTTTAAAGATTTAA
ST-E00192:51:H2KG3CCXX:3:1218:6552:13474 - GGAAGCTTTAGACcATTTCCAgTTTTTTATTACTACTATAgAATAATGCTATATGTTAaCCTTTATTTGTATTTTAGATCATTTTCTTAGGGTAGGTTTTCAGAAtTGGGATTACTTTGTCAAAGCGTGTGAACCATTTTAAAGATTTAA
ST-E00192:51:H2KG3CCXX:2:2220:27380:69766- AAGCTTTAGACCATTTCCAGTTTTTTATTCCTACTATAAAATAATGTGATATGTTAACCTTTATTTGTATTTTAGATCATTTTCTTAGGGTAGGTTTTCAGAATTAGAATTACTTTGTCAAAGGGTGTGAACCATTTCAAAGATTTAAGT
ST-E00192:51:H2KG3CCXX:2:2220:27380:69977- AAGCTTTAGACCATTTCCAGTTTTTTATTCCTACTATAAAATAATGTGATATGTTAACCTTTATTTGTATTTTAGATCATTTTCTTAGGGTAGGTTTTCAGAATTAGAATTACTTTGTCAAAGGGTGTGAACCATTTCAAAGATTTAAGT
ST-E00192:51:H2KG3CCXX:6:1207:26354:12578- TTTAGAcCATTTCcaGTTTTTTATTCCTACTATAAAATAATGTGATATGTTAACCTTTATTTGTATTTTAGATCATTTTCTTAGGGTAGGTTTTCAGAATTAGAATTACTTTGTCAAAGGGTGTGAACCATTTCAAAGATTTAAGTGTAT
ST-E00192:51:H2KG3CCXX:2:1102:24406:63033- GACCATTTCCAGTTTTTTATTCCTACTATAAAATAATGTGATATGTTAACCTTTATTTGTATTTTAGATCATTTTCTTAGGGTAGGTTTTCAGAATTAGAATTACTTTGTCAAAGGGTGTGAACCATTTCAAAGATTTAAGTGTATATGT
ST-E00192:51:H2KG3CCXX:2:1102:22771:63473- GACCATTTCCAGTTTTTTATTCCTACTATAAAATAATGTGATATGTTAACCTTTATTTGTATTTTAGATCATTTTCTTAGGGTAGGTTTTCAGAATTAGAATTACTTTGTCAAAGGGTGTGAACCATTTCAAAGATTTAAGTGTATATGT
ST-E00192:51:H2KG3CCXX:6:1217:14093:53153- CCATTTCCAGTTTTTTATTCCTTCTATAAAATAATGCTATATGTTAACCTTTATTTGTATTTTAGATCATTTTCTTAGGGTAGGTTTTCAGAATTGGAATTACTTTGTCAAAGGGTGTGAACCATTTTAAAGATTTAAGTGTATATGTAT
ST-E00192:51:H2KG3CCXX:8:2112:3740:12507 - TTTATTCCTTCTATAAAATAATGCTATATGTTAACCTTTATTTGTATTTTAGATCATTTTCTTAGGGTAgGTTTTCAGAATTGGAATTACTTTGTCAAAGGGTGTGAACCATTTTAAAGATTTAAGTGTATATGTATATTTCTTTTTCAA
ST-E00192:51:H2KG3CCXX:1:2118:31084:32479- ACTACTATAAAATAATGCTATATGTTAACCTTTATTTGTATTTTAGATCATTTTCTTAGGGTAGGTTTTCAGAATTGGGATTACTTTGTCAAAGCGTGTGAACCATTTTAAAGATTTAAGTGTATACGTATATTTCTTTTTCAAAATCTT
ST-E00192:51:H2KG3CCXX:1:1213:9688:8218  - CTACTATAAAATAATGTGATATGTTAACCTTTATTTGTATTTTAGATCATTTTCTTAGGGTAGGTTTTCAGAATTAGAATTACTTTGTCAAAGGGTGTGAACCATTTCAAAGATTTAAGTGTATATGTATATTTCTTTTTCAAAATCTTA
ST-E00192:51:H2KG3CCXX:6:2210:10246:21983- ATAATGTGATATGTTAACCTTTATTTGTATTTTAGATCATTTTCTTAGGGTAGGTTTTCAGAATTAGAATTACTTTGTCAAAGGGTGTGAACCATTTCAAAGATTTAAGTGTATATGTATATTTCTTTTTCAAAATCTTATTCTACCTAT
ST-E00192:51:H2KG3CCXX:6:2210:10256:22106- ATAATGTGATATGTTAACCTTTATTTGTATTTTAGATCATTTTCTTAGGGTAGGTTTTCAGAATTAGAATTACTTTGTCAAAGGGTGTGAACCATTTCAAAGATTTAAGTGTATATGTATATTTCTTTTTCAAAATCTTATTCTACCTAT
ST-E00192:51:H2KG3CCXX:2:1222:16864:1520 - TATTTTAGATCATTTTCTTAGGGTAGGTTTTCAGAATTGGGATTACTTTGTCAAAGCGTGTGAACCATTTTAAAGATTTAAGTGTATACGTATATTTCTTTTTCAAAATCTTATTCTACCTATACACTCATATTTGTAAAAGACTGCCAT
```

---

chr11 22363026 + 5 chr2 222205926 + 0 CTX 60 44 33 0 0.99625 0 0.982898550724638 0 1 chr11 22362910 150 chr2 222205957

GAGGTCTTGCAGCTTGGTGTCACCCCACCACCCTAATCCCCGAGGGTGGGGGGAGGTCGACGGCCCTCCTCCCAACCCCGACGGGTCCTGAAAAATGGGAATGGGTCAGCATCGGGGCATTATTTTTCTCAGTGGAATTATAAATGTATT

```
22362880 22362900 22362920 22362940 22362960 22362980 22363000 22363020 22363040 22363060 22363080 22363100 22363120 22363140 22363160

chr11:22362877-22363173 CCAAGGGCACCTGCAGAATGTGAGAGAGCCTGGGAGGTCTTGCAGCTTGGTGTCACCCCACCACCCTAATCCCCGAGGGTGGGGGGAGGTCGACGGCCCTCCTCCCAACCCCGACGGGTCCTGAAAAATGGGAATGGGTCAGCATCGGGGGTACCTAAGCCGCCAAGTCCTTGACTCGCCCCTGCTCTGCCGCGCAGGGTGCTGGAGAAGAAGCAAGACACCGGGGAGACAATCGAGCTGACGGAGGATGGGAAGCCCCTAGAGGTGCCCGAGAGGAAGGCGCCGCTGTGCGACTGC

ST-E00144:68:H2LCKCCXX:4:2205:31774:57443+ CAAGGGCACCTGCAGAATGTGAGAGAGCCTGGGAGGtCTTGCAGCTTGGTGTCACCCCACCACCCTAATCCCCGAGGGTGGGgGGAGGtcGaCGgCCCtcctCCcAACCCCGAcgGgtcctGaaaaagggcaacGcGtcaGcaTgggGgg
ST-E00144:68:H2LCKCCXX:1:1204:16387:9554 + AGGGCACCTGCAGAATGTGAGAGAGCCTGGGAGGTCTTGCAGCTTGGTGTCACCCCACCACCCTAATCCCCGAGGGTGGGGGGAGGTCGACGGCCCTCCTCCCAACCCCGACGGGTCCTGAAAAATGGGAATGGGTCAGCATCGGGGGtA
ST-E00144:68:H2LCKCCXX:4:1223:7546:65582 + AGGGCACCTGCAGAATGTGAGAGAGCCTGGGAGGTCTTGCAGCTTGGTGTCACCCCACCACCCTAATCCCCGAGGGTGGGGGGAGGTCGACGGCCCTCCTCCCAACCCCGACgGGTCCTGAaAAaTGGGgATGGGTCAGCAtCGgGGGTa
ST-E00144:68:H2LCKCCXX:8:1209:5080:30193 + GGGCACCTGCAGAATGTGAGAGAGCCTGGGAGGtCTTGCAGCTTGGTGTCACCCCAcCACCCTAATcCCCGAGGGTGGGGGGAGgtcGACGGCCcTCCTcCCAACCCCGACGGGTcctGaaaaatgGGAATGGGaCgGcAtcGGgGGtac
ST-E00144:68:H2LCKCCXX:6:1220:20478:5212 + AGAATGTGAGAGAGCCTGGGAGGTCTTGCAGCTTGGTGTCACCCCACCACCCTAATCCCCGAGGGTGGGGGGAGGtCGACGGCCCTCCTCCCAACCCCGaCGGGTCCTGAAAAATGGGAATGGGTCAGCATCGGGGGtACCTAAGCCGCC
ST-E00144:68:H2LCKCCXX:8:1119:24162:46332+ GAGAGAGCCTGGGAGGtCTTGCAGCTTGGTGTCACCCCACCACCCTAATCCCCGAGgGTGGGGGGAGGtCGACGGCCCTCCTCCCAACCCCGACGGGgCCtGAAaaaTGGGAAtGGGTCaGCATCGGGGgtacCtAcGCCGCCaAGTCCT
ST-E00144:68:H2LCKCCXX:8:1216:4938:64651 + AGCCTGGGAGGTCTTGCAGCTTGGTGTCACCCCACCACCCTAATCCCCGAGGGTGGGGGGAGGTCGACGGCCCTCCTCCCAACCCCGACGGGTCCTGaAAAAtGGGAATGGGtcagCATCGGGGGtacCtcaGcCGCCaAGtCCtTGACT
ST-E00144:68:H2LCKCCXX:4:1213:19229:13035+ GGGAGGTCTTGCAGCTTGGTGTCACCCCACCACCCTAATCCCCGAGGGTGGGGGGAGGTCGACGGCCCTCCTCCCAACCCCGACGGGTCCTGAAAAATGGGAATGGGTCAGCATCGGGGGTACCTAAGCCGCCAAGTCCTTGACTCGCCC
ST-E00144:68:H2LCKCCXX:1:1124:15900:15566+ GGAGGTCTTGCAGCTTGGTGTCACCCCACCACCCTAATCCCCGAGGGTGGGGGGAGGTCGACGGCCCTCCTCCCAACCCCGACGGGTCCTGAaAaatGGGAATGGGTCAGCATCGGGgGTACCTAAGCCGCCAAgtcCTTGACtcGCCCC
ST-E00144:68:H2LCKCCXX:1:1212:19534:31600+ GAGGTCTTGCAGCTTGGTGTCACCCCACCACCCTAATCCCCGAGGGTGGGGGGAGGTCGACGGCCCTCCTCCCAACCCCGACGGGTCCTGAAAAATGGGAATGGGTCAGCATCGGGGCATTATTTTTCTCAGTGGAATTATAAATGTATT
ST-E00144:68:H2LCKCCXX:1:1212:19737:31740+ GAGGTCTTGCAGCTTGGTGTCACCCCACCACCCTAATCCCCGAGGGTGGGGGGAGGTCGACGGCCCTCCTCCCAACCCCGACGGGTCCTGAAAAATGGGAATGGGTCAGCATCGGGGCaTTATTTTTCTCAGTGGAATTATAAATGTATT
ST-E00144:68:H2LCKCCXX:1:1212:19848:31793+ GAGGTCTTGCAGCTTGGTGTCACCCCACCACCCTAATCCCCGAGGGTGGGGGGAGGTCGACGGCCCTCCTCCCAACCCCGACGGGTCCTGAAAAATGGGAATGGGTCAGCATCGGGGCATTATTTTTCTCAGTGGAATTATAAATGTATT
ST-E00144:68:H2LCKCCXX:3:1120:8186:41656 + GAGGTCTTGCAGCTTGGTGTCACCCCACCACCCTAATCCCCGAGGGTGGGGGGAGGTCGACGGCCCTCCTCCCAACCCCGACGGGTCCTGAAAAATGGGaATGGGTCAGCATCGGGGCATTATTTTTCTCAGTGGAATTATAAATGTaT
ST-E00144:68:H2LCKCCXX:3:1120:8247:41656 + GAGGTCTTGCAGCTTGGTGTCACCCCACCACCCTAATCCCCGAGGGTGGGGGGAGGTCGACGGCCCTCCTCCCAACCCCGACGGGTCCTGAAAAATGGGAATGGGTCAGCATCGGGGCATTATTTTTCTCAGTGGAATTATAAATGTATT

ST-E00144:68:H2LCKCCXX:3:1221:22233:23688+ CAGCTTGGTGTCACCCCACCACCCTAATCCCCGAGGGTGGGGGaGGcCGACGGCCCtCCTCCCAACCCCGACGGGTCCtGAgAaATGGGcaTGGGtcaGCaTCGGGGGtaCctAagCCGCCAagtccttGACTCgcCCCtGCtCtGccG
ST-E00144:68:H2LCKCCXX:3:1221:22274:23759+ CaGCTTGGTGTCACCCCACCACCCTAATCCCCGAGGGtGGGGGaGgtCGACGGCCCTCCTCCCAACCCCGACGGGtCCTGAAAaAtGGGAatGGGtCAGCcTCGGGGGtACCTAaGCCGCCAAGtCCtTGACTCGCCCCTGCtCTGCCG
ST-E00144:68:H2LCKCCXX:8:1114:7222:46490 + ACCACCCTAATCCCCGaGGGTGGGGgGAGGtCGACGgCCCTCCTCCCAACCCCGACgGGTCCTGAAAAATGGGAATGGGTCAGCAcCGGGGGTaCCTAAGCCGCCAAGTCCcTGACTCGCCCCtGCTCTGCCGCGCAGgGTGCtgGcG
ST-E00144:68:H2LCKCCXX:6:1123:9495:63649 + TAATCCCCGAGGGTGGGGGGAGGTCGACGGCCCTCCTCCCAACCCCGACGGGTCCTGAAAAATGGGAATGGGTCAGCATCGGGGGtACCTAAGCCGCCAAGtCCTTGcCTCGCCCCTGCTCTGCCGCGCaGGGtgctgGaGAaGaAGCaA
ST-E00144:68:H2LCKCCXX:6:1222:27237:38474+ GGCCCTCCTCCCAACCCCGACGGGTCCTGAAAAATGGGAATGGGTCAGCATCGGGGGtaCCTAAGCCGCCAAGTCCTTGACTCGCCCCTGCtCTGCCGCGCaGGGgGCTGgagAaGAAGCAAGAcacCGGGGagaCAAtCgAGCtGAcGg
ST-E00144:68:H2LCKCCXX:3:2123:17027:30633+ CTCCTCCCAACCCCGACGGGTCCTGAAAAATGGGAATGGGTCAGCATCGGGGGTACCTAAGCCGCCAAGTCCTTGACTCGCCCCTGCTCTGCCGCGCAGGGTGCTGGAGAAGAAGCAAGACACCGGGGAGACAATCGAGCTGACGGAGGA
ST-E00144:68:H2LCKCCXX:3:2213:30719:12648+ CCTCCCAACCCCGACGGGtCCTGAAAAATGGGAATGGGTCAGCATCGGGGGtACCTAAGCCGCCAAGTCCTtGACTCGCCCCTGCTCTGCCGCGCAGGGtGCtgGAGAAGAAGCaAGacaccGGGGaGacaatcGAGctgacgGaggagG
ST-E00144:68:H2LCKCCXX:1:1212:18417:2522 + CCAACCCCGACGGGTCCTGAAAAATGGGAATGGGTCAGCATCGGGGGTACCTAAGCCGCCAAGTCCTTGACTCGCCCCTGCTCTGCCGCGCAGGGTGCtGGAGAAGAAGCAAGACACCGGGGAGACAAtCGAGCTGaCGGAGGaTGGGAA
ST-E00144:68:H2LCKCCXX:6:2120:13474:67129+ CCAACCCCGACGGGTCCTGAAAAATGGGAATGGGTCAGCATCGGGGGTACCTAAGCCGCCAAGTCCTTGACTCGCCcCTGCTCTGCCGCGCAGGGTGCTGGAGAAGAAGCAAGACACCGGGgAgACAATCGAGCTGACGGAGGATGGGAA
ST-E00144:68:H2LCKCCXX:8:2123:18285:26413+ CAACCCCGACGGGTCCTGAAAAATGGGAATGGGTCAGCATCGGGGGTACCTAAGCCGCCAAGTCCTTGACTCGCCCCTGCTCTGCCGCGCAGGGTGCTGGAGAAGAAGCAAGACACCGGGGAGACAaTCGAGCTGACGGAGGATGGGAAG
ST-E00144:68:H2LCKCCXX:3:1224:15240:55104+ CCGACGGGTCCTGAAAAATGGGAATGGGTCAGCATCGGGGGTACCTAAGCCGCCAAGTCCTTGACTCGCCCCTGCTCTGCCgCGCAGGGgGCTGGAGAAgAAGCAAGACACCGGgGAGACAATCGAGCTGACGGAGGATGGGAAGCCCct
ST-E00144:68:H2LCKCCXX:4:1110:25644:68800+ CGACGggTccTGaAacAtGgGcaTGgGTCaGcATCGGgggTaCctAAGCCGCCaAGtCctTgAcTcgccccTGCTcTgCCgcGCaGGGTGCaGGcgaCGAAGCAAGACAccgtggAgACatTcgAgctcaCggcggatgGgAaGcccta
ST-E00144:68:H2LCKCCXX:7:1101:7546:51166 + ACGGGTCCTGAAAAATGGGAATGGGTCAGCATCGGGGGTACCTAAGCCGCCAAGTCCTTGACTCGCCcCTGCTCTGCCGCGCAGGGtgCTGGaGAAGAAGCAAGACACCGgGGAGACAATCGaGCTGACGGAgGAtGGGAAGCCCC
ST-E00144:68:H2LCKCCXX:8:1102:15991:72755+ GGGTCCTGAAAAATGGGAATGGGTCAGCaTCGGGGGTACCTAAGCCGCCAAGTCCTTGACTCGCCcCTGCTCTGCCgCGCAgGgTGCTGGagAAGAAGCAaGaCACCGGGGAGACAAtCGAGCTGACGGAGGATGGGAAGCCCCaAGAGG
ST-E00144:68:H2LCKCCXX:3:1111:5933:67393 + TCCTGAAAaATGGGAATGGGTCAGCaTCgGGcgtaCCTAAGCCGCCAAgTcCtTGACTCGCCCCtGCtcTGCCGCGCAggGtGCTGGAGaagAAGCaagACACCGGGGaGaCaatCGaGctgaCGGaggaTGGGaagCCcCtagaggcgC
ST-E00144:68:H2LCKCCXX:8:2121:4562:56159 + TGAAAAATGGGAATGGGTCAGCATCGGGGGTACCTAAGCCGCCAAGTCCTTGACTCGCCCCTGCTCTGCCGCGCAGGGTGCTGGAGAAGAAGCAAGACACCGGGGAGACAATCGAGCTGACGGAGGATGGGAAGCCCCTAGAGGtGCCCG
ST-E00144:68:H2LCKCCXX:1:2212:26456:50551+ ATGGGTCAGCATCGGGGGTACCTAAGCCGCCAAGTCCTTGACTCGCCCCTGCTCTGCCGCGCAGGGTGCTGGAGAAGAAGCAAGACACCGGGGAGACAATCGAGCTGACGGAGGATGGGAAGCCCCTAGAGGTGCCCGAGAGGAAGGCGC
ST-E00144:68:H2LCKCCXX:6:2218:27440:25394+ GGGTCAGCATCGGGGGTACCTAAGCCGCCAAGTCCTTGACTCGCCCCTGCTCTGCCGCGCAGGGTGCTGGAGAAGAAGCAAGACACCGGGGaGACAATCGAGCTGaCGGAGGaTGGGAAGCCCCTaGAGGtgcccGaGaGGAAGGCGCcg
ST-E00144:68:H2LCKCCXX:1:1218:14865:18028+ GGTCAGCATCGGGGGTACCTAAGCCGCCAAGTCCTTGACTCGCCCCTGCTCTGCCGCGCAGGGTGCTGGAGAAGAAGCAAGACACCGGGGAGACAATCGAGCTGACGGAGGATGGGAAGCcCCTAGAGgTGCcCGAGAGGAAGGCGCCGC
ST-E00144:68:H2LCKCCXX:4:1119:31460:44310+ GTCAGCATCGGGGGTACCTAAGCCGCCAAGTCCTTGACTCGCCCCTGCTCTGCCGCGCAGGGTGCTGGAGAAGAAGCAAGACACCGGGGAGACAATCGAGCTGACGGAGGATGGGAAGCCCCtAGAGGTGCCCGAgaGGAAGGCGCCGCT
ST-E00144:68:H2LCKCCXX:1:1117:4654:19856 + aGCATCGGGGGTACCTAAGCCGCCAAGTCCTTGACTCGCCCCTGCTCTGCCGCGCAGGGTGCTGGAGAAGAAGCAaGACACCGGGGAGACAATCGAGCTGACGGAGGATGGGAAGCCCCTAGAGGTGCCCGAGAGGAAGGCGCCGCTGTG
ST-E00144:68:H2LCKCCXX:1:2116:28405:15918+ CATCGGGGGTACCTAAGCCGCCAAGTCCTTGACTCGCCCCTGCTCTGCCGCGCAGGGTGCTGGAGAAGAAGCAAGACACCGGGGAGACAATCGAGCTGACGGAGGATGGGAAGCCCCtAGAGGTGCCCGAGAGGAAGGCGCCGCTGTGCG
ST-E00144:68:H2LCKCCXX:3:1203:5060:58462 + ATCGGGGGTACCTAAGCCGCCAAGTCCTTGACTCGCCCCTGCTCTGCCGCGCAGGGTGCTGGAGAAGAAGCAAGACACCGGGGAGACAATCGAGCTGACGGAGGATGGGAAGCCCCTAGAGGTGCCCGAGAGGAAGGCGCCGcTGTGCGA
ST-E00144:68:H2LCKCCXX:6:1107:19422:12349+ GGGGTACCTAAGCCGCCAAGTCCTTGACTCGCCCCTGCTCTGCCGCGCAGGGTGCTGGAGAAGAAGCAAGACACCGGGGAGACAATCGAGCTGACGGAGGATGGGAAGCCCCTAGAGGTGCCCGAGAGGAAGGCGCCGCTGTGCGACTGC
ST-E00144:68:H2LCKCCXX:7:1103:2877:21491 + GGGGtACCTAAGCCGCCAAgTCCTTGacTcGccCcTGcTCTGCCgCGcAgGGtGCTGGAGAAGAAgCAAGACACCGGGGAGACAATCGAGCtGACGGAGGATGGgAAGcCCCTAGAGGTGCcCGAgAGGAAGGCGCCGCTGTGCGACtGC
ST-E00144:68:H2LCKCCXX:4:2122:29064:66215- ccaaGGGCaCCtGCaGAAtgtGaGAGAGCCTGGGAGgtcTTGCAGCTtGGcGTCaCcCCaCCaCCCTAAgCCCCGAGGGTGGGGGGAGGTCGACGGCCCTCCTCCCAACCCCGACGGGTCCTGAAAAATGGGAATGGGTCAGCATCGGGG
ST-E00144:68:H2LCKCCXX:8:1109:28293:12033- AAGGGCACCTGCAGAATGTGAGAGAGCCTGGGAGGTCTTGCAGCTTGGTGTCACCCCACCACCCTAATCCCCGAGGGTGGGGGGAGGTCGACGGCCCTCCTCCCAACCCcGACGGGTCCTGAAAAATGGGAATGGGTCAGCATCGGGGGT
ST-E00144:68:H2LCKCCXX:4:1210:7820:7198  - cacaggGcaCctgCAcAATGtGaGatAGCCTGGgaGGtCttgcAgCTTGGtGtCaccCCaCCaCCCtaAtccCCGAGGGtgggGGGAggTcGACGgCCcTCCTCCCAACCCCGACGgGTCCTGAAAAaTGGGAATGGGTCAGCATCGGGG
ST-E00144:68:H2LCKCCXX:8:1109:28303:11910- AGGGCACCTGCAGAATGTGAGAGAGCCTGGGAGGTCTTGCAGCTTGGTGTCACCCCACCACCCTAAtCCCCGAGGGTGGGGGGAGGTCGACGGCCCTCCTCCCAACCCCGACGGGTCCTGAAAAATGGGAATGGGTCAGCATCGGGGGT
ST-E00144:68:H2LCKCCXX:3:2123:13464:42675- GAAtgtgAGagAGcCtGGGAGgtCtTGCAGcgtGGtGTCACcCCACCaCCCtAcCtCCCCGaGGGTGGGGGGAGGTCgacGGCCCtcCtCCCaACCCCGACGgGtCCTGAAAAATGGGAatGgGTCcGcATCGGGGGTACCTAAGCCGCCA
ST-E00144:68:H2LCKCCXX:3:2207:26791:35450- AGAGCCTGGGAGGTCTTGCAGCTTGGTGTCACCCCACCACCCTAATCCCCGAGGGTGGGGGGAGGTCGACGGCCCTCCTCCCAACCCCGACGGGTCCTGAAAAATGGGAATGGGTCAGCATCGGGGGTACCTAAGCCGCCAAGTCCTTGA
ST-E00144:68:H2LCKCCXX:3:2207:26842:35502- AGAGCCTGGGAGGTCTTGCAGCTTGGTGTCACCCCACCACCCTAATCCCCGAGGGTGGGGGGAGGTCGACGGCCCTCCTCCCAACCCCGACGGGTCCTGAAAAATGGGAATGGGTCAGCATCGGGGGTACCTAAGCCGCCAAGTCCTTGA
ST-E00144:68:H2LCKCCXX:1:2107:32434:21174- GCCtGGGAGGTCTtGCAGCTTGGTGTCaCCCCACCACCCTaAtCCCCGAGGGTGGGGGGAGGTCGACGGCCCTCCTCCCAACCCCGACGGGTCCTGAAAAATGGGAATGGGTCAGCATCGGGGGTACCTAAGCCGCCAAGTCCTTGACTC
ST-E00144:68:H2LCKCCXX:8:2215:12692:21298- GCCTGGGAGGTCttGCAGCTTGGTGTcaCCCCACCACCCTAAtCCCCGAGGGTGGGGGGAGGTCGACGGCCCTCCTCCCAACCCCGACGGGTCCTGAAAAATGGGAATGGGTCAGCATCGGGGGTACCTAAGCCGCCAAGTCCTTGACTC
ST-E00144:68:H2LCKCCXX:1:1207:22893:63719- GAGGTCTTGCAGCTTGGTGTCACCCCACCaCCCTAATCCCCGAGGGTGGGGGGAGGTCGACGGCCCTCCTCCCAACCCCGACGGGTCCTGAAAAATGGGAATGGGTCAGCATCGGGGGTACCTAAGCCGCCAAGTCCTTGACTCGCCCCT
ST-E00144:68:H2LCKCCXX:6:2104:16549:22352- GTCTTGCAGCTTGGTGTCACCCCACCACCCTAAtCCCCGAGGGTGGGGGGAGGTCGACGGCCCTCCTCCCAACCCCGACGGGTCCTGAAAAATGGGAATGGGTCAGCATCGGGGGTACCTAAGCCGCCAAGTCCTTGACTCGCCCCTGCT
ST-E00144:68:H2LCKCCXX:3:1219:11353:35256- CTTGCAGCTTGGTGTCACCCCACCACCCTaATCCCCGAGGGTGGGGGGAGGTCGACGGCCCTCCTCCCAACCCCGACGGGTCCTGAAAAATGGGAATGGGTCAGCATCGGGGGTACCTAAGCCGCCAAGTCCTTGACTCGCCCCTGCTCT
ST-E00144:68:H2LCKCCXX:6:2218:9150:55456 - TTGCAGCTtGGTGTCACCCCACCACCCTAATCCCCGAGGGTGGGGGGAGGTCGACGGCCCTCCTCCCAACCCCGACGGGTCCTGAAAAATGGGAATGGGTCAGCATCGGGGGTACCTAAGCCGCCAAGTCCTTGACTCGCCCCTGCTCTG
ST-E00144:68:H2LCKCCXX:1:1113:10084:5282 - TGTCAcCCCacCACCCTAATCCCCGAGGGTGGGGGGAGGTCGACGGCCCTCCTCCCAaCCCCGACGGGTCCTGAAAAATGGGAATGGGTCAGCATCGGGGGTACCTAAGCCGCCAAGTCCTTGACTCGCCCCTGCTCTGCCGCGCAGGGT
ST-E00144:68:H2LCKCCXX:8:1224:27917:1889 - CCcCAcCcCCCtaAtCCCCGAGGGTGGGGGGAGGtCGACgGCCCTCctCCCaaCCCCGaCGGGTCCTGAAAAATGGGAATGGGTCAGCAtCGGGGGTACCTAAGCCGCCAAGTCCTTGACTCGCCCCTGCTCTGCCGCGCAGGGTGCT
ST-E00144:68:H2LCKCCXX:3:1213:9972:46297 - cCCCTactCccCggGgtGGGGGGAGGTCgacggCCCtCctCcCAACCcCGACGGGtCCTGAAAAATGGGAATGGGTCAGCAtCGGGGGTACCTAAGCCGCCAAGTCCTTGACTCGCCCCTGCTCTGCCGCGCAGGGTGCTGGAGAAGAA
ST-E00144:68:H2LCKCCXX:6:1112:17676:42095- TaatCCCCGAGGGtGGGGGGAGGTCGACGGCCCTCCtCCCAACCCCGACGGGTCCTGAAAAATGGGAATGGGTCAGCATCGGGGGTACCTAAGCCGCCAAGTcCTTGACTCGCCCCTGCTCTGCCGCGCAGGGTGCTGGAGAAGAAGCAA
ST-E00144:68:H2LCKCCXX:8:2122:19940:10011- aatCcccGAGGGtGGGGGGAGGTCGACGGCCCTCCTCCCAACCCCGACGGGTCCTGAAAAATGGGAATGGGTCAGCATCGGGGGTACCTAAGCCGCCAAGTCCTTGACTCGCCCCTGCTCTGCCGCGCAGGGTGCTGGAGAAGAAGCAAG
ST-E00144:68:H2LCKCCXX:8:2105:6562:30193 - cccgcatcccCgAGGGtgGGgggcGgtcgACGGCCCtCCTcCcacCcCCGacGgGtcCtGaaacATGGGaaTGGgtCaGcatCGGGGGtAcCtaAGcCGCCaaGTCCTTGACTCGcCcCcGCTCtgCCGCGCAGGGTGCTGGAGAAGAAG
ST-E00144:68:H2LCKCCXX:7:1107:13027:24655- CCCGAGGGTGGGGGGAGGTCGACGGCCCTCCTCCCAaCCCCGACGGGTCCTGAAAAATGGGAATGGGTCAGCATCGGGGGTACCTAAGCCGCCAAGTCCTTGACTCGCCCCTGCTCTGCCGCGCAGGGTGCTGGAGAAGAAGCAAGACAC
ST-E00144:68:H2LCKCCXX:8:2120:25756:20893- GGtGGGGGGAGGtCGcCGGCCCtCCTCCCaaCCCCGACGGGTCCTGAAaaATGGGAATGGGTCAGCATCGGGGGTaCCtAAGCCGCCAAGTCCTTGACTCGCCCCTGCTCTGCCGCGCAGGGTGCTGGAGAAGAAGCAAGACACCGGGGA
ST-E00144:68:H2LCKCCXX:6:2122:23035:36294- GTCGACGGCCCtcCtCCCAACCCCGACGGGTCCTGAAAaaTGGGAATGGGTCAGCATCGGGGGTACCTaAGCCGCCAAGTCCTTGACTCGCCCCTGCTCTGCCGCGCAGGGTGCTGGAGAAGAAGCAAGACACCGGGGAGACAATCGAGC
ST-E00144:68:H2LCKCCXX:4:1119:19148:68553- TCcgCCCacCcCcgaCGGGtCCTGAAAaATGGGaATGGGTCAGCATCGGGGGTACCTAAGCCGCCAAGTCCTTGACTCGCCCCTGCTCTGCCGCGCAGGGTGCTGGAGAAGAAGCAAGACACCGGGGAGACAATCGAGCTGACGGAGGAT
ST-E00144:68:H2LCKCCXX:4:2120:10602:39792- GCCccCCAaCCcCGaCGGGgCCTGAAAAAtGGGAATGGGTCAGCATaTGGGGTACCTAAGCCGCCAAGTCctTGACTCGCCCCTGCTCTGCCGCGCAGGGTGCTGGAGAAGAAGCAAGACACCGGGGAGACAATCGAGCTGACGGAGGAT
ST-E00144:68:H2LCKCCXX:7:1223:30303:47721- ggcgGgtccTgaAAaaggGGaagGGGTCagcatCGGGGGtAcCTAAGCCGCCaAGTCCgTGactcgccCcTGcTCtGCCGCGCAGGGTGCTGGAGAAGAAGCAAGACACCGGGGAGACAATCGAGCTGACGGAGGATGGGAAGCCCCTAG
ST-E00144:68:H2LCKCCXX:1:2223:9729:60572 - CGggtccTGAacaATGGGaATGgGTCAGCAtCGGGGGTACCTAaGCCGCCaAGTCCTTGcCTCGCCCCTGCTCTGCCGCGCAGGGTGCTGGAGAAGAAGCAAGACACCGGGGAGACAATCGAGCTGACGGAGGATGGGAAGCCCCTAGAG
ST-E00144:68:H2LCKCCXX:7:2117:13677:37067- CTGAaaAATGGGaatGGGTCAGCaTCGGGGGTACCtaAGCCGCcAAGTCCTTGaCTCGCCCcTGCTCTGCCGcGCAGGGTGcgGGAGAAGAAGCAAGAcacCGGGGAGACAATcGAGCTGACGGAGGAGGGGAAGCCCCTAGAGGTGCCC
ST-E00144:68:H2LCKCCXX:8:1209:5080:30193 - GGGAATGGGTCAGCATCGGGGGTACCTAAGCCGCCAAGTCCTTGACTCGCCCCTGCtCTGCCGCGCAGGGTGCTGGAGAAGAAGCAAGACACCGGGGAGACAATCGAGCTGACGGAGGATGGGAAGCCCCTAGAGGTGCCCGAGAGGAAG
ST-E00144:68:H2LCKCCXX:8:1113:13220:6741 - gTCAGCaTCgggggtgcCtaagaCGCCAAGTCCTcGaCTCGCCCCTGCTCTGCCGCGCaGgGTgcTGgagAAGAAgcaAGACACcGGGGAGACAATCGAGCTGaCGGAGGATGGGAAGCCCCTAGAGGTgCcCGAGAGGAAGGCGCCGCT
ST-E00144:68:H2LCKCCXX:1:2106:21208:43537- TCAGCATCGGGGGTACCTAAGcCGCCAAGTCCTTGActCGCCCCTGCTCTGCCGCGCAGGGTGCTGGAGAAGAAGCAAGACACCGGGGAGACAATCGAGCTGACGGAGGATGGGAAGCCCCTAGAGGTGCCCGAGAGGAAGGCGCCGCTG
ST-E00144:68:H2LCKCCXX:1:1104:14946:37999- GcatcGGGGGtAcctaagcCGCCAaGTCCTTGactcGccCCTGCTCtGCCGCGCAGGGTGcTGGAGAAGAAGCAagACAcCgGGGAGACAATCGAGCTGACGGAGGATGGGAAGCCCCTAGAGGTGcCCGAGAGGAAGGCGCCGCtGTGC
```

```
ST-E00192:51:H2KG3CCXX:1:2223:13748:13808+ CCAAGGGCACCTGCAGAATGTGAGAGAGCCTGGGAGGTCTTGCAGCTTGGTGTCACCCCACCACCCTAATCCCCGAGGGTGGGGGGAGGtCGACGGCCCTCCTCCCAACCCCGACGGGTCCTGAAAAATGGGAATGGGtCaGCAtCGGGG
ST-E00192:51:H2KG3CCXX:3:2220:13301:44961+ AAGGGCACCTGCAGAATGTGAGAGAGCCTGGGAGGTCTTGCAGCTTGGTGTCACCCCACCACCCTAATCCCCGAGGGTGGGGGGAGGtCGACGGCCCTCCTCCCAACCCCGACGGGTCCTGAAaAATGGGAATGGGTCAGCATCGGGGG
ST-E00192:51:H2KG3CCXX:6:2109:30029:16867+ AGGGCACCTGCAGAATGTGAGAGAGCCTGGGAGGTCTTGCAGCTTGGTGTCACCCCACCACCCTAATCCCCGAGGGTGGGGGGagGgCGACGGCCCTCCTCCCAACCCCGACGGGTCCTGcaAaatGGGAatggGtcAGCATCGGGGG
ST-E00192:51:H2KG3CCXX:6:2109:30018:16920+ AGGGCACCTGCAGAATGTGAGAGAGCCTGGGAGGTCTTGCAGCTTGGTGTCACCCCACCACCCTAATCCCCGAGGGTGGGGGGaGGTCGACGGCCCTCCTCCCAACCCCGACGGGTCCTGaAAAATGGGAATGGGTCAGCATCGGGGGtA
ST-E00192:51:H2KG3CCXX:8:1118:27024:5053 + TgCAGCTTGGTGTCACCCCACCACCCTAATCCCCGAGGGTGGGGGGgGGtCGaCGGCCCTCCTCCCAACCCCGaCggGTCcTGAAAaATGGGAATGGGTCAGCATCGGGGGtACCTAAGCgGCCAAGTCctTGACTCGCCCCtgcTCTGC
ST-E00192:51:H2KG3CCXX:8:1204:16641:4930 + ACCACCCTAATCCCCGAGGGTGGGGGGAGGTCGACGGCCCTCCTCCCAACCCCGACGGGTCCTGAAAaaTGGGAATGGGTCAGCATCGGGGGtACCTAAGCCGCCAAGTCCTTGACTCGCCCCTGCTCTGCCGCGCagGGtGCTGGaGaA
ST-E00192:51:H2KG3CCXX:8:1224:30100:56335+ GAGGGTGGGGGGAGGtCGACGGCCCTCCTCCCAACCCCGACGGGTCCTGAAAAATGGGAATGGGTCAGCATCGGGGGTACCTAAGCCGCCAAGTCCTTGACTCGCCCCTGcTctGCCGCGCAGGGTGCTGGAGAAGAaGCaAGACACCGG
ST-E00192:51:H2KG3CCXX:6:1212:29521:26466+ GGTGGGGGGAGGTCGACGGCCCTCCTCCCAACCCCGACGGGtCCTGAAAAAtGGGaaTGGGTCAGCATCGGGGGTACCTAAgCcGCCAAGTCCTtGACTCGCCCCTGCTCTGCCGcGCaGGGTGCtGGcGAaGaAGCAAGgCaCcggGGC
ST-E00192:51:H2KG3CCXX:8:1115:27806:62945+ GGTCCTGAAAAATGGGAATGGGTCAGCATCGGGGGTACCTAAGCCGCCAAGTCCTTGACTCGCCCCTGCTCTGCCGCGCAGGGTGCTGGAGAAGAAGCAAGACACCGGGGaGACAATCGAGCTGACGGAGGATGGGAAGCCcCtAgAGGT
ST-E00192:51:H2KG3CCXX:8:1115:28019:64229+ GGTCCTGAAAAATGGGAATGGGTCAGCATCGGGGGTACCTAAGCCGCCAAGTCCTTGACTCGCCCCTGCTCTGCCGCGCAGGGTGCTGGAGAAGAAGCAAGACACCGGGGAGACAAtCGAGCTGACGGAGGaTGGGAAGCCCCTAGAGGT
ST-E00192:51:H2KG3CCXX:3:1121:11312:7163 + GAAAAATGGGAATGGATCAGCATCGGGGGTACCTAAGCCGCCAAGTCCTTGACTCGCCCCTGCTCTGCCGCGCAGGGTGCTGGAGAAGAAGCAAGACACCGGGGAGACAATCGAGCTGACGGAGGATGGGAAGCCCCTAGAGGTGCCCGA
ST-E00192:51:H2KG3CCXX:3:1121:11332:7233 + GAAAAATGGGAATGGGTCAGCATCGGGGGTAcCTAAGCCGCCaAGTcCTTGACTCGCcCCTGCtcTGCcgcGCaGGGTGctGgAGAAGAaGCAAGACACCGGGGagACAATCGAGCTGACGGAGGATGGGAAGCCCataGaGGTGCccGA
ST-E00192:51:H2KG3CCXX:3:2211:13322:37507+ TGGGAATGGGTCAGCATCGGGGGTACCTAAGCCGCCAAGTCCTTGACTCGCCCCTGCTCTGCCGCGCAGGGTGCTGGAGAAGAAGCAAGACACCGGGGAGACAATCGAGCTGACGGAGGATGGGAAGCCCCTAGAGGTGCCCGAGAGGAA
```

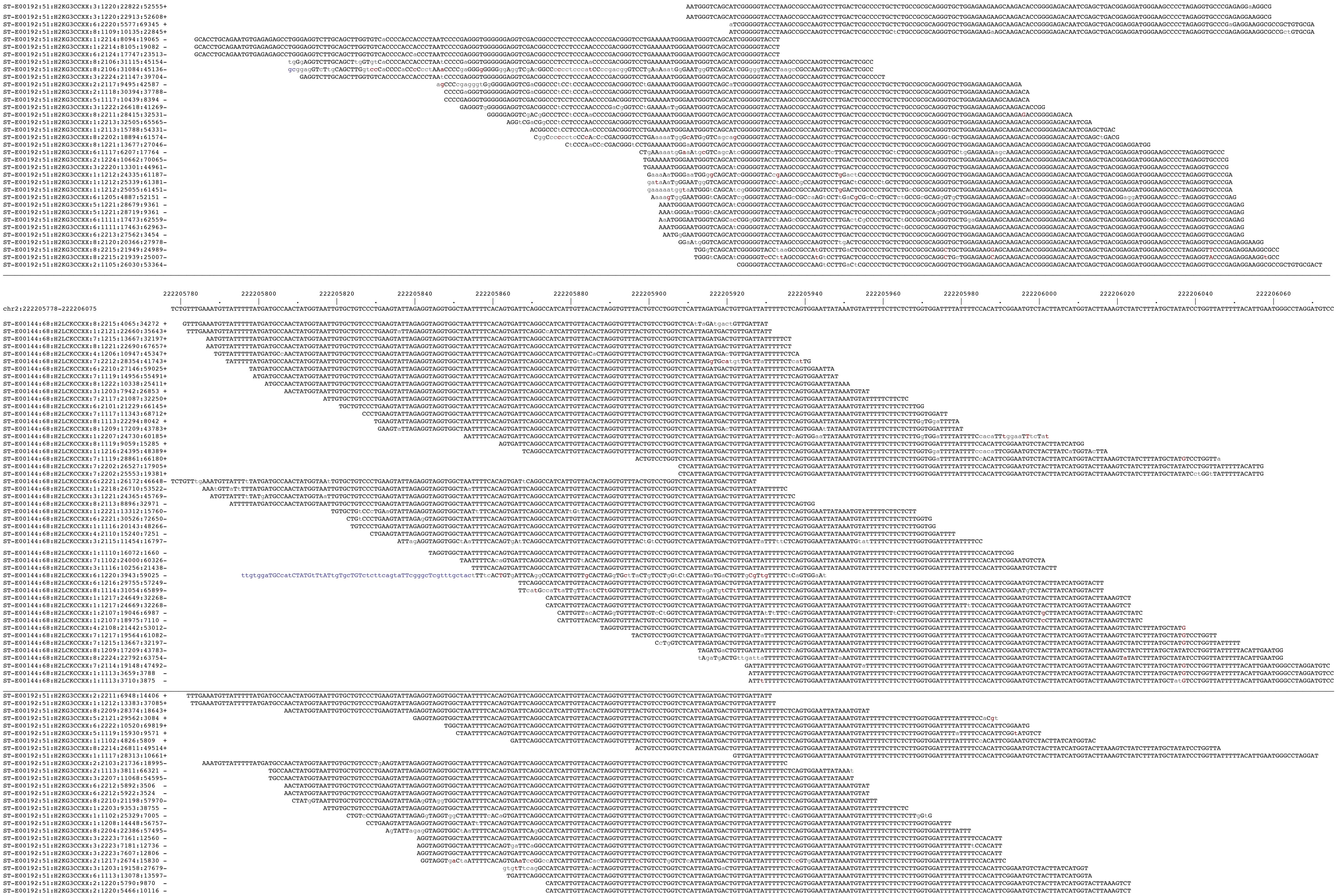
222205780
222205800
222205820
222205840
222205860
222205880
222205900
222205920
222205940
222205960
222205980
222206000
222206020
222206040
222206060
chr2:222205778-222206075

```
ST-E00192:51:H2KG3CCXX:5:1201:6430:20120 -   CTAGGTGTTTACTGTCCTgGTCTCATTAgATgACTGTTGATTATTTTTCTCAGTGGAATTATAAATGTATTTTTCTTCTCTTGGTGGATTTTATTTTCCACATTCGGAATGTCTACTTATCATGGTACTTAAAGTCTATCTTTATGCTAT
ST-E00192:51:H2KG3CCXX:8:1117:2827:69995 -   GGTGTTTACTGTCCTGGTCTCATTAGATGACTGTTGATTATTTTTCTCAGTGGAATTATAAATGTATTTTTCTTCTCTTGGTGGATTTTATTTTCCACATTCGGAATGTCTACTTATCATGGTACTTAAAGTCTATCTTTATGCTATATC
ST-E00192:51:H2KG3CCXX:2:1105:16742:15197-   TTAtTGTCCTgGTCTCATTAGATGACTGTTgAtTAtTTtTCTCAGTGGAATTATAAATGTATTTTTCTTCTCTTGGTGGATTTTaTTTTCCACATTCGGAATGTCTACTTATCATGGTaCTTAAAGTCTATCTTTATGCTATGTCCTGGT
ST-E00192:51:H2KG3CCXX:2:2107:12936:61135-   GTTGATTATTTTTCTCAGTGGAATTATAAATGTATTTTTCTTCTCTTGGTGGATTTTATTTTCCACATTCGGAATGTCTACTTATCATGGTACTTAAAGTCTATCTTTATGCTATATCCTGGTTATTTTTTCATTGAATGGGCCTAGGAT
```

---

chr3 105628400 - 2 chr17 18024298 + 3 CTX 44 72 75 75 0.956666666666667 0 0.951111111111111 0 1 chr3 105628474 150 chr17 18024372

ATACACAATGAAGTACTATTCAGTCATAAAAAAGAATGAGATCTAGTCATTTGCAACAACATGGACGGAACTGGACCCGGAGGTGCCCCCCGACCTACTAGCCTTCCCAGGGCCCCGACCCTCGTTCAGGGGCTCCCGCCGGAGAGGGGC

```
                          105628260  105628280  105628300  105628320  105628340  105628360  105628380  105628400  105628420  105628440  105628460  105628480  105628500  105628520  105628540
chr3:105628256-105628549  TGGAAGTTGACATGGATCATATCTGAAGGCTCCAATAGTATCAATGCAGTTGGAATCAAGgtttgatttttaactcccaaaaataagtgagaacatgtcatctctcttttctgtgcctgacttatttcacataacataatgatctccagttccgtccatgttgttgcaaatgactagatctcattctttttttatgactgaatagtacttcattgtgtatatgtaccacattttctttatccattcatctgtttatggactcttaggctgcttccaaaCATAATTTGTCTTTAAA

ST-E00144:68:H2LCKCCXX:1:1105:14215:30299+  GGAAGTTGACATGGATCATATCTGAAGGCTCCAATAGTATCAATGCAGTTGGAATCAAGGTTTGATTTTTAACTCCCAAAAATAAGTGAGAACATGTCATCTCTCTTTTCTGTGCCTGACTTATTTCACATAACATAATGATCTCCAGTT
ST-E00144:68:H2LCKCCXX:8:1119:30414:24954+  ATGGATCATATCTGAAGGCTCCAATAGTATCAATGCAGTTGGAATCAAGGTTTGATTTTTAACTCCCAAAAATAAGTGAGAACATGTCATCTCTCTTTTCTGTGCCTGACTTATTTCACATAACATAATGATCTCCAGTTCCGTCcatGT
ST-E00144:68:H2LCKCCXX:1:1207:13545:39370+  CATATCTGAAGGCTCCAATAGTATCAATGCAGTTGGAATCAAGGTTTGATTTTTAACTCCCAAAAATAAGTGAGAACATGTCATCTCTCTTTTCTGTGCCTGACTTATTTCACATAACATAATGATCTCCAGTTCCGTCCATGTTGTTGC
ST-E00144:68:H2LCKCCXX:8:2206:18326:25288+  TCCAATAGTATCAATGCAGTTGGAATCAAGGTTTGATTTTTAACTCCCAAAAATAAGTGAGAACATGTCATCTCTCTTTTCTGTGCCTGACTTATTTCACATAACATAATGATCTCCAGTTCCGTCCATGTTGTTGCAAATGACTAGATC
ST-E00144:68:H2LCKCCXX:4:2123:16692:49145+  CAATAGTATCAATGCAGTTGGAATCAAGGTTTGATTTTTAACTCCCAAAAATAAGTGAGAACATGTCATCTCTCTTTTCTGTGCCTGACTTATTTCACATAACATAATGATCTCCAGTTCCGTCCATGTTGTTGCAAATGACTAGATCTC
ST-E00144:68:H2LCKCCXX:8:2101:30435:69679+  AATAGTATCAATGCAGTTGGAATCAAGGTTTGATTTTTAACTCCCAAAAATAAGTGAGAACATGTCATCTCTCTTTTCTGTGCCTGACTTATTTCACATAACATAATGATCTCCAGTTCCGTCCaTGTTGTTGCAAATGACTAGATCTCA
ST-E00144:68:H2LCKCCXX:7:2221:9455:32637 +  ATGCAGTTGGAATCAAGGTTTGATTTTTAACTCCCAAAAATAAGTGAGAACATGTCATCTCTCTTTTCTGTGCCTGACTTATTTCACATAACATAATGATCTCCAGTTCCGTCCATGTTGTTGCAAATGACTAGATCTCATTCTTTTTTTA
ST-E00144:68:H2LCKCCXX:3:2205:24527:18801+  GTTGGAATCAAGGTTTGATTTTTAACTCCCAAAAATAAGTGAGAACATGTCATCTCTCTTTTCTGTGCCTGACTTATTTCACATAACATAATGATCTCCAGTTCCGTCCATGTTGTTGCAAATGACTAGATCTCATTCTTTTTTTATGACT
ST-E00144:68:H2LCKCCXX:1:2113:5851:54911 +  GGAATCAAGGTTTGATTTTTAACTCCCaaAAatcAGTGAGAAcATGTCatCTCTCTTTTCTGTGCCTGACTTATTTCACATAACATAATGATCTCCCGTtcCTtCcaTGTtGTTGCAcAgGACTAGATCTCATTCTTTTtTtATGACTGAA

ST-E00144:68:H2LCKCCXX:8:2106:24487:13703+  AGGTTTGATTTTTAACTCCCAAAAATAAGTGAGAACATGTCATCTCTCTTTTCTGTGCCTGACTTATTTCACATAACATAATGATCTCCAGTTCCGTCCATGTTGTTGCAAATGACTAGATCTCATTcTTTTTTATGACTGAATAGTACT
ST-E00144:68:H2LCKCCXX:4:1214:30861:15812+  TTTGATTTTTAACTCCCAAAAATAAGTGAGAACATGTCATCTCTCTTTTCTGTGCCTGACTTATTTCACATAACATAATGATCTCCAGTTCCGTCCATGTTGTTGCAAATGACTAGATCTCATTCTTTTTTTATGACTGAATAGTACTTCAT
ST-E00144:68:H2LCKCCXX:4:1124:7323:62664 +  CCCAAAAATAAGTGAGAACATGTCATCTCTCTTTTCTGTGCCTGACTTATTTCACATAACATAATGATCTCCAGTTCCGTCCATGTTGTTGCAAATGACTAGATCTCATTCTTTTTTTATGACTGAATAGTACTTCATTGTGTATATGTACc
ST-E00144:68:H2LCKCCXX:3:2121:19655:61697+  AATAAGTGAGAACATGTCATCTCTCTTTTCTGTGCCTGACTTATTTCACATAACATAATGATCTCCAGTTCCGTCCATGTTGTTGCAAATGACTAGATCTCATTCTTTTTTTATGACTGAATAGTACTTCATTGTGTATATGTACCACATT
ST-E00144:68:H2LCKCCXX:4:1215:8105:11910 +  AGTGAGAACATGTCATCTCTCTTTTCTGTGCCTGACTTATTTCACATAACATAATGATCTCCAGTTCCGTCCATGTTGTTGCAAATGACTAGATCTCATTCTTTTTTTaTGACTGAATAGTACTTCATTGTGTATATGTaCCACATTTTCTT
ST-E00144:68:H2LCKCCXX:3:1102:16387:10890+  GAACATGTCATCTCTCTTTTCTGTGCCTGACTTATTTCACATAACATAATGATCTCCAGTTCCGTCCATGTTGTTGCAAATGACTAGATCTCATTCTTTTTTTATGACTGAATAGTACTTCATTGTGTATATGTACCACATTTTCTTTATCC
ST-E00144:68:H2LCKCCXX:3:2204:32566:9167 +  GAACATGTCATCTCTCTTTTCTGTGCCTGACTTATTTCACATAACATAATGATCTCCAGTTCCGTCCATGTTGTTGCAAAtGACTAGATCTCATTCTTTTTTTATGACTGAAtAGTAttTCAtTgtgTATATGTacCACctTTtctTTtTgC
ST-E00144:68:H2LCKCCXX:8:2116:13251:72263+  CATGTCATCTCTCTTTTCTGTGCCTGACTTATTTCACATAACATAATGATCTCCAGTTCCGTCCATGTTGTTGCAAATGACTAGATCTCATTCTTTTTTTATGACTGAATAGTACTTCATTGTGTATATGTACCACATTTTCTTTATCCaTT
ST-E00144:68:H2LCKCCXX:8:2207:5263:33217 +  CTCTTTTCTGTGCCTGACTTATTTCACATAACATAATGATCTCCAGTTCCGTCCATGTTGTTGCAAATGACTAGATCTCATTCTTTTTTTATGACTGAATAGTACTTCATTGTGTATATGTACCACATTTTCTTTATCCATTCATCTGTTTA
ST-E00144:68:H2LCKCCXX:1:2119:23989:5282 +  TGCCTGACTTATTTCACATAACATAATGATCTCCAGTTCCGTCCATGTTGTTGCAAATGACTAGATCTCATTCTTTTTTTATGACTGAATAGTACTTCATTGTGTATATGTACCACATTTTCTTTATCCATTCATCTGTTTATGGAcTcTT
ST-E00144:68:H2LCKCCXX:3:1121:23939:46543+  CTCCAGTTCCGTCCATGTTGTTGCAAATGACTAGATCTCATTCTTTTTTTATGACTGAATAGTACTTCATTGTGTATATGTACCACATTTTCTTTATCCATTCATCTGTTTATGGACTCTTAGGCTGCTTCCAAACATAATTTGTCTTTAA
ST-E00144:68:H2LCKCCXX:8:1224:22244:63438-  TGGAAGTTGACATGGATCATATCTGAAGGCTCCAATAGTATCAATGCAGTTGGAATCAAGGTTTGATTTTTAACTCCCAAAAATAAGTGAGAACATGTCATCTCTCTTTTCTGTGCCTGACTTATTTCACATAACATAATGATCTCCAGT
ST-E00144:68:H2LCKCCXX:1:1112:22132:10433-  ACATGGATCATATCTGAAGGCTCCAATAGTATCAATGCAGTTGGAATCAAGGTTTGATTTTTAACTCCCAAAAATAAGTGAGAACATGTCATCTCTCTTTTCTGTGCCTGACTTATTTCACATAACATAATGATCTCCAGTTCCGTCCAT
ST-E00144:68:H2LCKCCXX:4:2111:32343:24603-  GaAcGCTCCAATAGTATCAaTGcagtTgGAAtcaaGGTTTGATTTTTAACtCCCAAAAATAAGTGAGAACATGTCATCTCTCTTTTCTGTGCCTGACTTAtTtCACATAACATAATGATCTCCAGTTCCGtCCATGTTGTTGCAAATGAC
ST-E00144:68:H2LCKCCXX:8:1223:22162:66989-  ATAGTATCAATGCAGTTgGAATCAAggTTTGATTTTTAACTCCCAAAAATAAGTGAGAACATGTCATCTCTCtTTCTGTGCCTGACTTATtgaCACATAACATAATGATCTCCAGTTCCGTCCATGTTGTTGCAAATGACTAGATCTCAT
ST-E00144:68:H2LCKCCXX:3:1207:14936:44310-  GTATCAATGCAGTTGGAATCAAGGTTTGATTTTTAACTCCCAAAAATAAGTGAGAACATGTCATCTCTCTTTTCTGTGCCTGACTTATTTCACATAACATAATGATCTCCAGTTCCGTCCATGTTGTTGCAAATGACTAGATCTCATTCT
ST-E00144:68:H2LCKCCXX:1:1101:16621:37594-  TTGATTTTTAACTCCCAAAAACAAGTGAGAACATGTCATCTCTCTTTTCTGTGCCTGACTTATTTCACATAACATAATGATCTCCAGTTCCGTCCATGTTGTTGCAAATGACTAGATCTCATTCTTTTTTTATGACTGAATAGTACTTCATT
ST-E00144:68:H2LCKCCXX:4:1103:10957:40443-  TTGATTTTTAACTCcCAAAAATAAGTGAGAACATGTCATCTCTCTTTTCTGTGCCTGACTTATTTCACATAACATAATGATCTCCAGTTCCGTCCATGTTGTTGCAAATGACTAGATCTCATTCTTTTTTTATGACTGAATAGTACTTCATT
ST-E00144:68:H2LCKCCXX:8:1113:4380:37067 -  TTGATTTTTAACTCCCAAAAATAAGTGAGAACATGTCATCTCTCTTTTCTGTGCCTGACTTATTTCACATAACATAATGATCTCCAGTTCCGTCCATGTTGTTGCAAATGACTAGATCTCATTCTTTTTTTATGACTGAATAGTACTTCATT
ST-E00144:68:H2LCKCCXX:8:2109:8541:12138 -  ATAAGTGAGAACATGTCATCTCTCTTTTCTGTGCCTGACTTATTTCACATAACATAATGATCTCCAGTTCCGTCCATGTTGTTGCAAATGACTAGATCTCATTCTTTTTTTATGACTGAATAGTACTTCATTGTGTATATGTACCACATTTT
ST-E00144:68:H2LCKCCXX:3:2102:14905:71753-  aGtGagaaCatgtcATCTCTCTTTTCTGtGCCTGaTTATTTCACATAACAtAATGATCTCCAGTTCCGTCCATGTTGTTGCAAATGACTAGATCTCATTCTTTTTTTATGACTGAATAGTACTTCATTGTGTATATGTACCACATTTTCTT
ST-E00144:68:H2LCKCCXX:3:2209:31328:7268 -  ctgtgcctCtcTcTTTTtctcgCTGACTTATTTCACAtaacataaTgaTctccAGTTCCGTcCATGTTGTTGCAAATGAcTAGATCTCATTCTTTTTTTATGACTGAATAGTACTTCATTGTGTATATGTACCACATTTTCTTTATCCATT
ST-E00144:68:H2LCKCCXX:4:2218:28669:48406-  CaTCTCTCTTTTCTGTGcCTgACTTATtTCACATAACATAATGATCTCCAGTTCCGTCCATGTTGTTGCAAATGACTAGATCTCATTCTTTTTTTATGACTGAATAGTACTTCATTGTGTATATGTACCACATTTTCTTTATCCATTCATCT
ST-E00144:68:H2LCKCCXX:1:1105:14215:30299-  TCTCTTTTCTGTGCCTGACTTATTTCACATAACATAATGATCTCCAGTTCCGTCCATGTTGTTGCAAATGACTAGATCTCATTCTTTTTTTATGACTGAATAGTACTTCATTGTGTATATGTACCACATTTTCTTTATCCATTCATCTGTTT
ST-E00144:68:H2LCKCCXX:8:2224:26172:27486-  TCTCTTTTCTGTGCCTGACTTATTTCACATAACATAATGATCTCCAGTTCCGTCCATGTTGTTGCAAATGACTAGATCTCATTCTTTTTTTATGACTGAATAGTACTTCATTGTGTATATGTACCACATTTTCTTTATCCATTCATCTGTTT
ST-E00144:68:H2LCKCCXX:6:2106:29602:71085-  CTCTTTTCTGTGCCTGACTTATTTCACATAACATAATGATCTCCAGTTCCGTCCATGTTGTTGCAAATGACTAGATCTCATTCTTTTTTTATGACTGAATAGTACTTCATTGTGTATATGTACCACATTTTCTTTATCCATTCATCTGTTTA
ST-E00144:68:H2LCKCCXX:7:2124:15920:10820-  CTGACTTATTTCACATAACATAATGATCTCCAGTTCCGTCCATGTTGTTGCAAATGACTAGATCTCATTCTTTTTTTATGACTGAATAGTACTTCATTGTGTATATGTACCACATTTTCTTTATCCATTCATCTGTTTATGGACTCTTAGG
ST-E00144:68:H2LCKCCXX:3:2224:24294:51483-  GACTTATTTCACATAACATAATGATCTCCAGTTCCGTCCATGTTGTTGCAAATGACTAGATCTCATTCTTTTTTTATGACTGAATAGTACTTCATTGTGTATATGTACCACATTTTCTTTATCCATTCATCTGTTTATGGACTCTTAGGCT
ST-E00144:68:H2LCKCCXX:1:2217:22173:40108-  TTATTTCACATAACATAATGATCTCCAGTTCCGTCCATGTTGTTGCAAATGACTAGATCTCATTCTTTTTTTATGACTGAATAGTACTTCATTGTGTATATGTACCACATTTTCTTTATCCATTCATCTGTTTATGGACTCTTAGGCTGCT
ST-E00144:68:H2LCKCCXX:1:2210:20031:7392 -  TTTCACATAACATAATGATCTCCAGTTCCGTCCATGTTGTTGCAAATGACTAGATCTCATTCTTTTTTTATGACTGAATAGTACTTCATTGTGTATATGTACCACATTTTCTTTATCCATTCATCTGTTTATGGACTCTTAGGCTGCTTCC
ST-E00144:68:H2LCKCCXX:1:2121:14185:33129-  TAACATAATGATCTCCAGTTCCGTCCATGTTGTTGCAAATGACTAGATCTCATTCTTTTTTTATGACTGAATAGTACTTCATTGTGTATATGTACCACATTTTCTTTATCCATTCATCTGTTTATGGACTCTTAGGCTGCTTCCAAaCATA
ST-E00144:68:H2LCKCCXX:1:1118:25400:2627 -  ATAATGATCTCCAGTTCCGTCCATGTTGTTGCAAATGACTAGATCTCATTCTTTTTTTATGACTGAATAGTACTTCATTGTGTATATGTACCACATTTTCTTTATCCATTCATCTGTTTATGGACTCTTAGGCTGCTTCCAAACATAATTT
ST-E00144:68:H2LCKCCXX:1:2214:21462:67252-  ATGATCTCCAGTTCCGTCCATGTTGTTGCAAATGACTAGATCTCATTCTTTTTTTATGACTGAATAGTACTTCATTGTGTATATGTACCACATTTTCTTTATCCATTCATCTGTTTATGGACTCTTAGGCTGCTTCCAAACATAATTTGTC
ST-E00144:68:H2LCKCCXX:4:2111:8013:4860  -  TCCAGTTCCGTCCATGTTGTTGCAAATGACTAGATCTCATTCTTTTTTTATGACTGAATAGTACTTCATTGTGTATATGTACCACATTTTCTTTATCCATTCATCTGTTTATGGACTCTTAGGCTGCTTCCAAACATAATTTGTCTTTAAA
ST-E00144:68:H2LCKCCXX:7:2203:13109:52643-  GCCCCTCTCcGGCGGGAGCCCCTgaacGAGGgtCGGGGCCCTGGgAAGGCTAGTAGGTcGGGGGGCACCTCCGGgTCCAGTTCCGTCCATGTTGTTGCAAATGACTAGATCTCATTCTTTTTTTATGACTGAATAGTACTTCATTGTGTAT
ST-E00144:68:H2LCKCCXX:7:2203:13271:52678-  GCCCCtCtCCGGCGGGAGCCCCTGAACGAGGGTCGGGCCCTGGGAAGGCTAGTAGGTCGGGGGGCACCTCCGGGTCCAGTTCCGTCCATGTTGTTGCAAATGACTAGATCTCATTCTTTTTTTATGACTGAATAGTACTTCATTGTGTAT
ST-E00144:68:H2LCKCCXX:8:1119:30414:24954-  TCCAGTTCCGTCCATGTTGTTGCAAATGACTAGATCTCATTCTTTTTTTATGACTGAATAGTACTTCATTGTGTATATGTACCACATTTTCTTTATCCATTCATCTGTTTATGGACTCTTAGGCTGCTTCCAAACATAATTTGTCTTTAAA
```

```
ST-E00192:51:H2KG3CCXX:8:1203:28902:59429+  TCATATCTGAAGGCTCCAATAGTATCAATGCAGTTGGAATCAAGGTTTGATTTTTAACTCCCAAAAATAAGTGAGAACATGTCATCTCTCTTTTCTGTGCCTGACTTATTTCACATAACATAATGATCTCCAGTTCCGtCCATGTTGTTG
ST-E00192:51:H2KG3CCXX:8:1104:24751:30791+  CATATCTGAAGGCTCCAATAGTATCAATGCAGTTGGAATCAAGGTTTGATTTTTAACTCCCAAAAATAAGTGAGAACATGTCATCTCTCTTTTCTGTGCCTGACTTATTTCACATAACATAATGATCTCCAGTTCCGTCCATGTTGTTGC
ST-E00192:51:H2KG3CCXX:8:1222:29135:14265+  GAAGGCTCCAATAGTATCAATGCAGTTGGAATCAAGGTTTGATTTTTAACTCCCAAAAATAAGTGAGAACATGTCATCTCTCTTTTCTGTGCCTGACTTATTTCACATAACATAATGATCTCCAGTTCCGTCCATGTtgTTGCAAATGAC
ST-E00192:51:H2KG3CCXX:8:2103:12002:25236+  AATAGTATCAATGCAGTTGGAATCAAGGTTTGATTTTTAACTCCCAAAAATAAGTGAGAACATGTCATCTCTCTTTTCTGTGCCTGACTTATTTCACATAACATAATGATCTCCAGTTCCGTCCATGTTGTTGCAAATGACTAGATCTCA
ST-E00192:51:H2KG3CCXX:8:2220:4065:2627  +  AGTATCAATGCAGTTGGAATCAAGGTTTGATTTTTAACTCCCAAAAATAAGTGAGAACATGTCATCTCTCTTTTCTGTGCCTGACTTATTTCACATAACATAATGATCTCCAGTTCCGTCCATGTTGTTGCAAATGACTAGATCTCATTC
ST-E00192:51:H2KG3CCXX:5:2124:6531:41673 +  CAAGGTTTGATTTTTAACTCCCAAAAATAAGTGAGAACATGTCATCTCTCTTTTCTGTGCCTGACTTATTTCACATAACATAATGATCTCCAgTTCcgTCCATGTTGTTGCAAATGACTAGAtctCATTCTTTtTtTTATGACtgAaTAGTA
ST-E00192:51:H2KG3CCXX:5:1220:23878:62752+  GGTTTGATTTTTAACTCCCAAAAATAAGTGAGAACATGTCATCTCTCTTTTCTGTGCCTGACTTATTTCACATAACATAATGATCTCCAGTTCCGTCCATGTTGTTGCAAATGACTAGATCTCATTCTTTTTTTATGACTGAATAGTACTT
ST-E00192:51:H2KG3CCXX:6:1112:25999:54261+  TTGATTTTTAACTCCCAAAAATAAGTGAGAACATGTCATCTCTCTTTTCTGTGCCTGACTTATTTCACATAACATAATGATCTCCAGTTCCGTCCATGTTGTTGCAAATGACTAGATCTCATTCTTTTTTTATGACTGAATAGTACTTCATT
ST-E00192:51:H2KG3CCXX:5:2123:19229:11523+  TAAGTGAGAACATGTCATCTCTCTTTTCTGTGCCTGACTTATTTCACATAACATAATGATCTCCAGTTCCGTCCATGTTGTTGCAAATGACTAGATCTCATTCTTTTTTTATGACTGAATAGTACTTCATTGTGTATATGTACCACATTTTC
ST-E00192:51:H2KG3CCXX:8:2208:5242:11066 +  ATGTCATCTCTCTTTTCTGTGCCTGACTTATTTCACATAACATAATGATCTCCAGTTCCGTCCATGTTGTTGCAAATGACTAGATCTCATTCTTTTTTTATGACTGAATAGTACTTCATTGTGTATATGTACCACATTTTCTTTATCCATTC
ST-E00192:51:H2KG3CCXX:2:2103:32059:27415+  CTCTCTTTTCTGTGCCTGACTTATTTCACATAACATAATGATCTCCAGTTCCGTCCATGTTGTTGCAAATGACTAGATCTCATTCTTTTTTTATGACTGAATAGTACTTCATTGTGTATATGTAcTacaTTTTtTTATtaTTcaTttGTT
ST-E00192:51:H2KG3CCXX:2:2103:32018:27451+  CTCTCTTTTCTGTGCCTGACTTATTTCACATAACATAATGATCTCCAGTTCCGTCCATGTTGTTGCAAATGACTAGATCTCATTCTTTTTTTATGACTGAATAGTACTTCATTGTGTATATGTAacaaATTTTtTTTATttATTaATaTGTT
ST-E00192:51:H2KG3CCXX:2:2220:13677:51905+  GTGCCTGACTTATTTCACATAACATAATGATCTCCAGTTCCGTCCATGTTGTTGCAAATGACTAGATCTCATTCTTTTTTTATGACTGAATAGTACTTCATTGTGTATATGTACCACATTTTCTTTATCCATTCATCTGTTTATGGACTcT
ST-E00192:51:H2KG3CCXX:2:2220:13687:51922+  GTGCCTGACTTATTTCACATAACATAATGATCTCCAGTTCCGTCCATGTTGTTGCAAATGACTAGATCTCATTCTTTTTTTATGACTGAATAGTACTTCATTGTGTATATGTACCACATTTTCTTTATCCATTCATCTGTTTATGGACTCT
ST-E00192:51:H2KG3CCXX:1:1202:25735:35960+  ACTTATTTCACATAACATAATGATCTCCAGTTCCGTCCATGTTGTTGCAAATGACTAGATCTCATTCTTTTTTTATGACTGAATAGTACTTCATTGTGTATATGTACCACATTTTCTTTATCcATTCATCTGTTTATGGACTcTTAGgcTg
ST-E00192:51:H2KG3CCXX:6:1106:22761:7268 -  AAGTTGACATGGATCATATCTGAAGGCTCCAATAGTATCAATGCAGTTGGAATCAAGGTTTGATTTTTAACTCCCAAAAATAAGTGAGAACATGTCATCTCTCTTTTCTGTGCCTGACTTATTTCACATAACATAATGATCTCCAGTTCC
ST-E00192:51:H2KG3CCXX:1:2114:26578:67991-  GATCATATCTGAAGGCTCCAATAGTATCAATGCAGTTGGAATCAAGGTTTGATTTTTAACTCCCAAAAATAAGTGAGAACATGTCATCTCTCTTTTCTGTGCCTGACTTATTTCACATAACATAATGATCTCCAGTTCCGTCCATGTTGT
ST-E00192:51:H2KG3CCXX:6:1110:4978:54454 -  TCTGAAGGCTCCAATAGTATCAATGCAGTTGGAATCAAGGTTTGATTTTTAACTCCCAAAAATAAGTGAGAACATGTCATCTCTCTTTTCTGTGCCTGACTTATTTCACATAACATAATGATCTCCAGTTCCGTCCATGTTGTTGCAAAT
ST-E00192:51:H2KG3CCXX:1:1112:18600:56775-  GTATCAATGCAgTTGGAATCAAGGTTTGATTTTTAACTCCCAAAAATAAGTGAGAACATGTCATCTCTCTTTTCTGTGCCTGACTTATTTCACATAACATAATGATCTCCAGTtCCGTCCATGTTGTTGCAAATGACTAGATCTCATTCT
ST-E00192:51:H2KG3CCXX:1:1112:18590:56792-  GTATCAATGCAGTTGGAATCAAGGTTTGATTTTTAACTCCCAAAAATAAGTGAGAACATGTCATCTCTCTTTTCTGTGCCTGACTTATTTCACATAACATAATGATCTCCAGTTCCGTCCATGTTGTTGCAAATGACTAGATCTCATTCT
ST-E00192:51:H2KG3CCXX:1:1112:18610:56792-  GTATCAATGCAGTTGGAATCAAGGTTTGATTTTTAACTCCCAAAAATAAGTGAGAACATGTCATCTCTCTTTTCTGTGCCTGACTTATTTCACATAACATAATGATCTCCAGTTCCGTCCATGTTGTTGCAAATGACTAGATCTCATTCT
ST-E00192:51:H2KG3CCXX:2:1209:17767:9308 -  TCTCTCTTTTCTGTGCCTGACTTATTTCACATAACATAATGATCTCCAGTTCCGTCCATGTTGTTGCAAATGACTAGATCTCATTCTTTTTTTATGACTGAATAGTACTTCATTGTGTATATGTACCACATTTTCTTTATCCATTCATCTGT
ST-E00192:51:H2KG3CCXX:8:1222:29135:14265-  TGCCTGACTTATTTCACATAACATAATGATCTCCAGTTCCGTCCATGTTGTTGCAAATGACTAGATCTCATTCTTTTTTTATGACTGAATAGTACTTCATTGTGTATATGTACCACATTTTCTTTATCCATTCATCTGTTTATGGACTCTT
ST-E00192:51:H2KG3CCXX:8:2103:12002:25236-  TGACTTATTTCACATAACATAATGATCTCCAGTTCCGTCCATGTTGTTGCAAATGACTAGATCTCATTCTTTTTTTATGACTGAATAGTACTTCATTGTGTATATGTACCACATTTTCTTTATCCATTCATCTGTTTATGGACTCTTAGGC
ST-E00192:51:H2KG3CCXX:8:1110:3598:69432 -  GACTTATTTCACATAACATAATGATCTCCAGTTCCGTCCATGTTGTTGCAAATGACTAGATCTCATTCTTTTTTTATGACTGAATAGTACTTCATTGTGTATATGTACCACATTTTCTTTATCCATTCATCTGTTTATGGACTCTTAGGCT
ST-E00192:51:H2KG3CCXX:1:1224:21959:7286 -  TTATTTCACATAACATAATGATCTCCAGTTCCGTCCATGTTGTTGCAAATGACTAGATCTCATTCTTTTTTTATGACTGAATAGTACTTCATTGTGTATATGTACCACATTTTCTTTATCCATTCATCTGTTTATGGACTCTTAGGCTGCT
ST-E00192:51:H2KG3CCXX:8:1203:32059:70944-  ACATAATGATCTCCAGTTCCGTCCATGTTGTTGCAAATGACTAGATCTCATTCTTTTTTTATGACTGAATAGTACTTCATTGTGTATATGTACCACATTTTCTTTATCCATTCATCTGTTTATGGACTCTTAGGCTGCTTCCAAACATAAT
ST-E00192:51:H2KG3CCXX:8:2102:5242:54454 -  AAtgaTCTCCAGTTCCGTCCATGTTGTTGCAAAtGACTAGATCTCATTCTTTTTTTATGACTGAATAGTACTTCATTGTGTATATGTACCACATTTTCTTTATCCATTCATCTGTTTATGGACTCTTAGGCTGCTTCCAAACATAATTTGT
ST-E00192:51:H2KG3CCXX:8:2102:5232:54507 -  AATGATCTCCAGTTCCgTCCATGTTGTTGCAAATGACTAGATCTCATTCTTTTTTTATGACTGAATAGTACTTCATTGTGTATATGTACCACATTTTCTTTATCCATTCATCTGTTTATGGACTCTTAGGCTGCTTCCAAACATAATTTGT
ST-E00192:51:H2KG3CCXX:1:1221:25857:63191-  ATGATCTCCAGTTCCGTCCATGTTGTTGCAAATGACTAGATCTCATTCTTTTTTTATGACTGAATAGTACTTCATTGTGTATATGTACCACATTTTCTTTATCCATTCATCTGTTTATGGACTCTTAGGCTGCTTCCAAACATAATTTGTC
ST-E00192:51:H2KG3CCXX:8:1206:18042:70470-  ATGATCTCCAGTTCCGTCCATGTTGTTGCAAATGACTAGATCTCATTCTTTTTTTATGACTGAATAGTACTTCATTGTGTATATGTACCACATTTTCTTTATCCATTCATCTGTTTATGGACTCTTAGGCTGCTTCCAAACATAATTTGTC
ST-E00192:51:H2KG3CCXX:3:2108:32749:25446-  TgctCtgcagTtCCGTCCATGTTgtTGCtAATGACTAGATCTCATTCTTcTTtTATGACTGAAtAGTaCTTCATTGTGTATATGTACCACATTTTCTTTATCCATTCATCTGTTTATGGcTCTTAGGCTGCTTCCAAaCatAATTTGtC
ST-E00192:51:H2KG3CCXX:8:2201:29481:50622-  cTCTCCAGtTCCGTcCATGTTgTtgCAAATGACTAgATCTCATTCTTTTTTTATGACTGAATAGTACTTCATTGTGTATATGTACCACATTTTCTTTATCCATTCATCTGTTTATGGACTCTTAGGCTGCTTCCAAACATAATTTGTCTTT
```

```
                          18024160  18024180  18024200  18024220  18024240  18024260  18024280  18024300  18024320  18024340  18024360  18024380  18024400  18024420  18024440
chr17:18024149-18024443   ccgctctccccggcgctctcgggcctgccccggccggcctcgccctacggctccctccgcgccaccccgccgccTGGGCCGCCCCAGCGCACGTGCCACCGGCGCCGCAGGCCAGCTGGTGGGCCTTCGTGGAGCCCCCTGCCGTGAGCCCGGAGGTGCCCCCGACCTACTAGCCTTCCCAGGGCCCCGACCCTCGTTCAGGGGCTCCCGCCGGAGAGGGGCGGCTTTCGGCTTCCCCGGGGCCTCTCCACGGGCGTCGCGGAGGCGAGCTTGGTCACCGCTGGCCTCGCCCC

ST-E00144:68:H2LCKCCXX:6:1219:12530:39933+  CCCGGCGCTCTCGGGCCTGCCCCGGCCGGCCTCGCCCTACGGCTCCCTCCGCGCCACCCCGCCGCCTGGGCCGCCCCAGCGCACGTGCCACCGGCGCCGCAGGCCAGCTGGTGGGCCTTCGTGGAGCCCCCTGCCGTGAGCCCGGAGGT
ST-E00144:68:H2LCKCCXX:6:2224:17585:18168+  CCCGGCGCTCTCGGGCCTGCCCCGGCCGGCCTCGCCCTACGGCTCCCTCCGCGCCACCCCGCCGCCTGgGCCGCCCCAGCGCACGTgCCACCgCGCCGCAGGCCAGCTGGtgGGCCTTCGtGGAGccCCTGCCGTGAGCCCGGaGGT
ST-E00144:68:H2LCKCCXX:8:2106:25806:29174+  CCGGCGCTCTCGGGCCTGCCCCGGCCGGCCTCGCCCTACGGCTCCCTCCGCGCCACCCCGCCGCCTGGGCCGCCCCAGCGCACGTGCCACCGGCGCCGCAGGCCAGCTGGTGGGCCTTCGTGGAGCCCCCTGCCGTGAGCCCGGaGGtG
ST-E00144:68:H2LCKCCXX:8:1121:32241:18907+  GGCGCTCTCGGGCCTGCCCCGGCCGGCCTCGCCCTACGGCTCCCTCCGCGCCACCCCGCCGCCtGGGCCGCCCCAgCGcacgTgCCACCgGCGCCgCagGCCAGCGgGcgggCCtgCgTggaGCCcCCtGcCGcgaGCCCgGcggcgac
ST-E00144:68:H2LCKCCXX:4:1212:24801:35204+  GCCCCGGCCGGCCTCGCCCTACGGCTCCCTCCGCGCCACCCCGCCGCCCCcCAGCGCACGTgCCACCggCGCCgCagGCcAgCtGctGggcCTcCGcggagcCCCCcgCggcgCCCcgCgGgccccCCgccctacacc
ST-E00144:68:H2LCKCCXX:8:1109:17209:6723 +  CCGGCCGGCCTCGCCCTACGGCTCCCTCCGCGCCACCCCGCCGCCtGGGCCGCCCCAGCGCACGTGCCACCGGCGCCGCAgGCCaGCGGGTGGGCCTTCGTGGaGCCCCCtGCCGTGAGCCCGGAGGtgcCCCCCGaCCtACTAGCCtt
ST-E00144:68:H2LCKCCXX:1:2207:6450:63719 +  GGCCGGCCTCGCCCTACGGCTCCCTCCGCGCCACCCCGCCGCCTGGGCCGCCCCAGCGCACGTGCCACCGGCGCCGCAGGCCAgCTGGTGGGCCtTCGTGGcGcCCCCtGCCgTcagcCCggaGgtgCCCCcCgacCtcccagcccccc
ST-E00144:68:H2LCKCCXX:6:1110:11677:64369+  GCCGGCCTCGCCCTACgCTCCCTCCGCGCCCCCCgCCgCCCTGGgcCgcCcCAgCgCtcgtGCCaCCGGCGCCgCagGCCaGCgggTGGGCTgcGtggagcccCcgCCgcGggccCgGagGTgCCCCCCgaCCtACaagccttCCC
ST-E00144:68:H2LCKCCXX:4:2121:9942:47264 +  GGCCTCGCCCTACGGCTCCCTCCGCGCCACCCCGCCGCCTgggCCGCCCCAgCGCacgTGCCaCCGGCgCCGCAGGCCCGCtggtgggcCttcgtgGagccCCCTgCcccgagccCGGCGccgcccCCCCgaCCtaCtAcccttcCctgg
ST-E00144:68:H2LCKCCXX:4:2121:9932:47316 +  GGCCTCGCCCTACGGCTCCCTCCGCGCCCCCCGCCGCCCtGGGCCGCCCCAGCGCACGtGCCACCGGCGCCGCAgGCCAGCGGtGGgcCtTCGTGgagcccCCggccgtGcgCCggaggtGcCcCCCgaCCtactaGccttcaCAgg
ST-E00144:68:H2LCKCCXX:6:1221:11474:2944 +  CTCCCTACGGCTCCCTCCGCGCCACCCCGCCGCCCTGGGCCGCCCCAGCGCACGTGCCACCGGCGCCGCAGGCCAGCTGGtGgGCCTTCGTGGAGCCCCCTGCCGTGAGCCCGGAGGTGCCCCCCGACCTACtAGCCTTCCCAGGgC
ST-E00144:68:H2LCKCCXX:8:1122:21817:37559+  CCCTACGGCTCCCTCCGCGCCACCCCGCCGCCCTGGGCCGCCCCAGCGCACGTGCCACCGGCGCCGCAGGCCAGCtGGTGGGCCttCGtGGaGccCCCTGCCGtgcgCCGGAGGtgCCCCcCgacTcccccgcCttCCCAGgGCCcGc
ST-E00144:68:H2LCKCCXX:4:1106:15169:9062 +  ACGGCTCCCTCCGCGCCACCCCGCCGCCCTGGGCCGCCCCAGCGCACGTGCCACCGGCGCCGCAgGCCaGCGgGTgGCCtTCGtGGaGCcCCCtGCCGTgaGCCCGGaGGtGCCcCCCGaCCtACgagCCTtCCCAggGCCCCGcCCCt
ST-E00144:68:H2LCKCCXX:6:2221:4694:69257 +  GCTCCCTCCGCGCCACCCCGCCGCCCTGGGCCGCCCCAGCGCACGTGCCACCGGCGCCGCAGGCCAGCTGGTGGGCCTTCGTGGAGCCCCCTGCCGTGAGCCCGGAGGTGCCCCCCGACCTACTAGCCTTCCCAGGgaCCCgACCctCGT
ST-E00144:68:H2LCKCCXX:3:1107:18681:30439+  TCCCTCCGCGCCACCCCGCCGCCCTGGGCCgCCCCAGCGCACGTGCCACCGGCGCCGCAGGCcCcGcGGTggccCctCGTggaGCCCCCTGCCGTGAGCCCGgAGgtGCCCCCCgaCctaccaGCCttcCCaggGCCCcGaCcCtcgTgC
```

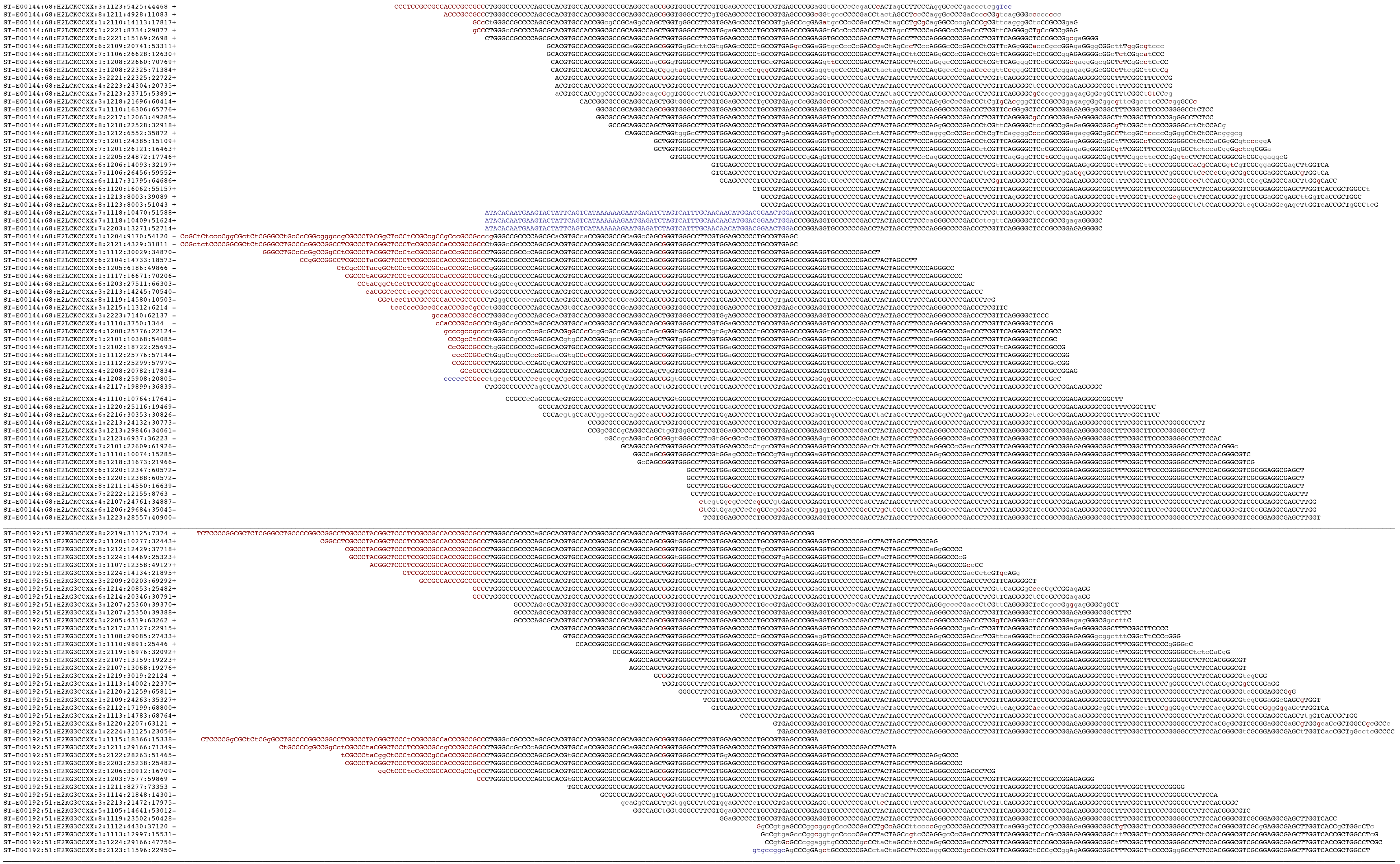

chr18 46048294 + 5 chr3 113001939 + 0 CTX 66 39 46 0 1 0 1 0 1 chr18 46048191 150 chr3 113001984

CCCTTCCCTTGTCCAGGGAAAATTGCAGTCGACACAAATACAGCCAATCCCAGGAGACAGTCATGCTGAAAGGGCCCTGTGTCCTAGAGGCTGCATATAATCCCAAAAGTTCACCAGTTCTTCCCAAAAGCACTACACCGCTTCTGCCGG

```
                    46048160  46048180  46048200  46048220  46048240  46048260  46048280  46048300  46048320  46048340  46048360  46048380  46048400  46048420  46048440
chr18:46048145-46048443    GGTACAATTCCCAGCTCTGAAAAGGCCGCACAGGGCCTGCTCTGCTCCCTTCCCTTGTCCAGGGAAAATTGCAGTCGACACAAATACAGCCAATCCCAGGAGACAGTCATGCTGAAAGGGCCCTGTGTCCTAGAGGCTGCATATAATCCCCAATTATCCCCCAGTTTCCCCAGGGAGAGAATGGAGAGGTGAGGGCTTTCAAAAGCCAGCATCCCTTCAGTCTCCATTATGCGGAGACTCACACAGCCTCCTTTCTCCAAACAGCACAGAAGGTCACAGGACACGGGGCAACAGAGGC
```

```
ST-E00144:68:H2LCKCCXX:3:1215:20721:69766+ GGTACAATTCCCAGCTCTGAAAAGGCCGCACAGGGCCTGATCTGCTCCCTTCCCTTGTCCAGGGAAAATTGCAGTCGACACAAATACAGCCAATCCCAGGAGaCAGTCaTGCTGAAAGGGCCCTGTGTCCTAGAGGCTGCATATAatCCC

ST-E00144:68:H2LCKCCXX:1:1117:4481:62699 + ACAATTCCCAGCTCTGAAAAGGCCGCACAGGGCCTGATCTGCTCCCTTCCCTTGTCCAGGGAAAATTGCAGTCGACACAAATACAgCCAATCCCAGGAGACAGTcATGcTGAAcGGGCcCTGTGTCctagaGgCtGCATATaATacccAa
ST-E00144:68:H2LCKCCXX:1:1117:4623:62770 + ACAATTCCCAGCTCTGAAAAGGCCGCACAGGGCCTGATCTGCTCCCTTCCCTTGTCCAGGGAAAATTGCAGTCGACACAAATACAGCCAATCCCAGGAGACAGTCATGCTGAAaGGGCcCTGTGTCCTAGAGgctGCATATAATCCCCAA
ST-E00144:68:H2LCKCCXX:1:1223:15352:50270+ CCAGCTCTGAAaAGGCCGCACAGGGCCTGATCTGCTCCCTTCCCTTGTCCAGGGAAaATTGCAGTCGACACAAATACAGCCAATCCCAGGAGACAGTCATGCTGAAAGGGCCCTGTGTCCTAGAGGCTGCATATAATCCCCAATTaTCCC
ST-E00144:68:H2LCKCCXX:8:1202:18539:14336+ CTGAAAAGGCCGCACAGGGCCTGATCTGCTCCCTTCCCTTGTCCAGGGAAAATTGCAGTCGACACAAATACAGCCAATCCCAGGAGACAGTCATGCTGAAAGGgCCCTGTGTCCTAGAGGCTGCATatAATCCCCAATTaTCcCCcaGTT
ST-E00144:68:H2LCKCCXX:3:2106:15260:70610+ AAAAGGCCGCACAGGGCCTGATCTGCTCCCTTCCCTTGTCCAGGGAAAATTGCAGTCGACACAAATACAGCCAATcCCAGGAGACAGTCATGCTGAAAGGGCCCTGTGTCCTAGAGGCTGCATAtAATCCCCAATtATCccCCAGTTTCC
ST-E00144:68:H2LCKCCXX:4:2202:25786:40706+ GGCCGCACAGGGCCTGaTCTGCTCCCTTCCCTTGTCCAGGGaAcATTGCAGTCGACACaAAtACAGCCAATcCCaGGAGACaGTCATGCTGAAAGGGCCCtgtGTCctAgAGGCTgCATATAATCCCCAATtATCCaccaGTTtaccCAG
ST-E00144:68:H2LCKCCXX:3:1216:15798:1115 + CAGGGCCTGATCTGCTCCCTTCCCTTGTCCAGGGAAAATTGCAGTCGACACAAATACAGCCAATCCCAGGAGACAGTCATGCTGAAAGGGCCCTGTGTCCTAGAGGCTGCATATAATCCCCAATTATCCCCCAGtTTCCCCAGGGAGAGA
ST-E00144:68:H2LCKCCXX:4:1109:10673:37384+ CAGGGCCTGATCTGCTCCCTTCCCTTGTCCAGGGAAAATTGCAGTCGACACAAATACAGCCAATCCCAGGAGACAGTCATGCTGAAAGGGCCCTGTGTCCTAGAGGCTGCATATAATCCCCAATTATCCCCCAGTTTCCCCAGGGAGaGA
ST-E00144:68:H2LCKCCXX:4:1213:17006:52819+ CAGGGCCTGATCTGCTCCCTTCCCTTGTCCAGGGAAAATTGCAGTCGACACAAATACAGCCAATCCCAGGAGACaGTCATGCTGAAAGGGCCCTGTGTCCTAGAGGCTGCATATAATCCCCAATTATCCCCCAGTTTCCCCAgGGAGaGA
ST-E00144:68:H2LCKCCXX:4:1115:8135:68536 + GaTCTGCTCCCTTCCCTTGTCCAGGGAAAATTGCAGTCGACACAAATACAGCCAATCCCAGGAGACAGTCATGCTGAAAGGGCCCTGTGTCCTAGAGGCTGCATATAATCCCCAATTATCCCCCAGTTTCCCCAGGgagaGAATGGAGag
ST-E00144:68:H2LCKCCXX:4:1104:29663:28681+ GCTCCCTTCCCTTGTCCAGGGAAAATTGCAGTCGACACAAATACAGCCAATCCCAGGAGACAGTCATGCTGAAaGGGCCCTGTGTCCTAGAGGCTGCATATAATCCCCAATTATCCCCCAGTTTCCCCAGGGAGAGaAtGGAGaGGtGAG
ST-E00144:68:H2LCKCCXX:7:1124:28801:6934 + GCTCCCTTCCCTTGTCCAGGGAAAATTGCAGTCGACACAAATACAGCCAATCCCAGgaGACAGTCATGCTGAAAGgGCCCTGTGTCCTAGAGGCTGCaTATAATCCCCAAtTATCCCCCcGttTCCCCAGGGAGAGAActgGAGAGGTGA
ST-E00144:68:H2LCKCCXX:7:2111:19940:40214+ GCTCCCTTCCCTTGTCCAGGGAAAATTGCAGTCGACACAAATACAGCCAATCCCAGGAGACAGTCATGCTGAAAGGGCCCTGTGTCCTAGAGGCTGCATATAATCCCCAATTATCCCCCAGTTTCCCCAGGGAGAGAATGGaGAgGtGAG
ST-E00144:68:H2LCKCCXX:7:2111:20234:40337+ GCTCCCTTCCCTTGTCCAGGGAAAATTGCAGTCGACACAAATACAGCCAATCCCAGGAGACAGTCATGCTGAAAGGGCCCTGTGTCcTAGAGGCTGCATATAATCCCCAATTATCCCCCAGtTTCCCCAGGGaGAGAATGGAGAGGTGAG
ST-E00144:68:H2LCKCCXX:7:2124:26679:8710 + GCTCCCTTCCCTTGTCCAGGGAAAATTGCAGTCGACACAAATACAGCCAATCCCAGGAGACAGTCATGCTGAAAGGGCCCTGTGTCCTAGAGGCTGCATATAATCCCCAATTATCCCCCAGTTTCCCCAGgGAGAgAATGGAGAGGTGAG
ST-E00144:68:H2LCKCCXX:8:2105:30222:38333+ CCCTTCCCTTGTCCAGGGAAAATTGCAGTCGACACAAATACAGCCAATCCCAGGAGACAGTCATGCTGAAAGGGCCCTGTGTCCTAGAGGCTGCATATAATCCCAAAAGTTCACCaGTTCTTCcCAAAAGCACTacaCCGCTTCTGCCGG
ST-E00144:68:H2LCKCCXX:8:2105:29988:38386+ CCCTTCCCTTGTCCAGGGAAAATTGCAGTCGACACAAATACAGCCAATCCCAGGAGACAGTCATGCTGAAAGGGCCCTGTGTCCTAGAGGCTGCATATAATCCCAAAAGTTCACCAGTTCTTCCCAAAAGCACTACACCGCTTCTGCCGg
ST-E00144:68:H2LCKCCXX:8:2105:30343:38474+ CCCTTCCCTTGTCCAGGGAAAATTGCAGTCGACACAAATACAGCCAATCCCAGGAGACAGTCATGCTGAAAGGGCCCTGTGTCCTAGAGGCTGCATATAATCCCAAAAGTTCACCAGTTCTTCCCAAAAGCACTACACCGCTTCTGCCGG
ST-E00144:68:H2LCKCCXX:8:2105:30465:38509+ CCCTTCCCTTGTCCAGGGAAAATTGCAGTCGACACAAATACAGCCAATCCCAGGAGACAGTCATGCTGAAAGGGCCCTGTGTCCTAGAGGCTGCATATAATCCCAAAAGTTCACCAGTTCTTCCCAAAAGCACTACACCGCTTCTGCCGG
ST-E00144:68:H2LCKCCXX:8:2105:30465:38614+ CCCTTCCCTTGTCCAGGGAAAATTGCAGTCGACACAAATACAGCCAATCCCAGGAGACAGTCATGCTGAAAGGGCCCTGTGTCCTAGAGGCTGCATATAATCCCAAAAGTTCACCAGTTCTTCCCAAAAGCACTACAccGCTTCTgcCGg
ST-E00144:68:H2LCKCCXX:7:2205:13119:65389+ CCTTCCCTTGTCCAGGGAAAATTGCAGTCGACACAAATACAGCCAATCCCAGGAGACAGTCAtGCTGAAAGGGCcCTGTGTCCTAGAGGCTGCATATAATCCCCAATTATCCCCCAGTTTCCCCAGGGaGaGaatGGaGAGGtGaGGGCT
ST-E00144:68:H2LCKCCXX:1:1103:3070:70241 + CTTCCCTTGTCCAGGGAAAATTGCAGTCGACACAAATACAGCCAATCCCAGGAGaCaGTCATGCTGAAAGgGcCCTGTGtCcTAGAGGCTgCATATAATCCCCAATTATCcCCCaGTTTCcCcaGgGAGAGaATgGAgagGTGAGGGC
ST-E00144:68:H2LCKCCXX:8:2119:23543:43853+ GGAAAATTGCAGTCGACACAAATACAGCCAATCCCAGGAGACAGTCATGCTGAAAGGGCCCTGTGTCCTAGAGGCTGCATATAATCCCCAATTATCCCCCAGTTTCCCCAGGGAGAGAATGGAGAGGTGAGGGCTttCAAAAGCCAGC
ST-E00144:68:H2LCKCCXX:6:2108:18630:3840 + AAAATTGCAGTCGACACAAATACAGCCAATCCCAGGAGACAGtCATGCTGAAAGGGCCCTGTGTCCTAGAGGCTGCATATAATCCCCAATTATCCCCCAGTTTCCCCAGGGAGAGAATGGAGAGGTGAGGGCTTTCAAAAGCCAGCATCC
ST-E00144:68:H2LCKCCXX:8:1118:7536:17992 + ATTGCAGTCGACACAAATACAGCCAATCCCAGGAGACAGTCATGCTGAAAGGGCCCTGTGTCCTAGAGGCTGCATATAATCCCCAATTATCCCCCAGTTTCCCCAgGGAGAGAATGGaGAGGTGAGGGCttTCAaAAGCCAGCATCCCTT
ST-E00144:68:H2LCKCCXX:8:1109:26172:13703+ ACACAAATACAGCCAATCCCAGGAGACAGTCATGCTGAAAGGGCCCTGTGTCCTAGAGGCTGCATATAATCCCCAATTATCCCCCAGTTTCCCCAGGGAGAGAATGGAGAGGTGAGGGCTTTCAAAAGCCAGCATCCCTTCAGTCTCCAT
ST-E00144:68:H2LCKCCXX:6:1103:7607:48670 + AAATACAGCCAATCCCAGGAGACAGtCATGCTGAAAGGGCCCTGTGTCCTAgAGGCtGCATATAAtCCCCAATTATcCCCCAGtTTCCCCAGGGaGAGAATGGAGAGGgGagGGCTTgCAAAAGCCAGCaTccCTTcAGgctccaTtATG
ST-E00144:68:H2LCKCCXX:1:2211:3923:71190 + ATACAGCCAATCCCAGGAGACAGTCATGCTGAAAGGGCCCTGTGTCCTAGAGGCTGCATATAATCCCCAATTATCCCCCAGTTTCCCCAGGGAGAGAAtGGAGAGGTGAGGGCTTTCAAAAgCCAGCATCCCTTCAGTCTCCATgATGCG
ST-E00144:68:H2LCKCCXX:1:2212:24741:26308+ CAGCCAATCCCAGGAGACAGTCATGCTGAAAGGGCCCTGTGTCCTAGAGGCTGCATATAATCCCCAATTATCCCCCAGTTTCCCCAGGGAGAGAATGGAGAGGTGAGGGCTTTCAAAAGCCAGCATCCCTTCAGTCTCCATTATGCGGAG
ST-E00144:68:H2LCKCCXX:1:2102:12703:71419+ CAATCCCAGGAGACAGTCATGCTGAAAGGGCCCTGTGTCCTAGAGGCTGCATATAATCCCCAATTATCCCCCAGTTTCCCCAGGGAGAGAATGGAGAGGTGAGGGCTTTCAAAAGCCAGCATCCCTTCAGTCTCCATTATtCgGAGaCTC
ST-E00144:68:H2LCKCCXX:7:2206:13880:3946 + CAATCCCAGGAGACAGTCATGCTGAAAGGGCCCTGTGTCCTAGAGGCTGCATATAATCCCCAATTATCCCCCAGTTTCCCCAGGGAGAGAATGGAGAGGtGAGGGCTTTCAAAAGCCAGCATCCCTTCAGTCtcCATTATGCGGAGACTC
ST-E00144:68:H2LCKCCXX:3:1106:21888:41761+ CCCAGGAGACAGTCATGCTGAAAGGGCCCTGTGTCCTAGAGGCTGCATATAATCCCCAATTATCCCCCAGTTTCCCCAGGGAGAGAATGGAGAGGTGAGGGCTTTCAAAAGCCAGCATCCCTTCAGTCTCCATTATGCGGAGACTCACAC
ST-E00144:68:H2LCKCCXX:8:1212:14844:40566+ AGGAGACAGTCATGCTGAAAGGGCCCTGTGTCCTAGAGGCTGCATATAATCCCCAATTATCCCCCAGTTTCCCCAGGGAGAGAATGGAGAGGTGAGGGCTTTCAAAAGCCAGCATCCCTTCAGTCTCCATTATGCGGAGACTCACAcAGC
ST-E00144:68:H2LCKCCXX:4:1211:26720:34553+ GGAGACAGTCATGCTGAAAGGGCCCTGTGTCCTAGAGGCTGCATATAATCCCCAATtATCCCCCAGTTTCCCCAGGGAGAGAATGGAGAGGTGAGGGCTTTCAAAAGCCAGCATCCCTTCAGtCTCCATTATGCGGAGaCTCACACAGCC
ST-E00144:68:H2LCKCCXX:7:1113:8927:53909 + AGtCATGCTGaAAGGGCCCTGTGTCCTaGAGGCTGCAtaTAATCCCCAATTATCCCCCAGTTTCCCCAGGGAGAGAAtGGAGaGGTGAGGGCTTTCAcaaGCCAGCATCCCTTCAGTCTCCATTATGCGGAGaCTCACACAGCCTCCTTt
ST-E00144:68:H2LCKCCXX:7:1103:16326:65459+ AAGGGCCCTGTGTCCTAGAGGCTGCATATAATCCCCAATTATCCCCCAGTTTCCCCAGGGAGAGAATGGAGAGGTGAGGGCTTTCAAAAGCCAGCATCCCTTCAGTCTCCATTATGCGGAGACTCACACAGCCTCCTTTCTCCAAACAGC
ST-E00144:68:H2LCKCCXX:7:2214:26699:10081+ GGCCCTGTGTCCTAGAGGCTGCATATAATCCCCAATTATCCCCCAGTTTCCCCAGGGAGAGAATGGAGAGGTGAGGGCTTTCAAAAGCCAGCATCCCTTCAGTCTCCATTATGCGGAGACTCACACAGCCtCCTTTCTCCAAACAGCACA
ST-E00144:68:H2LCKCCXX:1:1105:25075:46191+ GCCCTGTGTCCTAGAGGCTGCATATAATCCCCAATTATCCCCCAGTTTCCCCAGGGAGAGAATGGAGAGGTGAGGGCTTTCAAAAGCCAGCATCCCTTCAGTCTCCATTATGCGGAGACTCACACAGCCTCCTTTCTCCAAACAGCACAG
ST-E00144:68:H2LCKCCXX:8:2109:32647:33252+ GCCCTGTGTCCTAGAGGCTGCATATAATCCCCAATTATCCCCCAGTTTCCCCAGGGAGAGAATGGAGAGGTGAGGGCTTTCAAAAGCCAGCATCCCTTCAGTCTCCATTATGCGGAGACTCACACAGCCTCCTTTCTCCAAACAGCACAG
ST-E00144:68:H2LCKCCXX:4:1215:32475:1449 + CCTAGAGGCTGCATATAATCCCCAATTATCCCCCAGTTTCCCCAGGGAGAGAATGGAGAGGTGAGGGCTTTCAAAAGCCAGCATCCCTTcAGtCTCCATTAtGCGGAgACTcACACAgcCtCCTTcCTccAAaCAGcAcAGAAGGtcaca
ST-E00144:68:H2LCKCCXX:7:2103:25502:66655+ CTAGAGGCTGCATATAATCCCCAATTATCCCCCAGTTTCCCCAGGGAGAGAATGGAGAGGTGAGGGCTTTCAAAAGCCAGCATCCCTTCAGTCTCCATTATGCGGAGACTCACACAGCCTCCTTTCTCCAAACAGCACAGAAGGTCACAG
ST-E00144:68:H2LCKCCXX:1:2106:12449:71612+ AGgCTGCATATAATCCaCAATTATCgCCCAGTTTCCCCAGGGAGAGAATGGAGAGgTGAGgGCTTTCAAAAGCCAGCATCCcTTCAGtCTCCAtTATGCGGAGACTCACACAGCCTcctTTCTcCaAAcAGcACAgAaGttcAcAgggAC
ST-E00144:68:H2LCKCCXX:4:1115:19797:35854+ GCTGCATATAATCCCCAATTaTCCCCCAGTTTCCCCcGGGAGAGAAtgGaGAGGTGAGGGCTTTCAAAAGCCAGCATCCCTTCAGtctCCATtatGCGGAGACTCACACaGCCTCCTTTCTCCAAACAGCACAGAAGGTaacAgGgacAc
ST-E00144:68:H2LCKCCXX:4:2217:28222:37788+ CTGCATATAATCCCCAATTATCCCCCAGTTTCCCCAGGGAGAGAATGGAGAGGTGAGGGCTTTCAAAAGCCAGCATCCCTTCAGTCTCCATTATGCGGAGACTCACACAGCcTCCTTTCTCCAAACaGCACAGAAGGTcACAGGGACACG
ST-E00144:68:H2LCKCCXX:6:1122:25289:57952+ CATATAATCCCCAATTATCCCCCAGTTTCCCCAGGGaGAGAATGGAGAGGTGAGGGCTTTCAAAAGCCAGCATCCCTTCAGTCTCCATTATGCGGAGACTCACACAGCCTCCTTTCTCCAAACAGCACAGAAGGTCACAGGGACACGGGG
ST-E00144:68:H2LCKCCXX:4:1103:22518:58181+ TCCCCAATTATCCCCCAGTTTCCCCAGGGAGAGAATGGAGAGGTGAGGGCTTTCAAAAGCCAGCATCCCTTCAGTCTCCATTATGCGGAGACTCACACAGCCTCCTTTCTCCAAACAGCACAGAAGGTCACAGGGACACGGGGCAACAGA
ST-E00144:68:H2LCKCCXX:8:2212:10845:65354- ATTCCCAGCTCTGAAAAGGCCGCACAgGGCCTGATCTGCTCCCTtCCCTTGTCCAGGGAAAATTGCAGTCGACACAAATACAGCCAATCCCAGGAGACAGTCATGCTGAAAGGGCCCTGTGTCCTAGAGGCTGCATATAATCCCCAATTA
ST-E00144:68:H2LCKCCXX:1:1120:28973:27697- TTCCCAGCTCTGAAAAGGCCGCACAGGGCCTGATCTGCTCCCTTCCCTTGTCCAGGGAAAATTGCAGTCGACACAAATACAGCCAATCCCAGGAGACAGTCATGCTGAAAGGGCCCTGTGTCCTAGAGGCTGCATATAATCCCCAATTAT
ST-E00144:68:H2LCKCCXX:8:2201:13220:58251- CCCAGCTcTGAAaaGGCCGCACAGGGCCTGATCTGCTCCCTTCCCTTGTcCAGGGAAAATTGCAGTCGACACAAATACAGCCAATCCCAGGAGACAGTCATGCTGAAAGGGCCCTGTGTCCTAGAGGCTGCATATAATCCCCAATTATCC
ST-E00144:68:H2LCKCCXX:8:2223:24659:56792- CcGCtCTGAaAaGGCCGCACaGGGCCTGAtCTGCTCCCTTCCCTTGtCCAGGGAAAATTGCAGtCGACACAAATACAGCCAATCCCAGGAGACAGTCATGCTGAAAGGGCCCTGTGTCCTAGAGGCTGCATATAATCCCCAATTATCCCC
ST-E00144:68:H2LCKCCXX:3:1206:14550:72614- AGCTCTGaAAaGGCCGCACAGGGCCTGATCTGCTCCCTTCCCTTGGCCAGGGAAAATTGCAGTCGACACAAATACAGCCAATCCCAGGAGACAGTCATGCTGAAAGGGCCCTGTGTCCTAGAGGCTGCATATAATCCCCAATTATCCCCC
ST-E00144:68:H2LCKCCXX:6:2113:10155:5264 - CGCACAGGGCCTGATCTGCTCCCTTCCCTTGTCCAGGGAAAATTGCAGTCGACACAAATACAGCCAATCCCAGGAGACAGTCATGCTGAAAGGGCCCTGTGTCCTAGAGGCTGCATATAATCCCCAATTATCCCCCAGTTTCCCCAGGGA
ST-E00144:68:H2LCKCCXX:7:2116:26486:43642- TCCCTTgTCCAGGGAAAATTGCAGTCGACACAAATACAGCCAATCCCAGGAGACAGTCATGCTGAAAGGGCCCTGTGTCCTAGAGGCTGCATATAATCCCCAATTATCCCCCaGTTTCCCCAGGGAGAGAATGGAGaGGTGAGGGCTTTC
ST-E00144:68:H2LCKCCXX:7:2219:19929:71700- TCCCTTGTCCAGGGAAAATTGCAGTCGACACAAATACAGCCAATCCCAGGAGACAGTCATGCTGAAAGGGCCCTGTGTCCTAGAGGCTGCATATAATCCCCAATTATCCCCCAGTTTCCCCAGGGAGAGAATGGAGAGGTGAGGGCTTTC
ST-E00144:68:H2LCKCCXX:7:1123:29135:33498- GTCCAgGGAAAATTGCAGTCGACACAAATACAGCCAATCCCAGGAGACAGTCATGCTGAAAGGGCCCTGTGTCCTAGAGGCTGCATATAATCCCCAATTATCCCCCAGTTTCCCCAGGGAGAGAATGGAGAGGTGAGGGCTTTCAAAAGC
ST-E00144:68:H2LCKCCXX:7:2104:12804:38966- CCAGGGAAAATTGCaGTCGACACAAATACAGCCAATCCCAGGAGACAGTCATGCTGAAAGGGCCCTGTGTCCTAGAGGCTGCATATAaTCCCCAATTATCCCCCAGTTTCCCCAGGGAGAGAATGGAGAGGTGAGGGCTTTCAAAAGCCA
ST-E00144:68:H2LCKCCXX:3:2113:16580:44943- AATTGCaGtCGACACAAATACAGCCAATCCCAGGAGACAGTCATGCTGAAAGGGCCCTGTGTCCTAGAGGCTGCATATAATCCCCAATTATCCCCCAGTTTCCCCAGGGAGAGAATGGAGAGGTGAGGGCTTTCAAAAGCCAGCATCCCT
ST-E00144:68:H2LCKCCXX:8:1121:5110:53100 - tagcAgacgacacgAATAcAGCCAAtcaCAggaGACAGTCATGCTGAAagGgCcctGTGtcCTAGAGGCtGaAtAtAacCcCCaATtAtcaCCCAgtTgcccCaGggAGAGAATGGAgAGGTgAGGgCtTTcaaaAGCCAgcatccCTtC
ST-E00144:68:H2LCKCCXX:8:2218:23096:22792- AGTCGACACAAAtACAGCCAATCCCAGGAGACAGtcAtGCTGAAAGGGCCCTGtGTCCTAGAGGCTGCATATAATCCCCAATtAtCCCCCAGTTTCCCCAGGGAGAGAATGGAGAGGTGAGGGCTTTCAAAAGCCAGCATCCCTTCAGTC
ST-E00144:68:H2LCKCCXX:6:1113:8480:40513 - GtcgACacaaATacagcCaCTCcCAggaGacagtCaTGCtGaAaGGGCCctGTGTCCTAGAGGCTGCATATaatcCCCAATTAtCCCCcAgTTTCCCCaGGGAGaGAATGGAGAGgTGAGGGCTtTCAAAaGCCAGCATCCCTTCAGTCt
ST-E00144:68:H2LCKCCXX:7:1123:3659:30439 - CGACACAAATaCAGCcAATCCcAGGAGACaGTCATGCTGAAAGGgCCCTGTGTCCTAGAGGCTGCATATAAtCCCCAATTAtCCCCCAGTTTCCCCAGGGAGAGAgTGGAGAGGTGAGGGcTTTCAAAAGCCAGCATCCCTTCAGTCtCC
ST-E00144:68:H2LCKCCXX:6:2212:11109:18942- GACACAAATACAGCCAATCCCAGGAGACAGTCATGCTGAAAGGGCCCTGTGTCCTAGAGGCTGCATATAATCCCCAATTATCCCCCAGTTTCCCCAGGGAGAGAATGGAGAGGTGAGGGCTTTCAAAAGCCAGCATCCCTTCAGTCTCCA
ST-E00144:68:H2LCKCCXX:6:1221:14986:39194- CAAaTACAGCCaAtCcCAGGAGACAGTCATGCTGAAAGGGCCCTGTGTCCTAGAGGCTGCATATAATCCCCAATTATCCCCCAGTTTCCCCAGGGAGAGAATGGAGAGGTGAGGGCTTTCAAAAGCCAGCATCCCTTCAGTCTCCATTAT
ST-E00144:68:H2LCKCCXX:7:1113:14966:63807- cAAATACAGCcAATCCCAGGAGACAGTCATGCTGAAAGGGCCCTGTGTCCTAGAGGCTGCATATAATCCCCAATTatCCCCCAGTTTCCCCAGGGAGAGAATGGAGAGGTGAGGGCTTTCAAAAGCCAGCATCCCTTCAGTCTCCATTAT
ST-E00144:68:H2LCKCCXX:8:1201:20914:8816 - CAGCCAATCcCAGGAGACAGTCATGCTGAAAGGGCCCTGTGTCCtAGAGGCTGCATATAATCCCCAATTATCCCCCAGTTTCCCCAGGGAGAGAATGGAGAGGTGAGGGCTTTCAAAAGCCAGCATCCCTTCAGTCTCCATTATGCGGAG
ST-E00144:68:H2LCKCCXX:6:2112:28049:39563- GCCAATCCCAGGAGACAGTCATGCTGAAAGGGCCCTGTGTCCTAGAGGCTGCATATAATCCCCAATTATCCCCCAGTTTCCCCAGGGAGAGAATGGAGAGGTGAGGGCTTTCAAAAGCCAGCATCCCTTCAGTCTCCATTATGCGGAGAC
ST-E00144:68:H2LCKCCXX:3:2124:19006:35116- TCCCAGGAGACAGTCATGCTGAAAGGGCCCTGTGTCCTAGAGGcTGCATATAaTCCCCAATTATcCCCCAGTttcCCCaGGGAGAGAATGGAGAGGTGAGGGCTTTCAAAAGCCAGCAtCCCTTCAGTCTCCATTATGCGGAGACTCACA
ST-E00144:68:H2LCKCCXX:6:1109:28303:59798- GACAGTCATGCTGAAAGGgCCCTGTGTCCTAGAGGCTGCATATaATCCCCAaTTATCCCCCAGTTTCCCCAGGGAGAGAATGGAGAGGTGAGGGCTTTCAAAAGCCAGCATCCCTTCAGTCTCCATTATGCGGAGACTCACACAGCCTCC
ST-E00144:68:H2LCKCCXX:1:1218:17209:16990- aGTCATGCTGaaAGGGCCCTgcGtCgTAGagGCTGCATATAATCcCCAATTAtCcCCCAGTTTCCCcaGGgAGAGAATGGAGAGGTGAGgGCttTCAAAAGCCAGCATCCCTTCAGTCTCCAtTATGCGGAGACtcACACAGCCTCCTTT
ST-E00144:68:H2LCKCCXX:1:2110:16824:28804- CATGCTGAAAGGGCCCTGTGTCCTAGAGGCTGCATATAATCCCCAATTATCCCCCAGTTTCCCCAGGGAGAGAATGGAGAGGTGAGGGCTTTCAAAAGCCAGCATCCCTTCAGTCTCCATTATGCGGAGACTCACACAGCCTCCTTTCTC
ST-E00144:68:H2LCKCCXX:1:1217:15088:30439- GCTGAAAGGGCCCTGTGtCCTAGAGGCTGCATatAATCCCCAaTTaTCCCCCAGTTTCCCCAGGGAGAGAATGGAGAGGTGAGGGCTTTCAAAAGCCAGCATCCCTTCAGTCTCCATTATGCGGAGACTCACACAGCCTCCTTTCTCCAA

ST-E00144:68:H2LCKCCXX:1:2123:4684:71806 - CTGtgtCCTaGAGGCTGcatatAaTCCCCAATtAtCcCCCAGTTTCCCCAGGGAGAGAATGGAGAGGTGAGGGCTTTCAAAAGCCAGCATCCCTTCAGTCTCCATTATGCGGAGACTCACACAGCCTCCTTTCTCCAAACAGCACAGAAG
ST-E00144:68:H2LCKCCXX:4:1203:20335:47369- CtAGAGGCTGCAtATAATCCCCAATTATCCCCCAGTTTCCCCAGGGAGAGAATGGAGAGGTGAGGGCTTTCAAAAGCCAGCATCCCTTCAGTCTCCATTATGCGGAGACTCACACAGCCTCCTTTCTCCAAACAGCACAGAAGGTCACAG
ST-E00144:68:H2LCKCCXX:8:2122:23817:17219- AGGCTGCATATAATCCCCAAtTAtCCCCCAGTTTCCCCAGGGAGAGAATGGAGAGGTGAGGGCTTTCAAAAGCCAGCATCCCTTCAGTCTCCATTATGCGGAGACTCACACAGCCTCCTTTCTCCAAACAGCACAGAAGGTCACAGGGAC
ST-E00144:68:H2LCKCCXX:3:2115:10591:32426- CCAATTATCCCcCAGTTTCCCCAGgGAGAGAATGGAGAGGTGAGGGCTTTCAAAAGCCAGCATCCCTTCAGTCTCCATTATGCGGAGACTCACACaGCCTCCTTTCTCCAAACAGCACAGAagGTCACAGGgACacgGggCaacaGaGgC
```

```
ST-E00192:51:H2KG3CCXX:2:1119:21066:42130+ CAGCTCTGAAAAGGCCGCACAGGGCCTGATCTGCTCCCTTCCCTTGTCCAGGGAAAATTGCAGTCGACACAAATACAGCCAATCCCAGGAGACAGTCATGCTGAAAGGGCCCTGTGTCCTAGAGGCTGCATATAATCCCCAATTATCCCC
ST-E00192:51:H2KG3CCXX:1:1201:6846:69538 + AAGGCCGCACAGGGCCTGATCTGCTCCCTTCCCTTGTCCAGGGAAAATTGCAGTCGACACAAATACAGCCaATCCCAGGAGACAGTCATGCTGAAAGGGCCCTGTGTCCTAGAGGCTGCATATaaTCCCCAATTatCCCCCAGTTTCaCC
ST-E00192:51:H2KG3CCXX:8:2201:31165:54665+ AGGCCGCACAGGGCCTGATCTGCTCCCTTCCCTTGTCCAGGGAAAATTGCAGTCGACACAAATACAGCCAATCCCAGGAGACAGTCATGCTGAAAGGGCCCTGTGTCCTAGAGGCTGCATATAATCCCCAATtaTCCCCCAGTTTCCCCA
ST-E00192:51:H2KG3CCXX:8:2201:31216:54718+ AGGCCGCACAGGGCCTGATCTGCTCCCTTCCCTTGTCCAGGGAAAATTGCAGTCGACACAAATACAGCCAATCCCAGGAGACAGTCATGCTGAAAGGGCCCTGTGTCCTAGAGGCTGCATATAATCCCCAATTATCCCCCaGTTTCCCCA
ST-E00192:51:H2KG3CCXX:2:2123:9028:52854 + GCACAGGGCCTGATCTGCTCCCTTCCCTTGTCCAGGGaAAATTGCAGTCGACACAAATACAGCCAATCCCAGGAGACAGTCATGCTGAAaGGGCCCTGTGTCCTAGAGGCTGCATATAATCCCCAATTATCCCCCAGTTTCCCCAgGGAG
ST-E00192:51:H2KG3CCXX:6:1215:7963:20383 + GCACAGGGCCTGATCTGCTCCCTTCCCTTGTCCAGGGAAAATTGCAGTCGACACAAATACAGCCAATCCCAGGAGACAGTCATGCTGAAAGGGCCCTGTGTCCTAGAGGCTGCATATAATCCcCAATTATCCCCCAGTTTCCCCAGGGAG
ST-E00192:51:H2KG3CCXX:8:2211:19016:70540+ GCACAGGGCCTGATCTGCTCCCTTCCCTTGTCCAGGGAAAATTGCAGTCGACACAAATACAGCCAATCCCAGGaGACAGTaaTGCTGAAAgGGCCcTGtGtcCTAGAGGctGCatAgAAtCCcCAATtAgcCccCAgtttcCCCAGGGag
ST-E00192:51:H2KG3CCXX:1:2123:17341:42675+ GGgcCTGATCTGCTCCCTTCCCtTGTCCAGGGAAAATTGCAGTCGACACAAATACAGCCAATCCCAGGAGACAGTCATGCTGAAAGGGCCCtGTGTCCTAGaGGCTGCATATaAGCCCcAattaTgccccAtgtTCCCcGgggAGaGAAT
ST-E00192:51:H2KG3CCXX:1:2123:17351:42693+ GGGCCTGATCTGCTCCCTTCCCTTGTCCAGGGAAAATTGCAGTCGACACAAATACAGCCAATCCCAGGAGACAGTCATGCTGAAAGGGCCCTGTGTCCTAGAGGCTGCATATAAgCcCAATTATTCCCCAatttCCCCagGGAGAGAAT
ST-E00192:51:H2KG3CCXX:3:2222:32820:59113+ TGATCTGCTCCCTTCCCTTGTCCAGGGAAAATTGCAGTCGACACAAATACAGCCAATCCCAGGAGACAGTCATGCTGAAAGGGCCCTGTGTCCTAGAGGCTGCATATAATCCCCAATTATCCCCCAGTTTCCCCAGGGAGAGAAtgGaGA
ST-E00192:51:H2KG3CCXX:5:2113:25553:34289+ GATCTGCTCCCTTCCCTTGTCCAGGGAAAATTGCAGTCGACACAAATACAGCCAATCCCAGGAGACAGTCATGCTGAAAGGGCCCTGTGTCCTAGAGGCTGCATATAATCCCCAATTATCCCCCAGTTTCCCCAGGGAGAGAATGGAGAG
ST-E00192:51:H2KG3CCXX:5:2113:25502:34377+ GATCTGCTCCCTTCCCTTGTCCAGGGAAAATTGCAGTCGACACAAATACAGCCAATCCCAGGAGACAGTCATGCTGAAAGGGCCCTGTGTCCTAGAGGCTGCATATAATCCCCAATTATCCCCCAGTTTCCCCAGGGAGAGAATGGAGAG
ST-E00192:51:H2KG3CCXX:5:1223:27603:51940+ TTCCCTTGTCCAGGGAAAATTGCAGTCGACACAAATACAGCCAATCCCAGGAGACAGTCATGCTGAAAGGGcCCTGTGTCCTAGAGGCTGCATATAATCCCCaATTATCCCCCAGTTTCCCCAGGGaGAGaATGGAGagGtGAgGGCTTT
ST-E00192:51:H2KG3CCXX:8:1204:26162:29543+ CCAGGGAAAATTGCAGTCGACACAAATACAGCCAATCCCAGGAGACAGTCATGCTGAAAGGGCCCTGtGTCcTAGAGGCTGCATATAATCCCCAATTATCCCCCAGTTTCCCCAGGGAGAGAATGGAGagGtGAGGGCTtTCAAAAGCCA
ST-E00192:51:H2KG3CCXX:8:2110:22741:32865+ AAAATTGCAGTCGACACAAATACAGCCAATCCCAGGAGACAGTCATGCTGAAAGGGCCCTGTGTCCTAGAGGCTGCATATAATCCCCAATTATCCCCCAGTTTCCCCAGGGAGAGAATGGAGAGGTGAGGGCTTTCAAAAGCCAGCATCC
ST-E00192:51:H2KG3CCXX:8:1208:2441:8429  + CGACACAAATACAGCCAATCCCAGGAGACAGTCATGCTGAAAGGGCCCTGTGTCCTAGAGGCTGCATATAATCCCCAATTATCCCCCAGTTTCCCCAGGGAGAGAATGGAGAGGTGAGGGCTTTCAAAAGCCAGCATCCCTTCAGTCTCC
ST-E00192:51:H2KG3CCXX:2:1216:6887:41867 + AAATACAGCCAATCCCAGGAGACAGTCATGCTGAAAGGGCCCTGTGTCCTAGAGGCTGCATATAATCCCCAATTATCCCCCAGTTTCCCCAGGGAGAGAATGGAGAGGTGAGGGCTTTCAAAAGCCAGCATCCCTTCAGTCTCCATTATG
ST-E00192:51:H2KG3CCXX:5:1123:4034:30105 + ATACAGCCAATCCCAGGAGACAGTCATGCTGAAAGGGCCCTGTGTCCTAGAGGCTGCATATAATCCCCAATTATCCCCCAGTTTCCCCAGGGAGAGAATGGAGAGGTGAGGGCTTTCAAAAGCCAGCATCCCTTCAGTCTCCaTTaTGCG
ST-E00192:51:H2KG3CCXX:1:1115:12114:66743+ CCAATCCCAGGAGACAGTCATGCTGAAAGGGCCCTGTGTCCTAGAGGCTGCATATAATCCCCAATTATCCCCCAGTTTCCCCAGGGAGAGAATGGAGAGGTGAGGGCTTTCAAAAGCCAGCATCCCTTCAGTCTCCATTATGCGGAGACT
ST-E00192:51:H2KG3CCXX:1:1115:11231:16305+ AATCCCAGGAGACAGTCATGCTGAAAGGGCCCTGTGTCCTAGAGGCTGCATATAATCCCCAATTATCCCCCaGTTTCCCCAGGGAGAGaATGGAGAGGTGAGGGCTTTCAAaAGCCAGCATCCCTTCAGTCTCCATTATGCGGAGACTCA
ST-E00192:51:H2KG3CCXX:3:1110:19848:40724+ AATCCCAGGAGACAGTCATGCTGAAAGGGCCCTGTGTCCTAGAGGCTGCATATAATCCCCAATTATCCCCCAGTTTCCCCAGGGAGAGAATGGAGAGGTGAGGGCTTTCAAAAGCCAGCATCCCTTCAGTCTCCaTTATGCGGaGACTCA
ST-E00192:51:H2KG3CCXX:3:1110:19879:40741+ AATCCCAGGAGACAGTCATGCTGAAAGGGCCCTGTGTCCTAGAGGCTGCATATAATCCCCAATTATCCCCCAGTTTCCCCAGGGAGAGAATGGAGAGGTGAGGGCTTTCAAAAGCCAGCATCCCTTCAGTCTCCATTATGcGGaGACtca
ST-E00192:51:H2KG3CCXX:8:1102:31094:52362+ CAGGAGACAGTCATGCTGAAAGGGCCCTGTGTCCTAGAGGCTGCATATAATCCCCAATTATCCCCCaGTTTCCCCaGGGAGAGaATGGAGAGGTGAGGGCTTTCaAAAGCCAGCATCCCTTCAGTCTCCATTATGcGGAGAcTCACACAG
ST-E00192:51:H2KG3CCXX:8:1202:11921:66584+ AGGAGACAGTCATGCTGAAAGGGCCCTGTGTCCTAGAGGCTGCATATAATCCCCAATTATCCCCCAGTTTCCCCAGgGAGAGAATGGAGaGGTGAGGGCTTTCAAAAGCCAGcATCCCTTCAGTCTCCAtTATGCGGAGACTCACACAGC
ST-E00192:51:H2KG3CCXX:1:2120:27978:18801+ AGTCATGCTGAAAGGGCCCTGTGTCCTAGAGGCTGCATATAATCCCCAATTATCCCCCAGTTTCCCCAGGGAGAGAATGGAGAGGTGAGGGCTTTCAAAAGCCAGCATCCCTTCAGTCTCCATTATGCGGAGACacACACAGCCTCcTtt
ST-E00192:51:H2KG3CCXX:1:2120:27471:19293+ AGTCATGCTGAAAGGGCCCTGTGTCCTAGAGGCTGCATATAATCCCCAATTATCCCCCAGTTTCCCCAGGGAGAGAATGGAGAGGTGAGGGCTTTCAAAAGCCAGCATCCCTTCAGTCTCCATTATGCGGAGACtaaCACAGCCtCCTTT
ST-E00192:51:H2KG3CCXX:2:1117:19787:47580+ AAAGGGCCCTGTGTCCTAGAGGCTGCATATAATCCCCAATTATCCCCCAGTTTCCCCAGGGAGAGAATGGAGAGGTGAGGGCTTTCAAAAGCCAGCATCCCTTCAGTCTCCATTATGCGGAGACTCaCACAGCCTCCTTTCTCCAAACAG
ST-E00192:51:H2KG3CCXX:2:1117:20153:47650+ AAAGGGCCCTGTGTCCTAGAGGCTGCATATAATCCCCAATTATCCCCCAGTTTCCCCAGGGAGAGAATGGAGAGGTGAGGGCTTTCAAAAGCCAGCATCCCTTCAGTCgCCATTATGCGGAGACTCACACAGCCTCCttTCTCCAAACAG
ST-E00192:51:H2KG3CCXX:3:1212:18316:9202 + AAGGGCCCTGTGTCCTAGAGGCTGCATATAATCCCCAATTATCCCCCAGTTTCCCCAGGGAGAGAATGGAGAGGTGAGGGCTTTCAAAAGCCAGCATCCCTTCAGTCTCCATTATGCGGAGACTCACACAGCCTCCTTTCTCCAAACAGC
ST-E00192:51:H2KG3CCXX:3:1212:18255:9273 + AAGGGCCCTGTGTCCTAGAGGCTGCATATAATCCCCAATTATCCCCCAGTTTCCCCAGGGAGAGAATGGAGAGGTGAGGGCTTTCAAAAGCCaGCATCCCTTCAGTCTCCATTATGCGGAGACTCACACAGCCTCCTTTCTCCAAACAGC
ST-E00192:51:H2KG3CCXX:1:2224:29785:59517+ AGGGCCCTGTGTCCTAGaGGCTgCATATAATCCCCAATTATCCCCCAGTTTCCCCAGGGAGaGAATGGAGAGGtGAGGGCTTTCAAAaGCCAGCATCCCTTCAGTCTCCATTATgcGGAgactCACACaGCCaCCttTCTCCaAaCAGCA
ST-E00192:51:H2KG3CCXX:1:2224:29755:59535+ AGGGCCCTGTGTCCTAGAGGCTGCATATAATCCCCAATTATCCCCCAGTTTCCCCAGGGAGAGAATGGAGAGGTGAGGGCTTTCAAAAGCCAGCaTCCCTTCAGTCTCCATtATGCGGAGaCtcAcACAGCCTCctTtctCCaAaCAGCA
ST-E00192:51:H2KG3CCXX:1:2224:29186:59886+ AGGGCCCTGTGTCCTAGAGGCTGCATATAATCCCCAATTATCCCCCAGTTTCCCCAGGGAGAGAATGGAGAGGTGAGGGCTTTCAAAAGCCAGCATCCCTTCAGtCTCCATTATGCGGAGACTCACACAGCCTCCTTTCTCCAaacAGCA
ST-E00192:51:H2KG3CCXX:2:1209:5709:27908 + AGGGCCCTGTGTCCTAGAGGCTGCATATAATCCCCAATTATCCCCCAGTTTCCCCAGGGAGAGAAtGGAGAGGTGAGGGCTTTCAAAAGCCAGCATCCCTTcAGTCTCCATTATGCGGAGACTCACACAGCCTCcTtTCTCCAAAcAGCA
ST-E00192:51:H2KG3CCXX:2:1209:5933:27978 + AGGGCCCTGTGTCCTAGAGGCtGCAtATAATCCCCAATTATCCCCCAGTTTCCCCAGGGAGAGAATGGAGAGGTGAGGGcTTTCAAAAGCCAGCATCCCTTCAGTCTCCATtATGCGGAGACTCACACAGCCTCCTTTCTCCAAAcAGCA
ST-E00192:51:H2KG3CCXX:8:2112:31561:38614+ CCTGTGTCCTAGAGGCTGCATATAATCCCCAATTATCCCCCAGTTTCCCCAGGGAGAGAATGGAGAGGTGAGGGCTTTCAAAAGCCAGCATCCCTTCAGTCTCCATTATGCGGAGACTCACACAGCCTCCTTTCTCCAAACAGCAcAGAA
ST-E00192:51:H2KG3CCXX:8:2112:31551:38632+ CCTGTGTCCTAGAGGCTGCATATAATCCCCAATTATCCCCCAGTTTCCCCAGGGAGAGAATGGAGAGGTGAGGGCTTTCAAAAGCCAGCATCCCTTCAGTCTCCATTATGCGGAGAcgCACACAGCCTCCTTTCtCCAAACAGCcCAGAA
ST-E00192:51:H2KG3CCXX:8:2211:3720:23759 + CCTGTGTCCTAGAGGCTGCATATAATCCCCAATTATCCCCCAGTTtCCCCAGGGAGAGAaTGGAGaGGTGAGGGCTTtcAAAAGCCAGCATCCCTTCAGTCTCCctTATGCGGAGActcagaCAGCcTCCTttCtCCAAACAgCACAGAA
ST-E00192:51:H2KG3CCXX:6:2219:27126:49145+ TGTCCTAGAGGCTGCATATAATCCCCAATTATCCCCCAGTTTCCCCAGGGAGAGAATGGAGAGGTGAGGGCTTTCAAAAGCCAGCATCCCTTCAGTCTCCaTTATGCGGAGACTCACACAGCCTCCTTTCtCCAAaCAGCACAGAAGGTC
ST-E00192:51:H2KG3CCXX:1:1114:10216:34061+ AGGCTGCATATAATCCCCAATTATCCCCCAGTTTCCCCAGGGAGAGAATGGAGAGGTGAGGGCTTTCAAAAGCCAGCATCCCTTCAGTCTCCATTATGCGGAGACTCACACAGCCTCCTTTCTCCAAACAGCACAGAAGGTCACAGGGAC
ST-E00192:51:H2KG3CCXX:1:1114:10125:34289+ AGgCTGCATATAATCCCCAATTATCCCCCAGTTTCCCCAGGGAGAGAATGGAGAGGTGAGGGCTTTCAAAAGCCAGCATCCCTTCAGTCTCCATTATGCGGAGACTCACACAGCCTCCTTTCTCCAAcCAGCACAGaAGGTCACaGgGAC
```

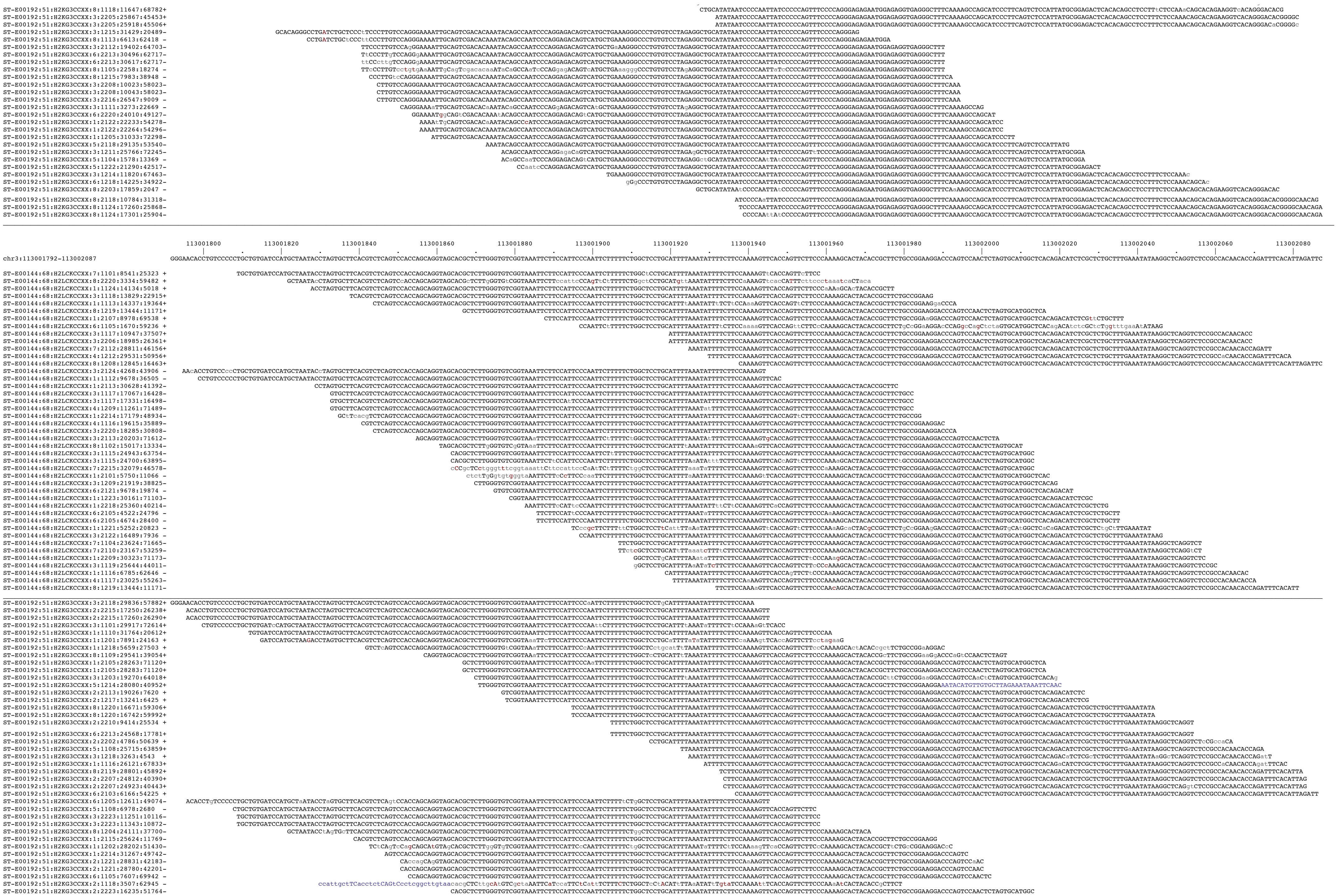

ST-E00192:51:H2KG3CCXX:8:1118:11647:68782+
ST-E00192:51:H2KG3CCXX:3:2205:25867:45453+
ST-E00192:51:H2KG3CCXX:3:2205:25918:45506+
ST-E00192:51:H2KG3CCXX:3:1215:31429:20489-
ST-E00192:51:H2KG3CCXX:8:1113:6613:62418 -
ST-E00192:51:H2KG3CCXX:3:2112:19402:64703-
ST-E00192:51:H2KG3CCXX:6:2213:30496:62717-
ST-E00192:51:H2KG3CCXX:6:2213:30617:62717-
ST-E00192:51:H2KG3CCXX:8:1105:2258:18274 -
ST-E00192:51:H2KG3CCXX:8:1215:7983:38948 -
ST-E00192:51:H2KG3CCXX:3:2208:10023:58023-
ST-E00192:51:H2KG3CCXX:3:2208:10043:58023-
ST-E00192:51:H2KG3CCXX:3:2216:26547:9009 -
ST-E00192:51:H2KG3CCXX:3:1111:3273:22669 -
ST-E00192:51:H2KG3CCXX:6:2220:24010:49127-
ST-E00192:51:H2KG3CCXX:1:2122:22233:54278-
ST-E00192:51:H2KG3CCXX:1:2122:22264:54296-
ST-E00192:51:H2KG3CCXX:1:1205:31033:72298-
ST-E00192:51:H2KG3CCXX:5:2118:29135:53540-
ST-E00192:51:H2KG3CCXX:3:1211:25766:72245-
ST-E00192:51:H2KG3CCXX:5:1104:1578:13369 -
ST-E00192:51:H2KG3CCXX:5:1222:21290:42517-
ST-E00192:51:H2KG3CCXX:3:1214:11820:67463-
ST-E00192:51:H2KG3CCXX:6:1218:14225:34922-
ST-E00192:51:H2KG3CCXX:8:2203:17859:2047 -
ST-E00192:51:H2KG3CCXX:8:2118:10784:31318-
ST-E00192:51:H2KG3CCXX:8:1124:17260:25868-
ST-E00192:51:H2KG3CCXX:8:1124:17301:25904-
113001800
113001820
113001840
113001860
113001880
113001900
113001920
113001940
113001960
113001980
113002000
113002020
113002040
113002060
113002080
chr3:113001792-113002087
GGGAACACCTGTCCCCCTGCTGTGATCCATGCTAATACCTAGTGCTTCACGTCTCAGTCCACCAGCAGGTAGCACGCTCTTGGGTGTCGGTAAATTCTTCCATTCCCAATTCTTTTTCTGGCTCCTGCATTTTAAATATTTTCTTCCAAAAGTTCACCAGTTCTTCCCAAAAGCACTACACCGCTTCTGCCGGAAGGACCCAGTCCAACTCTAGTGCATGGCTCACAGACATCTCGCTCTGCTTTGAAATATAAGGCTCAGGTCTCCGCCACAACACCAGATTTCACATTAGATTC
ST-E00144:68:H2LCKCCXX:7:1101:8541:25323 +
ST-E00144:68:H2LCKCCXX:8:2220:3334:59482 +
ST-E00144:68:H2LCKCCXX:1:1124:14134:5018 +
ST-E00144:68:H2LCKCCXX:3:1118:13829:22915+
ST-E00144:68:H2LCKCCXX:1:1113:14337:19364+
ST-E00144:68:H2LCKCCXX:8:1219:13444:11171+
ST-E00144:68:H2LCKCCXX:1:2107:8978:69538 +
ST-E00144:68:H2LCKCCXX:6:1105:1670:59236 +
ST-E00144:68:H2LCKCCXX:3:1117:10947:37507+
ST-E00144:68:H2LCKCCXX:3:2206:18985:26361+
ST-E00144:68:H2LCKCCXX:7:2112:28811:46156+
ST-E00144:68:H2LCKCCXX:4:1212:29531:50956+
ST-E00144:68:H2LCKCCXX:8:1208:12845:16463+
ST-E00144:68:H2LCKCCXX:3:2124:4268:43906 -
ST-E00144:68:H2LCKCCXX:1:1112:9678:36505 -
ST-E00144:68:H2LCKCCXX:1:2113:30628:41392-
ST-E00144:68:H2LCKCCXX:3:1117:17067:16428-
ST-E00144:68:H2LCKCCXX:3:1117:17331:16498-
ST-E00144:68:H2LCKCCXX:4:1209:11261:71489-
ST-E00144:68:H2LCKCCXX:1:2214:17179:48934-
ST-E00144:68:H2LCKCCXX:4:1116:19615:35889-
ST-E00144:68:H2LCKCCXX:3:2220:18285:30808-
ST-E00144:68:H2LCKCCXX:3:2113:20203:71612-
ST-E00144:68:H2LCKCCXX:8:1102:15017:13334-
ST-E00144:68:H2LCKCCXX:3:1115:24943:63754-
ST-E00144:68:H2LCKCCXX:3:1115:24700:63895-
ST-E00144:68:H2LCKCCXX:7:2215:32079:46578-
ST-E00144:68:H2LCKCCXX:1:2101:5750:11066 -
ST-E00144:68:H2LCKCCXX:3:1209:21919:38825-
ST-E00144:68:H2LCKCCXX:6:2121:9678:19874 -
ST-E00144:68:H2LCKCCXX:1:1223:30161:71103-
ST-E00144:68:H2LCKCCXX:1:2218:25360:40214-
ST-E00144:68:H2LCKCCXX:6:2105:4522:24796 -
ST-E00144:68:H2LCKCCXX:6:2105:4674:28400 -
ST-E00144:68:H2LCKCCXX:1:1221:5252:20823 -
ST-E00144:68:H2LCKCCXX:3:2122:16489:7936 -
ST-E00144:68:H2LCKCCXX:7:1104:23624:71665-
ST-E00144:68:H2LCKCCXX:7:2110:23167:53259-
ST-E00144:68:H2LCKCCXX:1:2209:30323:71173-
ST-E00144:68:H2LCKCCXX:3:1119:25644:44011-
ST-E00144:68:H2LCKCCXX:1:1116:6785:62646 -
ST-E00144:68:H2LCKCCXX:4:1117:23025:55263-
ST-E00144:68:H2LCKCCXX:8:1219:13444:11171-
ST-E00192:51:H2KG3CCXX:3:2118:29836:57882+
ST-E00192:51:H2KG3CCXX:2:2215:17250:26238+
ST-E00192:51:H2KG3CCXX:2:2215:17260:26290+
ST-E00192:51:H2KG3CCXX:3:1101:29917:72614+
ST-E00192:51:H2KG3CCXX:1:1110:31764:20612+
ST-E00192:51:H2KG3CCXX:1:1201:7891:24163 +
ST-E00192:51:H2KG3CCXX:1:1218:5659:27503 +
ST-E00192:51:H2KG3CCXX:8:1109:29541:39054+
ST-E00192:51:H2KG3CCXX:1:2105:28263:71120+
ST-E00192:51:H2KG3CCXX:1:2105:28283:71120+
ST-E00192:51:H2KG3CCXX:3:1203:19270:64018+
ST-E00192:51:H2KG3CCXX:5:1214:28080:40952+
ST-E00192:51:H2KG3CCXX:2:2113:19026:7620 +
ST-E00192:51:H2KG3CCXX:2:1217:13241:6425 +
ST-E00192:51:H2KG3CCXX:8:1220:16671:59306+
ST-E00192:51:H2KG3CCXX:8:1220:16742:59992+
ST-E00192:51:H2KG3CCXX:2:2210:9414:25534 +
ST-E00192:51:H2KG3CCXX:6:2213:24568:17781+
ST-E00192:51:H2KG3CCXX:2:2202:4786:50639 +
ST-E00192:51:H2KG3CCXX:5:1108:25715:63859+
ST-E00192:51:H2KG3CCXX:3:1218:3263:4543 +
ST-E00192:51:H2KG3CCXX:1:1116:26121:67833+
ST-E00192:51:H2KG3CCXX:8:2119:28801:45892+
ST-E00192:51:H2KG3CCXX:2:2207:24812:40390+
ST-E00192:51:H2KG3CCXX:2:2207:24923:40443+
ST-E00192:51:H2KG3CCXX:6:2103:6166:54225 +
ST-E00192:51:H2KG3CCXX:6:1205:12611:49074-
ST-E00192:51:H2KG3CCXX:5:1108:6978:2680 -
ST-E00192:51:H2KG3CCXX:3:2223:11251:10116-
ST-E00192:51:H2KG3CCXX:3:2223:11343:10872-
ST-E00192:51:H2KG3CCXX:8:1204:24111:37700-
ST-E00192:51:H2KG3CCXX:1:2115:25624:11769-
ST-E00192:51:H2KG3CCXX:1:1202:28202:51430-
ST-E00192:51:H2KG3CCXX:1:2214:31267:49742-
ST-E00192:51:H2KG3CCXX:2:1221:28831:42183-
ST-E00192:51:H2KG3CCXX:2:1221:28780:42201-
ST-E00192:51:H2KG3CCXX:6:1105:7607:69942 -
ST-E00192:51:H2KG3CCXX:2:1118:3507:62945 -
ST-E00192:51:H2KG3CCXX:2:2223:16235:51764-

```
ST-E00192:51:H2KG3CCXX:2:1117:23512:5897 - ACGCTCTTGGGTGTCGGTAAATTCTTCCATTCCCAATTCTTTTTCTGGCTCCTGCATTTTAAATATTTTCTTCCAAAAGTTCACCAGTTCTTCCCAAAAGCACTACACCGCTTCTGCCGGAAGGACCCAGTCCAACTCTAGTGCATGGCT
ST-E00192:51:H2KG3CCXX:5:1212:10876:11751- GCTCTTGGGTGTCGGTAAATTCTTCCATTCCCAatTCTTTTTCTGGCTCCTGCATTTTAAATATTTTCTTCCAAAAGTTCACCAGtTCTTCCCAAAAGCACTACACCGCTTCTGCCGGAAGGACCCAGTCCAACTCTAGTGCATGGCTCA
ST-E00192:51:H2KG3CCXX:5:2111:16773:7198 - GCTCTtGGgTGTCGGTAAATTCTTCCATTCCCAATTCTTTTTCTGGCTCCTGCATTTTAAATATTTTCTTCCAAAAGTTCACCAGTTCTTCCCAAAAGCACTACACCGCTTcTGCCGGAAGGACCCAGTCCAACTCTAGTGCATGGCTCA
ST-E00192:51:H2KG3CCXX:5:1116:7303:13861 - CTCTTGGGTGTCGGTAAATTCTTCCATTCCCAATTCTTTTTCTGGCTCCTGCATTTTAAATATTTTCTTCCAAAAGTTCACCAGTTCTTCCCAAAAGCACTACACCGCTTCTGCCGGAAGGACCCAGTCCAACTCTAGTGCATGGCTCAC
ST-E00192:51:H2KG3CCXX:2:1222:12845:9571 - GTCGGTAAATTCTTCCATtCCCAATTCTTTTTCTGGCTCCTGCATTTTAAATATTTTCTTCCAAAAGTTCACCAGTTCTTCCCAAAAGCACTACACCGCTTCTGCCGGAAGGACCCAGTCCAACTCTAGTGCATGGCTCACAGACATCTC
ST-E00192:51:H2KG3CCXX:2:2222:13890:8323 - GTCGGTAAATTCTTCCATTCCCAATTCTTTTTCTGGCTCCTGCATTTTAAATATTTTCTTCCAAAAGTTCACCAGTTCTTCCCAAAAGCACTACACCGCTTCTGCCGGAAGGACCCAGTCCAACTCTAGTGCATGGCTCACAGACATCTC
ST-E00192:51:H2KG3CCXX:5:1221:19645:40864- TAAATTCTTCCATTCCCAATTCTTTTTCTGGCTCCTGCATTTTAAATATTTTCTTCCAAAAGTTCACCAGTTCTTCCCAAAAGCACTACACCGCTTCTGCCGGAAGGACCCAGTCCAACTCTAGTGCATGGCTCACAGACATCTCGCTCT
ST-E00192:51:H2KG3CCXX:2:2118:30871:3911 - ATTCTTTTTCTGGCTCCTgCATTTTAAaTATTTTCTTCCAAAAGTTCACCAGTTCTTCCCAAAAGCACTACACCGCTTCTGCCGGAAGGACCCAGTCCAACTCTAGTGCATGGCTCACAGACATCTCGCTCTGCTTTGAAATATAAGGCT
ST-E00192:51:H2KG3CCXX:3:1119:30983:42992- TTCTTTTTCTGGCTCCTGCATTTTAAATATTTTCTTCCAAAAGTTCACCAGTTCTTCCCAAAAGCACTACACCGCTTCTGCCGGAAGGACCCAGTCCAACTCTAGTGCATGGCTCACAGACATCTCGCTCTGCTTTGAAATATAAGGCTC
ST-E00192:51:H2KG3CCXX:1:1110:14489:22300- TCTGGCTCCTGCATTTTAAATATTTTCTTCCAAAAGTTCACCAGTTCTTCCCAAAAGCACTACACCGCTTCTGCCGGAAGGACCCAGTCCAACTCTAGTGCATGGCTCACAGACATCTCGCTCTGCTTTGAAATATAAGGCTCAGGTCTC
ST-E00192:51:H2KG3CCXX:1:1217:14925:68729- TGGCTCCTGCATTTTAAaTATtTtCTTCCaAAAGTTCACCAGTTCTTCCCAAAAGCACTACACCGCTTCTGCCGGAAGGACCCAGTCCAACTCTAGTGCATGGCTCACAGACATCTCGCTCTGCTTTGAAATATAAGGCTCAGGTCTCCG
ST-E00192:51:H2KG3CCXX:5:2106:30029:20629- GCTCCTGCATTTTAAATATTTTCTTCCAaaAGTTCACCAGTTCTTCCCAAAAGCACTACACCGCTTCTGCCGGAAGGACCCAGTCCAACTCTAGTGCATGGCTCACAGACATCTCGCTCTGCTTTGAAATATAAGGCTCAGGTCTCCGCC
ST-E00192:51:H2KG3CCXX:3:2102:31145:59658- CTCCTGCATTTTAaATATTTTCTTCCAAAAGTTCACCAGTTCTTCCCAAAaGCACTACACCGCTTCTGCCGGAAGGACCCAGTCCAACTCTAGTGCATGGCTCACAGACATCTCGCTCTGCTTTGAAATATAAGGCTCAGGTCTCCGCCA
ST-E00192:51:H2KG3CCXX:6:2112:30414:56036- TTtgaaATaTTttCTTCCAAtAGTTctccagTTCTtCcCaAAatCaCTacaCCGCttCTGCCGgAAGGACCCAGtCcAtCtctagTGCAcGGCTCACAgaCaTCTCGCTCTGCTTTGAAATATAAGGCTCaGGTCTCCGCCACAACaCCA
ST-E00192:51:H2KG3CCXX:2:1216:25177:38315- TTTAAATATTTTCTTCCAAAAGTTCACCAGTtCTTCCCAAAAGCACTACACCGCTTCTGCCGGAAGGACCCAGTCCAACTCTAGTGCATGGCTCACAGACATCTCGCTCTGCTTTGAAATATAAGGCTCAGGTCTCCGCCACAACACCAG
ST-E00192:51:H2KG3CCXX:5:2112:12083:23829- AATATTTTCTTCCAAAAGTTCACCAGTTCTTCCCAAAAGCACTACACCGCTTCTGCCGGAAGGACCCAGTCCAACTCTAGTGCATGGCTCACAGACATCTCGCTCTGCTTTGAAATATAAGGCTCAGGTCTCCGCCACAACACCAGATTT
```

chr3 16793207 + 6 chr3 16793260 + 0 DEL 5 4 53 0 0.823333333333333 0 1 0 1 chr3 16793063 198 chr3 16793312

TTTCTGTATTTTTTTAAGATGATTGTATTATATTTATTCTTTAAATTAAGATTGTAGGCAGAGCTTGCAGTGAGCTGAGATAGTGCCACTGCAGTCCCGCCTGGGCGAAAGAGCGAGACTCCGTCTAAATATATATATATATATATAGTAGTTAATTGTAATACTTGGTTTCTATTGTTAAGCCAACCTCTCATTCCTGA

```
16793080 16793100 16793120 16793140 16793160 16793180 16793200 16793220 16793240 16793260 16793280 16793300 16793320
chr3:16793063-16793323 tttctgtatttttttaagatgattgtattatatttattctttaaattaagattgtaggcagagcttgcagtgagctgagatagtgccactgcagtcccgcctgggcgaaagagcgagactccgtctaaaaatatatatatatatatatatatatatatatatatatatatatatatatatatatatatatatatggtagttaattgtaatacttggtttctattgttaagccaacctctcattcctgaaatgaaattaa

ST-E00144:68:H2LCKCCXX:7:2205:14682:32479+ TTAAGATGATTGTATTATATTTATTCTTTAAATTAAGATTGTAGGCAGAGCTTGCAGTGAGCTGAGATAGTGCCACTGCAGTCCCGCCTGGGCGAAAGAGCGAGACTCCGTCTAAATATATATATATATATATAGTAGTTAATTGTAATACT
ST-E00144:68:H2LCKCCXX:1:2218:5019:18907 + AGATGATTGTATTATATTTATTCTTTAAATTAAGATTGTAGGCAGAGCTTGCAGTGAGCTGAGATAGTGCCACTGCAGTCCCGCCTGGGCGAAAGAGCGAGACTCCGTCTAAATATATATATATATATATAGTAGttaATTGTaatactTGG
ST-E00144:68:H2LCKCCXX:4:2117:30810:38614+ TAAATTAAGATTGTAGGCAGAGCTTGCAGTGAGCTGAGATAGTGCCACTGCAGTCCCGCCTGGGCGAAAGAGCGAGACTCCGTCTAAATATATATATATATATATAGTAGTTAATTGTAATACTTGGTTTCTATTGTTAAGCCAACCTCTCA
ST-E00144:68:H2LCKCCXX:4:2117:29724:40284+ TAAATTAAGATTGTAGGCAGAGCTTGCAGTGAGCTGAGATAGTGCCACTGCAGTCCCGCCTGGGCGAAAGAGCGAGACTCCGTCTAAATATATATATATATATATaTaGtctTTaATTgTaAtacttggTaTatatTGttacGcCAacctCt
ST-E00144:68:H2LCKCCXX:4:2103:6745:51536 + AGATTGTAGGCAGAGCTTGCAGTGAGCTGAGATAGTGCCACTGCAGTCCCGCCTGGGCGAAAGAGCGAGACTCCGTCTAAATATATATATATATATATaGTAGTTAaTTGTAATACTTGGtTtCTATTGTTAAGCcaacCTctcaTTCCTGA
ST-E00144:68:H2LCKCCXX:4:2103:6613:51659 + AGATTGTAGGCAGAGCTTGCAGTGAGCTGAGATAGTGCCACTGCAGTCCCGCCTGGGCGAAAGAGCGAGACTCCGTCTAAATATATATATATATATATAGTAGTtAATTGTAATACTTgGTTTCTaTTGTTAAGCcAACCTCTCATtCctGa
ST-E00144:68:H2LCKCCXX:7:2214:8277:40478 - TtTCTgTATTTTTTTAAGAtgATTGTATTATaTTTATTCtTTAAaTTAAgATTGTAtGCAGAGCTTGCAGTGAGCTGAGATAGTGCCACTGCAgTcCCGCCTGGGCGAAAGAGCGAGACTCCGTCTAAATATATATATATATATATAGTAGT
ST-E00144:68:H2LCKCCXX:7:2214:8247:40495 - TTTCTgTATTTTTTTAaGATGaTTGTATTATATTTATTCTTTAAATTAAGATTGTAGGCAGAGCTTGCAGTGAGCTGAgATAGTGCCACTGCAGTCCCGCCTGGGCGAAAGAGCGAgaCTCCGTCTAAATATATATATATATATATaGTAGT

ST-E00192:51:H2KG3CCXX:3:1218:10064:45295+ AGAGCtTGCAGTGAGCTGAGATAGTGCCACTGCAGTCCcgCCTGGGCgaAAGAGCGAGACTCCGtCTAA****************************************************ATATATATATATATATAGTAGtTAATTGTAATACTTGGTTtCTATTGTTAAGCcaACCtctcaTTCctgAAtTGAAAaTAa
```

```
16793140 16793160 16793180 16793200 16793220 16793240 16793260 16793280 16793300 16793320 16793340 16793360 16793380 16793400
chr3:16793122-16793406 agagcttgcagtgagctgagatagtgccactgcagtcccgcctgggcgaaagagcgagactccgtctaaaaatatatatatatatatatatatatatatatatatatatatatatatatatatatatatatatatatatatatggtagttaattgtaatacttggtttctattgttaagccaacctctcattcctgaaatgaaattaatttggccatatgttatcatcttttcctactatgtttttaaatttagtttgcttatattttgtttaggatttttgtatctgtat

ST-E00144:68:H2LCKCCXX:8:1109:6745:54138 + ATATATATGGTAGTTAATTGTAATACTTGGTTTCTATTGTTAAGCCAACCTCTCATTCCTGAAATGAAATTAATTTGGCCATATGTTATCATCTTTTCCTACTATGTTTTTAAATTTAGTTTGCTTATATTTTGTTTAGGATTTTTGTAT
ST-E00144:68:H2LCKCCXX:8:1109:7272:54208 + ATATATATGGTAGTTAATTGTAATACTTGGTTTCTATTGTTAAGCCAACCTCTCATTCCTGAAATGAAATTAATTTGGCCATATGTTATCATCTTTTCCTACTATGTTTTTAAaTTTaGTTTGcTTATaTTTTGTTTAGGaTtTTTGT
ST-E00144:68:H2LCKCCXX:4:2206:29694:41638+ ATATGGTAGTTAATTGTAATACTTGGTTTCTATTGTTAAGCCAACCTCTCATTCCTGAAATGAAATTAATTTGGCCATATGTTATCATCTTTTCCTACTATGTTTTTAAATTTAGTTTGCTTATATTTTGTTTAGGATTTTTgTaTcTgT
ST-E00144:68:H2LCKCCXX:8:2106:18204:8130 + TAGTAGTTAATTGTAATACTTGGTTTCTATTGTTAAGCCAACCTCTCATTCCTGAAATGAAATTAATTTGGCCATATGTTATCATCTTTTCCTACTATGTTTTTAAATTTAGTTTGCTTATATTTTGTTTAGGATTTTTGTATCTGTAT
ST-E00144:68:H2LCKCCXX:7:2205:14682:32479- TATATATATATATAGTAGTTAATTGTAATACTTGGTTTCTATTGTTAAGCCAACCTCTCATTCCTGAAATGAAATTAATTTGGCCATATGTTATCATCTTTTCCTACTATGTTTTTAAATTTAGTTTGCTTATATTTTGTTTAGGATTTT

ST-E00192:51:H2KG3CCXX:3:1218:10064:45295+ AGAGCtTGCAGTGAGCTGAGATAGTGCCACTGCAGTCCcgCCTGGGCgaAAGAGCGAGACTCCGtCTAA****************************************************ATATATATATATATATAGTAGtTAATTGTAATACTTGGTTtCTATTGTTAAGCcaACCtctcaTTCctgAAtTGAAAaTAa
```

chr3 182445178 + 4 chr3 182445225 + 2 DEL 3 1 47 47 1 0 1 0 1 chr3 182445033 208 chr3 182445327

GGAGAATATCTTCACTGCCTCCTTCACAGGTTGTTGGCACAAGAAAATGAAATGGTATGCATTAAAACACCTGTAAAGCAATAACGATGCATATTATAATATTACAATCTAGATACAATCTAGATTAATAATATAATCTAGATACATATTATAATATTTTTATTTGTATTGTTTATAAAATGTCTCATAGGTGAAATTAGTCAAC

```
182445040 182445060 182445080 182445100 182445120 182445140 182445160 182445180
chr3:182445033-182445183 ggagaatatcttcactgcctccttcacaggttgttggcacaagaaaatgaaatggtatgcattaaaacacctgtaaaGCAATAACGATGCatattataatattacaatctagatacaatctagattaataatataatctagatacaatcta

ST-E00144:68:H2LCKCCXX:3:2211:3720:45347 + AAAATGAAATGGTATGCATTAAAACACCTGTAAAGCAATAACGATGCATATTATAATATTACAATCTAGATACAATCtAGATTAATAATATAATCTAGATACATATTA
ST-E00144:68:H2LCKCCXX:3:1218:14195:27169+ GCAtTAAAACACCTGTAAAGCAAtAACGATGCATATTATAATATTACAATCTAGATACAATCTAGATTAATAATAtAATCTAGATACA
ST-E00144:68:H2LCKCCXX:3:1218:14164:27222+ gCAatAAAACACcTGTAAAgcAaTAACgATgcATaTTATAATATTAcAAtctAGATAcAATCTAGATTAATAATATAATCTAGATACA
ST-E00144:68:H2LCKCCXX:3:1218:14185:27222+ GcATTAAAACACCTGTAAAGCAATAACGATGCATATTATAATATTACAATCTAGATACAATCTAGATTAATAATATaATCTAGATACA
ST-E00144:68:H2LCKCCXX:6:2115:22609:58269- ggtGAAtAtCgTCaCTGcctccttCACAGGTTgtctGCACAAtAAAATGAAATGGTATGCATTAAAACACctgtAAAGcaatAACGATGCATATTaTAATATTACAATCtAgATaCAaTCTAGATTAATAAtATAATCTagATACATATT
ST-E00144:68:H2LCKCCXX:6:2115:22467:58550- GGAGAaTATCTTCACTGCCTCCTTCACAGGTtGtTggCACAAGAAAATGAAATGGTATGCATTAAAACACcTGTAAAGCAATAACGATGCATATTATAATATTACAATCTAGATACAATCTAGATTAATAATATAATCTAGATACATATT
ST-E00144:68:H2LCKCCXX:7:2219:6856:61996 - TtCaCTGCCTCCTgCACAgGTTGTTGGCACAAGAAAATGAAATGGTATGCATTAAAACACCTGTAAAGCAATAaCGATGCATATTAtAATATTAcAATCTAGATACAATCTAGATTAATAATATAATCTAGATaCATATTA

ST-E00192:51:H2KG3CCXX:8:2219:13190:35098- GAGAATATCTTCACTGCCTCCTTCACAGGTTGTTGGcACAAGAAAATGAAATGGTATGCATTAAAACACCTGTAAAGCAATAACGATGCATATTATAATATTACAATCTAGATACAATCTAGATTAATAATATAATCTAGATACAATCTA
ST-E00192:51:H2KG3CCXX:8:2219:13220:35116- GAGAATATCTTCACTGCCTCCTTCACAGGTTGTTGGCACAAGAAAATGAAATGGTATGCATTAAAACACCTGTAAAGCAATAACGATGCATATTATAATATTACAATCTAGATACAATCTAGATTAATAATATAATCTAGATACAATCTA
ST-E00192:51:H2KG3CCXX:8:2219:13281:35116- GAGAATATCTTCACTGCCTCCTTCACAGGTTGTTGGCACAAGAAAATGAAATGGTATgCATTAAAACACCTGTAAAGCAATAACGATGCATATTATAATATTACAATCTAGATACAATCTAGATTAATAATATAATCTAGATACAATCTA
```

```
182445240 182445260 182445280 182445300 182445320
chr3:182445225-182445327 aatctagatacaatctagattaataatataatctagatacatattataatatTTTTATTTGTATTGTTTATAAAATGTCTCATAGGTGAAATTAGTCAAGTGG

ST-E00144:68:H2LCKCCXX:3:1218:14195:27169+ AATCTAGATACAATCTAGATTAATAATAtAATCTAGATACATATTATAATATTTTTATTTGTAtTGTTTAtAAAATGTCtcATAGGTGAAATTAGTCAAgTgG
ST-E00144:68:H2LCKCCXX:3:1218:14164:27222+ AAtctAGATAcAATCTAGATTAATAATATAATCTAGATACATATTATAATAtTTTTATtTGTaTTGTTTATAAAAggtctaatAggtgaaatTAgtcAAGTGG
ST-E00144:68:H2LCKCCXX:3:1218:14185:27222+ AATCTAGATACAATCTAGATTAATAATATaATCTAGATACATattATAATATTTTTATtTGTATTGTTtATAAAATGTCTCaTAgGTGAAATTAGTcAAGTGG
ST-E00144:68:H2LCKCCXX:3:2211:3720:45347 + atTGTTTATaAAATGTctcATAGgtG
```

chr3 193200880 + 3 chr7 8519225 - 3 CTX 19 21 32 29 0.989166666666667 0 1 0 1 chr7 8519105 240 chr3 193200763

AACTCAAGACGTCTTTCATATGTATAAATGTATATCTTTGTATATCTGTCTCTGCCCCCTTAAAGGCTTAGGAACAGTGAGCAACTTAGTGCAAAAAGCACTCCTAGCATCCAGATCGTGGGCCATTTTTTTAAGTGTATCAATCATTGATTCCTTTTATTTCTGAGGCTATCTCAGTGTATAGACATACTAGTTTGTTCAT

```
193200760 193200780 193200800 193200820 193200840 193200860 193200880 193200900 193200920 193200940 193200960 193200980 193201000 193201020
chr3:193200742-193201024 acaccattccttggaatagccaaagactggaacccaaaaatgcatcaacaggtgaatggatgaacaaactagtatgtctatacactgagatagcctcagaaataaaaggaatcaatgattgatacacttaaaaaaatggataaatcccaaaataactatgaactttgatcccccccaaaattaaccaaaaatatattataaaagacccaaagaatacatactgtaaaataccatttatgtataatattgagcctggaaatgaatctacagtgacagaaagctg

ST-E00144:68:H2LCKCCXX:6:1208:28131:64914+ ATAGCCAAAGACTGGAACCCAAAAATGCATCAACAGGTGAATGGATGAACAAACTAGTATGTCTATACACTGAGATAGCCTCAGAAATAAAAGGAATCAATGATTGATACACTTAAAAAAATGGATAAAtCcCAAAATAACTATGAACTT
ST-E00144:68:H2LCKCCXX:6:2209:20467:65530+ ATAGCCAAAGACTGGAACCCAAAAATGCATCAACAGGTGAATGGATGAACAAACTAGTATGTCTATACACTGAGATAGCCTCAGAAATAAAAGGAATCAATGATTGATACACTTAAAAAAATGGATAAATCcCAAAATAACTATGAACTT
ST-E00144:68:H2LCKCCXX:8:1218:23279:3875 + AAAGACTGGAACCCAAAAATGCATCAACAGGTGAATGGATGAACAAACTAGTATGTCTATACACTGAGATAGCCTCAGAAATAAAAGGAATCAATGATTGATACACTTAAAAAAATGGCCCACGATCTGGATGCTAGGAGTGCTTTTTGC
ST-E00144:68:H2LCKCCXX:8:1218:23350:3963 + AAAGACTGGAACCCAAAAATGCATCAACAGGTGAATGGATGAACAAACTAGTATGTCTATACACTGAGATAGCCTCAGAAATAAAAGGAATCAATGATTGATACACTTAAAAAAATGGCCCACGATCTGGATGCTAGGAGTGCTTTTTGC
ST-E00144:68:H2LCKCCXX:8:1218:23462:3981 + AAAGACTGGAACCCAAAAATGCATCAACAGGTGAATGGATGAACAAACTAGTATGTCTATACACTGAGATAGCCTCAGAAATAAAAGGAATCAATGATTGATACACTTAAAAAAATGGCCCACGATCTGGATGCTAGGAGTGCTTTTTGC
ST-E00144:68:H2LCKCCXX:6:2221:28598:36610+ CCCAAAAATGCATCAACAGGTGAATGGATGAACAAACTAGTATGTCTATACACTGAGATAGCCTcAGAAATAAAAGGAAtCAATGATTGATACACTTAAAAAAAtGgatAAATCccAAAATAACTATgAAATTTGAtCccCcccAAAATc
ST-E00144:68:H2LCKCCXX:6:2221:28658:36786+ CCCAAAAAtGCATCAACAGGtGAAtGGATGAaCAAACTAGtAtGTCTATaCACTGcGatAgCCtCAGAAAtAAAAGGAAtCaaTGATtGaTACACTTacaAAAATGgaTaAATcCCAaaAgaACaatgAACTTTgatCCCCCCCAaacTc
ST-E00144:68:H2LCKCCXX:4:2120:24020:69432+ AAATGCATCAACAGGTGAATGGATGAACAAACTAGTATGTCTATACACTGAGATAGCCTCAGAAATAAAAGGAATCAATGATTGATACACTTAAAAAAATGGATAAATCCCAAAATAACTATGAACTTTGATCCCCCCCAAAATTAACCA
ST-E00144:68:H2LCKCCXX:8:1124:5121:21790 + GTGAATGGATGAACAAACTAGTATGTCTATACACTGAGATAGCCTCAGAAATAAAAGGAATCAATGATTGATACACTTAAAAAAATGGATAAATCCCAAAATAACTATGAACTTTGATCCCCCCCAAAATTAACCAAAAATATATTATAA
ST-E00144:68:H2LCKCCXX:4:2117:32830:14547+ AAACTAGTATGTCTATACACTGAGATAGCCTCAGAAATAAAAGGAATCAATGATTGATACACTTAAAAAAATGGATAAATCCCAAAATAACTATGAACTTTGATCCCCCCCAAAATTAACCAAAAATATATTATAAAAGACCCAAAGAAT
ST-E00144:68:H2LCKCCXX:6:2106:17534:62805+ ACTGAGATAGCCTCAGAAATAAAAGGAATCAATGATTGATACACTTAAAAAAATGGATAAATCCCAAAATAACTATGAACTTTGATCCCCCCCAAAATTAACCAAAAATATATTATAAAAGACCCAAAGAATACATACTGTAAAATACCA
ST-E00144:68:H2LCKCCXX:4:1114:28729:1502 + AAGGAATCAATGATTGATACACTTAAAAAAATGGATAAATCCCAAAATAACTATGAACTTTGATCCCCCCCAAAATTAACCAAAAATATATTATAAAAGACCCAAAGAATACATACTGTAAAATACCATTTATGTATAATATTGAGCCT
ST-E00144:68:H2LCKCCXX:6:1221:16549:63701+ AATCAATGATTGATACACTTAAAAAAATGGATAAATCCCAAAATAACTATGAACTTTGATCCCCCCCAAAATTAACCAAAAATATATTATAAAAGACCCAAAGAATACATACTGTAAAATACCATTTATGTATAATATTGAGCCTGGAAA
ST-E00144:68:H2LCKCCXX:1:2107:20356:40091+ CAATGATTGATACACTTAAAAAAATGGATAAATCCCAAAATAACTATGAACTTTGATCCCCCCCAAAATTAACCAAAAATATATTATAAAAGACCCAAAGAATACATACTGTAAAATACCATTTATGTATAATATTGAGCCTGGAAATGA
ST-E00144:68:H2LCKCCXX:7:1208:16661:70575+ ATTGATACACTTAAAAAAATGGATAAATCCCAAAATAACTATGAACTTTGATCCCCCCCAAAATTAACCAAAAATATATTATAAAAGACCCAAAGAATACATACTGTAAAATACCATTTATGTATAATATTGAGCCTGGAAATGAATCTA
ST-E00144:68:H2LCKCCXX:6:1121:28192:56863- ATAGCCAAAGAcTgGAACCCAAAAATGCATCAACAgGTGAATGGATGAACAAACTAGTATGTCTATACACTGAGATAGCCTCAGAAATAAAAGGAATCAATGATTGATACACTTAAAAAAATGGATAAATCCCAAAATAACTATGAACTT
ST-E00144:68:H2LCKCCXX:1:2222:14144:54472- GACTGGAACCCAAAAATGCATCAACAGGTGAATGGATGAACAAACTAGTATGTCTATACACTGAGATAGCCTCAGAAATAAAAGGAATCAATGATTGATACACTTAAAAAAATGGATAAATCCCAAAAtAACTATGAACTTTGATCCCCC
ST-E00144:68:H2LCKCCXX:1:2222:14225:54472- GACTGGAACCCAAAAATGCATCAACAGGTGAATGGATGAACAAACTAGTATGTCTATACACTGAGATAGCCTCAGAAATAAAAGGAATCAATGATTGATACACTTAAAAAAATGGATAAATCCCAAAATAACTATGAACTTTGATCCCCC
ST-E00144:68:H2LCKCCXX:1:1208:8358:34782 - cacCAaAAATgcatcacaAGGTGAATGgatgAACAACcTAGtcTGTCTaTACAcTGaGATAGCctCAGaaATAAcaGgaATCAATGaTTGATACAcTTAAAAAAATGGATAaaTCCCAAAATAACTATGAACTTTGATCCCCCCCAAAAT
ST-E00144:68:H2LCKCCXX:3:2122:13819:24374- AAAATGCATCAACAGGTGAATGGATGAACAAACTAGTATGTCTATACACTGAGATAGCCTCAGAAATAAAAGGAATCAATGATTGATACACTTAAAAAAATGGATAAATCCCAAAATAACTATGAACTTTGATCCCCCCCAAAATTAACC
ST-E00144:68:H2LCKCCXX:1:1208:8379:34782 - cccCAAcacTgacTCaacaGGTGAATGGAtgAACAAACTAGTATGTCTATaCACTGAGATAGCCTCAGAAATAAAAGGAATCAATGATTGATACACTTAAAAAAATGGATAAATCCCAAAATAACTATGAACTTTGATCCCCCCCAAAAT
ST-E00144:68:H2LCKCCXX:3:1222:26496:51922- AAAcTagtatGtCaAtAcACtGAAAtAgcCtcAGAAataAAAGGAAtCAAtGaTTGATACACTTAAAAAAATGGaTAAATCCCAAAATaACtATGAACTTTGATCCCCCCCAAAATTAACCAAAAATATATTATAAAAGaCCCAAAGAAT
```

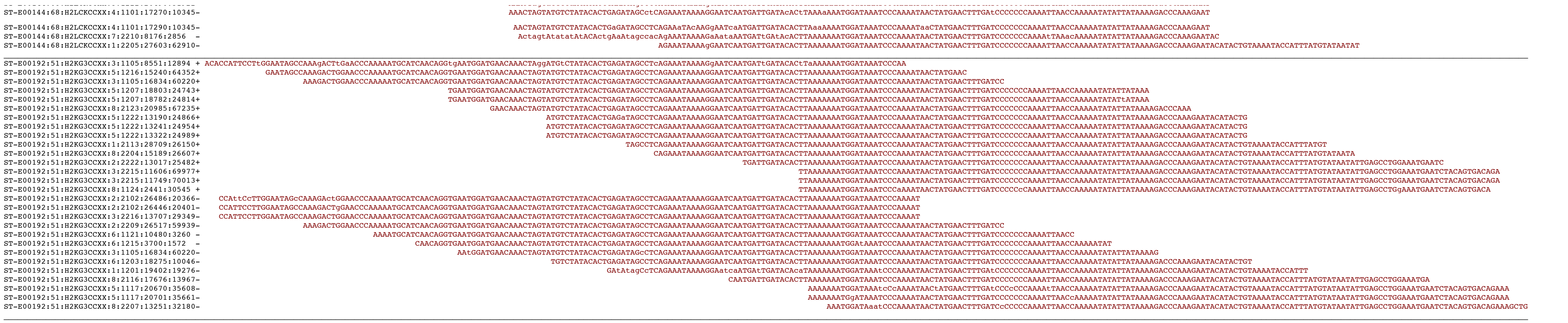

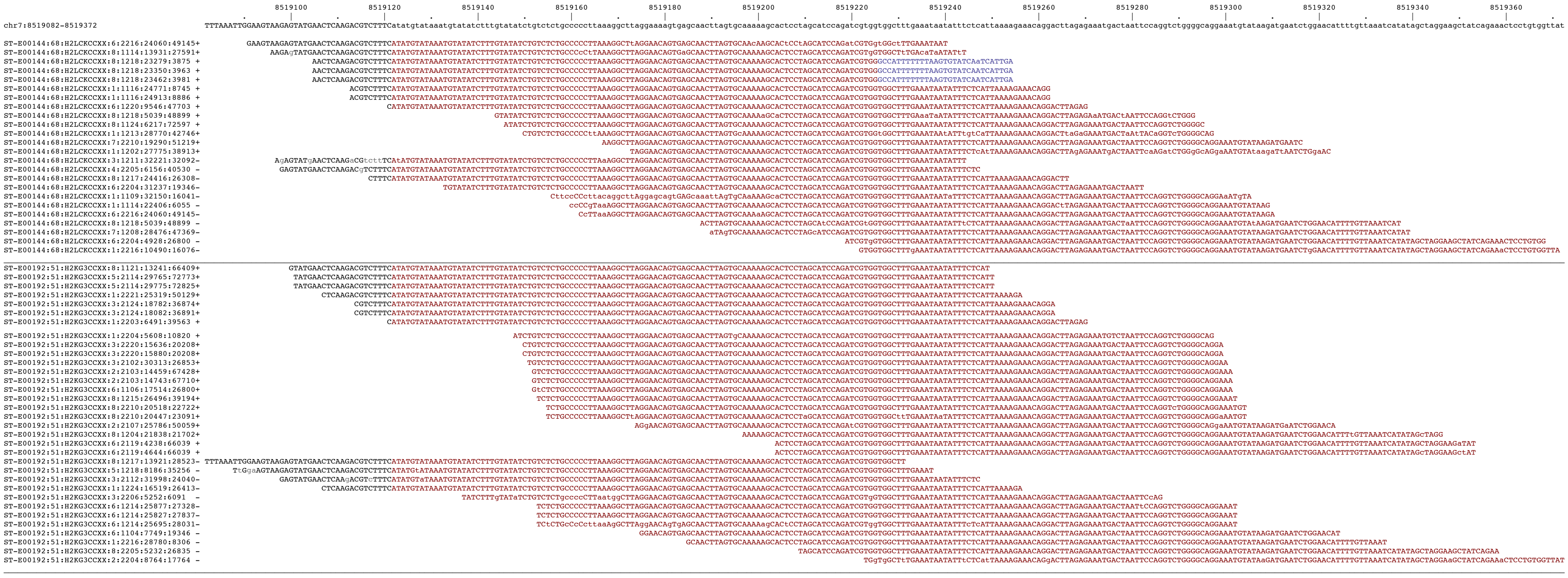

chr8 4183325 + 0 chr12 46404933 + 5 CTX 27 34 0 33 1 0 0.952857142857143 0 1 chr8 4183294 150 chr12 46405049

TTCCCATTTTAAATGCTGGCCCATTTAAAATGATACAGGCGCCTGCCACCACGACTGGCTAATTTTTTTGTATTTTTAGTAGAGACGGGGTTTCACCGTGTTAGCCAGGATGGTCTCGATCTCCTGACCTCGTGATCCTCCTGCCTCGGC

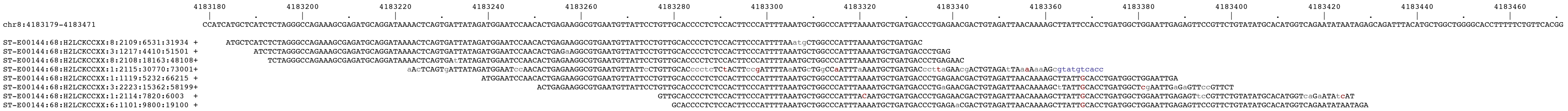

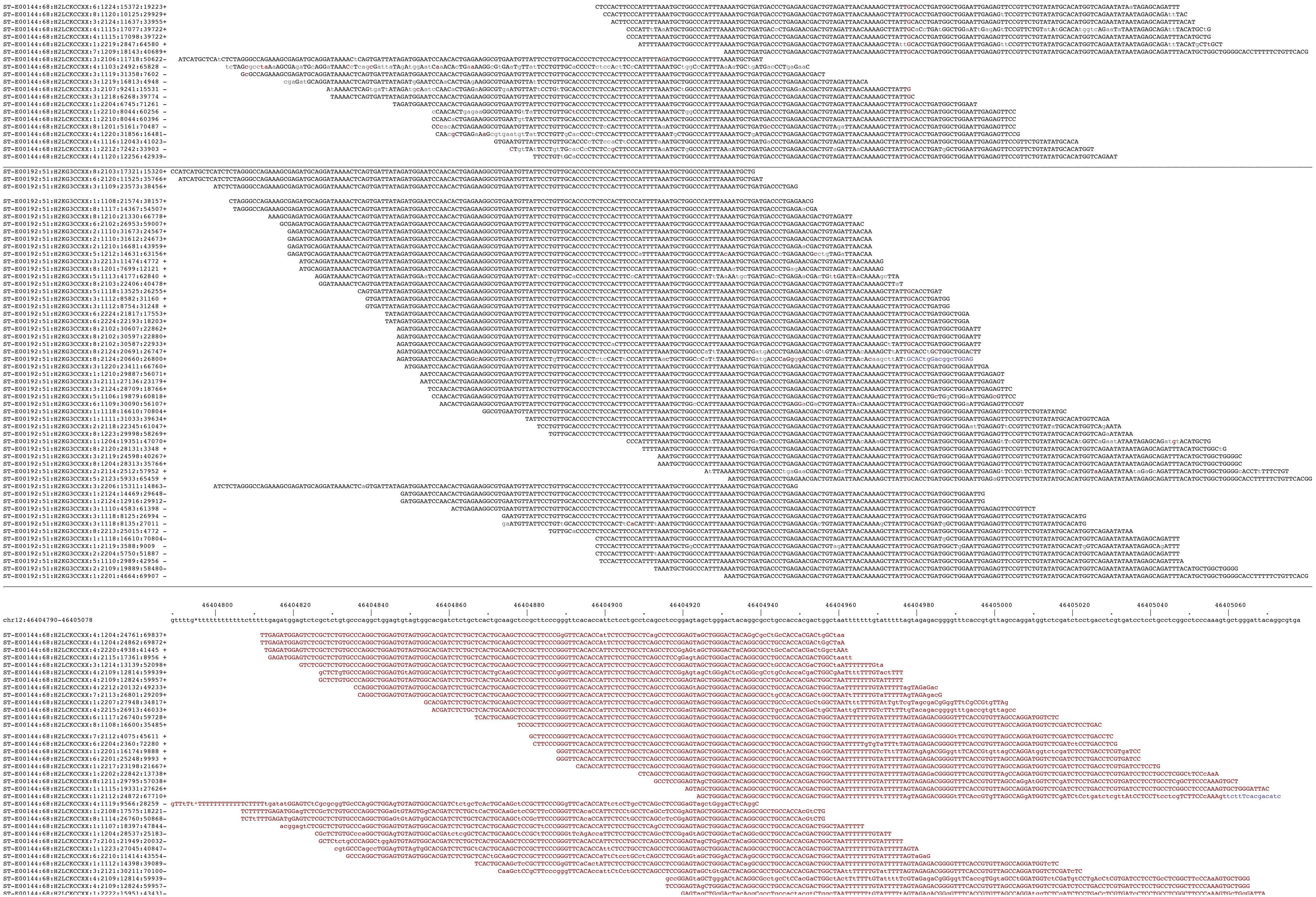
chr12:46404790-46405078
46404800
46404820
46404840
46404860
46404880
46404900
46404920
46404940
46404960
46404980
46405000
46405020
46405040
46405060

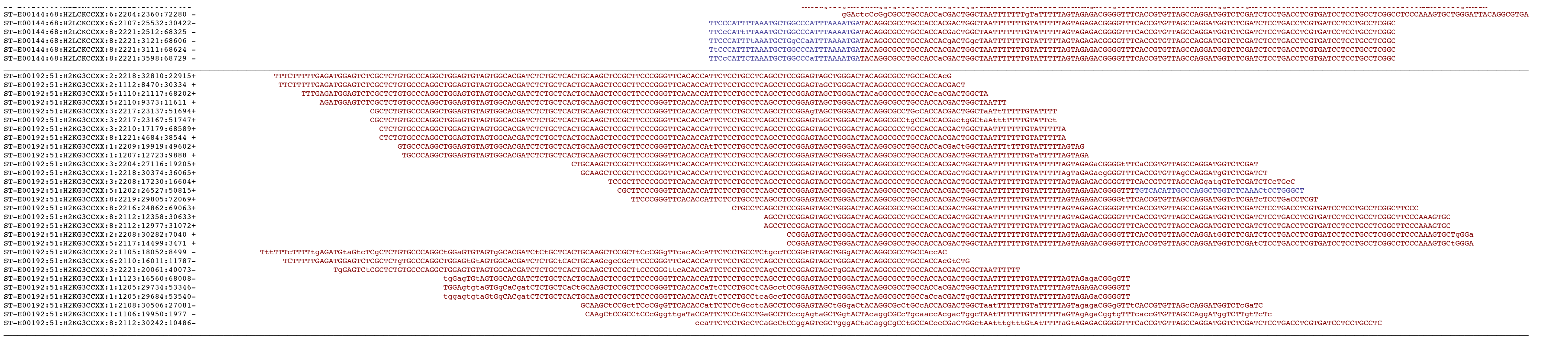

chr21 18908743 + 0 chr12 61947473 + 5 CTX 62 20 0 54 0.871428571428572 0 0.997488010961407 0 1 chr21 18908690 150 chr12 61947568

AGCTGTTTCTCCCATGTAGGCTTCATTGTTCGGGTTCTTTGCTTCTCTGGATGCCTCTATAGTGTCTATGGGCAGGAAAATTTGGAACTAAGTTCCAGTTTAGCAACTACTCAATTCCTAACCAATCTACATTGATACAATTATGTCTTC

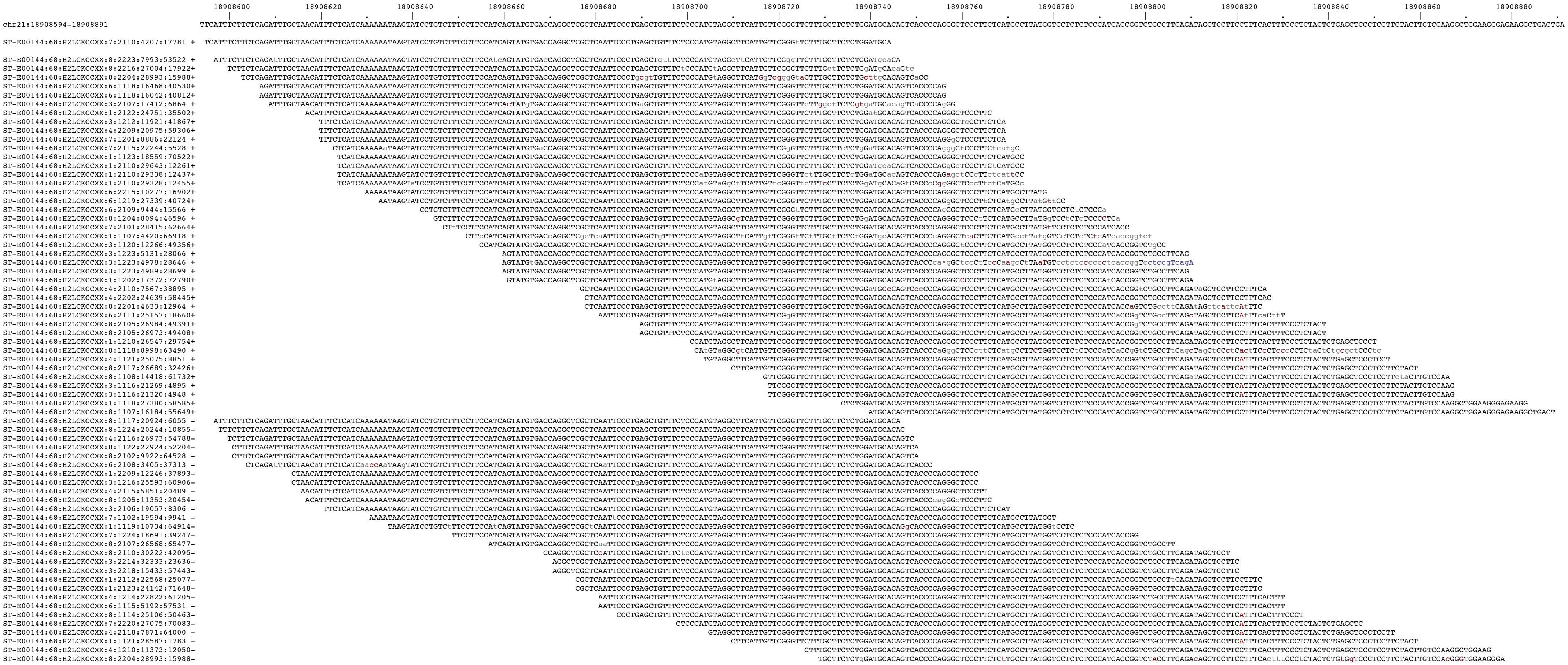

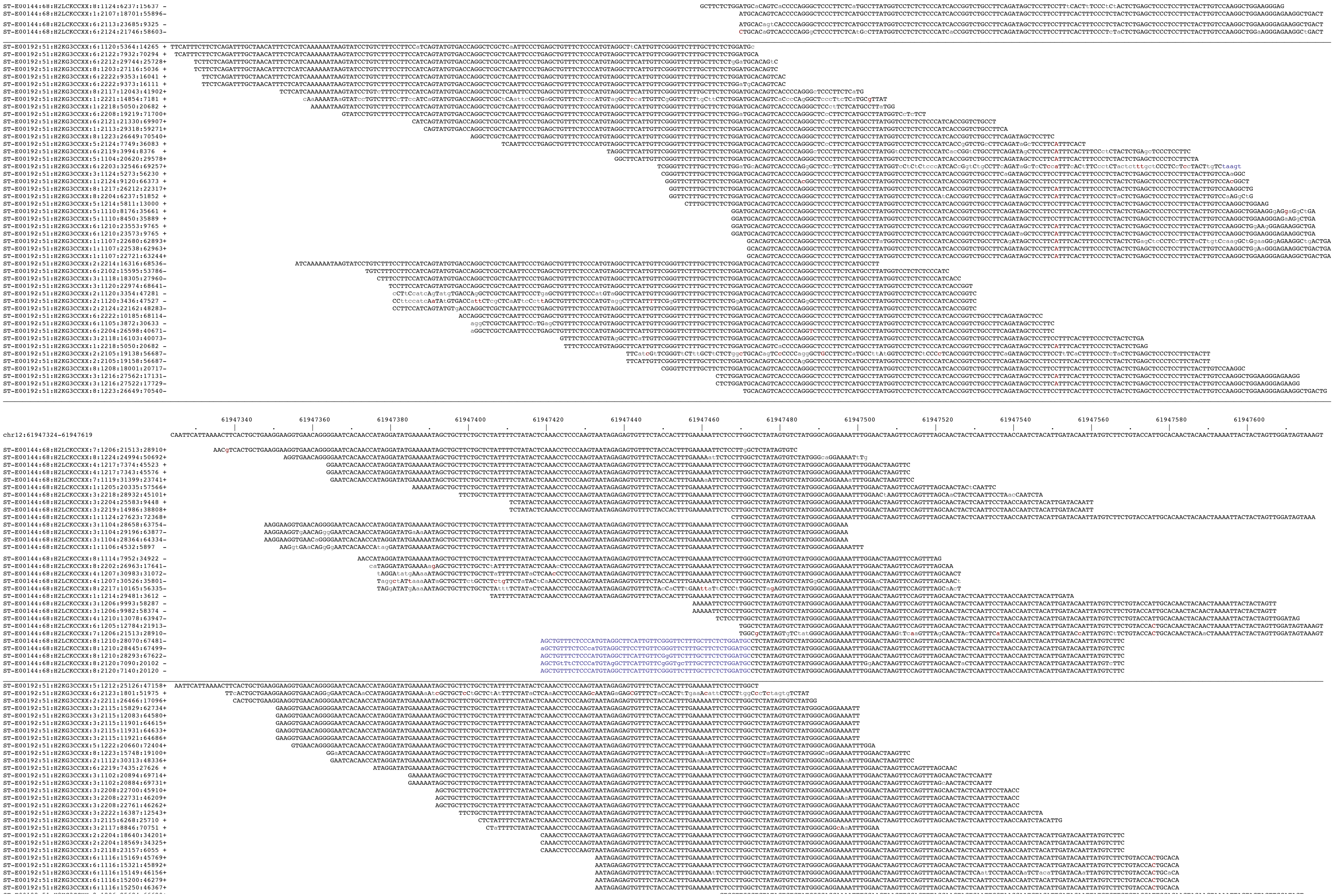

```
ST-E00192:51:H2KG3CCXX:2:1206:25684:66620+  [illegible]
ST-E00192:51:H2KG3CCXX:5:2122:8795:50270 - CAATTCATTAAAACTTCACTGCtGAAGGAAGGTGAACAGGGGAATCACAACCATAGGATATGAAAAATAGCTGCTTCTGCTCTATTTTCTATACTCAAACCTCCCCAAGTAATAGAGAGTGTTTCTACCACTTTGAAAAATTCTCCTTGGC
ST-E00192:51:H2KG3CCXX:3:2117:10409:48389-         TAAAACTTCACTGCTGAAGGAAGGTGAACAGGGGAATCACAACCATAGGATATGAAAAATAGCTGCTTCTGCTCTATTTTCTATACTCAAACCTCCCCAAGTAATAGAGAGTGTTTCTACCACTTTGAAAAATTCTCCTTGGCTCTATAGT
ST-E00192:51:H2KG3CCXX:1:1118:21391:13756-                 TGCTGAAGGAAGGTGAACAGGGGAATCACAACCATAGGATATGAAAAATAGCTGCTTCTGCTCTATTTTCTATACTCAAACCTCCCCAAGTAATAGAGAGTGTTTCTACCACTTTGAAAAATTCTCCTTGGCTCTATAGTGTCTATGGGC
ST-E00192:51:H2KG3CCXX:6:1108:8287:36417 -                     GAAGGAAGGTGAACAGGGGAATCACAACCATAGGATATGAAAAATAGCTGCTTCTGCTCTATTTTCTATACTCAAACCTCCCCAAGTAATAGAGAGTGTTTCTACCACTTTGAAAAATTCTCCTTGGCTCTATAGTGTCTATGGGCAGGAA
ST-E00192:51:H2KG3CCXX:6:1108:8196:36505 -                     GAAGGAAGGTGAACAGGGGAATCACAACCATAGGATATGAAAAATAGCTGCTTCTGCTCTATTTTCTATACTCAAACCTCCCCAAGTAATAGAGAGTGTTTCTACCACTTTGAAAAATTCTCCTTGGCTCTATAGTGTCTATGGGCAGGAA
ST-E00192:51:H2KG3CCXX:8:2221:6897:29297 -                        GGAAGGTGAACAGGGGAATCACAACCATAGGATATGAAAAATAGCTGCTTCTGCTCTATTTTCTATACTCAAACCTCCCCAAGTAATAGAGAGTGTTTCTACCACTTTGAAAAATTCTCCTTGGCTCTATAGTGTCTATGGGCAGGAAAAT
ST-E00192:51:H2KG3CCXX:8:2207:20021:38667-                         GAAggTGAACAGGGGAATCACAACCATAGGATATGAAAAATAGCTGCTTCTGCTCTATTTTCTATACTCAAACCTCCCCAAGTAATAGAGAGTGTTTCTACCACTTTGAAAAATTCTCCTTGGCTCTATAGTGTCTATGGGCAGGAAAATT
ST-E00192:51:H2KG3CCXX:2:1103:24609:72175-                          AAGGTGAACAGGGGAATCACAACCATAGGATATGAAAAATAGCTGCTTCTGCTCTATTTTCTATACTCAAACCTCCCCAAGTAATAGAGAGTGTTTCTACCACTTTGAAAAATTCTCCTTGGCTCTATAGTGTCTATGGGCAGGAAAATTT
ST-E00192:51:H2KG3CCXX:2:1103:24629:72175-                          AAGGTGAACAGGGGAATCACAACCATAGGATATGAAAAATAGCTGCTTCTGCTCTATTTTCTATACTCAAACCTCCCCAAGTAATAGAGAGTGTTTCTACCACTTTGAAAAATTCTCCTTGGCTCTATAGTGTCTATGGGCAGGAAAATTT
ST-E00192:51:H2KG3CCXX:3:2216:2553:49725 -                          AAGGTGAACAGGGGAATCACAACCATAGgATATGAAAAATAGCTGCTTCTGCTCTATTTTCTATACTCAAACCTCCCCAAGTAATAGAGAGTGTTTCTACCACTTTGAAAAATTCTCCTTGGCTCTATAGTGTCTATGGGCAGGAAAATTT
ST-E00192:51:H2KG3CCXX:2:1103:24629:72351-                                AAAAGgggAAtCaaAACCaaAcGATATGAcaaAcAGCTGCTTCTGCTCTAttttaTATACgCAAACCTCCCAAgTAAtaGAGAgTGTTtCTACCACTTTGAAAAAttCTCCTTgGCTCTATAGTGTCTATGGgCAGGAAAATTT
ST-E00192:51:H2KG3CCXX:5:1206:4857:55052 -                                AaCagaagAAtCAcAacCAaAGGaTattAaaAATAGcTGctTCTGCTctATTTTctATAcgCAAAcCTCCaAAGtAaTAGAGaGTGTTTctACcActtTGAAaAatTcTCaTTGGCTCTATAGTGTCTATGGgcAGGAAaATtTGGAACa
ST-E00192:51:H2KG3CCXX:6:2214:15027:43695-                                aAcAgggaaaTCAcatccAtaatAtATaAaaaATAgCTGCTccTGctCTATTTTcTATacTCaAacCTCcCAAGTaATAGAGAGTgTTTCTACCaaTtTGAAAatTTCTCCTTgGCTCTATAGTGTCTATGGGCAGGAAAATTTgGAcCT
ST-E00192:51:H2KG3CCXX:6:1221:19077:65828-                                 ACAGGgGAATCACAACCATAGGATATGAAAAATAGCTGCTTCTGCTCTATTTTCTATACTCAAACCTCCCCAAGTAATAGAGAGTGTTTCTACCACTTTGAAAAATTCTCCTTGGCTCTATAGTGTCTATGGGCAGGAAAATTTGGAACTA
ST-E00192:51:H2KG3CCXX:6:1221:18864:66127-                                 ACAGGGGAATCACAACCATAGGATATGAAAAATAGCTGCTTCTGCTCTATTTTCTATACTCAAACCTCCCCAAGTAATAGAGAGTGTTTCTACCACTTTGAAAAATTCTCCTTGGCTCTATAGTGTCTATGGGCAGGAAAATTTGGAACTA
ST-E00192:51:H2KG3CCXX:6:1221:18935:66954-                                 ACAGGGGAATCACAACCATAGGATATGAAAAATAGCTGCTTCTGCTCTATTTTCTATACTCAAACCTCCCCAAGTAATAGAGAGTGTTTCTACCACTTTGAAAAATTCTCCTTGGCTCTATAGTGTCTATGGGCAGGAAAATTTGGAACTA
ST-E00192:51:H2KG3CCXX:2:2119:11738:2698 -                                       GAATCACAACCATAGGATATGAAAAATAGCTGCTTCTGCTCTATTTTCTATACTCAAACCTCCCCAAGTAATAGAGAGTGTTTCTACCACTTTGAAAAATTCTCCTTGGCTCTATAGTGTCTATGGGCAGGAAAATTTGGAACTAAGTTCC
ST-E00192:51:H2KG3CCXX:8:1216:15839:57618-                                       GAATCACAACCATAGGATATGAAAAATAGCTGCTTCTGCTCTATTTTCTATACTCAAACCTCCCCAAGTAATAGAGAGTGTTTCTACCACTTTGAAAAATTCTCCTTGGCTCTATAGTGTCTATGGGCAGGAAAATTTGGAACTAAGTTCC
ST-E00192:51:H2KG3CCXX:3:2112:4106:25693 -                                          TCACAACCATAggATATGAAAAATAGCTGCTTCTGCTCTATTTTCTATACTCAAACCTCCCCAAGTAATAGAGAGTGTTTCTACCACTTTGAAAAATTCTCCTTGGCTCTATAGTGTCTATGGGCAGGAAAATTTGGAACTAAGTTCCAGT
ST-E00192:51:H2KG3CCXX:6:1117:30211:10609-                                          TCACAACCATAGGATATGAAAAATAGCTGCTTCTGCTCTATTTTCTATACTCAAACCTCCCCAAGTAATAGAGAGTGTTTCTACCACTTTGAAAAATTCTCCTTGGCTCTATAGTGTCTATGGGCAGGAAAATTTGGAACTAAGTTCCAGT
ST-E00192:51:H2KG3CCXX:2:1116:20072:20260-                                                                TCTGCTCTATTTTCTATACTCAAACCTCCCCAAGTAATAGAGAGTGTTTCTACCACTTTGAAAAATTCTCCTTGGCTCTATAGTGTCTATGGGCAGGAAAATTTGGAACTAAGTTCCAGTTTAGCAACTACTCAATTCCTAACCAATCTA
ST-E00192:51:H2KG3CCXX:2:1116:19909:20788-                                                                TTCTGCTCTATTTTCTATACTCAAACCTCCCCAAGTAATAGAGAGTGTTTCTACCACTTTGAAAAATTCTCCTTGGCTCTATAGTGTCTATGGGCAGGAAAATTTGGAACTAAGTTCCAGTTTAGCAACTACTCAATTCCTAACCAATCTA
ST-E00192:51:H2KG3CCXX:3:2117:8846:70751 -            GgCGACCACCGAGATCTACACTCTTTCCCTACACGACGCTCTTCCGATCTATTTTCTATACTCAAACCTCCCCAAGTAATAGAGAGTGTTTCTACCACTTTGAAAAATTCTCCTTGGCTCTATAGTGTCTATGGGCAGGAAAATTTGGA
ST-E00192:51:H2KG3CCXX:8:2203:31470:69995-                                                                   CTCTATTTTCTATACTCAAaCCTCCCCAAGTAATAGAGAGTGTTTCTACCACTTTGAAAAATTCTCCTTGGCTCTATAGTGTCTATggGCAGGAAAATTTGGAACTAAGTTCCAGTTTAGCAACTACTCAATTCCTAACCAATCTACATTG
ST-E00192:51:H2KG3CCXX:5:2107:24791:28259-                                                                      ATTTTCTATACTCAAACCTCCCCAAGTAATAGAGAGTGTTTCTACCACTTTGAAAAATTCTCCTTGGCTCTATAGTGTCTATGGGCAGGAAAATTTGGAACTAAGTTCCAGTTTAGCAACTACTCAATTCCTAACCAATCTACATTGATAC

ST-E00192:51:H2KG3CCXX:3:1210:28598:68852-                                                                        tTTCTATACTCAAACCTCCCCAAGTAATAGAGAGTGTTTCTACCACTTTGAAAAATTCTCCTTGGCTCTATAGTGTCTATGGGCAGGAAAATTTGGAACTAAGTTCCAGTTTAGCAACTACTCAATTCCTAACCAATCTACATTGATACAA
ST-E00192:51:H2KG3CCXX:3:1210:28618:68852-                                                                        tttCTATACTCAAACCtCCCAAGTAATAGAGAGTGTTTCTACCACTTTGAAAAATTCTCCTTGGCTCTATAGTGTCTATGGGCAGGAAAATTTGGAACTAAGTTCCAGTTTAGCAACTACTCAATTCCTAACCAATCTACATTGATACAA
ST-E00192:51:H2KG3CCXX:2:2109:6318:66444 -                                                                          TCTATaCTCAAaCCtcCCAAGTAATAGAGAGTGTTTCTACCACTTTGAAAAAttCTCCTTGGCTCTaTAGTGTCTATGGGCAGGAAAATTTGGAACTAAGTTCCAGTTTAGCAACTACTCAATTCCTAACCAATCTACATTGATACAATT
ST-E00192:51:H2KG3CCXX:8:1112:25400:51641-                                                                              ACTCAAACCTCCCCAAGTAATAGAGAGTGTTTCTACCACTTTGAAAAATTCTCCTTGGCTCTATAGTGTCTATGGGCAGGAAAATTTGGAACTAAGTTCCAGTTTAGCAACTACTCAATTCCTAACCAATCTACATTGATACAATTATGTC
ST-E00192:51:H2KG3CCXX:6:2123:1801:51975 -                                                                                                  TGTTTCTACCaCttTGAAaaAATTCTCCTTGGCTCTATAGTGTCTATGGGCAGGAAAATTTGGAACTAAGTTCCAGTTTAGCAACTACTCAATTCCGAACCAATCTACATTGATACAAtTATGTCTTCTGTACCACTGCACAACtACAACT
ST-E00192:51:H2KG3CCXX:8:1109:27187:36030-                                                                                                  TGTTTCTACCACTTTGAAAAATTCTCCTTGGCTCTATAGTGTCTATGGGCAGGAAAATTTGGAACTAAGTTCCAGTTTAGCAACTACTCAATTCCTAACCAATCTACATTGATACAATTATGTCTTCTGTACCATTGCACAACTACAACT
ST-E00192:51:H2KG3CCXX:5:2109:5831:70136 -                                                                                                   GTTTCTACCACTTTGAAAAATTCTCCTTGGCTCTATAGTGTCTATGGGCAGGAAAATTTGGAACTAAGTTCCAGTTTAGCAACTACTCAATTCCTAACCAATCTACATTGATACAATTATGTCTTCTGTACCATTGCACAACTACAACTA
ST-E00192:51:H2KG3CCXX:5:2110:6562:1537  -                                                                                                   gTTTCTACCACTTTGAAAAATTCTCCTTGGCTCTATAGTGTCTATGGGCAGGAAAATTTGGAACTAAGTTCCAGTTTAGCAACTACTCAATTCCTAACCAATCTACATTGATACAATTATGTCTTCTGTACCATTGCACAACTACAACTA
ST-E00192:51:H2KG3CCXX:5:2110:6420:1572  -                                                                                                   gTttCTACcaCTTtgAAAAATtCTCCTTGGCTCTATAgTgtCtATggGCAgGAAAATTTGgAaCTAAGTTCCAgTTTAGCAACTACTCAAcTCCTAACCaATCTaCATTgATACAATTATGTCTTCTGTACCATTGCaCaTTGCACAACTAcAACTA
ST-E00192:51:H2KG3CCXX:5:2110:6481:1608  -                                                                                                   gtttctACCActatGAAAAATTCCTtGGCTCTATAGTGTCTATGGGCAGGAAAATTTGGAACTAAGTTCCAGTTTAGCAACTACTCAATTCCTAACCAATCTACATTGATACAATTATGTCTTCTGTACCATTGCACAACTACAACTA
ST-E00192:51:H2KG3CCXX:3:2118:23157:6055 -                                                                                                     TTCTACCACTTTGAAAAATTCTCCTTGGCTCTATAGTGTCTATGGGCAGGAAAATTTGGAACTAAGTTCCAGTTTAGCAACTACTCAATTCCTAACCAATCTACATTGATACAATTATGTCTTCTGTACCATTGCACAACTACAACTAAA
ST-E00192:51:H2KG3CCXX:2:2115:3456:37331 -                                                                                                      TCTACCACTTTGAAAAATTCTCCTTGGCTCTATAGTGTCTATGGGCAGGAAAATTTGGAACTAAGTTCCAGTTTAGCAACTACTCAATTCCTAACCAATCTACATTGATACAATTATGTCTTCTGTACCATTGCACAACTACAACTAAAA
ST-E00192:51:H2KG3CCXX:1:2115:17585:9589 -                                                                                                                  TCTCCTTGGCTCTATAGTGTCTATGGGCAGGAAAATTTGGAACTAAGTTCCAGTTTAGCAACTACTCAATTCCTAACCAATCTACATTGATACAATTATGTCTTCTGTACCATTGCACAACTACAACTAAAATTACTACTAGTTGGATAG
ST-E00192:51:H2KG3CCXX:2:2211:26466:17096-                                                                                                                  TCTCCTTGGCTCTATAGTGTCTATGGGCAGGAAAATTTGGAACTAAGTTCCAGTTTAGCAACTACTCAATTCCTAACCAATCTACATTGATACAATTATGTCTTCTGTACCATTGCACAACTACAACTAAAATTACTACTAGTTGGATAG
```

---

chr12 112562745 + 7 chr12 112562789 + 0 DEL 21 24 39 0 1 0 0.915 0 1 chr12 112562599 184 chr12 112562825

TATTGCCCAGGCTGGTCTCTAATTCCTGAGCTCAAGAGATCCACCCGCCTTGGCCTCCATATTTGCAATTATAATCTGCAATGGAATCATAATTTTAAATTGCTGTATTGTTTGTAATATGTGCTTGGTTATACTTATATAATTATTCTCTCCTTTATTATACATGAGGTAAGAGAAGCTCAG

```
                           112562600           112562620           112562640           112562660           112562680           112562700           112562720           112562740           112562760           112562780           112562800           112562820           112562840           112562860           112562880
                                |         .         |         .         |         .         |         .         |         .         |         .         |         .         |         .         |         .         |         .         |         .         |         .         |         .         |         .         |         .         |         .
chr12:112562596-112562894     ttatattgcccaggctggtctctaattcctgagctcaagagatccacccgccttggcctccATATTTGCAATTATAATCTGCAATGGAATCATAATTTTAAATTGCTGTATTGTTTGTAATATGTGCTTGGTTATACTTATATAATTATAAGTTGGTTTGTAATATGTGCTTGGTTATACTTATATAattattctctcctttattatacatgaggtaagagaagctcagggaatccattcaggcagcaagtggagaaactagctctcaaacctgcatctcaggctctcattcttccac

ST-E00144:68:H2LCKCCXX:6:2124:23685:27433+  TATATTGCCCAGGCTGGTCTCTAATTCCTGAGCTCAAGAGATCCACCCGCCTTGGCCTCCATATTTGCAATTATAATCTGCAATGGAATCATAATTTtAAATTGCTGTATTGTTTGTAATATGTGCTTGGTTATACTTATATAATTATT
ST-E00144:68:H2LCKCCXX:8:2116:4704:18678 +    TATTGCCCAGGCTGGTCTCTAATTCCTGAGCTCAAGAGATCCACCCGCCTTGGCCTCCATATTTGCAATTATAATCTGCAATGGAATCATAATTTTAAATTGCTGTATTGTTTGTAATATGTGCTTGGTTATACTTATATAATTATTcT
ST-E00144:68:H2LCKCCXX:1:2115:13931:8288 +                GGTCTCTAATTCCTGAGCTCAAGAGATCCACCCGCCTTGGCCTCCATATTTGCAATTATAATCTGcAATGGAATCAcAAGTTTAAATcGCTGTCTTGTTTGTAATATGTGCTTGGTTATACTTATATAATTATTCTCTCCTTTATTATA
ST-E00144:68:H2LCKCCXX:7:1107:24030:68571+                     TAATTCCTGAGCTCAAGAGATCCACCCGCCTTGGCCTCCATATTTGCAATTATAATCTGCAATGGAATCATAATTTTAAATTGCTGTATTGTTTGTAATATGTGCTTGGTTATACTTATATAATTATAAGTTGGTTTGTAATATGTGCT
ST-E00144:68:H2LCKCCXX:1:2221:19199:15619+                           TGAGCTCAAGAGATCCACCCGCCTTGGCCTCCATATTTGCAATTATAATCTGCAATGGAATCATAATTTTAAATTGCTGTATTGTTTGTAATATGTGCTTGGTTATACTTATATAATTATAAGTTGGTTTGTAATATGTGCTTGGTTAT
ST-E00144:68:H2LCKCCXX:1:2221:19270:15988+                           TgAgctcAAGAGATCCACacgccttggCctCCATATTTGCAATTAtAATCTGCAATGgaATCATAATtTTAAATTGCTGTATtGtttgtAATATGtgcTTGGTTATACtTATATAATTAtAAGtagGTTTgTAATAtGtgcttggTgAT
ST-E00144:68:H2LCKCCXX:6:1215:9455:42165 +                                        CACCCGCCTTGGCCTCCATATTTGCAATTATAATCTGCAATGGAATCATAATTTTAAATTGCTGTATTGTTTGTAATATGTGCTTGGTTATACTTATATAATTATTCTCTCCTTTATTATACATGAGGTAAGAGAAGCTcAGGGAATCC
ST-E00144:68:H2LCKCCXX:6:1215:8358:42236 +                                        CACCCGCCTTGGCCTCCATATTTGCAATTATAATCTGCAATGGAATCATAATTTTAAATTGCTGTATTGTTTGTAATATGTGCTTGGTTATACTTATATAATTATTCTCtCCTTTATTATACATGAGGTAAGAGAAGCTCAGGGAAtCC
ST-E00144:68:H2LCKCCXX:4:1119:10713:19874+                                               CCATATTTGCAATTATAATCTGCAATGGAATCATAATTTTAAATTGCTGTATT*****************************************GTTTGTAATATGTGCTTGGTTATACTTATATAATTATTCTCTCCTTTATTATACATGAGGTAAGAGAAGCTCAGGGAATCCATTCAGGCAGCAAGT
ST-E00144:68:H2LCKCCXX:8:1118:7475:2276  +                                                          TGCAATGGAATCATAATTTTAAATTGCTGTATTGTTTGTAATATGTGCTTGGTTATACTTATATAATTATAAGTTGGTTTGTAATATGTGCTTGGTTATACTTATATAATTATTCTCTCCTTTATTATACATGAGGTAAGAGAAGCTCAG
ST-E00144:68:H2LCKCCXX:3:1222:16813:34518+                                                                            TATaTAaTTATAAGTTGGTTtGTAataTGTGCTTGGTtATACTTATATAATtatTcTCTCcTTtaTTATACATGAGGTAAGAGAAGCTCAgggAAtCcaTTCAGGCAGCAAttGGAGAaacTAGctctcAAACCTGCAtaccaGGctCTc
ST-E00144:68:H2LCKCCXX:2:1204:4085:21473 -    TattgCcCAgGctgGtCTCTaATTCCTGAGCTCAAGAgacCCcCCGCCTTGGCCtCCATATTTGCAATTaTAATCTGCAATGGAATCATAATTTTAAATTGCTGTATTGTTTGTAATATGTGCTTGGTTATACTTATATAATTATAT
ST-E00144:68:H2LCKCCXX:1:2108:12307:3225 -      tgCCCAGGCtGGTCTCTAATTCCTGAGCTCAAGAGATCCACCCGCCTTGGCCTCCATATTTGCAATTATAATCTGCAATGGAATCATAATTTTAAATTGCTGTATTGTTTGTAATATGTGCTTGGTTATACTTATATAATTATAAGTTG
ST-E00144:68:H2LCKCCXX:3:2107:31886:67903-   cTcGCCCAGGCtcGTCTCtAATTCCTGaGctcaaGaGaTCCACCCGCCTTGGCCTCCATATTTGCAATTATAATCTGCAATGGAATCATAATTTTAAATTGCTGTATTGTTTGTAATATGTGCTTGGTTATACTTATATAATTATTCTC
ST-E00144:68:H2LCKCCXX:7:1116:3882:66795 -                GGtCtCtaattccTgaGCTcaaGaGaTCaCCCGCcttggCtCCataTTTGCAATTATAATCTGCAaTGgaATCATAATTTTAAaTTGCTGTATTGTTTGTAATATGTGCTTGGTTAtACTTATATAATTATTctCTCCTTTATtATA
ST-E00144:68:H2LCKCCXX:6:2111:15575:14547-                                AGagATCCACCCGCCTTGGCCTCCATATTTGCAATTATAATCTGCAATGGAATCATAATTTTAAATTGCTGTATTGTTTGTAATATGTGCTTGGTTATACTTATATAATTATTCTCTCCTTTATTATACATGAGGTAAGAGAAGCTCAG
ST-E00144:68:H2LCKCCXX:1:2115:13931:8288 -                                  AGATCCACCCGCCTTGGCCTCCATATTTGCAATTATAATCTGCAATGGAATCATAATTTTAAATTGCTGTATTGTTTGTAATATGTGCTTGGTTATACTTATATAATTATTCTCTCCTTTATTATACATGAGGTAAGAGAAGCTCAGGG
ST-E00144:68:H2LCKCCXX:7:1107:24030:68571-                                                TTGGCCTCCATATTTGCAATTATAATCTGCAATGGAATCATAATTTTAAATTGCTGTATTGTTTGTAATATGTGCTTGGTTATACTTATATAATTATAAGTTGGTTTGTAATATGTGCTTGGTTATACTTATATAATTATTCTCTCCTT
ST-E00144:68:H2LCKCCXX:3:1120:17260:40636-                                                                 TCTGCAATGGAATCATAATTTTAAATTGCTGTATTGTTTGTAATATGTGCTTGGTTATACTTATATAATTATAAGTTGGTTTGTAATATGTGCTTGGTTATACTTATATAATTATTCTCTCCTTTATTATACATGAGGTAAGAGAAGCTC
ST-E00144:68:H2LCKCCXX:6:2216:7343:25921 -                                                                  CTGCAATGGAATCATAATTTTAAATTGCTGTATTGTTTGTAATATGTGCTTGGTTATACTTATATAATTATAAGTTGGTTTGTAATATGTGCTTGGTTATACTTATATAATTATTCTCTCCTTTATTATACATGAGGTAAGAGAAGCTCA
ST-E00144:68:H2LCKCCXX:4:2205:12550:17852-                                                                      GGAATCATAATTTTAAATTGCTGTATTGTTTGTAATATGTGCTTGGTTATACTTATATAATTATAAGTTGGTTTGTAATATGTGCTTGGTTATACTTATATAATTATTCTCTCCTTTATTATACATGAGGTAAGAGAAGCTCAGGGAATC
ST-E00144:68:H2LCKCCXX:4:1114:14144:1274 -                                                                                   TTGTTTGTAATATGTGCTTGGTTATACTTATATAATTATAAGTTGGTTTGTAATATGTGCTTGGTTATACTTATATAATTATTCTCTCCTTTATTATACATGAGGTAAGAGAAGCTCAGGGAATCCATTCAGGCAGCAAGTGGAGAAACT
ST-E00144:68:H2LCKCCXX:6:2223:12165:10749-                                                                                         ATGTgCTTGGTTataCTTATATAATTATAAGTTgGTTTGTaatagGTtCTTGGTTATAaTTATATAATTATTCTCTCCTTTATTATACATGaGgTAAGAGAAGCTCAGGGAAtCCATTCAGGCAGCAAGTGGAGAAACTAGCTCTCAAAC
ST-E00144:68:H2LCKCCXX:6:2206:21797:49830-                                                                                              TATATaATTATAAGTTGGTTtGTacTaTGTGCTtGGTTATACTTATATAATTCTTCTCTCCTTTATTATACATGaGGtAAGaGAAGCtCAGGGAATCcaTTCaGGCAGCAAGTGGAGAAACTAGCTCTCAAACCTGCATCTCagGCTCTC

ST-E00192:51:H2KG3CCXX:2:2116:2187:61222 +        cCAGGcTGGTCTCTAATTCCTGAGCTCAAGAGATCCACACGcCTtGGCcTCCATATTTGCAatTATAATCTGCAAtGGAATCATAATTTTAAaTTGCTGTATTGTTTGtAATATGTGCTgGgtTATAcTtataTAATTATAaGTtGGtT
ST-E00192:51:H2KG3CCXX:8:1206:31389:8147 +                          GgtcAAGAGATcCACCCGcCTTGGCCTCcATATTTgCaATTaTAATCtGCaATGGaatCATAGtttTaAATTGCTGTATtGTgTGTAaTaTGTGCttGGTTATACTTATATgATgatAcgTtGcaTTGTaaTATGTGtttGGTTATacT
ST-E00192:51:H2KG3CCXX:8:2105:27116:58796+                                  ATCCACCCGCCTTGGCCTCCATATTTGCAATTATAATCTGCAATGGAATCATAATTTTAAATTGCTGTATTGTTTGTAATATGTGCTTGGTTATACTTATATAATTATAAGTTGGTTTGTAATATGTGCTTGGTTATACTTATATAATT
ST-E00192:51:H2KG3CCXX:3:1202:30810:64281+                                          CCTTGGCCTCCATATTTGCAATTATAATCTGCAATGGAATCATAATTTTAAATTGCTGTATTGTTTGTAATATGTGCTTGGTTATACTTATATAATTATAAGTTGGTTTGTAATATGTGCTTGGTTATACTTATATAATTATTCTCC
ST-E00192:51:H2KG3CCXX:3:1202:30729:64492+                                          CCTTGGCCTCCATATTTGCAATTATAATCTGCAATGGAATCATAATTTTAAATTGCTGTATTGTTTGTAATATGTGCTTGGTTATACTTATATAATTATAAGTTGGTTTGTAATATGTGCTTGGTTATACTTATATAATTATTCTCC
ST-E00192:51:H2KG3CCXX:8:2216:28750:67604+                                                            GCAATTATAATCTGCAATGGAATCATAATTTTAAATTGCTGTATTGTTTGTAATATGTGCTTGGTTATACTTATATAATTATAAGTTGGTTTGTAATATGTGATTGGTTATACTTATATAATTATTcTCTCCTTTATTATACATGAGcta
ST-E00192:51:H2KG3CCXX:3:2211:12885:53944+                                                              AATTATAATCTGCAATGGAATCATAATTTTAAATTGCTGTATTGTTTGTAATATGTGCTTGGTTATACTTATATAATTATAAGTTGGTTTGTAATATGTGCTTGGTTATACTTATATAATTATTCTCTCCTTTATTATACATGAGGTAAG
ST-E00192:51:H2KG3CCXX:1:2212:31094:34043+                                                                                   TTTAAATTGCTGTATTGTTTGTAATATGTGCTTGGTTATACTTATATAATTATAAGTTGGTTTGTAATATGTGCTTGGTTATACTTATATAATTATTCTCTCCTTTATTATACATGAGGTAAGAGAAGCTCAGGGAATCCATTCAGGCAG
ST-E00192:51:H2KG3CCXX:2:1216:11617:13949+                                                                                            CTGTATTGTTTGTAATATGTGCTTGGTTATACTTATATAATTATAAGTTGGTTTGTAATATGTGCTTGGTTATACTTATATAATTATTCTCTCCTTTATTATACATGAGGTAAGAGAAGCTCAGGGAATCCATTCAGGCAGCAAGTGGAG
ST-E00192:51:H2KG3CCXX:8:1209:17016:2733 +                                                                                                          GCTTGGTTATACTTATATAATTATAAGTTGGTTTGTAATATGTGCTTGGTTATACTTATATAATTATTCTCTCCTTTATTATACATGAGGTAAGAGAAGCTCAGGGAATCCATTCAGGCAGCAAGTGGAGAAACTAGCTCTCAAACCTGC
ST-E00192:51:H2KG3CCXX:8:2216:13890:41902+                                                                                                              TTATACTTATATAATTATAAGTTGGTTTGTAATATGTGCTTGGTTATACTTATATAATTATTCTCTCCTTTATTATACATGAGGTAAGAGAAGCTCAGGGAATCCATTCAGGCAGCAAGTGGAGAAACTAGCTCTCAAACCTGCATCTCA
ST-E00192:51:H2KG3CCXX:6:1216:25958:55245+                                                                                                                ATATAATTATAAGTTGGTTTGTAATATGTGCTTGgTTATACTTAtATATTATTCTCTCCTtTATTATACATGAGGTAAGAGAAGCTCAGGGAATCCATTCAGGCAGCAAGTGGAGAAACTAGCTCTCAAACTGCATCTCAGGcTgtCA
ST-E00192:51:H2KG3CCXX:5:2120:29491:8657 +                                                                                                                       TAAGTTGGTTTGTAATATGTGCTTGGTTATACTTATATAATTATTCTCTCCTTTATTATACATGAGGTAAGAGAAGCTCAGGGAATCCATTCAGGCAGCAAGTGGAGAAACTAGCTCTCAAACCTGCATCTCAGGCTCTCATTCTTCCAC
ST-E00192:51:H2KG3CCXX:8:2216:23401:43150- TTATaTTGCCCAGGCTGGTCTCTAATTCCTGAGCTCAAGAGATCCACCCGCCTTGGCCTCCATATTTGCAATTATAATCTGCAATGGAATCATAATTTTAAATTGCTGTATTGTTTGTAATATGTGCTTGGTTATACTTATATAATTATAT
ST-E00192:51:H2KG3CCXX:5:1109:11434:45172-       CCAGGCTGGTCTCTAATtTCCTGAGCTCAAGAGATCCACCCGCCTTGGCCTCCATATTTGCAATTATAATCTGCAATGGAATCATAATTTTAAATTGCTGTATTGTTTGTAATATGTGCTTGGTTATACTTATATAATTATAAGTTGGT
ST-E00192:51:H2KG3CCXX:8:2206:12916:67358-            TGGTCTCTAATTCCTGAGCTCAAGAGATCCACCCGCCTTGGCCTCCATATTTGCAATTATAATCTGCAATGGAATCATAATTTTAAATTGCTGTATTGTTTGTAATATGTGCTTGGTTATACTTATATAATTATAAGTTGGTTTGTAAT
ST-E00192:51:H2KG3CCXX:1:2213:23695:11312-             GTCTCTAATTCCTGAGCTCAAGAGATCCACCCGCCTTGGCCTCCATATTTGCAATTATAATCTGCAATGGAATCATAATTTTAAATTGCTGTATTGTTTGTAATATGTGCTTGGTTATACTTATATAATTATAAGTTGGTTTGTAATAT
ST-E00192:51:H2KG3CCXX:1:1203:13809:70804-                                          GCCTTGGCCTCCatATTTGCAATTATAATCTGCAATGGAATCATAATTTTAAATTGCTGTATT*****************************************GTTTGTAATATGTGCTTGGTTATACTTATATAATTATTCTCTCCTTTATTATACATGAGGTAAGAGAAGCTCAGGGAATCCATTCA
ST-E00192:51:H2KG3CCXX:3:1105:30587:10521-                                                              TGCAATTAtaATCTGCAATGGAATCATAATTTTAAATtGCTGTATT*****************************************GTTTGTAATATGTGCTTGGTTATACTTATATAATTATTCTCTCCTTTATTATACATGAGGTAAGAGAAGCTCAGGGAATCCATTCAGGCAGCAAGTGGAGAAAC
```

```
                                  112562660           112562680           112562700           112562720           112562740           112562760           112562780           112562800           112562820           112562840           112562860           112562880           112562900           112562920
                            .         |         .         |         .         |         .         |         .         |         .         |         .         |         .         |         .         |         .         |         .         |         .         |         .         |         .         |         .
chr12:112562645-112562936    gccttggcctccATATTTGCAATTATAATCTGCAATGGAATCATAATTTTAAATTGCTGTATTGTTTGTAATATGTGCTTGGTTATACTTATATAATTATAAGTTGGTTTGTAATATGTGCTTGGTTATACTTATATAattattctctcctttattatacatgaggtaagagaagctcagggaatccattcaggcagcaagtggagaaactagctctcaaacctgcatctcaggctctcattcttccactgcaAAAAAGCAAAGTGCTCAAAAATATGGTCACAGATCTG

ST-E00144:68:H2LCKCCXX:4:1119:10713:19874+       CCATATTTGCAATTATAATCTGCAATGGAATCATAATTTTAAATTGCTGTATT*****************************************GTTTGTAATATGTGCTTGGTTATACTTATATAATTATTCTCTCCTTTATTATACATGAGGTAAGAGAAGCTCAGGGAATCCATTCAGGCAGCAAGT
ST-E00144:68:H2LCKCCXX:8:1118:7475:2276  +                         TGCAATGGAATCATAATTTTAAATTGCTGTATTGTTTGTAATATGTGCTTGGTTATACTTATATAATTATAAGTTGGTTTGTAATATGTGCTTGGTTATACTTATATAATTATTCTCTCCTTTATTATACATGAGGTAAGAGAAGCTCAG
ST-E00144:68:H2LCKCCXX:3:1222:16813:34518+                                           TATaTAaTTATAAGTTGGTTtGTAataTGTGCTTGGTtATACTTATATAATtatTcTCTCcTTtaTTATACATGAGGTAAGAGAAGCTCAgggAAtCcaTTCAGGCAGCAAttGGAGAaacTAGctctcAAACCTGCAtaccaGGctCTc
ST-E00144:68:H2LCKCCXX:1:2109:2705:66584 +                                                      AGTTGGTTTGTAATATGTGCTTGGTTATAatTATATAATTATTCTCTCCTTTATTATACATGAGGTAAGAGAAGCTCAGGGAATCCATTCAGGCAGCAAgTGGAGGaACTAGCTctcAAACCTGCATCTCAGGCTCTCATTcTTccacTG
ST-E00144:68:H2LCKCCXX:1:2101:9201:8851  +                           TGGAATCATAATTTTAAATTGCTGTATTGTTTGTAATATGTGCTTGGTtTATAcTTATATAATTATTCTCTCCTTTATTATACATGAGGTAAGAGAAGctcAGggAAtccAttcAGGCAacAgTggagaAACtAgctcTCAAAccTGaAT
ST-E00144:68:H2LCKCCXX:7:1205:22447:13369+                                                   TATTGTTTGTAATATGTGCTTGGTTATACtTATATAATTATTCTCtCCTTTATTATACATGAGGTAAGAGAAGCTCAGGGAAtcCATTCAGGcAGCAAGTgGAGAAACTAGCtcgcAAACcTGCATCTCAGgCTCTCATTCTTCgaCTGC
ST-E00144:68:H2LCKCCXX:4:1211:32211:21667+                                                                    GTTATACTTATATAATTATTCTCTCCTTTATTATACATGAGGTAAGAGAAGCTCAGGGAATCCATTCAGGCAGCAAGTGGAGAAACTAGCTCtcAAACCTGCATcTCAGGCTctcATTCTTcaActGcaAAAAAgAAAGTGctCAAAAA
ST-E00144:68:H2LCKCCXX:1:2119:27735:16059+                                                                       TATACTTATATAATTATTCTCTCCTTTATTATACATGAGGTAAGAGAAGCTCAGGGAATCCATTCAGGCAGCAAGTGGAGAAACTAGCTCTCAAACCTGCATCTCAGGCTCTCATTCTTCCACTGCAAAAAAGCAAAGTGCTCAAAAAT
ST-E00144:68:H2LCKCCXX:8:2114:18722:42077+                                                                                  ATTATTCTCTCCTTTATTATACATGAGGTAAGAGAAGCTCAGGGAATCCATTCAGGCAGCAAGTGGAGAAACTAGCTCTCAAACCTGCATCTCAGGCTCTCATTCTTCCACTGCAAAAAAGCAAAgtGCTCAAAAATATGGTCACAGAT
ST-E00144:68:H2LCKCCXX:8:1112:17169:30703+                                                                                    TTCTCTCCTTTATTATACATGAGGTAAGAGAAGCTCAGGGAATCCATTCAGGCAGCAAGTGGAGAAACTAGCTCTCAAACCTGCATCTCAGGCTCTCATTCTTCCACTGCAAAAAAGCAAAGTGCTCAAaAAATATGgTcACAGATCTG
ST-E00144:68:H2LCKCCXX:7:1107:24030:68571-   TTGGCCTCCATATTTGCAATTATAATCTGCAATGGAATCATAATTTTAAATTGCTGTATTGTTTGTAATATGTGCTTGGTTATACTTATATAATTATAAGTTGGTTTGTAATATGTGCTTGGTTATACTTATATAATTATTCTCTCCTT
ST-E00144:68:H2LCKCCXX:3:1120:17260:40636-                   TCTGCAATGGAATCATAATTTTAAATTGCTGTATTGTTTGTAATATGTGCTTGGTTATACTTATATAATTATAAGTTGGTTTGTAATATGTGCTTGGTTATACTTATATAATTATTCTCTCCTTTATTATACATGAGGTAAGAGAAGCTC
ST-E00144:68:H2LCKCCXX:6:2216:7343:25921 -                    CTGCAATGGAATCATAATTTTAAATTGCTGTATTGTTTGTAATATGTGCTTGGTTATACTTATATAATTATAAGTTGGTTTGTAATATGTGCTTGGTTATACTTATATAATTATTCTCTCCTTTATTATACATGAGGTAAGAGAAGCTCA
ST-E00144:68:H2LCKCCXX:4:2205:12550:17852-                        GGAATCATAATTTTAAATTGCTGTATTGTTTGTAATATGTGCTTGGTTATACTTATATAATTATAAGTTGGTTTGTAATATGTGCTTGGTTATACTTATATAATTATTCTCTCCTTTATTATACATGAGGTAAGAGAAGCTCAGGGAATC
ST-E00144:68:H2LCKCCXX:4:1114:14144:1274 -                                     TTGTTTGTAATATGTGCTTGGTTATACTTATATAATTATAAGTTGGTTTGTAATATGTGCTTGGTTATACTTATATAATTATTCTCTCCTTTATTATACATGAGGTAAGAGAAGCTCAGGGAATCCATTCAGGCAGCAAGTGGAGAAACT
ST-E00144:68:H2LCKCCXX:6:2223:12165:10749-  [illegible]
```

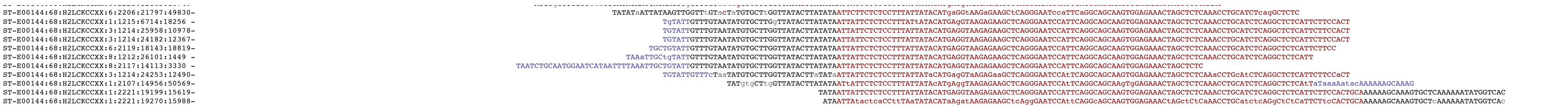

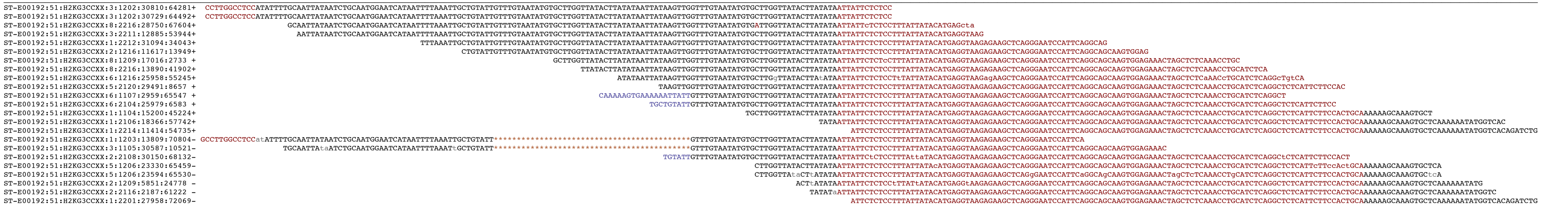

chr3 124910758 - 0 chr2 82709281 + 6 CTX 67 26 0 66 0.88 0 1 0 1 chr3 124910823 150 chr2 82709364

TTCCGAACCACGAGGACTGTTCTGACAATTATTGACTTTCAGGAGATTGCTCATTTGTGGTTTGTGAAGACTGAATTTTGCTTGTCAAAGTGGATGTAAGAGGGGTTTTAACTTTTGAGTTATTGCTAAGTGGTGGAAATGGATTCTATG

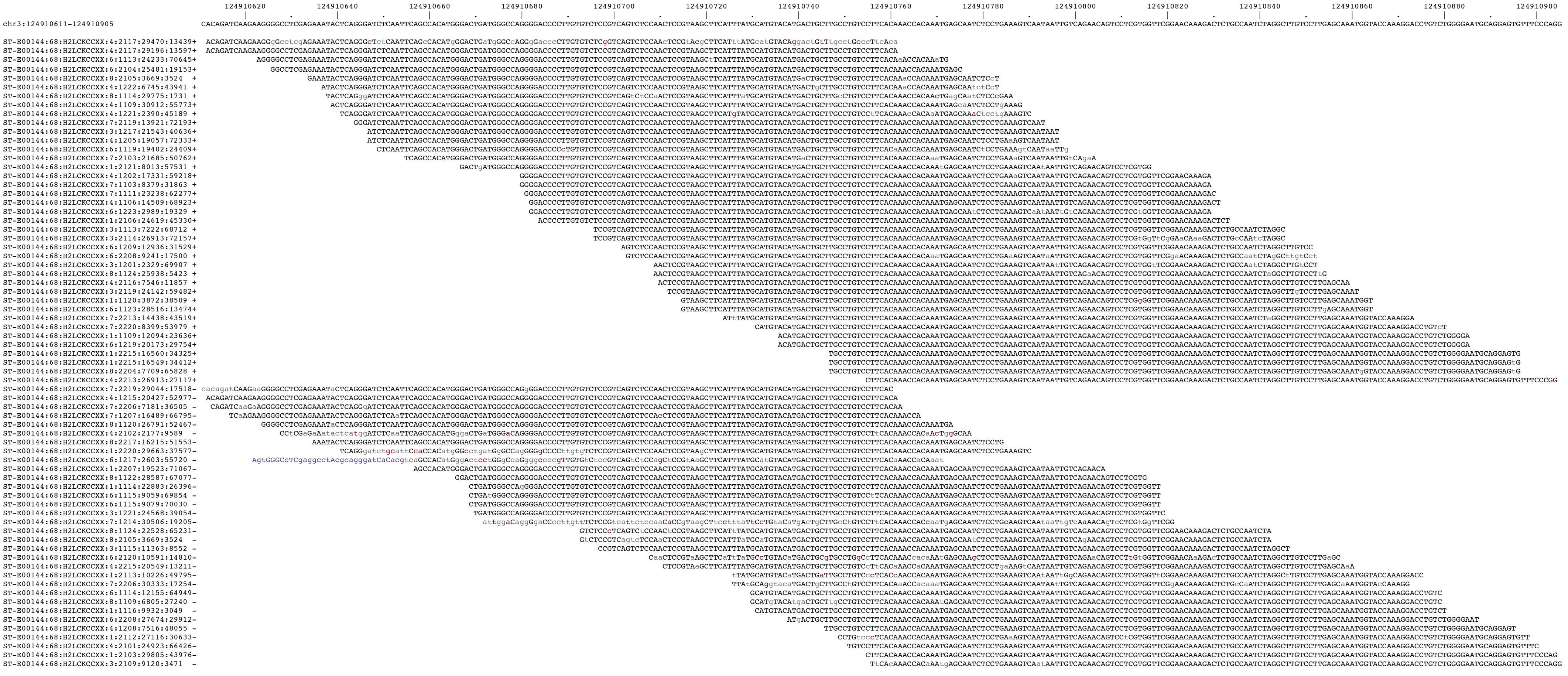

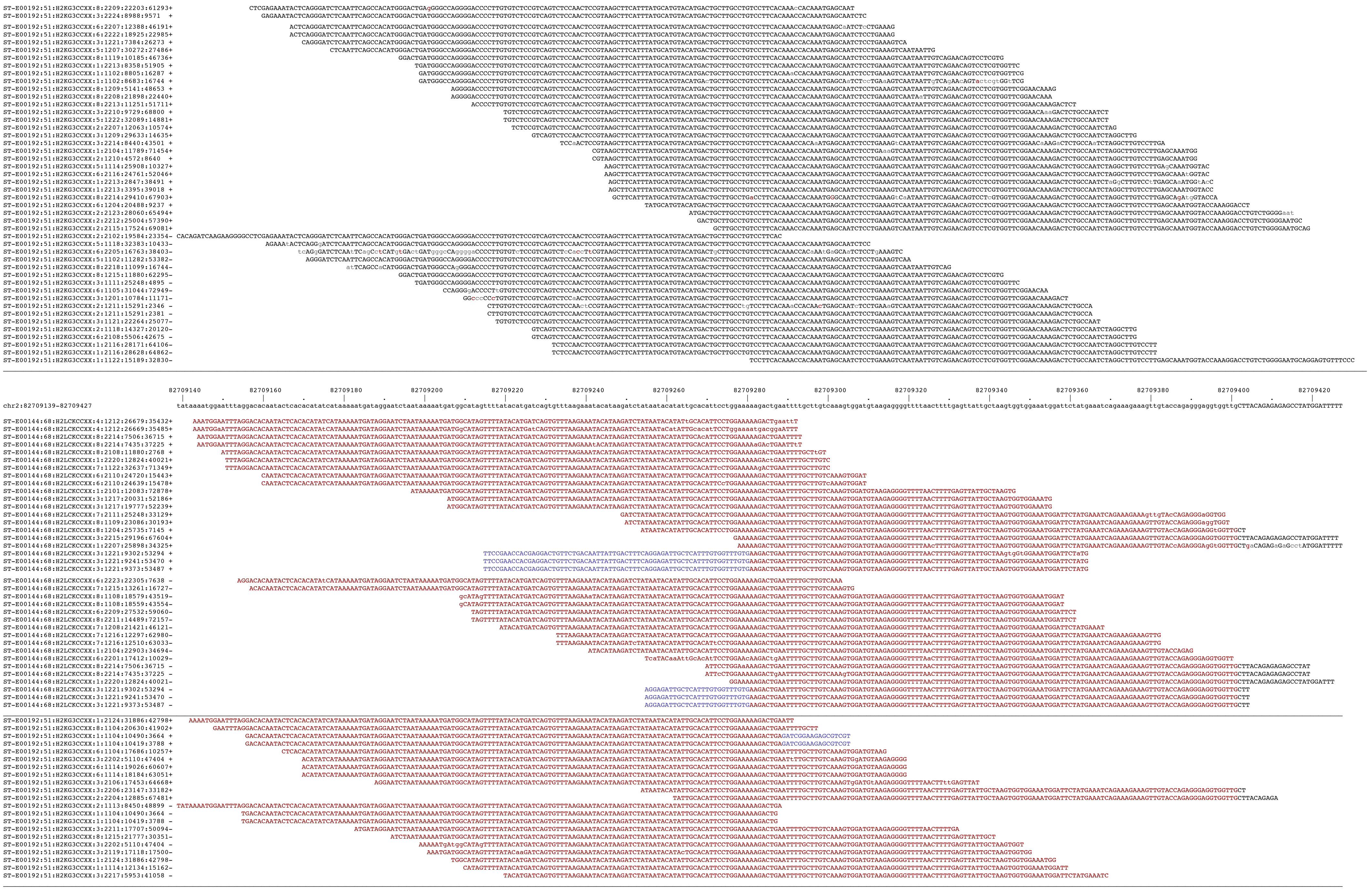

chr2 124853632 + 5 chr1 32476210 + 0 CTX 35 49 59 0 0.868888888888889 0 1 0 1 chr2 124853542 150 chr1 32476268

GCATAGTGAGCAGATTGCAGAGGAGAGATTCAAAACTAAGTCCCCCTGTCTTCAAACTGCTCTTAAAGATTTTTCTCAGTGGGTCCCTGAATAGGGTTCTCGCCTGGAAGAAGAGCACATCTCTCCCGCTCAGCCTACTCTCAGGGAAAG

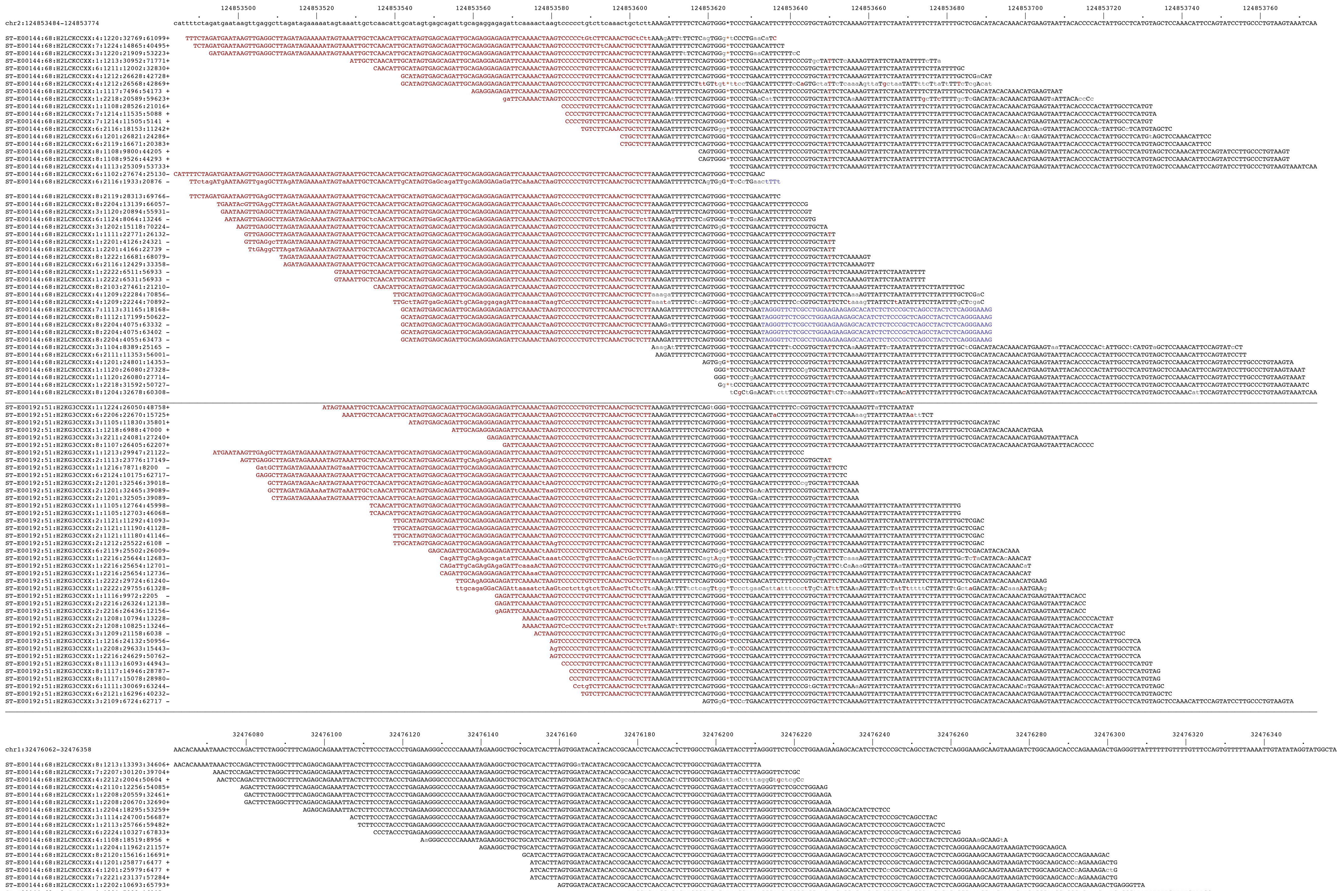
chr2:124853484–124853774
chr1:32476062–32476358

```
ST-E00144:68:H2LCKCCXX:1:1221:7922:16305 + AACCTCAACCACTCTTGGCCTGAGATTACCTTTAGGGTTCTCGCCTGGAAGAAGAGCACATCTCTCCCGCTCAGCCTACTCTCAGGGAAAGCAAGTAAAGATCTGGCAAGCACCCAGAAAGAcTGaGGGtTATTTTTTTGTTTTGTtTCCa
ST-E00144:68:H2LCKCCXX:6:1112:21939:23390+ CTCTTGGCCTGAGATTACCTTTAGGGTTCTCGCCTGGaAgAAGAGCACATcTCTCCCGCtCAGCCTACTCTCAGGGAAAGCAAGTAAAGATCTGGCAAGCACCCAGAAAGACTGaGGGTTATtTTTtGTTTTGTTTtCAGTGTTTTTAAc
ST-E00144:68:H2LCKCCXX:3:2211:6339:30650 + ACCTTTAGGGTTCTCGCCTGGAAGAAGAGCACATCTCTCCCGCTCAGCCTACTCTCAGGGAAAGCAAGTAAAGATCTGGCAAGCACCCAGAAAGACTGAGGGTTATTTTTTGTTTTGTTTCCAGTGTTTTTAAAATTGTATATAGTTATG
ST-E00144:68:H2LCKCCXX:8:2210:25004:26167- AAAATAaactCcagTCTTcTAGGCTTTCAGAGCAGAAATTaCtccTCctTaCCCTGAGAAGGGCCCCCAGAATAGAAGGCTGCTGCATCACTTAGTGGATACATACACCGCAACCTCAACCACTCTTGGCCTGAGATTACCTTTAGGGTT
ST-E00144:68:H2LCKCCXX:6:1118:7841:57232 - AATAAACTCCAGACTTCTAGGCTTTCAGAGCAGAAATTACTCTTCCCTACCCTGAGAAGGGCCCCCAAAATAGAAGGCTGCTGCATCACTTAGTGGATACATACACCGCAACCTCAACCACTCTTGGCCTGAGATTACCTTTAGGGTTCT
ST-E00144:68:H2LCKCCXX:1:1103:5699:56054 - CAGACTTCTAGGCTTTCAGAGCAGAAATTACTCTTCCCTACCCTGAGAAGGGCCCCCAAAATAGAAGGCTGCTGCATCACTTAGTGGATACATACACCGCAACCTCAACCACTCTTGGCCTGAGATTACCTTTAGGGTTCTCGCCTGGAA
ST-E00144:68:H2LCKCCXX:6:1120:3903:49848 - AGACTTCTAGGCtTtCAGAGCAGAAATTACTCTTCCCTACCCTGAGAAGGGCCCCCAAAATAGAAGGCTGCTGCATCACTTAGTGGATACATAcACCGCAACCTCAACCACTCTTGGCCTGAGATTACCTTTAGGGTTCTCGCCTGGAAG
ST-E00144:68:H2LCKCCXX:6:2208:22447:63051- GCTTTCAGAGCAGAAATTACTCTTCCCTACCCTGAGAAGGGCCCCCAAAATAGAAGGCTGCTGCATCACTTAGTGGATACATACACCGCAACCTCAACCACTCTTGGCCTGAGATTACCTTTAGGGTTCTCGCCTGGAAGAAGAGCACAT
ST-E00144:68:H2LCKCCXX:8:1123:21645:53188- TTCAGAGCAGAAATTACTCTTCCCTACCCTGAGAAGGGCCCCCAAAATAGAAGGCTGCTGCATCACTTAGTGGATACATACACCGCAACCTCAACCACTCTTGGCCTGAGATTACCTTTAGGGTTCTCGCCTGGAAGAAGAGCACATCTC
ST-E00144:68:H2LCKCCXX:1:1211:22091:40812- CAGAAATTaCTCTTCCCTACCCTGAGAAGGGCCCCCAAAATAGAAGGCTGCTGCATCACTTAGTGGATACATACACCGCAACCTCAACCACTCTTGGCCTGAGATTACCTTTAGGGTTCTCGCCTGGAAGAAGAGCACATCTCTCCCGCT
ST-E00144:68:H2LCKCCXX:1:1121:17301:30228- GAaaTTACTCTTCCCTaCCCTGAGAaGGGCCCCCAAAATAGAAgGCTGCTGCATCACtTAGTGGATACATACACCGCAACcTCAACCACTCTTGGCCTGAGATTACCTTTAGGGTTCTCGCCTGGAAGAAGAGCACATCTCTCCCGCTCA
ST-E00144:68:H2LCKCCXX:6:1105:23086:12754- CTCTTCCCTAcCCTGAGAAGGGCCCCaaAAATAgAAgGCTGCTGCATCACTTAGTGGATACATACACCGCAACCTCAACCaCTCTTGGCCTGAGATTACCTTTAGGGTTCTCGCCTGGAAGAAGAGCACATCTCTCCCGCTCAGCCTACT
ST-E00144:68:H2LCKCCXX:3:1120:32830:59025- ttCcCtacCCtGAGAaGggCCcCCAaaATaGAAGGCtGCTGCATCACTTAGTGGaTaCATACaCCGCAACCTCAACCACTCTTGGCCTGAGATTACCTTTAGGGTTCTCGCCTGGAAGAAGAGCACATCtCTCCCGCTCAGCCTACTCTC
ST-E00144:68:H2LCKCCXX:1:1207:29917:72404- CTACCCTGAGAAGGGCCCCCAaaataGAAGGCTGCTGCATCACTTAGTGGATACATACACCGCAACCTCAACCaCTCTTGGCCTGAGATTACCTTTAGGGTTCTCGCCTGGAAGAAGAGCACATCTCTCCCGCTCAGCCTACTCTCAGGG
ST-E00144:68:H2LCKCCXX:1:2223:21645:57232- GAAGGGCCCCCAAAATAGAAGGCTGCTGCATCACTTAGTGGATACATACACCGCAACCTCAACCACTCTTGGCCTGAGATTACCTTTAGGGTTCTCGCCTGGAAGAAGAGCACATCTCTCCCGCTCAGCCTACTCTCAGGGAAAGCAAGT
ST-E00144:68:H2LCKCCXX:8:2215:27725:5880 - GAAGGGCCCCCAAAATAGAAGGCTGCTGCATCACTTAGTGGATACATACACCGCAACCTCAACCaCTCTTGGCCTGAGATTACCTTTAGGGTTCTCGCCTGGAAGAAGAGCACATCTCTCCCGCTCAGCCTACTCTCAGGGAAAGCAAGT
ST-E00144:68:H2LCKCCXX:4:2110:6166:27714 - CcaAAAtagaaGGCTGcTGCATCACTTAGTGGATACATACaCCGCAACCTCAaCCaCTCTTgGCCTGAGATTACCTTTaGGGTTCTCGCCTGGAAGAAGAGCACATCtCTCCCGCTCAGCCtACTCTCAGGGAAAGCAAGTAAAGATCtG
ST-E00144:68:H2LCKCCXX:3:1119:2908:68132 - aaatAgAAGGCtgCtgcATCACTTAGTgGATAcAtacAcCgCAAcCTCaaCCcCTCttgGCCTGAGATTACCTTtagGGTTcTCGCCTGGAAGAAGAGCACATCTCTCCCGCTcAGCCTACTCTCAGGGAAAGCAAGTAAAGATCTGGCA
ST-E00144:68:H2LCKCCXX:3:2214:24659:21632- GCTGCTGCATCACTtAGTGGATACaTACaCCGCAACCTCAACCACTCTTGGCCTGaGATTACCTTTAGGGTTCTCGCCTGGAAGAAGAGCACATCTCTCCCGCTCAGCCTACTCTCAGGGAAAGCAAGTAAAGATCTGGCAAGCACCCAG
ST-E00144:68:H2LCKCCXX:6:1208:6724:49145 - GCTGcaccACTTAGTGgATACATACACCGCAACCTCAaCccCTCTtGGCCTGAGATTACCTTTAGGGTTCTCGCCTGGAAGAAGAGCACATCTCTCCCGCTCAGCCTACtCTCAGGGAAAGCAAGTAAAGATCTGGCAAGCACCCAGAAA
ST-E00144:68:H2LCKCCXX:8:1207:29887:61838- CATCACTTAGTGGATACATACACCGCAACCTCAACCACTCTTGGCCTGAGATTACCTTTAGGGTTCTCGCCTGGAAGAAGAGCACATCTCTCCCGCTCAGCCTACTCTCAGGGAAAGCAAGTAAAGATCTGGCAAGCACCCAGAAAGACT
ST-E00144:68:H2LCKCCXX:1:1121:10713:19979- CACTTAGTGGATACATACACCGCAACCTCAACCACTCTTGGCCTGAGATTACCTTTAGGGTTCTCGCCTGGAAGAAGAGCACATCTCTCCCGCTCAGCCTACTCTCAGGGAAAGCAAGTAAAGATCTGGCAAGCACCCAGAAAGACTGAG
ST-E00144:68:H2LCKCCXX:4:1205:29146:15443- TAGtggagACaTACACCGCaACCtCAaccACTCTTGGCCtgaGATTACCTTTAGGGTTCgCGCCTGGAAGAAGAGCACatCTCTCCCGCTCAGCCTACTCTCAgGGAAAGCAAGTAAAGATCTGGCAAGCACCCAGAaAGACTGAGGGTT
ST-E00144:68:H2LCKCCXX:6:2107:22030:4420 - GTGGATACataCaCCGCAACCTCAACCACTCTtgGCCTGAGATTaCCTTTAGGGTTCTCGCCTGGAAGAAGAGCACATCTCTCCCGCTCAGCCTACTCTCAGGGAAAGCAAGTAAAGATCTGGCAAGCACCCAGAAAGACTGAGGGTTAT
ST-E00144:68:H2LCKCCXX:7:1118:23238:62383- TACATACACCGCAACCTCAACCACTCTTGGCCTGAGATTACCTTTAGGGTTCTCGCCTGGAAGAAGAGCACATCTCTCCCGCTCAGCCTACTCTCAGGGAAAGCAAGTAAAGATCTGGCAAGCACCCAGAAAGACTGAGGGTTATTTTTT
ST-E00144:68:H2LCKCCXX:8:1221:29003:41497- ataCACCgcaACCgCAACCACtCTTGGCctgaGATTACCGTTAGGGTTctCGCCTGGAAGAAGAGCACATCTCTCCCGCTCAGCCTACTCTCAGGGAAAGCAAGTAAAGATCTGGCAAGCACCCAGAAAGACTGAGGGTTATTTTTTTGTT
ST-E00144:68:H2LCKCCXX:6:1223:9820:41497 - CGCAaCCTCAACCACTCTTGGCCTGAGATTACCTTTAGGGTTCTCGCCTGGAAGAAGAGCACATCtCTCCCGCTCAGCCTACTCTCAGGGAAAGCAAGTAAAGATCTGGCAAGCACCCAGAAAGACTGAGGGTTATTTTTTTGTTTTGTTT
ST-E00144:68:H2LCKCCXX:3:1120:9353:55878 - ACCaCTCTTGGCCTGAGATTACCTTTAGGGTTCTCGCCTGGAAGAAGAGCACATCTCTCCCGCTCAGCCTACTCTCAGGGAAAGCAAGTAAAGATCTGGCAAGCACCCAGAAAGACTGAGGGTTATTTTTTTGTTTTGTTTCCAGTGTTTT
ST-E00144:68:H2LCKCCXX:6:1211:19046:42218- ACCACTCTTGGCCTGAGATTACCTTTAGGGTTCTCGCCTGGAAGAAGAGCACATCTCTCCCGCTCAGCCTACTCTCAGGGAAAGCAAGTAAAGATCTGGCAAGCACCCAGAAAGACTGAGGGTTATTTTTTTGTTTTGTTTCCAGTGTTTT
ST-E00144:68:H2LCKCCXX:1:1205:30607:44521- CCcCTcttgGCCTGAGATTACCTTTAGGGTTCTCGCCTGGAAGAAGAGCACATCTCTCCCGCTCAGCCTACTCTCAGGGAAAGCAAGTAAAGATCTGGCAAGCACCCAGAAAGACTGAGGGTTATTTTTTTGTTTTGTTTCCAGTGTTTT
ST-E00144:68:H2LCKCCXX:1:2205:29947:32936- CCCACCtcaaaCcctcTTGGCCtgaggTTgcCtTtaggGTTCTCGCCtgGAAGAAGAGCACAtCaCTCCCGCTCAGCCTaCTCTCAGGGAAAGCAAGTAAAGATCTGGCAAGCACCCAGAAaGACTGAGGGTTATTTTTTTGTTTTGTTTC
ST-E00144:68:H2LCKCCXX:8:2220:12753:38280- TTGGCCTGAGATTACCTTTAGGGTTCTCGCCTGGAAGAAGAGCACATCTCTCCCGCTCAGCCTACTCTCAGGGAAAGCAAGTAAAGATCTGGCAAGCACCCAGAAAGACTGAGGGTTATTTTTTTGTTTTGTTTCCAGTGTTTTTAAAATT
ST-E00144:68:H2LCKCCXX:6:2118:32617:41251- CTGAGATTACCTTTAGGGTTCTCGCCTGGAAGAAGAGCACAtCTCTCCCGCTCAGCCTACTCTCAGGGAAAGCAAGTAAAGATCTGGCAAGCACCCAGAAAGACTGAGGGTTATTTTTTTGTTTTGTTTCCAGTGTTTTTAAAATTGTATA
ST-E00144:68:H2LCKCCXX:7:2207:30120:39704- CTTTAGGGTTCTCGCCTGGAAGAAGAGCACATCTCTCCCGCTCAGCCTACTCTCAGGGAAAGCAAGTAAAGATCTGGCAAGCACCCAGAAAGACTGAGGGTTATTTTTTTGTTTTGTTTCCAGTGTTTTTAAAATTGTATATAGTTATGGC
```

```
ST-E00192:51:H2KG3CCXX:2:2219:3913:9150  + AATAAACTCCAGACTTCTAGGCTTTCAGAGCAGAAATTACTCTTcCCTAcCCTGAGAaGGGCCCCCAAAATAGAAGGCTGCTGCATCACTTAGTGGaTACATACACCGCAACaTCAACCACTCTTGGCCTGaGATTaCcTTTaGGGTTcT
ST-E00192:51:H2KG3CCXX:2:2117:20609:11136+ TCTAGGCTTTCAGAGCAGAAATTACTCTTCCCTACCCTGAGAAGGGCCCCCAAAATAGAAGGCTGCTGCATCACTTAGTGGATACATACACCGCAACCTCAACCACTCTTGGCCTGAGATTACCTTTAGGGTTCTCGCCTGGAAGAAGAG
ST-E00192:51:H2KG3CCXX:2:2117:20599:11154+ tCTAGGCTTTCAGAGCAGAAATTACTCTTCCCTACCCTGAGAAGGGCCCCCAAAATAGAAGGCTGCTGCATCACTTAGTGGATACATACACCGCAACCTCAACCACTCTTGGCCTGAGATTACCTTTAGGGTTCTCGCCTGGAAGAAGAG
ST-E00192:51:H2KG3CCXX:3:1121:9810:19961 + GAAATTACTCTTCCCTACCCTGAGAAGGGCCCCCAAAATAGAAGGCTGCTGCATCACTTAGTGGATACATACACCGCAACCTCAACCACTCTTGGCCTGAGATTACCTTTAGGGTTCTCGCCTGGAAGAAGAGCACATCTCTCCCGCTCA
ST-E00192:51:H2KG3CCXX:3:1121:9820:19979 + GAAATTACTCTTCCCTACCCTGAGAAGGGCCCCCAAAATAGAAGGCTGCTGCATCACTTAGTGGATACATACACCGCAACCTCAACCACTCTTGGCCTGAGATTACCTTTAGGGTTCTCGCCTGGAAGAAGAGCACATCTCTCCCGCTCA
ST-E00192:51:H2KG3CCXX:8:2113:30425:39247+ TtCCCTACCCTGAGAAGGGCcCCcAAAATAGAAGGcTGcTGCATCACTTAGTGGaTAcaTAcatCGCAACCTCAACCACTCTTGGcCTGAGAttAcctTTAGGGTtCgtGCCTGGAAGAAGAGcactgctctcacgctgaGcctacgctC
ST-E00192:51:H2KG3CCXX:1:2107:31805:17026+ AAGGGCCCCCAAAATAGAAGGCTGCTGCATCACTTAGTGGATACATACACCGCAACCTCAACCACTCTTGGCCTGAGATTACCTTTAGGGTTCTCGCCTGGAAGAAGAGcAcATCTaTCCCgCTCAGCCTACTctCAGGGAAAGcAAGtA
ST-E00192:51:H2KG3CCXX:8:2117:21635:18326+ GAAGGCTGCTGCATCACTTAGTGGATACATACACCGCAACCTCAACCACTCTTGGCCTGAGATTACCTTTAGGGTTCTCGCCTGGAAGAAGAGCACATCTCTCCCGCTCAGCCTACTCTCAGGGAAAGCAAGTAAAGATCTGGCAAGCAC
ST-E00192:51:H2KG3CCXX:6:2221:11617:16270+ CTGCATCACTTAGTGGATACATACACCGCAACCTCAACCACTCTTGGtCTGAGATTACCTTTAGGGTTCTCGCCTGGAAGAAGAGCACATCTCTCCCGCTCAGCCTACTCTCAGGGAAAGCAAGTAAAGATCTGGCAAGCACCCAGAAAG
ST-E00192:51:H2KG3CCXX:1:2223:11677:47773+ CACTTAGTGGATACATACACCGCAACCTCAACCACTCTTGGCCTGAGATTACCTTTAGGGTTCTCGCCTGGAAGAAGAGCACATCTCTCCCGCTCAGCCTACTCTCAGGGAAAGCAAGTAAAGATCTGGCAAGCACCCAGAAAGACTGAG
ST-E00192:51:H2KG3CCXX:5:2122:21980:14529+ CTTAGTGGATACATACACCGCAACCTCAACCACTCTTGGCCTGAGATTACCTTTAGGGTTCTCGCCTGGAAGAAGAGCACATCTCTCCCGCTCAGCCTACTCTCAGGGAAAGCAAGTAAAGATCTGGCAAGCACCCAGAAAGACTGAGGG
ST-E00192:51:H2KG3CCXX:5:2122:22020:15057+ CTTAGTGGATACATACACCGCAACCTCAACCACTCTTGGCCTGAGATTACCTTTAGGGTTCTCGCCTGGAAGAAGAGCACATCTCTCCCGCTCAGCCTACTCTCAGGGAAAGCAAGTAAAGATCTGGCAAGCACCCAGAAAGACTGAGGG
ST-E00192:51:H2KG3CCXX:8:1211:5861:16182 + GCAACCTCAACCACTCTTGGCCTGAGATTACCTTTAGGGTTCTCGCCTGGAAGAAGAGCACATCTCTCCCGCTCAGCCTACTCTCAGGGAAAGCAAGTAAAGATCTGGCAAGCACCCAGAAAGACTGAGGGTTATTTTTTTGTTTTGTTTC
ST-E00192:51:H2KG3CCXX:5:1210:32262:49321+ ACCTCAACCACTCTTGGCCTGAGATTACCTTTAGGGTTCTCGCCTGGAAGAAGAGCACATCTCTCCCGCTCAGCCTACTCTCAGGGAAAGCAAGTAAAGATCTGGCAAGCACCCAGAAAGACTGAGGGTTATTTTTTTGTTTTGTTTCCAG
ST-E00192:51:H2KG3CCXX:5:1210:32241:49356+ ACCTCAACCACTCTTGGCCTGAGATTACCTTTAGGGTTCTCGCCTGGAAGAAGAGCACATCTCTCCCGCTCAGCCTACTCTCAGGGAAAGCAAGTAAAGATCTGGCAAGCACCCAGAAAGAcTGAGGGTTATTTTTTTGTTTTGTTTCCAG

ST-E00192:51:H2KG3CCXX:3:2102:29816:28031+ TCTTGGCCTGAGATTACCTTTAGGGTTCTCGCCTGGAAGAAGAGCACATCTCTCCCGCTCAGCCTACTCTCAGGGAAAGCAAGTAAAGATCTGGCAAGCACCCAGAAAGACTGAGGGTTATTTTTTTGTTTTGTTTCCAGTGTTTTTAAAA
ST-E00192:51:H2KG3CCXX:6:2109:3781:48688 + CTTGGCCTGAGATTACCTTTAGGGTTCTCGCCTGGAAGAAGAGCACATCTCTCCCGCTCAGCCTACTCTCAGGGAAAGCAAGTAAAGATCTGGCAAGCACCCAGAAAGACTGAGGGTTATTTTTTTGTTTTGTTTCCAGTGTTTTTAAAAT
ST-E00192:51:H2KG3CCXX:2:1110:21858:56300- AAATAaaCTCCAgACTTCTAGGCTTTCAGAGCAGAAATTACTCTTCCCTACCCTGAGAAGGGCCCCCAAAATAGAAGGCTGCTGCATCACTTAGTGGATACATACACCGCAACCTCAACCACTCTTGGCCTGAGATTACCTTTAGGGTTC
ST-E00192:51:H2KG3CCXX:1:2122:16793:35960- AATAAACTCCAGACTTCTAGGCTTTCAGAGCAGAAATTACTCTTCCCTACCCTGAGAAGGGCCCCCAAAATAGAAgGCTGCTGCATCACTTAGTGGATACATACACCGCAACCTCAACCACTCTTGGCCTGAGATTACCTTTAGGGTTCT
ST-E00192:51:H2KG3CCXX:1:2122:16570:36030- AATAAACTCCAGACTTCtAGgCTTTCAGAGCAGAAATTACTCTTCCCTACCCTGAGAAGGGCCCCCAAAATAGAAGGCTGCTGCATCACTTAGTGGATACATACACCGCAACCTCAACCACTCTTGGCCTGAGATTACCTTTAGGGTTCT
ST-E00192:51:H2KG3CCXX:1:2122:16631:36311- aAtaAaCTCCAGACTTCTAGGCTTTCAGAGCAGAAATTACTCTTCCCTACCCTGAGAAGGGCCCCCAAAATAGAAgGCTGCTGCATCACTTAGTGGATACATACACCGCAACCTCAACCACTCTTGGCCTGAGATTACCTTTAGGGTTCT
ST-E00192:51:H2KG3CCXX:2:1219:3933:68676 - ATacaCtCCaGACtTcTagGCtttcagAGcAgaAAtTACTCTTCCCTaCCCtGAGAagggCCCCCaAaaTAGAAGGCTGCTGCATCACTTAgTGGATACATACACCGCAACCTCAACCACTCTTGGCCTGAGATTACCTTTAGGGTTCTC
ST-E00192:51:H2KG3CCXX:1:1207:21015:30580- ACTCCAGACTTCTAGGCTTTCAGAGCAGAAATTACTCTTCCCTACCCTGAGAAGGGCCCCCAAAATAGAAGGCTGCTGCATCACTTAGTGGATACATACACCGCAACCTCAaCCAcTCttGGCCTGAgAtTACcTTTAGGGTTCTCGCct
ST-E00192:51:H2KG3CCXX:1:2208:27633:64897- TTCAGAGCAGAAATTACTCTTCCCTACCCTGAGAAGGGCCCCCAAAATAGAAgGCTGCTGCATCACTTAGTGGATACATACACCGCAACCTCAACCACTCTTGGCCTGAGATTACCTTTAGGGTTCTCGCCTGGAAGAAGAGCACATCTC
ST-E00192:51:H2KG3CCXX:5:2120:15392:7022 - cagAgcaGAAATTACTCTtCCCtACCCTGAGAaGgGCCCCCAAAATAGAaGGCTGCTGCATCACTTAGTGGATACATACACCGCAACCTCAACCaCTCTTGGCCTGAGATTACCTTTAGGGTTCTCGCCTGGAAGAAGAGCACATCTCTC
ST-E00192:51:H2KG3CCXX:2:2218:5060:62682 - GAAATtaCTCtTCCCTACCCTGAGAAGGGCCCCCAAAATAGAAGGCTGCTGCATCACTTAGTGGATACATACACCGCAACCTCAACCACTCTTGGCCTGAGATTACCTTTAGGGTTCTCGCCTGGAAGAAGAGCACATCTCTCCCGCTCA
ST-E00192:51:H2KG3CCXX:3:1209:7039:24726 - GAAATTACTCTTCCCTACCCTGAGAAGGGCCCCCAAAATAGAAGGCTGCTGCATCACTTAGTGGATACATACACCGCAACCTCAACCACTCTTGGCCTGAGATTACCTTTAGGGTTCTCGCCTGGAAGAAGAGCACATCTCTCCCGCTCA
ST-E00192:51:H2KG3CCXX:5:1102:8399:26660 - ATTACTCTTCCCtACCCTGAGAAGGGCCCCCAAAATAGAAGGCTGCTGCATCACTTAGTGGATACATACACCGCAACCTCAACCACTCTTGGCCTGAGATTACCTTTAGGGTTCTCGCCTGGAAGAAGAGCACATCTCTCCCGCTCAGCC
ST-E00192:51:H2KG3CCXX:6:1206:22538:37436- ATTaCTCTTCCCTACCCTGAGAAGGGCCCCCAAAATAGAAGGCTGCTGCATCACTTAGTGGATACATACACCGCAACCTCAACCACTCTTGGCCTGAGATTACCTTTAGGGTTCTCGCCTGGAAGAAGAGCACATCTCTCCCGCTCAGCC
ST-E00192:51:H2KG3CCXX:5:1123:23868:21421- ACCCTGAGAAgGGCCCCCAAaATAGAaGGCTGCTGCATCACtgAGTGGATACATACACCGCAACCTCAACCACTCTTGGCCTGAGATTACCTTTAGgGTTCTCGCCTGGAAGAAGAGCACATCTCTCCCGCTCAGCCTACTCTCAGGGAA
ST-E00192:51:H2KG3CCXX:8:1114:26811:18537- ACcCTGAGAAgGGCCCcCAAAAtaGaaGgCTGcTGcAtCaCtTAGTGGATACAtACACCGCAACCTCAaCCACTCTtGGCCTgAGaTTACCTTtAgtGTTCTCGCCTGgAAtAAGaGCACATCTcTCCCGCTCaGCCTaCTCTCAgggAA
ST-E00192:51:H2KG3CCXX:2:1218:30455:11910- CCCTGAGAAGGGCCCCCAAAATaGAagGCTGCTGCATCACTTaGTGGATACATACACCGCAcCCTCAACCaCTCTTGGCCTGAGATTACCTTTAGGGTTCTCGCCTGGAAGAAGAGCACATCTCTCCCGCTCAGCCTACTCTCAGGGAA
ST-E00192:51:H2KG3CCXX:6:2218:32424:71753- CTGAgAAGGGCCCCCAAAATAGAAGGCTGCTGCATCACTTAGTGGATACATACACCGCAACCTCAACCACTCTTGGCCTGAGATTACCTTTAGGGTTCTCGCCTGGAAGAAGAGCACATCTCTCCCGCTCAGCCTACTCTCAGGGAAAGC
ST-E00192:51:H2KG3CCXX:8:2101:8876:10995 - CtGagaAgggCCCCCaaacTAGAaGGCTGCtcCATCACTTAGTGGATACATaCaCCGCAACCTCAACCaCTCTTGGCCTGAGATTACCTtTAGGGTTCTCGCCTGgAAGAAGAGCACATCTCTCCCGCTCAGCCTACTCTCAGGGAAAGC
ST-E00192:51:H2KG3CCXX:6:2114:19077:21280- GAGAaGGGCCCCCAAAATAGAAGGCTGCTGCATCACTTAGTGGATACATACACCGCAACCTCAACCACTCTTGGCCTGAGATTACCTTTAGGGTTCTCGCCTGGAAGAAGAGCACATCTCTCCCGCTCAGCCTACTCTCAGGGAAAGCAA
ST-E00192:51:H2KG3CCXX:6:2114:18914:21350- GAGAAgGGCCCCCAAAATAGAAGGCTGCTGCATCACTTAGTGGATACATACACCGCAACCTCAACCACTCTTGGCCTGAGATTACCTTTAGGGTTCTCGCCTGGAAGAAGAGCACATCTCTCCCGCTCAGCCTACTCTCAGGGAAAGCAA
ST-E00192:51:H2KG3CCXX:2:2221:25542:13527- AGAAGGGCCCCCAAAATAGAAGGCTGCTGCATCACTTAGTGGATACATACACCGCAACCTCAACCACTCTTGGCCTGAGATTACCTTTAGGGTTCTCGCCTGGAAGAAGAGCACATCTCTCCCGCTCAGCCTACTCTCAGGGAAAGCAAG
ST-E00192:51:H2KG3CCXX:1:2110:30820:44046- AGGGCCCCCAAAATAGAAGGCTGCTGCATCACTTAGTGGATACATACACCGCAACCTCAACCACTCTTGGCCTGAGATTACCTTTAGGGTTCTCGCCTGGAAGAAGAGCACATCTCTCCCGCTCAGCCTACTCTCAGGGAAAGCAAGTAA
ST-E00192:51:H2KG3CCXX:5:2123:17219:26431- aaattgaAggCTGCtGCATCACTTAGTgGaTACATaCaCCGCAACCTCAACCActCTTGGCCTGAGATTACCTTTAGGGTTCTCGCCTGGAAGAAGAGCaCaTCTCTCCCGCTCAGCCTACTCTCAGGGAAAGCAAGTAAAGATCTGGCA
ST-E00192:51:H2KG3CCXX:5:2123:17148:28418- AAATAGAaGgCTGCTGCAtCACTTAGTGgATACATACcCCGCAACCTCAACCACTCTTGGCCTGAGATTACCTTTAGGGTTCTCGCCTGGAAGAAGAGCACATCTCTCCCGCTCAGCCTACTCTCAGGGAAAGCAAGTAAAGATCTGGCA
ST-E00192:51:H2KG3CCXX:5:1103:19696:71577- AAGGCTGCTGCATCACTTAGTGGATACATACACCGCAACCTCAACCACTCTTGGCCTGAGATTACCTTTAGGGTTCTCGCCTGGAAGAAGAGCACATCTCTCCCGCTCAGCCTACTCTCAGGGAAAGCAAGTAAAGATCTGGCAAGCACC
ST-E00192:51:H2KG3CCXX:5:1103:19838:71612- AAGGCTGCTGCATCACTTAGTGGATACATACACCGCAACCTCAACCACTCTTGGCCTGAGATTACCTTTAGGGTTCTCGCCTGGAAGAAGAGCACATCTCTCCCGCTCAGCCTACTCTCAGGGAAAGCAAGTAAAGATCTGGCAAGCACC
ST-E00192:51:H2KG3CCXX:2:1207:7445:53382 - GCTGCTGCATCaCgTgGTGGAtACAtACACCGCAaCCTCaACCaCTCTTGGCCTGgGATTACCTTTAGGGTTCTCGCCTGGAAGAAGAGCACATCTCTCCCGCTCAGCCTACTCTCAGGGAAAGCAAGTAAAGATCTGGCAAGCACCCAG
ST-E00192:51:H2KG3CCXX:1:1118:30364:60941- TTAGTGGATACATACACCGCAACCTCAACCACTCTTGGCCTGAGATTACCTTTAGGGTTCTCGCCTGGAAGAAGAGCACATCTCTCCCGCTCAGCCTACTCTCAGGGAAAGCAAGTAAAGATCTGGCAAGCACCCAGAAAGACTGAGGGT
ST-E00192:51:H2KG3CCXX:8:2102:4765:64422 - aatccatACAtACaCCcCAACCTCaaCCaCtctTagcCtgagaTTACCTTTAttGTTCTcgCCTGGAAGAAGAGCACATCTCTCCCGCTCAGCCTACTCTCAGGGAAAGCAAGTAAAGATCTGGCAAGCACCCAGAAAgACTGAGGGTTA
ST-E00192:51:H2KG3CCXX:5:2106:22913:57847- TACATACACCGCAACCTCAACCACTCTTGGCCTGAGATTACCTTTAGGGTTCTCGCCTGGAAGAAGAGCACATCTCTCCCGCTCAGCCTACTCTCAGGGAAAGCAAGTAAAGATCTGGCAAGCACCCAGAAAGACTGAGGGTTATTTTTT
ST-E00192:51:H2KG3CCXX:5:2106:23604:58656- TACATACACCGCAaCCTCAACCaCTcTTGGCCTGAGATTACCTTTAGGGTTCTCGCCTGGAAGAAGAGCACATCtCtCCCGCTCAGCCTACTCTCAGGGAAAGCAAGTAAAGATCTGGCAAGCACCCAGAAAGACTGAGGGTTATTTTTT
ST-E00192:51:H2KG3CCXX:8:2113:16722:44099- TACATACACCGCAACCTCAACCACTCTTGGCCTGAGATTACCTTTAGGGTTCTCGCCTGGAAGAAGAGCACATCTCTCCCGCTCAGCCTACTCTCAGGGAAAGCAAGTAAAGATCTGGCAAGCACCCAGAAAGACTGAGGGTTATTTTTT
ST-E00192:51:H2KG3CCXX:5:1213:11880:12613- ACCGCAACCTCAACCACTCTTGGCCTGAGATTACCTTTAGGGTTCTCGCCTGGAAGAAGAGCACATCTCTCCCGCTCAGCCTACTCTCAGGGAAAGCAAGTAAAGATCTGGCAAGCACCCAGAAAGACTGAGGGTTATTTTTTTGTTTTGT
ST-E00192:51:H2KG3CCXX:3:1102:7283:27503 - TTAGGGTTCtCGCCTGGAAGAAGAGCACATCTCTCCCGCTCAGCCTACTCTCAGGGAAAGCAAGTAAAGATCTGGCAAGCACCCAGAAAGACTGAGGGTTATTTTTTTGTTTTGTTTCCAGTGTTTTTAAAATTGTATATAGTTATGGCTA
```

---

chr2 216657668 + 8 chr9 79474177 - 0 CTX 9 24 45 0 0.671240256256803 0 1 0 1 chr2 216657552 150 chr9 79474145

CTCACTGATGACAAGGTTCGTGGCTGACACCCCATAACAAAAGACAGATTAACAAGAGAAAATCATAACAAAATTGTTTAACTAGAGTTTTACATGACATGGGAGCCTTCAGAAATGGATTACACTTTGAAAGCATATTAAAGTGATTTG

```
216657540 216657560 216657580 216657600 216657620 216657640 216657660 216657680 216657700 216657720 216657740 216657760 216657780
chr2:216657527-216657792 aggggagaaaaggtattatctttttctcactgatgacaaggttcgtggctgacaccccataacaaaagacagattaacaagagaaaatcataacaaaattgtttaactagagttttacatgacatgggagccttcagaaatgaataccctcaaaccaggggaaaattgtgatttttttttttcttttttcttgtcttttttttttttttttttttttggcttaggttctatgaagaacgggcagtcatgtagaactacaactggaca

ST-E00144:68:H2LCKCCXX:3:1210:31693:49953+ CTCACTGATGACAAGGTTCGTGGCTGACACCCCATAACAAAAGACAGATTAACAAGAGAAAATCATAACAAAATTGTTTAACTAGAGTTTgACATGACATGgGAGCCTTCAGAAATGAATACCCTCAAACCAGGGGAAAATTGTGATTTT
ST-E00144:68:H2LCKCCXX:4:1123:7780:49180 + CTCACTGATGACAAGGTTCGTGGCTGACACCCCATAACAAAAGACAGATTAACAAGAGAAAATCATAACAAAATTGTTTAACTAGAGTTTTACATGACAtGGGAGCctTCAGAAATGGATTACACTTTGAAAGCATATTcAagtgATTTG
ST-E00144:68:H2LCKCCXX:4:1123:7597:49321 + CTCACTGATGACAAGGTTCGTGGCTGACACCCCATAACAAAAGACAGATTAACAAGAGAAAATCATAACAAAATTGTTTAACTAGAGTTTTACATGACATGGGAGCcTTCAGAAATGGATTACACTTTGAAAGCATATTAAAGTGATTTG
ST-E00144:68:H2LCKCCXX:4:1123:7597:49356 + CTCACTGATGACAAGGTTCGTGGCTGACACCCCATAACAAAAGACAGATTAACAAGAGAAAATCATAACAAAATTGTTTAACTAGAGTTTTACATGACATGGGAGCCTTCAGAAATGGATTACACTTTGAAAGCATATTAAAGTGATTTG
ST-E00144:68:H2LCKCCXX:4:1123:7567:49584 + CTCACTGATGACAAGGTTCGTGGCTGACACCCCATAACAAAAGACAGATTAACAAGAGAAAATCATAACAAAATTGTTTAACTAGAGTTTTACATGACATGGGAGCCTTCAGAAATGGATTACACTTTGAAAGCATATTAAAGTGATTTG
ST-E00144:68:H2LCKCCXX:1:2217:3953:26449 + AAGGTTCGTGGCTGACACCCCATAACAAAAGACAGATTAACAAGAGAAAATCATAACAAAATTGTTTAACTAGAGTTTTACATGACATGGGAGCCTTCAGAAATGAATACCCTCAAACCAGGGGAAAATTgTGATtTTTTTTTCTTTtT
ST-E00144:68:H2LCKCCXX:6:2218:31876:12226+ ATAACAAAATTGTTTAACTAGAGTGTTACATGACATgGGAGCCTTCAGAAATGAATACCCTCAAACCAGGGGAAAATTGTGATTTTTTTTTGCTGTTtcTtGTcttTTGTTTTTTTTTTTTTTgtTTTgTTTTtT
ST-E00144:68:H2LCKCCXX:3:1101:2279:32479 + TTGTTTAACTAGAGTTTTACATGACATGGGAGCCTTCAGAAATGAATACCCTCAAACCAGGGGAAAATTGTGATTTTTTTTTTCTTTTTCTTGTCTTTTTTTTTTTTTTTTTtttTTTtgTTtTttttagtatgGgcagTcatGTA
ST-E00144:68:H2LCKCCXX:1:1104:14377:6319 - TCTTTCTCTCACTGATGACAAGGTTCGTGGCTGACACCCCATAACAAAAGACAGATTAACAAGAGAAAATCATAACAAAATTGTTTAACTAGAGTTTTACATGACATGGGAGCCTTCAGAAATGAATACCCTCAAACCAGGGGAAAATTG
ST-E00144:68:H2LCKCCXX:8:2212:9414:52819 - tCACTGATGACaAgGTTCGtgGCTGACACcCCATAACAAAAGACAGATTAACAAGAGAAAATCAtAACAAAATTGTTTAACTAGAGTTTTACATGACATGGGAGCCTTCAGAAATGAATACCCtCAAACCAGGGGAAAATTGTGATTTTT
ST-E00144:68:H2LCKCCXX:4:1123:7780:49180 - AAGGTTCGTGGCTGACACCCCATAACAAAAGACAGATTAACAAGAGAAAATCATAACAAAATTGTTTAACTAGAGTTTTACATGACATGGGAGCCTTCAGAAATGGATTACACTTTGAAAGCATATTAAAGTGATTTGGAATCCTGGCAT
ST-E00144:68:H2LCKCCXX:4:1123:7597:49321 - AAGGTTCGTGGCTGACACCCCATAACAAAAGACAGATTAACAAGAGAAAATCATAACAAAATTGTTTAACTAGAGTTTTACATGACATGGGAGCCTTCAGAAATGGATTACACTTTGAAAGCATATTAAAGTGATTTGGAATCCTGGCAT
ST-E00144:68:H2LCKCCXX:4:1123:7597:49356 - AAGgTTCGTgGCTGAcACCCCATAACAAAAGACAGATTAACAAGAGAAAATCATAACAAAATTGTTTAACTAGAGTTTTACATGACATGGGAGCCTTCAGAAATGGATTACACTTTGAAAGCATATTAAAGTGATTTGGAATCCTGGCAT
ST-E00144:68:H2LCKCCXX:4:1123:7567:49584 - AAGGTTCGTGGCTGACACCCCATAACAAAAGACAGATTAACAAGAGAAAATCATAACAAAATTGTTTAACTAGAGTTTTACATGACATGGGAGCCTTCAGAAATGGATTACACTTTGAAAGCATATTAAAGTGATTTGGAATCCTGGCAT
ST-E00144:68:H2LCKCCXX:8:1113:13555:39634- GTgGCTGACACCCCATAACAAAAGACAGATTAACAAGAGAAAATCATAACAAAATTGTTTAACTAGAGttTTACATGACATgggAgCCTTCAgAAATGAATACCCTCAAACCAGGGGAAAATTGTGATTTTTTTTTGCTTTTTCTTGTCT
ST-E00144:68:H2LCKCCXX:8:1113:13352:40126- GTGGCTGACACCCCATAACAAAAGACAGATTAACAAGAGAAAATCATAACAAAATTGTTTAACTAGAGTTTTACATGACATGGGAGCCTTCAGAAATGAATACCCTCAAACCAGGGGAAAATTGTGATTTTTTTTTTCTTTTTCTTGTCT
```

```
ST-E00192:51:H2KG3CCXX:3:2203:16245:8112 + AGGGGAGAAAAGGTATTATCTTTCTCTCACTGATGACAAGGTTCGTGGCTGACACCCCATAACAAAAGACAGATTAACAAGAGAAAATCATAACAAAATTGTTTAACTAGAGTTTTACATGACATGGgAGCCTTCAGAAATGAATACCCcT
ST-E00192:51:H2KG3CCXX:3:2214:3172:4034  + GGGAGAAAAGGTATTATCTTTCTCTCACTGATGACAAGGTTCGTGGCTGACACCCCATAACAAAAGACAGATTAACAAGAGAAAATCATAACAAAATTGTTTAACTAGAGTTTTACATGACATGGGAGCCTTCAGAAATGAATACCCTCA
ST-E00192:51:H2KG3CCXX:2:1111:15240:25218+ CTCACTGATGACAAGGTTCGTGGCTGACACCCCATAACAAAAGACAGATTAACAAGAGAAAATCATAACAAAATTGTTTAACTAGAGTTTTACATGACATGGGAGCCTTCAGAAATGAATACCCTCAAACCAGGGGAAAATTGTGATTTT
ST-E00192:51:H2KG3CCXX:3:1216:30831:37243+ ACTGATGACAAGGTTCGTGGCTGACACCCCATAACAAAAGACAGATTAACAAGAGAAAATCATAACAAAATTGTTTAACTAGAGTTTTACATGACATGGGAGCCTTCAGAAATGAATACCCTCAAACCAGGGGAAAATTGTGATTTTTTT
ST-E00192:51:H2KG3CCXX:2:2121:2268:56159 + GTGGCTGAcACCCCaTAACAAAAGAcAGATTAACAAGAGAAAATCATAAcAAAaTtGtTtAAcTAGagtttTAcATGACATGGGaGccTTcAGAAATGAATACcaTCAAacCAggGGaAAATTGTGaTTTTTT
[illegible]
```

```
ST-E00192:51:H2KG3CCXX:2:2121:2218:56212 +   GTGGCTGACACCCCATAACAAAAGACAGATTAACAAGAGAAAATCATAACAAAATTGTTTAACTAGAGTTTTACATGACATGGGAGCCTTCAGAAATGAATACCCTCAAACCAGGGGAAAATTGTGATTTTTTTTTCTTTTTCTTgT
ST-E00192:51:H2KG3CCXX:3:2221:27024:47844+   CAGATTAACAAGAGAAAATCATAACAAAATTGTTTAACTAGAGTTTTACATGACATGGGAGCCTTCAGAAATGAATACCCTCAAACCAGGGGAAAATTaTGATTTTTTTTTTCTTTTTCTTGTCTTTTTTTTTTTTTTTTTGtTTTtgtT

ST-E00192:51:H2KG3CCXX:3:2221:27045:47844+   CAGATTAACAAGAGAAAATCATAACAAAATTGTTTAACTAGAGTTTTACATGACATGGGAGCCTTCAGAAATGAATACCCTCAAACCAGGGGAAAATTgTGATTTTTTTTTTCTTTTTCTTGTCTTTTTTTTTTTTTTTTTGTTTTtgtT
ST-E00192:51:H2KG3CCXX:3:2221:27004:47879+   CAGATTAACAAGAGAAAATCATAACAAAATTGTTTAACTAGAGTTTTACATGACATGGGAGCCTTCAGAAATGAATACCCTCAAACCAGGGGAAAATTcTGATTTTTTTTTTCTTTTTCTTGTCTTTTTTTTTTTTTTTTTtgtTTTGgTT
ST-E00192:51:H2KG3CCXX:8:1110:26293:7972 +   ATTAACAAGAGAAAATCATAACAAAATTGTTTAACTAGAGTTTTACATGACATGGGAGCCTTCAGAAATGAATACCCTCAAACCAGGGGAAAATTGTGATTTTTTTTTTCTTTTTCTTGTCTTTTTTTTTTTTTTTTTTTttTTTggtTTT
ST-E00192:51:H2KG3CCXX:8:1212:22041:48283+   TTAACAAGAGAAAaTCATAACAAAATTGTTTAACTAGAGTTTTACATGACATGGGAGCCTTCAGAAATGAATACCCTCAAACCAGGGGAAAATTGTGATTTTTTTTTTCTtTTTCTTcTcTTTTTTTTTTTTTTTTTTTTTTTTGtT
ST-E00192:51:H2KG3CCXX:8:1212:19006:49074+   TTAACAAGAGAAAATCATAACAAAATTGTTTAACTAGAGTTTTACATGACATGGGAGCCTTCAGAAATGAATACCCTCAAACCAGGGGAAAATTGTGATTTTTTTTTTCTTTTTCTTGTCTTTTTTTTTTTTTTTTTTTTTTTTtTTTT
ST-E00192:51:H2KG3CCXX:3:2108:5861:33340 +   AAGAGAAAATCATAACAAAATTGTTTAACTAGAGTTTTACATGACATGGGAGCCTTCAGAAATGAATACCCTCAAACCAGGGGAAAATTGTGATTTTTTTTTTCTTTTTCTTGTCTTTTTTTTTTTTTTTTTTTTTTTTtTTTTTTTTTTT
ST-E00192:51:H2KG3CCXX:5:1218:27572:54032+   ATAACAAAATTGTTTAACTAGAGTTTTACATGACATGGGAGCCTTCAGAAATGAATACCCTCAAACCAGGGGAAAATTGTGATTTTTTTTTTCTTTTTCTTGTCTTTTTTTTTTTTTTTTTTttTTTtgTTTTTTT
ST-E00192:51:H2KG3CCXX:1:2123:25624:19153+   AAAATTGTTTAACTAGAGTTTTACATGACATGGGAGCCTTCAGAAATGAATACCCTCAAACCAGGGGAAAATTGTGATTTTTTTTTTCTTTTTCTTGTCTTTTTTTTTTTTTTTTTTTTTTTTTTTTTTTTTTTTTTTTtttTTtgTTT
ST-E00192:51:H2KG3CCXX:8:1202:14936:9536 +   AGTTTTACATGACATGGGAGCCTTCAGAAATGAATACCCTCAAACCAGGGGAAAATTGTGATTTTTTTTTTCTTTTTCTTGTCTTTTTTTTTTTTTTTTTGtt******TTaggTTtTATGAagAAcggGctgttATgTAGAACTacaagttgacA
ST-E00192:51:H2KG3CCXX:1:2214:8815:10995 -   GTATTATCTTTTTCTCACTGATGACAAGGTTCGTGGCTGACACCCCATAACAAAAGACAGATTAACAAGAGAAAATCATAACAAAATTGTTTAACTAGAGTTTTACATGACATGGGAGCCTTCAGAAATGAATACCCTCAAACCAGGGGA
ST-E00192:51:H2KG3CCXX:1:2214:8835:10995 -   GTATTATCTTTTTCTCACTGATGACAAGGTTCGTGGCTGACACCCCATAACAAAAGACAGATTAACAAGAGAAAATCATAACAAAATTGTTTAACTAGAGTTTTACATGACATGGGAGCCTTCAGAAATGAATACCCTCAAACCAGGGGA
ST-E00192:51:H2KG3CCXX:8:1109:15433:13879-   ATCTTTTTCTCACTGATGACAAGGTTCGTGGCTGACACCCCATAACAAAAGACAGATTAACAAGAGAAAATCATAACAAAATTGTTTAACTAGAGTTTTACATGACATGGGAGCCTTCAGAAATGAATACCCTCAAACCAGGGGAAAATT
ST-E00192:51:H2KG3CCXX:2:2121:16499:60871-   CTCACTGATGACAAGGTTCGTGGCTGACACCCCATAACAAAAGACAGATTAACAAGAGAAAATCATAACAAAATTGTTTAACTAGAGTTTTACATGACATGGGAGCCTTCAGAAATGAATACCCTCAAACCAGGGGAAAATTGTGATTTT
ST-E00192:51:H2KG3CCXX:6:2121:6379:3717  -   TCACTGATGACAAGGTTCGTGGCTGACACCCCATAACAAAAGACAGATTAACAAGAGAAAATCATAACAAAATTGTTTAACTAGAGTTTTACATGACATGGGAGCCTTCAGAAATGAATACCCTCAAaCCAGGGGAAAATTGTGATTTTT
ST-E00192:51:H2KG3CCXX:3:1204:13616:56933-   CACTGATGACAAGGTTCGTGGCTGACACCCCATAACAAAAGACAGATTAACAAGAGAAAATCATAACAAAATTGTTTAACTAGAGTTTTACATGACATGGGAGCCTTCAGAAATGAATACCCTCAAACCAGGGGAAAATTGTGATTTTTT
ST-E00192:51:H2KG3CCXX:6:2102:12134:71771-   ATGACAAGGTTCGTGGcTgACACccCATAACAAAAGACAGATTAACAAGAGAAAATCATAACAAAATTGTTTAACTAGAGTTTTACATGACATGGGAGCCTTCAGAAATGAATACCCTCAAACCAGGGGAAAATTGTGATTTTTTTTTTC
ST-E00192:51:H2KG3CCXX:6:2102:12276:71771-   ATGACAAGGTTCGTGgCTGACACCCCATAACAAAAGACAGATTAACAAGAGAAAATCATAACAAAATTGTTTAACTAGAGTTTTACATGACATGGGAGCCTTCAGAAATGAATACCCTCAAgCCAGGGGAAAATTGTGATTTTTTTTTTC
ST-E00192:51:H2KG3CCXX:5:1104:5516:17447 -   AgAgAAaAacAtAACAAAAttgaaaAactAgAgtTtaACATAAAATgGGAGCCTtCAgAAAtGAATACCCTCAAACCAGGGGAAAATTGTGATTTTTTTTTTCTTTTTCTTGTC*TTTTTTTTTTTTTTTTTTTTTTGGCTTAG
ST-E00192:51:H2KG3CCXX:1:2101:21076:52591-   AAAaccAcAAAAAAAatGTtaAaCaAgAgttttAaAtaAtATggGAGCCTTtagAAatGAaTaCCCTcAaCCAGGGGAAAATTGTGATTTTTTTTTCTCTTTTTCTTGTcTTTTTTTTTTTTTTTTTTTTTTTGGCTTAGGTTCT
ST-E00192:51:H2KG3CCXX:1:2101:21269:52678-   AAAaaaaataaAAaaAttGtttaaaAaaAgtAaaaaAaaAcaggagAgcattAAGAAATaAAtaCCcTcAagccgGGGGAAAaTtgtGATTTTTTTTTCTTTTTCTTGTcTTTTTTTTTTTTTTTTTTTTTTTGGCTTAGGTTCT
```

```
                                                 79474040            79474060            79474080            79474100            79474120            79474140            79474160            79474180            79474200            79474220            79474240            79474260            79474280            79474300            79474320
chr9:79474030-79474323                        TGGATTGAAAGAGATACTACACTGCTTTGTCATCTAGATACACTTCCAAAATAGATGGACAGAATCTTTTCATAATCAATGTAAAAAAGAAAGTATATTGACTATGCCAGGATTCCAAATCACTTTAATATGCTTTCAAAGTGTAATCCTATATACCGCTAAAATGAGACTTCTTAACTTCTTATCTCTTTTTAAAAGCCTACAAAATGCCTACATATTCAGATTCAATGGCCCATTAAATGAAGCTGCACTTGTTAGGCAAAAGCACTTTCAAGCCACCCAGAAGAATTGACC

ST-E00144:68:H2LCKCCXX:3:2114:17788:40179+   TGGATTGAAAGAGATACTACACTGCTTTGTCATCTAGATACACTTCCAAAATAGATGGACAGAATCTTTTCATAATCAATGTAAAAAAGAAAGTATATTGACTATGCCAGGATTCCAAATCACTTTAATATGCTTTCAAAGTGTAATCcT
ST-E00144:68:H2LCKCCXX:1:1205:26507:35942+   CTGCTTTGTCATCTAGATACACTTCCAAAATAGATGGACAGAATCTTTTCATAATCAATGTAAAAAAGAAAGTATATTGACTATGCCAGGATTCCAAATCACTTTAATATGCTTTCAAAGTGTAATCCTATATACCGCTAAAATGAGAcT
ST-E00144:68:H2LCKCCXX:8:1103:25015:47668+   TTGTCATCTAGATACACTTCCAAAATAGATGGACAGAATCTTTTCATAATCAATGTAAAAAAGAAAGTATATTGACTATGCCAGGATTCCAAATCACTTTAATATGCTTTCAAAGTGTAATCCTATATACCGCTAAAATGAGACTTCTTA
ST-E00144:68:H2LCKCCXX:8:2224:2705:11945 +   TAGATACACTTCCAAAATAGATGGACAGAATCTTTTCATAATCAATGTAAAAAAGAAAGTATATTGACTATGCCAGGATTCCAAATCACtTTAATATGCtTTCAAAGTGTAATCCtaTATACaccTAAAATGAgACTacTTaAaTtcTga
ST-E00144:68:H2LCKCCXX:6:1203:23086:46930+   CCAAAATAGATGGACAGAATCTTTTCATAATCAATGTAAAAAAGAAAGTATATTGACTATGCCAGGATTCCAAATCACTTTAATATGCTTTCAAAGTGTAATCCTATATACCGCTAAAATGAGACTTCTTAACTTcTTATCTCTTTTTAA
ST-E00144:68:H2LCKCCXX:8:1106:23025:37225+   ATAGATGGACAGAATCTTTTCATAATCAATGTAAAAAAGAAAGTATATTGACTATGCCAGGATTCCAAATCACTTTAATATGCTTTCAAAGTGTAATCCTATATACCGCTAAAATGAGACTTCTTAACTTCTTATCTCTTTTTAAAAgC
ST-E00144:68:H2LCKCCXX:7:1222:10206:40970+   TAGaTGGACAGAATCTTTTCATAATCAATGTAaAAAAGAAAGTATATTGACTATGCCAGGaTtCCAAATCACTTTAATATGCTTTCAAAGTGTaATCCTATATACCGCtAAAATGAGACttCtTAACTTcTTATCTCTTTTTAaaagcct
ST-E00144:68:H2LCKCCXX:7:2106:22315:5053 +   GACTATGCCAGGATTCCAAATCACTTTAATATGCTTTCAAAGTGTAATCCTATaTACCGCTAAAATGAGACTTCctaACTTCTTATCTCTTTTTAAAAGCCTACAAAATGCCTACATATtCaGAtTCAATGGCCcatTAAATGaAGCTGC
ST-E00144:68:H2LCKCCXX:4:1205:14570:42447+   AGGATTCCAAATCACTTTAATATGCTTTCAAAGTGTAATCCTATATACCGCTAAAATGAGACTTCTTAACTTCTTATCTCTTTTTAAAAGCCtACAAAatGCCTACATATTCAGATTCAATGGCCCATTAAATGAAGCTGCACTTGTTAG
ST-E00144:68:H2LCKCCXX:4:1114:11708:63262+   ATTCCAAATCACTTTAATATGCTTTCAAAGTGTAATCCTATATACCGCTAAAATGAGACTTCTTAACTTCTTATCTCTTTTTAAAAGCCTACAAAATGCCTACATATTCAGATTCAATGGCCCATTAAATGAAGCTGCACTTGTTAGGCA
ST-E00144:68:H2LCKCCXX:3:2213:23380:71138+   GTGTAATCCTATATACCGCTAAAATGAGACTTCTTAACTTCTTATCTCTTTTTAAAAGCCTACAAAATGCCTACATATTCAGATTCAATGGCCCATTAAATGAAGCTGCACTTGTTAGGCAAAaGCACTTTCAAGCCATCCAGaAgAATT
ST-E00144:68:H2LCKCCXX:3:2213:24091:71700+   GTGaAATCCTATATACCGCTAAAATGAGACTTCTTAACTTCTTATCTCTTTTTAAAAGCCTACAAAATGCCTACATATTCAGATTCAATGGCCCATTAAATGAAGCTGCACTTGTTAGGCAAAAGCACTTTCAaGCCAtCCAGAAGAATT
ST-E00144:68:H2LCKCCXX:1:2218:15007:33463+   AATCCTATATACCGCTAAAATGAGACTTCTTAACtTCTTATCTCTTTTTAAAAGCCTACAAAATGCCTACATATTCAGATTCAATGGCCCATTAAATGAAGCTGCACTTgtTAGGCAAAAGCACTTTCAAGCCACCcaGAAGAAttgACC
ST-E00144:68:H2LCKCCXX:3:2109:5486:24673 -   gAttGAAAGAGATACTACactgCTTTGTCATCTAgATACAcTtCCAAAATAGAtGGACAGAATCtTTTCATAATCAAtgTAAAAAAGAAAGTATATTGACTATGCCAGGATTCCAAATCACTTTAATATGCTTTCAAAGTGTAATCCTAT
ST-E00144:68:H2LCKCCXX:3:2201:18985:52344-   GAtTGAAAGAGATACTACACTGCTTTGTCATCTAGATACacTTCCAAAATAGATGGACAGAAtCTTTTCATAATCAATGTAAAAAAGAAAGTATATTGACTATGCCAGGATTCCAAATCACTTTAATATGCTTTCAAAGTGTAATCCTAT
ST-E00144:68:H2LCKCCXX:7:1118:26588:39634-   GAGATACTACACTGCTTTGTcATCTAGATACACTTCCAAAAtAGATGGACAGAATCTTTtCATAATCAATGTAAAAAAGAAAGTATATTGACTATGCCAGGATTCCAAATCACTTTAATATGCTTTCAAAGTGTAATCCTATATACCGCT
ST-E00144:68:H2LCKCCXX:7:1209:15534:29982-   GAGATACTACACTGCTTTGTCATCTAGATACACTTCCAAAATAGATGGACAGAATCTTTTCATAATCAATGTAAAAAAGAAAGTATATTGACTATGCCAGGATTCCAAATCACTTTAATATGCTTTCAAAGTGTAATCCTATATACCGCT
ST-E00144:68:H2LCKCCXX:7:2223:4298:47264 -   ATACTACACTGCTTTGTCATCTAGATACACTTCCAAAATAGATGGACAGAATCTTTTCATAATCAATGTAAAAAAGAAAGTATATTGACTATGCCAGGATTCCAAATCACTTTAATATGCTTTCAAAGTGTAATCCTATATACCGCTAAA
ST-E00144:68:H2LCKCCXX:6:1121:11332:44925-   TACACTGCTTTGTCATCTAGATACACTTCCAAAATAGATGGACAGAATCTTTTCATAATCAATGTAAAAAAGAAAGTATATTGACTATGCCAGGATTCCAAATCACTTTAATATGCTTTCAAAGTGTAATCCTATATACCGCTAAAATGA
ST-E00144:68:H2LCKCCXX:6:1121:11373:44925-   TACACTGCTTTGTCATCTAGATACACTTCCAAAATAGATGGACAGAATCTTTTCATAATCAATGTAAAAAAGAAAGTATATTGACTATGCCAGGATTCCAAATCACTTTAATATGCTTTCAAAGTGTAATCCTATATACCGCTAAAATGA
ST-E00144:68:H2LCKCCXX:4:1208:14804:20454-   ACACTGCTTTGTCATCTAGATACACTTCCAAAATAGATGGACAGAATCTTTTCATAATCAATGTAAAAAAGAAAGTATATTGACTATGCCAGGATTCCAAATCACTTTAATATGCTTTCAAAGTGTAATCCTATATACCGCTAAAATGAG
ST-E00144:68:H2LCKCCXX:3:1103:22315:45101-   TCATCTAGATACACTTCCAAAATAGATGGACAGAATCTTTTCATAATCAATGTAAAAAAGAAAGTATATTGACTATGCCAGGATTCCAAATCACTTTAATATGCTTTCAAAGTGTAATCCTATATACCGCTAAAATGAGACTTCTTAACT
ST-E00144:68:H2LCKCCXX:7:2220:28364:32057-   CTagatacacttcCAAAATaGATggACAgAATCTTTTCATAATCAATGTAAAAAAGAAAGTATATTGACTATGCCAGGATTCCAAATCACTTTAATATGCTTTCAAAGTGTAATCCTATATACCGCTAAAATGAGACTTCTTAACTTCTT
ST-E00144:68:H2LCKCCXX:1:1110:7283:38720 -   ACACTTCCAAAATAGATGGACAGAATCTTTTCATAAgCAATGTAAAAAAGAAAGTATATTGACTATGCCAGGATTCCAAATCACTTTAATATGCTTTCAAAGTGTAATCCTATATACCGCtAAAATGAGACTTCTTAACTTCTTATCTCT
ST-E00144:68:H2LCKCCXX:4:1209:13383:39194-   CAAAATAGATGGACAGAATCTTTTCATAATCAATGTAAAAAAGAAAGTATATTGACTATGCCAGGATTCCAAATCACTTTAATATGCTTTCAAAGTGTAATCCTATATACCGCTAAAATGAGACTTCTTAACTTCTTATCTCTTTTTAAA
ST-E00144:68:H2LCKCCXX:7:1118:8034:58550 -   GAAAGTATATTGACTATGCCAgGATTCCAAATCACTTTAATATGCTTTCAAAGTGTAATCCTATATACCGCTAAAATGAGACTTCTTAACTTCTTATCTCTTTTTAAAAGCCTACAAAATGCCTACATATTCAGATTCAATGGCCCATTA
ST-E00144:68:H2LCKCCXX:1:1205:26507:35942-   gAcTATgccAggattccaaATCccTtTAATATGCTTTCAAAGTGTAATCCTATATACCGCTAAAATGAGACTTCTTAACTTCTTATCTCTTTTTAAAAGCCTACAAAATGCCTACATATTCAGATTCAATGGCCCATTAAATGAAGCTGC
ST-E00144:68:H2LCKCCXX:8:2211:7820:13386 -   AactttAatccTAtAtaccGCTAAAatgAcACttCTTAACttCTtATCTCTTTttAAAAGCCTACAAaATGCctACATATTCAGATtCAATGGCCCatTAAatGAAGCTGCACtTGTTAGGCAAAAGCACTTTCAAGCCACCCAGAAgAA

ST-E00192:51:H2KG3CCXX:6:1119:6836:22651 +   GGATTGAAAGAGATACTACACTGCTTTGTCATCTAGATACACTTCCAAAATAGATGGACAGAATCTTTTCATAATCAATGTAAAAAAGAAAGTATATTGACTATGCCAGGATTCCAAATCACTTTAATATGCTTTaAAAGTGTAATCCTA
ST-E00192:51:H2KG3CCXX:3:1117:12398:71911+   GAAAGAGATACTACACTGCTTTGTCATCTAGATACACTTCCAAAATAGATGGACAGAATCTTTTCATAATCAATGTAAAAAAGAAAGTATATTGACTATGCCAGGATTCCAAATCACTTTAATATGCTTTCAAAGTGTAATCCTATATAC
ST-E00192:51:H2KG3CCXX:3:1117:12337:71946+   GAAAGAGATACTACACTGCTTTGTCATCTAGATACACTTCCAAAATAGATGGACAGAATCTTTTCATAATCAATGTAAAAAAGAAAGTATATTGACTATGCCAGGATTCCAAATCACTTTAATATGCTTTCAAAGTGTAATCCTATATAC
ST-E00192:51:H2KG3CCXX:2:2110:9932:66127 +   AGATACTACACTGCTTTGTCATCTAGATACACTTCCAAAATAGATGGACAGAATCTTTTCATAATCAATGTAAAAAAGAAAGTATATTGACTATGCCAGGATTCCAAATCACTTTAATATGCTTTCAAaGTGTaATCCTATATACCGCTA
ST-E00192:51:H2KG3CCXX:2:2110:10693:66321+   AGATACTACACTGCTTTGTCATCTAGATACACTTCCAAAATAGATGGACAGAATCTTTTCATAATCAATGTAAAAAAGAAAGTATATTGACTATGCCAGGATTCCAAATCACTTTAATATGCTTTCAAAGTGTAATCCTATATACCGCTA
ST-E00192:51:H2KG3CCXX:2:2114:10256:54559+   CAaCTAGATAcAcTtccAAAATAGATgGAcAGAATcTtTTaAtaATCAATGTAAAAAAGAAAGTATATTGACTATGcCAGGATTccAAATCACTTTAATATGCtTtCAAAGTGTAATcatATATACcgcTAAAATGAgACTTCaTAACTT
ST-E00192:51:H2KG3CCXX:2:2114:10277:54630+   CATCTAGATACACTTCCAAAATAGATGGACAGAATCTTTTCATAATCAATGTAAAAAAGAAAGTATATTGACTATGCCAGGATTCCAAATCACTTTAATATGCTTTCAAAGTGTAATCcTATATACCGCTAAaATGAGACTTCTTAACTT
ST-E00192:51:H2KG3CCXX:8:2215:19716:9273 +   ATACACTTCCAAAATAaATGGACAGtATCTTTTCATAATCAATGTAAAAAAGAAAGTATATTGACTATGCCAGGATTCCAAATCACTTTAATATGCTTTCtAAGTGTAATCCTATATACCGGTAAAgTGAGACTTCTTAACTTCTTATCT
ST-E00192:51:H2KG3CCXX:6:2220:16763:22616+   ACACTTCCAAAATAGATGGaCAGaaTCTTTTCATAATCAatGTAAAAAAGaaAGTATATTgaCTAtGCCAGGATtCCAacTCACTTTAATATGCTttCcAAGTGTAATCCTATaTACCGCTAAcatgAGaCTtcTtAACTTCTTATCTCT
ST-E00192:51:H2KG3CCXX:1:2105:23309:65846+   ATGGACAGAATCTTTTCATAATCAATGTAAAAAAGAAAGTATATTGACTATGCCAGGATTCCAAATCACTTTAATATGCTTTCAAAGTGTAATCCTATATACCGCTAAAATGAGACTTCTTAACTTCTTATCTCTTTTTAAAAGCCaACA
ST-E00192:51:H2KG3CCXX:1:2105:23248:65916+   ATGGACAGAATCTTTTCATAATCAATGTAAAAAAGAAAGTATATTGACTATGCCAGGATTCCAAATCACTTTAATATGCTTTCAAAGTGTAATCCTATATACCGCTAAAATGAGACTTCTTaACTTCTTATCTCTTTTTAAAAGCCgaCA
ST-E00192:51:H2KG3CCXX:2:2201:7516:12085 +   TGGACAGAATCTTTTCATAATCAATGTAAAAAAGAAAGTATATTGACTATGCCAGGATTCCAAATCACTTTAATATGCTTTCAAAGTGTAATCCTATATACCGCTAAAATGAGACTTCTTAACTTCTTATCTCTTTTTAAAAGCCTACAA
ST-E00192:51:H2KG3CCXX:2:2221:25197:38878+   CATAATCAATGTAAAAAAGAAAGTATATTGACTATGCCAGGATTCCAAATCACTTTAATATGCTTTCAAAGTGTAATCCTATATACCGCTAAAATGAGACTTCTTAACTTCTTATCTCTTTTTAAAAGCCTACAAAATGcCTACATATTC
ST-E00192:51:H2KG3CCXX:8:1105:3710:70153 +   CAATGTAAAAAAGAAAGTATATTGACTATGCCAGGATTCCAAATCACTTTAATATGCTTTCAAAGTGTAATCCTATATACCGCTAAAATGAGACTTCTTAACTTCTTATCTCTTTTTAAAAGCCTACAAAATGCcTACATATTCAGATTC
ST-E00192:51:H2KG3CCXX:8:2107:31734:15180+   CAATGTAAAAAAGAAAGTATATTGACTATGCCAGGATTCCAAATCACTTTAATATGCTTTCAAAGTGTAATCCTATATACCGCTAAAATGAGACTTCTTAACTTCTTATCTCTTTTTAAAAGCCTACAAAAtGCCTACATATTCAGAtTC

ST-E00192:51:H2KG3CCXX:1:1118:9333:8060  +   TGTAAAAAAGAAAGTATATTGACTATGCCAGGATTCCAAATCACTTTAATATGCTTTCAAAGTGTAATCCTATATACCGCTAAAATGAGACTTCTTAACTTCTTATCTCTTTTTAAAAgCCTACAAAATGCCTACATATTCAGATTCAAT
ST-E00192:51:H2KG3CCXX:1:1118:9373:8060  +   TGTAAAAAAGAAAGTATATTGACTATGCCAGGATTCCAAATCACTTTAATATGCTTTCAAAGTGTAATCCTATATACCGCTAAAATGAGACTTCTTAACTTCTTATCTCTTTTTAAAAGCCTACAAAATGCCTACATATTCAGATTCAAT
ST-E00192:51:H2KG3CCXX:8:1123:15616:47176+   TGTAAAAAAGAAAGTATATTGACTATGCCAGGATTCCAAATCACTTTAATATGCTTTCAAAGTGTAATCCTATATACCGCTAAAATGAGACTTCTTAACTTCTTATCTCTTTTTAAAAGCCTACAAAATGCCTACATATTCAGATTCAAT
ST-E00192:51:H2KG3CCXX:2:2222:8176:27257 +   AAAAAAGAAAGTATATTGACTATGCCAGGATTCCAAATCACTTTAATATGCTTTCAAAGTGTAATCCTATATACCGCTAAAATGAGACTTCTTAACTTCTTATCTCTTTTTAAAAGCCTACAAAATGccTACATATTCAGATTCAATGGC
ST-E00192:51:H2KG3CCXX:6:2101:15961:17395+   AAGAAAGTATATTGACTATGCCAGGATTCCAAATCACTTTAATATGCTTTCAAAGTGTAATCCTATATACCGCTAAaATGAGACTTCTTAACTTCTTATCTCTTTTTAAAAGCCTACAAAATGCCTACATATTCAGATTCAATGGCcCAT
ST-E00192:51:H2KG3CCXX:6:2101:16001:17430+   AAGAAAGTATATTGACTATGCCAGGATTCCAAATCACTTTAATATGCTTTCAAAGTGTAATCCTATATACCGCTAAAATGAGACTTCTTAACTTCTTATCTCTTTTTAAAAGCCTACAAAATGCCTACATATTCAGATTCAATGGCCCAT
ST-E00192:51:H2KG3CCXX:8:1106:24060:29842+   AGGATTCCAAATCACTTTAATATGCTTTCAAAGTGTAATCCTATATACCGCTAAAATGAGACTTCTTAACTTCTTATCTCTTTTTAAAAGCCTACAAAATGCCTACATATTCAGATTCAATGGCCCATTAAATGAAGCTGCACTTGTTAG
ST-E00192:51:H2KG3CCXX:1:1102:32191:5317 +   TTCCAAATCACTTTAATATGCTtTCAaaGTGTAAtCCtaTATaCCGCtAAAATgAGACTtCtTAACTTctTATCTcTtTTTAAAAGCCTACAAAATGcCTgcataTTcagaTtCAATGtcccatTAAATGAaacTGCAgtcttTcgtcga
ST-E00192:51:H2KG3CCXX:1:1102:32160:5370 +   TTCCAAATCACTTTAATATGCTTTCAAAGTGTAATCCTATATACCGCTAAAATGAGACTTCTTAACTTctTaTCTCTTTTTAAAAGCCTACAAAATGCcTACATATTCAGATTCAATGGCCcATTAAAtGAAgatGCACTTGTTaggcaA
ST-E00192:51:H2KG3CCXX:3:2219:18448:69485+   CCAAATCACTTTAATATGCTTTCAAAGTGTAATCCTATATACCGCTAAaATGAGaCTTCTTAACTTCTTATCTCTTTTTAAAAGCCTACAAAATGCCTACATATTCAGATTCaATGGCCCATTAAATGAAGCTGCACTTGTTAGGCAAaA
ST-E00192:51:H2KG3CCXX:6:2112:7648:19838 +   ATATGCTTTCAAAGTGTAATCCTATATACCGCTAAAATGAGACTTCTTAACTTCTTATCTCTTTTTAAAAGCCTACAAAATGCCTACATATTCAGATTCAATGGCCCATTAAATGAAGCTgCACTTGTTAgGCAAAAGCACTTTCAAGCc
ST-E00192:51:H2KG3CCXX:6:2112:8105:20559 +   ATATGCTTTCAAAGTGTAATCCTATATACCGCTAAAATGAGACTTCTTAACTTCTTATCTCTTTTTAAAAGCCTACAAAATGCCTACATATTCAGATTCAATGGCCCATTAAATGAAGCTGCACTTGTTAGGCAAAAGCACTTTCAAGCC
ST-E00192:51:H2KG3CCXX:5:1115:10307:52889+   CTTTCAAAGTGTAATCCTATATACCGCTAAAATGAGACTTCTTAACTTCTTATCTCTTTTTAAAAGCCTACAAAATGCCTACATATTCAGATTCAATGGCCCATTAAATGAAGCTGCACTTGTTAGGCAAAAGCACTTTCAAGCCACCCA
ST-E00192:51:H2KG3CCXX:3:1224:31074:52678+   AAgTGTAATCCtATATACCGCTAAAATGAGACtTCtTAAcTtCTTATCTCTTTTTAAAAGCCTACAAAATgcctACATATTCAGATTCAATGGCCCATTAAATGAAGCTGCACTTGTTAGGCAAAAGCACTTTCAAGCCACCCAgAAGAA
ST-E00192:51:H2KG3CCXX:2:1117:22670:66602-   AGATACTACACTGCTTTGTCATCTAGATACACTTCCAAAATAGATGGACAGAATCTTTTCATAATCAATGTAAAAAAGAAAGTATATTGACTATGCCAGGATTCCAAATCACTTTAATATGCTTTCAAAGTGTAATCCTATATACCGCTA
ST-E00192:51:H2KG3CCXX:2:1117:22619:66620-   AGATACTACACTGCTttGTCAtCTAGATACACTtCCAAAATAGATgGACAGAATCtTTTCATAaTCAATGTAAAAAAGAAAGTATAtTGACTATGCCAGGATtCCAAATCACTTTAATATGCTTTCAAAGTGTAATCCTATATaCCgCTA
ST-E00192:51:H2KG3CCXX:6:2113:19422:16955-   AGATACTACACTGCTTTGTCATCTAGATACACTTCCAAAATAGATGGACAGAATCTTTTCATAATCAATGTAAAAAAGAAAGTATATTGACTATGCCAGGATTCCAAATCACTTTAATATGCTTTCAAAGTGTAATCCTATATACCGCTA
ST-E00192:51:H2KG3CCXX:3:2124:18762:39511-   TGCTTTGTCATCTAGATACACTTCCAAAATAGATGgACAGAAtCTTTTCATAATCAATGTAAAAAAGAAAGTATATTGACTATGCCAGGATTCCAAATCACTTTAATATGCTtTCAAAGTGTAATCCtaTATACCGCTAAAATGAGACTT
ST-E00192:51:H2KG3CCXX:3:2124:18772:39528-   TGCTTTGTCATCTAGATACACTTCCAAAATAGATGGACAGAATCTTTTCATAATCAATGTAAAAAAGAAAGTATATTGACTATGCCAGGATTCCAAATCACTTTAATATGCTTTCAAAGTGTAATCCTATATACCGCTAAAATGAGACTT
ST-E00192:51:H2KG3CCXX:5:1106:30628:4649 -   TGTCATCTAGATACACTTCCAAAATAGATGGACAGAATCTTTTCATAATCAATGTAAAAAAGAAAGTATATTGACTATGCCAGGATTCCAAATCACTTTAATATGCTTTCAAAGTGTAATCCTATATACCGCTAAAATGAGACTTCTTAA
ST-E00192:51:H2KG3CCXX:8:1109:25218:31811-   TCTAGATACACTtacAAAATAGATGGACAGAATCTTTTCATAAtCAAtGTAAAAAAGAAAGTATATTGACTATGCCAGGATTCCAAATCACTTTAATATGCTTTCAAAGTGTAATCCTATATACCGCTAAAATGAGACTTCTTAACTTCT
ST-E00192:51:H2KG3CCXX:3:1208:21523:48793-   CTTCCAAAATAGATGGACAGAATCTTTTCATAATCAATGTAAAAAAGAAAGTATATTGACTATGCCAGGATTCCAAATCACTTTAATATGCTTTCAAAGTGTAATCCTATATACCGCTAAAATGAGACTTCTTAACTTCTTATCTCTTTT
ST-E00192:51:H2KG3CCXX:6:2116:26395:60502-   TAATCAATGTAAAAAAGAAAGTATATTGACTATGCCAGGATTCCAAATCACTTTAATATGCTTTCAAAGTGTAATCCTATATACCGCTAAAATGAGACTTCTTAACTTCTTATCTCTTTTTAAAAGCCTACAAAATGCCTACATATTCAG
ST-E00192:51:H2KG3CCXX:2:1212:10378:55087-   AAAGAAAGTATATTGACTATGCCAGGATTCCAAATCACTTTAATATGCTTTCAAAGTGTAATCCTATATACCGCTAAAATGAGACTTCTTAACTTCTTATCTCTTTTTAAAAGCCTACAAAATGCCTACATATTCAGATTCAATGGCCCA
ST-E00192:51:H2KG3CCXX:1:1216:11160:22264-   ATGCCAGGATTCCAAATCACTTTAATATGCTTTCAAAGTGTAATCCTATATACCGCTAAAATGAGACTTCTTAACTTCTTATCTCTTTTTAAAAGCCTACAAAATGCCTACATATTCAGATTCAATGGCCCATTAAATGAAGCTGCACTT
```

chr13 74442419 + 3 chr8 130236562 - 1 CTX 23 36 54 82 0.566682615629984 0 0.937142857142857 0 1 chr13 74442289 185 chr8 130236495

AAAATATAAAATTAGCCGGGCATGGTGGCATGTGCCTGTAATCCTAGCTACTCGGGAGGCTGAGGCAGGAGAATCACTTGAACCCAGGAGGTGCAGGTTGCGGTGAGCCGAGATGGTACTCCAGCCTGGGCGACAGAGCGAGATTCCCTCCATTTCAAAATAAATAAATAAATGAATACATACAT

```
                                              74442280            74442300            74442320            74442340            74442360            74442380            74442400            74442420            74442440            74442460            74442480            74442500            74442520            74442540            74442560
chr13:74442274-74442561                       accccgtctctactaaaaatataaaattagccgggcatggtggcatgtgcctgtaatcctagctactcgggaggctgaggcaggagaatcacttgaacccaggaggtgcaggttgcggtgagccgagatggtactccagcctgggcaacaagagcaaaactccatctcaaaagaaaaaaGGCGGGGGCGGT**GGGGGGGGGGGGTGATTTCAGTACAAAGCCTACAGACATTATAAAAATATTAAGAtttttgttcgtttgtttttttgtttttgagacagagtctcact

ST-E00144:68:H2LCKCCXX:4:1116:5252:14178 +   CTACTAAAAatATAAAATTAGCCGGGCATGGTGGCATGTGCCTGTAaTCCTAGCTACTCGGgagGCtGaGGCaGGaGaATCACTTGaACCCAGgcgGTGCAGGTTGCGGtGAGCCGAGcTGGTACTcCAgCCtGGGCAACaaGA
ST-E00144:68:H2LCKCCXX:8:1109:3202:41603 +   CTAAAAATATAAAATTAGCCGGGCATGGTGGCATGTGCCTGTAATCCTAGCTACTCGGGAGGCTGAGGCAGGAGAATCACTTGAACCCAGGAGGTGCAGGTtGCGGTGAGCCGAGATGGTACTCCAGcCTGGGCAAcAAGAGCAAAACTC
ST-E00144:68:H2LCKCCXX:6:2217:12753:46015+   AAAATATAAAATTAGCCGGGCATGGTGGCATGTGCCTGTAATCCTAGCTACTCGGGAGGCTGAGGCAGGAGAATCACTTGAACCCAGGAGGTGCAGGTTGCGGTGAGCCGAGATGGTACTCCAGCCTGGGCGACAGAGCGAGATtCCCTC
ST-E00144:68:H2LCKCCXX:6:2217:11972:48389+   AAAATATAAAATTAGCCGGGCATGGTGGCATGTGCCTGTAATCCTAGCTACTCGGGAGGCTGAGGCAGGAGAATCACTTGAACCCAGGAGGTGCAGGTTGCGGTGAGCCGAGATGGTACTCCAGCCTGGGCGACAGAGCGAGATTCCCTC
ST-E00144:68:H2LCKCCXX:1:1114:12601:43044+   AtATAAAATTAGCCGGGCATGGTGGCATGTGCCTGTAATCCTAGCTACTCGGGAGGCTGAGGCAGGAGAATCACTTGAACCCAGGAGGTGCAGGTTGCGGTGAGCCGAGATGGTACTCCAGCCTGGGCAaCAAGAGCAAAACTccATCTC
ST-E00144:68:H2LCKCCXX:4:1213:31896:22212+   CATGGTGGCATGTGCCTGTAATCCTAGCTACTCGGGAGGCTGAGGCAGGAGAATCACTTGAACCCAGGAGGtGCaGGTTGCGGTGAGCCGAGATGGtcCtCCaGCCTGGGcAgCaaGAGCAAAACcccatCtcaaaAGAAaAAcGgtgGg
ST-E00144:68:H2LCKCCXX:8:2108:20386:19118+   TGGCATGTGCCTGTAATCCTAGCTACTCGGGAGGCTGAGGCAGGAGAATCACTTGAACCCAGGAGGTGCAGGtTGCGGTGAGCCGAGATGGTACTCCAGCCTGGGCAACAAGAGCAAAACTCCATCTCAAAAGAAAAAAGGTGGGGGCGG
ST-E00144:68:H2LCKCCXX:1:1112:12226:10257+   GGCATGTGCCTGTAATCCTAGCTACTCGGGAGGCTGAGGCAGGAGAATCACTTGAACCCAGGAGGTGCAGGTTGCGGtGAGCCGAGATGGTACTCCAGCCTGGGCAACAAGAGCAAAACTCCATCTCAAAAGAAAAAAGGTGGGGGCGG
ST-E00144:68:H2LCKCCXX:6:2202:31226:56001+   [illegible]
```

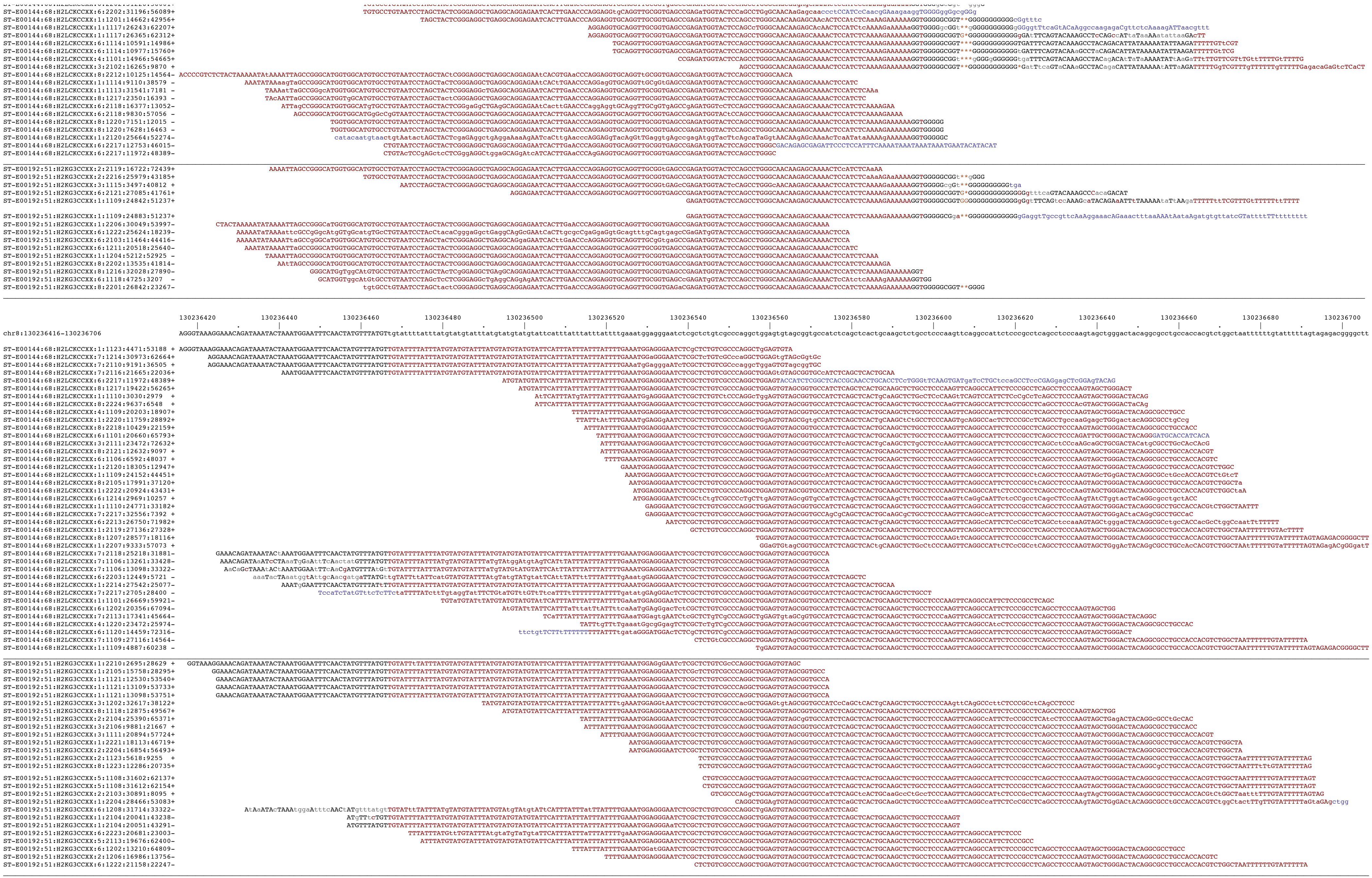

chr10 58580022 + 5 chr16 76683671 - 0 CTX 27 22 57 0 0.888235294117647 0 1 0 1 chr10 58579930 150 chr16 76683615

TATTACAGGCATATCTGGAGATATTGCAAGTTCAGTTACAGAATACCTCAATAAAGTGAATATCACAATAAACAGAGTCACACAATGTTTTTTTTTGAGGTGAAAAAAAATCAATATGGAGTTTCCTAGTTGAAGGAAGAGACTGACTAA

58579880 58579900 58579920 58579940 58579960 58579980 58580000 58580020 58580040 58580060 58580080 58580100 58580120 58580140 58580160

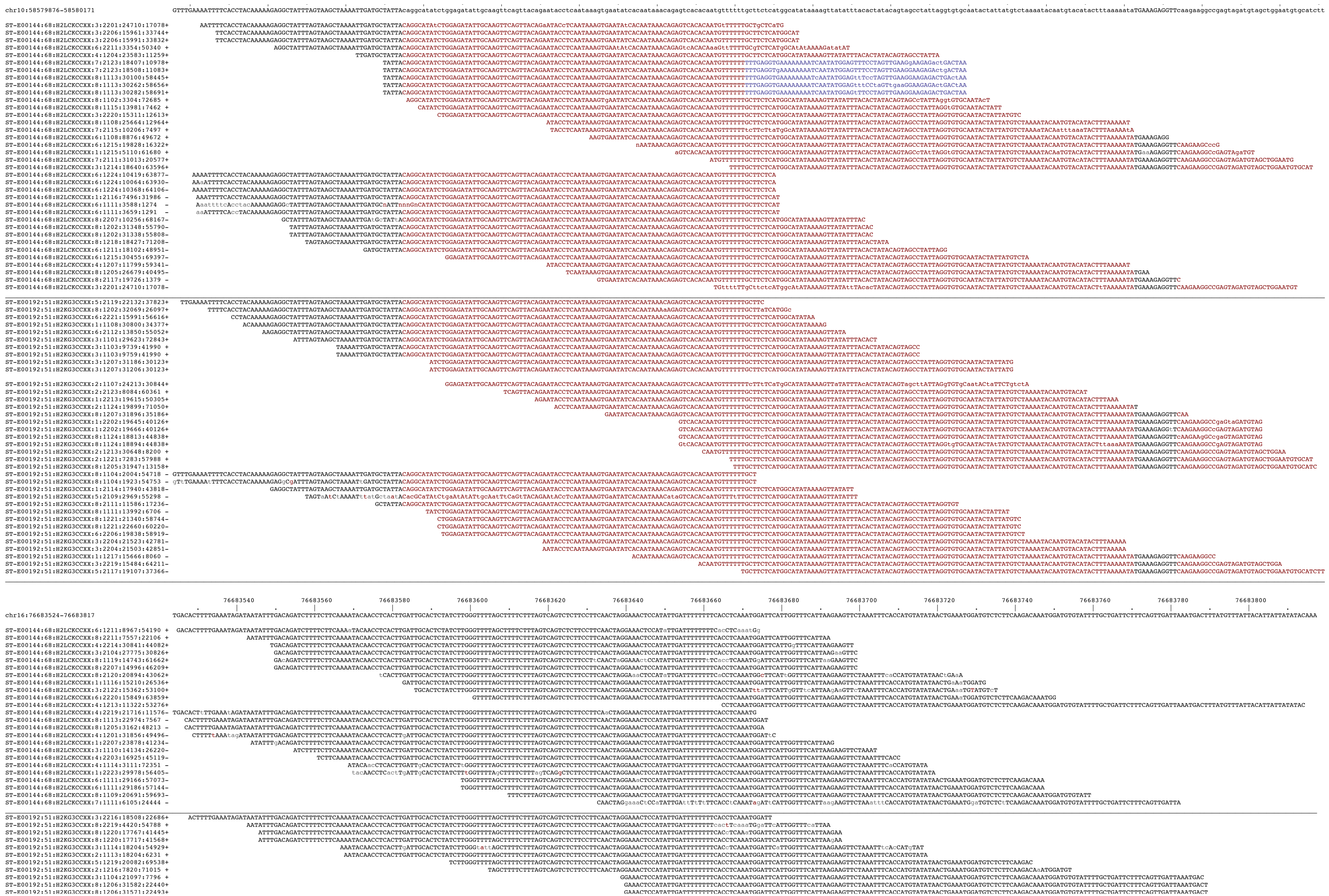
chr10:58579876-58580171
chr16:76683524-76683817

```
ST-E00192:51:H2KG3CCXX:1:2224:15078:16322+  ACTCCATATTGATTTTTTTTCACCTCAAATGGATTCATTGGTTTCATTAAGAAGTTCTAAATTTCACCATGTATATAACTGAAATGGATGTCTCTTCAAGACAAATGGATGTGTATTTTGCTGATTCTTTCAGTTGATTAAATGACTTTA
ST-E00192:51:H2KG3CCXX:3:1217:27116:14670+  TCCATATTGATTTTTTTTCACCTCAAaTGGATTCATTGGTTTCATTAAgAAGTTCTAAaTTtCACCATGTATATAACTGAAAtGGATGtctCTTCaAGACAAATGGATgtGTATTTTgCTGATTCTTTCtgTTGATTAAATGaCTtTATG

ST-E00192:51:H2KG3CCXX:2:2114:23056:69450+  ATTTTTTTCACCTCAAATGGATTCATTGGTTTCATTAAGAAGTTCTAAATTTCACCATGTATATAACTGAAATGGATGTCTCTTCAAGACAAATGGATGTGTATTTTGCtGATTCTTTCAGTTGATTAAATGACTTTatgTTTATTACA
ST-E00192:51:H2KG3CCXX:2:2114:23076:69450+  ATTTTTTTCACCTCAAATGGATTCATTGGTTTCATTAAGAAGTTCTAAATTTCACCATGTATATAACTGAAATGGATGTCTCTTCAAGACAAATGGATGTGTATTTTGCTGATTCTTTCAGTTGATTAAATGACTTTATGTTTATTACA
ST-E00192:51:H2KG3CCXX:2:2114:25563:70417+  ATTTTTTTCACCTCAAATGGATTCATTGGTTTCATTAAGAAGTTCTAAATTTCACCATGTATATAACTGAAATGGATGTCTCTTCAAGACAAATGGATGTGTATTTTGCTGATTCTTTCAGTTGATTAAATGACTTTATGTTTATTACA
ST-E00192:51:H2KG3CCXX:8:1220:15819:23477+  TTCACCTCAAATGGATTCATTGGTTTCATTAAGAAGTTCTAAATTTCACCATGTATATAACTGAAATGGATGTCTCTTCAAGACAAATGGATGTGTATTTTGCTGATTCTTTCAGTTGATTAAATGACTTTATGTTTATTACATTATTAT
ST-E00192:51:H2KG3CCXX:6:2213:11089:18555+  CACCTCAAATGGATTCATTGGTTTCATTAAGAAGTTCTAAATTTCACCATGTATATAACTGAAATGGATGTCTCTTCAAGACAAATGGATGTGTATTTTGCTGATTCTTTCAGTTGATTAAATGACTTTATGTTTATTACATTATTATAT
ST-E00192:51:H2KG3CCXX:2:1120:26121:25499+  CAAATGGATTCATTGGTTTCATTAAGAAGTTCTAAATTTCACCATGTATATAACTGAAATGGATGTCTCTTCAAGACAAATGGATGTGTATTTTGCTGATTCTTTCAGTTGATTAAATGACTTTATGTTTATTACATTATTATATATACAAA
ST-E00192:51:H2KG3CCXX:5:1204:19899:10046-  ACACTTTTGAAATAgATAATATttgacAGatCTTTTCTTcAAAaaACAACCtcacTTGATTGCACTCTATCTTGGgTTTTAGCTTTTCTTTagTcagcCtCTTCCTcCaacTaGGAAaCTCCatATggATtTTTTTCACCTCAAATGGA
ST-E00192:51:H2KG3CCXX:6:1110:30861:36768-  CACTTTTGAAATAGATAATATTTGACAGATCTTTTCTTCAAAATACAACCTCACTTGATTGCACTCTATCTTGGGTTTTAGCTTTTCTTTTAGTCAGTCTCTTCCTTCAACTAGGAAACTCCATATTGATTTTTTTCACCTCAAATGGAT
ST-E00192:51:H2KG3CCXX:5:1209:3304:41040 -  TTTGAAATAGATAATATTTGACAGATCTTTTCTTCAAAATACAACCTCACTTGATTGCACTCTATCTTGGGTTTTAGCTTTTCTTTTAGTCAGTCTCTTCCTTCAACTAGGAAACTCCATATTGATTTTTTTCACCTCAAATGGATTCA
ST-E00192:51:H2KG3CCXX:5:1209:3324:41040 -  TTTGAAATAGATAATATTTGACAGATCTTTTCTTCAAAATACAACCTCACTTGATTGCACTCTATCTTGGGTTTTAGCTTTTCTTTTAGTCAGTCTCTTCCTTCAACTAGGAAACTCCATATTGATTTTTTTCACCTCAAATGGATTCA
ST-E00192:51:H2KG3CCXX:5:1209:3334:41058 -  TTTGAAATAGATAATATTTGACAGATCTTTTCTTCAAAATACAACCTCACTTGATTGCACTCTATCTTGGGTTTTAGCTTTTCTTTTAGTCAGTCTCTTCCTTCAACTAGGAAACTCCATATTGATTTTTTTCACCTCAAATGGATTCA
ST-E00192:51:H2KG3CCXX:3:1223:8967:46314 -  CAAAATACAACCTCACTTgaTTGCACTCTATCTTGGGTTTTAGCTTTTCTTTTAGTCAGTCTCTTCCtTCAACTAGGAaaCTCCATATTGATTTTTTTCACCTCAAATGGATTCATTGGTTTCATTAAGAAGTTCTAAATTTCaCCATGT
ST-E00192:51:H2KG3CCXX:2:2123:27775:36100-  ctcccaACCtCacttgATTgctCTcTATcttgGCTTTTAGCTTTTCTTTTAGTCAGTcTCTTCCTTcAaCTAGGAAACTCCATATTGATTTTTTTCACCTCAAATGGATTCATTGGTTTCATTAAGAAGTTCTAAATTTCACCATGTATAT
ST-E00192:51:H2KG3CCXX:3:2212:21279:29455-  CAACCTCACTTGATTGCACTCTATCTTGGGTTTTAGCTTTTCTTTTAGTCAGTCTCTTCCTTCAACTAGGAAACTCCATATTGATTTTTTTCACCTCAAATGGATTCATTGGTTTCATTAAGAAGTTCTAAATTTCACCATGTATATAAC
ST-E00192:51:H2KG3CCXX:3:2212:21300:29455-  CAACCTCACTTGATTGCACTCTATCTTGGGTTTTAGCTTTTCTTTTAGTCAGTCTCTTCCTTCAACTAGGAAACTCCATATTGATTTTTTTCACCTCAAATGGATTCATTGGTTTCATTAAGAAGTTCTAAATTTCACCATGTATATAAC
ST-E00192:51:H2KG3CCXX:3:2212:21127:29472-  CAACCTCACTTGATTGCACTCTATCTTGGGTTTTAGCTTTTCTTTTAGTCAGTCTCTTCCTTCAACTAGGAAACTCCATATTGATTTTTTTCACCTCAAATGGATTCATTGGTTTCATTAAGAAGTTCTAAATTTCACCATGTATATAAC
ST-E00192:51:H2KG3CCXX:3:2212:21097:29490-  CAACCTCACTTGATTGCACTCTATCTTGGGTTTTAGCTTTTCTTTTAGTCAGTCTCTTCCTTCAACTAGGAAACTCCATATTGATTTTTTTCACCTCAAATGGATTCATTGGTTTCATTAAGAAGTTCTAAATTTCACCATGTATATAAC
ST-E00192:51:H2KG3CCXX:2:1120:11728:11119-  CTCTATCTTGGGTTTTAGCTTTTCTTTTAGTCAGTCTCTTCCTTCAACTAGGAAACTCCATATTGATTTTTTTCACCTCAAATGGATTCATTGGTTTCATTAAGAAGTTCTAAATTTCACCATGTATATAACTGAAATGGATGTCTCTgC
ST-E00192:51:H2KG3CCXX:8:2202:4268:33639 -  ggGTTTTAGCTTTTcTTTaGTCAGTCTCTTCCTTCAACTAGGaaaCTcCaTATTGATTTTTTTCtCCTCAAATGGATTCATTGGTTTCATTAAGAAGTTCTAAATTTCACCATGTATATAACTGAAATGGATGTCTCTTCAAGACAAAT
ST-E00192:51:H2KG3CCXX:6:1209:13230:54225-  TTTCTTTTAGTCAGTCTCTTCCTTCAACTAggAAACTCCATATTGATTTTTTTCACCTCAAATGGATTCATTGGTTTCATTAAGAAGTTCTAAATTTCACCATGTATATAACTGAAATGGATGTCTCTTCAAGACAAATGGATGTGTATT
ST-E00192:51:H2KG3CCXX:2:2214:17778:20542-  CTTCAACTAGgAAACTCCATATTGATTTTTTTCACCTCAAATGGATTCATTGGTTTCATTAAGAAGTTCTAAATTTCACCATGTATATAACTGAAATGGATGTCTCTTCAAGACAAATGGATGTGTATTTTGCTGATTCTTTCAGTTGA
ST-E00192:51:H2KG3CCXX:3:1203:18102:13088-  AGGAAACTCCATATTGATTTTTTTCACCTCAAATGGATTCATTGGTTTCATTAAGAAGTTCTAAATTTCACCATGTATATAACTGAAATGGATGTCTCTTCAAGACAAATGGATGTGTATTTTGCTGATTCTTTCAGTTGATTAAATGA
ST-E00192:51:H2KG3CCXX:3:1203:28811:60396-  AGGAaccTCCATATTGATTTTTTT-CACCTCaaAtGGATTCATTgGTTTCAttAAGAAGTtCTAAATTTCACCATGTATATAACTGAAATGGATGTCTCTTCAAGAcAAATGgaGGTGTATTTTGCTtGATTCTTTCAGTTGATTAAATgA
ST-E00192:51:H2KG3CCXX:8:2219:4420:54788 -  AAACTCCATATTGATTTTTTTCACCTCAAATGGATTCATTGGTTTCATTAAGAAGTTCTAAATTTCACCATGTATATAACTGAAATGGATGTCTCTTCAAGACAAATGGATGTGTATTTTGCTGATTCTTTCAGTTGATTAAATGACT
ST-E00192:51:H2KG3CCXX:1:2124:12469:34166-  CTCCATATTGATTTTTcTCACCTCAAATGGATTCATtGGTTTCATTAAGAAGTTCtAAATTTCACCATGTATATAACTGAAATGGATGTCTCTTCAAGACAAATGGATGTGTATTTTGCTGATTCTTTCAGTTGAtTAAATGACTTTAT
ST-E00192:51:H2KG3CCXX:1:2124:12977:34412-  CTCCATATTGATTTTTgTCACCTCAAATGGATTCATgGGTTTCATTAAGAAGTTCGAAATTTCACCATGTATATAACTGAAATGGATGTCTCTTCAAGACAAATGGATGTGTATTTTGCTGATTCTTTCAGTTGAGTAAATGACTTTAT
```

---

chr10 72344569 + 5 chr10 106230197 + 0 DEL 52 17 43 0 0.985714285714286 0 1 0 1 chr10 72344437 150 chr10 106230213

AGTGGAAACACTAACAAAGGAGGGTGCGTTCCTCCTCCTGCTTTCTGAGGATGCCCTACTCTGTAACAAAGGAGTTTCCAATGCATTTACTTCTTTCATTGTTCTCTGTGACCTGCTCCCAGTTCTTTCCTACTCAGATAGTAAGGAATT

```
                       72344440            72344460            72344480            72344500            72344520            72344540            72344560            72344580            72344600            72344620            72344640            72344660            72344680            72344700
chr10:72344432-72344717  TtataagtggaaacactaacaaaggagggtgcgttcctcctcctgctttctgaggatgccctactctgtaacaaaggagtttccaatgcatttacttctttcattgttctctgtgacctgctcccagttctttcctacacaagatccaggaactgtccctggggtctggatccgtacccctctttttctggcaacaGTATTATTTTCAAATACACTACAGAAAATAAGCAAGAATGAGCTACTGCATCACCCCATGGGGGTCCCTGCAGTACCAGCTTCCAGGAG

ST-E00144:68:H2LCKCCXX:8:1208:2451:26589 +  AAGTGGAAACACTAACAAAGGAGGGTGCGTTCCTCCTCCTGCTTTCTGAGGATGCCCTACTCTGTAACAAAGGAGGTTCCAATGCATTTACTTCTTTCATTcTTCTCtGTGACCtgcTCCCAGttcTtTCCTctaCAAGATCCGgGAacg
ST-E00144:68:H2LCKCCXX:8:2121:17828:41902+  AAGTGGAAACACTAACAAAGGAGGGTGCGTTCCTCCTCCTGCTTTCTGAGGATGCCCTACTCTGTAACAAAGGAGTTTCCAATGCATTTACTTCTTTCATTGTTCTCTGTGACCTGCTCCCAGTTCTTTCCTACACAAGATCCGGGAACT
ST-E00144:68:H2LCKCCXX:8:2121:17098:42570+  AAGTGGAAACACTAACAAAGGAGGGTGCGTTCCTCCTCCTGCTTTCTGAGGATGCCCTACTCTGTAACAAAGGAGTTTCCAATGCATTTACTTCTTTCATTGTTCTCTGTGACCTGCTCCCAGTTCTTTCCTACACAAGATCCGGGAACT
ST-E00144:68:H2LCKCCXX:3:2219:9008:50885 +  AGTGGAAACACTAACAAAGGAGGGTGCGTTCCTCCTCCTGCTTTCTGAGGATGCCCTACTCTGTAACAAAGGAGTTTCCAATGCATTTACTTCTTTCATTGTTCTCTGTGACCTGCTCCCAGTTCTTTCCTACACAAGATcCGGGAACtG
ST-E00144:68:H2LCKCCXX:4:2214:10053:44468+  AGTGGAaACACTAACAAaGGagGGTgCGTTCCTCCTCCTGCTTTCTGAGGATGCCCTACTCTGTAACAaAgGAgTtTCCAaTGCATTTACTTCTTTCATTgTtcTCTGTGaCCTGCTcCcagttCTTTCCTACTCAgatcgtaaggaaTT
ST-E00144:68:H2LCKCCXX:4:2214:9901:44697 +  AGTGGAAACACTAACAAAGGAGGGTGCGTTCCTCCTCCTGCTTTCTGAGGATGCCCTACTCTGTAACAAAGGAGTTTCCAATGCATTTACTTCTTTCATTGTTCTCTGTGACCTGCTCCCAGTTCTTTCCTACTCAGaTAGTAAGGAatTT
ST-E00144:68:H2LCKCCXX:4:2214:10033:44785+  AGTGGAAACACTAACAAAGGAGGGTGCGTTCCTCCTCCTGCTTTCTGAGGATGCCCTACTCTGTAACAAAGGAGTTTCCAATGCATTTACTTCTTTCATTGTTCTCTGTGACCTGCTCCCAGTTCTTTCCTACTCAGAtAGTAAGGAATT
ST-E00144:68:H2LCKCCXX:8:2121:31571:8499 +  GGAAACACTAACAAAGGAGGGTGCGTTCCTCCTCCTGCTTTCTGAGGATGCCCTACTCTGTAACAAAGGAGTTTCCAATGCATTTACTTCTTTCATTGTTCTCTGTGACCTGCTCCCAGTTCTTTCCTACACAAGATCCAGGAACTGTCC
ST-E00144:68:H2LCKCCXX:6:1103:23421:54015+  AACACTAcCaAAGGAGGGTGCGTTCCTCCTcCTGCTTTCTGAGGATGCCcTACtCTGTAACAAAGGAGTTTCCAATGCATTTACTTCTTTCATTGTTCTCTGTGcCCTGCTCCcaGTTctTTCCTAcacAAGATCaAtGAACTGTaCcTG
ST-E00144:68:H2LCKCCXX:6:1108:28780:1695 +  ACACTAACAAAGGAGGGTGCGTTCCTCCTCCTGCTTTCTGAGGATGCCCTACTCTGTAACAAAGGAGTTTCCAATGCATTTACTTCTTTCATTGTTCTCTGTGACCTGCTCCCAGTTCTTTCCTACACAAGATCCGGGAACTGTCCCTGG
ST-E00144:68:H2LCKCCXX:3:1106:17402:68272+  CTAACAAAGGAGGGTGCGTTCCTCCTCCTGCTTTCTGAGGATGCCCTACTCTGTAACAAAGGAGTTTCCAATGCATTTACTTCTTTCATTGTTCTCTGTGACCTGCTCCCAGTTCTTTCCTACACAAGATCCGGGAACTGTCCCTGGGGG
ST-E00144:68:H2LCKCCXX:7:1111:9008:71314 +  TAACAAAGGAGGGTGCGTTCCTCCTCCTGCTTTCTGAGGATGCCCTACTCTGTAACAAAGGAGTTTCCAATGCATTTACTTCTTTCATTGTTCTCTGTGACCTGCTCCCAGTTCTTTCCTACACAAGATCCAGGAACTGTCCCTGGGGGT
ST-E00144:68:H2LCKCCXX:4:1209:14286:22616+  ACAAAGGAGGGTGCGTTCCTCCTCCTGCTTTCTGAGGATGCCCTACTCTGTAACAAAGGAGTTTCCAATGCATTTACTTCTTTCATTGTTCTCTGTGACCTGCTCCCAGTTCTTTCCTACACAAGATCCGGGAACTGTCCCTGGGGGTCT
ST-E00144:68:H2LCKCCXX:7:1102:4684:22194 +  CAAAGGAGGGTGCGTTCCTCCTCCTGCTTTCTGAGGATGCCCTACTCTGTAACAAAGGAGtTTCCAATGCATTTACTTCTTTCATTGTTCTCTGTGACCTGCTCCCAGTTCTTTCCTACACAAGAtCCGGGAACTGTCCCTGGGGGTCT
ST-E00144:68:H2LCKCCXX:8:1212:20366:18660+  AAAGGAGGGTGCGTTCCTCCTCCTGCTTTCTGAGGATGCCCTACTCTGTAACAAAGGAGTTTCCAATGCATTTACTTCTTTCATTGTTCTCTGTGACCTGCTCCCcGTTCTTTCCTACACAAGATCCGGGAaCTGTCCCTGGGGGTCTGG
ST-E00144:68:H2LCKCCXX:7:1118:32414:16533+  GAGGGTGCGTTCCTCCTCCTGCTTTCTGAGGATGCCCTACTCTGTAACAAAGGAGTTTCCAATGCATTTACTTCTTTCATTGTTCTCTGTGACCTGCTCCCAGTTCTTTCCTACACAAGATCCGGgAACtGTCCCTGGGGGcctGGATCC
ST-E00144:68:H2LCKCCXX:6:2108:14753:60027+  CCTCCTGCTTTCTGAGGATGCCCTACTCTGTAACAAAGGAGTTTCCAATGCATTTACTTCTTTCATTGTTCTCTGTGACCTGCTCCCAGTTCTTTCCTACACAAGATCCGGGAACTGTCCCTGGGGGTCTGGATCCGTACCCCTCTTTTT
ST-E00144:68:H2LCKCCXX:6:2216:18163:58093+  CCTCCTGCTTTCTGAGGATGCCCTACTCTGTAACAAAGgAGTTTCCAATGCATTTACTTCTTTCATTGTTCTCTGTGACCTGCTCCCAGTTCTtTCCTACACAAGATCCGGGAACTGTCCCTGGGGtCTGGAtCcGTACCCCTCTTTTT
ST-E00144:68:H2LCKCCXX:6:1103:7435:70030 +  TTTCTGAGGATGCCCTACTCTGTAACAAAGGAGTTTCCAATGCATTTACTTCTTTCATTGTTCTCTGTGACCTGCTCCCAGTTCTTTCCTACACAAGaTCCAGGAACTGTCCCTGGGGGTCTGGATCCGTACCCCTCTTTTTCTGgCaaC
ST-E00144:68:H2LCKCCXX:6:1114:15291:10749+  GAGGATGCCCTACTCTGTAACAAAGGAGTTTCCAATGCATTTACTTCTTTCATTGTTCTCTGTGACCTGCTCCCAGTTCTTTCCTACACAAGATCCAGGAACTGTCCCTGgGGGtCTGGATcCGTACCCCTCTTTTTCTGGCAACAGTAT
ST-E00144:68:H2LCKCCXX:3:2105:6054:59060 +  AGGATGCCCTACTCTGTAACAAAGGAGTTTCCAATGCATTTACTTCTTTCATTGTTCTCTGTGACCTGCTCCCAGTTCTTTCCTACACAAGATCCGGGAACTGTCCCTGGGGGTCTGGATCCGTACCCCTCTTTTTCTGGcaaCAGTAtT
ST-E00144:68:H2LCKCCXX:1:2210:14702:34061+  GGATGCCCTACTCTGTAACAAAGGAGTTTCCAATGCATTTACTTCTTTCATTGTTCTCTGTGACCTGCTCCCAGTTCTTTCCTACACAAGATCCAGGAACTGTCCCTGGGGGTCTGGAgccGTaCcCCTCTTTTTCTGGcAACAGTATTA
ST-E00144:68:H2LCKCCXX:8:1217:10622:11382+  TGTAACAAAGGAGTTTCCAATGCATTTACTTCTTTCATTGTTCTCTGTGACCTGCTCCCAGTTCTTTCCTACACAAGATCCGGGAACTGTCCCTGGGGGTCTGGATCCGTACCCCTCTTTTTCTGGCAACAGTATTATTTTCAAATACAC
ST-E00144:68:H2LCKCCXX:6:1205:12926:49619+  ACAAAgGcGTTTCCAATGCATTTACTTCTTTcATTGTTCTCTGTGACCTGCTCCcAGTTCTTTcCTaCACAaGatCCGGGAACTGTCCcTGGGggtCTGGcgcCGTAcCCcTCTttTTCTGGCaaCcgtattATgtTcaaATaCACtaCa
ST-E00144:68:H2LCKCCXX:7:2121:29876:19276+  ACAAAGGAGTTTCCAATGCATTTACTTCTTTCATTGTTCTCTGTGACCTGCTCCCAGTTCTTTCCTACACAAGATCCGGGAACTGTCCCTGGGGGTCTGGATCCGTACCCCTCTTTTTCTGGCAACAGTATTATTTTCAAaTacACtACA
ST-E00144:68:H2LCKCCXX:7:2121:30536:27451+  ACAAAGGAGTTTCCAATGCATTTACTTCTTTCATTGTTCTCTGTGACCTGCTCCCAGTTCTTTCCTACACAAGATCCGGGAACTGTCCCTGGGGGTCTGGATCCGTACCCCTCtCTTTTCTGGCAACAGTATTATTTTCAAATACACTAC
ST-E00144:68:H2LCKCCXX:1:2109:2847:57091 +  CAAaGGgGttTCCAAtGCATTTACTtcTTTcatTgTTCTCTGTGacGTGCTCCcAGTTCTttCcTACACAAGatccAggAactaTCcCTGaGGTTCTGtatCCGagaccatcttttTcTGtcaagagtattcTTTTaacacAcactACAG
ST-E00144:68:H2LCKCCXX:8:2109:9911:28119 +  aAGGAGTTTCCAATGCATTTACTTCTTTCATTGTTCTCTGTGACCTGCTCCCAGTTCTTTCCTACACAAGATCCGGGAACTGtcCCTGGGGgtCTGGATcCGTACCCCTCTTTTTCTGGCaaCAgTATTATTTTcAaAT
ST-E00144:68:H2LCKCCXX:8:2111:17240:11453+  AAGGAGTTTCCAATGCATTTACTTCTTTCATTGTTCTCTGTGACCTGCTCCCAGTTCTTTCCTACACAAGATCCGGGAACTGTCCCTGGGGGTCTGGATCCGTACCCCTCTTTTTCTGGCAACAGTATTATTTTCAAATACaCTACAGaA
ST-E00144:68:H2LCKCCXX:8:2111:17169:11470+  AAGGAGTTTCCAATGCATTTACTTCTTTCATTGTTCTCTGTGACCTGCTCCCAGTTCTTTCCTACACAAGATCCGGGAACTGTCCCTGGGGGTCTGGATCCGTACCCCTCTTTTTCTGGCAACAGTATTATTTTCAAATACACTACAGAA
ST-E00144:68:H2LCKCCXX:3:1204:17108:58621+  CAATGCATTTACTTCTTTCATTGTTCTCTGTGACCTGCTCCCAGTTCTTTCCTACACAAGATCCAGGAACTGTCCCTGGGGGTCTGGATCCGTACCCCTCTTTTTCTGGCAACAGTATTATTTTCAAATACACTACaAGAAAATAAGCAAG
ST-E00144:68:H2LCKCCXX:4:1124:26030:15461+  CAATGCATTTACTTCTTTCATTGTTCTCTGTGACCTGCTCCCAGTTCTTTCCTACACAAGATCCAGGAACTGTCCCTGGGGGTCTGGATCCGTACCCCTCTTTTTCTGGCAACAGTaTATTTTCAaATACACTACAGAAAATaAGCAAG
ST-E00144:68:H2LCKCCXX:1:2216:14651:22827+  CTTCATTGTTCTCTGTGACCTGCTCCCAGTTCTTTCCTACACAAGATCCAGGAACTGTCCCTGGgGGTCTGGATCCGTACCCCTCTTTTTCTGGCAACAGTATTATTTTCAAATaCACTCcAGAAAATAAGCAGaATGAGCTACT

ST-E00144:68:H2LCKCCXX:7:2217:29410:22300+  TTTCATTGTTCTCTGTGACCTGCTCCCAGTTCTTTCCTACACAAGATCCAGGAACTGTCCCTGGGGGTCTGGATCCGTACCCCTCTTTTTCTGGCAACAGTATTATTTTCAcatAcaCTACAGaaATAAGcaAgAAtGAGCtactgcat
ST-E00144:68:H2LCKCCXX:6:2106:22041:47967+  GTGACCTGCTCCCAGTTCTTTCCTACACAAGATCCGGGAACTGTCCCTGGGGGTCTGGaTCCGTACCCCTCTTTTTCTGGCAACAGTATTATTTTCAAATACACTACAGAAAATAAGCAAGAATGAGCTACtGCATCACCCCATGgGGG
ST-E00144:68:H2LCKCCXX:3:1114:13271:24198+  GCTCCCAGTTCTTTCCTACACAAGATCCGGGAACTGTCCCTGGGGGTCTGGATCCGTACCCCTCTTTTTCTGGCAACAGTATTATTTTCAaATACACTACAGAAAATAAGCAAGAATGAGCTACTGCATCACCCCAtggGGGTCCCTGCA
ST-E00144:68:H2LCKCCXX:8:1106:23523:66180-  GGAAACACTAACAAAGGAGGGTGCGTTCCTCCTCCTGCTTTCTGAGGATGCCCTACTCTGTAACAAAGGAGTTTCCAATGCATTTACTTCTTTCATTGTTCTCTGTGACCTGCTCCCAGTTCTTTCCTACACAAGATCCAGGAACTGTCC
ST-E00144:68:H2LCKCCXX:3:1217:16326:40425-  TAACAAAGGAGGGTGCGTTCCTCCTCCTGCTTTCTGAGGATGCCCTACTCTGTAACAAAGGAGTTTCCAATGCATTTACTTCTTTCATTGTTCTCTGTGACCTGCTCCCAGTTCTTTCCTACACAAGATCCGGGAACTGTCCCTGGGGGT
ST-E00144:68:H2LCKCCXX:7:1212:8094:40653 -  CAaaGGAGGGTGCGTTCCTCCTCCTGCTTTCTGAGGATGCCCTACTCTGTAACAAAGGAGTTTCCAATGCATTTACTTCTTTCtTCATTGTTCTCTGTGACCTGCTCCCAGTTCTTTCCTACACAAGATCCGGGAACTGTCCCTGGGGGTCTG
ST-E00144:68:H2LCKCCXX:7:1212:7973:40689 -  CAAAGGAGGGTGCGTTcCTCCTCCTGCTTTCTGAGGATGCCCTACTCTGTAACAAAGGAGTTTCCAATGCATTTACTTCTTTCATTGTTCTCTGTGACCTGCTCCCAGTTCTTTCCTACACAAGATCCGGGAACTGTCCCTGGGGGTCTG
ST-E00144:68:H2LCKCCXX:8:1215:2380:45804 -  aggacggtGCGttgCtcctcCtGCttTCttaGgaTGCCCTACTCTGtAACaaaGgaGTTTCCAATGCaTTTACTTCTTTCatTGTTCTCTGTGaCCTGCTCCCAGTTCTTTCCTACACAAGATCCAggAgCTGTCCCTGGGGGTCTGgAT
ST-E00144:68:H2LCKCCXX:7:2209:30628:59746-  GAGGGTGCGTTCCTCCTCtgcTTTCTGAGGATGCCCTACTCTGTAACAAAGGAGTTTCCAATGCATTTACTTCTTTCATTGTTCTCTGTGACCTGCTCCCAGTTCTTTCCTACACAAGATCCGGGAACTGTCCCTGGGGGTCTGGATCC
ST-E00144:68:H2LCKCCXX:8:2106:14621:50586-  GAGGGTGCGTTCCTCCTCCTGCTTTCTGAGGATGCCCTACTCTGTAACAAAGGAGTTTCCAATGCATTTACTTCTTTCATTGTTCTCTGTGACCTGCTCCCAGTTCTTTCCTACACAAGATCCGGGAACTGTCCCTGGGGGTCTGGATCC
ST-E00144:68:H2LCKCCXX:4:2214:10053:44468-  CGTTCCTCCTCCTGCTTTCTGAgGATGCCCTACTCTGTAACAAAGGAGTTTCCAATGCATTTACTTCTTTCATTGTTCTCTGTGACCTGCTCCCAGTTCTTTCCTACTCAGATAGTAAGGAATTTTGCCTGCATTTTTCTGTAGAAGTTT
ST-E00144:68:H2LCKCCXX:4:2214:9901:44697 -  CGTTCCTCCTCCTGCTTTCTGAGGATGCCCTACTCTGTAACAAAGGAGTTTCCAATGCATTTACTTCTTTCATTGTTCTCTGTGACCTGCTCCCAGTTCTTTCCTACTCAGATAGTAAGGAATTTTGCCTGCATTTTTCTGTAGAAGTTT
ST-E00144:68:H2LCKCCXX:4:2214:10033:44785-  CGTTCCTCCTCCTGCTTTCTGAGGATGCCCTACTCTGTAACAAAGGAGTTTCCAATGCATTTACTTCTTTCATTGTTCTCTGTGACCTGCTCCCAGTTCTTTCCTACTCAGATAGTAAGGAATTTTGCCTGCATTTTTCTGTAGAAGTTT
ST-E00144:68:H2LCKCCXX:4:1107:11637:26923-  GTTCCTCCTCCTGCTTTCTGAGGATGCCCTACTCTGTAACAAAGGAGTTTCCAATGCATTTACTTCTTTCATTGTTCTCTGTGACCTGCTCCCAGTTCTTTCCTACACAAGATCCGGGAACTGTCCCTGGGGGTCTGGATCCGTACCCCT
ST-E00144:68:H2LCKCCXX:3:2110:26710:4122 -  CCTCCTCCTGCTTTCTGAGGATGCCCTACTCTGTAACAAAGGAGTTTCCAATGCATTTACTTCTTTCATTGTTCTCTGTGACCTGCTCCCAGTTCTTTCCTACACAAGATCCAGGAACTGTCCCTGGGGGTCTGGATCCGTACCCCTCTT
ST-E00144:68:H2LCKCCXX:3:2207:10490:25570-  TCCTGCTTTCTGAGGATGCCCTACTCTGTAACAAAGGAGTTTCCAATGCATTTACTTCTTTCATTGTTCTCTGTGACCTGCTCCCAGTTCTTTCCTACACAAGATCCAGGAACTGTCCCTGGGGGTCTGGATCCGTACCCCTCTTTTTCT
ST-E00144:68:H2LCKCCXX:4:2116:22132:29982-  CtTTCTGAGGATGCCCTACTCTGTAACAAAGGAGTTTCCAATGCATTTACTTCTTTCATTGTTCTCTGTGACCTGCTCCCAGTTCTTTCCTACACAAGATCCGGGAACTGTCCCTGGGGGTCTGGATCCGTACCCCTCTTTTTCTGGCAA
ST-E00144:68:H2LCKCCXX:1:1203:26131:50059-  TCTGAGGATGCCCTACTCTGTAACAAAGGAGTTTCCAATGCATTTACTTCTTTCATTGTTCTCTGTGACCTGCTCCCAGTTCTTTCCTACACAAGATCCGGGAACTGTCCCTGGGGGTCTGGATCCGTACCCCTCTTTTTCTGGCAACAG
ST-E00144:68:H2LCKCCXX:8:2119:29755:12771-  TGCCCTACTCTGTAACAAAGGAGTTTCCAATGCATTTACTTCTTTCATTGTTCTCTGTGACCTGCTCCCAGTTCTTTCCTACACAAGATCCGGGAACTGTCCCTGGGGGTCTGGATCCGTACCCCTCTTTTTCTGGCAACAGTATTATTT
ST-E00144:68:H2LCKCCXX:8:2119:29724:12789-  TGCCCTACTCTGTAACAAAGGAGTTTCCAATGCATTTACTTCTTTCATTGTTCTCTGTGACCTGCTCCCAGTTCTTTCCTACACAAGATCCGGGAACTGTCCCTGGGGGTCTGGATCCGTACCCCTCTTTTTCTGGCAACAGTATTATTT
ST-E00144:68:H2LCKCCXX:4:1120:4116:41251 -  CCTACtCTGTAACAAAGGagTTTCCAATGCATTTACTTCTTTCaTTGTTCTCTGTGACCTGCTCCCAGTTCTTTCCTACACAAGATCCAGGAACTGTCCCTGGGGGTCTGGATCCGTACCCCTCTTTTTCTGGCAACAGTATTATTTTCA
ST-E00144:68:H2LCKCCXX:1:2121:29227:18748-  CTGTAACAAAGGAGTTTCCAATGCATTTACTTCTTTCATTGTTCTCTGTGACCTGCTCCCAGTTCTTTCCTACACAAGATCCGGGAACTGTCCCTGGGGGTCTGGATCCGTACCCCTCTTTTTCTGGCAACAGTATTATTTTCAAATACA
ST-E00144:68:H2LCKCCXX:1:2121:29227:18854-  CTGTAACAAAGGAGTTTCCAATGCATTTACTTCTTTCATTGTTCTCTGTGACCTGCTCCCAGTTCTTTCCTACACAAGATCCGGGAACTGTCCCTGGGGGTCTGGATCCGTACCCCTCTTTTTCTGGCAACAGTATTATTTTCAAATACA
ST-E00144:68:H2LCKCCXX:8:1202:3801:49250 -  TcACaaaGGAGTTTCCAacgcaTTTaCTTCTTtCattGTTCtCTGTGACCTgCCCCAGTTCTTTCCTACaCAAGAtCCGGGAACTGTCCCTGGGGGTCTGGATCCGTACCCCTCTTTTTCTGGCAACAGTATTATTTTCAAATACACTA
ST-E00144:68:H2LCKCCXX:8:1101:25096:24954-  GCATTTACTTCtTTCATTGTTCTCTGTGACCTGCTCCCAgTTCTTTCCTACACAAGATCCGGGAACTGTCCCTGGGGGTCTGGATCCGTACCCCTCTTTTTCTGGCAACAGTATTATTTTCAAATACACTACAGAAAATAAGCAAGAATG
ST-E00144:68:H2LCKCCXX:4:1110:22457:62365-  CATTTACTTCTTTCATTGTTCTCTGTGACctgctCCCAGTTCTTTCCTACACAAGATCCGGGAACTGTCCCTGGGGGTCTGGATCCGTACCCCTCTTTTTCTGGCAACAGTATTATTTTCAAATACACTACAGAAAATAAGCAAGAATGA
ST-E00144:68:H2LCKCCXX:1:1112:28181:9519 -  TCTttCATTGTTCTCTGTGaCCTGCTCCCAGTTCTTTCCTACACAAGATCCGGGAACTGTCCCTGGGGGTCTGGATCCGTACCCCTCTTTTTCTGGCAACAGTATTATTTTCAAATACACTACAGAAAATAAGCAAGAATGAGCTACTGC
ST-E00144:68:H2LCKCCXX:1:2205:7343:2082  -  aCCTGCTCCCAGTTCTTTCCTACACAAGATCCGGGAACTGTCCCTGGGGGTCTGGATCCGTACCCCTCTTTTTCTGGCAACAGTATTATTTTCAAATACACTACAGAAAATAAGCAAGAATGAGCTACTGCATCACCCCATGGGGGTCCC
ST-E00144:68:H2LCKCCXX:8:1121:4775:37507 -  TGCTCCCAGTTCTTTCCTACACAAGATCCGGGAACTGTCCCTGGGGGTCTGGATCCGTACCCCTCTTTTTCTGGCAACAGTATTATTTTCAAATACACTACAGAAAATAAGCAAGAATGAGCTACTGCATCACCCCATGGGGGTCCCTGC
ST-E00144:68:H2LCKCCXX:8:1121:5039:36381 -  CTCCCAGTTCTTTCCTACACAAGATCCGGGAACTGtCCCTGGGGGTCTGGATCCGTACCCCTCTTTTTCTGGCAACAGTATTATTTTCAAATACACTACAGAAAATAAGCAAGAATGAGCTACTGCATCACCCCATGGGGGTCCCTGC
ST-E00144:68:H2LCKCCXX:1:1114:15260:45928-  CAGTTCTTTCCTACACAaGATCCGGGAACTGTCCCTGGGGGTCTGGATCCGTACCCCTCTTTTTCTGGCAACAGTATTATTTTCAAATACACTACAGAAAATAAGCAAGAATGAGCTACTGCATCACCCCATGGGGGTCCCTGCAGTACC
ST-E00144:68:H2LCKCCXX:8:1208:2451:26589 -  TTCTTtcCTACACAagaTCCGGGAACtGTCcctgGGGGTCTGGATCCGTACCCCTCTTTTTCTGGCAACAGTATTATTTTCAAATACACTACAGAAAATAAGCAAGAATGAGCTACTGCATCACCCCATGGGGGTCCCTGCAGTACCAG

ST-E00192:51:H2KG3CCXX:1:2202:29755:44099+  ATAAGTGGAAACACTAACAAAGGAGGGTGCGTTCCTCCTCCTGCTTTCTGAGGATGCCCTACTcTGTAACAAAGGAGTTTCCAATGCATTTACTTcTTTCATTGTTCTCTGTGACCTGCTCCcAGTTCTTTCctAcAcAAGAtccggGAA
ST-E00192:51:H2KG3CCXX:3:1208:4775:30263 +  AACACTAACAAAGGAGGGTGCGTTCCTCCTCCTGCTTTCTGAGGATGCCCTACTCTGTAACAAAGGAGTTTCCAATGCATTTACTTCTTTCATTGTTCTCTGTGACCTGCTCCCAGTTCTTTCCTACACAAGATCCGGGAACTGTCcCTG
ST-E00192:51:H2KG3CCXX:3:1212:15220:12595+  ACACTAACAAAGGAGGGTGCGTTCCTCCTCCTGCTTTCTGAGGATGCCCTACTCTGTAACAAAGGAGTTTCCAATGCATTTACTTCTTTCATTGTTCTCTGTGACCTGCTCCCAGTTCTTTCCTACACAAGCTCCaGGaACTGTCCCTGG
ST-E00192:51:H2KG3CCXX:1:2114:8358:23108 +  AAGGAGGGTGCGTTCCTCCTCCTGCTTTCTGAGGATGCCCTACTCTGTAACAAAGGAGTTTCCAATGCATTTACTTCTTTCATTGTTCTCTGTGACCTGCTCCCAGTTCTTTCCTACACAAGATCCGGGAACTGTCCCTGGGGGTCTGGA
ST-E00192:51:H2KG3CCXX:2:1124:7029:25446 +  CGTTCCTCCTCCTGCTTTCTGAGGATGCCCTACTCTGTAACAAAGGAGTTTCCAATGCATTTACTTCTTTCATTGTTCTCTGTGACCTGCTCCCAGTTCTTTCCTACACAAGATCCGGGAACTGTCCCTGGGGGTCTGGaTCCGTACCCC
ST-E00192:51:H2KG3CCXX:8:2104:1954:23653 +  CCTCCTCCTGCTTTCTGAGGATGCCCTACTCTGTAACAAAGGAGtTTCtaaTgCATTtACTTCTTTCATTGTTCTCTGTGACCTGatCcaAGtTCtTTCCTACACAAGATCCAGGAACTGTCCCTGGGGGTCTGGATCCGTACCCCTCTT
ST-E00192:51:H2KG3CCXX:5:1113:24893:61627+  CCTCCTGCTTTCTGAGGATGCCCTACTCTGTAACAAAGGAGTTTCCAATGCATTTACTTCTTTCATTGTTCTCTGTGACCTGCTCCCAGTTCTTTCCTACACAAGATCCGGGAACTGTCCCTGGGGGTCTGGATCCGTACCCCTCTTTTT
ST-E00192:51:H2KG3CCXX:1:1116:27501:40970+  ATGCCCTACTCTGTAACAAAGGAGTTTCCAATGCATTTACTTCTTTCATTGTTCTCTGTGACCTGCTCCCAGTTCTTTCCTACACAAGATCCGGGAACTGTCCCTGGGGGTCTGGATCCGTACCCCTCTTTTTCTGGCAACAGTATTATT
ST-E00192:51:H2KG3CCXX:5:1120:19990:56476+  ATGCCCTACTCTGTAACAAAGGAGTTTCCaATGCaTttACTTCTTTcATTGTTCTCTGTGACCTGCTCCCAGTTCTTTccTACACAAGATCCagGAACTGTCCCTGGGGgTCTGGATCCgTACCCCTcTTTTtCTgGCAACAGTATTATT
ST-E00192:51:H2KG3CCXX:3:2218:29338:22880+  CCTACTCTGTAACAAAGGAGTTTCCAATGCATTTACTTCTTTCATTGTTCTCTGTGACCTGCTCCCAGTTCTTTCCTACACAAGATCCAGGAACTGTCCCTGGGGGTCTGGATCCGTACCCCTCTTTTTCTGGCAACAGTATTATTTTCA
ST-E00192:51:H2KG3CCXX:3:1117:18782:46367+  ACTCtGTAACAAAGGAGTTTCCAATGCATTtACTTCTTTCATTGTTCTCTGTGACCTGCTCCCAGTTCTTTCCTACACAAGATCCGGGAACTGTCCCTGGGGGTCTGGATCCgtaccCCTCTTTTTCTGgcaacagtattaTTTTaaaT
ST-E00192:51:H2KG3CCXX:1:1221:29359:43378+  TaCTtCttTcATTGTTCTCTgTGACCTGCTCCAGTTCTTTCCTaCACAAgAtCcAggaacTgtCCtTGtgGtCtGgaTCCttaCCCCcCTtTtTCTGGCAACaGTATTATTTTCAAtActcTACcGAAaataAgcaaGaaTgagcTA
```

```
ST-E00192:51:H2KG3CCXX:3:2121:23137:5809 +   TTCTTTCATTGTTCTCTGTGACCTGCTCCCAGTTCTTTCCTACACAAGATCCGGGaACTGTCCCTGGGGGTCTGGATCCGTACCCCTCTTTTTCTGGCAACAGTATTATTTTCAAATACACTACAGAaAATAAGCAAGAaTGAGCTACTG
ST-E00192:51:H2KG3CCXX:6:1119:25319:47316+   GTTCTCTGTGACCTGCTCCCAGTTCTTTCCTACACAAGATCCGGGAACTGTCCCTGGGGGTCTGGATCCGTACCCCTCTTTTTCTGGCAACAGTATTATTTTCAAATACACTACAGAAAATAAGCAAGAATGAGCTACTGCATCACCcCA
ST-E00192:51:H2KG3CCXX:6:1201:20193:67516+   GTTCTCTGTGACCTGCTCCCAGTTCTTTCCTACACAAGATCCAGGAACTGTCCCTGGGGGTCTGGATCCGTACCCCTCTTTTTCTGGCAACAGTATTATTTTCAaATACACtaCAGAAAATAAGCAAGAATGAGCTACTGCATCACCCCA
ST-E00192:51:H2KG3CCXX:6:2220:25695:17764+   GTTCTCTGTGACCTGCTCCCAGTTCTTTCCTACACAAGATCCAGGAACTGTCCCTGGGGGTCTGGATCCGTACCCCTCTTTTTCTGGCAACAGTATTATTTTCAAATACACTACaGAAAATAAGCAAGAATGAGCTACTGCATCACCCcA
ST-E00192:51:H2KG3CCXX:8:2210:32739:59535+   CTGTGACCTGCTCCCAGTTCTTTCCTACACAAGATCCAGGAACTGTCCCTGGGGGTCTGGATCCGTACCCCTCTTTTTCTGGCAACAGTATTATTTTCAAATACACTACAGAAAATAAGCAAGAATGAGCTGCTGCATCACCCCATGGGG
ST-E00192:51:H2KG3CCXX:3:1118:2979:58585 +   GACCTGCTCCCAGTTCTTTCCTACACAAGATCCGGGAACTGTCCCTGGGGGTCTGgATCCGTACCCCTCTTTTTCTGGCAACAGTATTATTTTCAAATACACTACAGAAAATAAGCAAGAATGAGCTACTGCATCACCCCATGGGGGTCC
ST-E00192:51:H2KG3CCXX:6:1222:18640:39440+   CTGCTCCCAGTTCTTTCCTACACAAGATCCGGGAACTGTCCCTGGGGGTCTGGATCCGTACCCCTCTTTTTCTGGCAACAGTATTATTTTCAAATACACTACAGAAAATAAGCAAGAATGAGCTACTGCATCACCCCATGGGGGTCCCTG
ST-E00192:51:H2KG3CCXX:6:1222:18559:39476+   CtGCTCCCAGTTCTTTCCTACaCAAGATCCGGGAACTGTcCCTGgGGGTCTGGATCCGTACCCCTCTTTTTCTGGCAacaGTAtTaTTTTCcAaTcCACTaCAGAAaATaagCAAGAATGcGCTACTGCATCACCccAtGGGGGTacCTG
ST-E00192:51:H2KG3CCXX:3:2201:9475:25956 +   GCTCCCAGTTCTTTCCTACACAAGATCCAGGAACTGTCCCTGGGGGTCTGGATCCGTACCCCTCTTTTTCTGGCAACAGTATTATTTTCAAATACACTACAGAAAATAAGCAAGAATGAGCTACTGCATCaCCCCATGGGGGTCCCTGCA
ST-E00192:51:H2KG3CCXX:1:2211:7902:71753 - TTATAAGTGGAAACACTAACAAAGGAGGGTGCGTTCCTCCTCCTGCTTTCTGAGGATGCCCTACTCTGTAACAAAGGAGTTTCCAATGCATTTACTTCTTTCATTGTTCTCTGTGACCTGCTCCCAGTTCTTTCCTACACAAGATCCGGG
ST-E00192:51:H2KG3CCXX:8:2211:31643:31688-   gTGGAAACACTAACAAAGgGAggGTGCGTTCCTCCTCCTGCTtTCTGAGGAtgCCCTACTCTGTAACAaaggAGTTTCCAATGCATTTACTTCTTTCATTGTTCTCcGTGACCTGCTCCCAGTTCTTTCCTACACAAGATCCAGGAACTGT
ST-E00192:51:H2KG3CCXX:2:1207:26953:10591-   AAACACTAACAAAGGAGGGTGCGTTCCTCCTCCTGCTTTCTGAGGATGCCCTACTCTGTAACAAAGGAGTTTCCAATGCATTTACTTCTTTCATTGTTCTCTGTGACCTGCTCCCAGTTCTTTCCTACACAAGATCCGGGAACTGTCCCT
ST-E00192:51:H2KG3CCXX:1:1103:12307:59869-   GAGGGTGcGttcCtCCgCCTGcTTTCTGAgggTGCCCTACgCTGTAACAAAGGAGTTTCCAATGCATTTACTTCTtTCATTGTTCTCTGTGACCTGCTCCCAGTTCTTTCCTaCACaAGATCCGGGAACTGTCCCTGGGGGTCTGGATCC
ST-E00192:51:H2KG3CCXX:1:2117:24842:68255-   TCCTCCTGCTTTCTgAGGATGCCCTACTCTGTAACAAAGGAGTTTCCAATGCATTTACTTCTTTCATTGTTCTCTGTGACCTGCTCCCAGTTCTTTCCTACACAAGATCCAGGAACTGTCCCTGGGGGTCTGGATCCGTACCCCTCTTTT
ST-E00192:51:H2KG3CCXX:1:2117:24832:68272-   TCCTCCTGCTTTCTGAGGATGCCCTACTCTGTAACAAAGGAGTTTCCAATGCATTTACTTCTTTCATTGTTCTCTGTGACCTGCTCCCAGTTCTTTCCTACACAAGATCCAGGAACTGTCCCTGGGGGTCTGGATCCGTACCCCTCTTTT
ST-E00192:51:H2KG3CCXX:1:2117:24852:68272-   TCCTCCTGCTTtCTGAGGATGCCCTACTCTGTAACAAAGGAGTTTCCAATGCATTTACTTCTTTCATTGTTCTCTGTGACCTGCTCCCAGTTCTTTCCTACACAAGATCCAGGAACTGTCCCTGGGGGTCTGGATCCGTACCCCTCTTTT
ST-E00192:51:H2KG3CCXX:1:2117:24578:68290-   TCCTCCTGCTTTCTGAGGATGCCCTACTCTGTAACAAAGGAGTTTCCAATGCATTTACTTCTTTCATTGTTCTCTGTGACCTGCTCCCAGTTCTTTCCTACACAAGATCCAGGAACTGTCCCTGGGGGTCTGGATCCGTACCCCTCTTTT
ST-E00192:51:H2KG3CCXX:1:2117:24974:68342-   TCCTCCTGCTTTCTGAGGATGCCCTACTCTGTAACAAAGGAGTTTCCAATGCATTTACTTCTTTCATTGTTCTCTGTGACCTGCTCCCAGTTCTTTCCTACACAAGATCCAGGAACTGTCCCTGGGGGTCTGGATCCGTACCCCTCTTTT
ST-E00192:51:H2KG3CCXX:5:1223:4583:56265 -   TCCTCCTGCTTTCTGAGGATGCCCTACTCTGTAACAAAGGAGTTTCCAATGCATTTACTTCTTTCATTGTTCTCTGTGACCTGCTCCCAGTTCTTTCCTACACAAGATCCAGGAACTGTCCCTGGGGGTCTGGATCCGTACCCCTCTTTT
ST-E00192:51:H2KG3CCXX:5:1210:13707:19469-   CcCTACTCTGTAACAAaGGAGTTTCCAATGCATTTACTTCTTTCATTGTTCTCTGTGACCTGCTCCCAGTTCTTTCCTACACAAGATCCGGGAACTGTCCCTGGGGGTCTGGATCCGTACCCCTCTTTTTCTGGCAACAGTATTATTTTC
ST-E00192:51:H2KG3CCXX:6:2122:21665:26079-   CTACTCTGTAACAAAGGAGTTTCCAATGCATTTACTTCTTTCATTGTTCTCTGTGACCTGCTCCCAGTTCTTTCCTACACAAGATCCGGGAACTGTCCCTGGGGGTCTGGATCCGTACCCCTCTTTTTCTGGCAACAGTATTATTTTCAA
ST-E00192:51:H2KG3CCXX:6:2109:20325:60748-   TCTGTAACaacGGAGTTTCCAATGCATTTACTTCTTTCATTGTTCTCTGTGACCTGCTCCCAGTTCTTTCCTACACAAGATCCAGGAACTGTCCCTGGGGGTCTGGATCCGTACCCCTCTTTTTCTGGCAACAGTATTATTTTCAAATAC
ST-E00192:51:H2KG3CCXX:5:1111:25096:27943-   GAGTTTCCAATGCATTTACTTCTTTCATTGTTCTCTGTGACCTGCTCCCAGTTCTTTCCTACACAAGATCCGGGAACTGTCCCTGGGGGTCTGGATCCGTACCCCTCTTTTTCTGGCAACAGTATTATTTTCAAATACACTACAGAAAAT
ST-E00192:51:H2KG3CCXX:2:2105:19686:27257-   AATGCATTTACTTCTTTCATTGTTCTCTGTGACCTGCTCCCAGTTCTTTCCTACACAAGATCCGGGAACTGTCCCTGGGGGTCTGGATCCGTACCCCTCTTTTTCTGGCAACAGTATTATTTTCAAATACACTACAGAAAATAAGCAAGA
ST-E00192:51:H2KG3CCXX:2:2105:19605:27503-   AATGCATTTACTTCTTTCATTGTTCTCTGTGACCTGCTCCCAGTTCTTTCCTACACAAGATCCGGGAACTGTCCCTGGGGGTCTGGATCCGTACCCCTCTTTTTCTGGCAACAGTATTATTTTCAAATACACTACAGAAAATAAGCAAGA
ST-E00192:51:H2KG3CCXX:8:2213:32850:63807-   atTtaCtTCTTTCATTGTTCtctgTGACCTgCTCCCAGTTCTTTCCTACACAAgATCCAGGAACTGTCCCTGGGGgTCTGGATCCGTACCCCTCTTTTTCTGGCAACAGTATTATTTTCAAATACACTACAGAAAATAAGCAAGAATGAG
ST-E00192:51:H2KG3CCXX:8:2213:32688:63912-   ATtTACTTCTTTCattGTTCTCTgtgACCTGCtCCCAgTTCTTTCCTACACAAGatCCAGGAACTgTCCCTgggGGTCTGGATCCGTACCCCTCTTTTTCTGGCAACAGTATTATTTTCAAATACACTACAGAAAATAAGCAAGAATGAG
ST-E00192:51:H2KG3CCXX:2:1103:8501:65442 -   tttCATTgtTcTcggtgACCTGCTCCCaGTtcttTCCTACaCAAGATCCGGGAACTGTCCCTGgGGGTCTGGatCCGtACCCCTCTgTTTCTGGCAACAGTATTATTTTCaAaTACACTACAGAAAATAAGCAAGAATGAGCTACTGCAt
ST-E00192:51:H2KG3CCXX:8:1205:20863:45646-   TTGTTCTCTGTGACCTGCTCCCAGTTCTTTCCTACACAAGATCCGGGAACTGTCCCTGGGGGTCTGGATCCGTACCCCTCTTTTTCTGGCAACAGTATTATTTTCAAATACACTACAGAAAATAAGCAAGAATGAGCTACTGCATCACC
ST-E00192:51:H2KG3CCXX:3:1121:1791:68167 -   gtggtTCTctgtgccctgctcccaattcttTCCtacactaGatCcGggaAcggtCCCttGGGGtCTGgATCcGtgCCCCTCTttTTCTGGCAACAataTATTTTCAAATACacTACAgAAAATAAGCAAGAATGAGCTACTGCATCACC
ST-E00192:51:H2KG3CCXX:2:2201:13210:6688 -   TtCTCTGTGACCTGCTCCCAGTTCTTTCCTACACAAGATCCGGGAACTGTCCCTGGGGGTCTGGATCCGTACCCCTCTTTTTCTGGCAACAGTATTATTTTCAAATACACTACAGAAAATAAGCAAGAATGAGCTACTGCATCACCCCAT
ST-E00192:51:H2KG3CCXX:5:2118:24598:72228-   ACACAAGATCCGGGAACTGTCCCTGGGGGTCTGGATCCGTaCCCCTCTTTTTCTGGCAACAGTATTATTTTCAAATACACTACAGAAAATAAGCAAGAATGAGCTACTGCATCACCCCATGGGGGTCCCTGCAGTACCAGCTTCCAGGAG
```

```
                          106230060           106230080           106230100           106230120           106230140           106230160           106230180           106230200           106230220           106230240           106230260           106230280           106230300           106230320           106230340
chr10:106230048-106230345 tttttgttcagtctgtggcttacctttttcattttcttaacagtgtattttgaagagcagacatttttgaattttaataaggcccaacttgttcttctttttctgatgtggtttgtactttttcttgttttgtctaagtaatttttgcctactcagatagtaaggaattttgcctgcattttctgtagaagtttgattatagttttagcttttatgttcatatttatgacctatttagagttaattttatgtaagatgtgaggttagaggttcatttaaaaaatttttattttaaatttt

ST-E00144:68:H2LCKCCXX:1:2102:7465:25991 +  TACCTTTTCATTTTCTTAACAGTGTATTTTGAAGAGCAGACATTTTGAATTTTAATAAGGCCCAACTTGTTCTTCTTTTTCTGATGTGGTTTGTACTTTTCTTGTTTTGTCTAAGTAATTTTTGCCTACTCAGATAGTAAGGAATTTTG
ST-E00144:68:H2LCKCCXX:1:1107:13647:72280+  CTTCTTTTTCTGATGTGGTTTGTACTTTTCTTGTTTTGTCTAAGTAATTTTTGCCTACTCAGATAGTAAGGAATTTTGCCTGCATTTTCTGTAGAAGTTTGATTATAGTTTTAGCTTTTATGTTCATATTTATGACCTATTTAGAGTTA
ST-E00144:68:H2LCKCCXX:6:1209:15809:30351+  GGTTTGTACTTTTCTTGTTTTGTCTAAGTAATTTTTGCCTACTCAGATAGTAAGGAATTTTGCCTGCATTTTCTGTAGAAGTTTGATTATAGTTTTAGCTTTTATGTTCATATTTATGACCTATTTAGAGTTAATTTTATGTAAGATGT
ST-E00144:68:H2LCKCCXX:1:2219:20214:23601+  GTACTTTTCTTGTTTTGTCTAAGTAATTTTTGCCTACTCAGATAGTAAGGAATTTTGCCTGCATTTTCTGTAgaagTTTGATTATAGTTTTAGCTTTTATGTTCATATTTATGACCTATTTAGAGTTAATTTTATGTAAGATgTGAGGT
ST-E00144:68:H2LCKCCXX:1:2214:29897:72755+  TTCTTGTTTTGTCTAAGTAATTTTTGCCTACTCAGATAGTAAGGAATTTTGCCTGCATTTTCTGTAGAAGTTTGATTATAGTTTTaGCTTTTATGTTCaTATTTATGACcTaTTTAggtttaaTTTTaTGTAagattttaggttggagg
ST-E00144:68:H2LCKCCXX:6:2124:10013:33569+  ACTCAGATAGTAAGGAATTTTGCCTGCATTTTCTGTAGAAGTTTGATTATAGTTTTAGCTTTTATGTTCATATTTATGACCTATTTAGAGTTAATTTTATGTAAGATGTGAGGTTAGAGGTTCATTTAAAAAATTTTTATTTTAAATTT
ST-E00144:68:H2LCKCCXX:7:1123:5557:53276 -  TGTGgCTTACCTTTTCATTTTCTTAACAGTGTATTTTGAAGAGCAGACATTTTGAATTTTAATAAGGCCCAACTTGTTCTTCTTTTTCTGATGTGGTTTGTACTTTTCTTGTTTTGTCTAAGTAATTTTTGCCTACTCAGATAGTAAGGA
ST-E00144:68:H2LCKCCXX:4:1124:10084:49444-  CCTTTTCATTTTCTTAACAGTGTATTTTGAAGAGCAGACATTTTGAATTTTAATAAGGCCCAACTTGTTCTTCTTTTTCTGATGTGGTTTGTACTTTTCTTGTTTTGTCTAAGTAATTTTTGCCTACTCAGATAGTAAGGAATTTTGCCT
ST-E00144:68:H2LCKCCXX:1:1203:8764:21385 -  TGTATTTTGAAGAGCAGACATTTTGAATTTTAATAAGGCCCAACTTGTTCTTCTTTTTCTGATGTGGTTTGTACTTTTCTTGTTTTGTCTAAGTAATTTTTGCCTACTCAGATAGTAAGGAATTTTGCCTGCATTTTCTGTAGAAgTTT
ST-E00144:68:H2LCKCCXX:8:2217:4644:69696 -  ATTTTGAATTTTAATAAGGCCCAACTTGTTCTTCTTTTTCTGATGTGGTTTGTACTTTTCTTGTTTTGTCTAAGTAATTTTTGCCTACTCAGATAGTAAGGAATTTTGCCTGCATTTTCTGTAGAAGTTTGATTATAGTTTTAGCTTTT
ST-E00144:68:H2LCKCCXX:8:2216:22071:29560-  TTGTTCTTCTTTTTCTGATGTGGTTTGTACTTTTCTTGTTTTGTCTAAGTAATTTTTGCCTACTCAGATAGTAAGGAATTTTGCCTGCATTTTCTGTAGAAGTTTGATTATAGTTTTAGCTTTTATGTTCATATTTATGACCTATTTAG
ST-E00144:68:H2LCKCCXX:4:1101:18955:9431 -  TGgTTTGTACTTTTCTTGTTTTGTCTAAGTAATTTTTGCCTACTCAGATAGTAAGGAATTTTGCCTGCATTTTCTGTAGAAGTTTGATTATAGTTTTAGCTTTTATGTTCATATTTATGACCTATTTAGAGTTAATTTTATGTAAGATG
ST-E00144:68:H2LCKCCXX:1:2203:5861:33129 -  TTTCTTGTTTTGTCTAAGTaATTTTTGCCTACTCAGATAGTAAGGAATTTTgCCTGCAtTTTCTGTAGAAGTTTGATTATAGTTTTAGCTTTTATGTTCATATTTATGACCTATTTAGAGTTAATTTTATGTAAGATGTGAGGTTAGAG
ST-E00144:68:H2LCKCCXX:1:2203:5882:33164 -  TTTCTTGTTTTGTCTAAGTAATTTTTGCCTACTCAGATAGTAAGGAATTTTGCCTGCATTTTCTGTAGAAGTTTGATTATAGTTTTAGCTTTTATGTTCATATTTATGACCTATTTAGAGTTAATTTTATGTAAGATGTGAGGTTAGAG
ST-E00144:68:H2LCKCCXX:6:1206:10693:72579-  TAAGtaATTTTTGCCTACTCAGATAGTAAGGAATTTTGCCTGCATTtTCTGTAGAAGTTTGATTATAGTTTTAGCTTTTATGTTCATAttTATGACCTATTTAGAGTTAATTTTATGTAAGATGTGAGGTTAGAGGtTCATTTAAAAAA
ST-E00144:68:H2LCKCCXX:3:2209:28171:14388-  AAGTAATTTTTGCCTACTCAGATAGTAAGGAATTTTGCCTGCATTTTCTGTAGAAGTTTGATTATAGTTTTAGCTTTTATGTTCATATTTATGACCTATTTAGAGTTAATTTTATGTAAGATGTGAGGTTAGAGGTTCATTTAAAAAAT
ST-E00144:68:H2LCKCCXX:3:2209:28192:14388-  AAGTAATTTTTGCCTACTCAGATAGTAAGGAATTTTGCCTGCATTTTCTGTAGAAGTTTGATTATAGTTTTAGCTTTTATGTTCATATTTATGACCTATTTAGAGTTAATTTTATGTAAGATGTGAGGTTAGAGGTTCATTTAAAAAAT
ST-E00144:68:H2LCKCCXX:3:1204:3862:72667 -  ttTTGCCTACTaAGATAGTAAGGAATtTTgCCTGCATTTTCTGTAGAAGTTTGATTATAGTTTTAGCTTTTATGTTCATATTTATGACCTATTTAGAGTTAATTTTATGTAAGATGTGAGGTTAGAGGTTcATTTAAAAAATTTTTATT
ST-E00144:68:H2LCKCCXX:7:2104:21229:46279-  CTCAGcTAGTAAGGAaTTTTGCCTGCATTTTCTGTAGCAGTTTGATTATcGTTTTAGCTTTTATGTTCATATTTATGACCTATTTAGAGTTAATTTTcTGTAcGATGTGAGGTTcGAggTTCATTTAAAAAATTTTTATTTTAAATTT
```

```
ST-E00192:51:H2KG3CCXX:6:1123:13687:30967+  AGTCTGTGGCTTACCTTTTCATTTTCTTAACAGTGTATTTTGAAGaGCAGACATTTTGAATTTTAATAAgGCCCaACTTGTTCTTCTTTTTCTGATGTGGtTTGTAcTTTTCTTGTTTTGTCTaAGTAATTTTTGCCTACTCagaTagTa
ST-E00192:51:H2KG3CCXX:6:1123:13707:30967+  AGTCTGTGGCTTACCTTTTCATTTTCTTAACAGTGTATTTTGAAGAGCAGACATTTTGAATTTTAATAAgGCCCAACTTGTTCTTCTTTTTCTGATGTGGTTTGTACTTTTCTTGTTTTGTCTAAGTAATTTTTGccTACTCAGaTAGTA
ST-E00192:51:H2KG3CCXX:2:2108:8206:12929 +  ATTTTCTTAACAGTGTATTTTGAAGAGCAGACATTTTGAATTTTAATAAGGCCCAACTTGTTCTTCTTTTTCTGATGTGGTTTGTACTTTTCTTGTTTTGTCTAAGTAATTTTTGCCTACTCAGATAGTAAGGGTGGAACCATAGAATGA
ST-E00192:51:H2KG3CCXX:6:2103:6927:4878  +  AGTGTATTTTGAAGAGCAGACATTTTGAATTTTAATAAGGCCCAACTTGTTCTTCTTTTTCTGATGTGGTTTGTACTTTTCTTGTTTTGTCTAAGTAATTTTTGCCTACTCAGATAGTAAGGAATTTTGCCTGCATTTTCTGTAGAAGT
ST-E00192:51:H2KG3CCXX:3:2214:26750:40548+  GTATTTTGAAGAGCAGACATTTTGAATTTTAATAAGGCCCAACTTGTTCTTCTTTTTCTGATGTGGTTTGTACTTTTCTTGTTTTGTCTAAGTAATTTTTGCCTACTCAGATAGTAAGGAATTTTGCCTGCATTTTCTGTAGAAGTTTG
ST-E00192:51:H2KG3CCXX:5:1221:4846:60590 +  AAGAGCAGACATTTTGAATTTTAATAAGGCCCAACTTGTTCTTCTTTTTCTGATGTGGTTTGTACTTTTCTTGTTTTGTCTAAGTAATTTTTGCCTACTCAGATAGTAAGGAATTTTGCCTGCATTTTCTGTAGAAGTTTGATTATAGT
ST-E00192:51:H2KG3CCXX:2:1111:10906:36733+  AGCAGACATTTTGAATTTTAATAAGGCCCAACTTGTTCTTCTTTTTCTGATGTGGTTTGTACTTTTCTTGTTTTGTCTAAGTAATTTTTGCCTACTCAGaTAGTAAGGAATTTTGCCTGCATTTTCTGTAGAAGTTTGATTATAGTTTT
ST-E00192:51:H2KG3CCXX:2:1111:10865:36803+  AGCAGACATTTTGAATTTTAATAAGGCCCAACTTGTTCTTCTTTTTCTGATGTGGTTTGTACTTTTCTTGTTTTGTCTAAGTAATTTTTGCCTACTCAGATAGTAAGGAATTTTGCCTGCATTTTCTGTAGAaGTTtGATTATAGTTTT
ST-E00192:51:H2KG3CCXX:6:2211:14763:22598+  CATTTTGAATTTTAATAAGGCcCAACTtGtTcTTCttTTtcTGATgTgGTTTGTACTTTTCTTGTTTTGTCTAAGTAtTtTTGCCTACTcaGATAGTAaGGAATTTTGccTgCAttTTTCtgTaGAAGTTTGATTATAGTTTTAGCTTT
ST-E00192:51:H2KG3CCXX:2:1208:26009:10644+  TTGAATTTTAATAAGGCCCAACTTGTTCTTCTTTTTCTGATGTGGTTTGTACTTTTCTTGTTTTGTCTAAGTAATTTTTGCCTACTCAGATAGTAAGGAATTTTGCCTGCATTTTCTGTAGAAGTTTGATTATAGTTTTAGCTTTTAT
ST-E00192:51:H2KG3CCXX:6:1104:16895:14512+  CCAACTTGTTCTTCTTTTTCTGATGTGGTTTGTACTTTTCTTGTTTTGTCTAAGTAATTTTTGCCTACTCAGATAGTAAGGaatTtTGCCTGCATTTTCTGTAGAAGttTGaTTATAGTTTTAGCTTTTATGTTCaTAtTTATGAC
ST-E00192:51:H2KG3CCXX:8:1216:8531:14512 +  CTTTTTCTGATGTGGTTTGTACTTTTCTTGTTTTGTCTAAGTAATTTTTGCCTACTCAGATAGTAAGGAATTTTGCCTGCATTTTCTGTAGAAGTTTGATTATAGTTTTAGCTTTTATGTTCATATTTATGacCTATtTAGAGTTAATT
ST-E00192:51:H2KG3CCXX:3:1113:7049:67921 +  GTAATTTTTGCCTACTCAGaTAGTAAGGAATTTTGCCTGCATTgTTctGcAgAAGtTTGaTTATAGatttAGctTTTATGTTCATATTTATGACctaTTTAGagtTAATTTTATGTAaGATGTGAGGgtAGAGGtTCAtttAAAAAATTT
ST-E00192:51:H2KG3CCXX:3:1113:7059:67938 +  GTAATTTTTGCCTACTCAGATAGTAAGGAATTTTGCCTGCATTTTCTGTAGAAGtTTGATTATAGTTTTAGCTTTTATGTTCATATTTATGACCTATTTAGAGTTAATTTTATGTAAGATGTGAGGTTAGAGGTTCATTTAAAAAATTT
ST-E00192:51:H2KG3CCXX:8:1112:14530:65793+  TGCCTACTCaGATAGTAaGGAAtTTGCCTGCATTTTCTGTAGaaGctTGaTTaTAGTTTTAGCtTTTATGTTCATATTTATGaCCTATTTAGaGTTAaTTTTATGTAaGATGTGAGgtTAGagGTTcaTTTAaAcAATTTtTATTTTA
ST-E00192:51:H2KG3CCXX:1:1117:28313:42517+  TACTCAGATAGTAAGGAATTTTGCCTGCATTTTCTGTAGAAGTTTGATTATAGTTTTAGCTTTTATGTTCATATTTATGACCTATTTAGAGTTAATTTTATGTAAGATGTGAGGTTAGAGGTTCATTTAAAAAATTTTTATTTTAAATT
ST-E00192:51:H2KG3CCXX:1:1117:28283:42535+  TACTCAGATAGTAAGGAATTTTGCCTGCATTTTCTGTAGAAGTTTGATTATAGTTTTAGCTTTTATGTTCATATTTATGACCTATTTAGAGTTAATTTTATGTAAGATGTGAGGTTAGAGGTTCATTTAAAAAATTTTTATTTTAAATT
ST-E00192:51:H2KG3CCXX:8:1114:30800:6952 - TTTTTGTTCAGTCTGTGGCTTACCTTTTCATTTTCTTAACAGTGTATTTTGAAGAGCAGACATTTTGAATTTTAATAAGGCCCAACTTGTTCTTCTTTTTCTGATGTGGTTTGTACTTTTCTTGTTTTGTCTAAGTAATTTTTGCCTACT
ST-E00192:51:H2KG3CCXX:6:2219:24832:37331-  GTCTGTGGCTTACCTTTTCATTTTCTTAACAGTGTATTTTGAAGAGCAGACATTTTGAATTTTAATAAGGCCCAACTTGTTCTTCTTTTTCTGATGTGGTTTGTACTTTTCTTGTTTTGTCTAAGTAATTTTTGCCTACTCAGATAGTAA
ST-E00192:51:H2KG3CCXX:5:1220:13281:26994-  TAACAGTGTATTTTGAAGAGCAGACATTTTGAATTTTAATAAGGCCCAACTTGTTCTTCTTTTTCTGATGTGGTTTGTACTTTTCTTGTTTTGTCTAAGTAATTTTTGCCTACTCAGATAGTAAGGAATTTTGCCTGCATTTTCTGTAG
ST-E00192:51:H2KG3CCXX:2:2210:5598:8587  -  ACTTGTTCTTCTTTTTCTGATGTgGTTTGTACTTTTCTTGTTTTGTCTAAGTAATTTTTGCCTACTCAGATAGTAAGGAATTTTGCCTGCATTTTCTGTAGAAGTTTGATTATAGTTTTAGCTTTTATGTTCATATTTATGACCTATTT
ST-E00192:51:H2KG3CCXX:2:2210:5527:8605  -  ACTTGTTCTTCTTTTTCTGATGTGGTTTGTACTTTTCTTGTTTTGTCTAAGTAATTTTTGCCTACTCAGATAGTAaGGAATTTTGCCTGCATTTTCTGTAGAAGTTTGATTATAGTTTTAGCTTTTATGTTCATATTTATgACCTATTT
ST-E00192:51:H2KG3CCXX:2:2210:6024:9607  -  ACTTGTTCTTCTTTTTCTGATGTGGTTTGTACTTTTCTTGTTTTGTCTAAGTAATTTTTGCCTACTCAGATAGTAAGGAATTTTGCCTGCATTTTCTGTAGAAGTTTGATTATAGTTTTAGCTTTTATGTTCATATTTATGACCTATTT
ST-E00192:51:H2KG3CCXX:2:1108:28486:10468-  CTGATGTGGTTTGTACTTTTCTTGTTTTGTCTAAGTAATTTTTGCCTACTCAGATAGTAAGGAATTTTGCCTGCATTTTCTGTAGAAGTTTGATTATAGTTTTAGCTTTTATGTTCATATTTATGACCTATTTAGAGTTAATTTTATGT
ST-E00192:51:H2KG3CCXX:6:1122:10399:28435-  CTGATGTGGTTTGTACTTTTCTTGTTTTGTCTAAGTAATTTTTGCCTACTCAGATAGTAAGGAATTTTGCCTGCATTTTCTGTAGAAGTTTGATTATAGTTTTAGCTTTTATGTTCATATTTATGACCTATTTAGAGTTAATTTTATGT
ST-E00192:51:H2KG3CCXX:6:2113:7313:18555 -  TACTTTTCTTgTTTTGTCTAAGTAATTTTTGCCTACTCAGATAGTAAGGAATTTTGCCTGCATTTTCTGTAGAAGTTTGATTATAGTTTTAGCTTTTATGTTCATATTTATGACCTATTTAGAGTTAATTTTATGTAAGATGTGAGGTT
ST-E00192:51:H2KG3CCXX:6:1107:21208:33199-  CTTTTCTTGTTTTGTCTAAGTAATTTTTGCCTACTCAGATAGTAAGGAATTTTGCCTGCATTTTCTGTAGAAGTTTGATTATAGTTTTAGCTTTTATGTTCATATTTATGACCTATTTAGAGTTAATTTTATGTAAGATGTGAGGTTAG
ST-E00192:51:H2KG3CCXX:2:1113:18691:35625-  TCTTGTTTTGTCTAAGTAATTTTTGCCTACTCAGATAGTAAGGAATTTTGCCTGCATTTTCTGTAGAAGTTTGATTATAGTTTTAGCTTTTATGTTCATATTTATGACCTATTTAGAGTTAATTTTATGTAAGATGTGAGGTTAGAGGT
ST-E00192:51:H2KG3CCXX:2:1204:27014:33235-  TTTTGTCTAAGTAATTTTTGCCTACTCAGATAGTAAGGAATTTTGCCTGCATTTTCTGTAGAAGTTTGATTATAGTTTTAGCTTTTATGTTCATATTTATGACCTATTTAGAGTTAATTTTATGTAAGATGTGAGGTTAGAGGTTCATT
ST-E00192:51:H2KG3CCXX:3:1211:25553:29648-  CTAAGTAATTTTTGCCTACTCAGATAGTAAGGAATTTTGCCTGCATTTTCTGTAGAAGTTTGATTATAGTTTTAGCTTTTATGTTCATATTTATGACCTATTTAGAGTTAATTTTATGTAAGATGTGAGGTTAGAGGTTCATTTAAAAA
ST-E00192:51:H2KG3CCXX:8:1202:30242:13298-  CTcAGATAGTaAGGAATTTTGCCTGCATTTTCTGTAGAAGTTTGATTATAGTTTTAGCTTTTATGTTCATATTTATGACCTATTTAGAGTTAATTTTATGTAAGATGTGAGGTTAGAGGTTCATTTAAAAAATTTTTATTTTAAATTTT
ST-E00192:51:H2KG3CCXX:8:1202:30435:13914-  CTCAGATAGTAAGGAATTTTGCCTGCATTTTCTGTAGAAGTTTGATTATAGTTTTAGCTTTTATGTTCATATTTATGACCTATTTAGAGTTAATTTTATGTAAGATGTGAGGTTAGAGGTTCATTTAAAAAATTTTTATTTTAAATTTT
```

---

chr10 104335895 + 4 chr11 91445205 + 1 CTX 33 50 96 52 0.794358974358974 0 0.727606899902818 0 2 chr10 104335840 111 chr11 91445238

ATGATCCACCCACCTCACCCTCCCAAAGTGCTGGGATTACAGGCATGAGGTACTGCAGCCAGCCAGGATTTTAACTTACAAATTTGGGGGGAACACAAACATTCAGTCTATACCTACCTTGAAAAAAAAGAATACACCTTTTAGATGGATATGTGTTGTTCTTCTTAGATTCATC

```
                           104335760           104335780           104335800           104335820           104335840           104335860           104335880           104335900           104335920           104335940           104335960           104335980           104336000           104336020           104336040
chr10:104335754-104336043  ccgctaattttttctgtg*************tgtagagatggggtcttggtatgttgtccaggctggtcttgaacttctggactgaagtgatccacccacctcagcctcccaaagtgctgggattacaggcatgaggtactgcaactgaccTCAGATGttaaaaacttagactatacagataaagtcaaagttctctgtgaccaccccacccaatctcagggccctcgctaaagaaaccctctttatccatttggtgtgcgtccttgcagcttgccagatttttttctatgt

ST-E00144:68:H2LCKCCXX:1:1110:16011:2926 +  CGCTAATTTTTCTGTG**TTTTTTTTTTTTGTAGAGATGGGGTCTTGGTATGTTGTCCAGGCTGGTCTTGAACTTCTGGaCTGAAGTGATCCACCCACCTCAGCCTCCCAAAGTGCTGGGATTACAGGCATGAGGTACTgCAACTGACCTCA
ST-E00144:68:H2LCKCCXX:6:1220:19747:59992+  AATTTTTCTGTG*TTTTTTTTTTTTgTttagATGGGGTcTTGGTaTGTTgTCCAGGCTGGTCTTGAACTTtTGGaCTgAAGTGATCCACCCACCTCAGCCTCCCAAAGTgCtGGGAtTaCAGGCATGagGTACTgCAACTGacCtcAGaT
ST-E00144:68:H2LCKCCXX:4:2217:12337:64633+  TTTTTTTGTAGAGATGGGGTCTTGGTATGTTGTCCAGGCTGGTCTTGAACTTCTGGACTGAAGTGATCCACCCACCTCAGCCTCCCAAAGTGCTGGGATTACAGGCATGAGGTACTGCAACTGACCTCAGATGTTAAAAACTTAGACTA
ST-E00144:68:H2LCKCCXX:7:2124:30435:66866+  GTAGAGATGGGGTCTTGGTATGTTGTCCAGGCTGGTCTTGAACTTCTGGACTGAAGTGATCCACCCACCTCAGCCTCCCAAAGTGCTGGGATTACAGGCATGAGGTACTGCAACTGACCTCAGATGTTAAAAACTTAGACTATACAGATA
ST-E00144:68:H2LCKCCXX:8:1217:15565:67165+  TAGAGATGGGGTCTTGGTATGTTGTCCAGGCTGGTCTTGAACTTCTGGACTGAAGTGATCCACCCACCTCAGCCTCCCAAAGTGCTGGGATTACAGGCATGAGGTACTGCAACTGACCTCAGATGTTAAAAACTTAGACTATACAGATAA
ST-E00144:68:H2LCKCCXX:1:1218:4907:19100 +  GATGGGGTCTTGGTATGTTGTCCAGGCTGGTCTTGAACTTCTGGACTGAAGTGATCCaCCCACCTCAGCCTCCCAAAGTGCTGGGATTACAGGCATGAGGTACTGCAACTGACCTCAGATGTTAAaACTTaGACTATACAGATAAAGT
ST-E00144:68:H2LCKCCXX:6:1121:18945:38175+  CCAGGCTGGTCTTGAACTTCTGGACTGAAGTGATCCACCCACCTCAGCCTCCCAAAGTGCTGGGATTACAGGCATGAGGTACTGCAACTGACCTCAGaTGtTAAAAACTTAGACTATACAGATaAAGTCAAAGTTCTCTGTGACCACCCC
ST-E00144:68:H2LCKCCXX:4:2224:26771:70329+  ATGATCCACCCACCTCACCCTCCCAAAGTGCTGGGATTACAGGCATGAGGTACTGCAGCCAGCCAGGATTTTAACTTACAAATTTGGGGGGAACACAAACATTCAGTCTATACCTACCTTGAAAAAAAAGAATACACCTTTTAGAtGGAt
ST-E00144:68:H2LCKCCXX:8:1110:16255:17588+  GATCCACCCACCTCAGCCTCCCAAAGTGCTGGGATTACAGGCATGaggTaCTtcAACTGACCTCAGATGTTAAAAACTTAGACTATACAGATAAAGTCAAAGTTCTCTGTGACCACCCCACCCAATCTCAGGGCCCTCGCTAAAGAAACC
ST-E00144:68:H2LCKCCXX:1:2104:22954:36891+  ATCCACCCACCTCAGCCTCCCAAAGTGCTGGGATTACAGGCATGAGGTACTGCAACTGACCTCAGATGTTAAAAACTTAGACTATACAGATAAAGTCAAAGTTCTCTGTGACCACCCCACCCAATCTCAGGGCCCTCGCTAAAGAAACCC
ST-E00144:68:H2LCKCCXX:6:2107:13403:26994+  ATCCACCCACCTCACCCTCCCAAAGTGCTGGGATTACAGGCATGAGGTACTGCAGCCAGCCAGGATTTTAACTTACAAATTTGGGGGGAACACAAACATTCAGTCTATACCTACCTTGAAAAAAAAGAATACACCTTTTAGATGGATATG
ST-E00144:68:H2LCKCCXX:7:1111:13210:2082 +  ACCCACCTCAGCCTCCCAAAGTGCTGGGATTACAGGCATGAGGTACTGCAACTGACCTCAGATGTTAAAAACTTAGACTATACAGATAAAGTCAAAGTTCTCTGTGACCACCCCACCCAATCTCAGGGCCCTCGCTAAAGAAACCCTCTT
ST-E00144:68:H2LCKCCXX:8:1119:12824:1238 +  CTCCCAAAGTGCTGGGATTACAGGCATGAGGTACTGCAACTGACCTCAGATGTTAAAAACTTAGACTATACAGATAAAGTCAAAGTTCTCTGTGACCACCCCACCCAATCTCAGGGCCCTCGCTAAAGAAACCCTCTTTATCCATTTGGT
ST-E00144:68:H2LCKCCXX:4:2220:16083:20981+  TaCAGGCATGAGGTACTGCAACTGACCTCAGaTGTTAAaACTTAGACTATACAGATAAAGTCAAAGTTCTCTGTGACCACCCCACCCAATCTCAGGGccCTCGCTAaAGAAACCCTCTTTATCCATTTGGtgTGCGTCCTTGCAGCTTG
ST-E00144:68:H2LCKCCXX:8:1102:28669:6741 +  CATGAGGTACTGCAACTGACCTCAGctGTTAAAAACTTAGACTATACAGATAAAGTCAAAGTTCTCTGTGACCACCcCACCCAATCTCAGGGCCCTCGCTAAAGAAACCCTCTTTATCCATtTggTGTgCGTCCTTGCAGCTTGCCAtat
ST-E00144:68:H2LCKCCXX:3:2103:29704:37577+  ATGAGGTACTGCAACTGACCTCAGATGTTAAAAACTTAGACTATACAGATAAAGTCAAAGTTCTCTGTGACCACCCCACCCAATCTCAGGGCCCTCGCTAAAGAAACCCTCTTTATCCATTTGGTGTGCGTCCTTGCAGCTTGCCAGATT
ST-E00144:68:H2LCKCCXX:8:1102:4836:39370 -  gggtTTTTctgtg**ttTtttgttTTTGtagaGATGggctctTgGcatgttgtCCaGgCTggTCcTGAaCgTCTaGACTgAagtGATCCacCCAcCTCAGCCtCCCAaAGTGCTGGGATTACaGGCAtgAGGtACTGCAaCTGACCTCAGaT
```

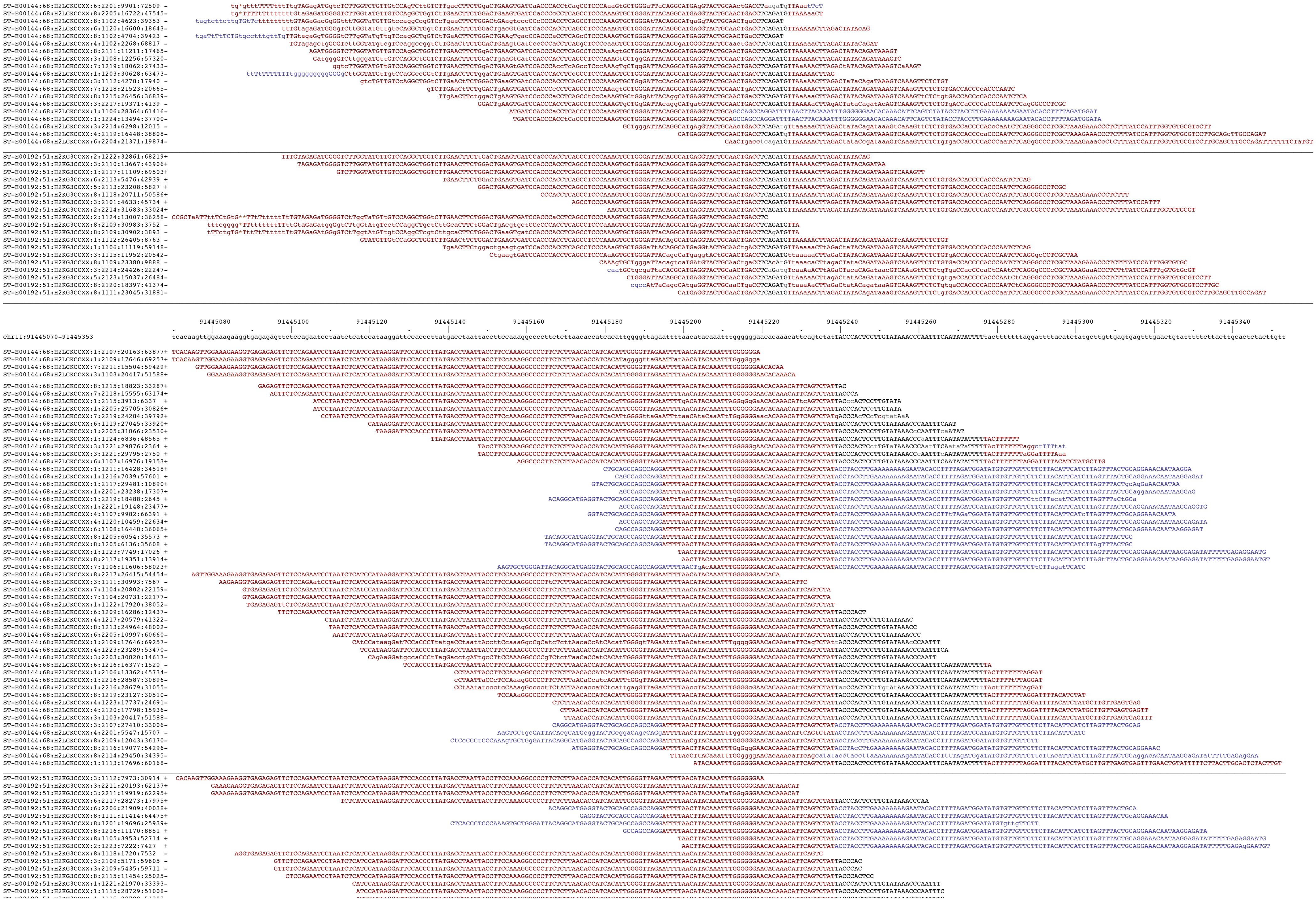

91445080
91445100
91445120
91445140
91445160
91445180
91445200
91445220
91445240
91445260
91445280
91445300
91445320
91445340
chr11:91445070-91445353

```
ST-E00192:51:H2KG3CCXX:1:1115:28780:51307- ATCCATAAGGATTCCACCCTTATGACCTAATTACCTTCCAAAGGCCCCTTCTCTTAACACCATCACATTGGGGTTAGAATTTTAACATACAAATTTGGGGGGAACACAAACATTCAGTCTATTACCCACTCCTTGTATAAACCCAATTTC
ST-E00192:51:H2KG3CCXX:5:1119:6227:64528 - GATTCCACCCTTATGACCTAaTTACCTTCCAAAGGCCCCTTCTCTTAACACCATCACATTGGGGTTAGAATTTTAACATACAAATTTGGGGGGAACACAAACATTCAGTCTATTACCCACTCCTTGTATAAACCCAATTTCAATATATTT
ST-E00192:51:H2KG3CCXX:2:1112:16397:72614- ATTCCACCCTTATGACCTAATTACCTTCCAAAGGCCCCTTCTCTTAACACCATCACATTGGGGTTAGAATTTTAACATACAAATTTGGGGGGAACACAAACATTCAGTCTATTACCCACTCCTTGTATAAACCCAATTTCAATATATTTT

ST-E00192:51:H2KG3CCXX:5:1109:11850:57671- CCTTATGACCTAATTACCTTCCAAAGGCCCCTTCTCTTAACACCATCACATTGGGGTTAGAATTTTAACATACAAATTTGGGGGGAACACAAACATTCAGTCTATTACCCACTCCTTGTATAAACCCAATTTCAATATATTTTTACTTTT
ST-E00192:51:H2KG3CCXX:6:2209:16590:17395- TATTACCTAATTACCTTCCAAAGgCCCCTTCTCTTAACACCATCACATTGGtgTTAGAATTTTAAcATACAAATTTGGGGGGAACACAAACATTCAGTCTATTAcCCACtCCTTGTATAAACCCAATTTCAATATATTTTTACTTTTTTT
ST-E00192:51:H2KG3CCXX:3:2120:5912:34975 - TTCCAAAgGCCCCTTCTCTTAACaCCATCACATTGGGGTTAGAATTTTAACATACAAATTTGGGGGGAACACAAACATTCAGTCTATTACCCACTCCTTGTATAAACCCAATTTCAATATATTTTTACTTTTTTTAGGATTTTACATCTA
ST-E00192:51:H2KG3CCXX:1:1118:20102:70136- TCCAAAgGCCCCTtCTaTTAaCaCcATCacATTGGgGTtAgAATTttAaCATACAAAttTGGGGGGAaCaCaAaCaTtcAGTCtATTACCCACTCCTTGTATAAACCCaaTTTgAAtATATTTTTACTTTTtTTaGGATTTTACATCTAT
ST-E00192:51:H2KG3CCXX:1:1118:20122:70241- TCCAAAGGCCCCTTCTCTTAACACCATCACATTGGGGTTAGAATTTTAACATACAAATTTGGGGGGAACACAAACATTCAGTCTATTACCCACTCCTTGTATAAACCCAATTTCAATATATTTTTACTTTTTTTAGGATTTTACATCTAT
ST-E00192:51:H2KG3CCXX:2:2214:7445:16498 - AAGGCCCCTTCTCTTAACACCATCACATTGGGGTTAGAATTTTAACATACAAATTTGGGGGGAACACAAACATTCAGTCTATTACCCACTCCTTGTATAAACCCAATTTCAATATATTTTTACTTTTTTTAGGATTTTACATCTATGCTT
ST-E00192:51:H2KG3CCXX:6:2105:25228:7251 - CTTAACACCAtCACATTGGGGTTAGAATTTTaaCATACAAATTTGGTGGGAACaCAaACATTCAGTCTATTACCCACTCcTTGTATAAACCCAATTTCAaTAtaTTTTTACTTTTTTTAGGATTTTACATCTATGCTTGTTGAGTGAGTT
ST-E00192:51:H2KG3CCXX:5:1113:14144:19030- AGGCATGAGGTACTGCAGCCAGCCAGGATTTTAACTTACAAATTTGGGGGGAACACAAACATTCAGTCTATACCTACCTTGAAAAAAAAGAATACACCTTTTAGATGGATATGTGTTGTTCTTCTTACATTCATCTTAGTTTACTGCAGG
ST-E00192:51:H2KG3CCXX:6:1115:23228:36065- CTGGGATTACAGGCATGAGGTACTGCAgCCAGCCAGGATTTTAACTTACAAATTTGGGGGGAACACAAACATTCAGTCTATACCTACCTTGAAAAAAAAGAATACACCTTTTAGATGGATATGTGTTGTTCTTCTTACATTCATCTTAGT
ST-E00192:51:H2KG3CCXX:8:1202:25735:34676- GCCAGcCAGGATTTTAACTTACAAATTtGGGGGGAACACAAACATTCAGTCTATACCTACCTTGAAAAAAAAGAATACACCTTTTAGATGGATATGTGTTGTTCTTCTTACATTCATCTTAGTTTACTGCAGGAAACAATAAGGAGATAT
ST-E00192:51:H2KG3CCXX:8:1218:29734:72720- TACTGCAGCCAGCCAGGATTTTAACTTACAAATTTGGGGGGAACACAAACATTCAGTCTATACCTACCTTGAAAAAAAAGAATACACCTTTTAGATGGATATGTGTTGTTCTTCTTACATTCATCTTAGTTTACTGCAGGAAACAATAAG
ST-E00192:51:H2KG3CCXX:3:2219:22579:26431- TAACTTACAAATTTGGGGGGAACACAAACATTCAGTCTATACCTACCTTGAAAAAAAAGAATACACCTTTTAGATGGATATGTGTTGTTCTTCTTACATTCATCTTAGTTTACTGCAGGAAACAATAAGGAGATATTTTTGAGAGGAATG
ST-E00192:51:H2KG3CCXX:8:2119:21919:69731- TAACTTACAAATTTGgGGgGAACACAAACATTCAGTCTATACCTACCtTgAAAAAAAAGAATACACCTTTTAGATGGATATGTGTTGTTCTTCTTACATTCATCTTAGTTTACTGCAGGAAACAATAAGGAGATATTTTTGAGAGGAATG
ST-E00192:51:H2KG3CCXX:2:2217:22335:23337- ATACAAATTTGGGGGGAACACAAACATTCAGTCTATTACCCACTCCTTGTATAAACCCAATTTCAATATATTTTTACTTTTTTTAGGATTTTACATCTATGCTTGTTGAGTGAGTTTGAACTGTATTTTTCTTACTTGCACTCTACTTGT
ST-E00192:51:H2KG3CCXX:2:1108:22903:40073- TACAAATTTGGGgGGAACACAAACATTCAGTCTATTACCCACTCCTTGTATAAACCCAATTTCAATATATTTTTACTTTTTTTAGGATTTTACATCTATGCTTGTTGAGTGAGTTTGAACTGTATTTTTCTTACTTGCACTCTACTTGTT
```
