# Supplementary material for: Genome and epigenome analysis of monozygotic twins discordant for congenital heart disease
Source: BMC Genomics. 2018 Jun 4;19:428. doi: 10.1186/s12864-018-4814-7 (PMC5987557; doi:10.1186/s12864-018-4814-7)
Supplement: Supplementary file 17 — Figure S8. Aberrant methylation in the upstream regions of NSD1. Visualizing the methylation levels of DMRs near NSD1 with UCSC genome browser. Methylated levels in the twins are showed in blue (D3) and red (D4). Transcription factor binding sites are also showed in zooming-in panels, which indicated by black bars with names marked in front. An arrow gives TSS and transcriptional orientation. Transcription factor binding sites, Pol II ChIP-seq and TBP ChIP-seq data from ENCODE. (PDF 781 kb) [file 12864_2018_4814_MOESM17_ESM.pdf]

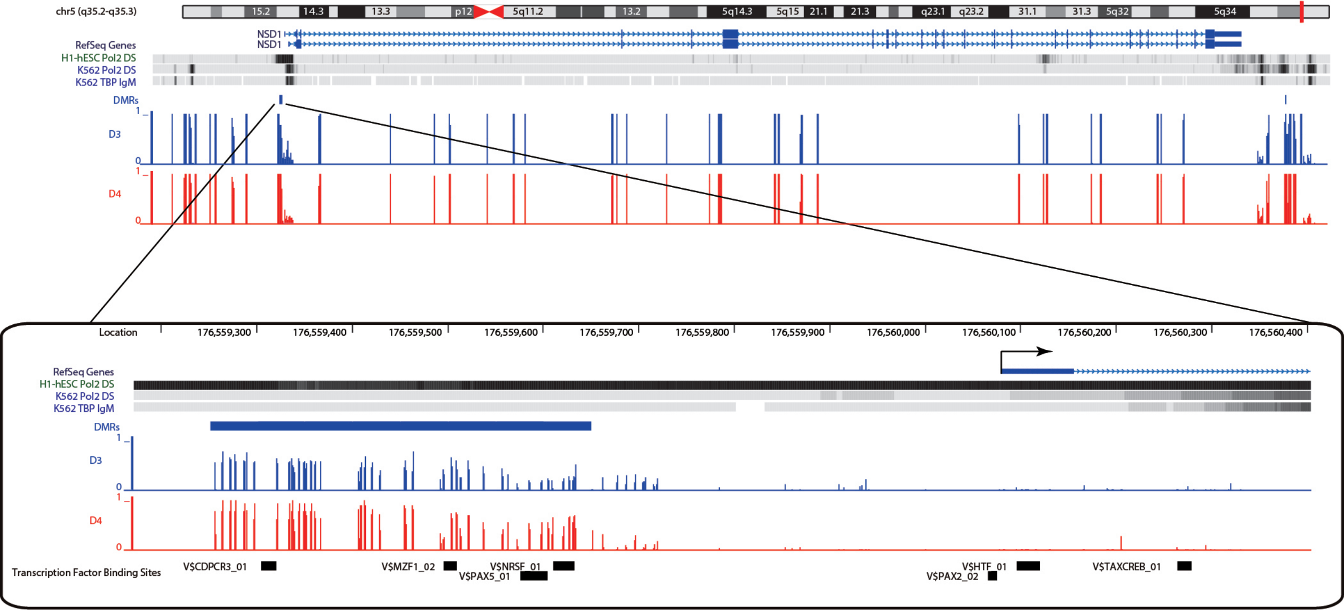

chr5 (q35.2-q35.3)
15.2
14.3
13.3
p12
5q11.2
13.2
5q14.3
5q15
21.1
21.3
q23.1
q23.2
31.1
31.3
5q32
5q34
NSD1
NSD1
RefSeq Genes
H1-hESC Pol2 DS
K562 Pol2 DS
K562 TBP IgM
DMRs
D3
D4
Location
176,559,300
176,559,400
176,559,500
176,559,600
176,559,700
176,559,800
176,559,900
176,560,000
176,560,100
176,560,200
176,560,300
176,560,400
RefSeq Genes
H1-hESC Pol2 DS
K562 Pol2 DS
K562 TBP IgM
DMRs
D3
D4
Transcription Factor Binding Sites
V$CDPCR3_01
V$MZF1_02
V$NRSF_01
V$PAX5_01
V$HTF_01
V$PAX2_02
V$TAXCREB_01
